# Improved herbicide discovery using physico-chemical rules refined by antimalarial library screening†

**DOI:** 10.1039/d1ra00914a

**Published:** 2021-02-23

**Authors:** Kirill V. Sukhoverkov, Maxime G. Corral, Julie Leroux, Joel Haywood, Philipp Johnen, Trevor Newton, Keith A. Stubbs, Joshua S. Mylne

**Affiliations:** The University of Western Australia, School of Molecular Sciences 35 Stirling Highway, Crawley Perth 6009 Australia joshua.mylne@uwa.edu.au keith.stubbs@uwa.edu.au; The ARC Centre of Excellence in Plant Energy Biology 35 Stirling Highway, Crawley Perth 6009 Australia; BASF SE Speyerer Straße 2 67117 Limburgerhof Germany

## Abstract

Herbicides have physico-chemical properties not unlike orally-delivered human drugs, but are known to diverge in their limits for proton donors, partition coefficients and molecular weight. To further refine rules specific for herbicides, we exploited the close evolutionary relationship between *Plasmodium falciparum* and plants by screening the entire Malaria Box, a chemical library of novel chemical scaffolds with activity against the blood stage of *P. falciparum*. Initial screening against *Arabidopsis thaliana* on agar media and subsequently on soil demonstrated the crucial nature of log *P* and formal charge are to active molecules. Using this information, a weighted scoring system was applied to a large chemical library of liver-stage effective antimalarial leads, and of the six top-scoring compounds, one had potency comparable to that of commercial herbicides. This novel compound, MMV1206386, has no close structural analogues among commercial herbicides. Physiological profiling suggested that MMV1206386 has a new mode of action and overall demonstrates how weighted rules can help during herbicide discovery programs.

## Abstract 2

Trawling hundreds of antimalarials for herbicides, we develop a weighted scoring system for the phys-chem ‘rules’ of herbicide-likeness. Using this, we discover the antimalarial MMV1206386 is herbicidal *via* a novel mode of action.

## Graphical abstract

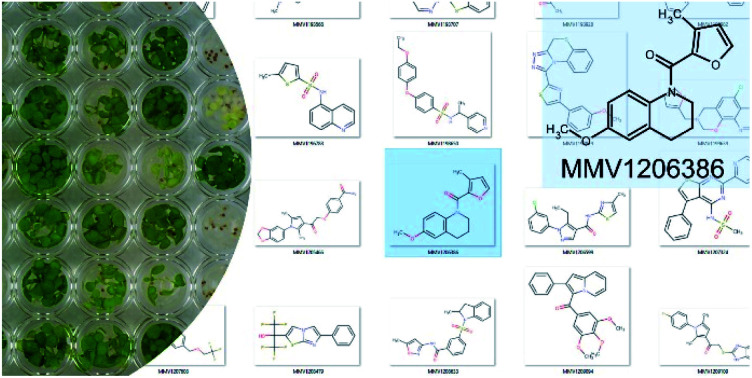

## Introduction

The implementation of herbicides in agriculture in the 1940s improved crop productivity, but the emergence of herbicide resistance in the last few decades threatens those gains in yield. The first case of herbicide resistance, triazine-resistant *Senecio vulgaris*, was documented in 1968 (ref. 1) and since then, the number of weed species resistant to one or more herbicides is now 262.^2^ Although practices such as herbicide rotation helps avoid the evolution of resistance and extends the life of herbicides, it relies on switching between modes of action.^3^ From the 1950s to the 1970s a new mode of action was introduced every two to three years, but this slowed in the 1980s. During the next 30 years no new herbicide mode of action was commercialised until tetflupyrolimet (inhibitor of plant dihydroorotate dehydrogenase) was announced by FMC Agricultural Solutions in 2019.^4,5^ Two old herbicides, cinmethylin and aclonifen, were rediscovered as having new modes of action,^6,7^ while new compounds with new modes of action have emerged, such as cyclopyrimorate (inhibits homogentisate solanesyltransferase, recently commercialised by Mitsui Chemical Agro Inc)^8^ and several novel cyclic methylphosphonates that target the pyruvate dehydrogenase complex.^5^ The traditional approach to discover herbicides is through mass chemical screening and only later determining the mode of action; as a result this process gives diminishing returns as highly active molecules increasingly match known herbicides or have a known mode of action.

The close relationship between the malaria parasite *Plasmodium falciparum* and plants like *Arabidopsis thaliana*^9,10^ means many antimalarial compounds are herbicidal and *vice versa e.g.* herbicides such as glyphosate, trifluralin, endothall and prodiamine are lethal to *Plasmodium* species^11–14^ as antimalarials cycloguanil, pyrimethamine, sulfadoxine, dihydroartemisinin and artesunate are herbicidal.^15^ The chemical tools for antimalarial drug discovery have burgeoned in the last decade with chemical libraries composed of chemically novel antimalarials made publicly available by large, international consortia.^16,17^ Exploiting this, two herbicidal compounds were discovered by screening a small subset of compounds from the Malaria Box chemical library against *A. thaliana*.^18,19^ Overall, the Malaria Box is a 400-compound set of structurally diverse and chemically simple drug-like molecules that are toxic to blood stage *P. falciparum* and have physico-chemical properties suitable for orally delivered drugs.^17^ The screening of this subset against *A. thaliana* grown on Murashige–Skoog agar medium revealed twenty highly herbicidal compounds.^18,19^ Ten of these were tested against soil-grown *A. thaliana*, and only two remained highly herbicidal, despite all ten molecules having physico-chemical properties within the broad range observed for commercial herbicides.^18,19^ There are lessons to be learned from looking at compounds that succeed as herbicides on plate-based assays, but fail in soil-based assays. Instead of a bias towards successes, considering the physico-chemical properties of compounds that fail to translate to soil-based assays might provide more sophisticated rules for predicting which herbicidal hits will continue to work when sprayed on the leaves of soil-grown plants.

Previous analyses,^20–22^ including a recent study of 334 commercial herbicides,^23^ demonstrated that although the physico-chemical properties of herbicides were similar to orally delivered drugs, herbicides possess fewer proton donors, a lower partition coefficient and molecular weight.^23,24^ To better understand the physico-chemical properties favoured by herbicides, we tested the entire Malaria Box for herbicidal activity of plate-based hits and determined which remained against soil-grown plants. There were correlations between some molecular properties and success in soil tests, which improved predictive ability. We used this newly refined set of rules to rank 631 liver-stage effective antimalarials *in silico*. Of the six highest-scoring compounds we found MMV1206386, a highly herbicidal tetrahydroquinoline derivative and chemically novel herbicidal compound, for which we obtained structure–activity information and determined that the compound potentially possesses a novel mode of action.

## Results

### Comparison of physico-chemical parameters of antimalarials and herbicides

**Fig. 1:**
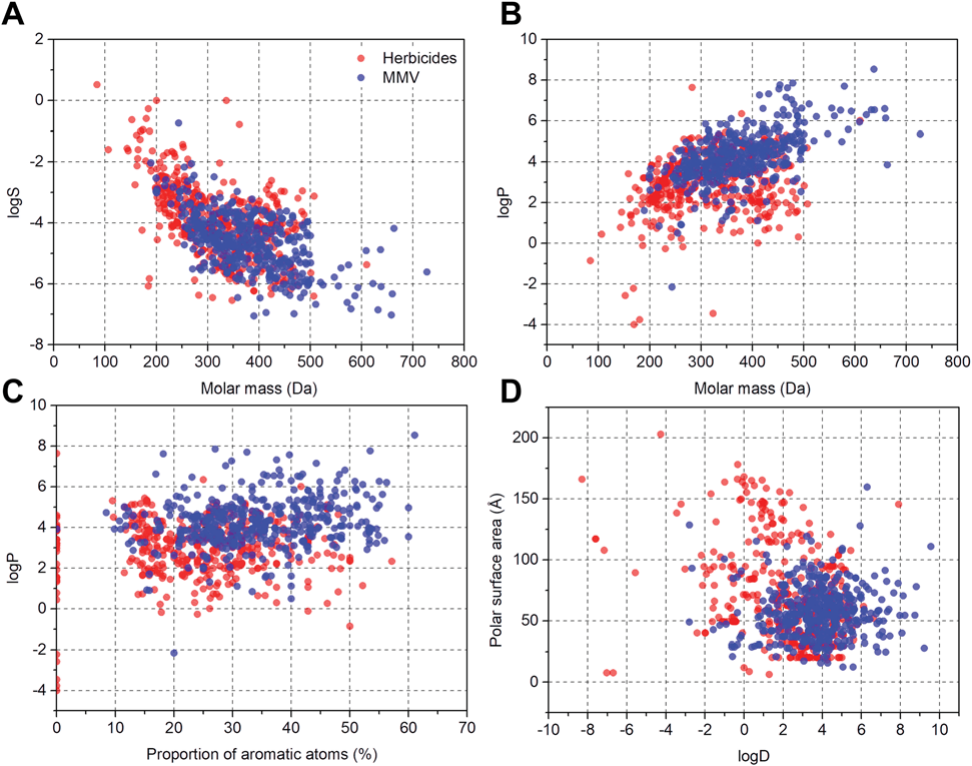
Cluster analysis of physico-chemical parameters of herbicides and the Malaria Box compounds. (A) molar mass – log *S*; (B) molar mass – log *P*; (C) proportion of aromatic atoms – log *P*; (D) log *D* – polar surface area; Red circles represent the 360 commercial herbicides and blue circles represent the MMV400 compounds. In general, the MMV400 have a narrower range for all physico-chemical properties assessed.

To evaluate the overall potential *in planta* bioavailability of compounds from the Malaria Box library (MMV400) we compared their solubility in water (log *S*), partition (log *P*) and distribution (log *D*) coefficients, molar mass, proportion of aromatic atoms and polar surface area with the corresponding parameters of 360 commercial herbicides (see ESI† Dataset 1). To visualise the distribution of values they were plotted as: molar mass – log *S* (Fig. 1A), molar mass – log *P* (Fig. 1B), proportion of aromatic atoms – log *P* (Fig. 1C) and log *D* – polar surface area (Fig. 1D). Overall, the physico-chemical properties of commercial herbicides and the MMV400 compounds were similar, however the range for each specific parameter was narrower for the MMV400 compounds. Compared to the herbicides, in general the MMV400 possessed lower water solubility and higher molar weight (Fig. 1A): 80% of the MMV400 had values of log *S* between −3.6 and −5.8, and molecular masses between 270 and 470 Da, whereas for 80% of the herbicides log *S* values varied between −2.2 and −5.5 and 430 and 200 Da respectively. The overall lower solubility of the MMV400 correlated with higher log *P* values: only 65% of MMV400 compounds had a log *P* below 4.6, whereas the log *P* of 90% of commercial herbicides did not exceed this value (Fig. 1B). Despite the higher log *P* of the MMV400, their proportion of aromatic atoms (20–50%) was below that of the herbicides (20–60%) (Fig. 1C). The MMV400 were also less polar: 90% of the MMV400 had a polar surface area less than 80 Å, and log *D* between 1.5 and 6, in contrast for herbicides 90th percentile of polar surface area was 130 Å, and log *D* fell within a range of −1.0 to 4.7 (Fig. 1D). Thus, in comparison with herbicides the MMV400 are in general more hydrophobic, less polar and possess lower water solubility, however their physico-chemical properties are still within the limits characteristic for commercial herbicides.

### Screening for herbicidal activity within the MMV400

MMV plates B and C (160 compounds) were screened previously^18,19^ so to create a complete dataset of antimalarials to help correlate physico-chemical properties with herbicidal activity, we screened and compiled data for the remaining 240 compounds (plates A, D and E) of the MMV400 against *Arabidopsis thaliana* grown aseptically on an agar medium. Seeds were sown on solid agar medium containing 80 μM of the MMV400 compound (see ESI† Dataset 2) or 80 μM control compound (typically a herbicide) left to germinate and grow for 16 days (see ESI† Fig. 1). All combined, 52 of the MMV400 demonstrated herbicidal activity with phenotypes from stunted growth to bleaching and inhibition of germination.

**Fig. 2:**
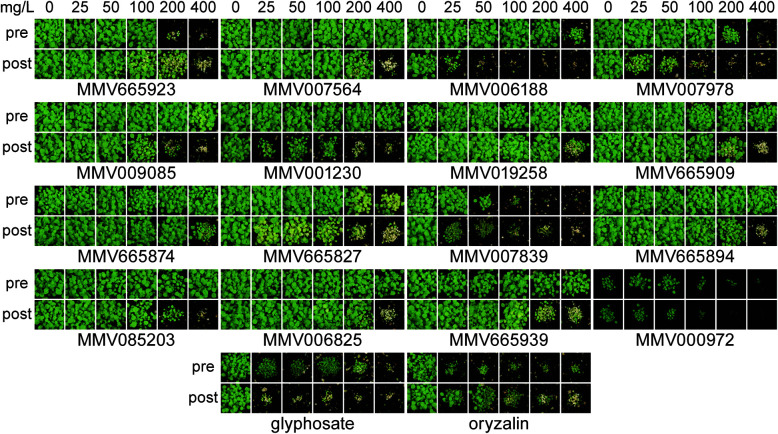
Herbicidal activity of selected MMV400 compounds against soil-grown *A. thaliana*. Each compound was applied on *A. thaliana* seeds on soil (pre) or on seedlings 3 and 6 days after germination (post). The antimalarials MMV007839 and MMV000972 were the most herbicidal. Commercial herbicides glyphosate and oryzalin were used as positive controls.

To evaluate the herbicidal activity of the active antimalarials under more relevant conditions, we chose 39 compounds based on commercial availability and structural diversity. These antimalarials were tested against *A. thaliana* pre- and post-emergence on soil with a concentration range from 0 to 400 mg L^−1^. Oryzalin and glyphosate were used as controls, being typical pre- and post-emergence herbicides respectively. Only 16 of the 39 compounds were active against plants grown on soil (Fig. 2), including those two previously known.^18,19^ The remaining 23 antimalarials did not inhibit the growth of the plants even at 400 mg L^−1^ (see ESI† Dataset 2).

Overall, the subset of 16 herbicidal MMV400 was mainly effective post-emergence. Only two compounds (MMV000972 and MMV007839) were effective both pre- and post-emergence, with strong growth inhibition at an application rate of 50 mg L^−1^ (Fig. 2). As shown previously,^18,19^ two compounds MMV007978 and MMV006188 were effective post-emergence and weakly active pre-emergence. MMV001230 was only active post-emergence (Fig. 2). These data show that, although the molecular properties of antimalarials are in general close to those of herbicides, just over half the antimalarials that were herbicidal on agar plate assays were inactive on soil. As a result, we thought it was beneficial to investigate how the physico-chemical properties of both the soil-active and inactive compounds correlated with activity.

### Correlating physico-chemical properties and herbicidal activity against soil-grown plants

To examine which properties might correlate with herbicidal activity for agar plates and soil, we examined the physico-chemical properties of the MMV400 compounds that were active on soil-grown plants *versus* compounds that were only herbicidal on agar. To better describe their molecular properties we used the set of descriptors that included log *P*, log *D*, log *S*, molecular mass, proportion of aromatic atoms, number of hydrogen bond donors and acceptors, number of rotatable bonds, polar surface area and formal charge. For each parameter we compared an average value for compounds active both on soil and agar plates (*n* = 16) and compounds which were not active on soil, but were active on agar plates (*n* = 23) and we used a two-sample *t*-test to determine statistical significance of any differences (Table 1).

**Table 1:** Average values of physico-chemical parameters for soil and plate-active antimalarials (±standard deviation) *vs.* plant-only active antimalarials (±standard deviation)

Parameter	Active on soil and plates	Plate-active only	*p*-Value^a^
Molar mass (g per mole^−1^)	351 ± 46	360 ± 52	0.60
Aromatic atoms (%)	32 ± 12	32 ± 11	0.90
Rotatable bond count	4.6 ± 2	5.2 ± 2.5	0.42
Hydrogen bond acceptor^b^	3.6 ± 1	3.0 ± 1.5	0.12
Hydrogen bond donor^b^	1.2 ± 1	1.6 ± 1	0.09
Polar surface area (Å^2^)	61.5 ± 17	54.6 ± 19	0.25
log *D*	3.6 ± 1	3.7 ± 1	0.93
log *S*	−4.3 ± 1	−4.6 ± 1	0.08
**log** ***P***	**3.5 ± 1**	**4.2 ± 1**	**0.03**

a *p*-Values are given for a two-sample *t*-test.

b The hydrogen bond donor and acceptor count are given for pH 7.4. Significant differences at significance level 0.05 is bolded.

The only significant (*p* < 0.05) difference in the average values was for log *P*. The mean log *P* for soil-active compounds (3.5 ± 1) was lower than for compounds that were inactive on soil (4.2 ± 1) and closer to the mean log *P* for commercial herbicides (2.9 ± 1.5). This indicated that high log *P* reduced activity against soil-grown plants for antimalarials that were herbicidal on agar. Herbicide-like values for log *S* (*p* = 0.08) and number of hydrogen bond donors (*p* = 0.09) were important, but just showed a trend (*p* < 0.1).

**Fig. 3:**
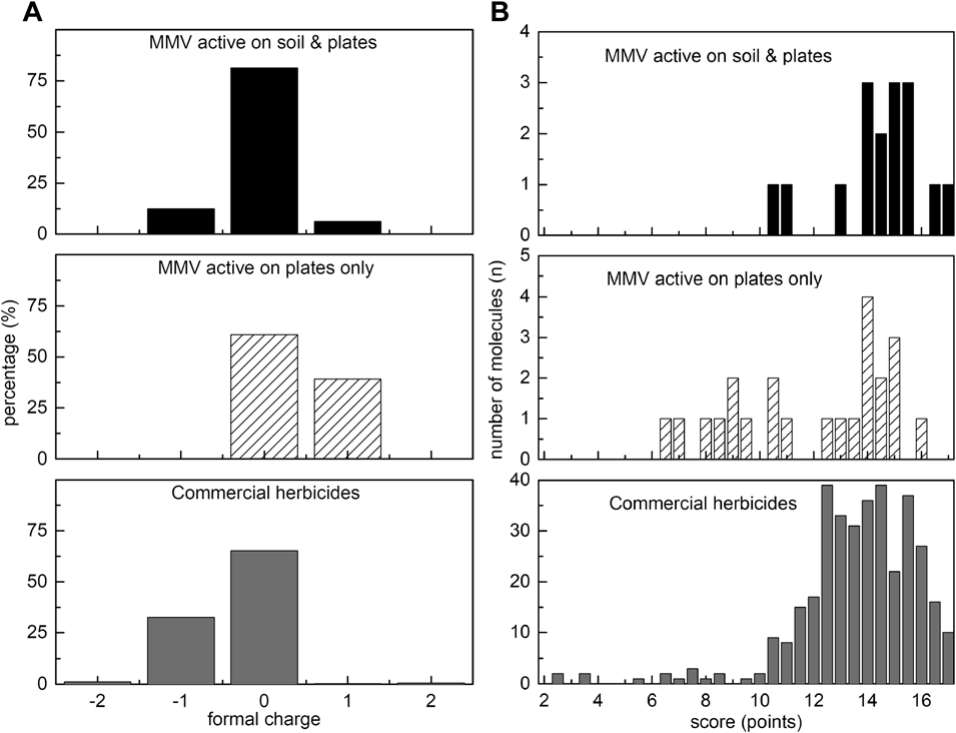
Charge and scores for herbicidal MMV400 compounds *vs.* commercial herbicides. (A) Herbicidal MMV400 and commercial herbicides tend to be neutral or negatively charged. The formal charge was calculated at pH 7.4: positive formal charge means that a molecule exists in solution in cationic form, negative charge – anionic form, and zero charge corresponds to neutral form; (B) distributions of scores for ‘herbicide-likeness’ for herbicidal MMV400 compounds and commercial herbicides. The MMV400 compounds that were only herbicidal on plates demonstrate a much wider distribution of scores, whereas the soil-active MMV400 closely matched the pattern of commercial herbicides.

To examine whether formal charge might correlate with activity against soil-grown plants, we calculated cumulative distribution functions of charge for soil-active antimalarials, commercial herbicides and antimalarials inactive on soil (Fig. 3A). It is worth noting that only three of the 360 commercial herbicides (Fig. 3A, dark grey bars) had a positive charge, with all having either neutral or slightly negative charge. By contrast, the soil inactive antimalarials had significantly higher proportion of positively charged compounds (Fisher's exact test *p* < 0.05) (Fig. 3A, dark grey bars). The soil-active antimalarials (Fig. 3A, black bars) more closely resembled commercial herbicides with most being neutral or having a single negative charge, however one of the 16 compounds had a single positive charge.

To quantify how similar a particular compound was to the herbicides, a weighted scoring system was developed based on the closeness to the average of each herbicide physico-chemical parameter. Scoring rules were split into continuous and discrete physicochemical parameters. Physicochemical properties that are continuous variables include molar mass, proportion of aromatic atoms, polar surface area, log *D*, log *S* and log *P*. For these continuous parameters, we developed a scoring system based on the number of standard deviations away from the herbicidal mean that a particular parameter for an MMV compound fell (Table 2). The standard score for a parameter within one standard deviation was 1.5, two standard deviations was 1.0, three standard deviations was 0.5 and more than three standard deviations the score given was −1.0. Discrete physicochemical parameters (that is parameters possessing whole number values only) included rotatable bond count, number of H-bond acceptors/donors and formal charge. For these discrete parameters, we used scoring rules based on how far a parameter value is from the corresponding mode value for the 360 commercial herbicide set (Table 3).

**Table 2:** Weighted scoring for continuous parameters

Parameter	Average value ± SD^a^	1 SD	2 SD	3 SD	>3 SD
Molar mass (g per mole)	317 ± 88	1.5	1.0	0.5	−1.0
Aromatic atoms (%)	25 ± 12	1.5	1.0	0.5	−1.0
PSA (Å^2^)	72 ± 39	1.5	1.0	0.5	−1.0
log *D*	2.2 ± 2.4	1.5	1.0	0.5	−1.0
log *S*	−3.5 ± 1	1.5	1.0	0.5	−1.0
log *P*	2.9 ± 1.5	3.0	2.0	1.0	−1.0

a The score given is based on being within 1, 2, 3 or >3 standard deviations (SD) from the average value (for 360 commercial herbicides). Note that the log *P* score is weighted differently.

**Table 3:** Weighted scoring for discrete parameters

Parameter	Mode value^a^	Value + 2	Value + 1	Value	Value − 1	Value − 2	<−2 or >+2
Rotatable bond count	5.0	0.5	1.0	1.5	1.0	0.5	−1.0
H-bond acceptor^b^	2.0	0.5	1.0	1.5	NA	NA	−1.0
H-bond donor^b^	0.0	0.5	1.0	1.5	1.0	0.5	−1.0
Formal charge^b^	0.0	−3.0	2.0	3.0	2.0	1.0	−1.0

a The mode value for the 360 commercial herbicides is shown and scores are givens for values above and below this. NA – not applicable.

b H-bond acceptor, H-bond donor and formal charge were counted at pH 7.4. Note that the score for formal charge is weighted heavily.

To weight the scoring system to account for the aforementioned importance of log *P* and formal charge (Table 1 and Fig. 3A) we doubled the standard score for log *P* values falling within one to three standard deviations, but no additional penalty if log *P* fell outside three standard deviations (Table 2). For the discrete parameter of formal charge, positive values were heavily penalised and neutral or negative values had a higher weighting (Table 3). The total score for herbicide-likeness was the sum of scores for all parameters with the maximum possible score being 18 points (Tables 2 and 3).

Using this scoring system, we plotted distribution curves for antimalarials active only on plates (Fig. 3B, white, diagonally hashed), the antimalarials active on plates and soil-grown plants (Fig. 3B, black) and commercial herbicides (Fig. 3B, dark grey). For the 39 MMV-antimalarials that were tested against soil-grown *A. thaliana* (see ESI† Dataset 2) the majority of soil-active antimalarials had scores of 14 or above, with the average score of 14.4 points (*n* = 16), that was close to 13.6 points for commercial herbicides (*n* = 360). Scores for plate active-only antimalarials for herbicide likeness varied from 6.5 to 17, with the average value 11.9 points (*n* = 23) which is significantly different (*p* < 0.05) from the average value for herbicides and soil-active antimalarials. This scoring system appears to give values consistent for compounds showing herbicidal activity in more natural, soil-grown conditions.

To test this weighted scoring system on a different dataset, we performed an *in silico* pre-screening of 631 molecules that were active against liver-stage malaria parasites, which possess low hepatotoxicity and potentially good oral bioavailability.^25^ Consisting of antimalarial compounds a high proportion should be herbicidal, but this liver-active set were structurally different to the blood stage MMV400 antimalarials. We hoped to demonstrate whether the aforementioned rules could focus attention only on compounds with appropriate physico-chemical properties of soil-active herbicidal compounds. We scored all 631 (whose names also have an MMV prefix) for herbicide-likeness and tested the top six scoring compounds that were commercially available.

**Fig. 4:**
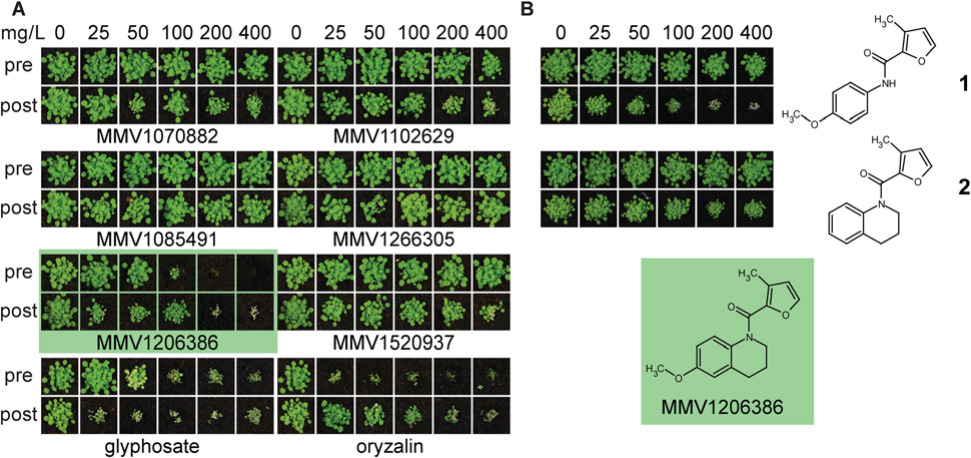
Herbicidal activity of liver-stage active MMV compounds on soil. (A) Herbicidal activity of liver-stage active MMV compounds against soil-grown *A. thaliana*. Comparison of herbicidal activity of selected antimalarials against *A. thaliana* treated pre- and post-emergently show that MMV1260386 (highlighted in green) was the most herbicidal and comparable with commercial herbicides; (B) analogues of MMV1260386 showed herbicidal activity did not depend on quinolone motif. Compound 1 which lacks the second ring was decreased in pre-, but nor post-emergence activity. Elimination of *p*-methoxy group (2), virtually eliminated herbicidal activity.

Four of these six top-scoring liver-stage active antimalarial compounds were herbicidal, but only MMV1206386 had activity commensurate with commercial herbicides. MMV1206386 completely inhibited the growth of *A. thaliana* at 200 mg L^−1^ application rate in both pre- and post-emergence application (Fig. 4A). Two compounds: MMV1085491 and MMV1266305 were completely inactive, despite a high score for herbicide-likeness (Fig. 4A), probably due to the absence of a plant target. Notably, both compounds share a similar structural core (see ESI† Dataset 3).

**Fig. 5:**
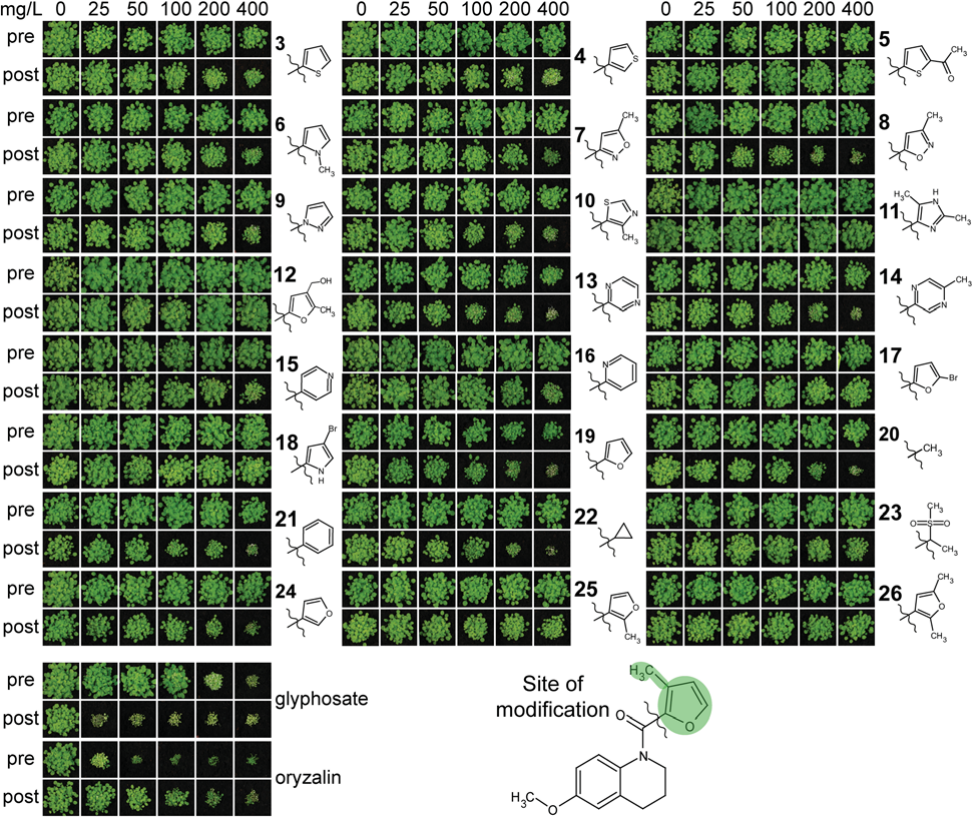
Effect of the 2-methyl furanyl group in MMV1260386 on herbicidal activity. Pre-emergence activity was eliminated in all molecules except 19. Compounds 5, 11, 12, 17, 18, 23, 25, and 26 completely lost herbicidal activity.

To assess the opportunities for improving herbicidal activity of MMV1206386, 25 structural analogues were screened. While none of structural changes increased herbicidal activity, some had no effect and many others decreased or abolished herbicidal activity (Fig. 4B and 5).

First of all, we checked if the tetrahydroquinoline motif was essential for herbicidal activity. Replacement of the motif with the appropriate methoxyphenyl group as in 1 did not change post-emergence herbicidal activity, but eliminated pre-emergence. In contrast, the methoxy group in the position (6) of quinolone ring was crucial for herbicidal activity: removal of this group in 2 completely eliminated pre-emergence activity and significantly reduced post-emergence activity.

To assess the importance of the furanyl motif (marked, Fig. 5) we replaced it with other heterocycles as in 3–19, 24–26, aromatic 21 and aliphatic groups 20, 22 and 23. In most cases the replacement of this group greatly reduced the activity both pre- and post-emergence. Replacing the furan motif with other five-membered heterocycles such as those in 3–10 eliminated pre-emergence activity and drastically decreased post-emergence activity. Replacement of the furanyl motif with a pyrimidinyl motif as in 13 and 14 also decreased herbicidal activity, but to a smaller extent than for replacement with a pyridinyl moiety, as in 15 and 16.

Increasing the heterocyclic nature of the five-membered ring as in 11 and 12 or increasing the functionality of the five membered ring as in 5, 17, 18, 26 also lost activity. Removal of the 3-methyl group as in 19 clearly reduced pre- and post-emergence herbicidal activity. Replacement of furan motif with groups which did not contain heterocyclic core 20–22 similarly decreased herbicidal activity or virtually eliminated as in 23. Changing the position of the link between the furan motif and the carbonyl group greatly reduced herbicidal activity in 24, and completely eliminated it in 25. With these results available, we examined the potential of MMV1206386 to act as a herbicide against more relevant weed species. Eight crop specific weed species in pre- and post-emergence application were assayed (Table 4).

**Table 4:** | MMV1206386 is strongly herbicidal against *Abutilon theophrasti* and *Setaria viridis*

Weed species	pre-1 kg ha^−1^	pre-2 kg ha^−1^	post-1 kg ha^−1^	post-2 kg ha^−1^
*Abutilon theophrasti*	0^a^	25	80	70
*Amaranthus retroflexus*	35	65	30	65
*Alopecurus myosuroides*	ND^b^	ND	0	0
*Avena fatua*	ND	ND	0	25
*Echinochloa crus-galli*	30	45	0	0
*Setaria viridis*	ND	ND	60	90
*Apera spica-venti*	0	0	ND	ND
*Setaria faberi*	0	0	ND	ND

a Herbicidal effect of MMV1206386 on different weed species was evaluated 21 days after treatment. The values obtained are herbicidal ratings of MMV1206386 when applied pre-emergence (pre-) or post-emergence (post-) against eight different crop weeds (values given as % of growth against the weed control).

b ND – not determined.

**Fig. 6:**
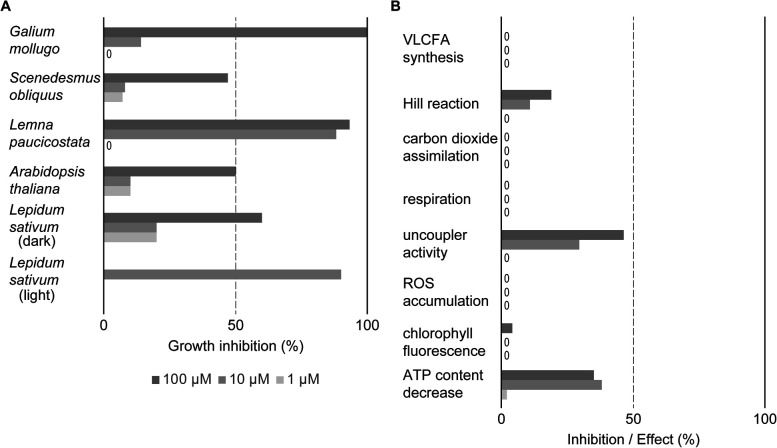
Evaluation of MMV1260386 by a BASF physiological profile.^19^ (A) MMV1260386 strongly inhibited growth of both whole plants and cell suspensions. The herbicidal effect of MMV1206386 was noticeably high under light growth conditions. “0” – no inhibitory effect; (B) MMV1260386 had moderate uncoupler and Hill reaction inhibitor activities. VLCFA – very long chain fatty acid; ROS – reactive oxygen species.

MMV1206386 was strongly active as a post-emergence herbicide against *Abutilon theophrasti* (velvetleaf) and *Setaria viridis* (green bristlegrass). Moderate susceptibility was observed in the case of *A. retroflexus* whereas *E. crus-galli* was moderately sensitive to pre-emergence treatment (45% of control at 2 kg ha^−1^), but tolerant to post-emergence treatment. Three species, *A. fatua, A. spica-venti* and *S. faberi* were resistant to MMV1206386. Based on these data we can assume that MMV1206386 has the potential to control a wide range of species, and should be more successful if used as a post-emergence herbicide. Since a search of MMV1206386 structural analogues among known commercial herbicides did not find any structurally similar molecules, we thought MMV1206386 might have a novel mode of action. To determine this we obtained an industry-standard physiological profile, which includes 13 different assays and generates a profile that can be compared to commercial herbicides, for many their modes of action being known.^19,26,27^ Firstly, we tested MMV1206386 against small plants and cell suspension to see which symptoms are induced by treatment with the compound (Fig. 6A).

MMV1206386 strongly inhibited cell division in heterotrophic *Galium mollugo* (cleaver) cell suspension culture at 100 μM. At lower concentrations cell division was only mildly affected. Growth of *Lemna paucicostata* (duckweed) was strongly inhibited when the compound was applied at 10 μM and 100 μM accompanied by rapid necrosis, reduced leaf growth and root growth inhibition. Treatment of plate-grown *A. thaliana* by MMV1206386 caused chlorosis, reduced leaf growth, root growth inhibition and hypocotyl swelling in seedlings, and these symptoms were similar to what was observed for soil-grown plants treated with MMV1206386. The germination inhibition assay with dark-grown *Lepidum sativum* (cress) showed that MMV1206386 had a moderate effect on germination rate. In contrast, when *L. sativum* plants were grown under light the inhibition of germination was more than 70% at all tested concentrations.

Compared to industry-standard physiological profiles of inhibitors of photosystem II, MMV1206386 mildly inhibited photosynthetic electron transport in photosystem II in isolated wheat thylakoids at 100 μM (Fig. 6B). In the same linecontrast, chlorophyll fluorescence measured in *Lemna paucicostata* was only slightly affected at 100 μM. MMV1206386 demonstrated robust or medium uncoupler activity: the electron transport activity dropped by 46% and 30% at 100 μM and 10 μM respectively upon treatment, as well as ATP levels decreased robustly. Interestingly, there was no effect on the cress very long chain fatty acid synthesis (at 1 μM), carbon dioxide assimilation (at 1000 μM), respiration (at 100 μM) or reactive oxygen species accumulation (at 10 μM and 100 μM). To find out what mode of action MMV1206386 had, this physiological profile was compared with the BASF database of physiological profiles representing all known modes of action for herbicides and reference compounds with standard modes of action. This comparison did not reveal any matches among fingerprint profiles for commercial herbicides, therefore suggested that MMV1206386 represents a novel mode of action.

## Discussion

Since the advent of Lipinski's rules for oral drugs in 1997 several studies have analysed the physico-chemical parameters of herbicides.^20–23,28^ Where the underpinning datasets were made known, these consisted of commercial herbicides, so none of these studies considered initial herbicidal hits that then failed in foliar applications.^20,22,23^ Only the study by Clarke considered data from both successful and unsuccessful herbicidal leads.^21^ Unfortunately, as the identities of their training set was suppressed, no subsequent analysis could use these data.

To create a dataset containing positive and negative data we completed screening of the 400-compound Malaria Box against *A. thaliana* on agar plates and then tested a sub-set against soil-grown plants. The reason to choose the Malaria Box was the fact that it was partially screened in previous studies and yielded many herbicidal hits, so screening the rest of the library would expand the training set.^18,19^ Screening the entire MMV400 increased the number of actives tested on soil and agar from 9 to 39 compounds plants, enabling statistical analyses like Fisher's exact test and two sample *t*-test.

To describe molecular properties of herbicides, previous studies used Lipinski's ‘rule of five’ itself or with minor corrections.^20–22^ The original ‘rule of five’ employed a few simple descriptors such as partition coefficient (log *P*), molecular weight, number of hydrogen bond donors and acceptors and rotatable bond count to predict oral bioavailability of a drug candidate.^24^ These parameters do not consider ionisation properties, polarity or solubility, so to better characterise the physico-chemical parameters of herbicidal and non-herbicidal molecules we used an extended set of descriptors.^23^ The set included all Lipinski's descriptors as well as: distribution coefficient (log *D*); characterises distribution of the dominant ionisation form at given pH, solubility coefficient (log *S*); characterises solubility of molecules, polar surface area, aromatic atom proportion and formal charge. Not surprisingly, compounds active against soil-grown plants as well as compounds active only against plate-grown plants fit within characteristic limits of physico-chemical properties for commercial herbicides. The log *P* of antimalarials active against soil-grown plants had a low average value of log *P* that did not differ significantly from commercial herbicides, whereas the antimalarials active only against agar-grown plants had significantly higher log *P* value.

Another parameter that differed was formal charge. Soil-active antimalarials were mostly neutral or negatively charged. Although positive charge facilitates absorption by roots or leaves,^29,30^ it might impede translocation of herbicide molecules. This is similar to what has been observed for nanoparticles^31^ and fertilizers,^32^ where in contrast negatively charged and neutral particles have increased translocation.^33^ In addition, organic soils are in general possess negatively charged materials and so positively charged herbicides are readily bound, limiting pre-emergent efficacy.^34^

Our hypothesis was a weighted scoring system for herbicide-likeness could allow *in silico* screening of libraries and find new herbicides. The only published scoring system for herbicide-likeness was one proposed by Avram *et al.* in 2014 (ref. 35) where quantitative estimates of drug-likeness based on the use of distribution functions were used to create a continuous ranking model. Although it worked well describing herbicide-likeness of molecules deposited in the AgroSAR patent database, it did not consider formal charge and did not discriminate between the different descriptors, being used, according to their importance. Moreover, using a continuous distribution fraction for describing discrete parameters such as hydrogen donor and acceptor count, although convenient, is not appropriate for discrete parameters.

The scoring system described here takes into account the importance of log *P* and formal charge for herbicide-likeness and scores discrete and continuous parameters differently. After the scoring system for herbicide-likeness was validated using the subset of Malaria Box compounds that were tested against soil-grown plants we applied it to a compound library of 631 antimalarials active against liver-stage *P. falciparum*.^25^ We chose this library as it consists of compounds with high antiprotozoal activity, so we expected a high proportion to be herbicidal, but the set would be structurally different from the blood stage antimalarials in the Malaria Box.

For the library targeted against liver-stage *P. falciparum*, eleven molecules scored 17 or more of a maximal possible 18 points, however only six were readily available. The only molecule to exhibit high herbicidal activity was MMV1206386, and a search did not reveal any herbicides with similar chemical structure. Structure–activity studies revealed critical aspects of its structure, and physiological profiling suggested that MMV1206386 has a new mode of action with an unusual combination of affected processes. While MMV1206386 demonstrated uncoupler activity, the respiration and carbon dioxide assimilation processes were not affected, although uncoupling is normally reflected in these processes. The combination of symptoms induced by MMV1206386 treatment in *A. thaliana* did not match with any of more than 2700 tested by BASF before. There were three matches (of more than 10 000 compounds) for the symptoms observed in *Lemna paucicostata* treated by MMV1206386, however the physiological profiles did not match those obtained for MMV1206386. The combination of structural uniqueness, distinct physiological profiles and unique combination of symptoms support the hypothesis that MMV1206386 has a new mode of action.

## Experimental

### Updating the Gandy *et al.* (2015) herbicide database

A database of commercial herbicides compiled in 2015 contained 334 compounds^23^ and as a prelude to this study, we updated this database. Dimethylarsinic acid, DSMA (disodium methyl arsenate) and MSMA (monosodium methyl arsenate) were removed as they are rarely or no longer used due to toxicity.^36^ Acrolein, which is used as a commercial algicide, was removed because the database is focused on herbicides applied against soil-grown weeds. Metolachlor, a racemic mixture of S-metolachlor and R-metolachlor, was excluded because it duplicated physico-chemical properties of S-metolachlor that are already present in the database. To check if we missed any herbicides in the first iteration we screened through “Alan Wood's Pesticides Common Names Compendium” and “Pesticide Properties Data Base by the University of Hertfordshire” and found 27 commercial herbicides not in the original database.^36,37^ These include four 4-hydroxyphenylpyruvate dioxygenase inhibitors (fenquinotrione, tioclorim, tolpyralate, tefuryltrione); five that affect protoporphyrinogen oxidase (flumipropyn, fluorodifen, tiafenacil, bencarbazone, trifludimoxazin); three photosystem II inhibitors (ametridione, dipropetryn, eglinazine); two synthetic auxins (4-chlorophenoxyacetic acid, halauxifen); and six herbicides from different groups, including the acetyl-CoA carboxylase inhibitor haloxyfop-P, the acetolactate synthase inhibitor monosulfuron, 1-deoxy-d-xylulose 5-phosphate synthase inhibitor bixlozone, a phytoene desaturase inhibitor metflurazon, an auxin transport inhibitor thidiazuron, and an inhibitor of long chain fatty acid synthesis terbuchlor. Seven of the newly added herbicides have unknown modes of action (benzipram, florpyrauxifen, haloxydine, isopolinate, pyribambenz-isopropyl, pyribambenz-propyl, trifopsime). In addition, a few herbicides have been introduced to market since 2015 and so were also added. These three herbicides each possess a novel mode of action: an inhibitor of pyruvate dehydrogenase (clacyfos), an inhibitor of homogentisate solanesyltransferase (cyclopyrimorate) and an inhibitor of dihydroorotate dehydrogenase (tetflupyrolimet). The SMILES codes of the newly added compounds were used to propagate their details and the new database (see ESI† Dataset 1).

### Calculation of physico-chemical properties of the Malaria Box compounds

To describe physico-chemical properties of the Malaria Box compounds a set of physico-chemical descriptors was generated. These included solubility coefficient (log *S*), partition coefficient (log *P*), distribution (log *D*) coefficient, molar mass, proportion of aromatic atoms, polar surface area, rotatable bond count, hydrogen bond donor count, hydrogen bond acceptor count and formal charge. The parameter values were calculated using calculator plugins included in Marvin Suite program package v. 20.19 (ChemAxon). Formal charge, hydrogen bond donor count, hydrogen bond acceptor count, were calculated for the major tautomeric form at pH 7.4.

### Herbicidal activity assay on plates

To assess herbicidal activity of the Malaria Box compounds they were tested against agar-grown *Arabidopsis thaliana*. A 2.5 μL aliquot of either an 8 mM solution of antimalarial in DMSO or pure DMSO (negative control) was placed into a well of a sterile transparent 96-well plate prior to pouring of 250 μL of molten MS-agar medium that contained 1% of agar, 4 g L^−1^ of Murashige–Skoog, 10 g L^−1^ of glucose and 0.3% 2-(*N*-morpholino) ethanesulfonic acid (v/v), pH 5.7. After the agar solidified roughly 30–40 ethanol-sterilised *A. thaliana* (Col-0) seeds were sown onto the surface as 25 μL of seed suspension in 0.1% agar. Prior to the experiment the seeds were stratified for three days to synchronise germination. After the surface of agar dried the plates were covered with lids and sealed with porous tape, transferred to a growth chamber and left to grow under long-day illumination (16 h light/8 h dark, 136 μmol m^−2^ per s^−1^) at 26 °C and 60% relative humidity. After sixteen days, lids were removed and images taken.

### Herbicidal activity assay on soil

To assess herbicidal activity ∼30 *A. thaliana* Col-0 seeds were sown in 63 × 63 × 59 mm pots consisting of Irish peat pre-wet before sowing. Seeds were treated for 3 days in the dark at 4 °C to synchronise germination and then grown in a chamber at 22 °C, with 60% relative humidity and in a 16 h light/8 h dark photoperiod. Antimalarial compounds and control herbicide oryzalin were initially dissolved in DMSO at 20 mg mL^−1^ and further diluted in water containing 0.02% surfactant (Brushwet, SST Australia, v/v) prior to treatments. Another herbicide control, glyphosate was diluted in the same manner, but the original 20 mg mL^−1^ stock was prepared in water. DMSO at a concentration of 2% (v/v) was used as a negative control. Seeds or seedlings were treated as previously detailed^19^ with 500 μL of 0, 25, 50, 100, 200 or 400 mg L^−1^ solutions of each compound. Pre-emergence treatments were done as trays were moved into their first long day, whereas post-emergence treatments were conducted three and six days after germination. Seedlings were grown for 16 days after treatment before photos were taken.

### Physiological profiling of MMV1206386

To determine the mode of action of MMV1206386 the effect of the compound on different physiological processes was studied according to protocols previously described.^26,27^ The influence of MMV1206386 on cell growth was studied in cell suspensions of phototrophic green algae *Scenedesmus obliquus*. The effect of MMV1206386 on Hill reaction rate was assessed in isolated wheat (*Triticum aestivum*) chloroplasts. Carbon assimilation and oxygen consumption was studied in heterotrophic *Galium mollugo* cell suspensions. Oxidative phosphorylation uncoupler activity, ATP content, chlorophyll fluorescence and accumulation of reactive oxygen species were measured in *Lemna paucicostata*. Toluidine-blue staining of *Lepidium sativum* hypocotyls was used for the detection of inhibition of very long chain fatty acid synthesis. Additionally the effect of MMV1206386 on *A. thaliana* seedling morphology was evaluated.

## Conclusions

By comparing failed and successful hits from the Malaria Box it was revealed how different molecular properties had different impacts on the chance of herbicidal activity. Having a suitable partition coefficient and formal charge appeared essential, whereas other parameters could range more widely. Based on these findings we developed a weighted scoring system for herbicide-likeness and used it to select top-scoring compounds from a large compound library of liver-stage effective antimalarial leads. One molecule (MMV1206386) had efficiency against soil-grown plants comparable to commercial herbicides and no close structural analogues in use and its physiological profile indicate a new mode of action. Thus, the weighted rules is a useful tool when using high throughput screening approaches in the discovery of new herbicides.

## Conflicts of interest

There are no conflicts to declare.

## Supplementary Material

RA-011-D1RA00914A-s001

RA-011-D1RA00914A-s002

RA-011-D1RA00914A-s003

RA-011-D1RA00914A-s004

RA-011-D1RA00914A-s005

RA-011-D1RA00914A-s006

RA-011-D1RA00914A-s007

RA-011-D1RA00914A-s008

RA-011-D1RA00914A-s009

RA-011-D1RA00914A-s010

RA-011-D1RA00914A-s011

RA-011-D1RA00914A-s012

RA-011-D1RA00914A-s013

RA-011-D1RA00914A-s014

RA-011-D1RA00914A-s015

RA-011-D1RA00914A-s016

RA-011-D1RA00914A-s017

RA-011-D1RA00914A-s018

RA-011-D1RA00914A-s019

RA-011-D1RA00914A-s020

RA-011-D1RA00914A-s021

RA-011-D1RA00914A-s022

RA-011-D1RA00914A-s023

RA-011-D1RA00914A-s024

RA-011-D1RA00914A-s025

RA-011-D1RA00914A-s026

RA-011-D1RA00914A-s027

RA-011-D1RA00914A-s028

RA-011-D1RA00914A-s029

RA-011-D1RA00914A-s030

RA-011-D1RA00914A-s031

RA-011-D1RA00914A-s032

RA-011-D1RA00914A-s033

RA-011-D1RA00914A-s034

RA-011-D1RA00914A-s035

RA-011-D1RA00914A-s036

RA-011-D1RA00914A-s037

RA-011-D1RA00914A-s038

RA-011-D1RA00914A-s039

RA-011-D1RA00914A-s040

RA-011-D1RA00914A-s041

RA-011-D1RA00914A-s042

RA-011-D1RA00914A-s043

RA-011-D1RA00914A-s044

RA-011-D1RA00914A-s045

RA-011-D1RA00914A-s046

RA-011-D1RA00914A-s047

RA-011-D1RA00914A-s048

RA-011-D1RA00914A-s049

RA-011-D1RA00914A-s050

RA-011-D1RA00914A-s051

RA-011-D1RA00914A-s052

RA-011-D1RA00914A-s053

RA-011-D1RA00914A-s054

RA-011-D1RA00914A-s055

RA-011-D1RA00914A-s056

RA-011-D1RA00914A-s057

RA-011-D1RA00914A-s058

RA-011-D1RA00914A-s059

RA-011-D1RA00914A-s060

RA-011-D1RA00914A-s061

RA-011-D1RA00914A-s062

RA-011-D1RA00914A-s063

RA-011-D1RA00914A-s064

RA-011-D1RA00914A-s065

RA-011-D1RA00914A-s066

RA-011-D1RA00914A-s067

RA-011-D1RA00914A-s068

RA-011-D1RA00914A-s069

RA-011-D1RA00914A-s070

RA-011-D1RA00914A-s071

RA-011-D1RA00914A-s072

RA-011-D1RA00914A-s073

RA-011-D1RA00914A-s074

RA-011-D1RA00914A-s075

RA-011-D1RA00914A-s076

RA-011-D1RA00914A-s077

RA-011-D1RA00914A-s078

RA-011-D1RA00914A-s079

RA-011-D1RA00914A-s080

RA-011-D1RA00914A-s081

RA-011-D1RA00914A-s082

RA-011-D1RA00914A-s083

RA-011-D1RA00914A-s084

RA-011-D1RA00914A-s085

RA-011-D1RA00914A-s086

RA-011-D1RA00914A-s087

RA-011-D1RA00914A-s088

RA-011-D1RA00914A-s089

RA-011-D1RA00914A-s090

RA-011-D1RA00914A-s091

RA-011-D1RA00914A-s092

RA-011-D1RA00914A-s093

RA-011-D1RA00914A-s094

RA-011-D1RA00914A-s095

RA-011-D1RA00914A-s096

RA-011-D1RA00914A-s097

RA-011-D1RA00914A-s098

RA-011-D1RA00914A-s099

RA-011-D1RA00914A-s100

RA-011-D1RA00914A-s101

RA-011-D1RA00914A-s102

RA-011-D1RA00914A-s103

RA-011-D1RA00914A-s104

RA-011-D1RA00914A-s105

RA-011-D1RA00914A-s106

RA-011-D1RA00914A-s107

RA-011-D1RA00914A-s108

RA-011-D1RA00914A-s109

RA-011-D1RA00914A-s110

RA-011-D1RA00914A-s111

RA-011-D1RA00914A-s112

RA-011-D1RA00914A-s113

RA-011-D1RA00914A-s114

RA-011-D1RA00914A-s115

RA-011-D1RA00914A-s116

RA-011-D1RA00914A-s117

RA-011-D1RA00914A-s118

RA-011-D1RA00914A-s119

RA-011-D1RA00914A-s120

RA-011-D1RA00914A-s121

RA-011-D1RA00914A-s122

RA-011-D1RA00914A-s123

RA-011-D1RA00914A-s124

RA-011-D1RA00914A-s125

RA-011-D1RA00914A-s126

RA-011-D1RA00914A-s127

RA-011-D1RA00914A-s128

RA-011-D1RA00914A-s129

RA-011-D1RA00914A-s130

RA-011-D1RA00914A-s131

RA-011-D1RA00914A-s132

RA-011-D1RA00914A-s133

RA-011-D1RA00914A-s134

RA-011-D1RA00914A-s135

RA-011-D1RA00914A-s136

RA-011-D1RA00914A-s137

RA-011-D1RA00914A-s138

RA-011-D1RA00914A-s139

RA-011-D1RA00914A-s140

RA-011-D1RA00914A-s141

RA-011-D1RA00914A-s142

RA-011-D1RA00914A-s143

RA-011-D1RA00914A-s144

RA-011-D1RA00914A-s145

RA-011-D1RA00914A-s146

RA-011-D1RA00914A-s147

RA-011-D1RA00914A-s148

RA-011-D1RA00914A-s149

RA-011-D1RA00914A-s150

RA-011-D1RA00914A-s151

RA-011-D1RA00914A-s152

RA-011-D1RA00914A-s153

RA-011-D1RA00914A-s154

RA-011-D1RA00914A-s155

RA-011-D1RA00914A-s156

RA-011-D1RA00914A-s157

RA-011-D1RA00914A-s158

RA-011-D1RA00914A-s159

RA-011-D1RA00914A-s160

RA-011-D1RA00914A-s161

RA-011-D1RA00914A-s162

RA-011-D1RA00914A-s163

RA-011-D1RA00914A-s164

RA-011-D1RA00914A-s165

RA-011-D1RA00914A-s166

RA-011-D1RA00914A-s167

RA-011-D1RA00914A-s168

RA-011-D1RA00914A-s169

RA-011-D1RA00914A-s170

RA-011-D1RA00914A-s171

RA-011-D1RA00914A-s172

RA-011-D1RA00914A-s173

RA-011-D1RA00914A-s174

RA-011-D1RA00914A-s175

RA-011-D1RA00914A-s176

RA-011-D1RA00914A-s177

RA-011-D1RA00914A-s178

RA-011-D1RA00914A-s179

RA-011-D1RA00914A-s180

RA-011-D1RA00914A-s181

RA-011-D1RA00914A-s182

RA-011-D1RA00914A-s183

RA-011-D1RA00914A-s184

RA-011-D1RA00914A-s185

RA-011-D1RA00914A-s186

RA-011-D1RA00914A-s187

RA-011-D1RA00914A-s188

RA-011-D1RA00914A-s189

RA-011-D1RA00914A-s190

RA-011-D1RA00914A-s191

RA-011-D1RA00914A-s192

RA-011-D1RA00914A-s193

RA-011-D1RA00914A-s194

RA-011-D1RA00914A-s195

RA-011-D1RA00914A-s196

RA-011-D1RA00914A-s197

RA-011-D1RA00914A-s198

RA-011-D1RA00914A-s199

RA-011-D1RA00914A-s200

RA-011-D1RA00914A-s201

RA-011-D1RA00914A-s202

RA-011-D1RA00914A-s203

RA-011-D1RA00914A-s204

RA-011-D1RA00914A-s205

RA-011-D1RA00914A-s206

RA-011-D1RA00914A-s207

RA-011-D1RA00914A-s208

RA-011-D1RA00914A-s209

RA-011-D1RA00914A-s210

RA-011-D1RA00914A-s211

RA-011-D1RA00914A-s212

RA-011-D1RA00914A-s213

RA-011-D1RA00914A-s214

RA-011-D1RA00914A-s215

RA-011-D1RA00914A-s216

RA-011-D1RA00914A-s217

RA-011-D1RA00914A-s218

RA-011-D1RA00914A-s219

RA-011-D1RA00914A-s220

RA-011-D1RA00914A-s221

RA-011-D1RA00914A-s222

RA-011-D1RA00914A-s223

RA-011-D1RA00914A-s224

RA-011-D1RA00914A-s225

RA-011-D1RA00914A-s226

RA-011-D1RA00914A-s227

RA-011-D1RA00914A-s228

RA-011-D1RA00914A-s229

RA-011-D1RA00914A-s230

RA-011-D1RA00914A-s231

RA-011-D1RA00914A-s232

RA-011-D1RA00914A-s233

RA-011-D1RA00914A-s234

RA-011-D1RA00914A-s235

RA-011-D1RA00914A-s236

RA-011-D1RA00914A-s237

RA-011-D1RA00914A-s238

RA-011-D1RA00914A-s239

RA-011-D1RA00914A-s240

RA-011-D1RA00914A-s241

RA-011-D1RA00914A-s242

RA-011-D1RA00914A-s243

RA-011-D1RA00914A-s244

RA-011-D1RA00914A-s245

RA-011-D1RA00914A-s246

RA-011-D1RA00914A-s247

RA-011-D1RA00914A-s248

RA-011-D1RA00914A-s249

RA-011-D1RA00914A-s250

RA-011-D1RA00914A-s251

RA-011-D1RA00914A-s252

RA-011-D1RA00914A-s253

RA-011-D1RA00914A-s254

RA-011-D1RA00914A-s255

RA-011-D1RA00914A-s256

RA-011-D1RA00914A-s257

RA-011-D1RA00914A-s258

RA-011-D1RA00914A-s259

RA-011-D1RA00914A-s260

RA-011-D1RA00914A-s261

RA-011-D1RA00914A-s262

RA-011-D1RA00914A-s263

RA-011-D1RA00914A-s264

RA-011-D1RA00914A-s265

RA-011-D1RA00914A-s266

RA-011-D1RA00914A-s267

RA-011-D1RA00914A-s268

RA-011-D1RA00914A-s269

RA-011-D1RA00914A-s270

RA-011-D1RA00914A-s271

RA-011-D1RA00914A-s272

RA-011-D1RA00914A-s273

RA-011-D1RA00914A-s274

RA-011-D1RA00914A-s275

RA-011-D1RA00914A-s276

RA-011-D1RA00914A-s277

RA-011-D1RA00914A-s278

RA-011-D1RA00914A-s279

RA-011-D1RA00914A-s280

RA-011-D1RA00914A-s281

RA-011-D1RA00914A-s282

RA-011-D1RA00914A-s283

RA-011-D1RA00914A-s284

RA-011-D1RA00914A-s285

RA-011-D1RA00914A-s286

RA-011-D1RA00914A-s287

RA-011-D1RA00914A-s288

RA-011-D1RA00914A-s289

RA-011-D1RA00914A-s290

RA-011-D1RA00914A-s291

RA-011-D1RA00914A-s292

RA-011-D1RA00914A-s293

RA-011-D1RA00914A-s294

RA-011-D1RA00914A-s295

RA-011-D1RA00914A-s296

RA-011-D1RA00914A-s297

RA-011-D1RA00914A-s298

RA-011-D1RA00914A-s299

RA-011-D1RA00914A-s300

RA-011-D1RA00914A-s301

RA-011-D1RA00914A-s302

RA-011-D1RA00914A-s303

RA-011-D1RA00914A-s304

RA-011-D1RA00914A-s305

RA-011-D1RA00914A-s306

RA-011-D1RA00914A-s307

RA-011-D1RA00914A-s308

RA-011-D1RA00914A-s309

RA-011-D1RA00914A-s310

RA-011-D1RA00914A-s311

RA-011-D1RA00914A-s312

RA-011-D1RA00914A-s313

RA-011-D1RA00914A-s314

RA-011-D1RA00914A-s315

RA-011-D1RA00914A-s316

RA-011-D1RA00914A-s317

RA-011-D1RA00914A-s318

RA-011-D1RA00914A-s319

RA-011-D1RA00914A-s320

RA-011-D1RA00914A-s321

RA-011-D1RA00914A-s322

RA-011-D1RA00914A-s323

RA-011-D1RA00914A-s324

RA-011-D1RA00914A-s325

RA-011-D1RA00914A-s326

RA-011-D1RA00914A-s327

RA-011-D1RA00914A-s328

RA-011-D1RA00914A-s329

RA-011-D1RA00914A-s330

RA-011-D1RA00914A-s331

RA-011-D1RA00914A-s332

RA-011-D1RA00914A-s333

RA-011-D1RA00914A-s334

RA-011-D1RA00914A-s335

RA-011-D1RA00914A-s336

RA-011-D1RA00914A-s337

RA-011-D1RA00914A-s338

RA-011-D1RA00914A-s339

RA-011-D1RA00914A-s340

RA-011-D1RA00914A-s341

RA-011-D1RA00914A-s342

RA-011-D1RA00914A-s343

RA-011-D1RA00914A-s344

RA-011-D1RA00914A-s345

RA-011-D1RA00914A-s346

RA-011-D1RA00914A-s347

RA-011-D1RA00914A-s348

RA-011-D1RA00914A-s349

RA-011-D1RA00914A-s350

RA-011-D1RA00914A-s351

RA-011-D1RA00914A-s352

RA-011-D1RA00914A-s353

RA-011-D1RA00914A-s354

RA-011-D1RA00914A-s355

RA-011-D1RA00914A-s356

RA-011-D1RA00914A-s357

RA-011-D1RA00914A-s358

RA-011-D1RA00914A-s359

RA-011-D1RA00914A-s360

RA-011-D1RA00914A-s361

RA-011-D1RA00914A-s362

RA-011-D1RA00914A-s363

RA-011-D1RA00914A-s364

RA-011-D1RA00914A-s365

RA-011-D1RA00914A-s366

RA-011-D1RA00914A-s367

RA-011-D1RA00914A-s368

RA-011-D1RA00914A-s369

RA-011-D1RA00914A-s370

RA-011-D1RA00914A-s371

RA-011-D1RA00914A-s372

RA-011-D1RA00914A-s373

RA-011-D1RA00914A-s374

RA-011-D1RA00914A-s375

RA-011-D1RA00914A-s376

RA-011-D1RA00914A-s377

RA-011-D1RA00914A-s378

RA-011-D1RA00914A-s379

RA-011-D1RA00914A-s380

RA-011-D1RA00914A-s381

RA-011-D1RA00914A-s382

RA-011-D1RA00914A-s383

RA-011-D1RA00914A-s384

RA-011-D1RA00914A-s385

RA-011-D1RA00914A-s386

RA-011-D1RA00914A-s387

RA-011-D1RA00914A-s388

RA-011-D1RA00914A-s389

RA-011-D1RA00914A-s390

RA-011-D1RA00914A-s391

RA-011-D1RA00914A-s392

RA-011-D1RA00914A-s393

RA-011-D1RA00914A-s394

RA-011-D1RA00914A-s395

RA-011-D1RA00914A-s396

RA-011-D1RA00914A-s397

RA-011-D1RA00914A-s398

RA-011-D1RA00914A-s399

RA-011-D1RA00914A-s400

RA-011-D1RA00914A-s401

RA-011-D1RA00914A-s402

RA-011-D1RA00914A-s403

RA-011-D1RA00914A-s404

RA-011-D1RA00914A-s405

RA-011-D1RA00914A-s406

RA-011-D1RA00914A-s407

RA-011-D1RA00914A-s408

RA-011-D1RA00914A-s409

RA-011-D1RA00914A-s410

RA-011-D1RA00914A-s411

RA-011-D1RA00914A-s412

RA-011-D1RA00914A-s413

RA-011-D1RA00914A-s414

RA-011-D1RA00914A-s415

RA-011-D1RA00914A-s416

RA-011-D1RA00914A-s417

RA-011-D1RA00914A-s418

RA-011-D1RA00914A-s419

RA-011-D1RA00914A-s420

RA-011-D1RA00914A-s421

RA-011-D1RA00914A-s422

RA-011-D1RA00914A-s423

RA-011-D1RA00914A-s424

RA-011-D1RA00914A-s425

RA-011-D1RA00914A-s426

RA-011-D1RA00914A-s427

RA-011-D1RA00914A-s428

RA-011-D1RA00914A-s429

RA-011-D1RA00914A-s430

RA-011-D1RA00914A-s431

RA-011-D1RA00914A-s432

RA-011-D1RA00914A-s433

RA-011-D1RA00914A-s434

RA-011-D1RA00914A-s435

RA-011-D1RA00914A-s436

RA-011-D1RA00914A-s437

RA-011-D1RA00914A-s438

RA-011-D1RA00914A-s439

RA-011-D1RA00914A-s440

RA-011-D1RA00914A-s441

RA-011-D1RA00914A-s442

RA-011-D1RA00914A-s443

RA-011-D1RA00914A-s444

RA-011-D1RA00914A-s445

RA-011-D1RA00914A-s446

RA-011-D1RA00914A-s447

RA-011-D1RA00914A-s448

RA-011-D1RA00914A-s449

RA-011-D1RA00914A-s450

RA-011-D1RA00914A-s451

RA-011-D1RA00914A-s452

RA-011-D1RA00914A-s453

RA-011-D1RA00914A-s454

RA-011-D1RA00914A-s455

RA-011-D1RA00914A-s456

RA-011-D1RA00914A-s457

RA-011-D1RA00914A-s458

RA-011-D1RA00914A-s459

RA-011-D1RA00914A-s460

RA-011-D1RA00914A-s461

RA-011-D1RA00914A-s462

RA-011-D1RA00914A-s463

RA-011-D1RA00914A-s464

RA-011-D1RA00914A-s465

RA-011-D1RA00914A-s466

RA-011-D1RA00914A-s467

RA-011-D1RA00914A-s468

RA-011-D1RA00914A-s469

RA-011-D1RA00914A-s470

RA-011-D1RA00914A-s471

RA-011-D1RA00914A-s472

RA-011-D1RA00914A-s473

RA-011-D1RA00914A-s474

RA-011-D1RA00914A-s475

RA-011-D1RA00914A-s476

RA-011-D1RA00914A-s477

RA-011-D1RA00914A-s478

RA-011-D1RA00914A-s479

RA-011-D1RA00914A-s480

RA-011-D1RA00914A-s481

RA-011-D1RA00914A-s482

RA-011-D1RA00914A-s483

RA-011-D1RA00914A-s484

RA-011-D1RA00914A-s485

RA-011-D1RA00914A-s486

RA-011-D1RA00914A-s487

RA-011-D1RA00914A-s488

RA-011-D1RA00914A-s489

RA-011-D1RA00914A-s490

RA-011-D1RA00914A-s491

RA-011-D1RA00914A-s492

RA-011-D1RA00914A-s493

RA-011-D1RA00914A-s494

RA-011-D1RA00914A-s495

RA-011-D1RA00914A-s496

RA-011-D1RA00914A-s497

RA-011-D1RA00914A-s498

RA-011-D1RA00914A-s499

RA-011-D1RA00914A-s500

RA-011-D1RA00914A-s501

RA-011-D1RA00914A-s502

RA-011-D1RA00914A-s503

RA-011-D1RA00914A-s504

RA-011-D1RA00914A-s505

RA-011-D1RA00914A-s506

RA-011-D1RA00914A-s507

RA-011-D1RA00914A-s508

RA-011-D1RA00914A-s509

RA-011-D1RA00914A-s510

RA-011-D1RA00914A-s511

RA-011-D1RA00914A-s512

RA-011-D1RA00914A-s513

RA-011-D1RA00914A-s514

RA-011-D1RA00914A-s515

RA-011-D1RA00914A-s516

RA-011-D1RA00914A-s517

RA-011-D1RA00914A-s518

RA-011-D1RA00914A-s519

RA-011-D1RA00914A-s520

RA-011-D1RA00914A-s521

RA-011-D1RA00914A-s522

RA-011-D1RA00914A-s523

RA-011-D1RA00914A-s524

RA-011-D1RA00914A-s525

RA-011-D1RA00914A-s526

RA-011-D1RA00914A-s527

RA-011-D1RA00914A-s528

RA-011-D1RA00914A-s529

RA-011-D1RA00914A-s530

RA-011-D1RA00914A-s531

RA-011-D1RA00914A-s532

RA-011-D1RA00914A-s533

RA-011-D1RA00914A-s534

RA-011-D1RA00914A-s535

RA-011-D1RA00914A-s536

RA-011-D1RA00914A-s537

RA-011-D1RA00914A-s538

RA-011-D1RA00914A-s539

RA-011-D1RA00914A-s540

RA-011-D1RA00914A-s541

RA-011-D1RA00914A-s542

RA-011-D1RA00914A-s543

RA-011-D1RA00914A-s544

RA-011-D1RA00914A-s545

RA-011-D1RA00914A-s546

RA-011-D1RA00914A-s547

RA-011-D1RA00914A-s548

RA-011-D1RA00914A-s549

RA-011-D1RA00914A-s550

RA-011-D1RA00914A-s551

RA-011-D1RA00914A-s552

RA-011-D1RA00914A-s553

RA-011-D1RA00914A-s554

RA-011-D1RA00914A-s555

RA-011-D1RA00914A-s556

RA-011-D1RA00914A-s557

RA-011-D1RA00914A-s558

RA-011-D1RA00914A-s559

RA-011-D1RA00914A-s560

RA-011-D1RA00914A-s561

RA-011-D1RA00914A-s562

RA-011-D1RA00914A-s563

RA-011-D1RA00914A-s564

RA-011-D1RA00914A-s565

RA-011-D1RA00914A-s566

RA-011-D1RA00914A-s567

RA-011-D1RA00914A-s568

RA-011-D1RA00914A-s569

RA-011-D1RA00914A-s570

RA-011-D1RA00914A-s571

RA-011-D1RA00914A-s572

RA-011-D1RA00914A-s573

RA-011-D1RA00914A-s574

RA-011-D1RA00914A-s575

RA-011-D1RA00914A-s576

RA-011-D1RA00914A-s577

RA-011-D1RA00914A-s578

RA-011-D1RA00914A-s579

RA-011-D1RA00914A-s580

RA-011-D1RA00914A-s581

RA-011-D1RA00914A-s582

RA-011-D1RA00914A-s583

RA-011-D1RA00914A-s584

RA-011-D1RA00914A-s585

RA-011-D1RA00914A-s586

RA-011-D1RA00914A-s587

RA-011-D1RA00914A-s588

RA-011-D1RA00914A-s589

RA-011-D1RA00914A-s590

RA-011-D1RA00914A-s591

RA-011-D1RA00914A-s592

RA-011-D1RA00914A-s593

RA-011-D1RA00914A-s594

RA-011-D1RA00914A-s595

RA-011-D1RA00914A-s596

RA-011-D1RA00914A-s597

RA-011-D1RA00914A-s598

RA-011-D1RA00914A-s599

RA-011-D1RA00914A-s600

RA-011-D1RA00914A-s601

RA-011-D1RA00914A-s602

RA-011-D1RA00914A-s603

RA-011-D1RA00914A-s604

RA-011-D1RA00914A-s605

RA-011-D1RA00914A-s606

RA-011-D1RA00914A-s607

RA-011-D1RA00914A-s608

RA-011-D1RA00914A-s609

RA-011-D1RA00914A-s610

RA-011-D1RA00914A-s611

RA-011-D1RA00914A-s612

RA-011-D1RA00914A-s613

RA-011-D1RA00914A-s614

RA-011-D1RA00914A-s615

RA-011-D1RA00914A-s616

RA-011-D1RA00914A-s617

RA-011-D1RA00914A-s618

RA-011-D1RA00914A-s619

RA-011-D1RA00914A-s620

RA-011-D1RA00914A-s621

RA-011-D1RA00914A-s622

RA-011-D1RA00914A-s623

RA-011-D1RA00914A-s624

RA-011-D1RA00914A-s625

RA-011-D1RA00914A-s626

RA-011-D1RA00914A-s627

RA-011-D1RA00914A-s628

RA-011-D1RA00914A-s629

RA-011-D1RA00914A-s630

RA-011-D1RA00914A-s631

RA-011-D1RA00914A-s632

RA-011-D1RA00914A-s633

RA-011-D1RA00914A-s634

RA-011-D1RA00914A-s635

RA-011-D1RA00914A-s636

RA-011-D1RA00914A-s637

RA-011-D1RA00914A-s638

RA-011-D1RA00914A-s639

RA-011-D1RA00914A-s640

RA-011-D1RA00914A-s641

RA-011-D1RA00914A-s642

RA-011-D1RA00914A-s643

RA-011-D1RA00914A-s644

RA-011-D1RA00914A-s645

RA-011-D1RA00914A-s646

RA-011-D1RA00914A-s647

RA-011-D1RA00914A-s648

RA-011-D1RA00914A-s649

RA-011-D1RA00914A-s650

RA-011-D1RA00914A-s651

RA-011-D1RA00914A-s652

RA-011-D1RA00914A-s653

RA-011-D1RA00914A-s654

RA-011-D1RA00914A-s655

RA-011-D1RA00914A-s656

RA-011-D1RA00914A-s657

RA-011-D1RA00914A-s658

RA-011-D1RA00914A-s659

RA-011-D1RA00914A-s660

RA-011-D1RA00914A-s661

RA-011-D1RA00914A-s662

RA-011-D1RA00914A-s663

RA-011-D1RA00914A-s664

RA-011-D1RA00914A-s665

RA-011-D1RA00914A-s666

RA-011-D1RA00914A-s667

RA-011-D1RA00914A-s668

RA-011-D1RA00914A-s669

RA-011-D1RA00914A-s670

RA-011-D1RA00914A-s671

RA-011-D1RA00914A-s672

RA-011-D1RA00914A-s673

RA-011-D1RA00914A-s674

RA-011-D1RA00914A-s675

RA-011-D1RA00914A-s676

RA-011-D1RA00914A-s677

RA-011-D1RA00914A-s678

RA-011-D1RA00914A-s679

RA-011-D1RA00914A-s680

RA-011-D1RA00914A-s681

RA-011-D1RA00914A-s682

RA-011-D1RA00914A-s683

RA-011-D1RA00914A-s684

RA-011-D1RA00914A-s685

RA-011-D1RA00914A-s686

RA-011-D1RA00914A-s687

RA-011-D1RA00914A-s688

RA-011-D1RA00914A-s689

RA-011-D1RA00914A-s690

RA-011-D1RA00914A-s691

RA-011-D1RA00914A-s692

RA-011-D1RA00914A-s693

RA-011-D1RA00914A-s694

RA-011-D1RA00914A-s695

RA-011-D1RA00914A-s696

RA-011-D1RA00914A-s697

RA-011-D1RA00914A-s698

RA-011-D1RA00914A-s699

RA-011-D1RA00914A-s700

RA-011-D1RA00914A-s701

RA-011-D1RA00914A-s702

RA-011-D1RA00914A-s703

RA-011-D1RA00914A-s704

RA-011-D1RA00914A-s705

RA-011-D1RA00914A-s706

RA-011-D1RA00914A-s707

RA-011-D1RA00914A-s708

RA-011-D1RA00914A-s709

RA-011-D1RA00914A-s710

RA-011-D1RA00914A-s711

RA-011-D1RA00914A-s712

RA-011-D1RA00914A-s713

RA-011-D1RA00914A-s714

RA-011-D1RA00914A-s715

RA-011-D1RA00914A-s716

RA-011-D1RA00914A-s717

RA-011-D1RA00914A-s718

RA-011-D1RA00914A-s719

RA-011-D1RA00914A-s720

RA-011-D1RA00914A-s721

RA-011-D1RA00914A-s722

RA-011-D1RA00914A-s723

RA-011-D1RA00914A-s724

RA-011-D1RA00914A-s725

RA-011-D1RA00914A-s726

RA-011-D1RA00914A-s727

RA-011-D1RA00914A-s728

RA-011-D1RA00914A-s729

RA-011-D1RA00914A-s730

RA-011-D1RA00914A-s731

RA-011-D1RA00914A-s732

RA-011-D1RA00914A-s733

RA-011-D1RA00914A-s734

RA-011-D1RA00914A-s735

RA-011-D1RA00914A-s736

RA-011-D1RA00914A-s737

RA-011-D1RA00914A-s738

RA-011-D1RA00914A-s739

RA-011-D1RA00914A-s740

RA-011-D1RA00914A-s741

RA-011-D1RA00914A-s742

RA-011-D1RA00914A-s743

RA-011-D1RA00914A-s744

RA-011-D1RA00914A-s745

RA-011-D1RA00914A-s746

RA-011-D1RA00914A-s747

RA-011-D1RA00914A-s748

RA-011-D1RA00914A-s749

RA-011-D1RA00914A-s750

RA-011-D1RA00914A-s751

RA-011-D1RA00914A-s752

RA-011-D1RA00914A-s753

RA-011-D1RA00914A-s754

RA-011-D1RA00914A-s755

RA-011-D1RA00914A-s756

RA-011-D1RA00914A-s757

RA-011-D1RA00914A-s758

RA-011-D1RA00914A-s759

RA-011-D1RA00914A-s760

RA-011-D1RA00914A-s761

RA-011-D1RA00914A-s762

RA-011-D1RA00914A-s763

RA-011-D1RA00914A-s764

RA-011-D1RA00914A-s765

RA-011-D1RA00914A-s766

RA-011-D1RA00914A-s767

RA-011-D1RA00914A-s768

RA-011-D1RA00914A-s769

RA-011-D1RA00914A-s770

RA-011-D1RA00914A-s771

RA-011-D1RA00914A-s772

RA-011-D1RA00914A-s773

RA-011-D1RA00914A-s774

RA-011-D1RA00914A-s775

RA-011-D1RA00914A-s776

RA-011-D1RA00914A-s777

RA-011-D1RA00914A-s778

RA-011-D1RA00914A-s779

RA-011-D1RA00914A-s780

RA-011-D1RA00914A-s781

RA-011-D1RA00914A-s782

RA-011-D1RA00914A-s783

RA-011-D1RA00914A-s784

RA-011-D1RA00914A-s785

RA-011-D1RA00914A-s786

RA-011-D1RA00914A-s787

RA-011-D1RA00914A-s788

RA-011-D1RA00914A-s789

RA-011-D1RA00914A-s790

RA-011-D1RA00914A-s791

RA-011-D1RA00914A-s792

RA-011-D1RA00914A-s793

RA-011-D1RA00914A-s794

RA-011-D1RA00914A-s795

RA-011-D1RA00914A-s796

RA-011-D1RA00914A-s797

RA-011-D1RA00914A-s798

RA-011-D1RA00914A-s799

RA-011-D1RA00914A-s800

RA-011-D1RA00914A-s801

RA-011-D1RA00914A-s802

RA-011-D1RA00914A-s803

RA-011-D1RA00914A-s804

RA-011-D1RA00914A-s805

RA-011-D1RA00914A-s806

RA-011-D1RA00914A-s807

RA-011-D1RA00914A-s808

RA-011-D1RA00914A-s809

RA-011-D1RA00914A-s810

RA-011-D1RA00914A-s811

RA-011-D1RA00914A-s812

RA-011-D1RA00914A-s813

RA-011-D1RA00914A-s814

RA-011-D1RA00914A-s815

RA-011-D1RA00914A-s816

RA-011-D1RA00914A-s817

RA-011-D1RA00914A-s818

RA-011-D1RA00914A-s819

RA-011-D1RA00914A-s820

RA-011-D1RA00914A-s821

RA-011-D1RA00914A-s822

RA-011-D1RA00914A-s823

RA-011-D1RA00914A-s824

RA-011-D1RA00914A-s825

RA-011-D1RA00914A-s826

RA-011-D1RA00914A-s827

RA-011-D1RA00914A-s828

RA-011-D1RA00914A-s829

RA-011-D1RA00914A-s830

RA-011-D1RA00914A-s831

RA-011-D1RA00914A-s832

RA-011-D1RA00914A-s833

RA-011-D1RA00914A-s834

RA-011-D1RA00914A-s835

RA-011-D1RA00914A-s836

RA-011-D1RA00914A-s837

RA-011-D1RA00914A-s838

RA-011-D1RA00914A-s839

RA-011-D1RA00914A-s840

RA-011-D1RA00914A-s841

RA-011-D1RA00914A-s842

RA-011-D1RA00914A-s843

RA-011-D1RA00914A-s844

RA-011-D1RA00914A-s845

RA-011-D1RA00914A-s846

RA-011-D1RA00914A-s847

RA-011-D1RA00914A-s848

RA-011-D1RA00914A-s849

RA-011-D1RA00914A-s850

RA-011-D1RA00914A-s851

RA-011-D1RA00914A-s852

RA-011-D1RA00914A-s853

RA-011-D1RA00914A-s854

RA-011-D1RA00914A-s855

RA-011-D1RA00914A-s856

RA-011-D1RA00914A-s857

RA-011-D1RA00914A-s858

RA-011-D1RA00914A-s859

RA-011-D1RA00914A-s860

RA-011-D1RA00914A-s861

RA-011-D1RA00914A-s862

RA-011-D1RA00914A-s863

RA-011-D1RA00914A-s864

RA-011-D1RA00914A-s865

RA-011-D1RA00914A-s866

RA-011-D1RA00914A-s867

RA-011-D1RA00914A-s868

RA-011-D1RA00914A-s869

RA-011-D1RA00914A-s870

RA-011-D1RA00914A-s871

RA-011-D1RA00914A-s872

RA-011-D1RA00914A-s873

RA-011-D1RA00914A-s874

RA-011-D1RA00914A-s875

RA-011-D1RA00914A-s876

RA-011-D1RA00914A-s877

RA-011-D1RA00914A-s878

RA-011-D1RA00914A-s879

RA-011-D1RA00914A-s880

RA-011-D1RA00914A-s881

RA-011-D1RA00914A-s882

RA-011-D1RA00914A-s883

RA-011-D1RA00914A-s884

RA-011-D1RA00914A-s885

RA-011-D1RA00914A-s886

RA-011-D1RA00914A-s887

RA-011-D1RA00914A-s888

RA-011-D1RA00914A-s889

RA-011-D1RA00914A-s890

RA-011-D1RA00914A-s891

RA-011-D1RA00914A-s892

RA-011-D1RA00914A-s893

RA-011-D1RA00914A-s894

RA-011-D1RA00914A-s895

RA-011-D1RA00914A-s896

RA-011-D1RA00914A-s897

RA-011-D1RA00914A-s898

RA-011-D1RA00914A-s899

RA-011-D1RA00914A-s900

RA-011-D1RA00914A-s901

RA-011-D1RA00914A-s902

RA-011-D1RA00914A-s903

RA-011-D1RA00914A-s904

RA-011-D1RA00914A-s905

RA-011-D1RA00914A-s906

RA-011-D1RA00914A-s907

RA-011-D1RA00914A-s908

RA-011-D1RA00914A-s909

RA-011-D1RA00914A-s910

RA-011-D1RA00914A-s911

RA-011-D1RA00914A-s912

RA-011-D1RA00914A-s913

RA-011-D1RA00914A-s914

RA-011-D1RA00914A-s915

RA-011-D1RA00914A-s916

RA-011-D1RA00914A-s917

RA-011-D1RA00914A-s918

RA-011-D1RA00914A-s919

RA-011-D1RA00914A-s920

RA-011-D1RA00914A-s921

RA-011-D1RA00914A-s922

RA-011-D1RA00914A-s923

RA-011-D1RA00914A-s924

RA-011-D1RA00914A-s925

RA-011-D1RA00914A-s926

RA-011-D1RA00914A-s927

RA-011-D1RA00914A-s928

RA-011-D1RA00914A-s929

RA-011-D1RA00914A-s930

RA-011-D1RA00914A-s931

RA-011-D1RA00914A-s932

RA-011-D1RA00914A-s933

RA-011-D1RA00914A-s934

RA-011-D1RA00914A-s935

RA-011-D1RA00914A-s936

RA-011-D1RA00914A-s937

RA-011-D1RA00914A-s938

RA-011-D1RA00914A-s939

RA-011-D1RA00914A-s940

RA-011-D1RA00914A-s941

RA-011-D1RA00914A-s942

RA-011-D1RA00914A-s943

RA-011-D1RA00914A-s944

RA-011-D1RA00914A-s945

RA-011-D1RA00914A-s946

RA-011-D1RA00914A-s947

RA-011-D1RA00914A-s948

RA-011-D1RA00914A-s949

RA-011-D1RA00914A-s950

RA-011-D1RA00914A-s951

RA-011-D1RA00914A-s952

RA-011-D1RA00914A-s953

RA-011-D1RA00914A-s954

RA-011-D1RA00914A-s955

RA-011-D1RA00914A-s956

RA-011-D1RA00914A-s957

RA-011-D1RA00914A-s958

RA-011-D1RA00914A-s959

RA-011-D1RA00914A-s960

RA-011-D1RA00914A-s961

RA-011-D1RA00914A-s962

RA-011-D1RA00914A-s963

RA-011-D1RA00914A-s964

RA-011-D1RA00914A-s965

RA-011-D1RA00914A-s966

RA-011-D1RA00914A-s967

RA-011-D1RA00914A-s968

RA-011-D1RA00914A-s969

RA-011-D1RA00914A-s970

RA-011-D1RA00914A-s971

RA-011-D1RA00914A-s972

RA-011-D1RA00914A-s973

RA-011-D1RA00914A-s974

RA-011-D1RA00914A-s975

RA-011-D1RA00914A-s976

RA-011-D1RA00914A-s977

RA-011-D1RA00914A-s978

RA-011-D1RA00914A-s979

RA-011-D1RA00914A-s980

RA-011-D1RA00914A-s981

RA-011-D1RA00914A-s982

RA-011-D1RA00914A-s983

RA-011-D1RA00914A-s984

RA-011-D1RA00914A-s985

RA-011-D1RA00914A-s986

RA-011-D1RA00914A-s987

RA-011-D1RA00914A-s988

RA-011-D1RA00914A-s989

RA-011-D1RA00914A-s990

RA-011-D1RA00914A-s991

RA-011-D1RA00914A-s992

RA-011-D1RA00914A-s993

RA-011-D1RA00914A-s994

RA-011-D1RA00914A-s995

RA-011-D1RA00914A-s996

RA-011-D1RA00914A-s997

RA-011-D1RA00914A-s998

RA-011-D1RA00914A-s999

RA-011-D1RA00914A-s1000

RA-011-D1RA00914A-s1001

RA-011-D1RA00914A-s1002

RA-011-D1RA00914A-s1003

RA-011-D1RA00914A-s1004

RA-011-D1RA00914A-s1005

RA-011-D1RA00914A-s1006

RA-011-D1RA00914A-s1007

RA-011-D1RA00914A-s1008

RA-011-D1RA00914A-s1009

RA-011-D1RA00914A-s1010

RA-011-D1RA00914A-s1011

RA-011-D1RA00914A-s1012

RA-011-D1RA00914A-s1013

RA-011-D1RA00914A-s1014

RA-011-D1RA00914A-s1015

RA-011-D1RA00914A-s1016

RA-011-D1RA00914A-s1017

RA-011-D1RA00914A-s1018

RA-011-D1RA00914A-s1019

RA-011-D1RA00914A-s1020

RA-011-D1RA00914A-s1021

RA-011-D1RA00914A-s1022

RA-011-D1RA00914A-s1023

RA-011-D1RA00914A-s1024

RA-011-D1RA00914A-s1025

RA-011-D1RA00914A-s1026

RA-011-D1RA00914A-s1027

RA-011-D1RA00914A-s1028

RA-011-D1RA00914A-s1029

RA-011-D1RA00914A-s1030

RA-011-D1RA00914A-s1031

RA-011-D1RA00914A-s1032

RA-011-D1RA00914A-s1033

RA-011-D1RA00914A-s1034

RA-011-D1RA00914A-s1035

RA-011-D1RA00914A-s1036

RA-011-D1RA00914A-s1037

RA-011-D1RA00914A-s1038

RA-011-D1RA00914A-s1039

RA-011-D1RA00914A-s1040

RA-011-D1RA00914A-s1041

RA-011-D1RA00914A-s1042

RA-011-D1RA00914A-s1043

RA-011-D1RA00914A-s1044

RA-011-D1RA00914A-s1045

RA-011-D1RA00914A-s1046

RA-011-D1RA00914A-s1047

RA-011-D1RA00914A-s1048

RA-011-D1RA00914A-s1049

RA-011-D1RA00914A-s1050

RA-011-D1RA00914A-s1051

RA-011-D1RA00914A-s1052

RA-011-D1RA00914A-s1053

RA-011-D1RA00914A-s1054

RA-011-D1RA00914A-s1055

RA-011-D1RA00914A-s1056

RA-011-D1RA00914A-s1057

RA-011-D1RA00914A-s1058

RA-011-D1RA00914A-s1059

RA-011-D1RA00914A-s1060

RA-011-D1RA00914A-s1061

RA-011-D1RA00914A-s1062

RA-011-D1RA00914A-s1063

RA-011-D1RA00914A-s1064

RA-011-D1RA00914A-s1065

RA-011-D1RA00914A-s1066

RA-011-D1RA00914A-s1067

RA-011-D1RA00914A-s1068

RA-011-D1RA00914A-s1069

RA-011-D1RA00914A-s1070

RA-011-D1RA00914A-s1071

RA-011-D1RA00914A-s1072

RA-011-D1RA00914A-s1073

RA-011-D1RA00914A-s1074

RA-011-D1RA00914A-s1075

RA-011-D1RA00914A-s1076

RA-011-D1RA00914A-s1077

RA-011-D1RA00914A-s1078

RA-011-D1RA00914A-s1079

RA-011-D1RA00914A-s1080

RA-011-D1RA00914A-s1081

RA-011-D1RA00914A-s1082

RA-011-D1RA00914A-s1083

RA-011-D1RA00914A-s1084

RA-011-D1RA00914A-s1085

RA-011-D1RA00914A-s1086

RA-011-D1RA00914A-s1087

RA-011-D1RA00914A-s1088

RA-011-D1RA00914A-s1089

RA-011-D1RA00914A-s1090

RA-011-D1RA00914A-s1091

RA-011-D1RA00914A-s1092

RA-011-D1RA00914A-s1093

RA-011-D1RA00914A-s1094

RA-011-D1RA00914A-s1095

RA-011-D1RA00914A-s1096

RA-011-D1RA00914A-s1097

RA-011-D1RA00914A-s1098

RA-011-D1RA00914A-s1099

RA-011-D1RA00914A-s1100

RA-011-D1RA00914A-s1101

RA-011-D1RA00914A-s1102

RA-011-D1RA00914A-s1103

RA-011-D1RA00914A-s1104

RA-011-D1RA00914A-s1105

RA-011-D1RA00914A-s1106

RA-011-D1RA00914A-s1107

RA-011-D1RA00914A-s1108

RA-011-D1RA00914A-s1109

RA-011-D1RA00914A-s1110

RA-011-D1RA00914A-s1111

RA-011-D1RA00914A-s1112

RA-011-D1RA00914A-s1113

RA-011-D1RA00914A-s1114

RA-011-D1RA00914A-s1115

RA-011-D1RA00914A-s1116

RA-011-D1RA00914A-s1117

RA-011-D1RA00914A-s1118

RA-011-D1RA00914A-s1119

RA-011-D1RA00914A-s1120

RA-011-D1RA00914A-s1121

RA-011-D1RA00914A-s1122

RA-011-D1RA00914A-s1123

RA-011-D1RA00914A-s1124

RA-011-D1RA00914A-s1125

RA-011-D1RA00914A-s1126

RA-011-D1RA00914A-s1127

RA-011-D1RA00914A-s1128

RA-011-D1RA00914A-s1129

RA-011-D1RA00914A-s1130

RA-011-D1RA00914A-s1131

RA-011-D1RA00914A-s1132

RA-011-D1RA00914A-s1133

RA-011-D1RA00914A-s1134

RA-011-D1RA00914A-s1135

RA-011-D1RA00914A-s1136

RA-011-D1RA00914A-s1137

RA-011-D1RA00914A-s1138

RA-011-D1RA00914A-s1139

RA-011-D1RA00914A-s1140

RA-011-D1RA00914A-s1141

RA-011-D1RA00914A-s1142

RA-011-D1RA00914A-s1143

RA-011-D1RA00914A-s1144

RA-011-D1RA00914A-s1145

RA-011-D1RA00914A-s1146

RA-011-D1RA00914A-s1147

RA-011-D1RA00914A-s1148

RA-011-D1RA00914A-s1149

RA-011-D1RA00914A-s1150

RA-011-D1RA00914A-s1151

RA-011-D1RA00914A-s1152

RA-011-D1RA00914A-s1153

RA-011-D1RA00914A-s1154

RA-011-D1RA00914A-s1155

RA-011-D1RA00914A-s1156

RA-011-D1RA00914A-s1157

RA-011-D1RA00914A-s1158

RA-011-D1RA00914A-s1159

RA-011-D1RA00914A-s1160

RA-011-D1RA00914A-s1161

RA-011-D1RA00914A-s1162

RA-011-D1RA00914A-s1163

RA-011-D1RA00914A-s1164

RA-011-D1RA00914A-s1165

RA-011-D1RA00914A-s1166

RA-011-D1RA00914A-s1167

RA-011-D1RA00914A-s1168

RA-011-D1RA00914A-s1169

RA-011-D1RA00914A-s1170

RA-011-D1RA00914A-s1171

RA-011-D1RA00914A-s1172

RA-011-D1RA00914A-s1173

RA-011-D1RA00914A-s1174

RA-011-D1RA00914A-s1175

RA-011-D1RA00914A-s1176

RA-011-D1RA00914A-s1177

RA-011-D1RA00914A-s1178

RA-011-D1RA00914A-s1179

RA-011-D1RA00914A-s1180

RA-011-D1RA00914A-s1181

RA-011-D1RA00914A-s1182

RA-011-D1RA00914A-s1183

RA-011-D1RA00914A-s1184

RA-011-D1RA00914A-s1185

RA-011-D1RA00914A-s1186

RA-011-D1RA00914A-s1187

RA-011-D1RA00914A-s1188

RA-011-D1RA00914A-s1189

RA-011-D1RA00914A-s1190

RA-011-D1RA00914A-s1191

RA-011-D1RA00914A-s1192

RA-011-D1RA00914A-s1193

RA-011-D1RA00914A-s1194

RA-011-D1RA00914A-s1195

RA-011-D1RA00914A-s1196

RA-011-D1RA00914A-s1197

RA-011-D1RA00914A-s1198

RA-011-D1RA00914A-s1199

RA-011-D1RA00914A-s1200

RA-011-D1RA00914A-s1201

RA-011-D1RA00914A-s1202

RA-011-D1RA00914A-s1203

RA-011-D1RA00914A-s1204

RA-011-D1RA00914A-s1205

RA-011-D1RA00914A-s1206

RA-011-D1RA00914A-s1207

RA-011-D1RA00914A-s1208

RA-011-D1RA00914A-s1209

RA-011-D1RA00914A-s1210

RA-011-D1RA00914A-s1211

RA-011-D1RA00914A-s1212

RA-011-D1RA00914A-s1213

RA-011-D1RA00914A-s1214

RA-011-D1RA00914A-s1215

RA-011-D1RA00914A-s1216

RA-011-D1RA00914A-s1217

RA-011-D1RA00914A-s1218

RA-011-D1RA00914A-s1219

RA-011-D1RA00914A-s1220

RA-011-D1RA00914A-s1221

RA-011-D1RA00914A-s1222

RA-011-D1RA00914A-s1223

RA-011-D1RA00914A-s1224

RA-011-D1RA00914A-s1225

RA-011-D1RA00914A-s1226

RA-011-D1RA00914A-s1227

RA-011-D1RA00914A-s1228

RA-011-D1RA00914A-s1229

RA-011-D1RA00914A-s1230

RA-011-D1RA00914A-s1231

RA-011-D1RA00914A-s1232

RA-011-D1RA00914A-s1233

RA-011-D1RA00914A-s1234

RA-011-D1RA00914A-s1235

RA-011-D1RA00914A-s1236

RA-011-D1RA00914A-s1237

RA-011-D1RA00914A-s1238

RA-011-D1RA00914A-s1239

RA-011-D1RA00914A-s1240

RA-011-D1RA00914A-s1241

RA-011-D1RA00914A-s1242

RA-011-D1RA00914A-s1243

RA-011-D1RA00914A-s1244

RA-011-D1RA00914A-s1245

RA-011-D1RA00914A-s1246

RA-011-D1RA00914A-s1247

RA-011-D1RA00914A-s1248

RA-011-D1RA00914A-s1249

RA-011-D1RA00914A-s1250

RA-011-D1RA00914A-s1251

RA-011-D1RA00914A-s1252

RA-011-D1RA00914A-s1253

RA-011-D1RA00914A-s1254

RA-011-D1RA00914A-s1255

RA-011-D1RA00914A-s1256

RA-011-D1RA00914A-s1257

RA-011-D1RA00914A-s1258

RA-011-D1RA00914A-s1259

RA-011-D1RA00914A-s1260

RA-011-D1RA00914A-s1261

RA-011-D1RA00914A-s1262

RA-011-D1RA00914A-s1263

RA-011-D1RA00914A-s1264

RA-011-D1RA00914A-s1265

RA-011-D1RA00914A-s1266

RA-011-D1RA00914A-s1267

RA-011-D1RA00914A-s1268

RA-011-D1RA00914A-s1269

RA-011-D1RA00914A-s1270

RA-011-D1RA00914A-s1271

RA-011-D1RA00914A-s1272

RA-011-D1RA00914A-s1273

RA-011-D1RA00914A-s1274

RA-011-D1RA00914A-s1275

RA-011-D1RA00914A-s1276

RA-011-D1RA00914A-s1277

RA-011-D1RA00914A-s1278

RA-011-D1RA00914A-s1279

RA-011-D1RA00914A-s1280

RA-011-D1RA00914A-s1281

RA-011-D1RA00914A-s1282

RA-011-D1RA00914A-s1283

RA-011-D1RA00914A-s1284

RA-011-D1RA00914A-s1285

RA-011-D1RA00914A-s1286

RA-011-D1RA00914A-s1287

RA-011-D1RA00914A-s1288

RA-011-D1RA00914A-s1289

RA-011-D1RA00914A-s1290

RA-011-D1RA00914A-s1291

RA-011-D1RA00914A-s1292

RA-011-D1RA00914A-s1293

RA-011-D1RA00914A-s1294

RA-011-D1RA00914A-s1295

RA-011-D1RA00914A-s1296

RA-011-D1RA00914A-s1297

RA-011-D1RA00914A-s1298

RA-011-D1RA00914A-s1299

RA-011-D1RA00914A-s1300

RA-011-D1RA00914A-s1301

RA-011-D1RA00914A-s1302

RA-011-D1RA00914A-s1303

RA-011-D1RA00914A-s1304

RA-011-D1RA00914A-s1305

RA-011-D1RA00914A-s1306

RA-011-D1RA00914A-s1307

RA-011-D1RA00914A-s1308

RA-011-D1RA00914A-s1309

RA-011-D1RA00914A-s1310

RA-011-D1RA00914A-s1311

RA-011-D1RA00914A-s1312

RA-011-D1RA00914A-s1313

RA-011-D1RA00914A-s1314

RA-011-D1RA00914A-s1315

RA-011-D1RA00914A-s1316

RA-011-D1RA00914A-s1317

RA-011-D1RA00914A-s1318

RA-011-D1RA00914A-s1319

RA-011-D1RA00914A-s1320

RA-011-D1RA00914A-s1321

RA-011-D1RA00914A-s1322

RA-011-D1RA00914A-s1323

RA-011-D1RA00914A-s1324

RA-011-D1RA00914A-s1325

RA-011-D1RA00914A-s1326

RA-011-D1RA00914A-s1327

RA-011-D1RA00914A-s1328

RA-011-D1RA00914A-s1329

RA-011-D1RA00914A-s1330

RA-011-D1RA00914A-s1331

RA-011-D1RA00914A-s1332

RA-011-D1RA00914A-s1333

RA-011-D1RA00914A-s1334

RA-011-D1RA00914A-s1335

RA-011-D1RA00914A-s1336

RA-011-D1RA00914A-s1337

RA-011-D1RA00914A-s1338

RA-011-D1RA00914A-s1339

RA-011-D1RA00914A-s1340

RA-011-D1RA00914A-s1341

RA-011-D1RA00914A-s1342

RA-011-D1RA00914A-s1343

RA-011-D1RA00914A-s1344

RA-011-D1RA00914A-s1345

RA-011-D1RA00914A-s1346

RA-011-D1RA00914A-s1347

RA-011-D1RA00914A-s1348

RA-011-D1RA00914A-s1349

RA-011-D1RA00914A-s1350

RA-011-D1RA00914A-s1351

RA-011-D1RA00914A-s1352

RA-011-D1RA00914A-s1353

RA-011-D1RA00914A-s1354

RA-011-D1RA00914A-s1355

RA-011-D1RA00914A-s1356

RA-011-D1RA00914A-s1357

RA-011-D1RA00914A-s1358

RA-011-D1RA00914A-s1359

RA-011-D1RA00914A-s1360

RA-011-D1RA00914A-s1361

RA-011-D1RA00914A-s1362

RA-011-D1RA00914A-s1363

RA-011-D1RA00914A-s1364

RA-011-D1RA00914A-s1365

RA-011-D1RA00914A-s1366

RA-011-D1RA00914A-s1367

RA-011-D1RA00914A-s1368

RA-011-D1RA00914A-s1369

RA-011-D1RA00914A-s1370

RA-011-D1RA00914A-s1371

RA-011-D1RA00914A-s1372

RA-011-D1RA00914A-s1373

RA-011-D1RA00914A-s1374

RA-011-D1RA00914A-s1375

RA-011-D1RA00914A-s1376

RA-011-D1RA00914A-s1377

RA-011-D1RA00914A-s1378

RA-011-D1RA00914A-s1379

RA-011-D1RA00914A-s1380

RA-011-D1RA00914A-s1381

RA-011-D1RA00914A-s1382

RA-011-D1RA00914A-s1383

RA-011-D1RA00914A-s1384

RA-011-D1RA00914A-s1385

RA-011-D1RA00914A-s1386

RA-011-D1RA00914A-s1387

RA-011-D1RA00914A-s1388

RA-011-D1RA00914A-s1389

RA-011-D1RA00914A-s1390

RA-011-D1RA00914A-s1391

RA-011-D1RA00914A-s1392

RA-011-D1RA00914A-s1393

RA-011-D1RA00914A-s1394

RA-011-D1RA00914A-s1395

RA-011-D1RA00914A-s1396

RA-011-D1RA00914A-s1397

RA-011-D1RA00914A-s1398

RA-011-D1RA00914A-s1399

RA-011-D1RA00914A-s1400

RA-011-D1RA00914A-s1401

RA-011-D1RA00914A-s1402

RA-011-D1RA00914A-s1403

RA-011-D1RA00914A-s1404

RA-011-D1RA00914A-s1405

RA-011-D1RA00914A-s1406

RA-011-D1RA00914A-s1407

RA-011-D1RA00914A-s1408

RA-011-D1RA00914A-s1409

RA-011-D1RA00914A-s1410

RA-011-D1RA00914A-s1411

RA-011-D1RA00914A-s1412

RA-011-D1RA00914A-s1413

RA-011-D1RA00914A-s1414

RA-011-D1RA00914A-s1415

RA-011-D1RA00914A-s1416

RA-011-D1RA00914A-s1417

RA-011-D1RA00914A-s1418

RA-011-D1RA00914A-s1419

RA-011-D1RA00914A-s1420

RA-011-D1RA00914A-s1421

RA-011-D1RA00914A-s1422

RA-011-D1RA00914A-s1423

RA-011-D1RA00914A-s1424

RA-011-D1RA00914A-s1425

RA-011-D1RA00914A-s1426

RA-011-D1RA00914A-s1427

RA-011-D1RA00914A-s1428

RA-011-D1RA00914A-s1429

RA-011-D1RA00914A-s1430

RA-011-D1RA00914A-s1431

RA-011-D1RA00914A-s1432

RA-011-D1RA00914A-s1433

RA-011-D1RA00914A-s1434

RA-011-D1RA00914A-s1435

RA-011-D1RA00914A-s1436

RA-011-D1RA00914A-s1437

RA-011-D1RA00914A-s1438

RA-011-D1RA00914A-s1439

RA-011-D1RA00914A-s1440

RA-011-D1RA00914A-s1441

RA-011-D1RA00914A-s1442

RA-011-D1RA00914A-s1443

RA-011-D1RA00914A-s1444

RA-011-D1RA00914A-s1445

RA-011-D1RA00914A-s1446

RA-011-D1RA00914A-s1447

RA-011-D1RA00914A-s1448

RA-011-D1RA00914A-s1449

RA-011-D1RA00914A-s1450

RA-011-D1RA00914A-s1451

RA-011-D1RA00914A-s1452

RA-011-D1RA00914A-s1453

RA-011-D1RA00914A-s1454

RA-011-D1RA00914A-s1455

RA-011-D1RA00914A-s1456

RA-011-D1RA00914A-s1457

RA-011-D1RA00914A-s1458

RA-011-D1RA00914A-s1459

RA-011-D1RA00914A-s1460

RA-011-D1RA00914A-s1461

RA-011-D1RA00914A-s1462

RA-011-D1RA00914A-s1463

RA-011-D1RA00914A-s1464

RA-011-D1RA00914A-s1465

RA-011-D1RA00914A-s1466

RA-011-D1RA00914A-s1467

RA-011-D1RA00914A-s1468

RA-011-D1RA00914A-s1469

RA-011-D1RA00914A-s1470

RA-011-D1RA00914A-s1471

RA-011-D1RA00914A-s1472

RA-011-D1RA00914A-s1473

RA-011-D1RA00914A-s1474

RA-011-D1RA00914A-s1475

RA-011-D1RA00914A-s1476

RA-011-D1RA00914A-s1477

RA-011-D1RA00914A-s1478

RA-011-D1RA00914A-s1479

RA-011-D1RA00914A-s1480

RA-011-D1RA00914A-s1481

RA-011-D1RA00914A-s1482

RA-011-D1RA00914A-s1483

RA-011-D1RA00914A-s1484

RA-011-D1RA00914A-s1485

RA-011-D1RA00914A-s1486

RA-011-D1RA00914A-s1487

RA-011-D1RA00914A-s1488

RA-011-D1RA00914A-s1489

RA-011-D1RA00914A-s1490

RA-011-D1RA00914A-s1491

RA-011-D1RA00914A-s1492

RA-011-D1RA00914A-s1493

RA-011-D1RA00914A-s1494

RA-011-D1RA00914A-s1495

RA-011-D1RA00914A-s1496

RA-011-D1RA00914A-s1497

RA-011-D1RA00914A-s1498

RA-011-D1RA00914A-s1499

RA-011-D1RA00914A-s1500

RA-011-D1RA00914A-s1501

RA-011-D1RA00914A-s1502

RA-011-D1RA00914A-s1503

RA-011-D1RA00914A-s1504

RA-011-D1RA00914A-s1505

RA-011-D1RA00914A-s1506

RA-011-D1RA00914A-s1507

RA-011-D1RA00914A-s1508

RA-011-D1RA00914A-s1509

RA-011-D1RA00914A-s1510

RA-011-D1RA00914A-s1511

RA-011-D1RA00914A-s1512

RA-011-D1RA00914A-s1513

RA-011-D1RA00914A-s1514

RA-011-D1RA00914A-s1515

RA-011-D1RA00914A-s1516

RA-011-D1RA00914A-s1517

RA-011-D1RA00914A-s1518

RA-011-D1RA00914A-s1519

RA-011-D1RA00914A-s1520

RA-011-D1RA00914A-s1521

RA-011-D1RA00914A-s1522

RA-011-D1RA00914A-s1523

RA-011-D1RA00914A-s1524

RA-011-D1RA00914A-s1525

RA-011-D1RA00914A-s1526

RA-011-D1RA00914A-s1527

RA-011-D1RA00914A-s1528

RA-011-D1RA00914A-s1529

RA-011-D1RA00914A-s1530

RA-011-D1RA00914A-s1531

RA-011-D1RA00914A-s1532

RA-011-D1RA00914A-s1533

RA-011-D1RA00914A-s1534

RA-011-D1RA00914A-s1535

RA-011-D1RA00914A-s1536

RA-011-D1RA00914A-s1537

RA-011-D1RA00914A-s1538

RA-011-D1RA00914A-s1539

RA-011-D1RA00914A-s1540

RA-011-D1RA00914A-s1541

RA-011-D1RA00914A-s1542

RA-011-D1RA00914A-s1543

RA-011-D1RA00914A-s1544

RA-011-D1RA00914A-s1545

RA-011-D1RA00914A-s1546

RA-011-D1RA00914A-s1547

RA-011-D1RA00914A-s1548

RA-011-D1RA00914A-s1549

RA-011-D1RA00914A-s1550

RA-011-D1RA00914A-s1551

RA-011-D1RA00914A-s1552

RA-011-D1RA00914A-s1553

RA-011-D1RA00914A-s1554

RA-011-D1RA00914A-s1555

RA-011-D1RA00914A-s1556

RA-011-D1RA00914A-s1557

RA-011-D1RA00914A-s1558

RA-011-D1RA00914A-s1559

RA-011-D1RA00914A-s1560

RA-011-D1RA00914A-s1561

RA-011-D1RA00914A-s1562

RA-011-D1RA00914A-s1563

RA-011-D1RA00914A-s1564

RA-011-D1RA00914A-s1565

RA-011-D1RA00914A-s1566

RA-011-D1RA00914A-s1567

RA-011-D1RA00914A-s1568

RA-011-D1RA00914A-s1569

RA-011-D1RA00914A-s1570

RA-011-D1RA00914A-s1571

RA-011-D1RA00914A-s1572

RA-011-D1RA00914A-s1573

RA-011-D1RA00914A-s1574

RA-011-D1RA00914A-s1575

RA-011-D1RA00914A-s1576

RA-011-D1RA00914A-s1577

RA-011-D1RA00914A-s1578

RA-011-D1RA00914A-s1579

RA-011-D1RA00914A-s1580

RA-011-D1RA00914A-s1581

RA-011-D1RA00914A-s1582

RA-011-D1RA00914A-s1583

RA-011-D1RA00914A-s1584

RA-011-D1RA00914A-s1585

RA-011-D1RA00914A-s1586

RA-011-D1RA00914A-s1587

RA-011-D1RA00914A-s1588

RA-011-D1RA00914A-s1589

RA-011-D1RA00914A-s1590

RA-011-D1RA00914A-s1591

RA-011-D1RA00914A-s1592

RA-011-D1RA00914A-s1593

RA-011-D1RA00914A-s1594

RA-011-D1RA00914A-s1595

RA-011-D1RA00914A-s1596

RA-011-D1RA00914A-s1597

RA-011-D1RA00914A-s1598

RA-011-D1RA00914A-s1599

RA-011-D1RA00914A-s1600

RA-011-D1RA00914A-s1601

RA-011-D1RA00914A-s1602

RA-011-D1RA00914A-s1603

RA-011-D1RA00914A-s1604

RA-011-D1RA00914A-s1605

RA-011-D1RA00914A-s1606

RA-011-D1RA00914A-s1607

RA-011-D1RA00914A-s1608

RA-011-D1RA00914A-s1609

RA-011-D1RA00914A-s1610

RA-011-D1RA00914A-s1611

RA-011-D1RA00914A-s1612

RA-011-D1RA00914A-s1613

RA-011-D1RA00914A-s1614

RA-011-D1RA00914A-s1615

RA-011-D1RA00914A-s1616

RA-011-D1RA00914A-s1617

RA-011-D1RA00914A-s1618

RA-011-D1RA00914A-s1619

RA-011-D1RA00914A-s1620

RA-011-D1RA00914A-s1621

RA-011-D1RA00914A-s1622

RA-011-D1RA00914A-s1623

RA-011-D1RA00914A-s1624

RA-011-D1RA00914A-s1625

RA-011-D1RA00914A-s1626

RA-011-D1RA00914A-s1627

RA-011-D1RA00914A-s1628

RA-011-D1RA00914A-s1629

RA-011-D1RA00914A-s1630

RA-011-D1RA00914A-s1631

RA-011-D1RA00914A-s1632

RA-011-D1RA00914A-s1633

RA-011-D1RA00914A-s1634

RA-011-D1RA00914A-s1635

RA-011-D1RA00914A-s1636

RA-011-D1RA00914A-s1637

RA-011-D1RA00914A-s1638

RA-011-D1RA00914A-s1639

RA-011-D1RA00914A-s1640

RA-011-D1RA00914A-s1641

RA-011-D1RA00914A-s1642

RA-011-D1RA00914A-s1643

RA-011-D1RA00914A-s1644

RA-011-D1RA00914A-s1645

RA-011-D1RA00914A-s1646

RA-011-D1RA00914A-s1647

RA-011-D1RA00914A-s1648

RA-011-D1RA00914A-s1649

RA-011-D1RA00914A-s1650

RA-011-D1RA00914A-s1651

RA-011-D1RA00914A-s1652

RA-011-D1RA00914A-s1653

RA-011-D1RA00914A-s1654

RA-011-D1RA00914A-s1655

RA-011-D1RA00914A-s1656

RA-011-D1RA00914A-s1657

RA-011-D1RA00914A-s1658

RA-011-D1RA00914A-s1659

RA-011-D1RA00914A-s1660

RA-011-D1RA00914A-s1661

RA-011-D1RA00914A-s1662

RA-011-D1RA00914A-s1663

RA-011-D1RA00914A-s1664

RA-011-D1RA00914A-s1665

RA-011-D1RA00914A-s1666

RA-011-D1RA00914A-s1667

RA-011-D1RA00914A-s1668

RA-011-D1RA00914A-s1669

RA-011-D1RA00914A-s1670

RA-011-D1RA00914A-s1671

RA-011-D1RA00914A-s1672

RA-011-D1RA00914A-s1673

RA-011-D1RA00914A-s1674

RA-011-D1RA00914A-s1675

RA-011-D1RA00914A-s1676

RA-011-D1RA00914A-s1677

RA-011-D1RA00914A-s1678

RA-011-D1RA00914A-s1679

RA-011-D1RA00914A-s1680

RA-011-D1RA00914A-s1681

RA-011-D1RA00914A-s1682

RA-011-D1RA00914A-s1683

RA-011-D1RA00914A-s1684

RA-011-D1RA00914A-s1685

RA-011-D1RA00914A-s1686

RA-011-D1RA00914A-s1687

RA-011-D1RA00914A-s1688

RA-011-D1RA00914A-s1689

RA-011-D1RA00914A-s1690

RA-011-D1RA00914A-s1691

RA-011-D1RA00914A-s1692

RA-011-D1RA00914A-s1693

RA-011-D1RA00914A-s1694

RA-011-D1RA00914A-s1695

RA-011-D1RA00914A-s1696

RA-011-D1RA00914A-s1697

RA-011-D1RA00914A-s1698

RA-011-D1RA00914A-s1699

RA-011-D1RA00914A-s1700

RA-011-D1RA00914A-s1701

RA-011-D1RA00914A-s1702

RA-011-D1RA00914A-s1703

RA-011-D1RA00914A-s1704

RA-011-D1RA00914A-s1705

RA-011-D1RA00914A-s1706

RA-011-D1RA00914A-s1707

RA-011-D1RA00914A-s1708

RA-011-D1RA00914A-s1709

RA-011-D1RA00914A-s1710

RA-011-D1RA00914A-s1711

RA-011-D1RA00914A-s1712

RA-011-D1RA00914A-s1713

RA-011-D1RA00914A-s1714

RA-011-D1RA00914A-s1715

RA-011-D1RA00914A-s1716

RA-011-D1RA00914A-s1717

RA-011-D1RA00914A-s1718

RA-011-D1RA00914A-s1719

RA-011-D1RA00914A-s1720

RA-011-D1RA00914A-s1721

RA-011-D1RA00914A-s1722

RA-011-D1RA00914A-s1723

RA-011-D1RA00914A-s1724

RA-011-D1RA00914A-s1725

RA-011-D1RA00914A-s1726

RA-011-D1RA00914A-s1727

RA-011-D1RA00914A-s1728

RA-011-D1RA00914A-s1729

RA-011-D1RA00914A-s1730

RA-011-D1RA00914A-s1731

RA-011-D1RA00914A-s1732

RA-011-D1RA00914A-s1733

RA-011-D1RA00914A-s1734

RA-011-D1RA00914A-s1735

RA-011-D1RA00914A-s1736

RA-011-D1RA00914A-s1737

RA-011-D1RA00914A-s1738

RA-011-D1RA00914A-s1739

RA-011-D1RA00914A-s1740

RA-011-D1RA00914A-s1741

RA-011-D1RA00914A-s1742

RA-011-D1RA00914A-s1743

RA-011-D1RA00914A-s1744

RA-011-D1RA00914A-s1745

RA-011-D1RA00914A-s1746

RA-011-D1RA00914A-s1747

RA-011-D1RA00914A-s1748

RA-011-D1RA00914A-s1749

RA-011-D1RA00914A-s1750

RA-011-D1RA00914A-s1751

RA-011-D1RA00914A-s1752

RA-011-D1RA00914A-s1753

RA-011-D1RA00914A-s1754

RA-011-D1RA00914A-s1755

RA-011-D1RA00914A-s1756

RA-011-D1RA00914A-s1757

RA-011-D1RA00914A-s1758

RA-011-D1RA00914A-s1759

RA-011-D1RA00914A-s1760

RA-011-D1RA00914A-s1761

RA-011-D1RA00914A-s1762

RA-011-D1RA00914A-s1763

RA-011-D1RA00914A-s1764

RA-011-D1RA00914A-s1765

RA-011-D1RA00914A-s1766

RA-011-D1RA00914A-s1767
