# Supplementary material for: Improved herbicide discovery using physico-chemical rules refined by antimalarial library screening
Source: RSC Adv. 2021 Feb 23;11(15):8459–67. doi: 10.1039/d1ra00914a (PMC8695207; doi:10.1039/d1ra00914a)
Supplement: RA-011-D1RA00914A-s001 [file RA-011-D1RA00914A-s001.pdf]

Electronic Supplementary Material (ESI) for RSC Advances.
This journal is © The Royal Society of Chemistry 2021

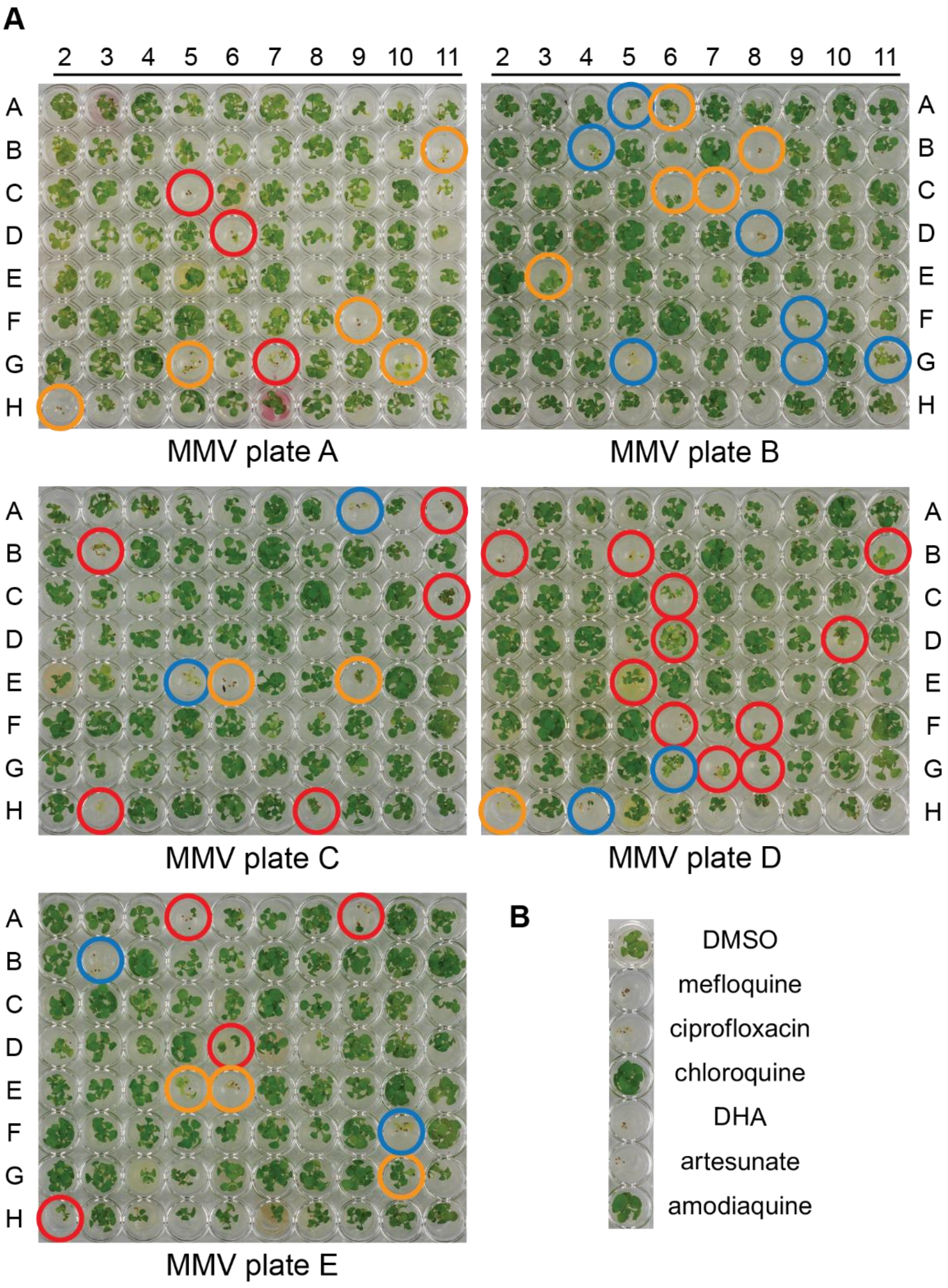

Supporting Figure 1 | Agar plate herbicidal activity of antimalarial compounds. (A) Activity of the MMV400 against *A. thaliana* at 80 µM. Orange circles indicate the compounds active against soil-grown plants, red circles indicate plate-only active compounds, blue circles denote plate-active compounds that were not tested for activity against soil-grown plants; (B) Positive controls at 80 µM, DHA – dihydroartemisinin, DMSO was used as a negative control at 1% (vol) concentration.
