# Supplementary figures and images for: Improved herbicide discovery using physico-chemical rules refined by antimalarial library screening (part 1 of 14)
Source: RSC Adv. 2021 Feb 23;11(15):8459–67. doi: 10.1039/d1ra00914a (PMC8695207; doi:10.1039/d1ra00914a)

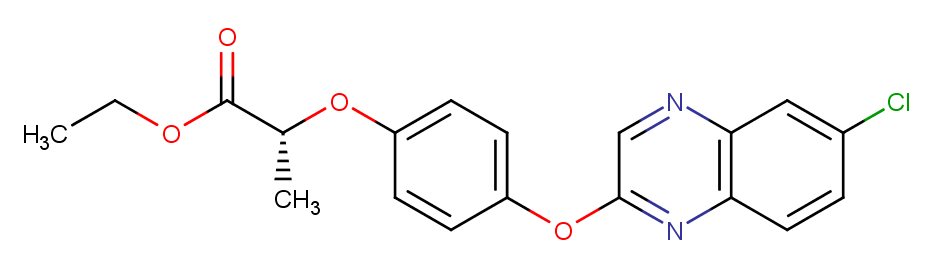

Supplement: RA-011-D1RA00914A-s003 [file RA-011-D1RA00914A-s003.png]

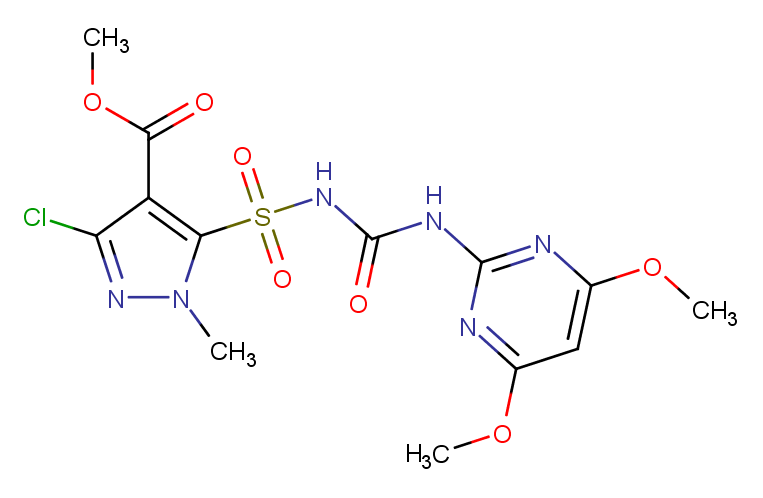

Supplement: RA-011-D1RA00914A-s004 [file RA-011-D1RA00914A-s004.png]

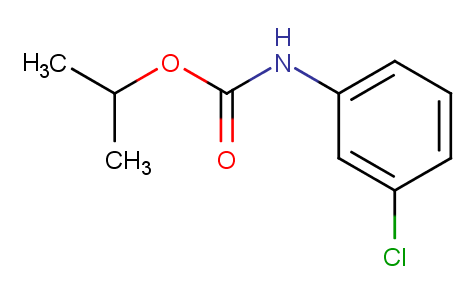

Supplement: RA-011-D1RA00914A-s005 [file RA-011-D1RA00914A-s005.png]

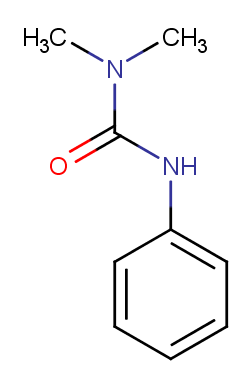

Supplement: RA-011-D1RA00914A-s006 [file RA-011-D1RA00914A-s006.png]

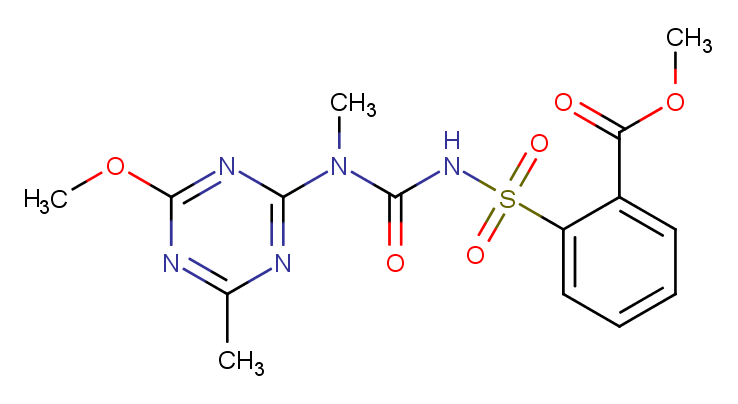

Supplement: RA-011-D1RA00914A-s007 [file RA-011-D1RA00914A-s007.png]

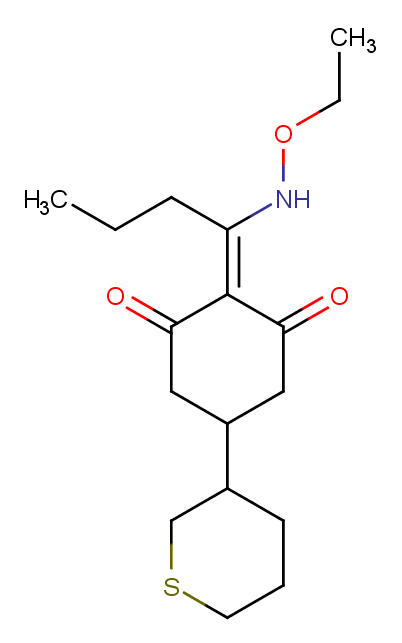

Supplement: RA-011-D1RA00914A-s008 [file RA-011-D1RA00914A-s008.png]

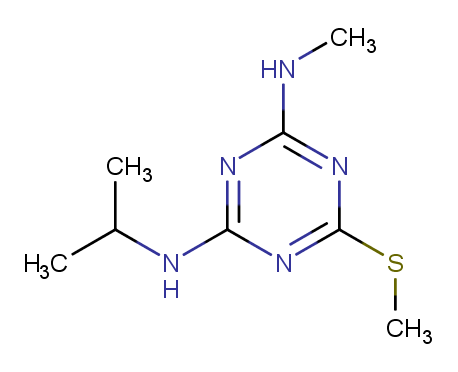

Supplement: RA-011-D1RA00914A-s009 [file RA-011-D1RA00914A-s009.png]

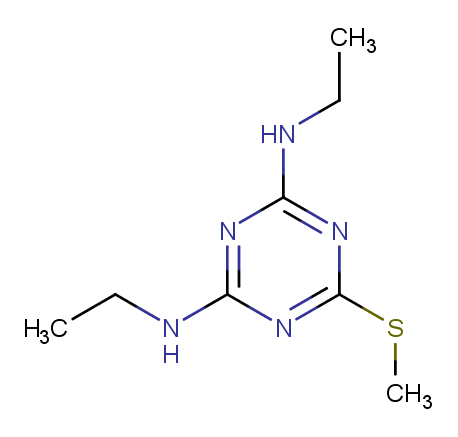

Supplement: RA-011-D1RA00914A-s010 [file RA-011-D1RA00914A-s010.png]

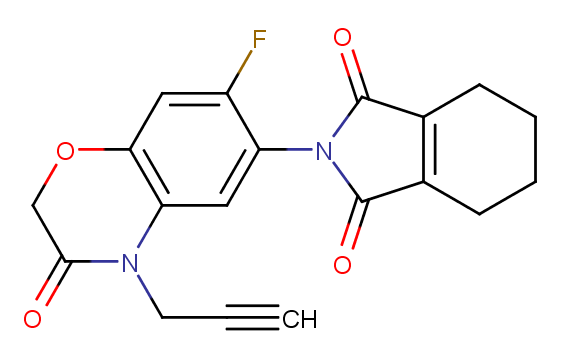

Supplement: RA-011-D1RA00914A-s011 [file RA-011-D1RA00914A-s011.png]

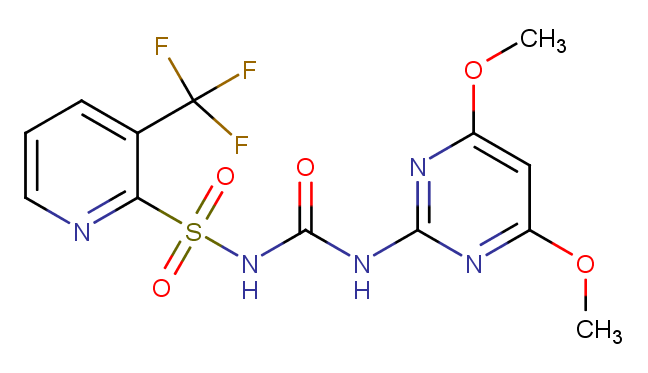

Supplement: RA-011-D1RA00914A-s012 [file RA-011-D1RA00914A-s012.png]

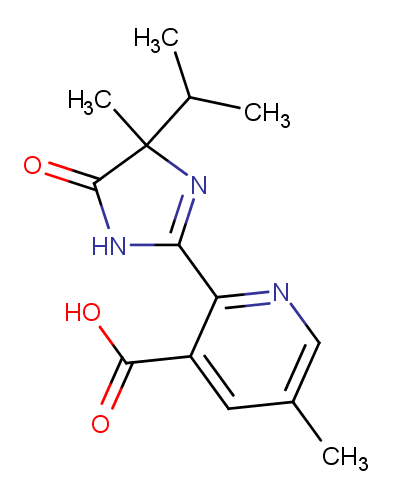

Supplement: RA-011-D1RA00914A-s013 [file RA-011-D1RA00914A-s013.png]

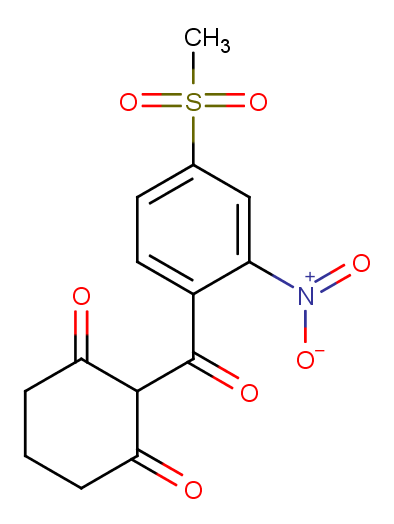

Supplement: RA-011-D1RA00914A-s014 [file RA-011-D1RA00914A-s014.png]

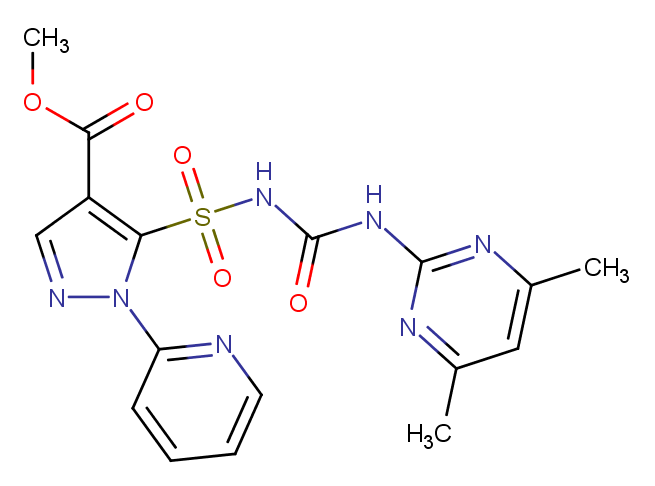

Supplement: RA-011-D1RA00914A-s015 [file RA-011-D1RA00914A-s015.png]

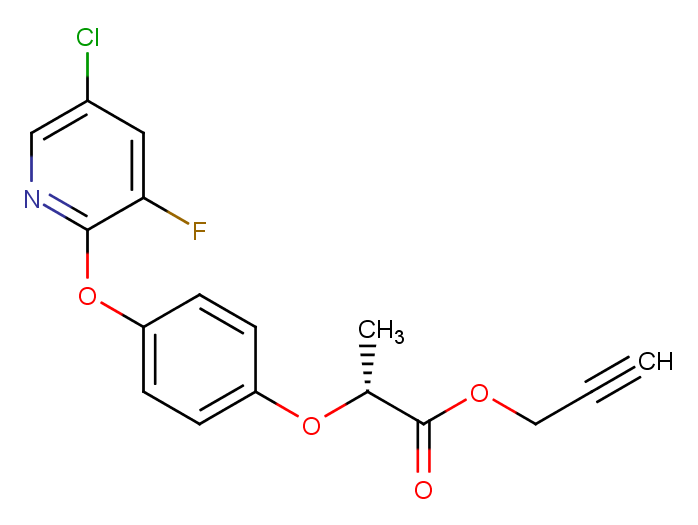

Supplement: RA-011-D1RA00914A-s016 [file RA-011-D1RA00914A-s016.png]

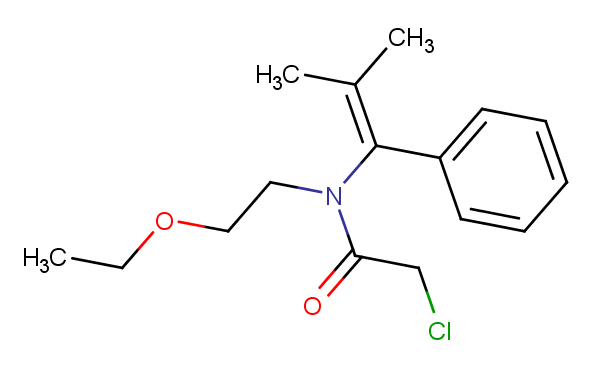

Supplement: RA-011-D1RA00914A-s017 [file RA-011-D1RA00914A-s017.png]

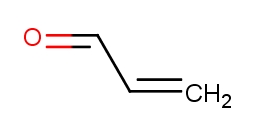

Supplement: RA-011-D1RA00914A-s018 [file RA-011-D1RA00914A-s018.png]

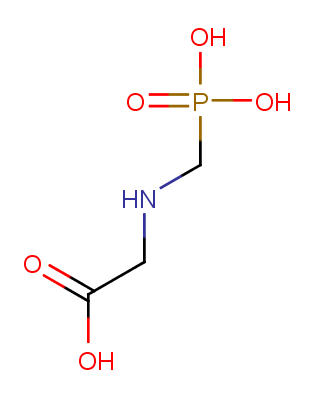

Supplement: RA-011-D1RA00914A-s019 [file RA-011-D1RA00914A-s019.png]

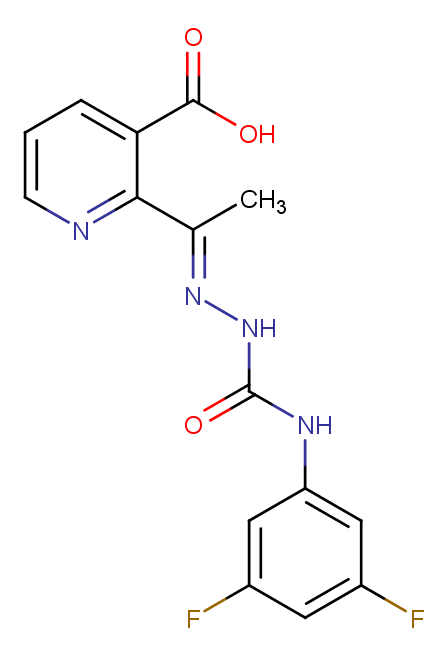

Supplement: RA-011-D1RA00914A-s020 [file RA-011-D1RA00914A-s020.png]

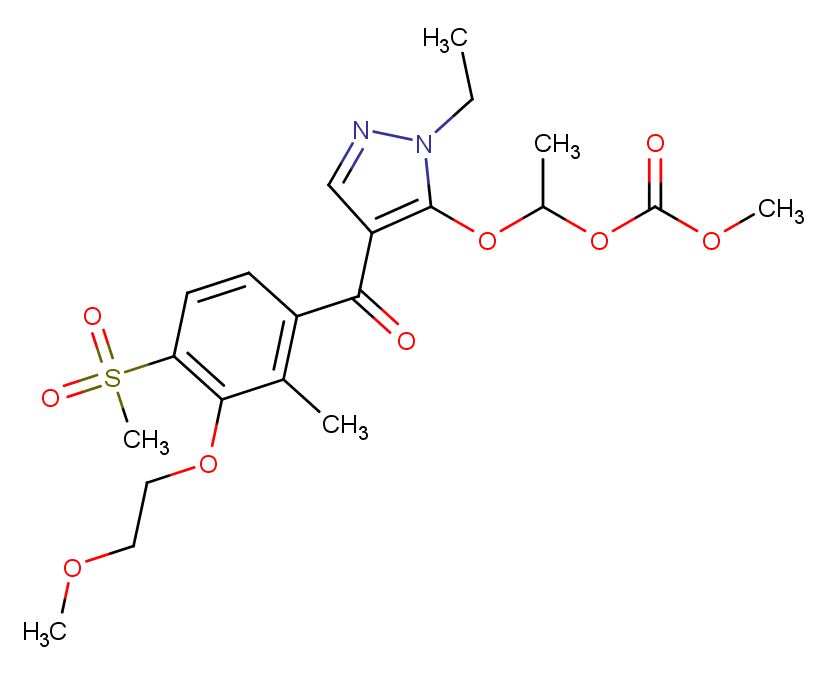

Supplement: RA-011-D1RA00914A-s021 [file RA-011-D1RA00914A-s021.png]

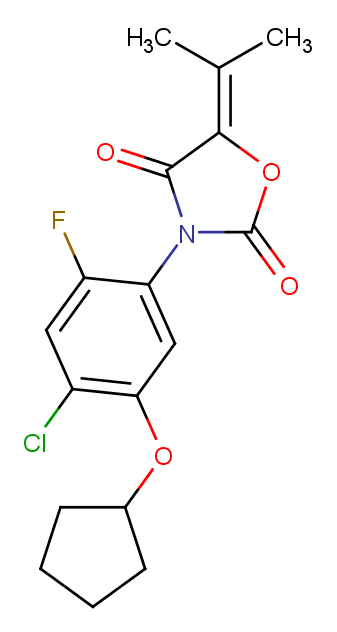

Supplement: RA-011-D1RA00914A-s022 [file RA-011-D1RA00914A-s022.png]

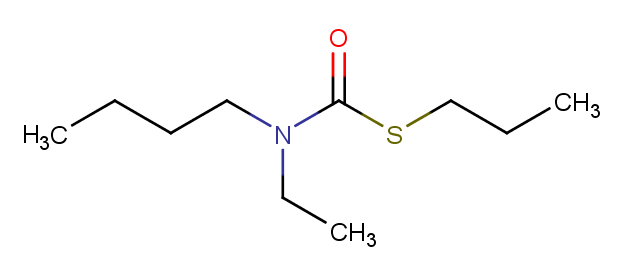

Supplement: RA-011-D1RA00914A-s023 [file RA-011-D1RA00914A-s023.png]

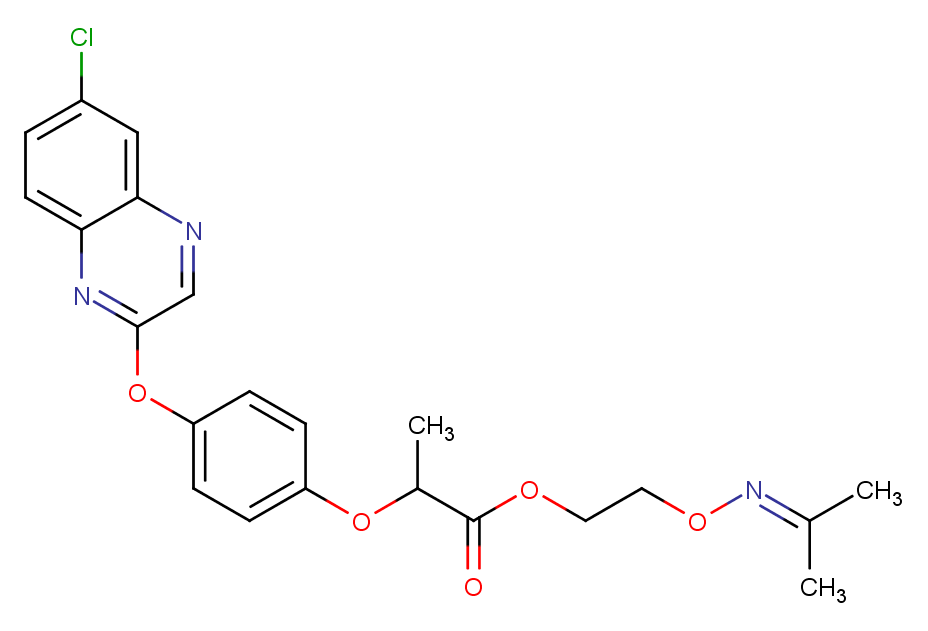

Supplement: RA-011-D1RA00914A-s024 [file RA-011-D1RA00914A-s024.png]

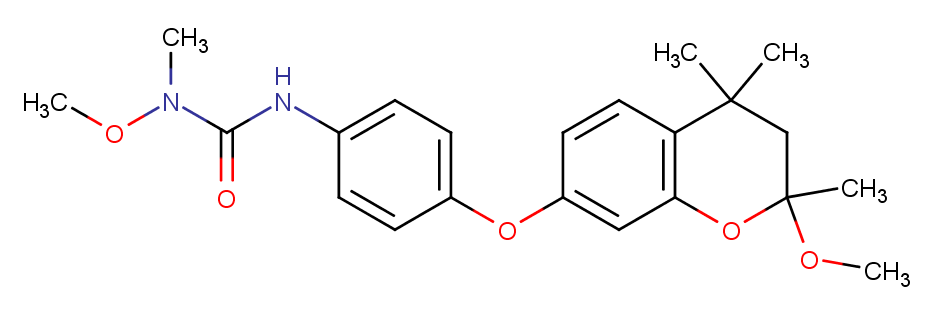

Supplement: RA-011-D1RA00914A-s025 [file RA-011-D1RA00914A-s025.png]

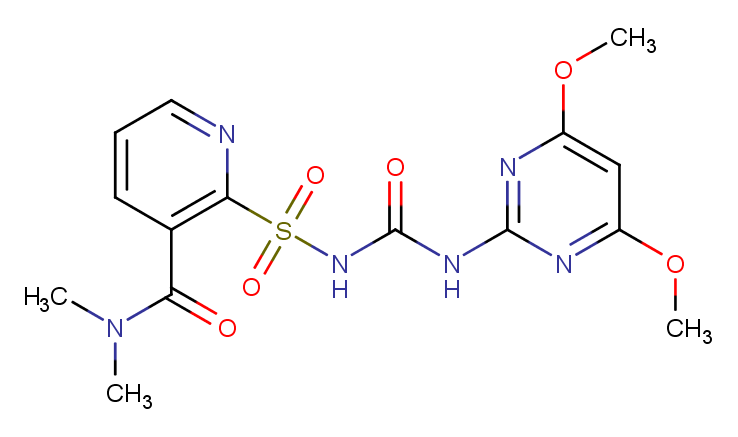

Supplement: RA-011-D1RA00914A-s026 [file RA-011-D1RA00914A-s026.png]

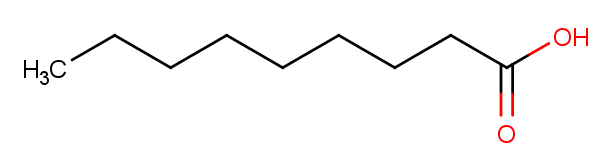

Supplement: RA-011-D1RA00914A-s027 [file RA-011-D1RA00914A-s027.png]

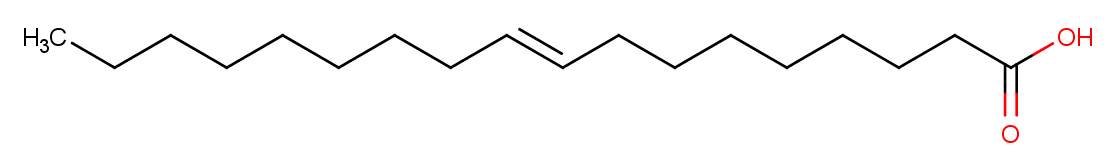

Supplement: RA-011-D1RA00914A-s028 [file RA-011-D1RA00914A-s028.png]

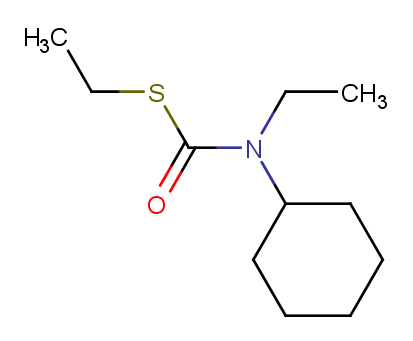

Supplement: RA-011-D1RA00914A-s029 [file RA-011-D1RA00914A-s029.png]

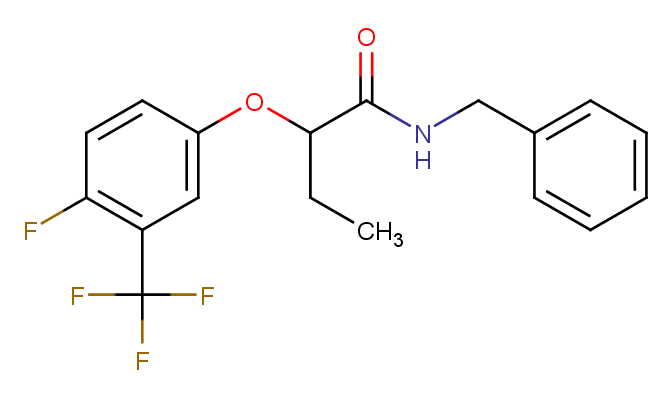

Supplement: RA-011-D1RA00914A-s030 [file RA-011-D1RA00914A-s030.png]

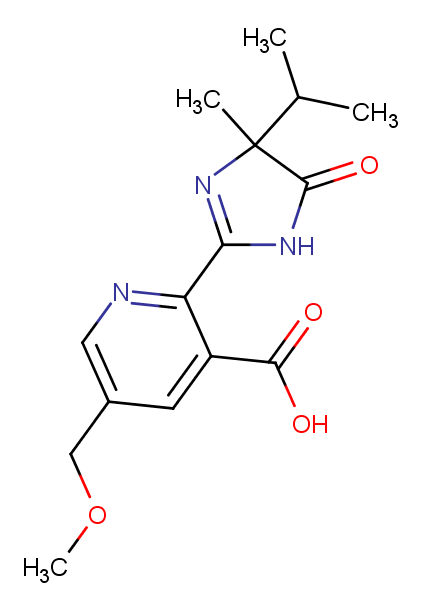

Supplement: RA-011-D1RA00914A-s031 [file RA-011-D1RA00914A-s031.png]

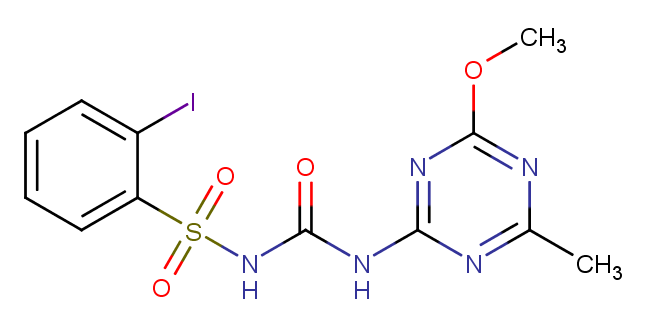

Supplement: RA-011-D1RA00914A-s032 [file RA-011-D1RA00914A-s032.png]

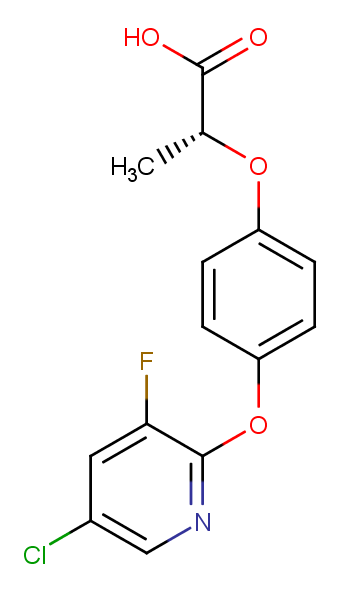

Supplement: RA-011-D1RA00914A-s033 [file RA-011-D1RA00914A-s033.png]

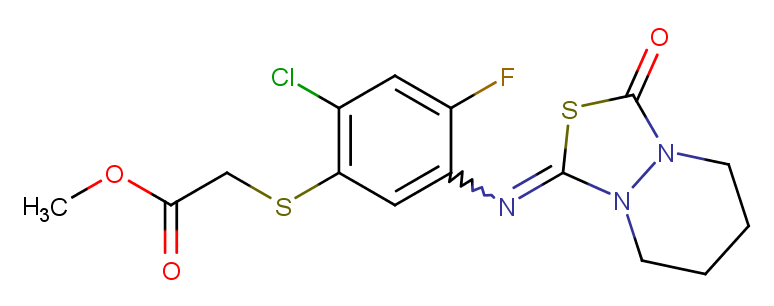

Supplement: RA-011-D1RA00914A-s034 [file RA-011-D1RA00914A-s034.png]

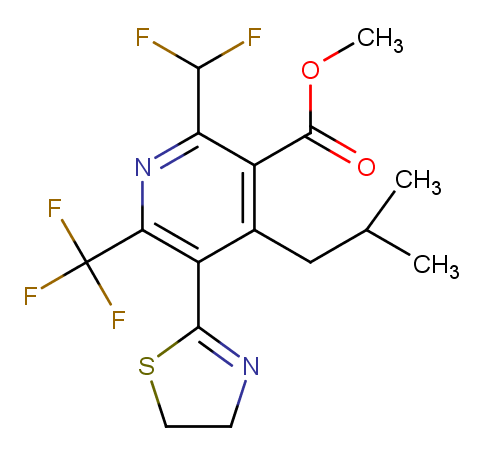

Supplement: RA-011-D1RA00914A-s035 [file RA-011-D1RA00914A-s035.png]

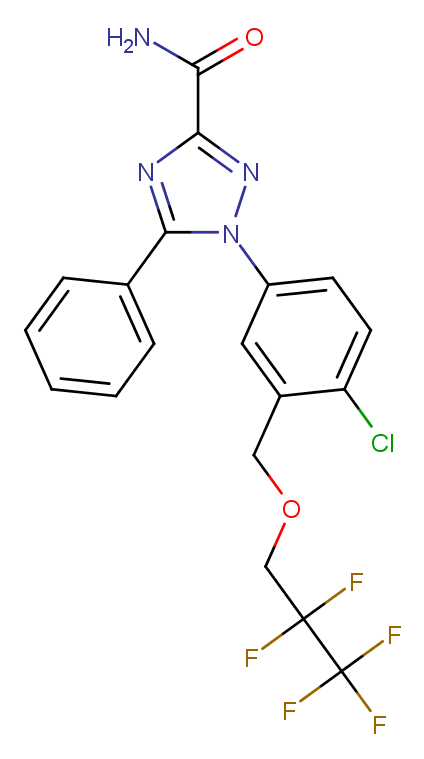

Supplement: RA-011-D1RA00914A-s036 [file RA-011-D1RA00914A-s036.png]

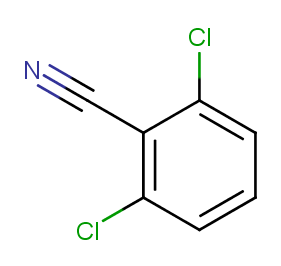

Supplement: RA-011-D1RA00914A-s037 [file RA-011-D1RA00914A-s037.png]

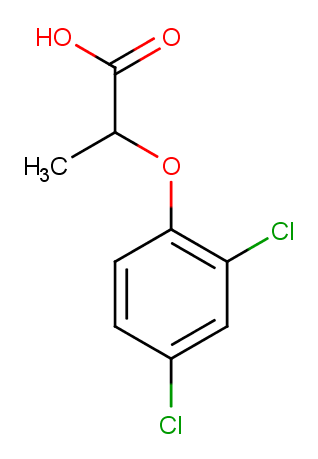

Supplement: RA-011-D1RA00914A-s038 [file RA-011-D1RA00914A-s038.png]

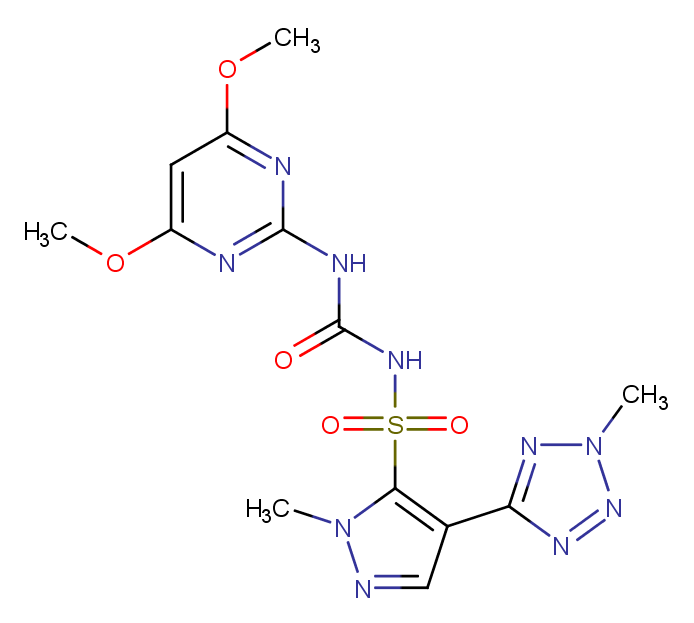

Supplement: RA-011-D1RA00914A-s039 [file RA-011-D1RA00914A-s039.png]

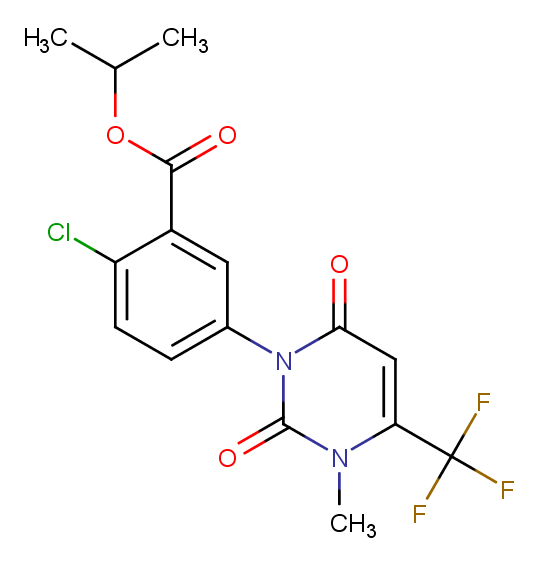

Supplement: RA-011-D1RA00914A-s040 [file RA-011-D1RA00914A-s040.png]

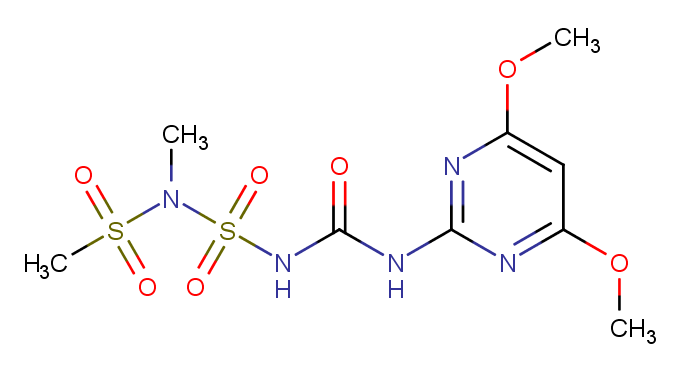

Supplement: RA-011-D1RA00914A-s041 [file RA-011-D1RA00914A-s041.png]

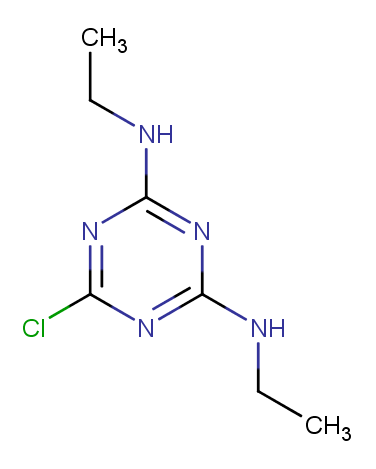

Supplement: RA-011-D1RA00914A-s042 [file RA-011-D1RA00914A-s042.png]

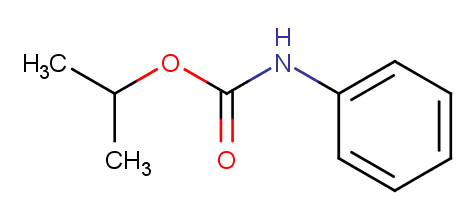

Supplement: RA-011-D1RA00914A-s043 [file RA-011-D1RA00914A-s043.png]

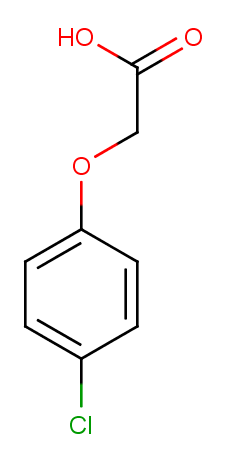

Supplement: RA-011-D1RA00914A-s044 [file RA-011-D1RA00914A-s044.png]

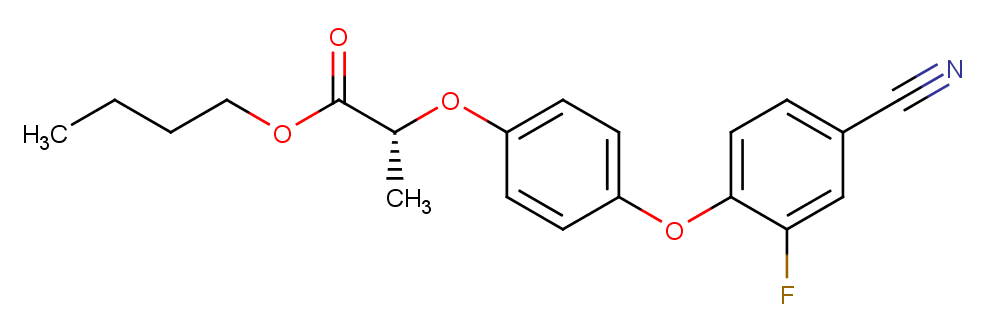

Supplement: RA-011-D1RA00914A-s045 [file RA-011-D1RA00914A-s045.png]

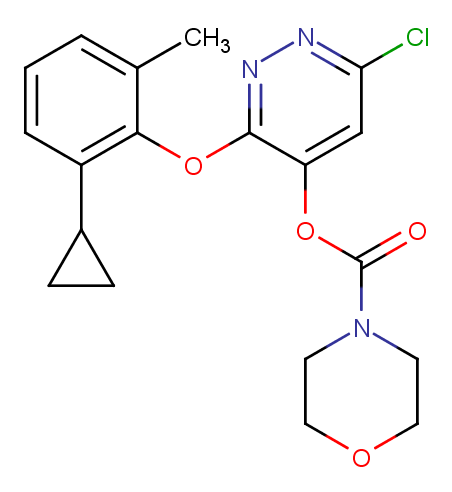

Supplement: RA-011-D1RA00914A-s046 [file RA-011-D1RA00914A-s046.png]

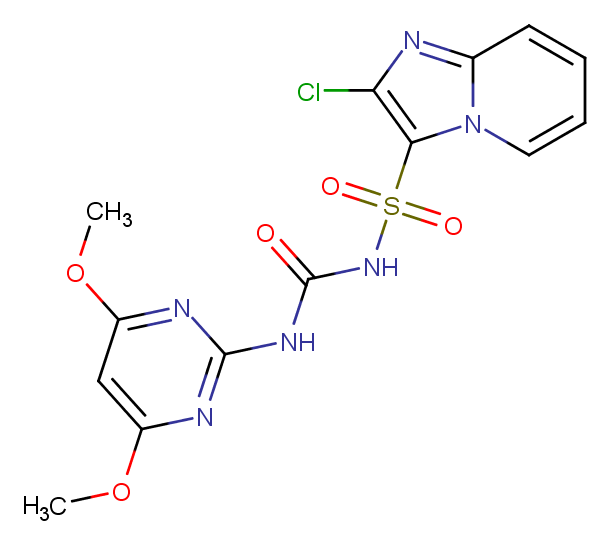

Supplement: RA-011-D1RA00914A-s047 [file RA-011-D1RA00914A-s047.png]

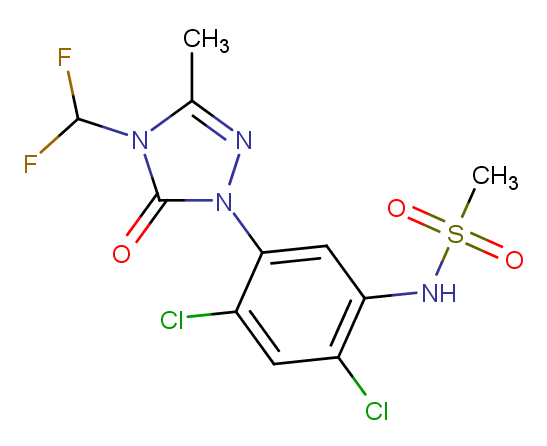

Supplement: RA-011-D1RA00914A-s048 [file RA-011-D1RA00914A-s048.png]

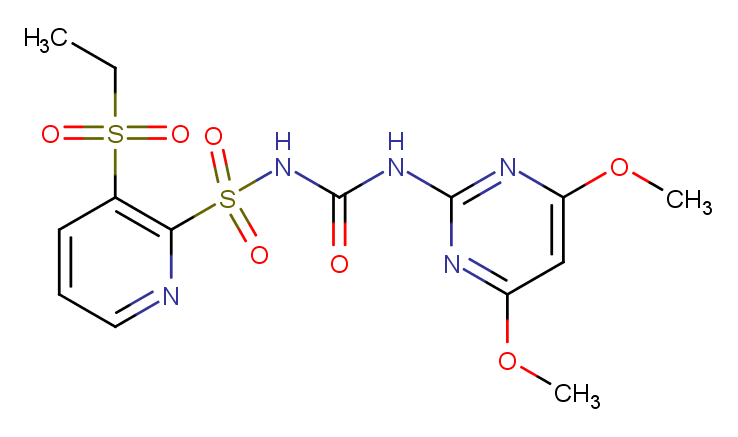

Supplement: RA-011-D1RA00914A-s049 [file RA-011-D1RA00914A-s049.png]

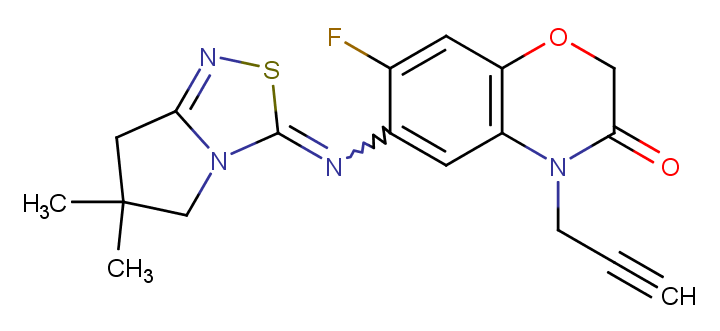

Supplement: RA-011-D1RA00914A-s050 [file RA-011-D1RA00914A-s050.png]

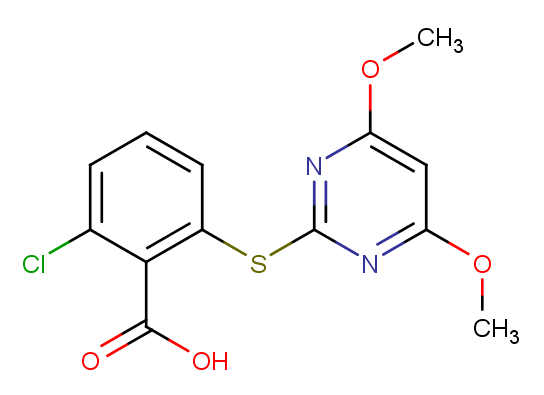

Supplement: RA-011-D1RA00914A-s051 [file RA-011-D1RA00914A-s051.png]

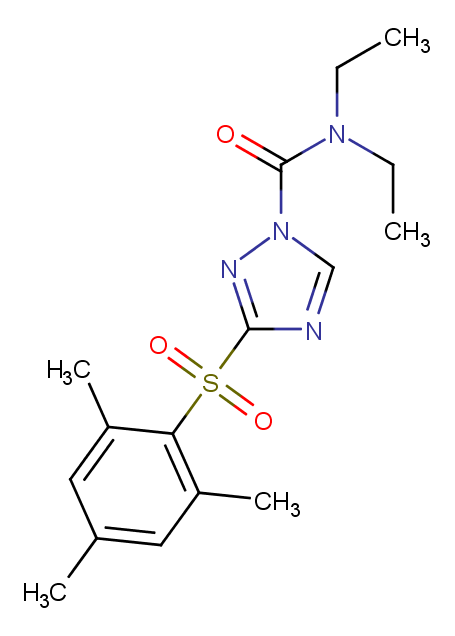

Supplement: RA-011-D1RA00914A-s052 [file RA-011-D1RA00914A-s052.png]

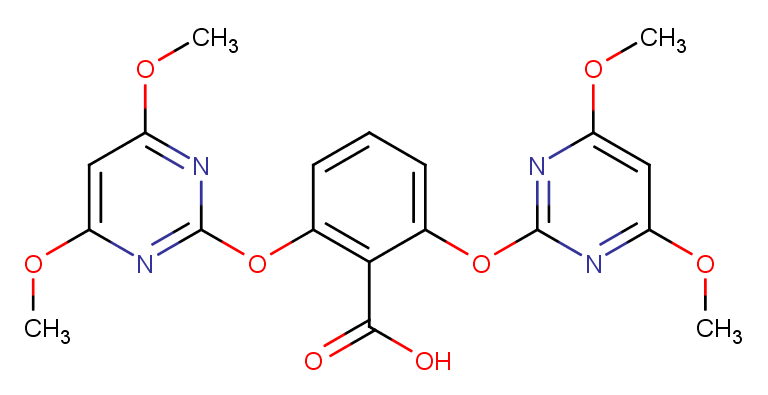

Supplement: RA-011-D1RA00914A-s053 [file RA-011-D1RA00914A-s053.png]

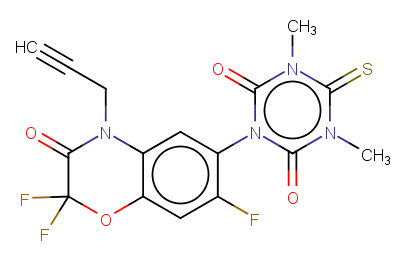

Supplement: RA-011-D1RA00914A-s054 [file RA-011-D1RA00914A-s054.png]

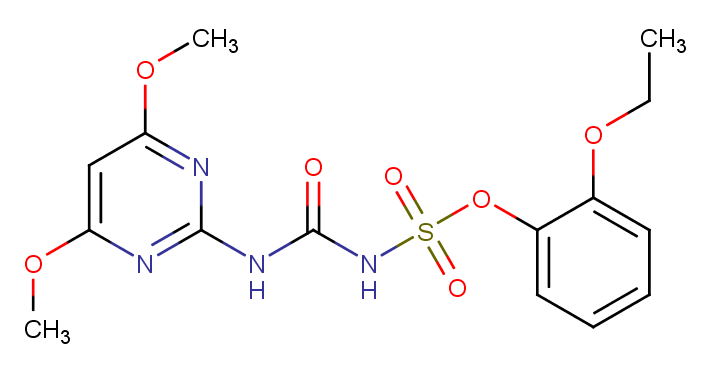

Supplement: RA-011-D1RA00914A-s055 [file RA-011-D1RA00914A-s055.png]

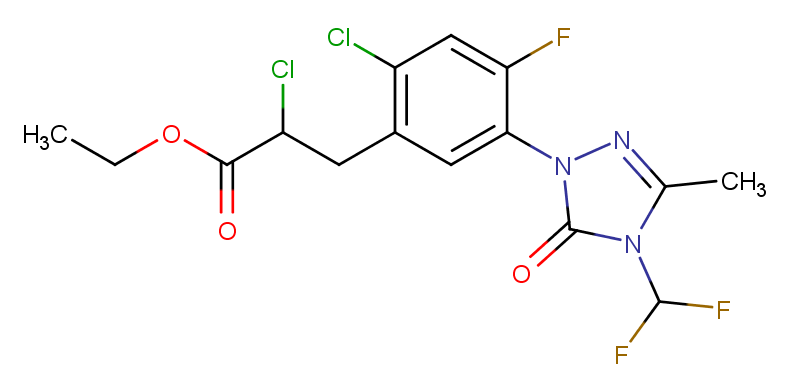

Supplement: RA-011-D1RA00914A-s056 [file RA-011-D1RA00914A-s056.png]

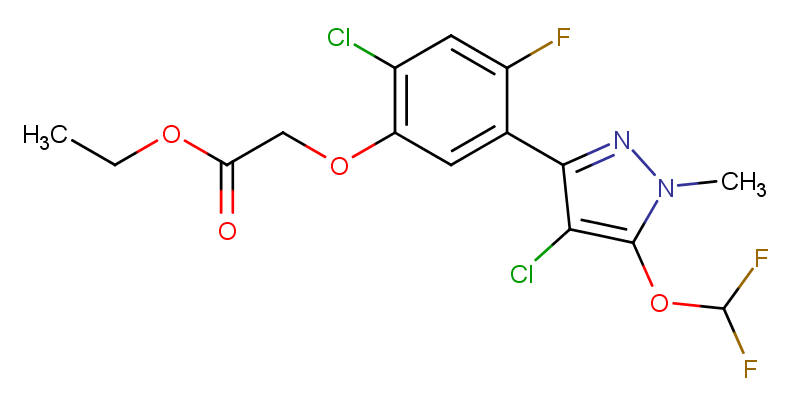

Supplement: RA-011-D1RA00914A-s057 [file RA-011-D1RA00914A-s057.png]

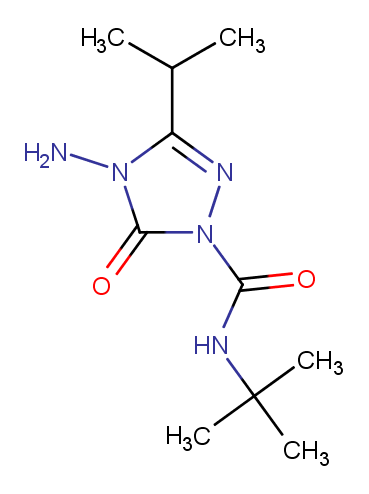

Supplement: RA-011-D1RA00914A-s058 [file RA-011-D1RA00914A-s058.png]

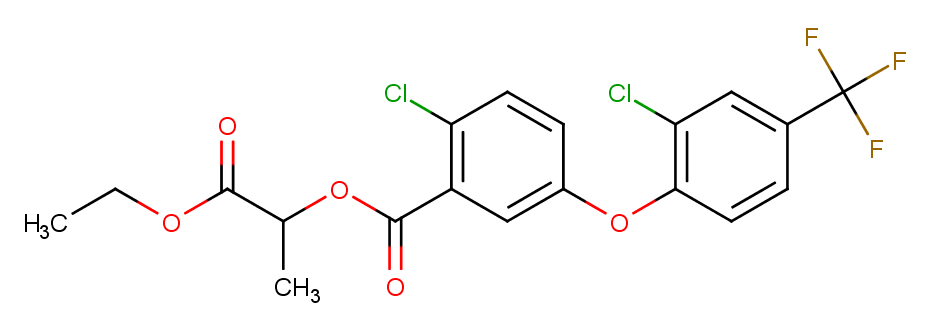

Supplement: RA-011-D1RA00914A-s059 [file RA-011-D1RA00914A-s059.png]

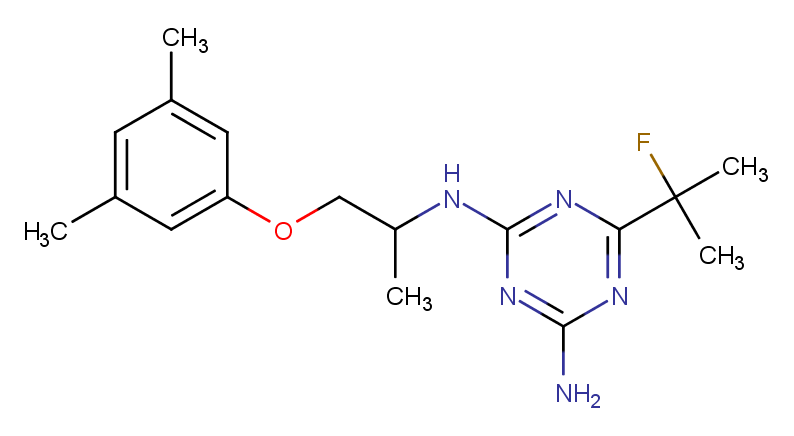

Supplement: RA-011-D1RA00914A-s060 [file RA-011-D1RA00914A-s060.png]

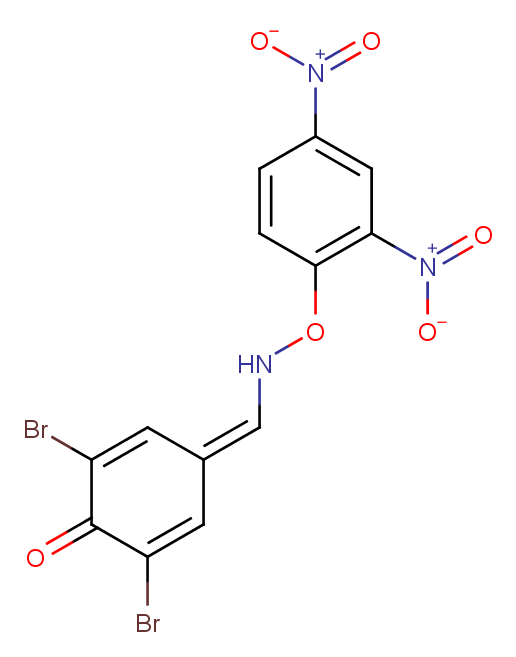

Supplement: RA-011-D1RA00914A-s061 [file RA-011-D1RA00914A-s061.png]

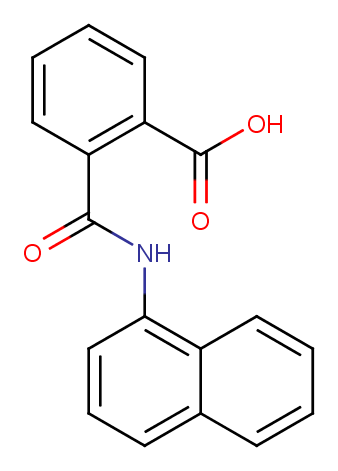

Supplement: RA-011-D1RA00914A-s062 [file RA-011-D1RA00914A-s062.png]

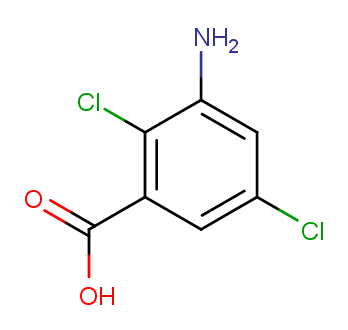

Supplement: RA-011-D1RA00914A-s063 [file RA-011-D1RA00914A-s063.png]

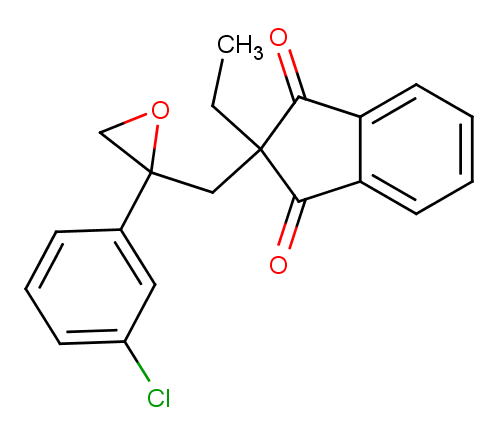

Supplement: RA-011-D1RA00914A-s064 [file RA-011-D1RA00914A-s064.png]

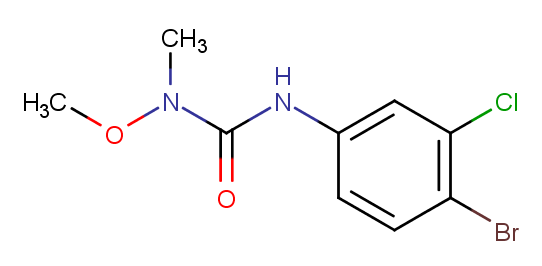

Supplement: RA-011-D1RA00914A-s065 [file RA-011-D1RA00914A-s065.png]

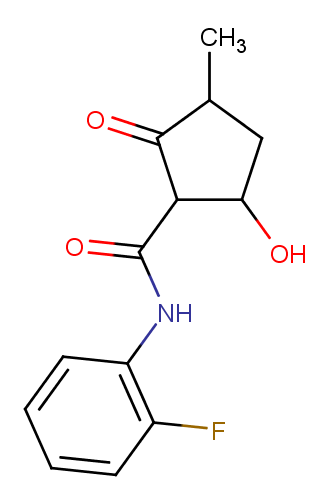

Supplement: RA-011-D1RA00914A-s066 [file RA-011-D1RA00914A-s066.png]

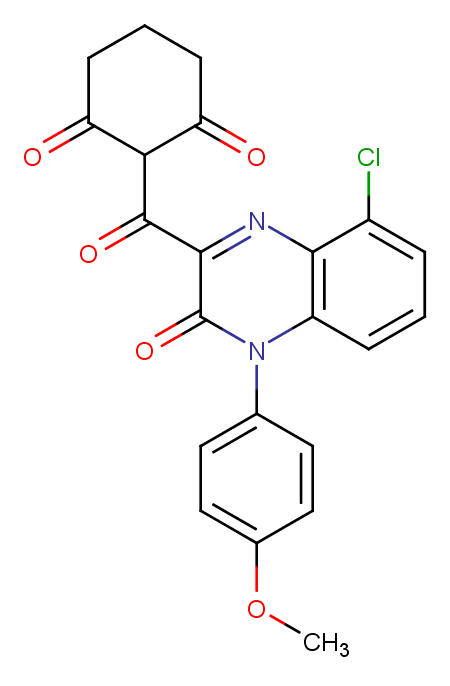

Supplement: RA-011-D1RA00914A-s067 [file RA-011-D1RA00914A-s067.png]

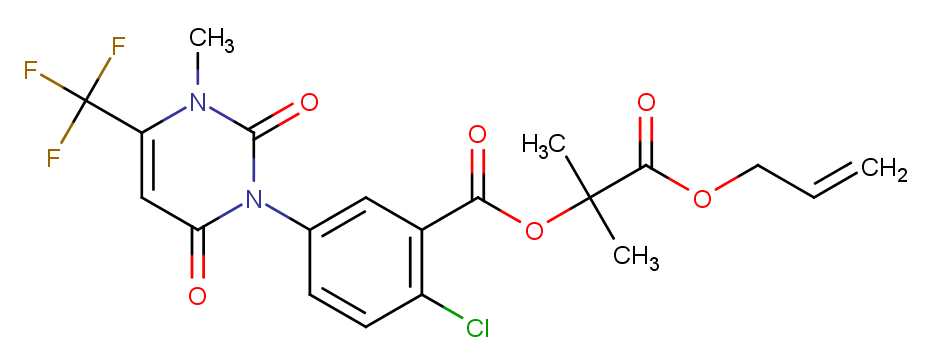

Supplement: RA-011-D1RA00914A-s068 [file RA-011-D1RA00914A-s068.png]

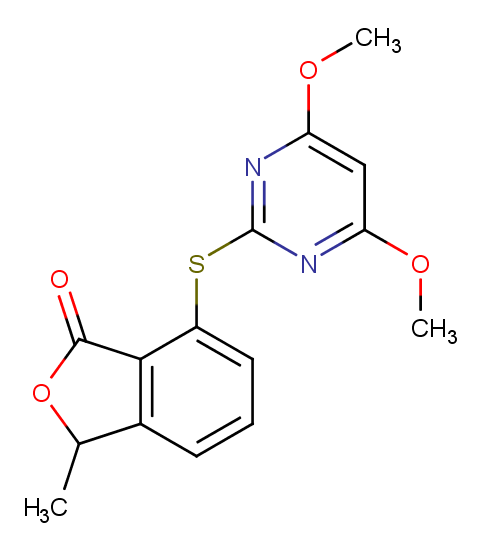

Supplement: RA-011-D1RA00914A-s069 [file RA-011-D1RA00914A-s069.png]

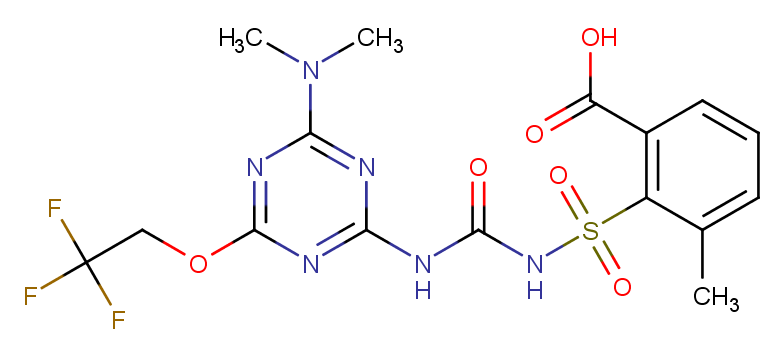

Supplement: RA-011-D1RA00914A-s070 [file RA-011-D1RA00914A-s070.png]

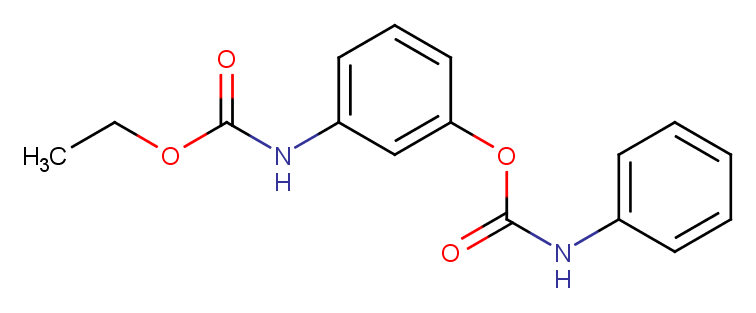

Supplement: RA-011-D1RA00914A-s071 [file RA-011-D1RA00914A-s071.png]

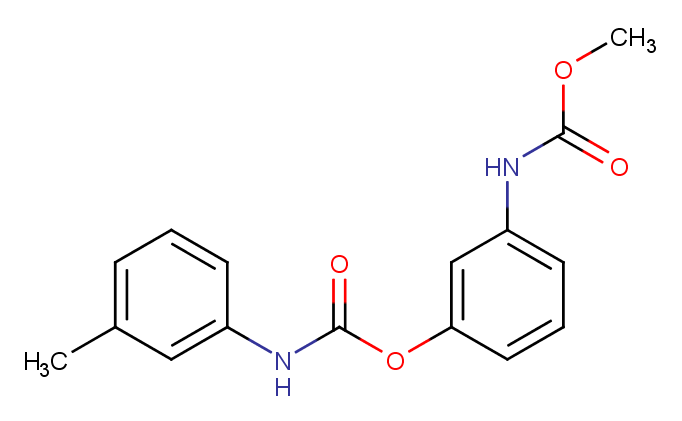

Supplement: RA-011-D1RA00914A-s072 [file RA-011-D1RA00914A-s072.png]

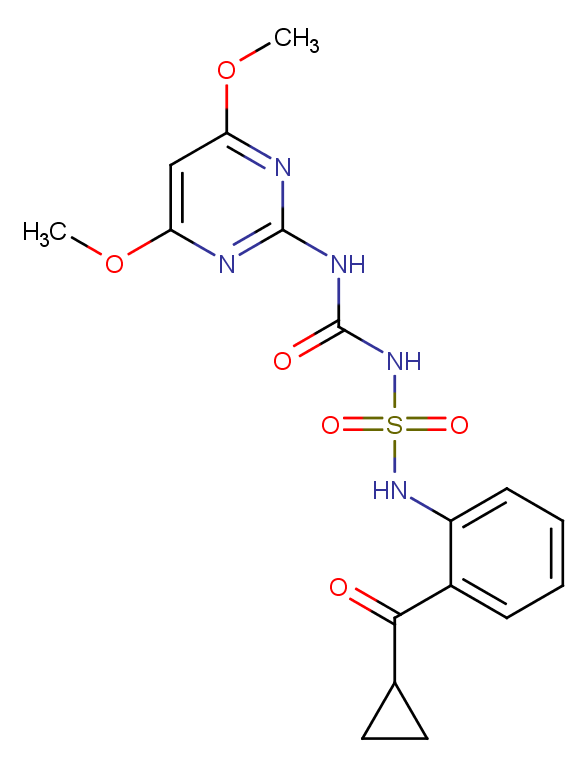

Supplement: RA-011-D1RA00914A-s073 [file RA-011-D1RA00914A-s073.png]

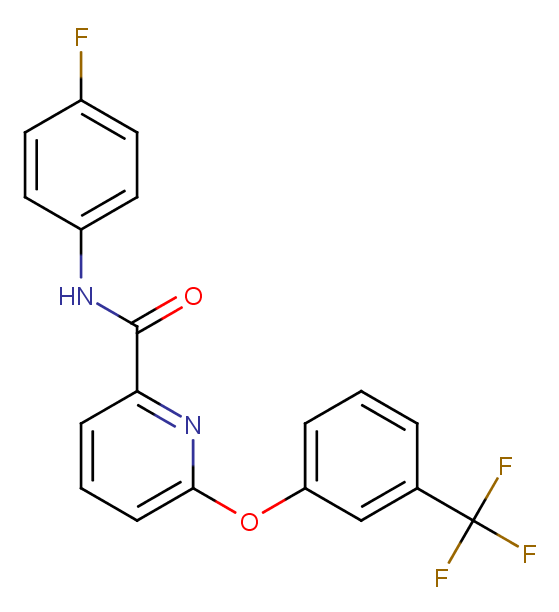

Supplement: RA-011-D1RA00914A-s074 [file RA-011-D1RA00914A-s074.png]

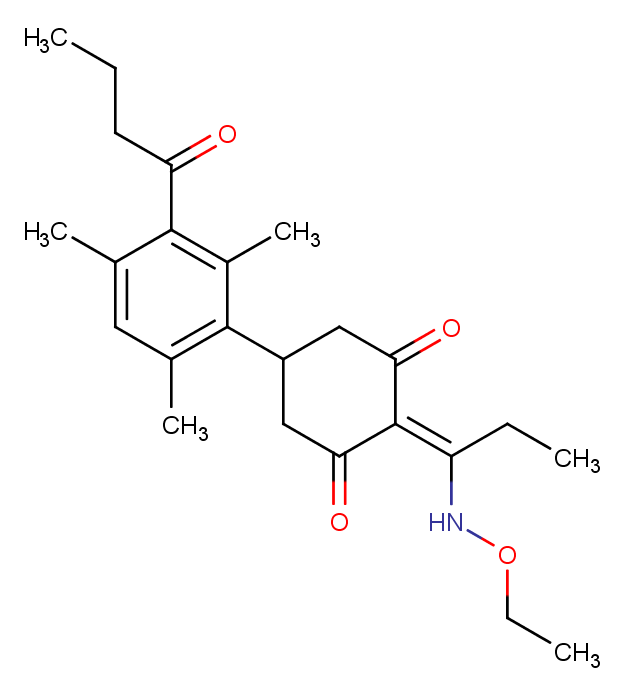

Supplement: RA-011-D1RA00914A-s075 [file RA-011-D1RA00914A-s075.png]

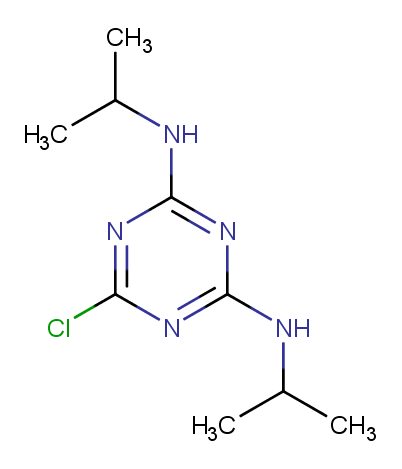

Supplement: RA-011-D1RA00914A-s076 [file RA-011-D1RA00914A-s076.png]

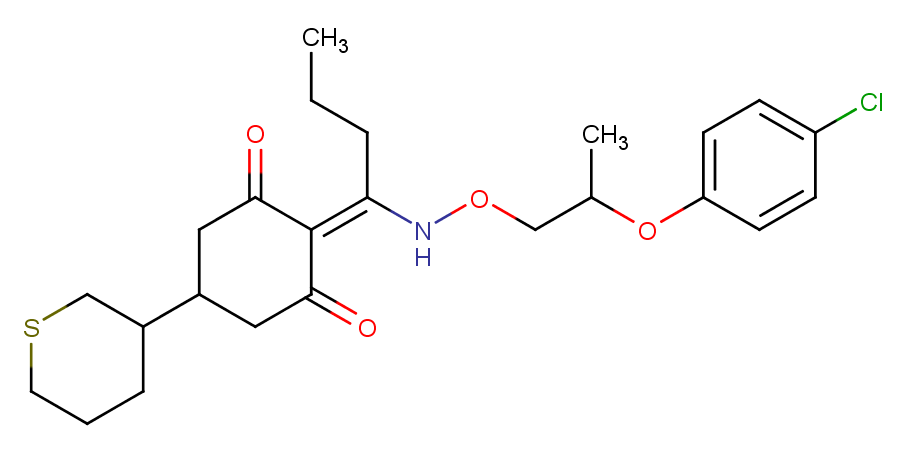

Supplement: RA-011-D1RA00914A-s077 [file RA-011-D1RA00914A-s077.png]

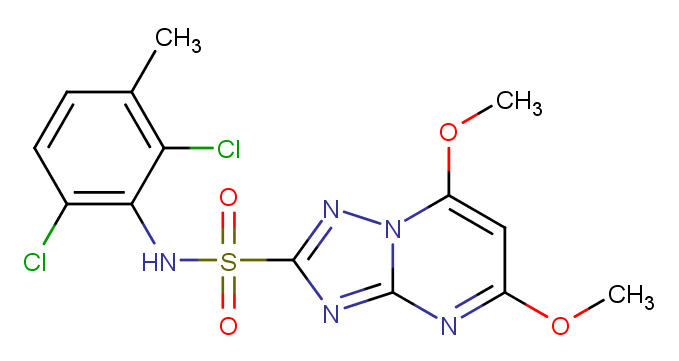

Supplement: RA-011-D1RA00914A-s078 [file RA-011-D1RA00914A-s078.png]

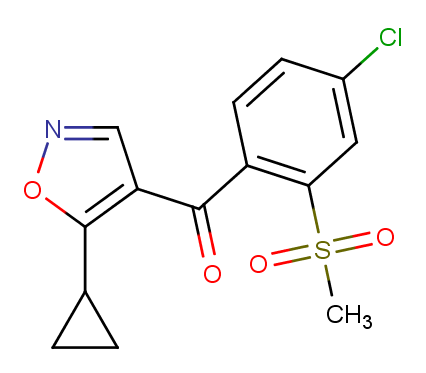

Supplement: RA-011-D1RA00914A-s079 [file RA-011-D1RA00914A-s079.png]

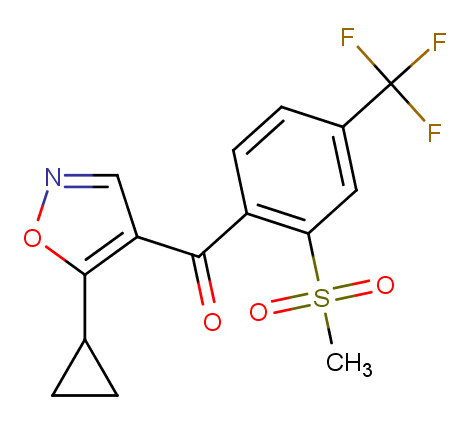

Supplement: RA-011-D1RA00914A-s080 [file RA-011-D1RA00914A-s080.png]

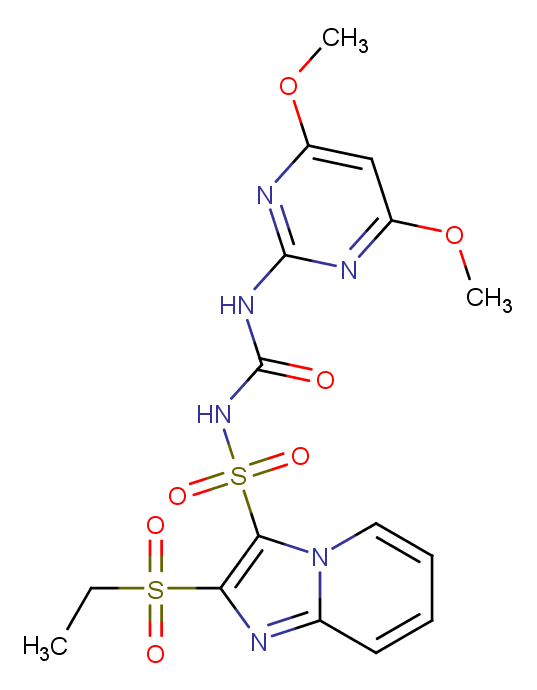

Supplement: RA-011-D1RA00914A-s081 [file RA-011-D1RA00914A-s081.png]

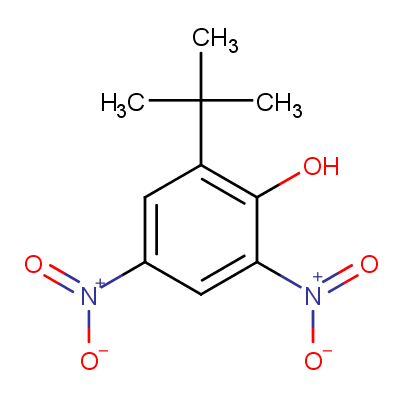

Supplement: RA-011-D1RA00914A-s082 [file RA-011-D1RA00914A-s082.png]

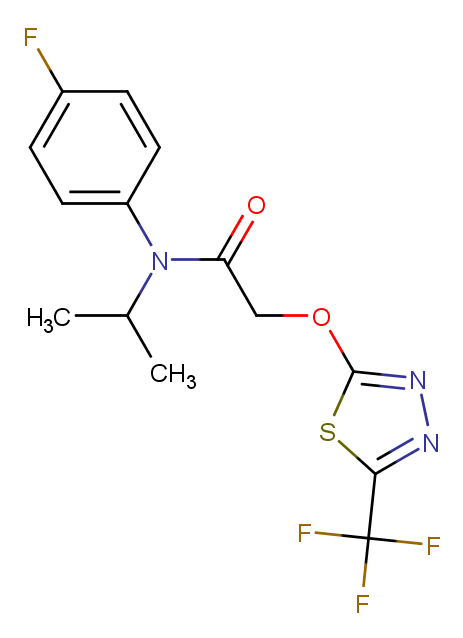

Supplement: RA-011-D1RA00914A-s083 [file RA-011-D1RA00914A-s083.png]

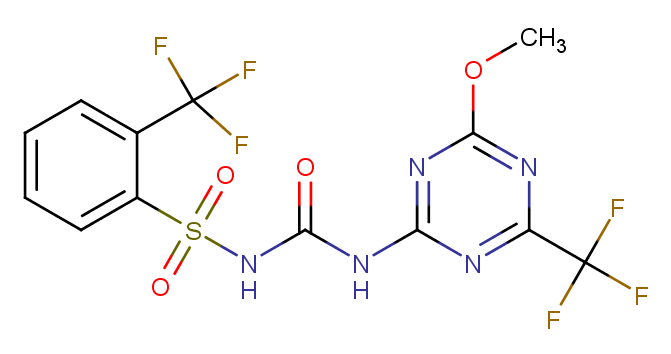

Supplement: RA-011-D1RA00914A-s084 [file RA-011-D1RA00914A-s084.png]

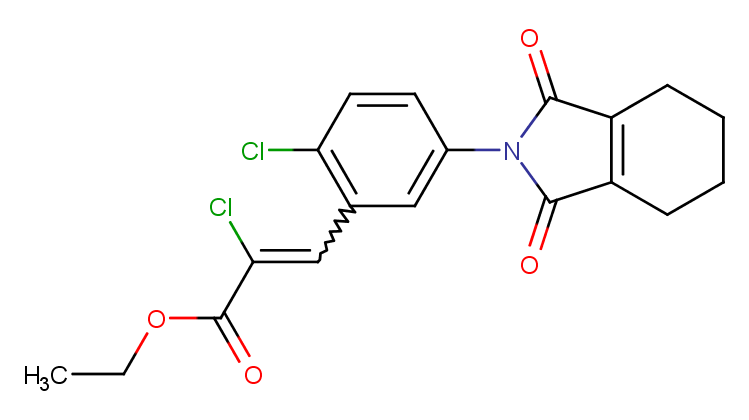

Supplement: RA-011-D1RA00914A-s085 [file RA-011-D1RA00914A-s085.png]

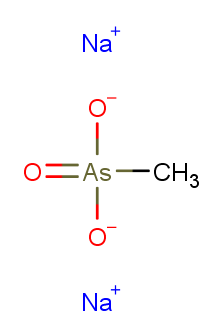

Supplement: RA-011-D1RA00914A-s086 [file RA-011-D1RA00914A-s086.png]

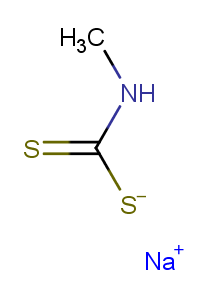

Supplement: RA-011-D1RA00914A-s087 [file RA-011-D1RA00914A-s087.png]

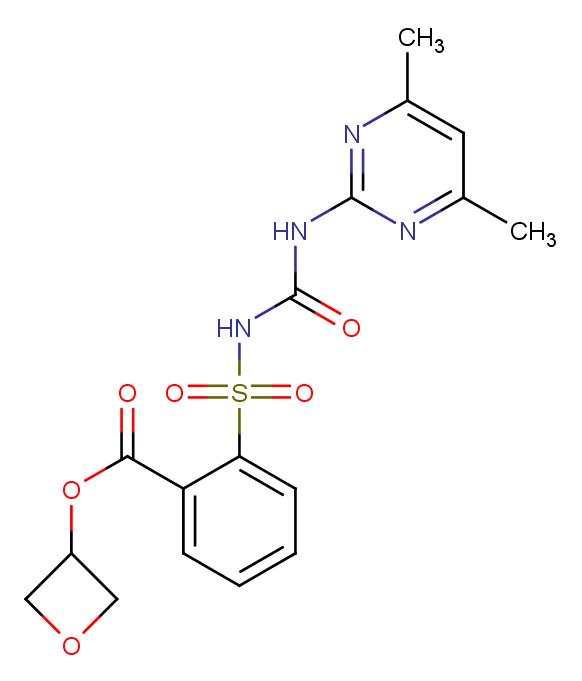

Supplement: RA-011-D1RA00914A-s088 [file RA-011-D1RA00914A-s088.png]

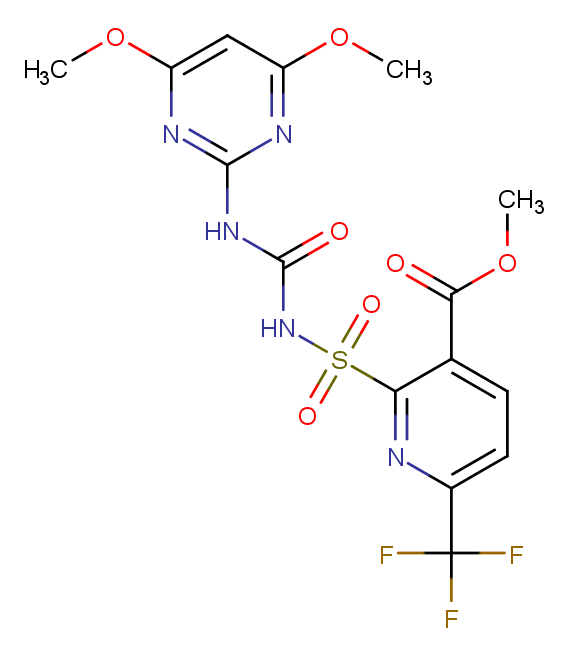

Supplement: RA-011-D1RA00914A-s089 [file RA-011-D1RA00914A-s089.png]

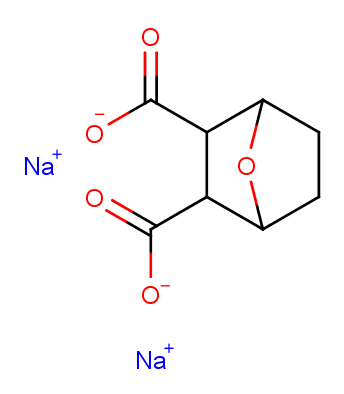

Supplement: RA-011-D1RA00914A-s090 [file RA-011-D1RA00914A-s090.png]

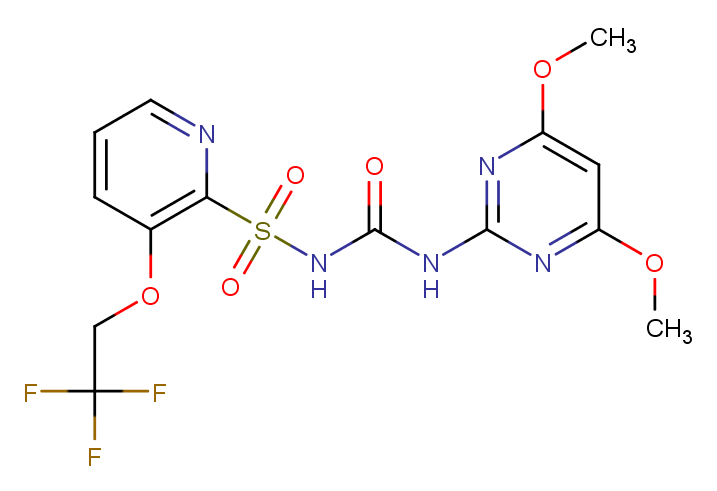

Supplement: RA-011-D1RA00914A-s091 [file RA-011-D1RA00914A-s091.png]

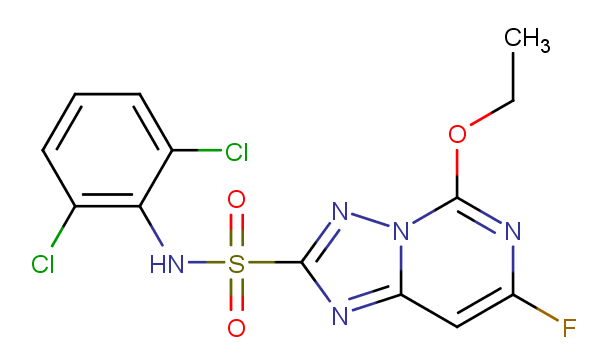

Supplement: RA-011-D1RA00914A-s092 [file RA-011-D1RA00914A-s092.png]

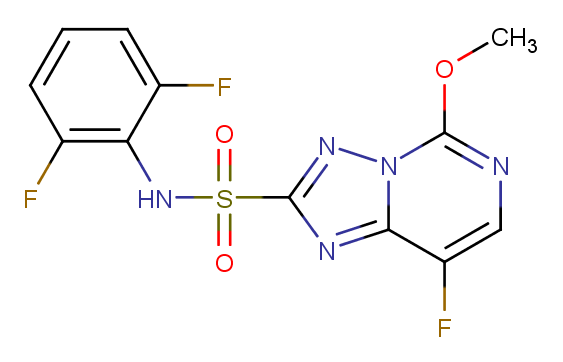

Supplement: RA-011-D1RA00914A-s093 [file RA-011-D1RA00914A-s093.png]

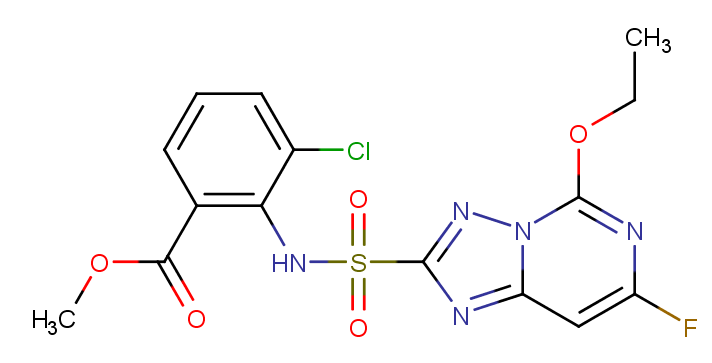

Supplement: RA-011-D1RA00914A-s094 [file RA-011-D1RA00914A-s094.png]

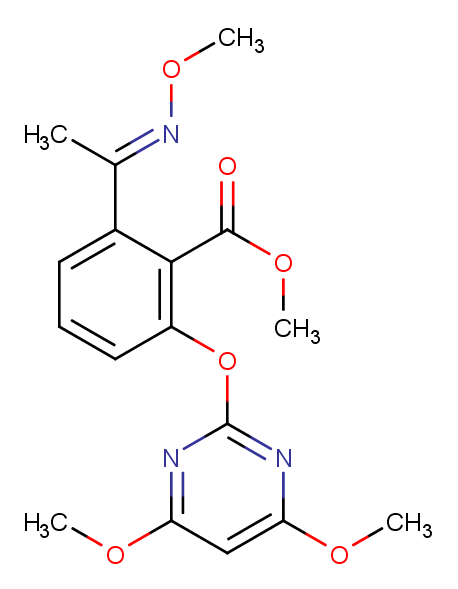

Supplement: RA-011-D1RA00914A-s095 [file RA-011-D1RA00914A-s095.png]

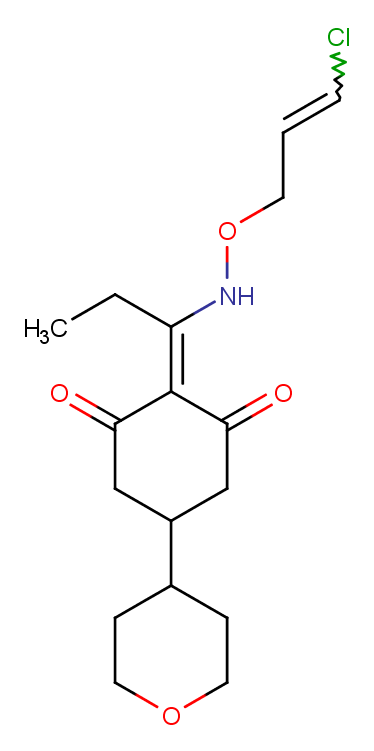

Supplement: RA-011-D1RA00914A-s096 [file RA-011-D1RA00914A-s096.png]

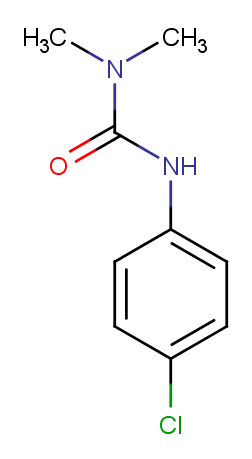

Supplement: RA-011-D1RA00914A-s097 [file RA-011-D1RA00914A-s097.png]

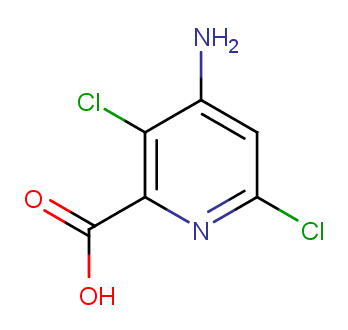

Supplement: RA-011-D1RA00914A-s098 [file RA-011-D1RA00914A-s098.png]

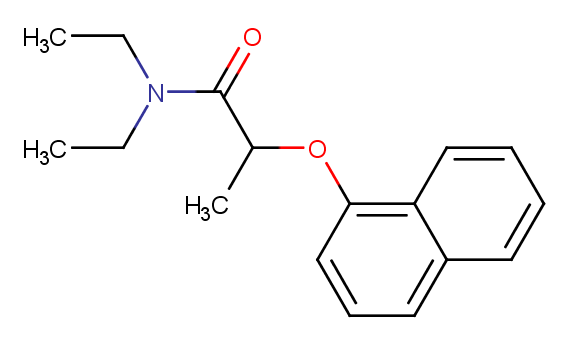

Supplement: RA-011-D1RA00914A-s099 [file RA-011-D1RA00914A-s099.png]

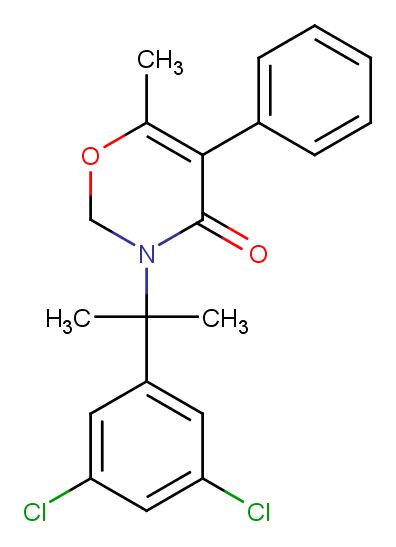

Supplement: RA-011-D1RA00914A-s100 [file RA-011-D1RA00914A-s100.png]

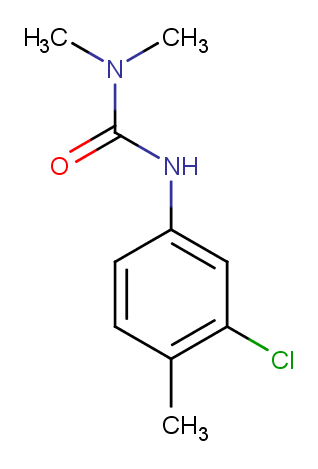

Supplement: RA-011-D1RA00914A-s101 [file RA-011-D1RA00914A-s101.png]

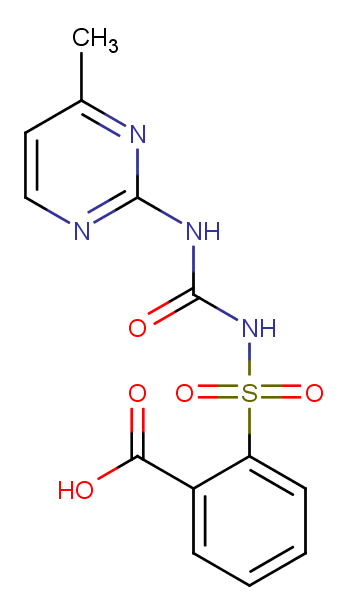

Supplement: RA-011-D1RA00914A-s102 [file RA-011-D1RA00914A-s102.png]
