# Supplementary figures and images for: Improved herbicide discovery using physico-chemical rules refined by antimalarial library screening (part 2 of 14)
Source: RSC Adv. 2021 Feb 23;11(15):8459–67. doi: 10.1039/d1ra00914a (PMC8695207; doi:10.1039/d1ra00914a)

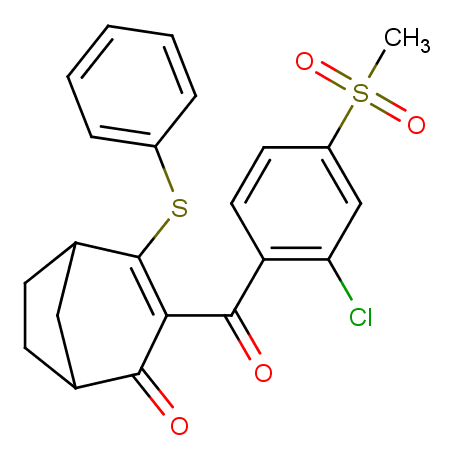

Supplement: RA-011-D1RA00914A-s103 [file RA-011-D1RA00914A-s103.png]

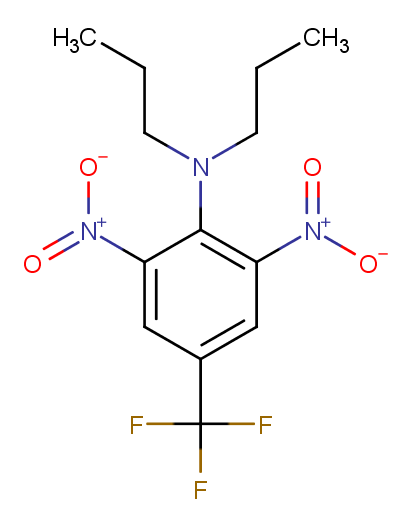

Supplement: RA-011-D1RA00914A-s104 [file RA-011-D1RA00914A-s104.png]

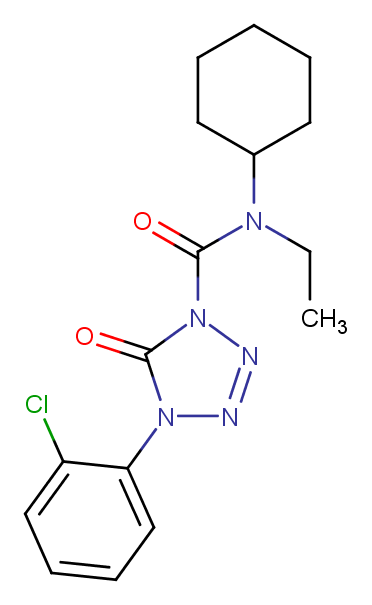

Supplement: RA-011-D1RA00914A-s105 [file RA-011-D1RA00914A-s105.png]

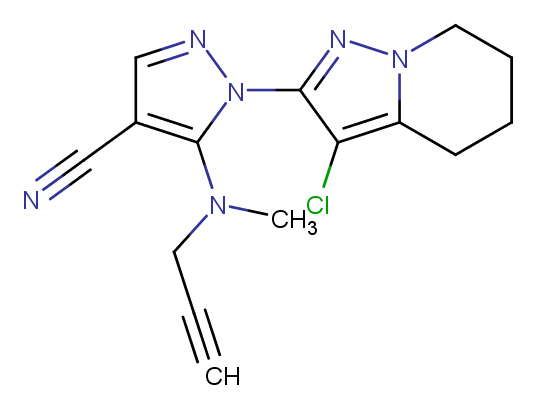

Supplement: RA-011-D1RA00914A-s106 [file RA-011-D1RA00914A-s106.png]

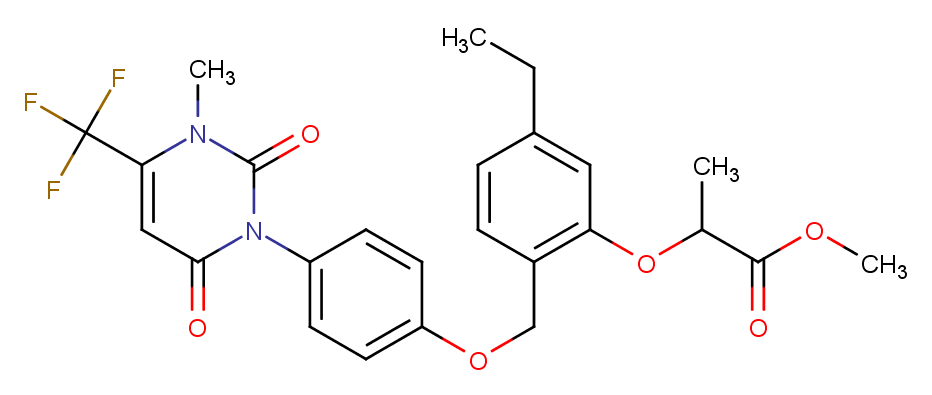

Supplement: RA-011-D1RA00914A-s107 [file RA-011-D1RA00914A-s107.png]

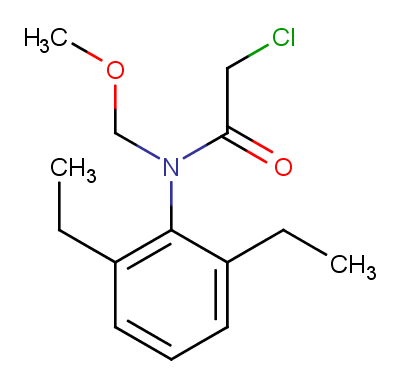

Supplement: RA-011-D1RA00914A-s108 [file RA-011-D1RA00914A-s108.png]

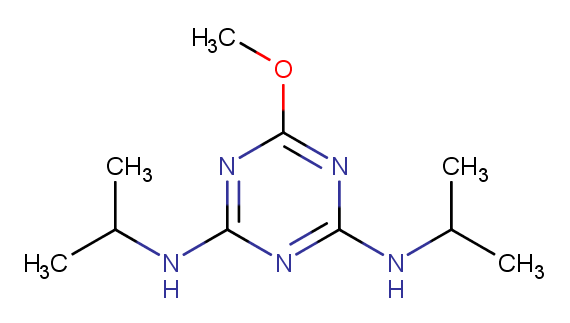

Supplement: RA-011-D1RA00914A-s109 [file RA-011-D1RA00914A-s109.png]

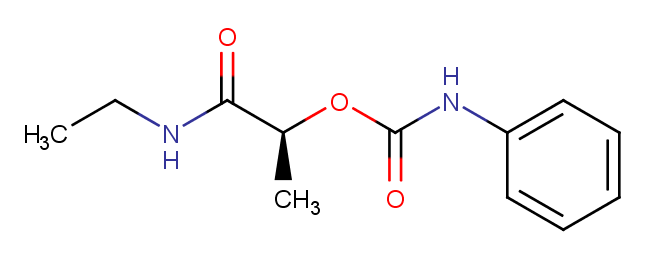

Supplement: RA-011-D1RA00914A-s110 [file RA-011-D1RA00914A-s110.png]

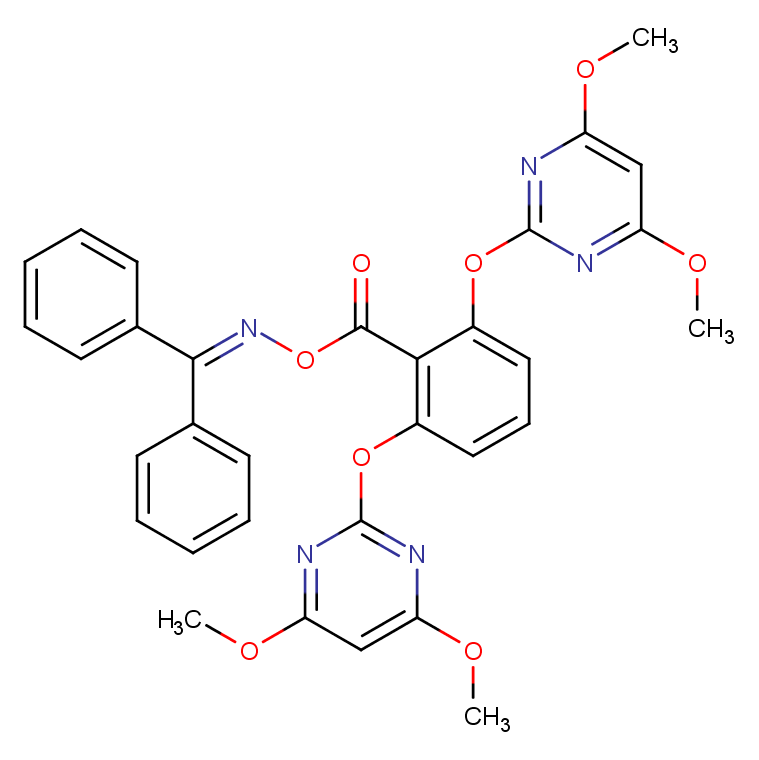

Supplement: RA-011-D1RA00914A-s111 [file RA-011-D1RA00914A-s111.png]

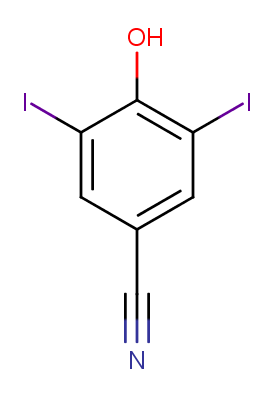

Supplement: RA-011-D1RA00914A-s112 [file RA-011-D1RA00914A-s112.png]

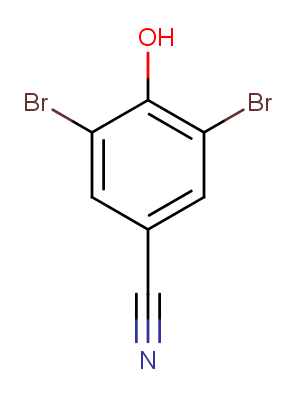

Supplement: RA-011-D1RA00914A-s113 [file RA-011-D1RA00914A-s113.png]

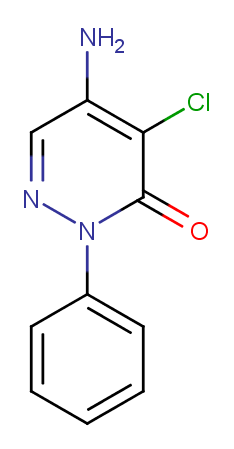

Supplement: RA-011-D1RA00914A-s114 [file RA-011-D1RA00914A-s114.png]

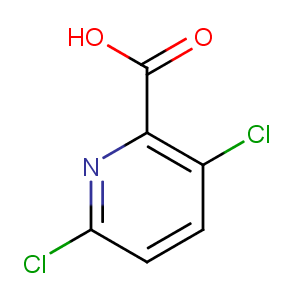

Supplement: RA-011-D1RA00914A-s115 [file RA-011-D1RA00914A-s115.png]

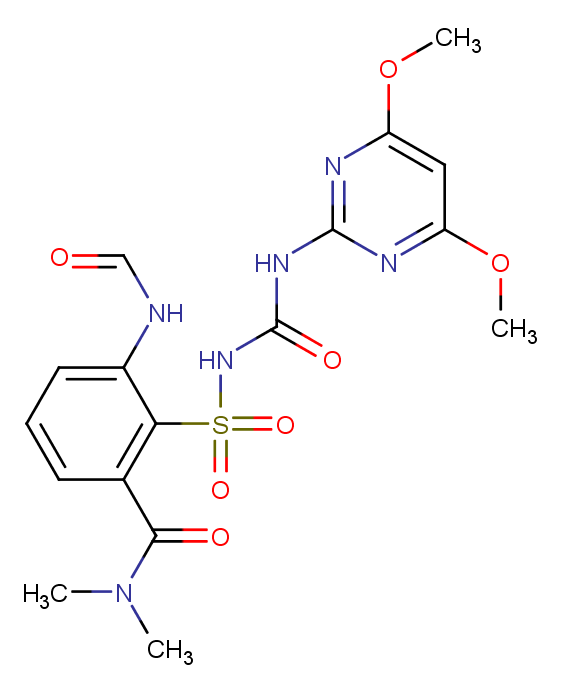

Supplement: RA-011-D1RA00914A-s116 [file RA-011-D1RA00914A-s116.png]

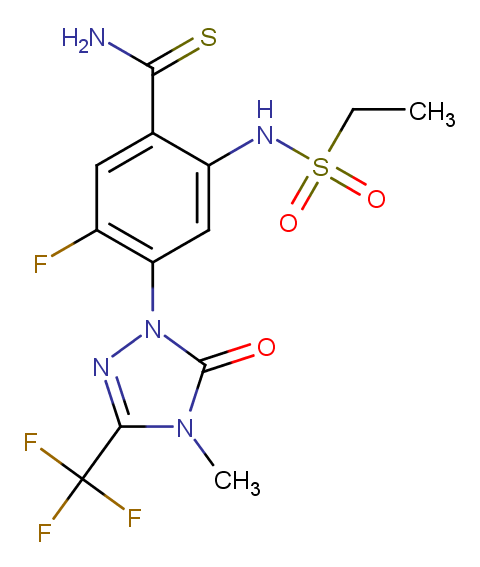

Supplement: RA-011-D1RA00914A-s117 [file RA-011-D1RA00914A-s117.png]

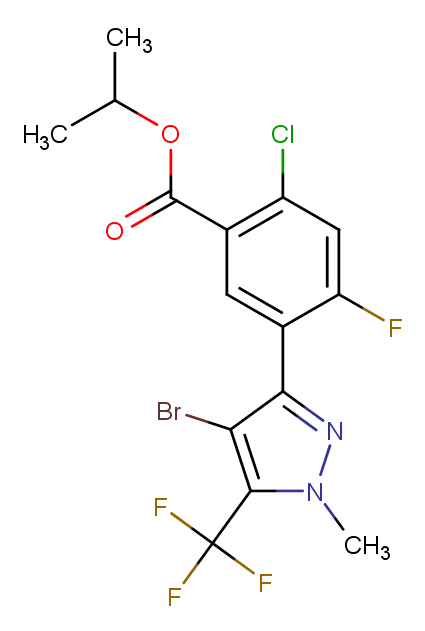

Supplement: RA-011-D1RA00914A-s118 [file RA-011-D1RA00914A-s118.png]

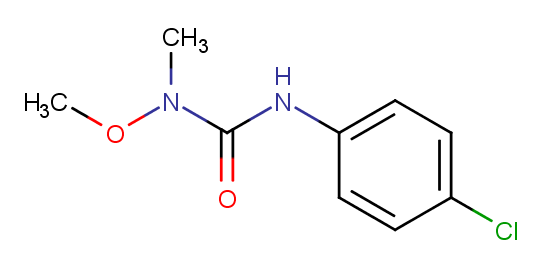

Supplement: RA-011-D1RA00914A-s119 [file RA-011-D1RA00914A-s119.png]

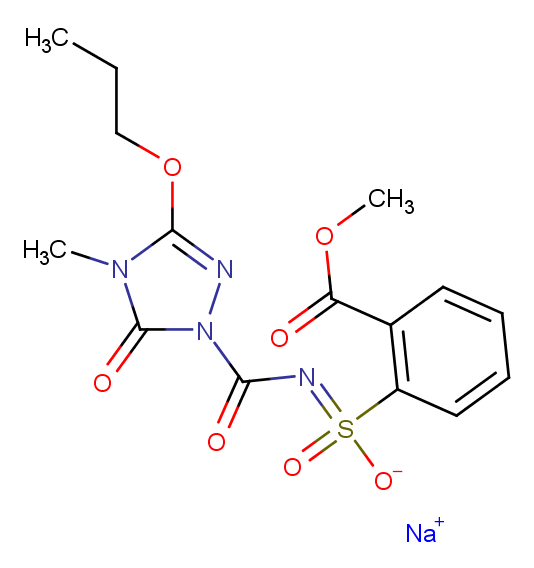

Supplement: RA-011-D1RA00914A-s120 [file RA-011-D1RA00914A-s120.png]

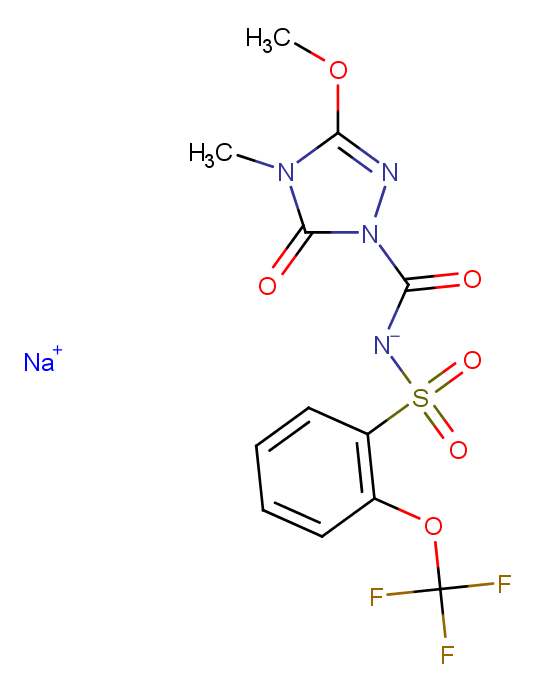

Supplement: RA-011-D1RA00914A-s121 [file RA-011-D1RA00914A-s121.png]

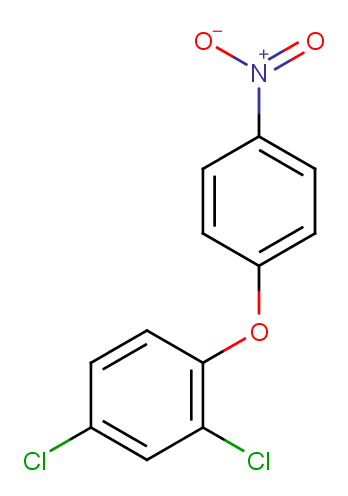

Supplement: RA-011-D1RA00914A-s122 [file RA-011-D1RA00914A-s122.png]

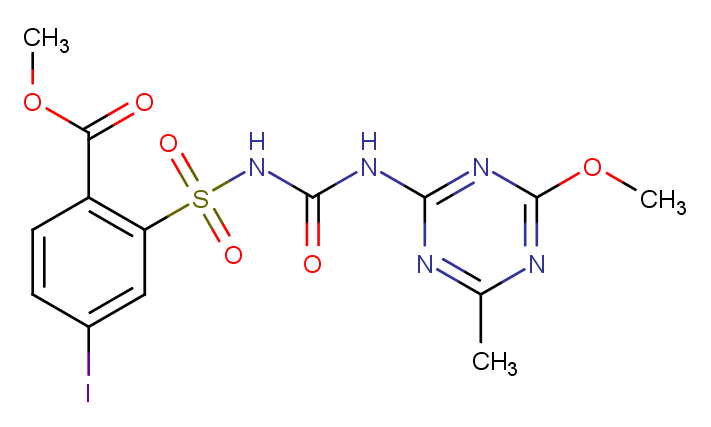

Supplement: RA-011-D1RA00914A-s123 [file RA-011-D1RA00914A-s123.png]

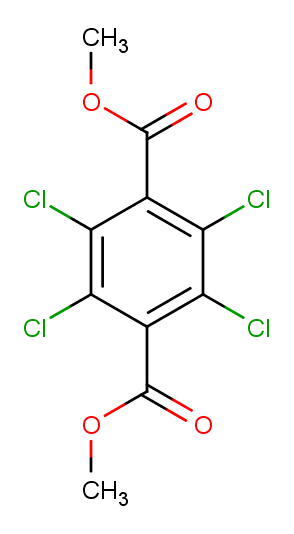

Supplement: RA-011-D1RA00914A-s124 [file RA-011-D1RA00914A-s124.png]

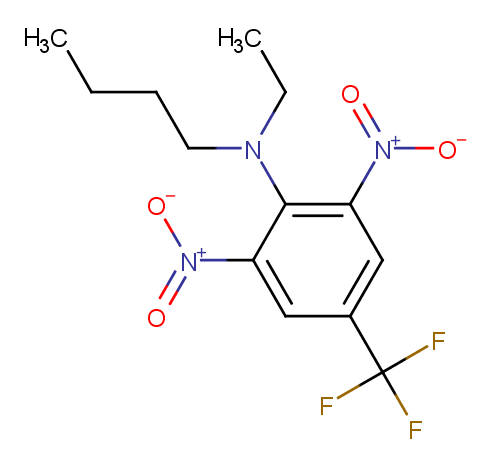

Supplement: RA-011-D1RA00914A-s125 [file RA-011-D1RA00914A-s125.png]

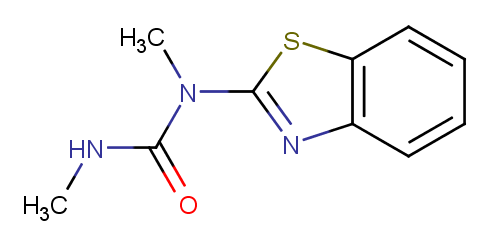

Supplement: RA-011-D1RA00914A-s126 [file RA-011-D1RA00914A-s126.png]

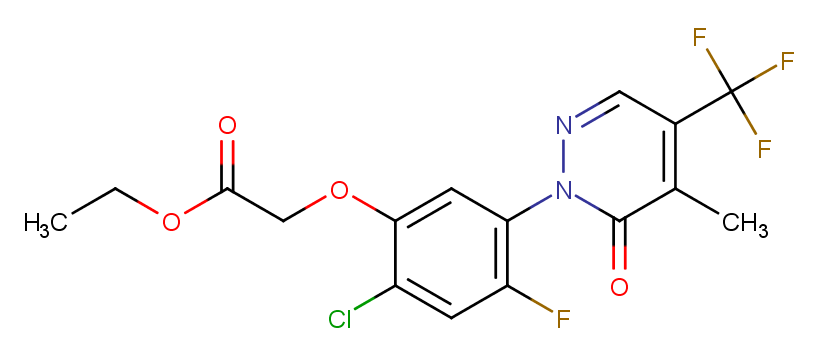

Supplement: RA-011-D1RA00914A-s127 [file RA-011-D1RA00914A-s127.png]

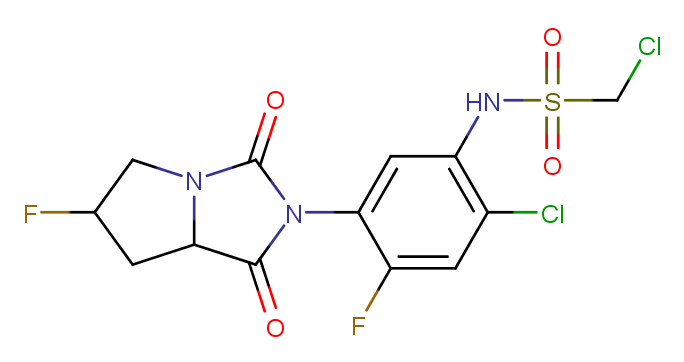

Supplement: RA-011-D1RA00914A-s128 [file RA-011-D1RA00914A-s128.png]

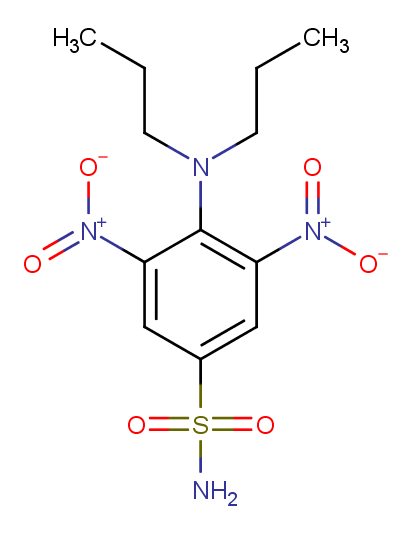

Supplement: RA-011-D1RA00914A-s129 [file RA-011-D1RA00914A-s129.png]

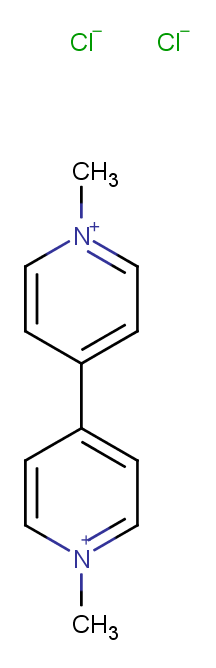

Supplement: RA-011-D1RA00914A-s130 [file RA-011-D1RA00914A-s130.png]

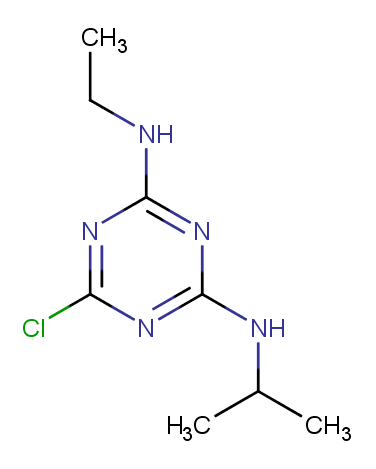

Supplement: RA-011-D1RA00914A-s131 [file RA-011-D1RA00914A-s131.png]

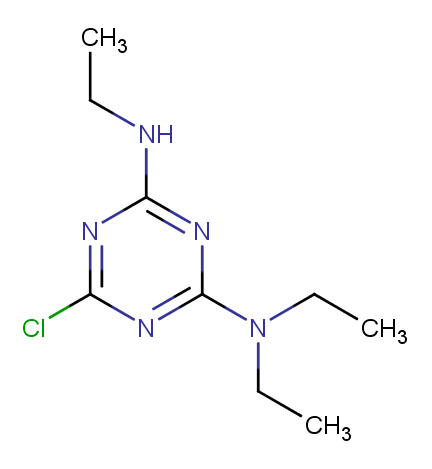

Supplement: RA-011-D1RA00914A-s132 [file RA-011-D1RA00914A-s132.png]

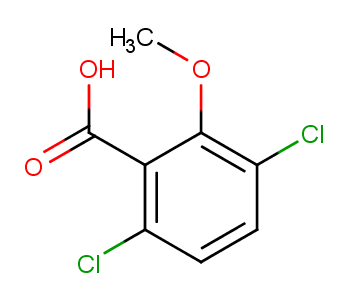

Supplement: RA-011-D1RA00914A-s133 [file RA-011-D1RA00914A-s133.png]

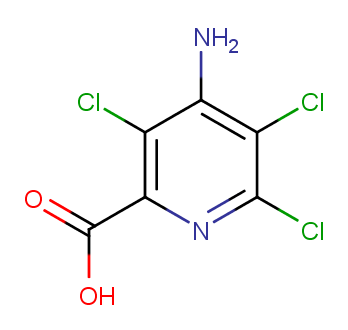

Supplement: RA-011-D1RA00914A-s134 [file RA-011-D1RA00914A-s134.png]

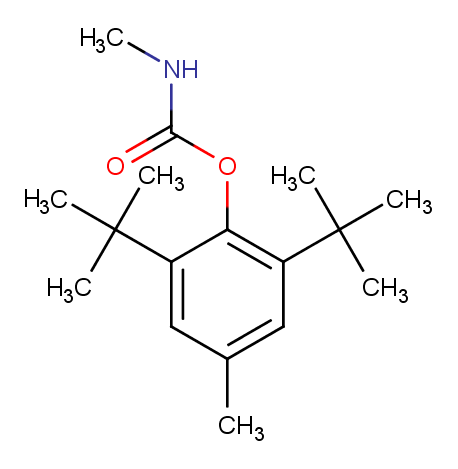

Supplement: RA-011-D1RA00914A-s135 [file RA-011-D1RA00914A-s135.png]

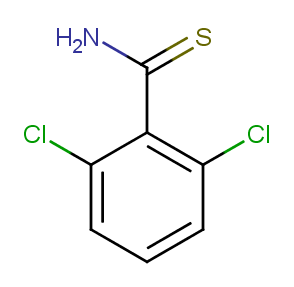

Supplement: RA-011-D1RA00914A-s136 [file RA-011-D1RA00914A-s136.png]

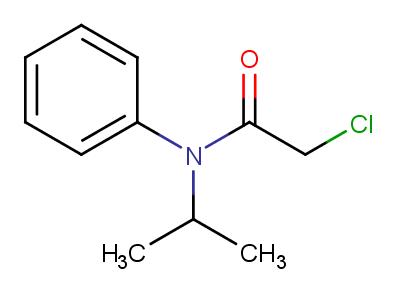

Supplement: RA-011-D1RA00914A-s137 [file RA-011-D1RA00914A-s137.png]

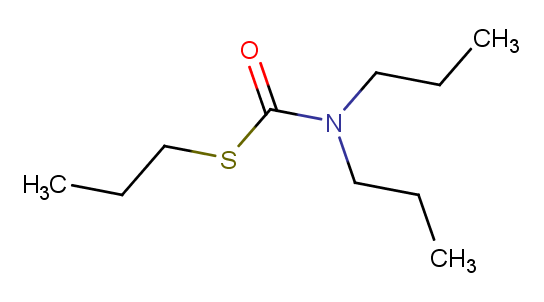

Supplement: RA-011-D1RA00914A-s138 [file RA-011-D1RA00914A-s138.png]

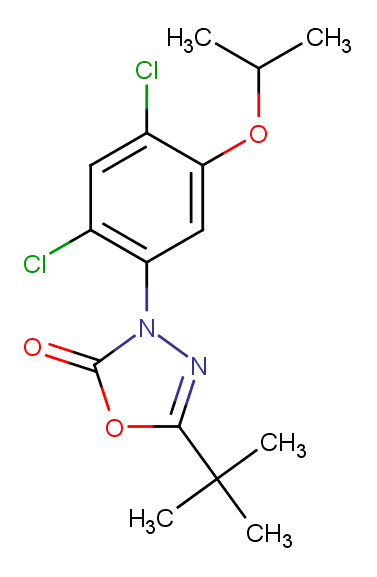

Supplement: RA-011-D1RA00914A-s139 [file RA-011-D1RA00914A-s139.png]

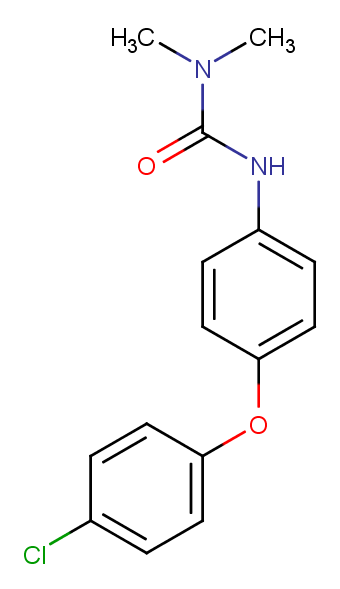

Supplement: RA-011-D1RA00914A-s140 [file RA-011-D1RA00914A-s140.png]

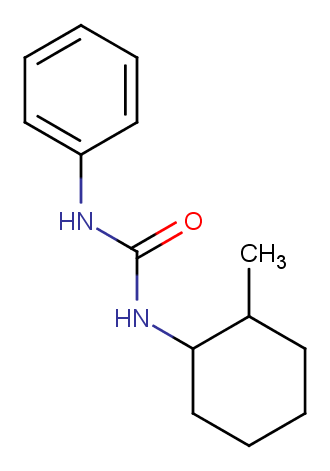

Supplement: RA-011-D1RA00914A-s141 [file RA-011-D1RA00914A-s141.png]

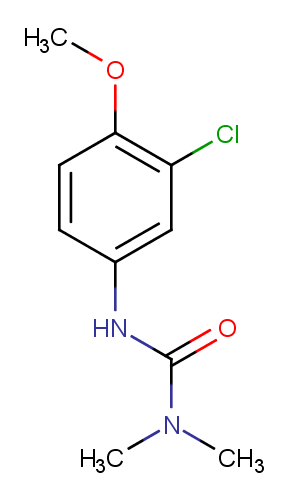

Supplement: RA-011-D1RA00914A-s142 [file RA-011-D1RA00914A-s142.png]

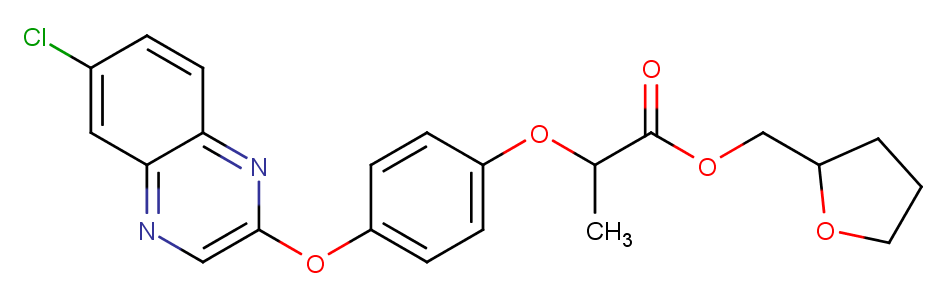

Supplement: RA-011-D1RA00914A-s143 [file RA-011-D1RA00914A-s143.png]

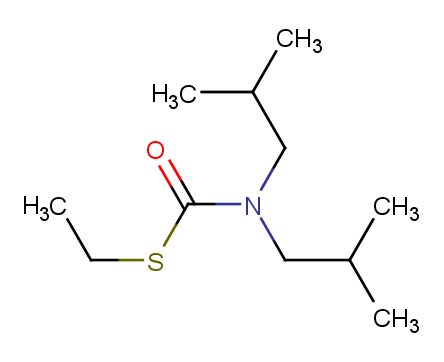

Supplement: RA-011-D1RA00914A-s144 [file RA-011-D1RA00914A-s144.png]

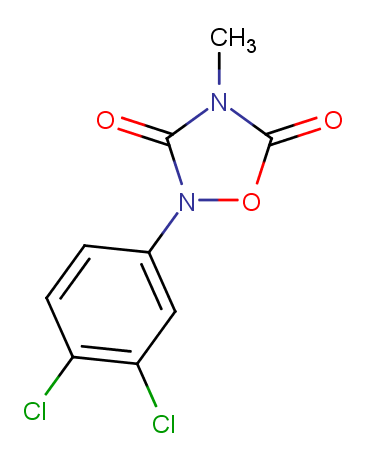

Supplement: RA-011-D1RA00914A-s145 [file RA-011-D1RA00914A-s145.png]

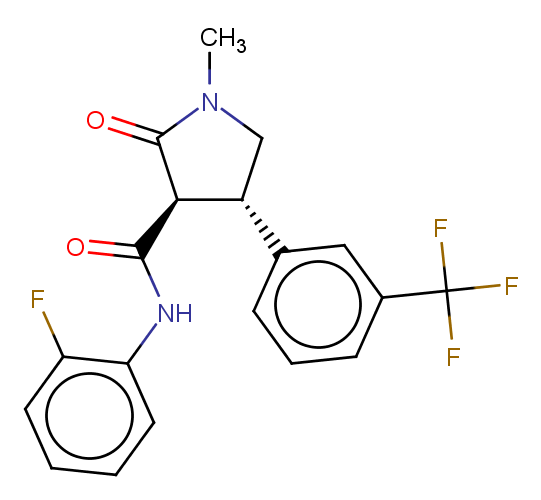

Supplement: RA-011-D1RA00914A-s146 [file RA-011-D1RA00914A-s146.png]

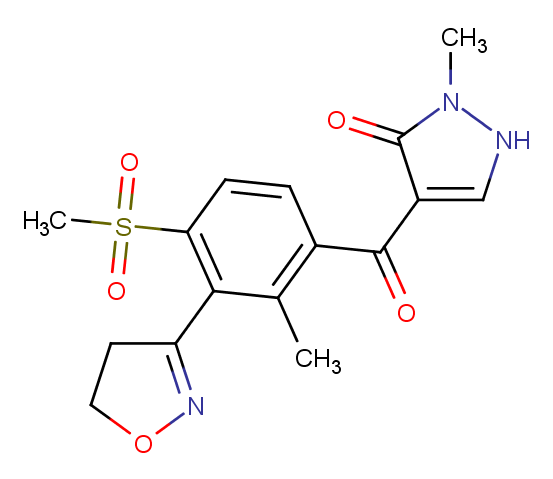

Supplement: RA-011-D1RA00914A-s147 [file RA-011-D1RA00914A-s147.png]

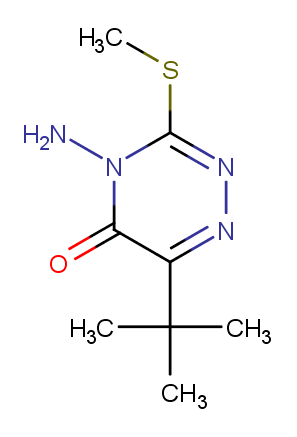

Supplement: RA-011-D1RA00914A-s148 [file RA-011-D1RA00914A-s148.png]

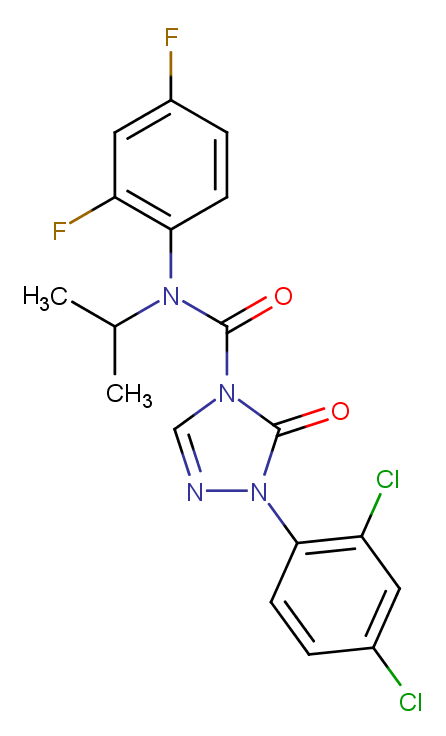

Supplement: RA-011-D1RA00914A-s149 [file RA-011-D1RA00914A-s149.png]

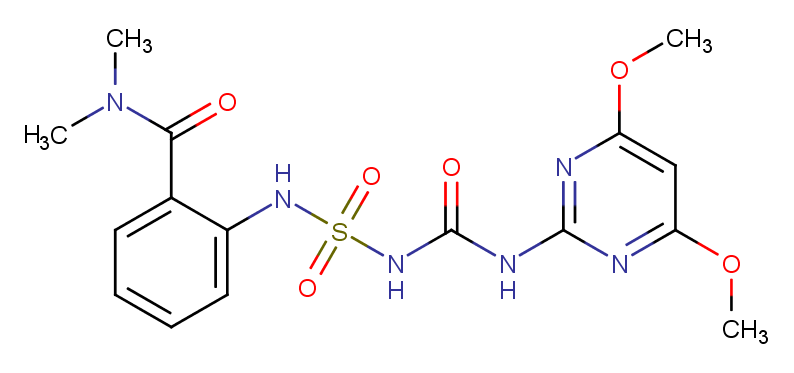

Supplement: RA-011-D1RA00914A-s150 [file RA-011-D1RA00914A-s150.png]

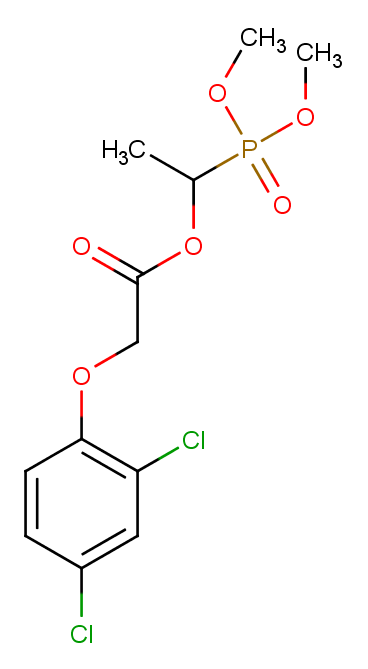

Supplement: RA-011-D1RA00914A-s151 [file RA-011-D1RA00914A-s151.png]

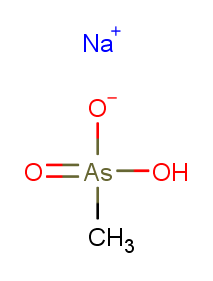

Supplement: RA-011-D1RA00914A-s152 [file RA-011-D1RA00914A-s152.png]

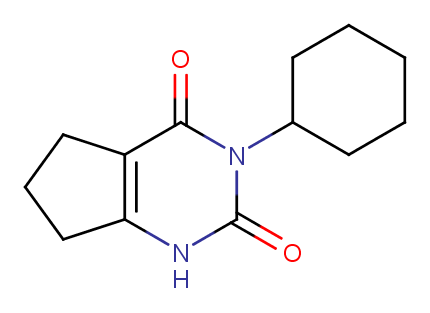

Supplement: RA-011-D1RA00914A-s153 [file RA-011-D1RA00914A-s153.png]

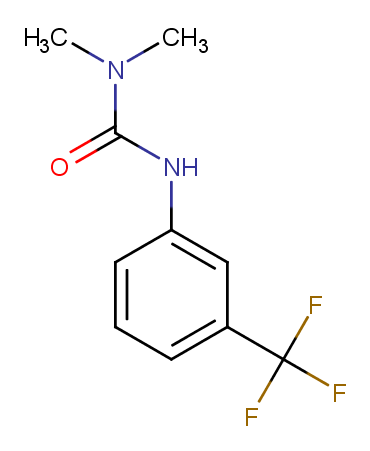

Supplement: RA-011-D1RA00914A-s154 [file RA-011-D1RA00914A-s154.png]

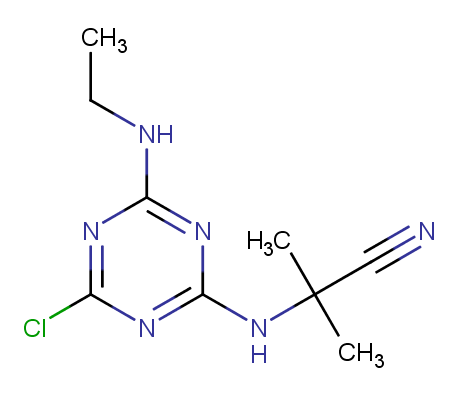

Supplement: RA-011-D1RA00914A-s155 [file RA-011-D1RA00914A-s155.png]

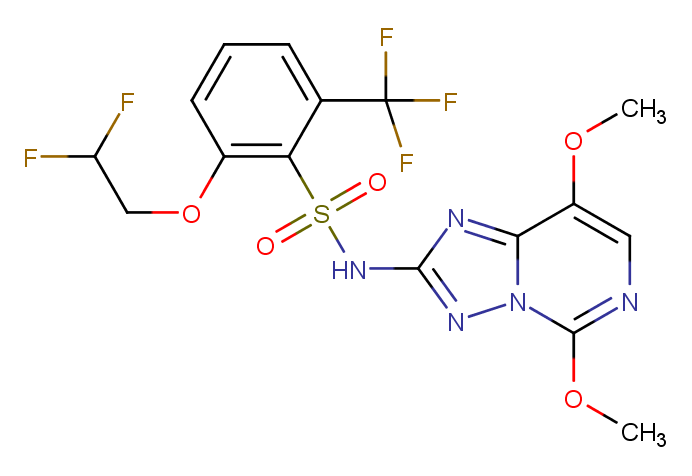

Supplement: RA-011-D1RA00914A-s156 [file RA-011-D1RA00914A-s156.png]

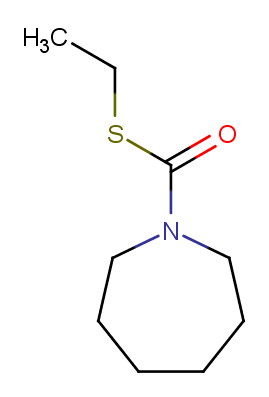

Supplement: RA-011-D1RA00914A-s157 [file RA-011-D1RA00914A-s157.png]

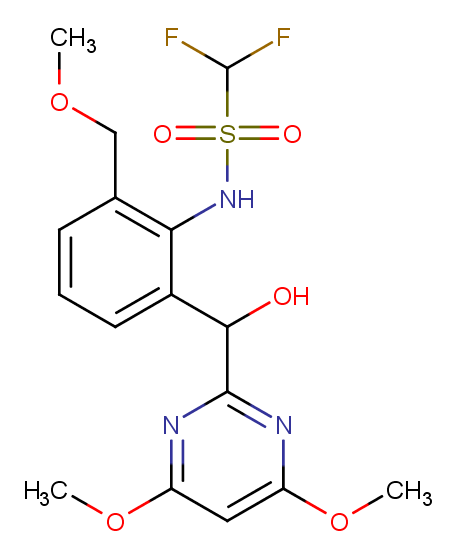

Supplement: RA-011-D1RA00914A-s158 [file RA-011-D1RA00914A-s158.png]

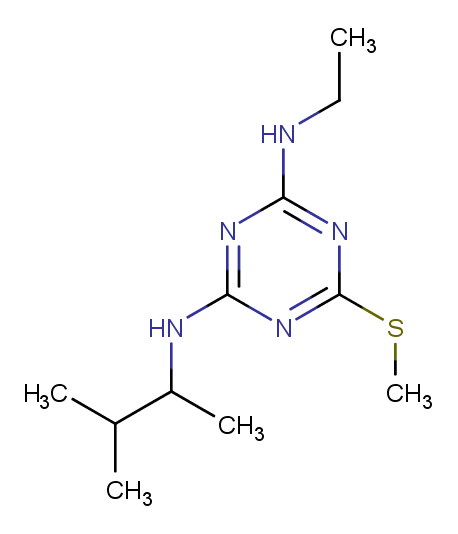

Supplement: RA-011-D1RA00914A-s159 [file RA-011-D1RA00914A-s159.png]

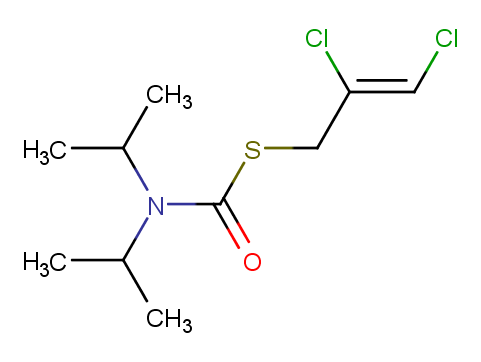

Supplement: RA-011-D1RA00914A-s160 [file RA-011-D1RA00914A-s160.png]

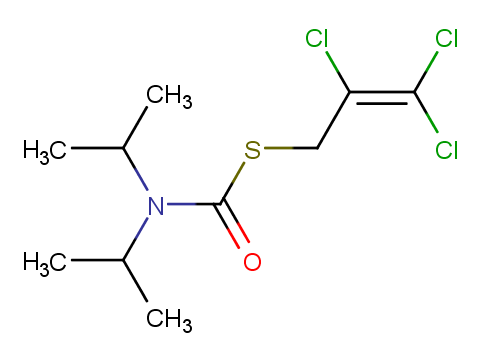

Supplement: RA-011-D1RA00914A-s161 [file RA-011-D1RA00914A-s161.png]

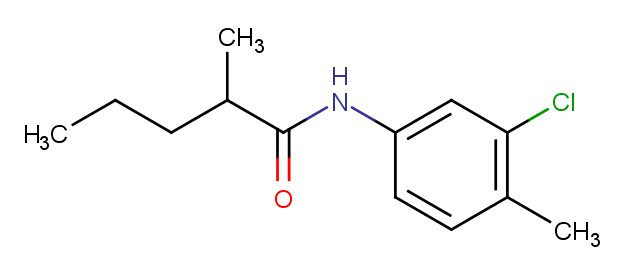

Supplement: RA-011-D1RA00914A-s162 [file RA-011-D1RA00914A-s162.png]

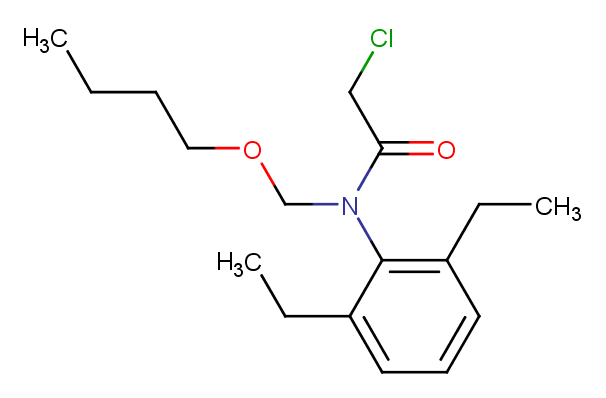

Supplement: RA-011-D1RA00914A-s163 [file RA-011-D1RA00914A-s163.png]

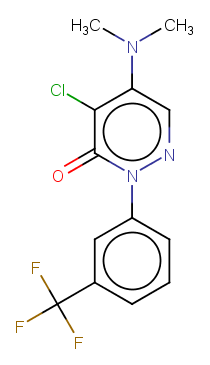

Supplement: RA-011-D1RA00914A-s164 [file RA-011-D1RA00914A-s164.png]

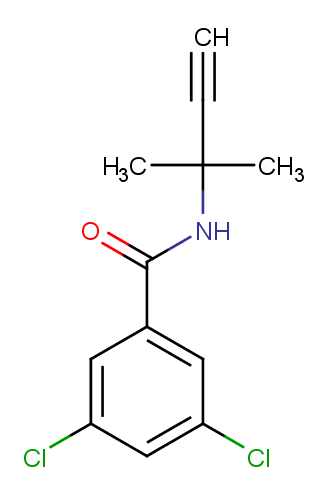

Supplement: RA-011-D1RA00914A-s165 [file RA-011-D1RA00914A-s165.png]

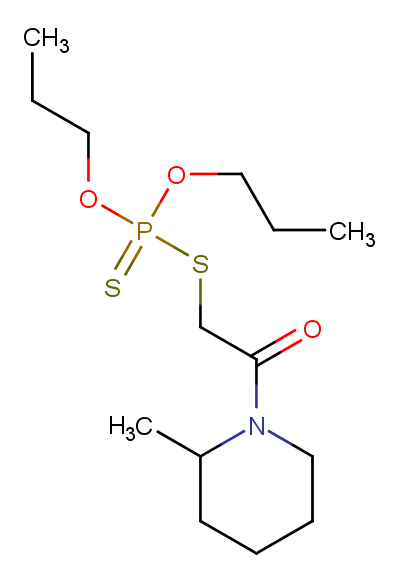

Supplement: RA-011-D1RA00914A-s166 [file RA-011-D1RA00914A-s166.png]

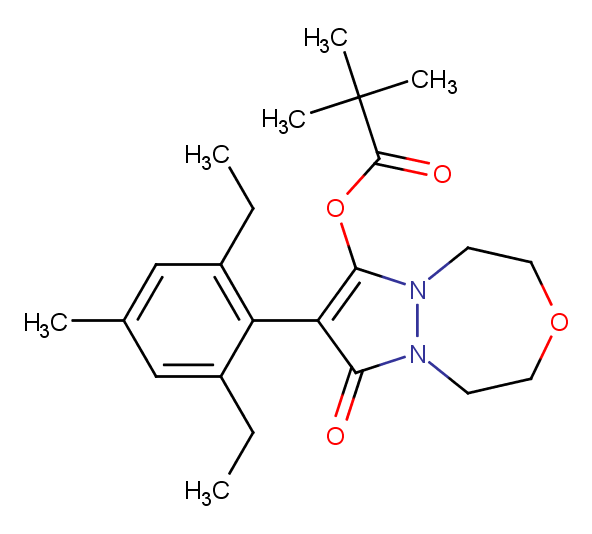

Supplement: RA-011-D1RA00914A-s167 [file RA-011-D1RA00914A-s167.png]

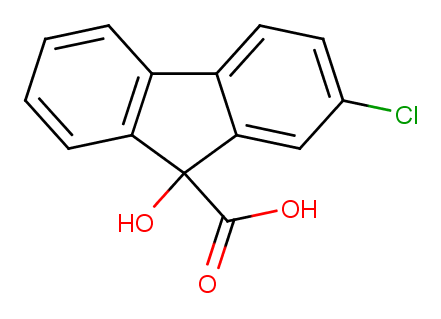

Supplement: RA-011-D1RA00914A-s168 [file RA-011-D1RA00914A-s168.png]

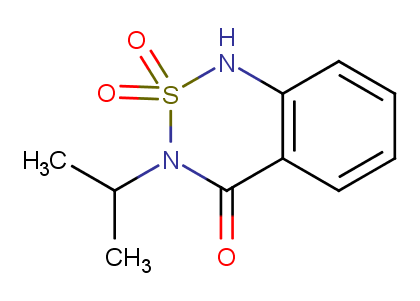

Supplement: RA-011-D1RA00914A-s169 [file RA-011-D1RA00914A-s169.png]

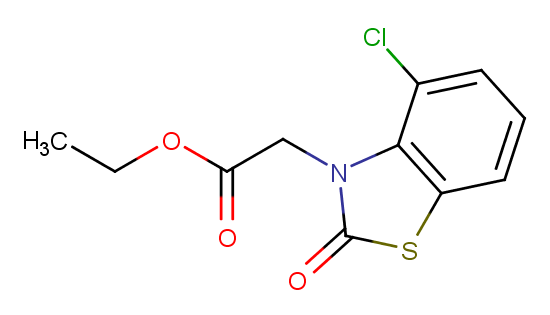

Supplement: RA-011-D1RA00914A-s170 [file RA-011-D1RA00914A-s170.png]

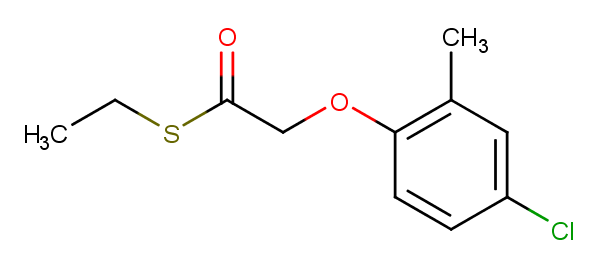

Supplement: RA-011-D1RA00914A-s171 [file RA-011-D1RA00914A-s171.png]

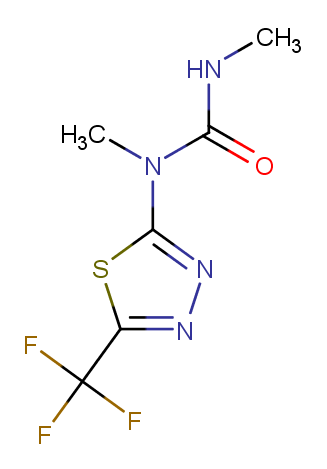

Supplement: RA-011-D1RA00914A-s172 [file RA-011-D1RA00914A-s172.png]

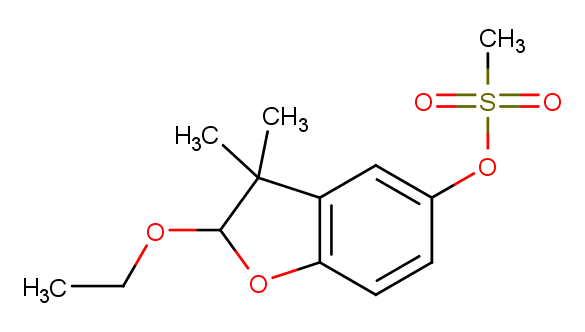

Supplement: RA-011-D1RA00914A-s173 [file RA-011-D1RA00914A-s173.png]

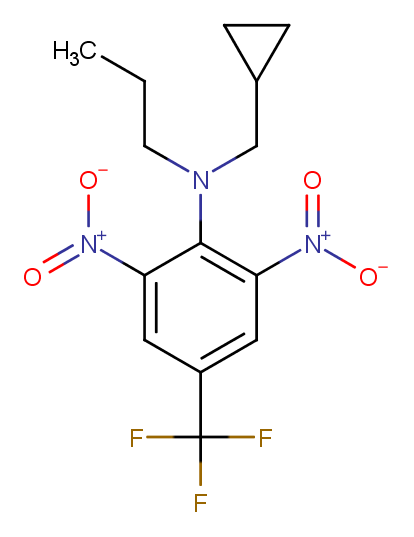

Supplement: RA-011-D1RA00914A-s174 [file RA-011-D1RA00914A-s174.png]

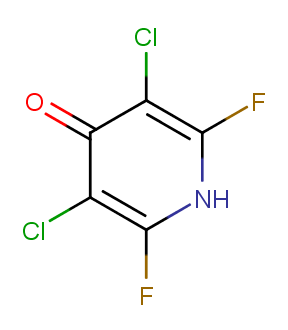

Supplement: RA-011-D1RA00914A-s175 [file RA-011-D1RA00914A-s175.png]

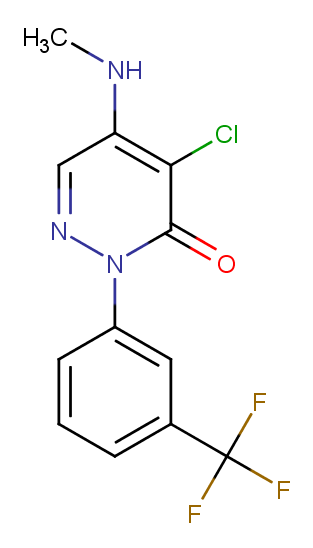

Supplement: RA-011-D1RA00914A-s176 [file RA-011-D1RA00914A-s176.png]

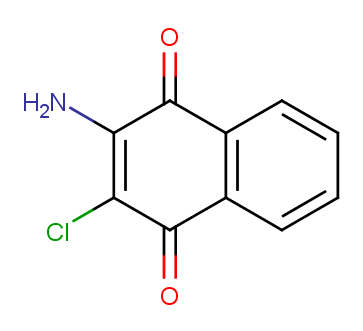

Supplement: RA-011-D1RA00914A-s177 [file RA-011-D1RA00914A-s177.png]

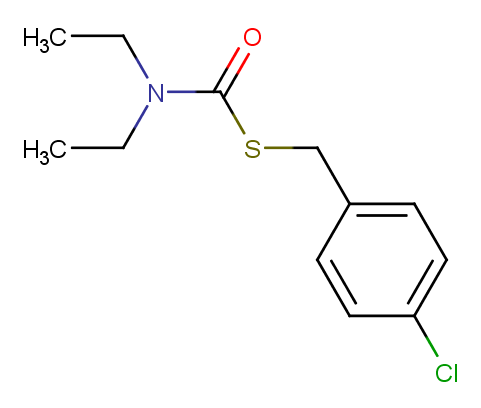

Supplement: RA-011-D1RA00914A-s178 [file RA-011-D1RA00914A-s178.png]

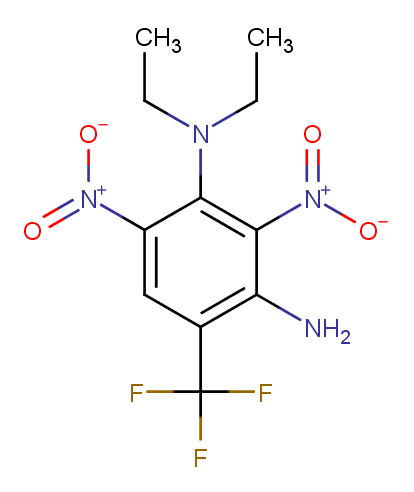

Supplement: RA-011-D1RA00914A-s179 [file RA-011-D1RA00914A-s179.png]

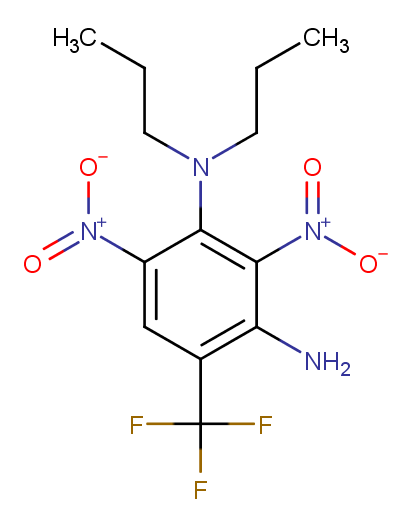

Supplement: RA-011-D1RA00914A-s180 [file RA-011-D1RA00914A-s180.png]

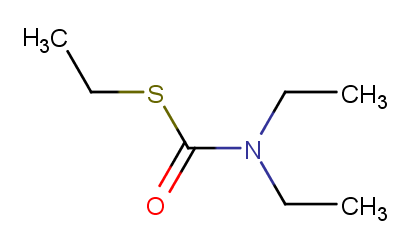

Supplement: RA-011-D1RA00914A-s181 [file RA-011-D1RA00914A-s181.png]

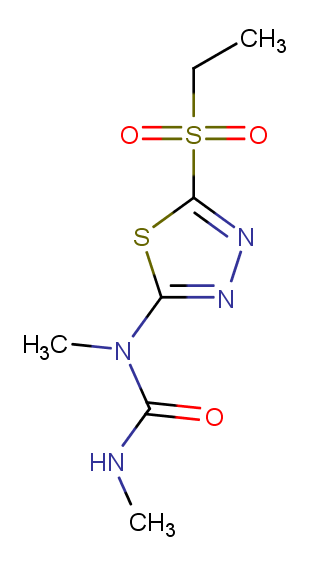

Supplement: RA-011-D1RA00914A-s182 [file RA-011-D1RA00914A-s182.png]

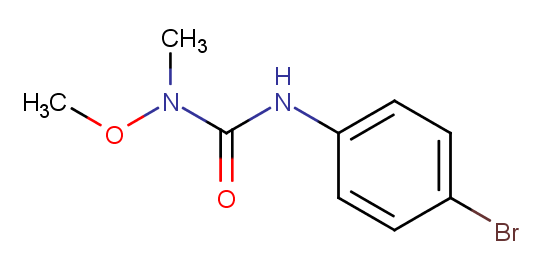

Supplement: RA-011-D1RA00914A-s183 [file RA-011-D1RA00914A-s183.png]

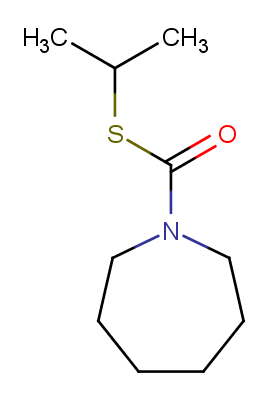

Supplement: RA-011-D1RA00914A-s184 [file RA-011-D1RA00914A-s184.png]

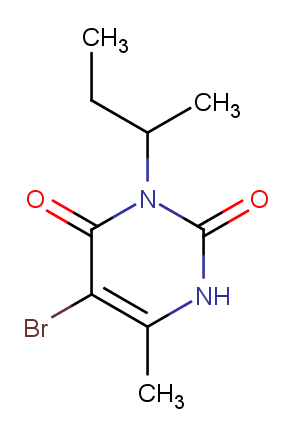

Supplement: RA-011-D1RA00914A-s185 [file RA-011-D1RA00914A-s185.png]

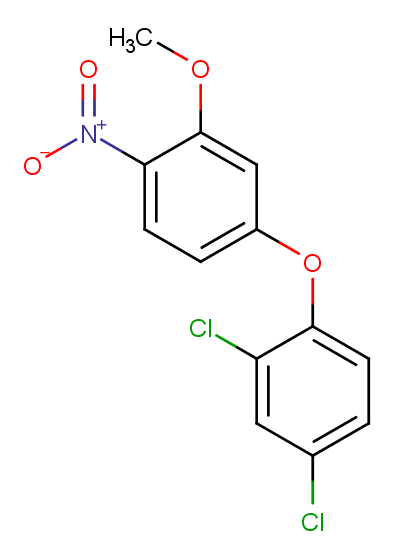

Supplement: RA-011-D1RA00914A-s186 [file RA-011-D1RA00914A-s186.png]

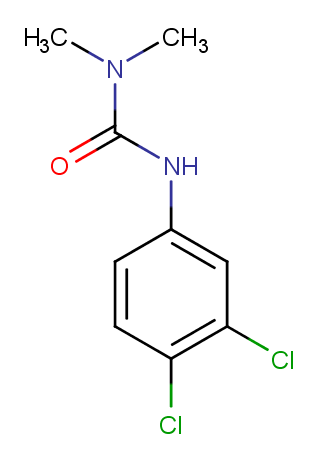

Supplement: RA-011-D1RA00914A-s187 [file RA-011-D1RA00914A-s187.png]

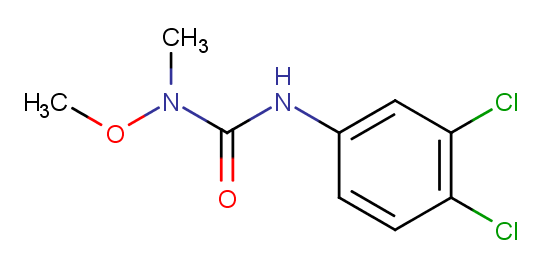

Supplement: RA-011-D1RA00914A-s188 [file RA-011-D1RA00914A-s188.png]

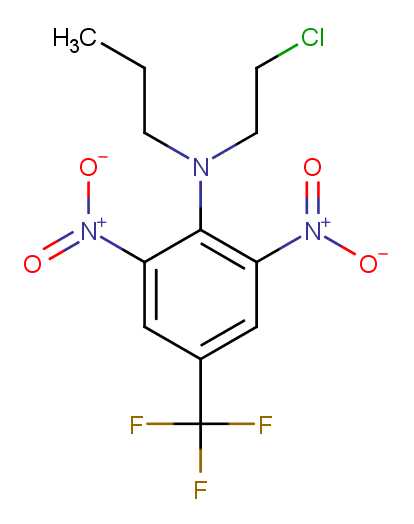

Supplement: RA-011-D1RA00914A-s189 [file RA-011-D1RA00914A-s189.png]

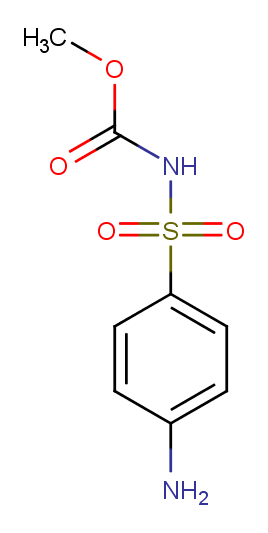

Supplement: RA-011-D1RA00914A-s190 [file RA-011-D1RA00914A-s190.png]

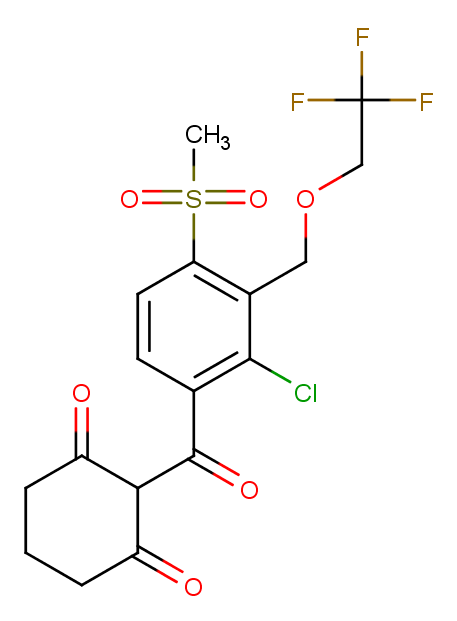

Supplement: RA-011-D1RA00914A-s191 [file RA-011-D1RA00914A-s191.png]

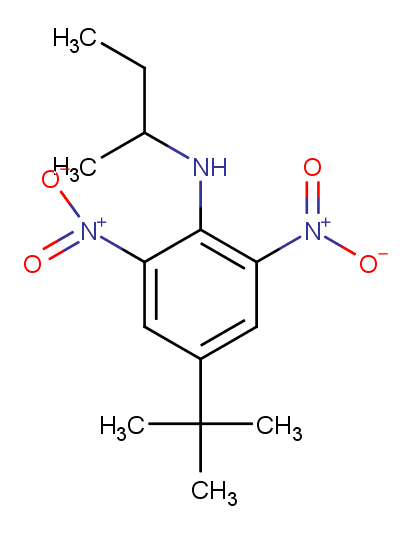

Supplement: RA-011-D1RA00914A-s192 [file RA-011-D1RA00914A-s192.png]

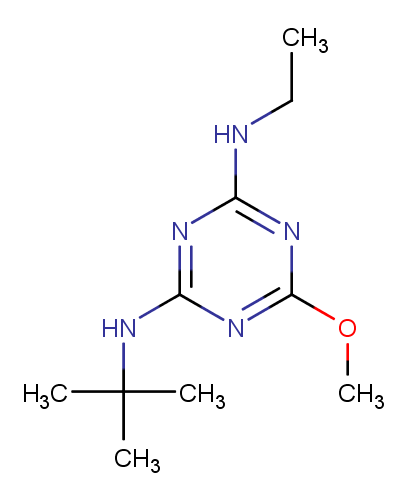

Supplement: RA-011-D1RA00914A-s193 [file RA-011-D1RA00914A-s193.png]

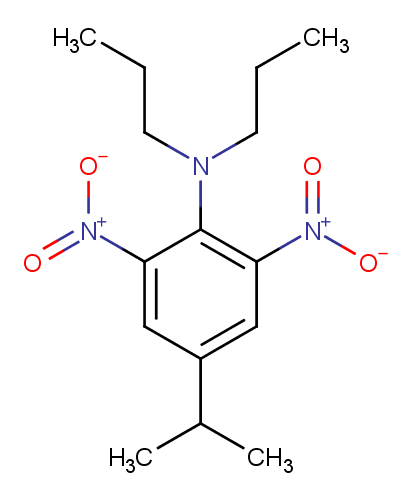

Supplement: RA-011-D1RA00914A-s194 [file RA-011-D1RA00914A-s194.png]

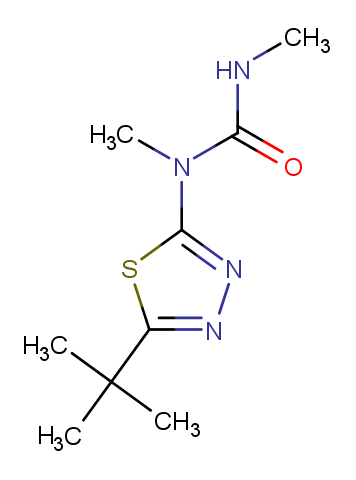

Supplement: RA-011-D1RA00914A-s195 [file RA-011-D1RA00914A-s195.png]

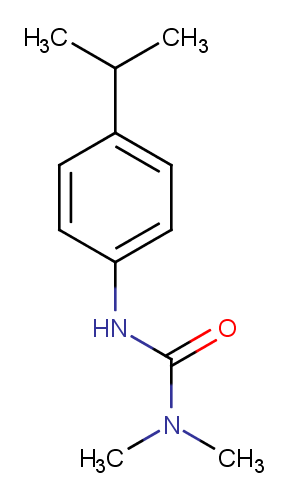

Supplement: RA-011-D1RA00914A-s196 [file RA-011-D1RA00914A-s196.png]

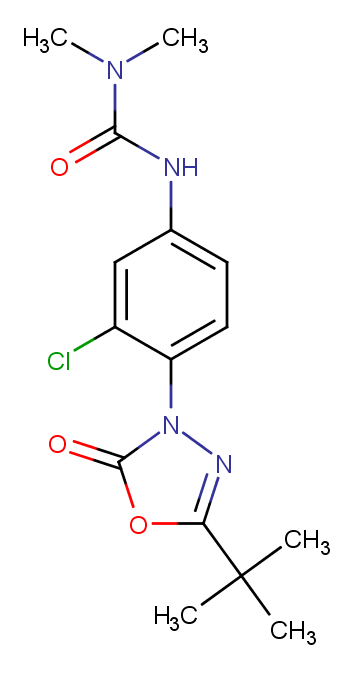

Supplement: RA-011-D1RA00914A-s197 [file RA-011-D1RA00914A-s197.png]

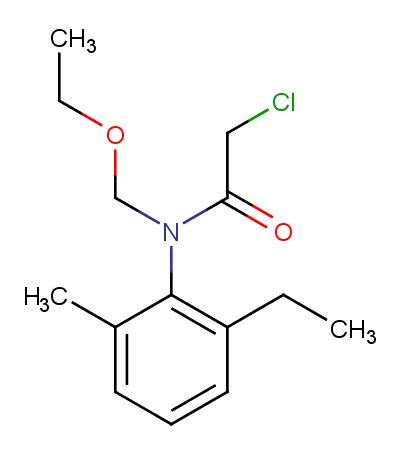

Supplement: RA-011-D1RA00914A-s198 [file RA-011-D1RA00914A-s198.png]

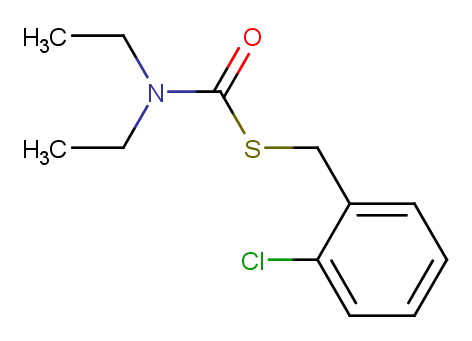

Supplement: RA-011-D1RA00914A-s199 [file RA-011-D1RA00914A-s199.png]

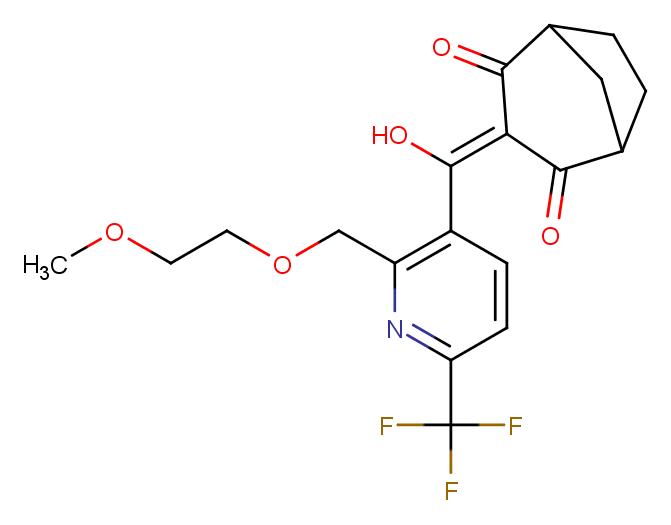

Supplement: RA-011-D1RA00914A-s200 [file RA-011-D1RA00914A-s200.png]

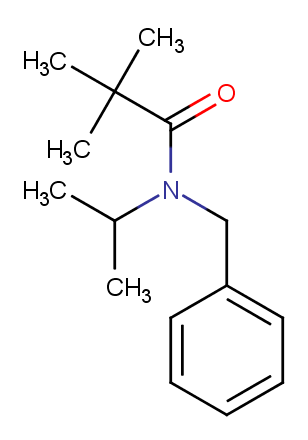

Supplement: RA-011-D1RA00914A-s201 [file RA-011-D1RA00914A-s201.png]

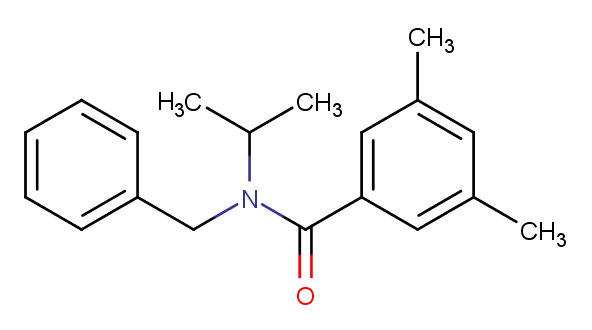

Supplement: RA-011-D1RA00914A-s202 [file RA-011-D1RA00914A-s202.png]
