# Supplementary figures and images for: Improved herbicide discovery using physico-chemical rules refined by antimalarial library screening (part 3 of 14)
Source: RSC Adv. 2021 Feb 23;11(15):8459–67. doi: 10.1039/d1ra00914a (PMC8695207; doi:10.1039/d1ra00914a)

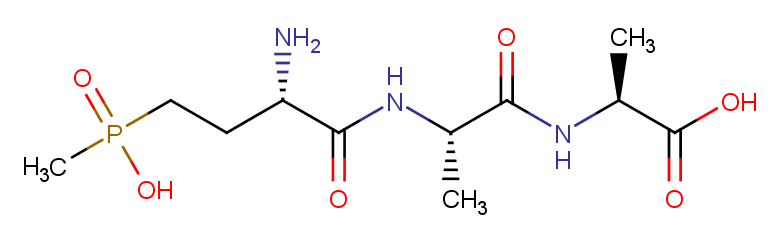

Supplement: RA-011-D1RA00914A-s203 [file RA-011-D1RA00914A-s203.png]

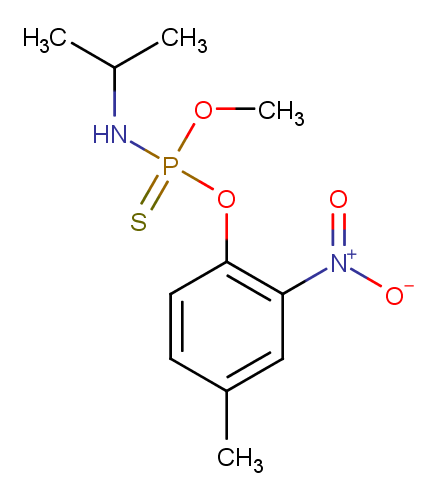

Supplement: RA-011-D1RA00914A-s204 [file RA-011-D1RA00914A-s204.png]

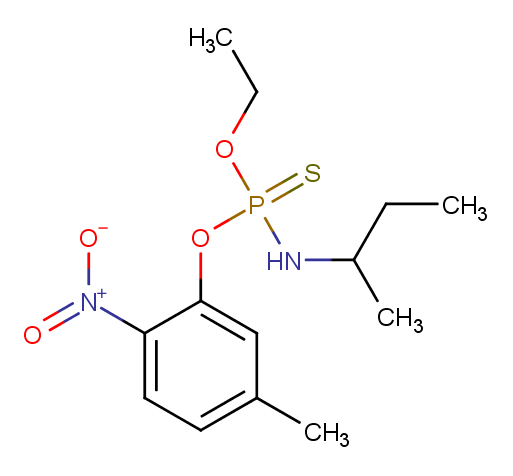

Supplement: RA-011-D1RA00914A-s205 [file RA-011-D1RA00914A-s205.png]

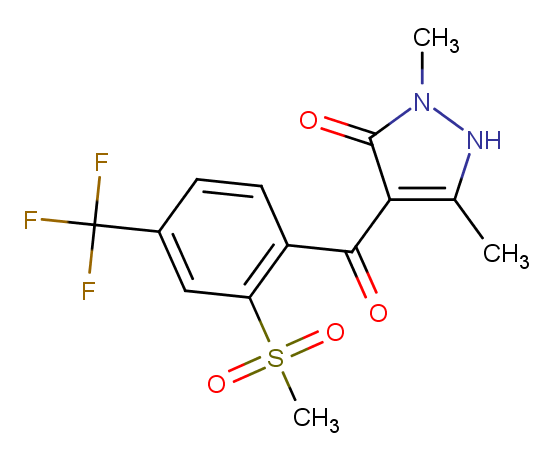

Supplement: RA-011-D1RA00914A-s206 [file RA-011-D1RA00914A-s206.png]

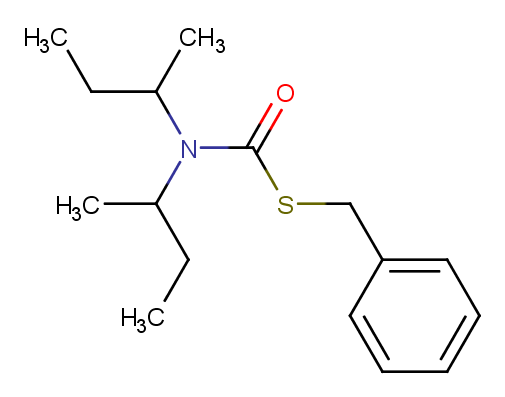

Supplement: RA-011-D1RA00914A-s207 [file RA-011-D1RA00914A-s207.png]

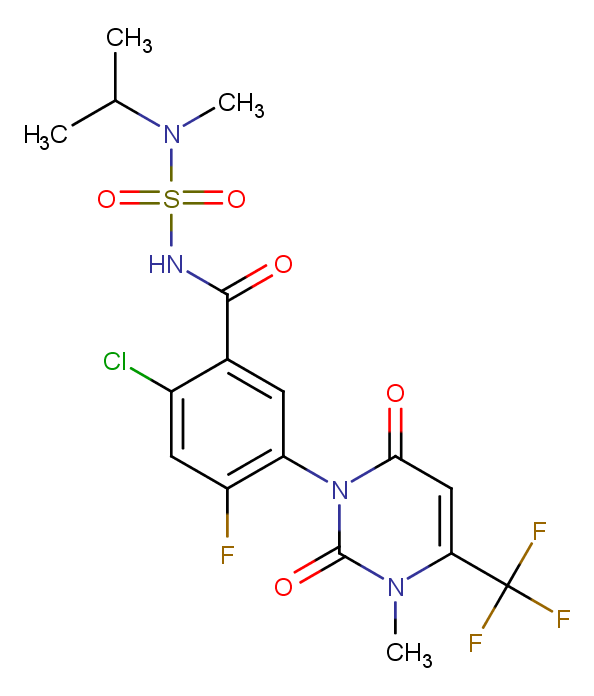

Supplement: RA-011-D1RA00914A-s208 [file RA-011-D1RA00914A-s208.png]

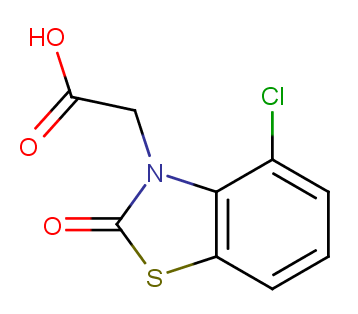

Supplement: RA-011-D1RA00914A-s209 [file RA-011-D1RA00914A-s209.png]

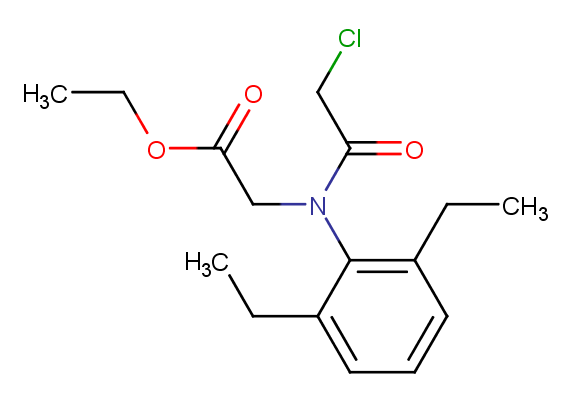

Supplement: RA-011-D1RA00914A-s210 [file RA-011-D1RA00914A-s210.png]

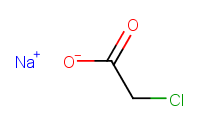

Supplement: RA-011-D1RA00914A-s211 [file RA-011-D1RA00914A-s211.png]

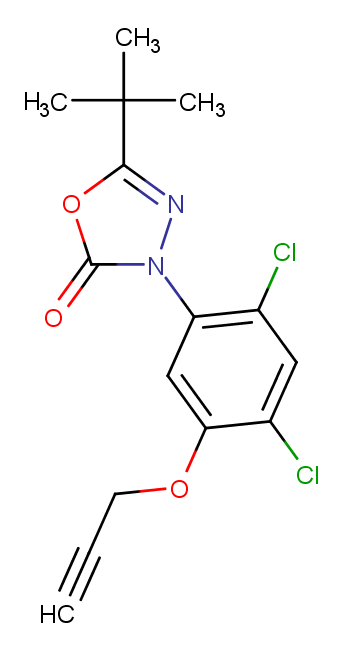

Supplement: RA-011-D1RA00914A-s212 [file RA-011-D1RA00914A-s212.png]

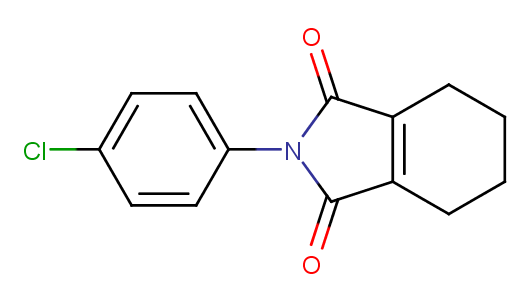

Supplement: RA-011-D1RA00914A-s213 [file RA-011-D1RA00914A-s213.png]

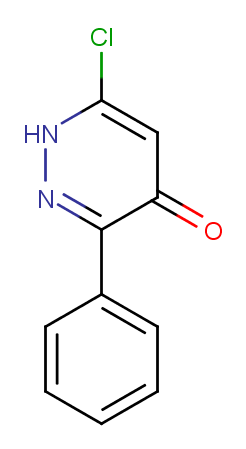

Supplement: RA-011-D1RA00914A-s214 [file RA-011-D1RA00914A-s214.png]

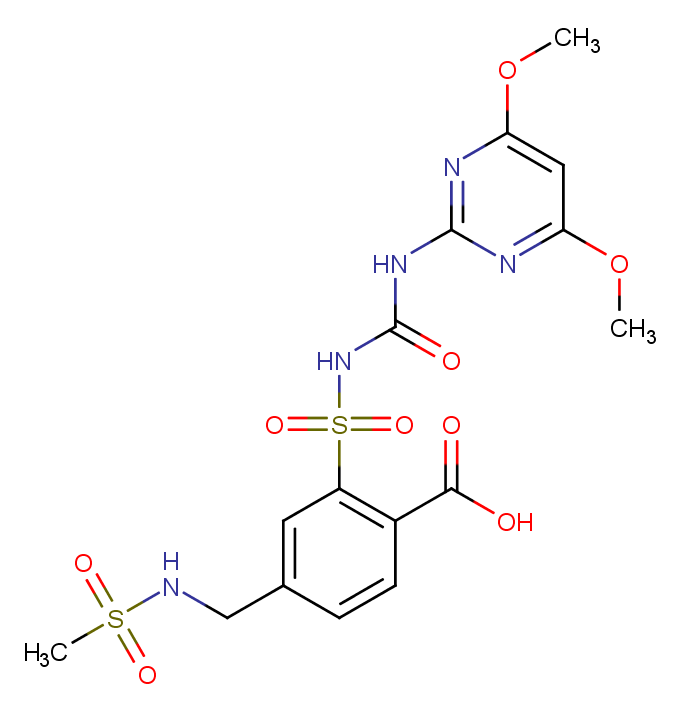

Supplement: RA-011-D1RA00914A-s215 [file RA-011-D1RA00914A-s215.png]

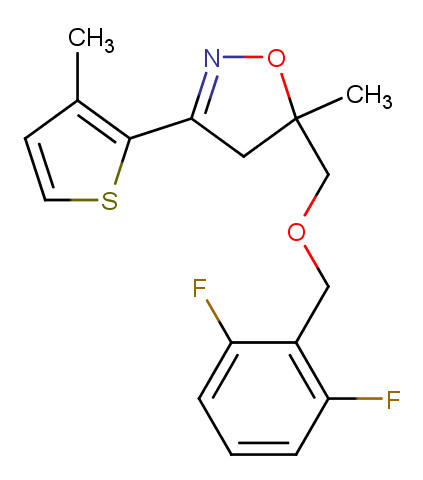

Supplement: RA-011-D1RA00914A-s216 [file RA-011-D1RA00914A-s216.png]

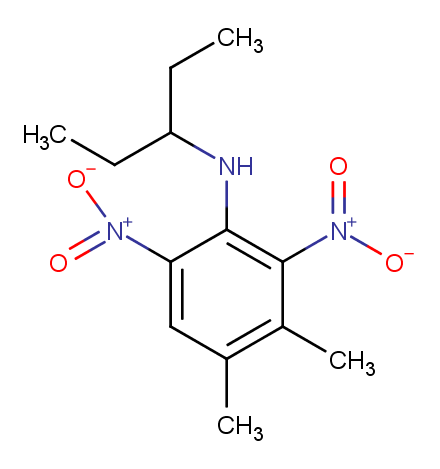

Supplement: RA-011-D1RA00914A-s217 [file RA-011-D1RA00914A-s217.png]

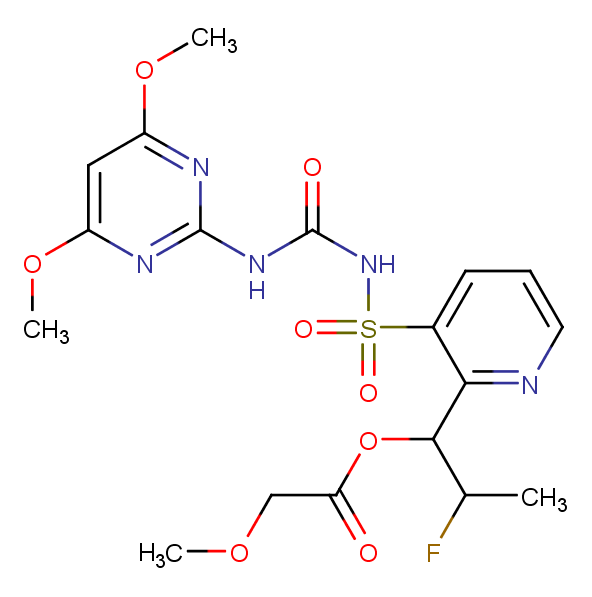

Supplement: RA-011-D1RA00914A-s218 [file RA-011-D1RA00914A-s218.png]

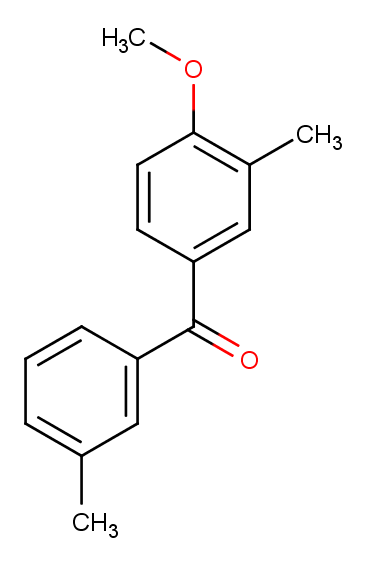

Supplement: RA-011-D1RA00914A-s219 [file RA-011-D1RA00914A-s219.png]

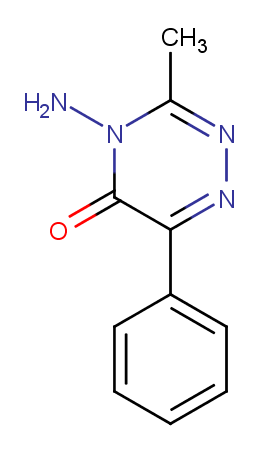

Supplement: RA-011-D1RA00914A-s220 [file RA-011-D1RA00914A-s220.png]

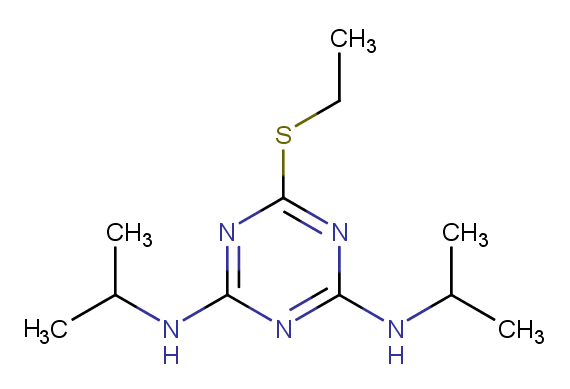

Supplement: RA-011-D1RA00914A-s221 [file RA-011-D1RA00914A-s221.png]

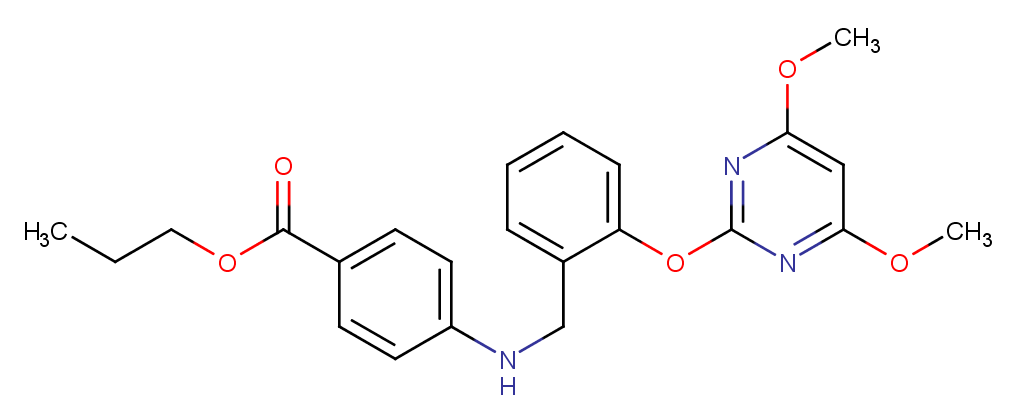

Supplement: RA-011-D1RA00914A-s222 [file RA-011-D1RA00914A-s222.png]

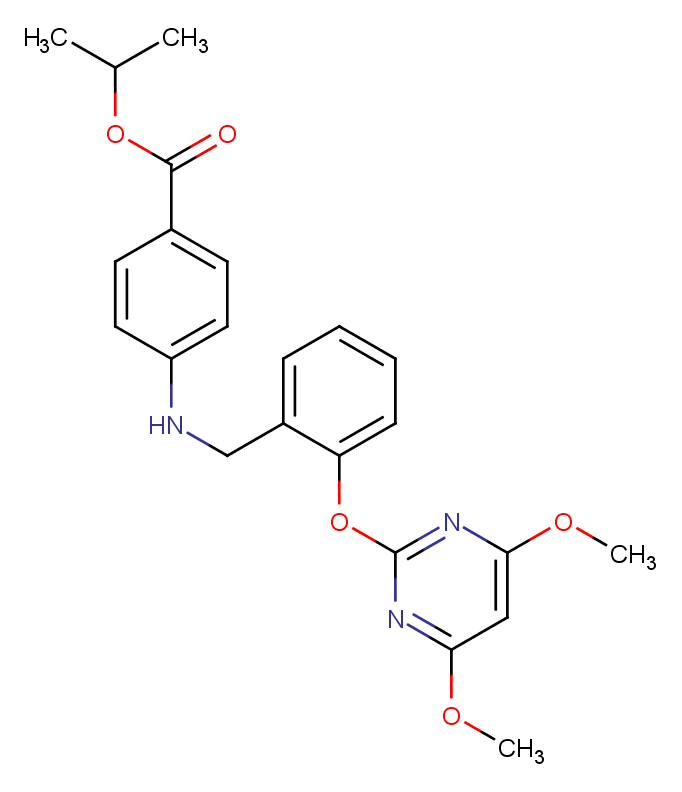

Supplement: RA-011-D1RA00914A-s223 [file RA-011-D1RA00914A-s223.png]

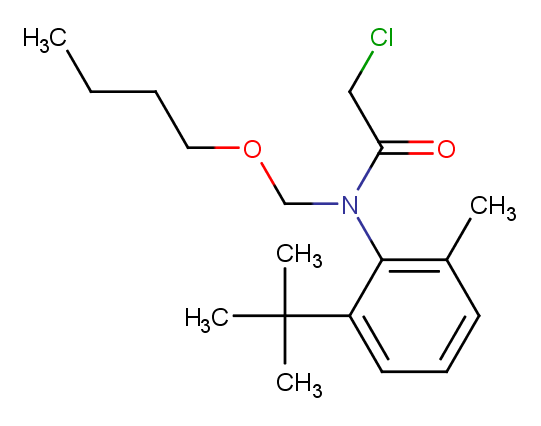

Supplement: RA-011-D1RA00914A-s224 [file RA-011-D1RA00914A-s224.png]

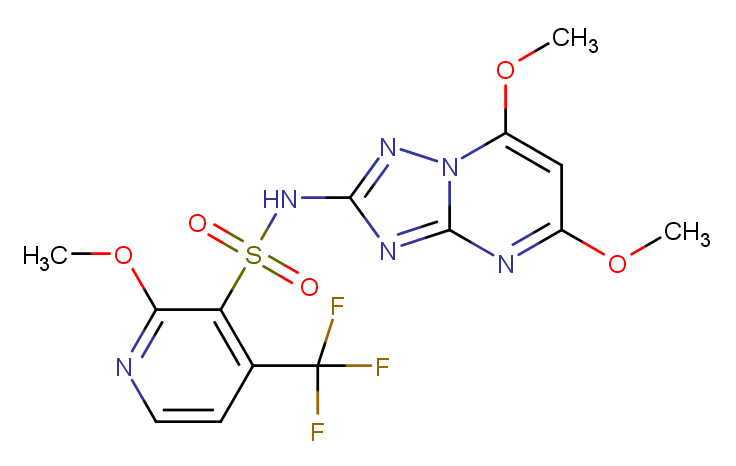

Supplement: RA-011-D1RA00914A-s225 [file RA-011-D1RA00914A-s225.png]

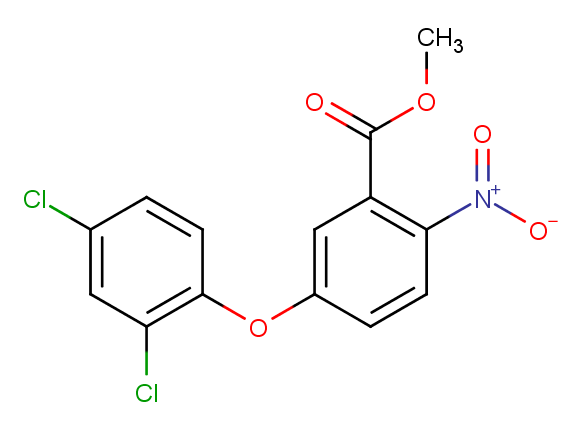

Supplement: RA-011-D1RA00914A-s226 [file RA-011-D1RA00914A-s226.png]

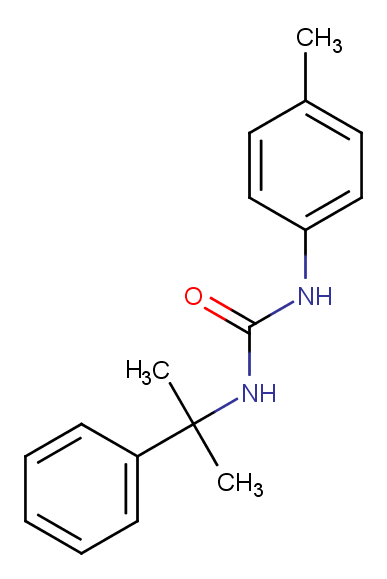

Supplement: RA-011-D1RA00914A-s227 [file RA-011-D1RA00914A-s227.png]

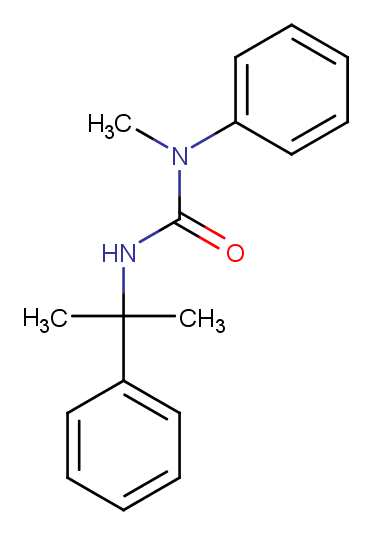

Supplement: RA-011-D1RA00914A-s228 [file RA-011-D1RA00914A-s228.png]

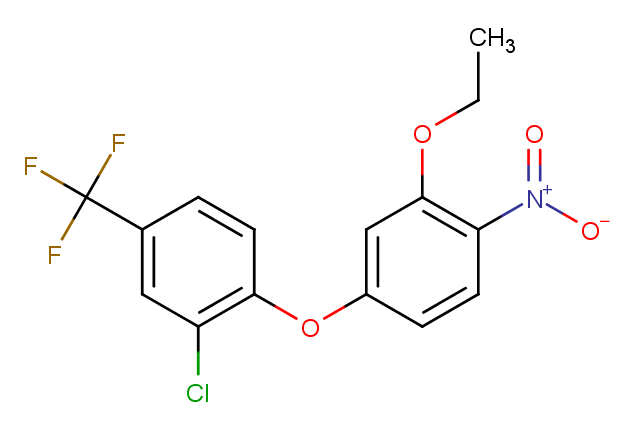

Supplement: RA-011-D1RA00914A-s229 [file RA-011-D1RA00914A-s229.png]

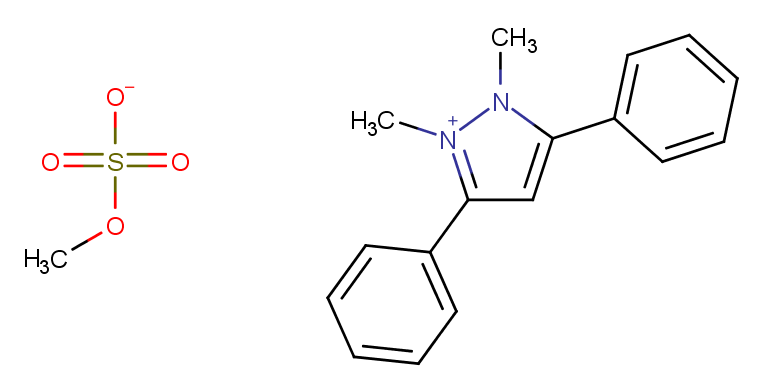

Supplement: RA-011-D1RA00914A-s230 [file RA-011-D1RA00914A-s230.png]

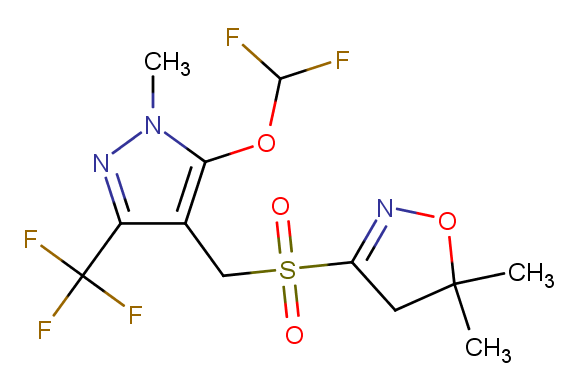

Supplement: RA-011-D1RA00914A-s231 [file RA-011-D1RA00914A-s231.png]

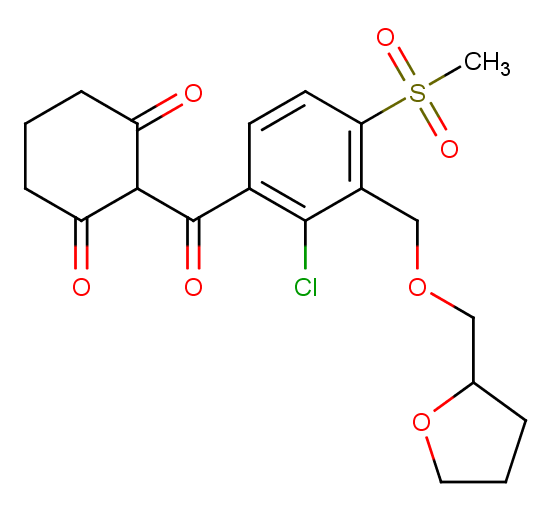

Supplement: RA-011-D1RA00914A-s232 [file RA-011-D1RA00914A-s232.png]

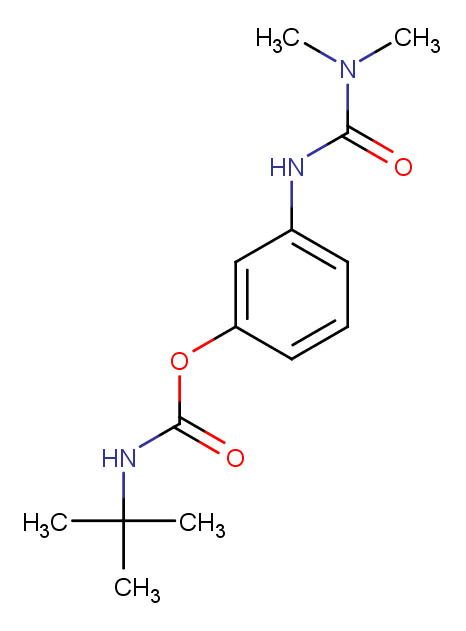

Supplement: RA-011-D1RA00914A-s233 [file RA-011-D1RA00914A-s233.png]

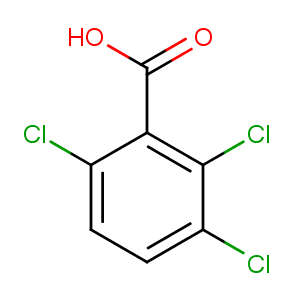

Supplement: RA-011-D1RA00914A-s235 [file RA-011-D1RA00914A-s235.png]

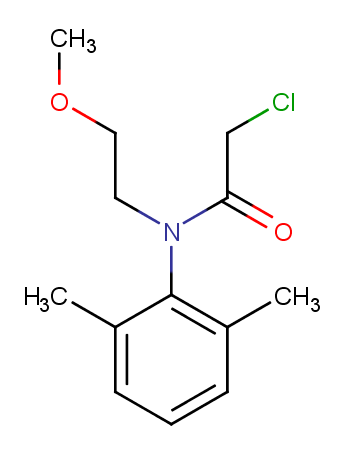

Supplement: RA-011-D1RA00914A-s236 [file RA-011-D1RA00914A-s236.png]

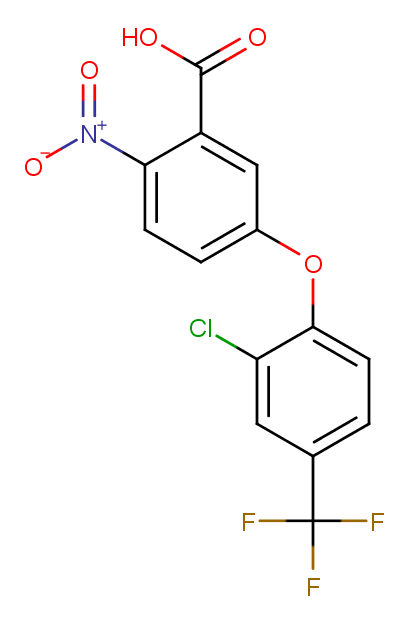

Supplement: RA-011-D1RA00914A-s237 [file RA-011-D1RA00914A-s237.png]

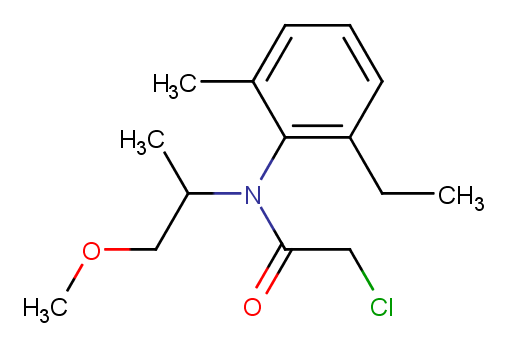

Supplement: RA-011-D1RA00914A-s238 [file RA-011-D1RA00914A-s238.png]

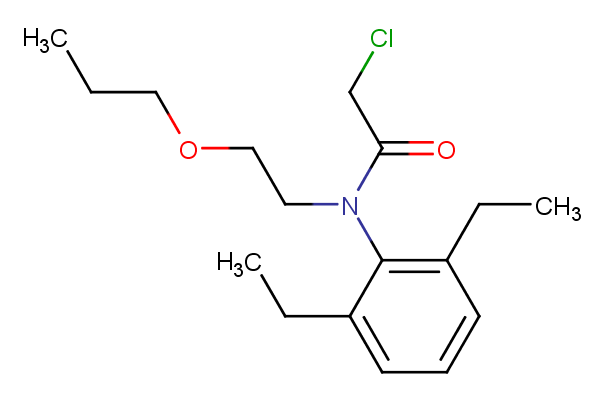

Supplement: RA-011-D1RA00914A-s239 [file RA-011-D1RA00914A-s239.png]

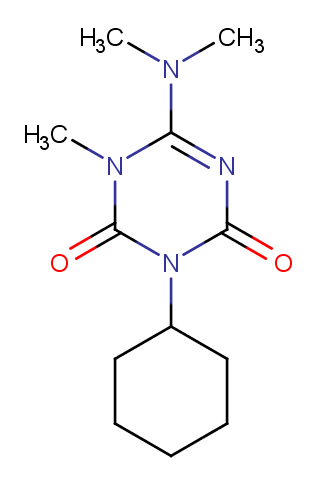

Supplement: RA-011-D1RA00914A-s240 [file RA-011-D1RA00914A-s240.png]

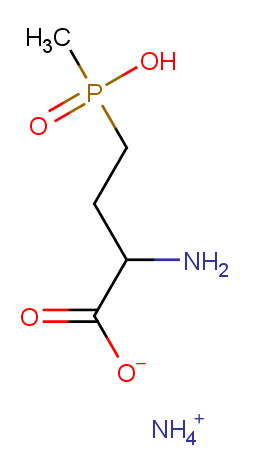

Supplement: RA-011-D1RA00914A-s241 [file RA-011-D1RA00914A-s241.png]

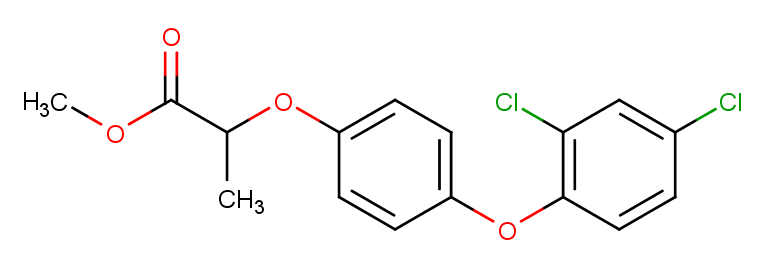

Supplement: RA-011-D1RA00914A-s242 [file RA-011-D1RA00914A-s242.png]

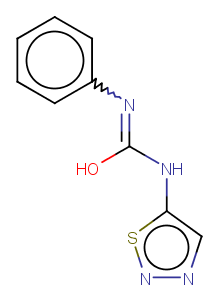

Supplement: RA-011-D1RA00914A-s243 [file RA-011-D1RA00914A-s243.png]

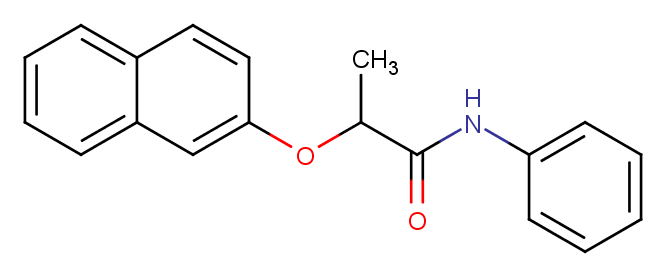

Supplement: RA-011-D1RA00914A-s244 [file RA-011-D1RA00914A-s244.png]

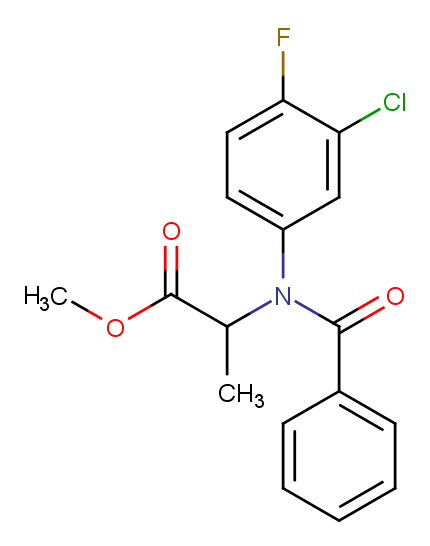

Supplement: RA-011-D1RA00914A-s245 [file RA-011-D1RA00914A-s245.png]

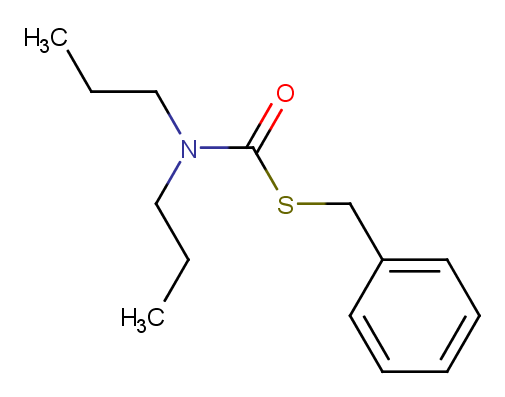

Supplement: RA-011-D1RA00914A-s246 [file RA-011-D1RA00914A-s246.png]

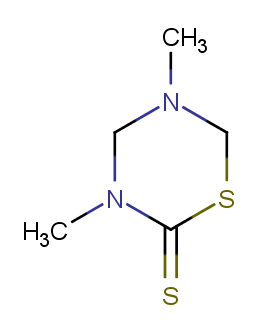

Supplement: RA-011-D1RA00914A-s247 [file RA-011-D1RA00914A-s247.png]

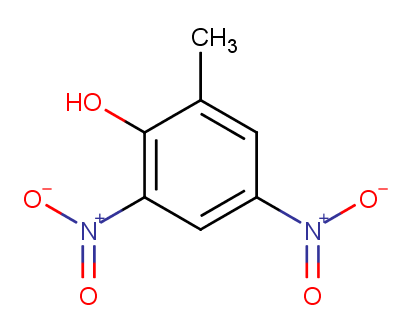

Supplement: RA-011-D1RA00914A-s248 [file RA-011-D1RA00914A-s248.png]

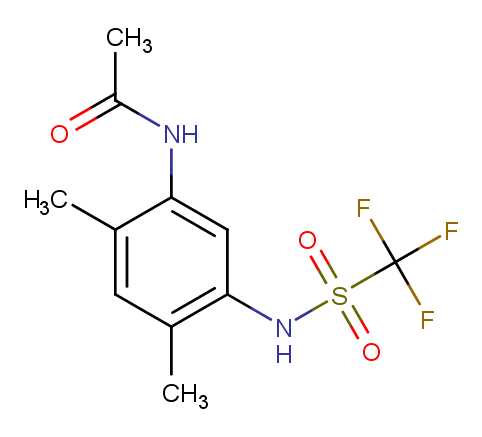

Supplement: RA-011-D1RA00914A-s249 [file RA-011-D1RA00914A-s249.png]

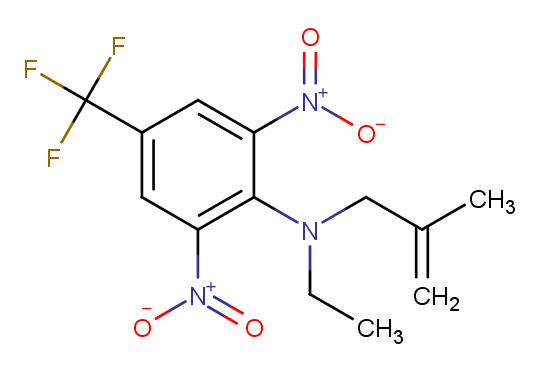

Supplement: RA-011-D1RA00914A-s250 [file RA-011-D1RA00914A-s250.png]

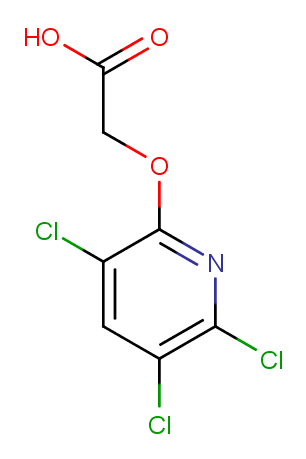

Supplement: RA-011-D1RA00914A-s251 [file RA-011-D1RA00914A-s251.png]

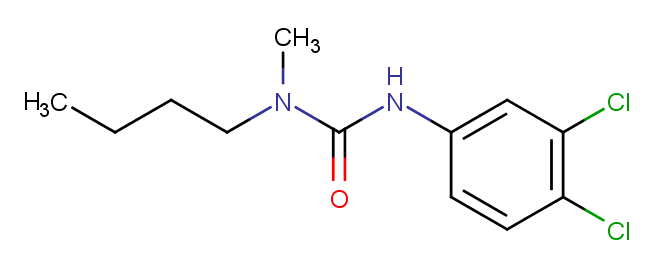

Supplement: RA-011-D1RA00914A-s252 [file RA-011-D1RA00914A-s252.png]

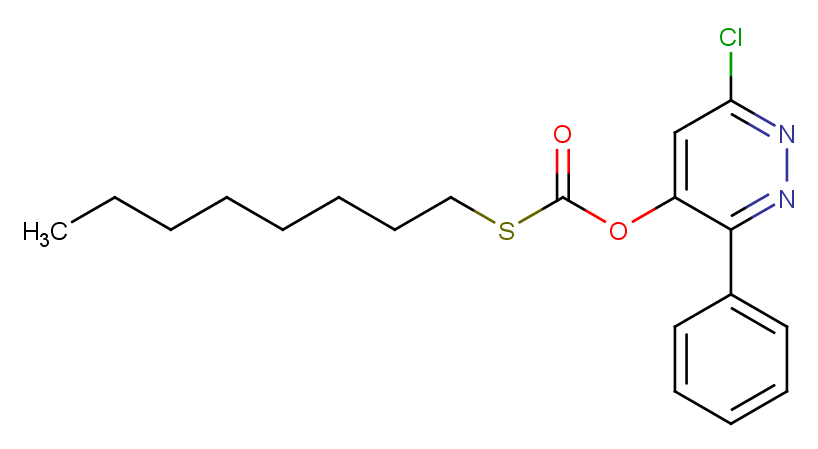

Supplement: RA-011-D1RA00914A-s253 [file RA-011-D1RA00914A-s253.png]

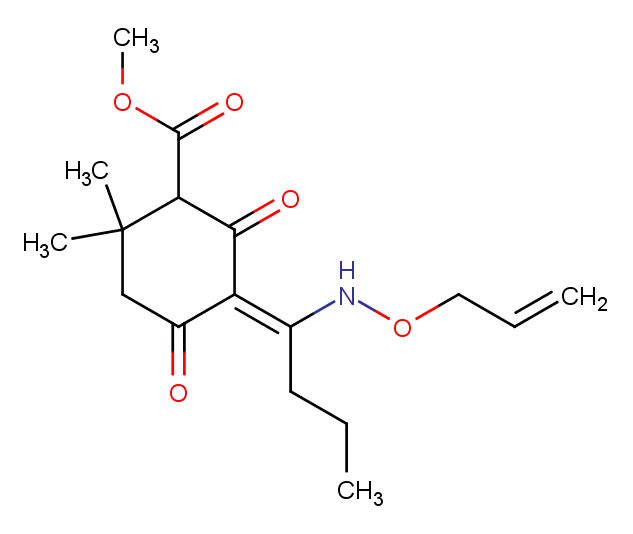

Supplement: RA-011-D1RA00914A-s254 [file RA-011-D1RA00914A-s254.png]

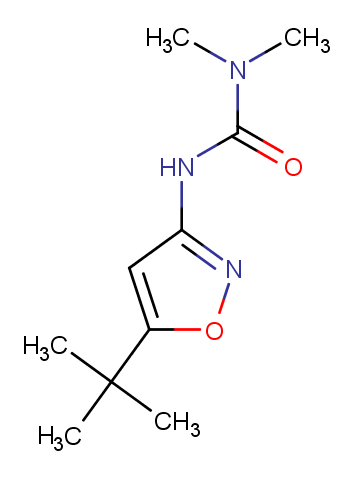

Supplement: RA-011-D1RA00914A-s255 [file RA-011-D1RA00914A-s255.png]

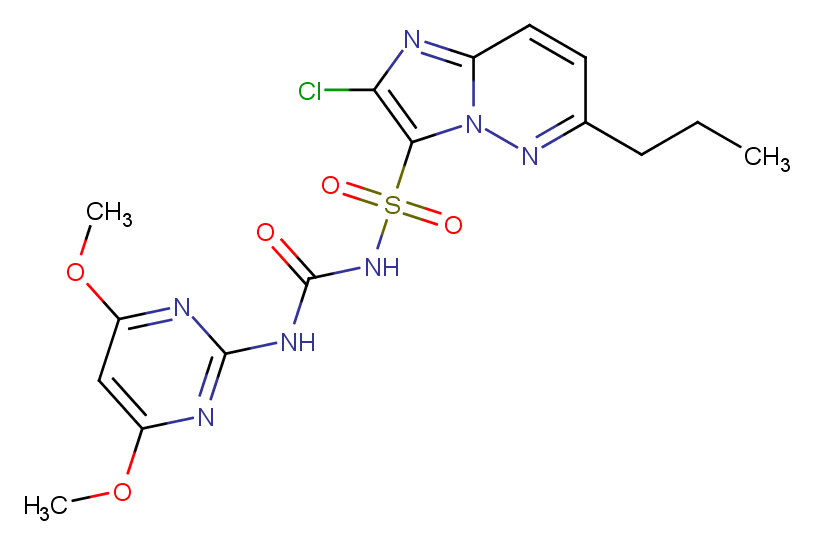

Supplement: RA-011-D1RA00914A-s256 [file RA-011-D1RA00914A-s256.png]

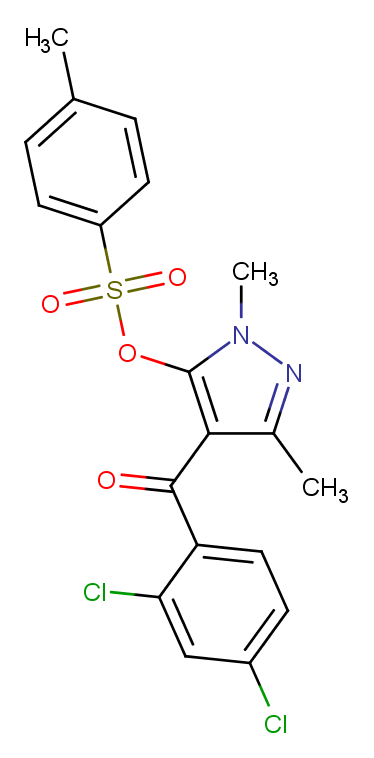

Supplement: RA-011-D1RA00914A-s257 [file RA-011-D1RA00914A-s257.png]

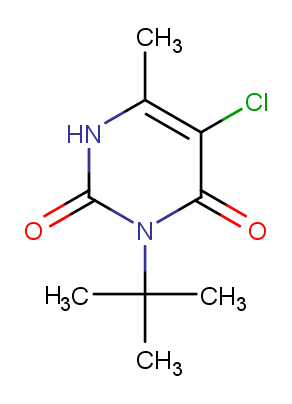

Supplement: RA-011-D1RA00914A-s258 [file RA-011-D1RA00914A-s258.png]

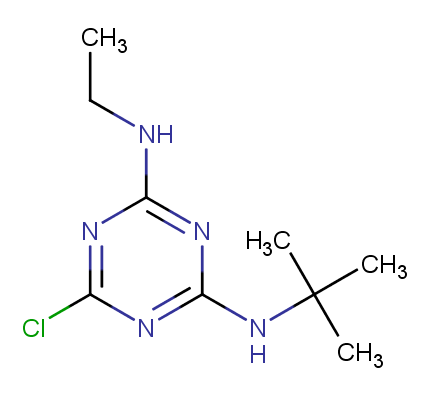

Supplement: RA-011-D1RA00914A-s259 [file RA-011-D1RA00914A-s259.png]

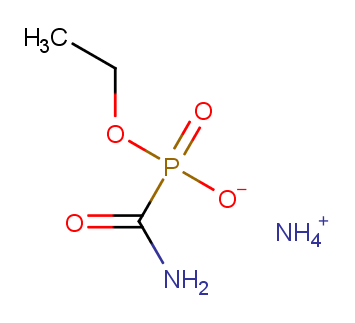

Supplement: RA-011-D1RA00914A-s260 [file RA-011-D1RA00914A-s260.png]

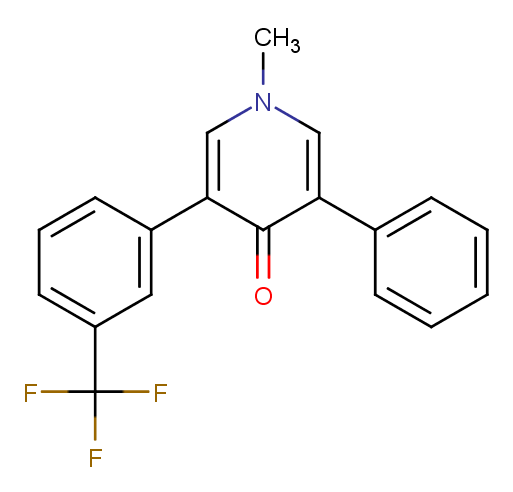

Supplement: RA-011-D1RA00914A-s261 [file RA-011-D1RA00914A-s261.png]

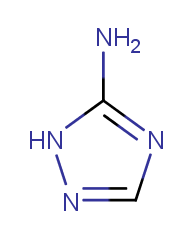

Supplement: RA-011-D1RA00914A-s262 [file RA-011-D1RA00914A-s262.png]

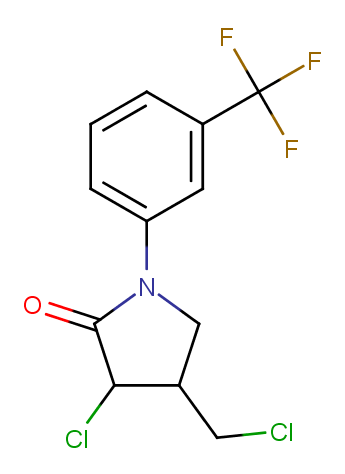

Supplement: RA-011-D1RA00914A-s263 [file RA-011-D1RA00914A-s263.png]

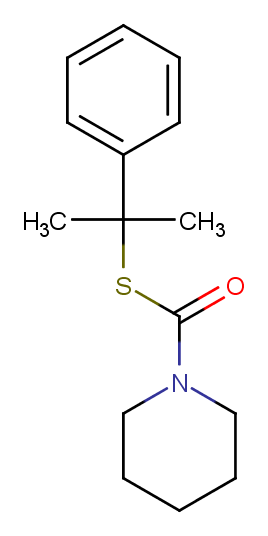

Supplement: RA-011-D1RA00914A-s264 [file RA-011-D1RA00914A-s264.png]

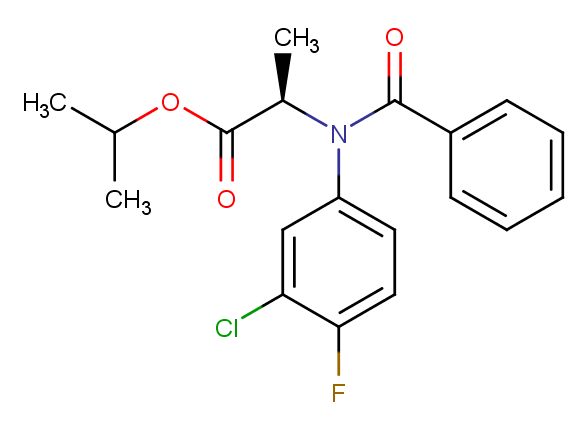

Supplement: RA-011-D1RA00914A-s265 [file RA-011-D1RA00914A-s265.png]

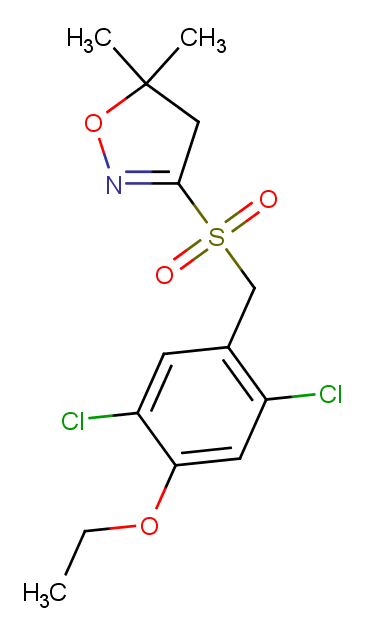

Supplement: RA-011-D1RA00914A-s266 [file RA-011-D1RA00914A-s266.png]

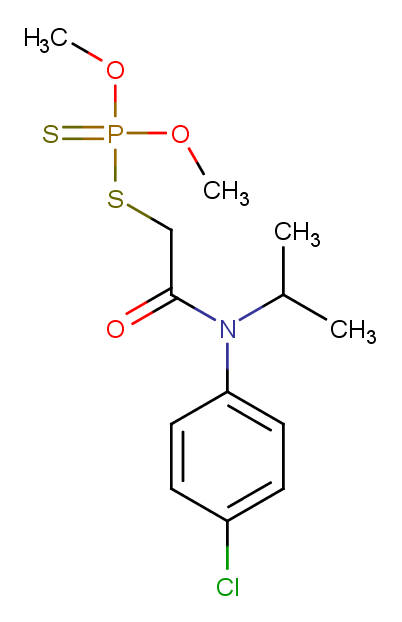

Supplement: RA-011-D1RA00914A-s267 [file RA-011-D1RA00914A-s267.png]

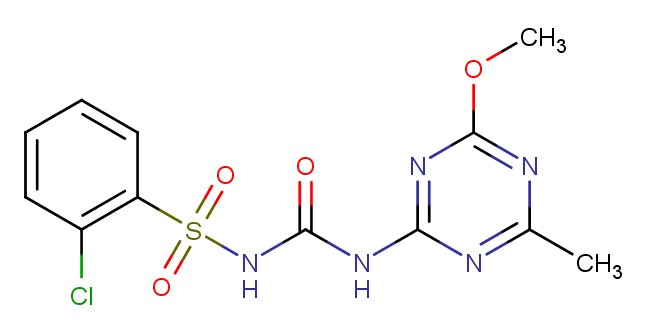

Supplement: RA-011-D1RA00914A-s268 [file RA-011-D1RA00914A-s268.png]

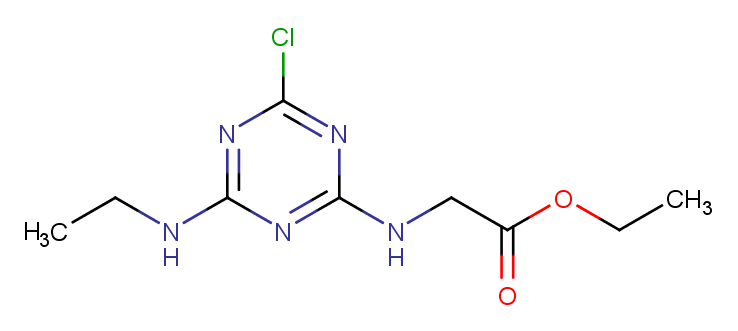

Supplement: RA-011-D1RA00914A-s269 [file RA-011-D1RA00914A-s269.png]

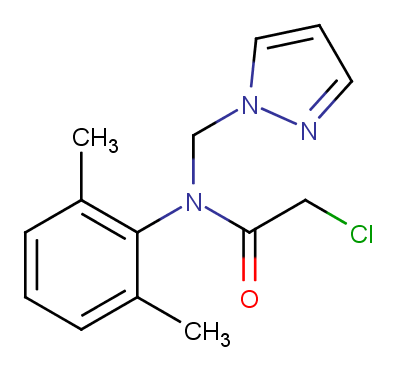

Supplement: RA-011-D1RA00914A-s270 [file RA-011-D1RA00914A-s270.png]

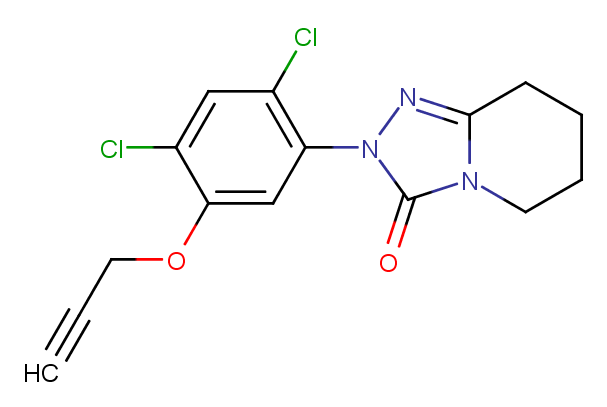

Supplement: RA-011-D1RA00914A-s271 [file RA-011-D1RA00914A-s271.png]

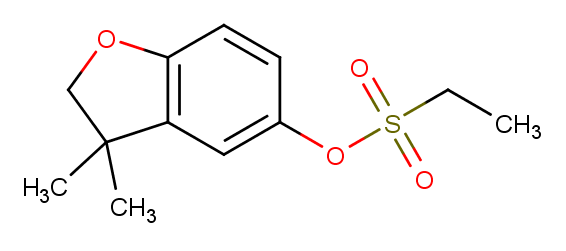

Supplement: RA-011-D1RA00914A-s272 [file RA-011-D1RA00914A-s272.png]

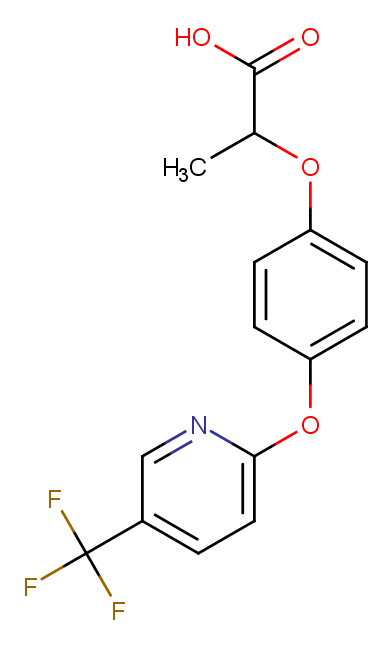

Supplement: RA-011-D1RA00914A-s274 [file RA-011-D1RA00914A-s274.png]

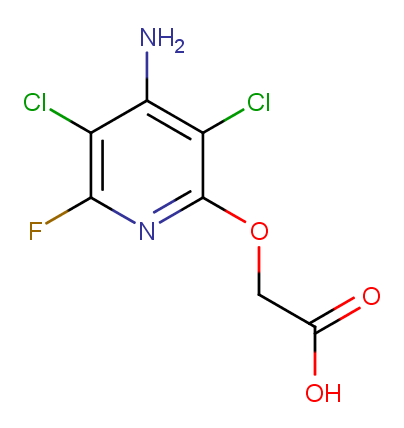

Supplement: RA-011-D1RA00914A-s275 [file RA-011-D1RA00914A-s275.png]

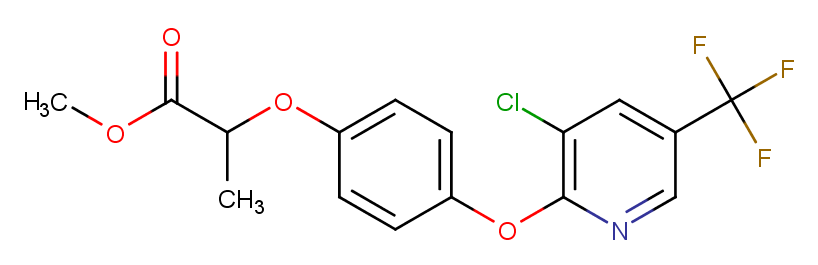

Supplement: RA-011-D1RA00914A-s276 [file RA-011-D1RA00914A-s276.png]

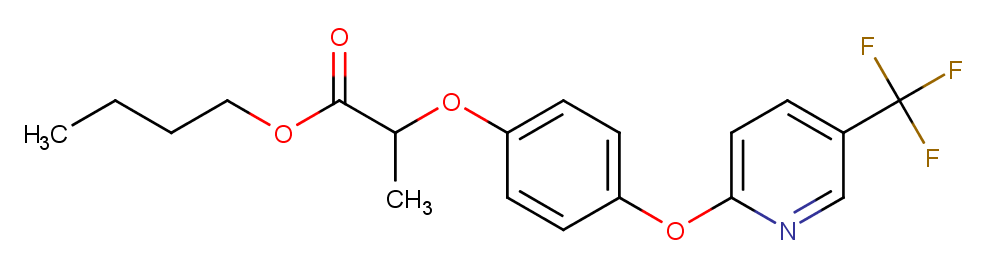

Supplement: RA-011-D1RA00914A-s277 [file RA-011-D1RA00914A-s277.png]

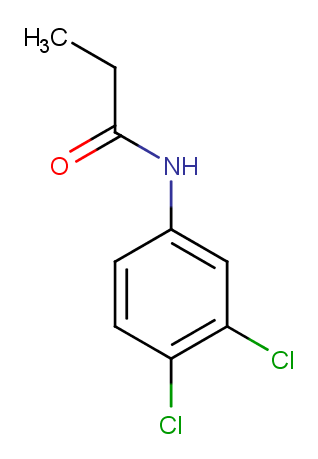

Supplement: RA-011-D1RA00914A-s278 [file RA-011-D1RA00914A-s278.png]

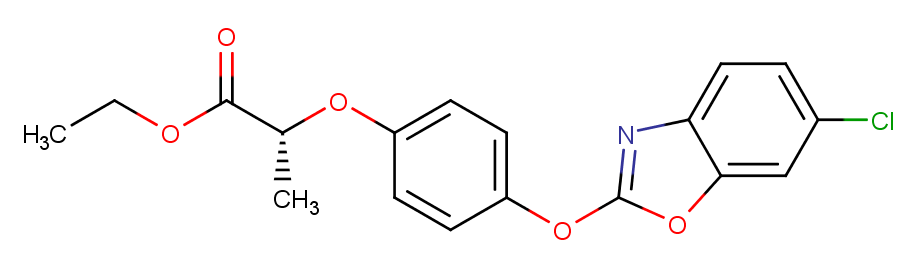

Supplement: RA-011-D1RA00914A-s279 [file RA-011-D1RA00914A-s279.png]

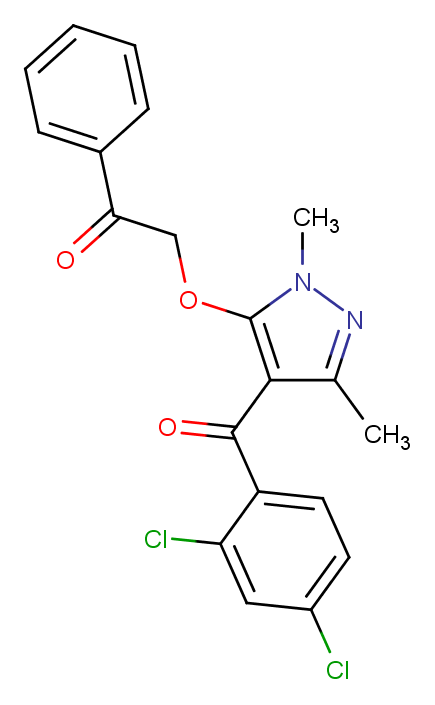

Supplement: RA-011-D1RA00914A-s280 [file RA-011-D1RA00914A-s280.png]

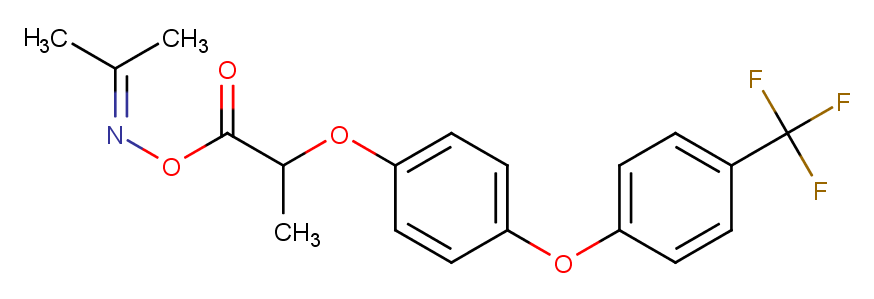

Supplement: RA-011-D1RA00914A-s281 [file RA-011-D1RA00914A-s281.png]

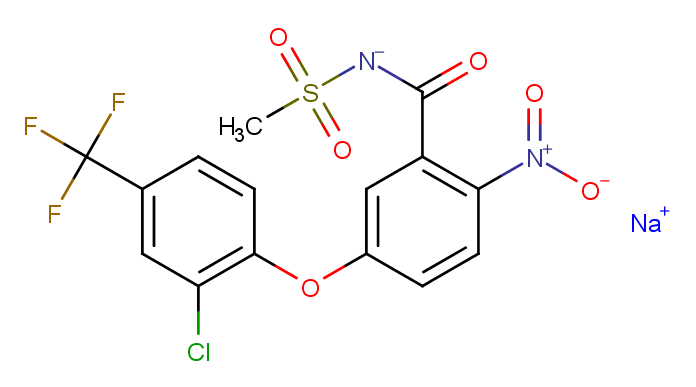

Supplement: RA-011-D1RA00914A-s282 [file RA-011-D1RA00914A-s282.png]

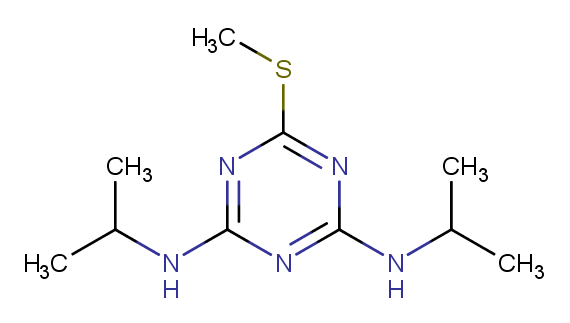

Supplement: RA-011-D1RA00914A-s283 [file RA-011-D1RA00914A-s283.png]

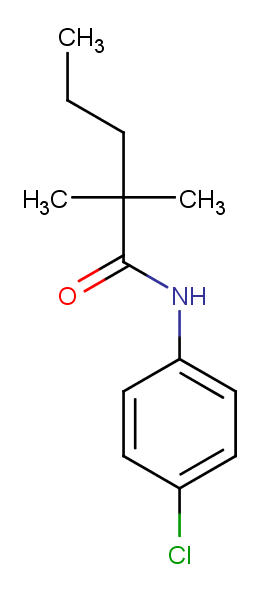

Supplement: RA-011-D1RA00914A-s284 [file RA-011-D1RA00914A-s284.png]

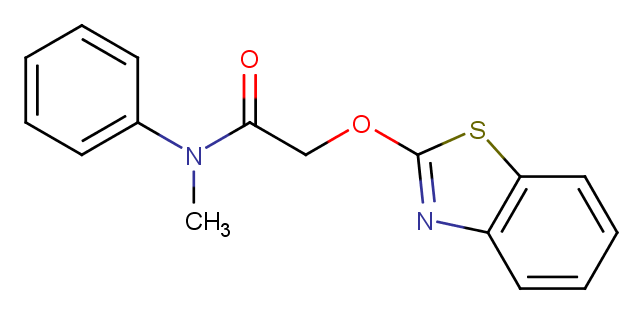

Supplement: RA-011-D1RA00914A-s285 [file RA-011-D1RA00914A-s285.png]

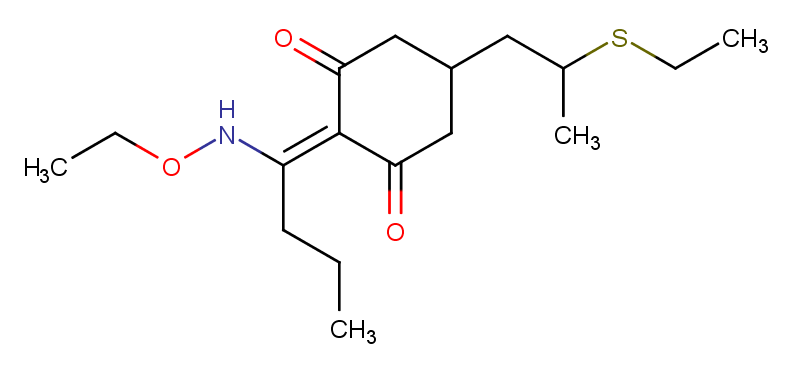

Supplement: RA-011-D1RA00914A-s286 [file RA-011-D1RA00914A-s286.png]

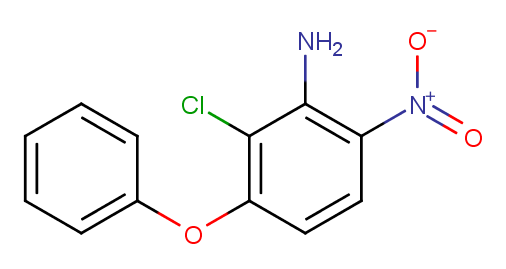

Supplement: RA-011-D1RA00914A-s287 [file RA-011-D1RA00914A-s287.png]

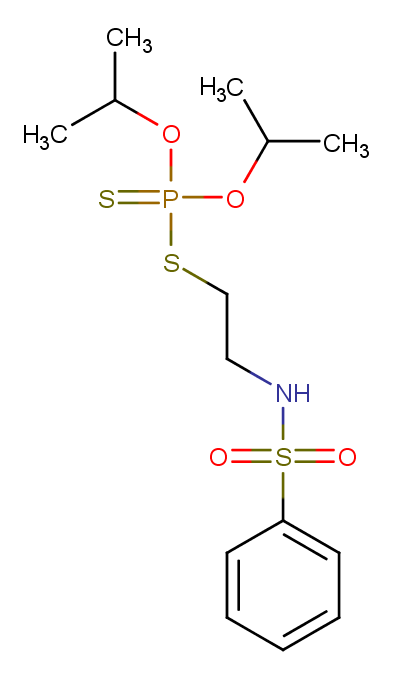

Supplement: RA-011-D1RA00914A-s288 [file RA-011-D1RA00914A-s288.png]

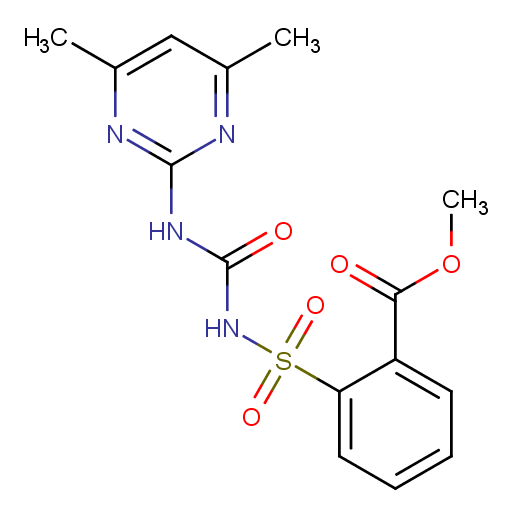

Supplement: RA-011-D1RA00914A-s289 [file RA-011-D1RA00914A-s289.png]

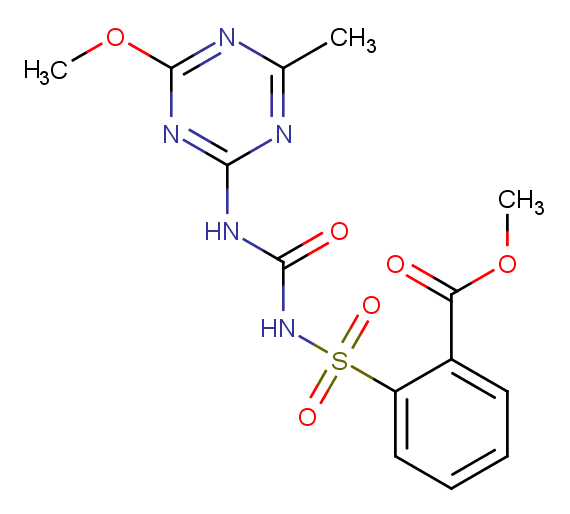

Supplement: RA-011-D1RA00914A-s290 [file RA-011-D1RA00914A-s290.png]

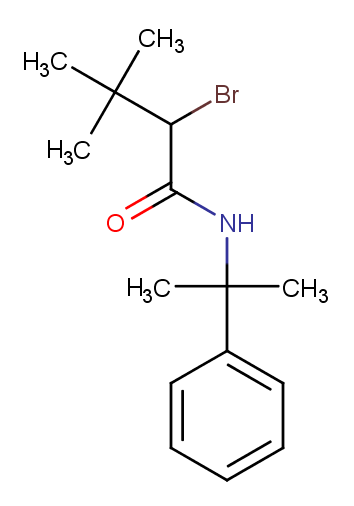

Supplement: RA-011-D1RA00914A-s291 [file RA-011-D1RA00914A-s291.png]

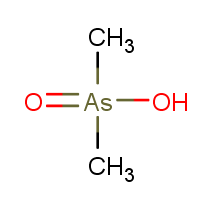

Supplement: RA-011-D1RA00914A-s292 [file RA-011-D1RA00914A-s292.png]

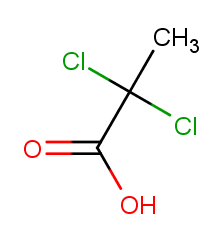

Supplement: RA-011-D1RA00914A-s293 [file RA-011-D1RA00914A-s293.png]

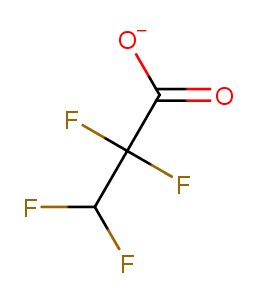

Supplement: RA-011-D1RA00914A-s294 [file RA-011-D1RA00914A-s294.png]

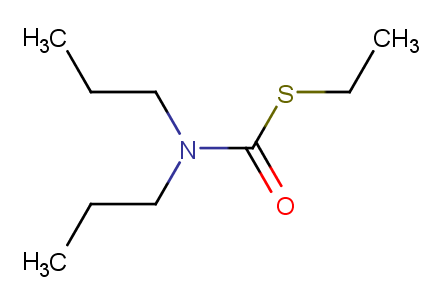

Supplement: RA-011-D1RA00914A-s295 [file RA-011-D1RA00914A-s295.png]

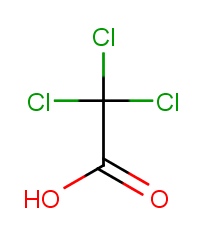

Supplement: RA-011-D1RA00914A-s296 [file RA-011-D1RA00914A-s296.png]

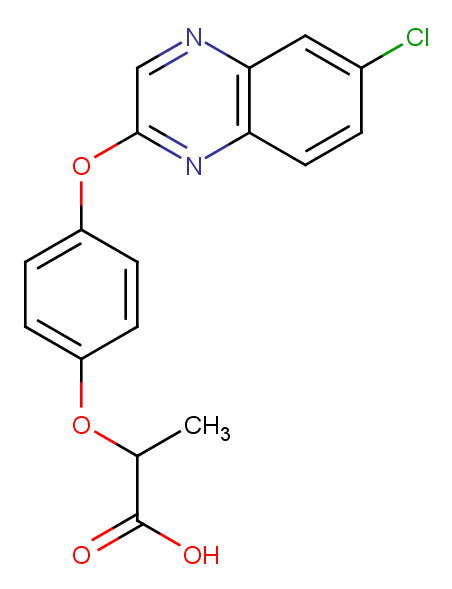

Supplement: RA-011-D1RA00914A-s297 [file RA-011-D1RA00914A-s297.png]

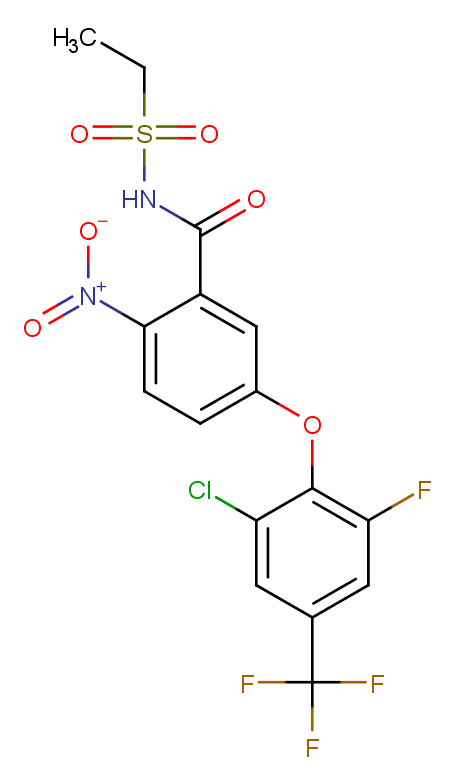

Supplement: RA-011-D1RA00914A-s298 [file RA-011-D1RA00914A-s298.png]

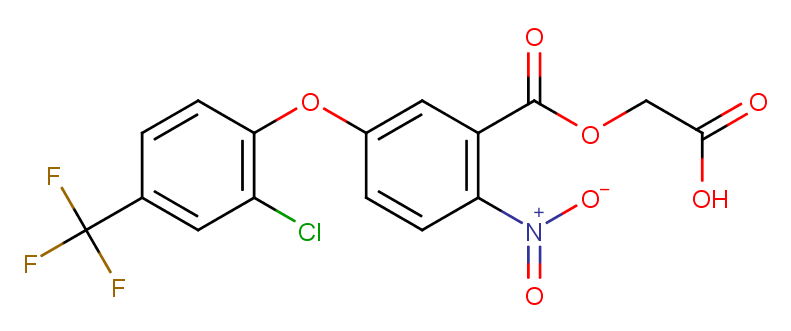

Supplement: RA-011-D1RA00914A-s299 [file RA-011-D1RA00914A-s299.png]

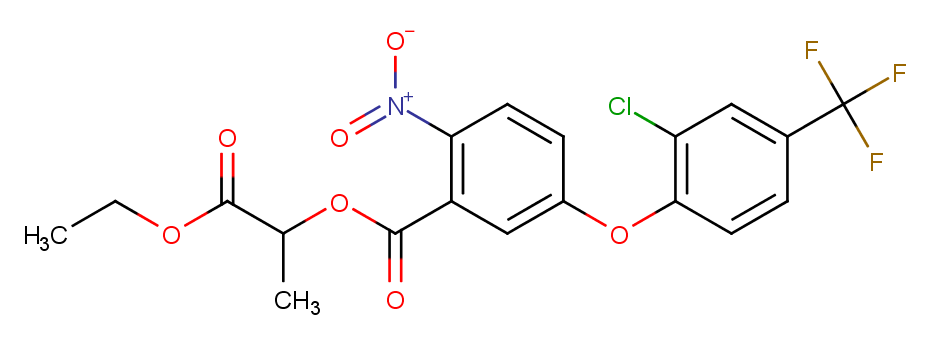

Supplement: RA-011-D1RA00914A-s300 [file RA-011-D1RA00914A-s300.png]

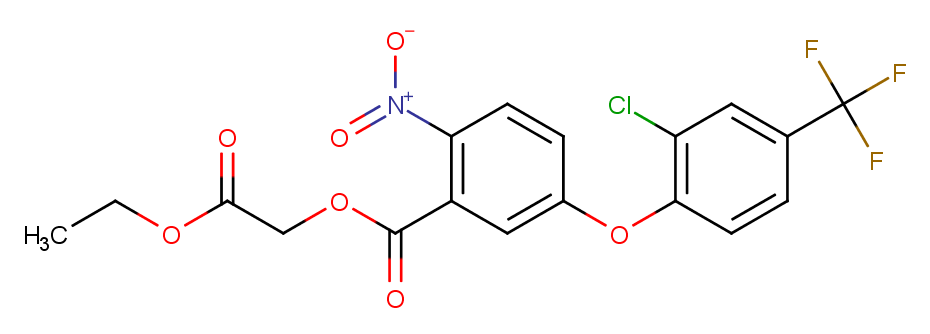

Supplement: RA-011-D1RA00914A-s301 [file RA-011-D1RA00914A-s301.png]

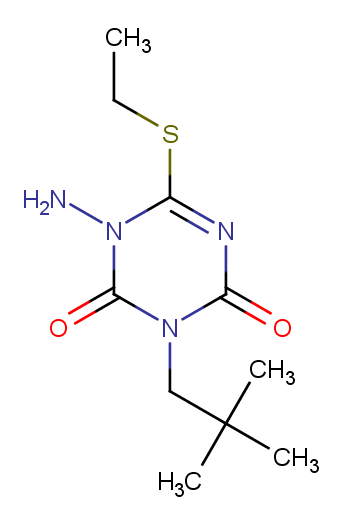

Supplement: RA-011-D1RA00914A-s302 [file RA-011-D1RA00914A-s302.png]

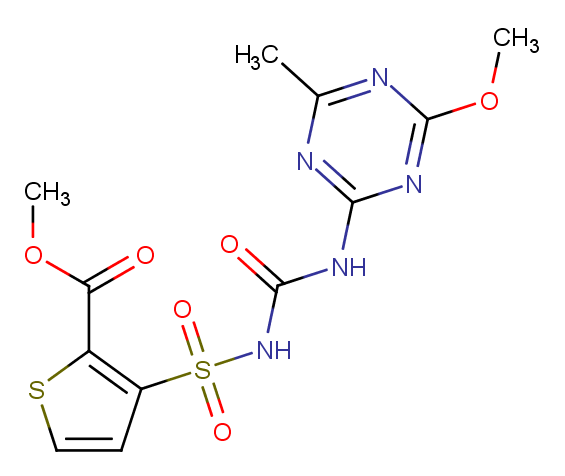

Supplement: RA-011-D1RA00914A-s303 [file RA-011-D1RA00914A-s303.png]

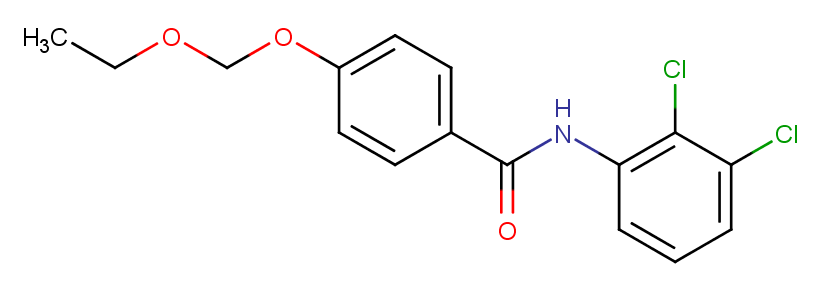

Supplement: RA-011-D1RA00914A-s304 [file RA-011-D1RA00914A-s304.png]
