# Supplementary figures and images for: Improved herbicide discovery using physico-chemical rules refined by antimalarial library screening (part 4 of 14)
Source: RSC Adv. 2021 Feb 23;11(15):8459–67. doi: 10.1039/d1ra00914a (PMC8695207; doi:10.1039/d1ra00914a)

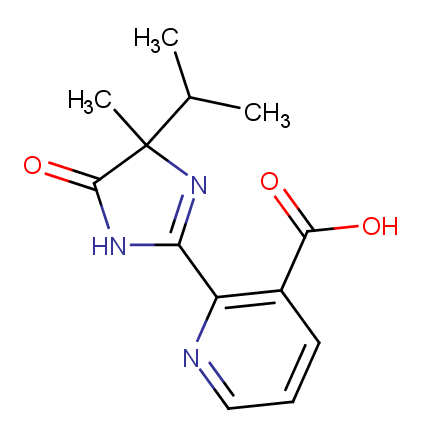

Supplement: RA-011-D1RA00914A-s305 [file RA-011-D1RA00914A-s305.png]

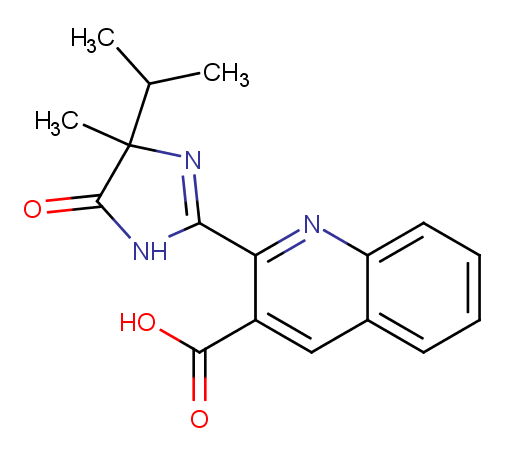

Supplement: RA-011-D1RA00914A-s306 [file RA-011-D1RA00914A-s306.png]

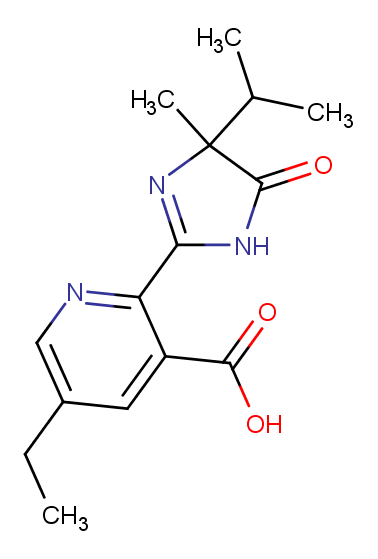

Supplement: RA-011-D1RA00914A-s307 [file RA-011-D1RA00914A-s307.png]

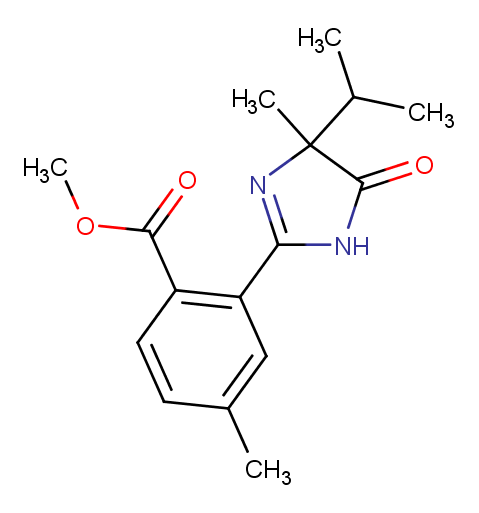

Supplement: RA-011-D1RA00914A-s308 [file RA-011-D1RA00914A-s308.png]

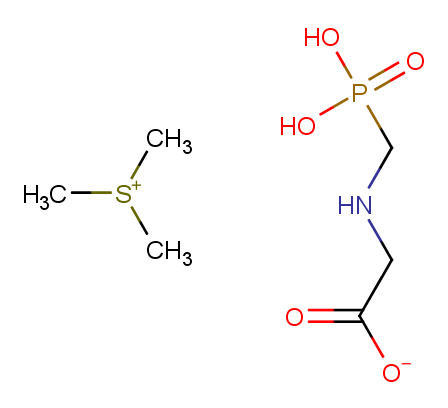

Supplement: RA-011-D1RA00914A-s309 [file RA-011-D1RA00914A-s309.png]

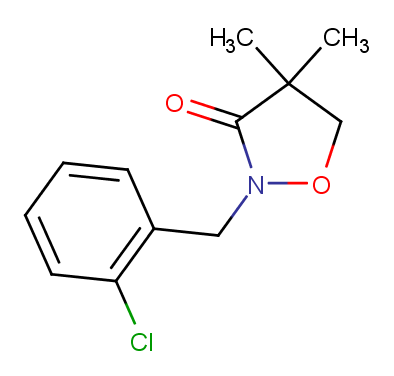

Supplement: RA-011-D1RA00914A-s310 [file RA-011-D1RA00914A-s310.png]

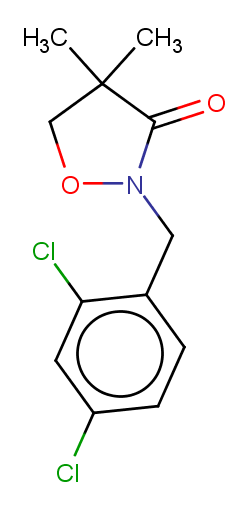

Supplement: RA-011-D1RA00914A-s311 [file RA-011-D1RA00914A-s311.png]

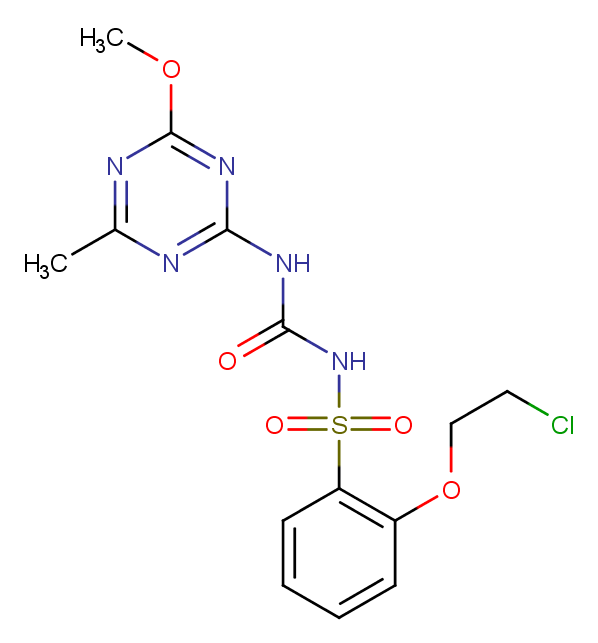

Supplement: RA-011-D1RA00914A-s312 [file RA-011-D1RA00914A-s312.png]

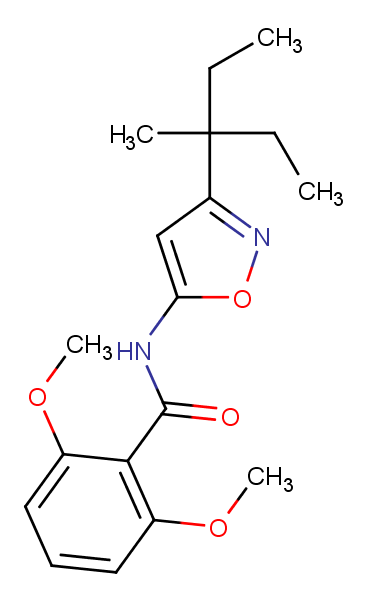

Supplement: RA-011-D1RA00914A-s313 [file RA-011-D1RA00914A-s313.png]

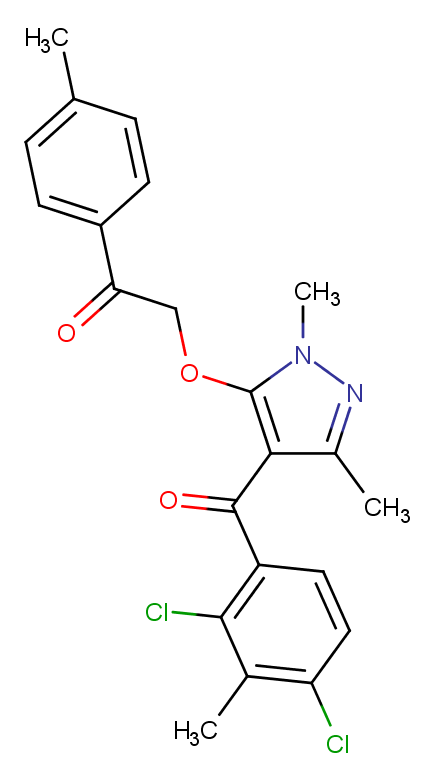

Supplement: RA-011-D1RA00914A-s314 [file RA-011-D1RA00914A-s314.png]

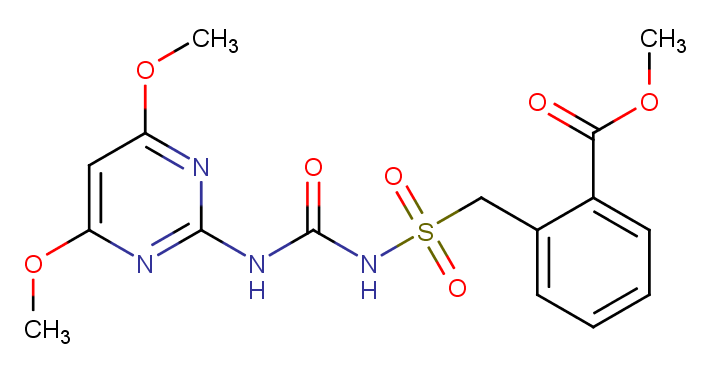

Supplement: RA-011-D1RA00914A-s315 [file RA-011-D1RA00914A-s315.png]

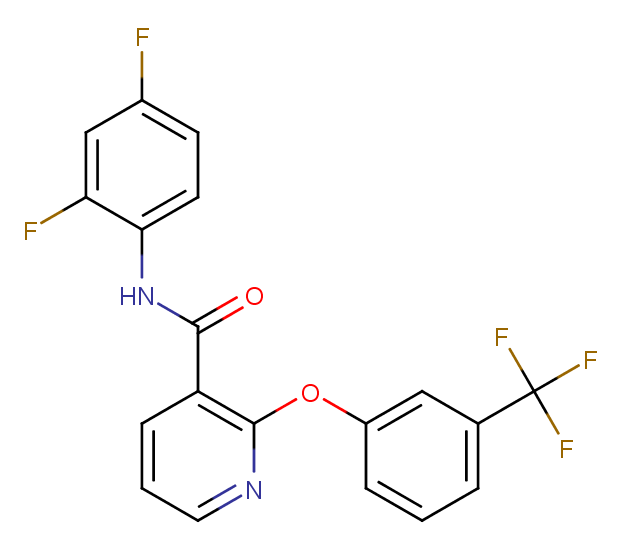

Supplement: RA-011-D1RA00914A-s316 [file RA-011-D1RA00914A-s316.png]

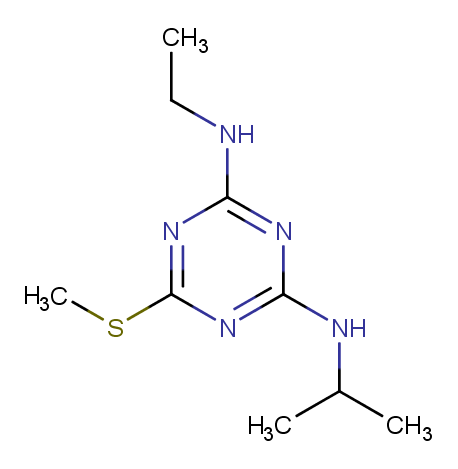

Supplement: RA-011-D1RA00914A-s317 [file RA-011-D1RA00914A-s317.png]

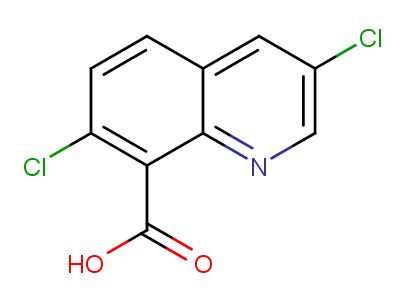

Supplement: RA-011-D1RA00914A-s318 [file RA-011-D1RA00914A-s318.png]

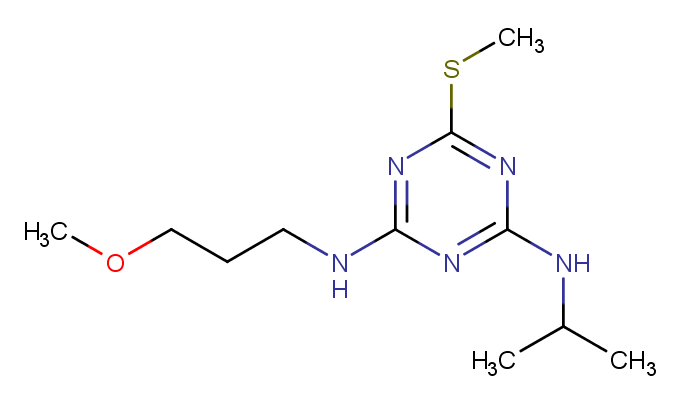

Supplement: RA-011-D1RA00914A-s319 [file RA-011-D1RA00914A-s319.png]

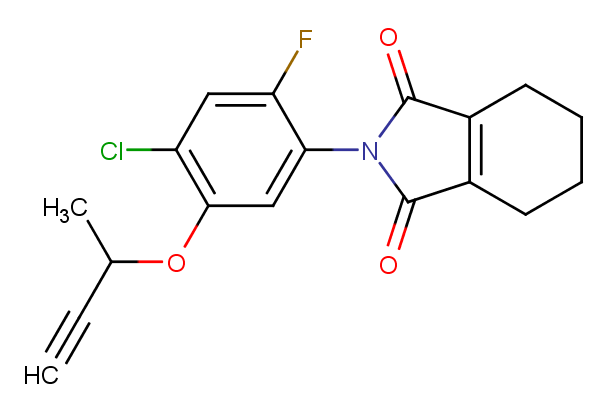

Supplement: RA-011-D1RA00914A-s320 [file RA-011-D1RA00914A-s320.png]

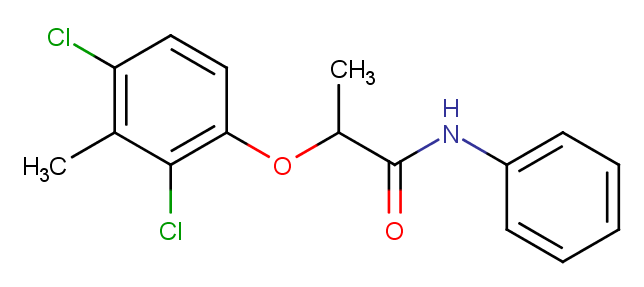

Supplement: RA-011-D1RA00914A-s321 [file RA-011-D1RA00914A-s321.png]

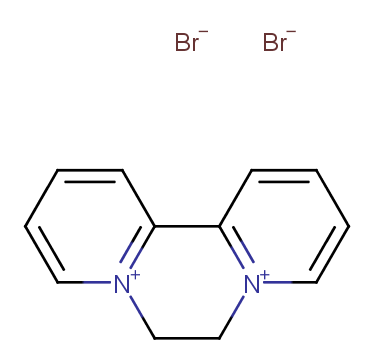

Supplement: RA-011-D1RA00914A-s322 [file RA-011-D1RA00914A-s322.png]

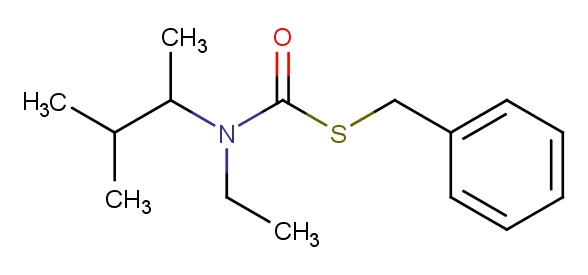

Supplement: RA-011-D1RA00914A-s323 [file RA-011-D1RA00914A-s323.png]

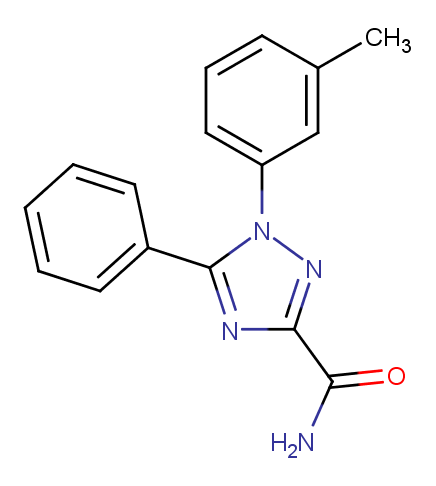

Supplement: RA-011-D1RA00914A-s324 [file RA-011-D1RA00914A-s324.png]

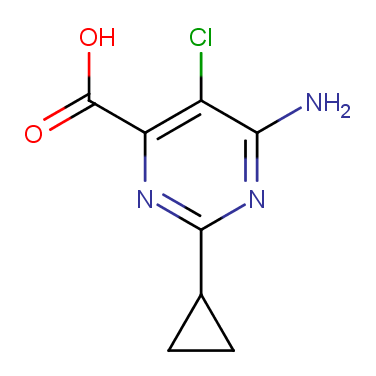

Supplement: RA-011-D1RA00914A-s325 [file RA-011-D1RA00914A-s325.png]

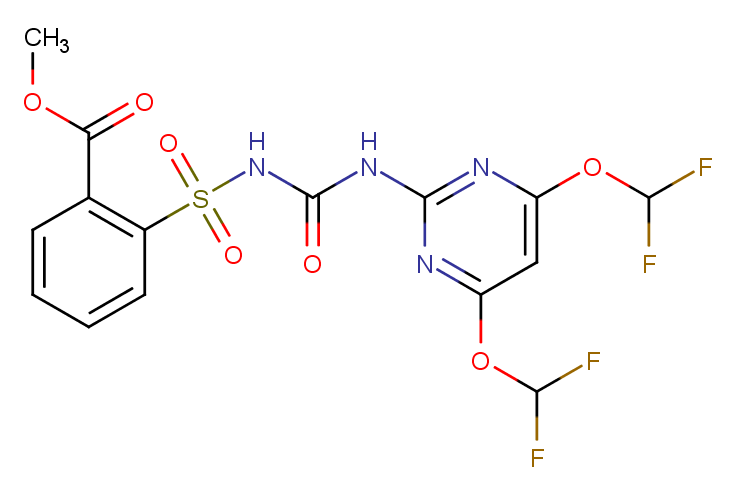

Supplement: RA-011-D1RA00914A-s326 [file RA-011-D1RA00914A-s326.png]

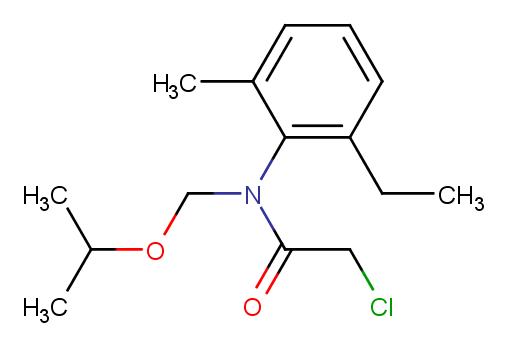

Supplement: RA-011-D1RA00914A-s327 [file RA-011-D1RA00914A-s327.png]

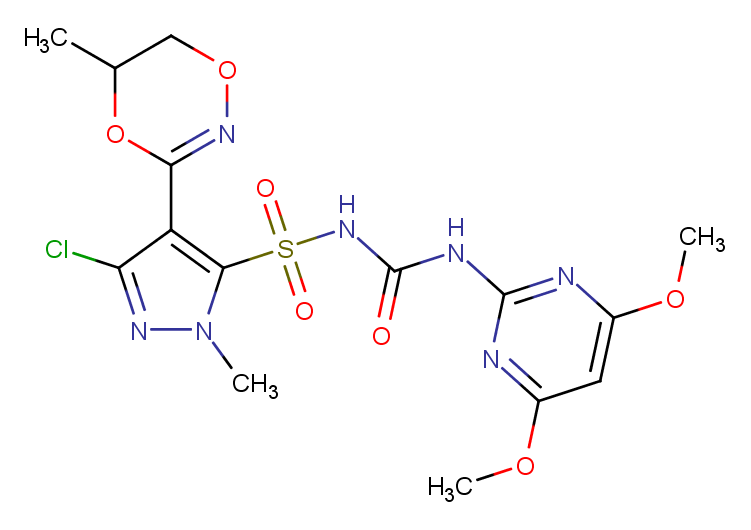

Supplement: RA-011-D1RA00914A-s328 [file RA-011-D1RA00914A-s328.png]

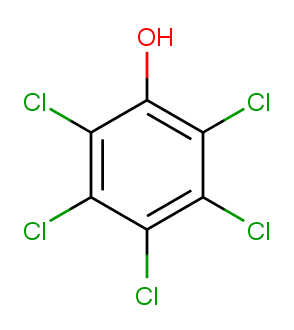

Supplement: RA-011-D1RA00914A-s329 [file RA-011-D1RA00914A-s329.png]

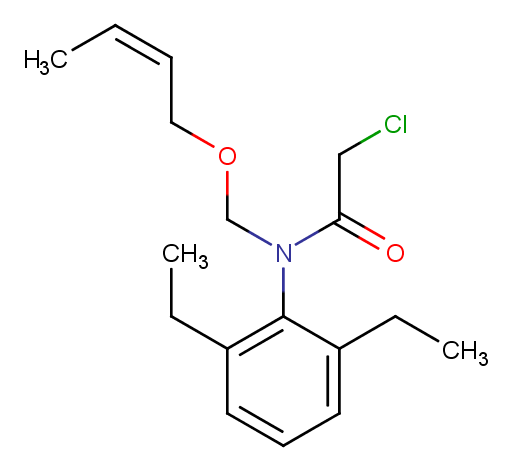

Supplement: RA-011-D1RA00914A-s330 [file RA-011-D1RA00914A-s330.png]

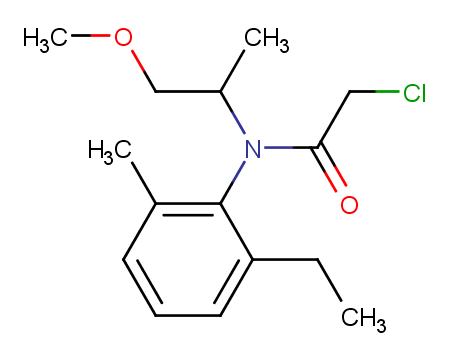

Supplement: RA-011-D1RA00914A-s331 [file RA-011-D1RA00914A-s331.png]

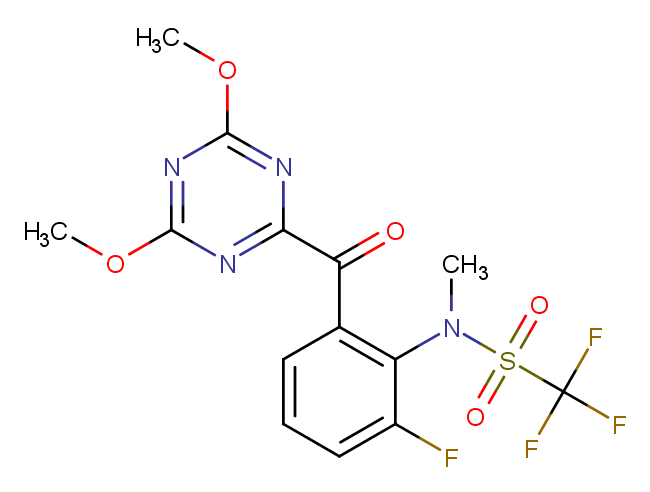

Supplement: RA-011-D1RA00914A-s332 [file RA-011-D1RA00914A-s332.png]

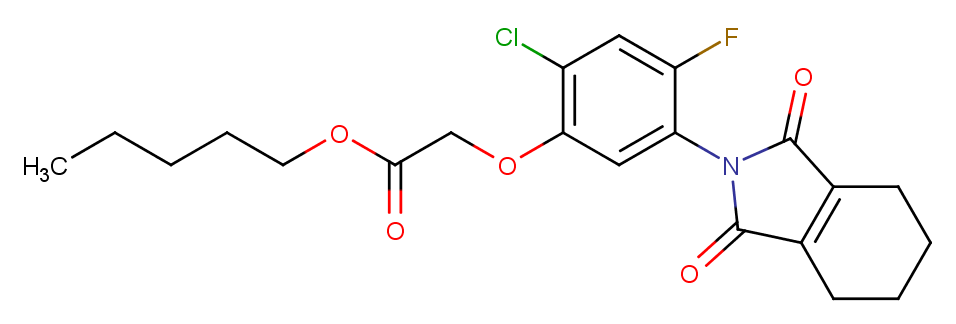

Supplement: RA-011-D1RA00914A-s333 [file RA-011-D1RA00914A-s333.png]

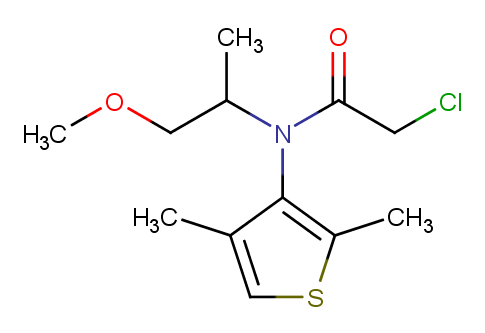

Supplement: RA-011-D1RA00914A-s334 [file RA-011-D1RA00914A-s334.png]

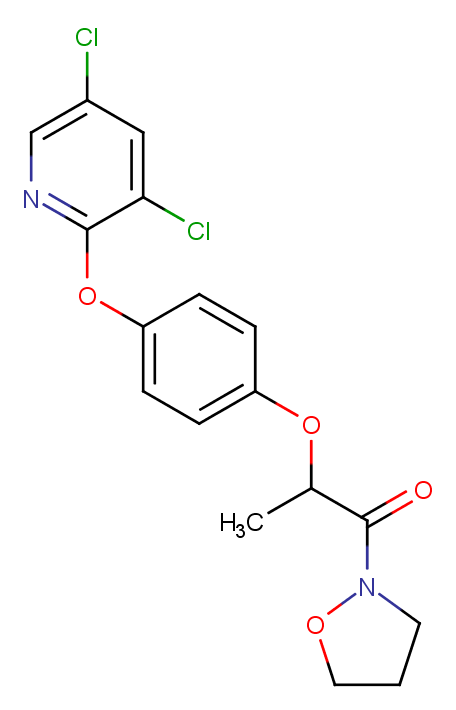

Supplement: RA-011-D1RA00914A-s335 [file RA-011-D1RA00914A-s335.png]

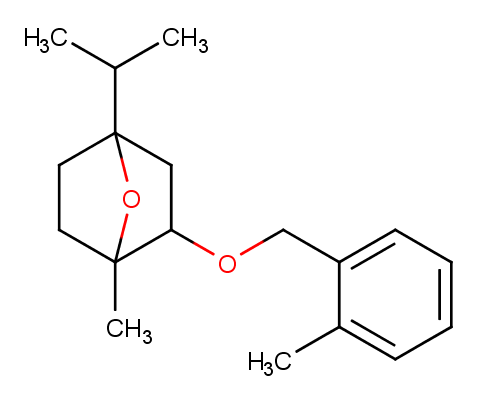

Supplement: RA-011-D1RA00914A-s336 [file RA-011-D1RA00914A-s336.png]

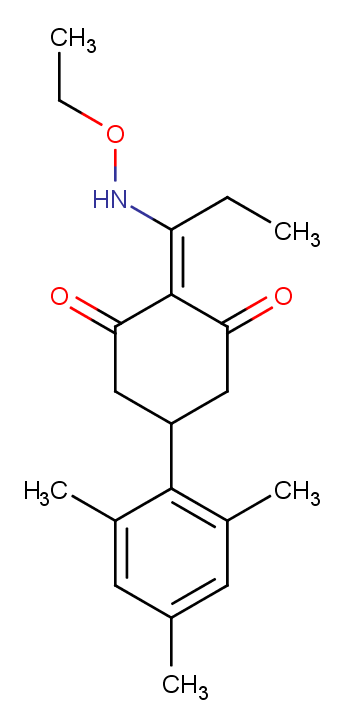

Supplement: RA-011-D1RA00914A-s337 [file RA-011-D1RA00914A-s337.png]

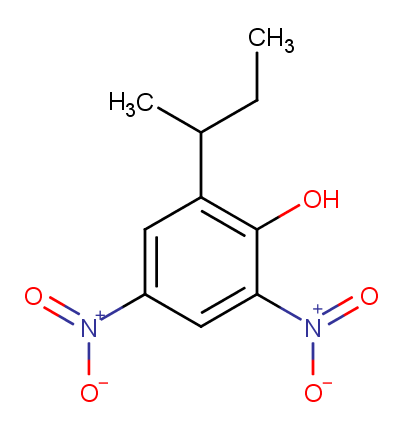

Supplement: RA-011-D1RA00914A-s338 [file RA-011-D1RA00914A-s338.png]

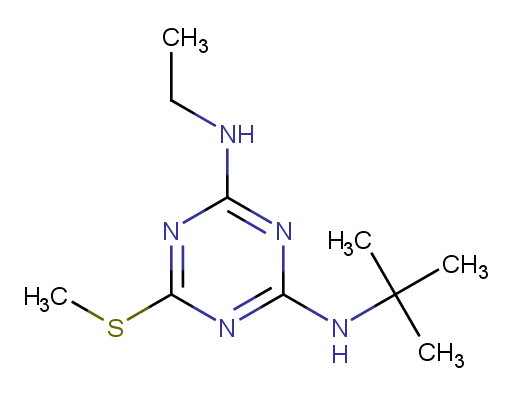

Supplement: RA-011-D1RA00914A-s339 [file RA-011-D1RA00914A-s339.png]

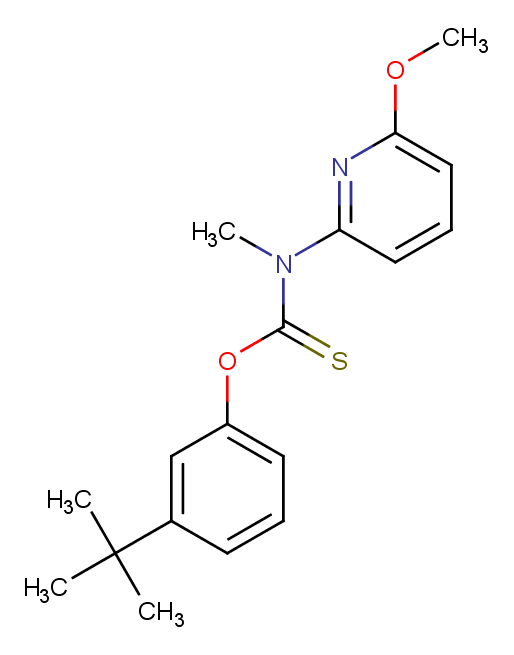

Supplement: RA-011-D1RA00914A-s340 [file RA-011-D1RA00914A-s340.png]

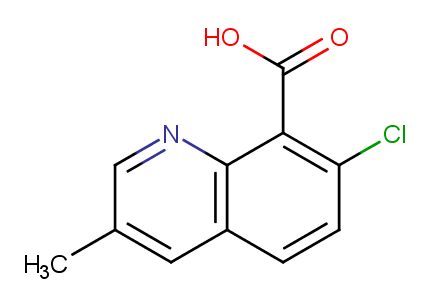

Supplement: RA-011-D1RA00914A-s341 [file RA-011-D1RA00914A-s341.png]

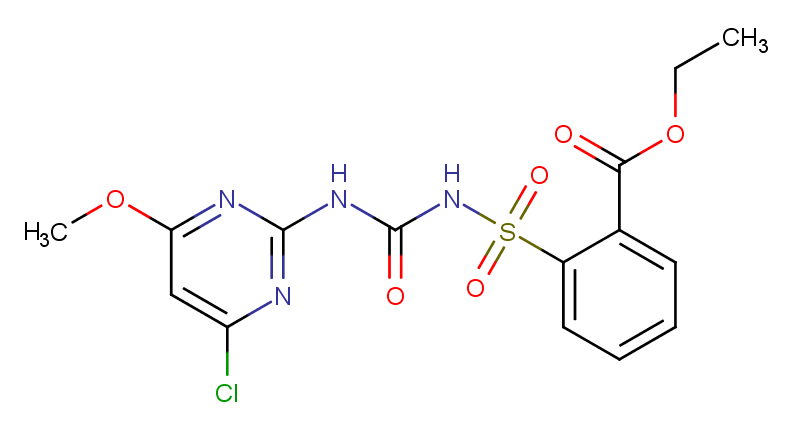

Supplement: RA-011-D1RA00914A-s342 [file RA-011-D1RA00914A-s342.png]

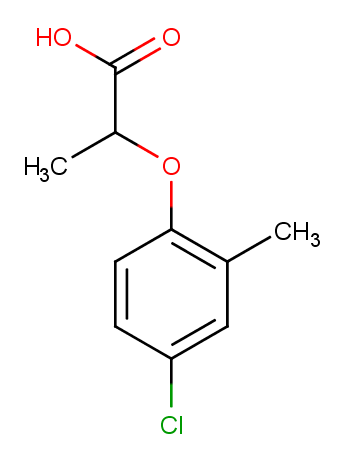

Supplement: RA-011-D1RA00914A-s343 [file RA-011-D1RA00914A-s343.png]

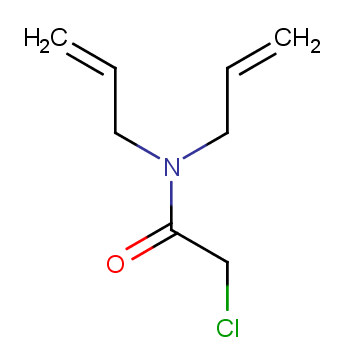

Supplement: RA-011-D1RA00914A-s344 [file RA-011-D1RA00914A-s344.png]

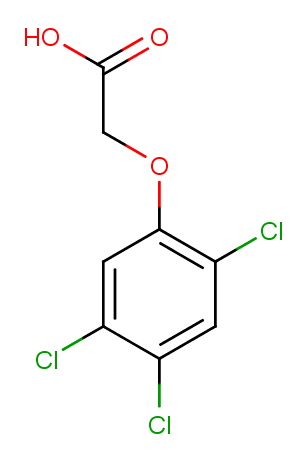

Supplement: RA-011-D1RA00914A-s345 [file RA-011-D1RA00914A-s345.png]

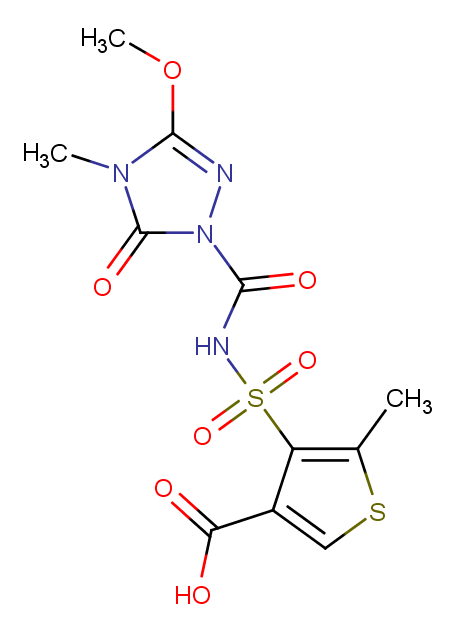

Supplement: RA-011-D1RA00914A-s346 [file RA-011-D1RA00914A-s346.png]

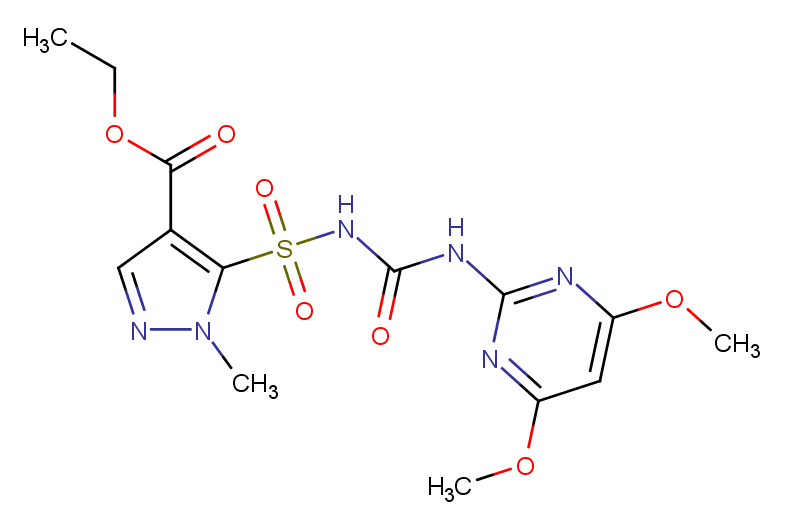

Supplement: RA-011-D1RA00914A-s347 [file RA-011-D1RA00914A-s347.png]

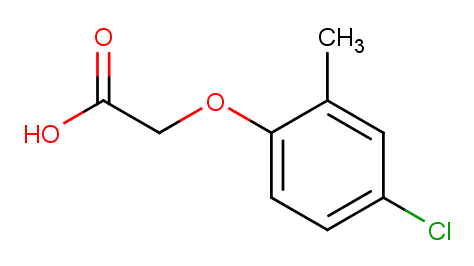

Supplement: RA-011-D1RA00914A-s348 [file RA-011-D1RA00914A-s348.png]

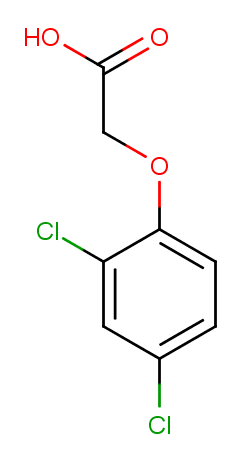

Supplement: RA-011-D1RA00914A-s349 [file RA-011-D1RA00914A-s349.png]

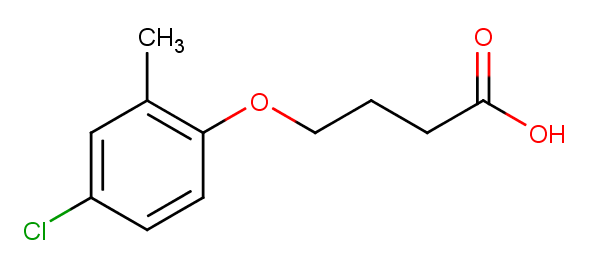

Supplement: RA-011-D1RA00914A-s350 [file RA-011-D1RA00914A-s350.png]

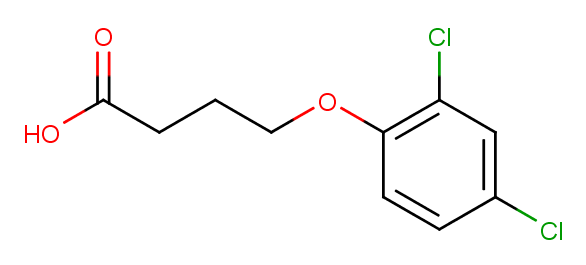

Supplement: RA-011-D1RA00914A-s351 [file RA-011-D1RA00914A-s351.png]

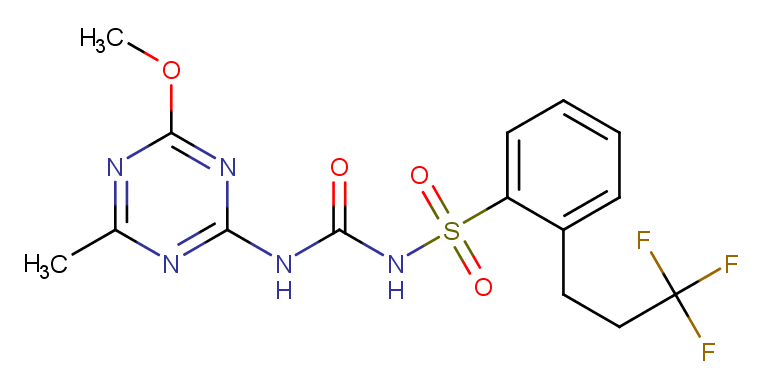

Supplement: RA-011-D1RA00914A-s352 [file RA-011-D1RA00914A-s352.png]

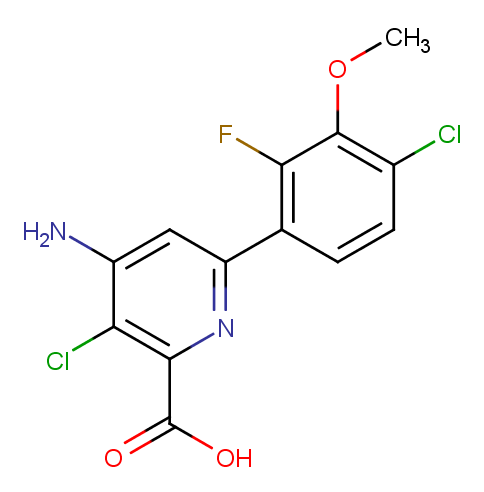

Supplement: RA-011-D1RA00914A-s353 [file RA-011-D1RA00914A-s353.png]

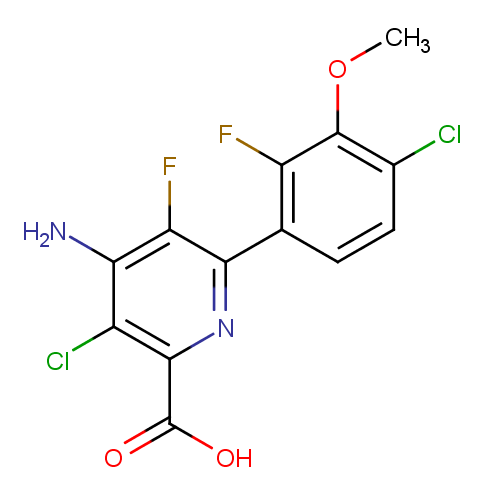

Supplement: RA-011-D1RA00914A-s354 [file RA-011-D1RA00914A-s354.png]

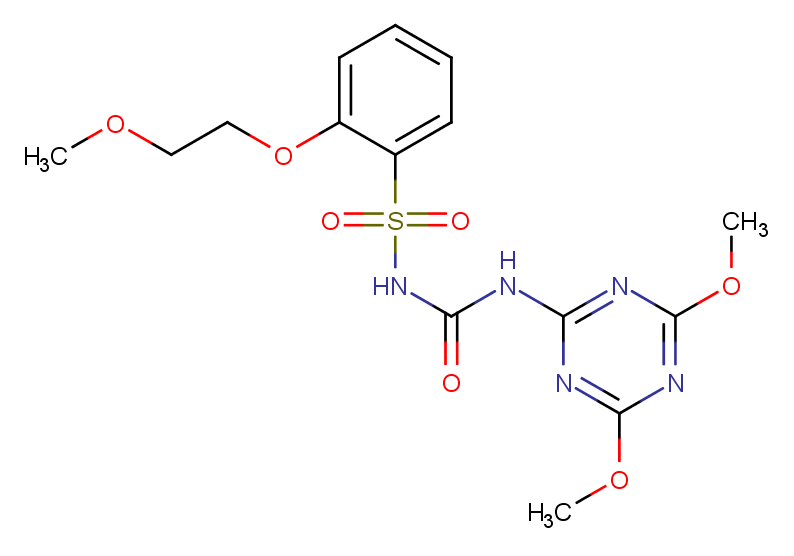

Supplement: RA-011-D1RA00914A-s355 [file RA-011-D1RA00914A-s355.png]

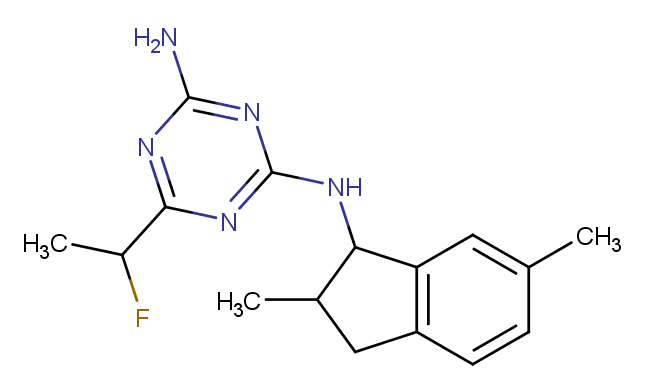

Supplement: RA-011-D1RA00914A-s356 [file RA-011-D1RA00914A-s356.png]

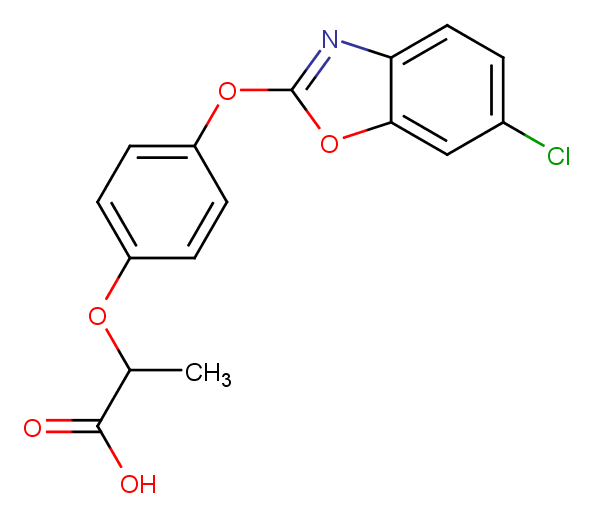

Supplement: RA-011-D1RA00914A-s357 [file RA-011-D1RA00914A-s357.png]

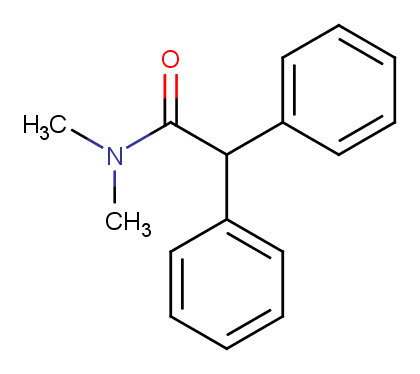

Supplement: RA-011-D1RA00914A-s358 [file RA-011-D1RA00914A-s358.png]

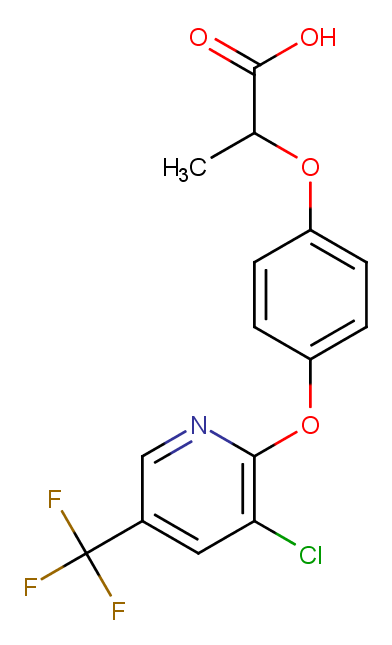

Supplement: RA-011-D1RA00914A-s359 [file RA-011-D1RA00914A-s359.png]

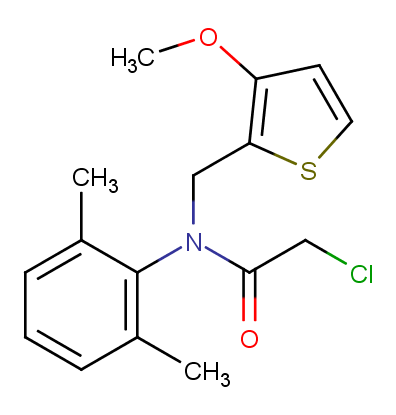

Supplement: RA-011-D1RA00914A-s360 [file RA-011-D1RA00914A-s360.png]

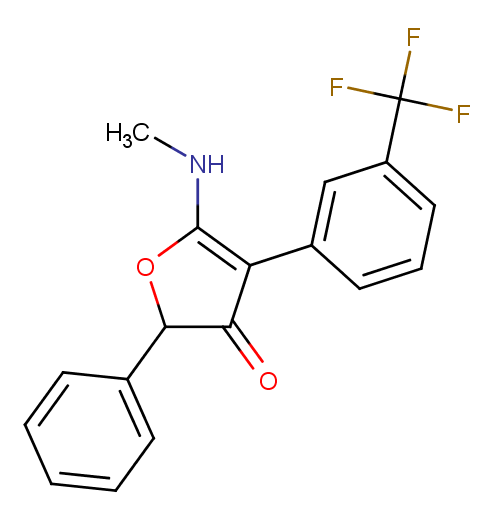

Supplement: RA-011-D1RA00914A-s361 [file RA-011-D1RA00914A-s361.png]

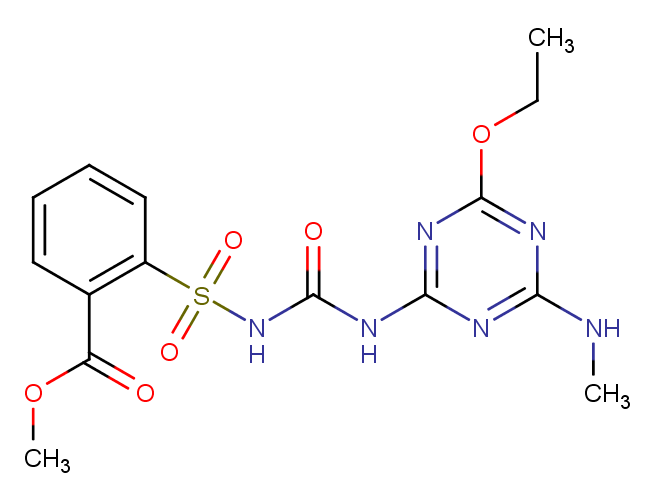

Supplement: RA-011-D1RA00914A-s362 [file RA-011-D1RA00914A-s362.png]

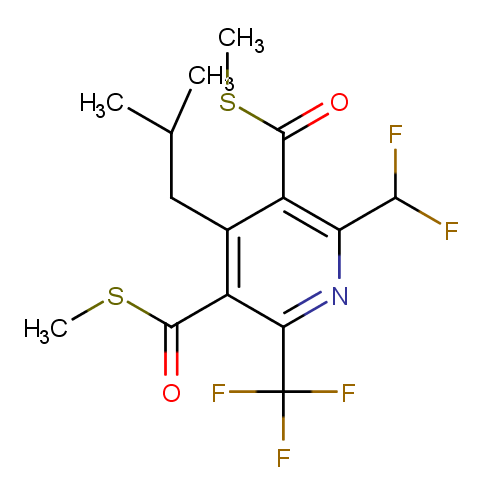

Supplement: RA-011-D1RA00914A-s363 [file RA-011-D1RA00914A-s363.png]

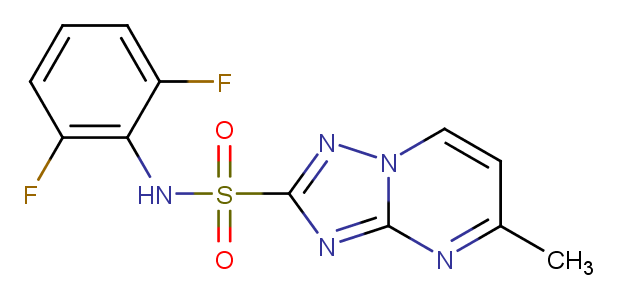

Supplement: RA-011-D1RA00914A-s364 [file RA-011-D1RA00914A-s364.png]

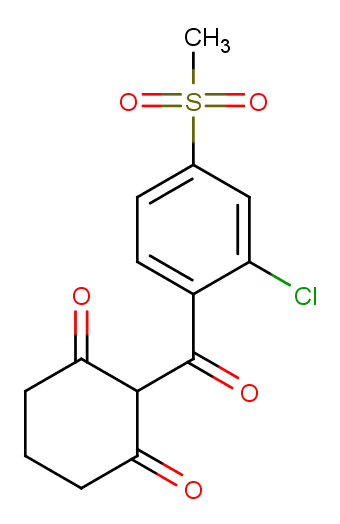

Supplement: RA-011-D1RA00914A-s365 [file RA-011-D1RA00914A-s365.png]

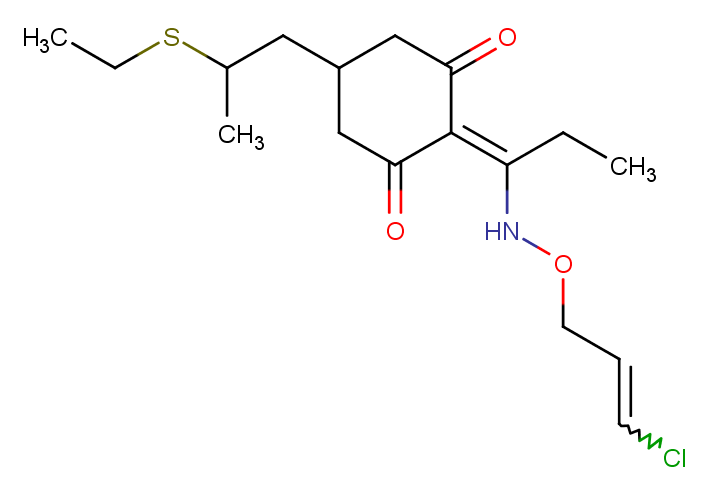

Supplement: RA-011-D1RA00914A-s366 [file RA-011-D1RA00914A-s366.png]

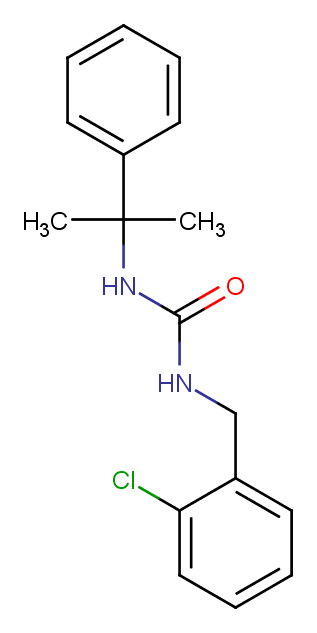

Supplement: RA-011-D1RA00914A-s367 [file RA-011-D1RA00914A-s367.png]

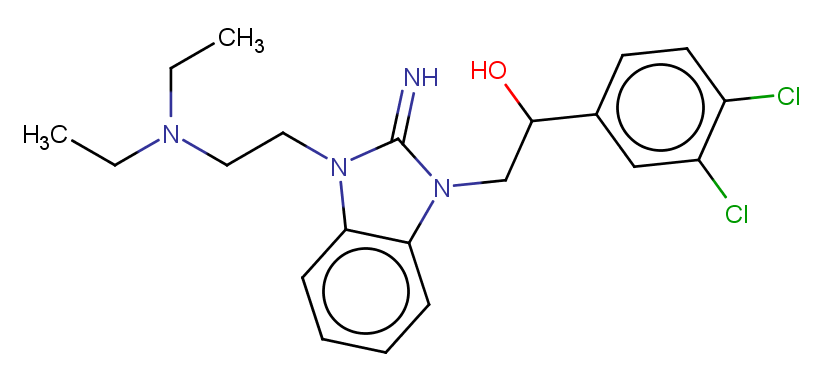

Supplement: RA-011-D1RA00914A-s735 [file RA-011-D1RA00914A-s735.png]

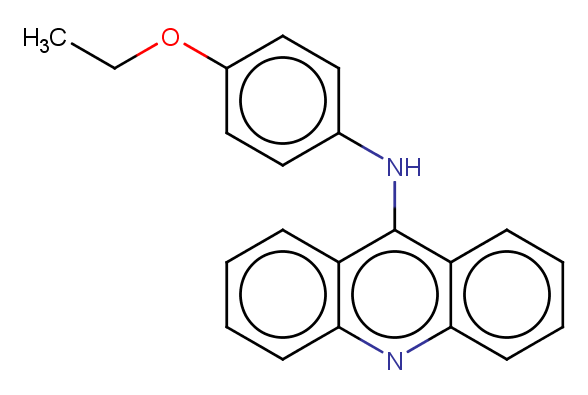

Supplement: RA-011-D1RA00914A-s736 [file RA-011-D1RA00914A-s736.png]

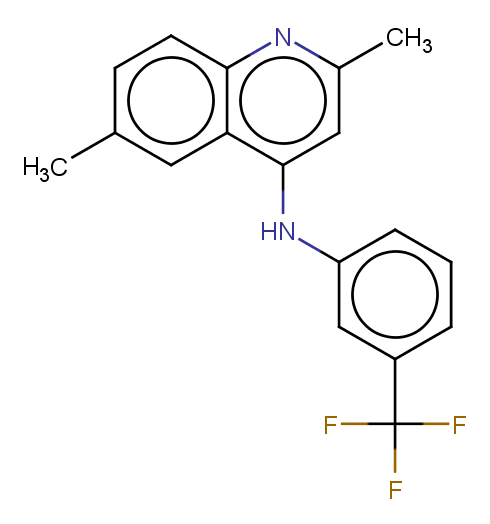

Supplement: RA-011-D1RA00914A-s737 [file RA-011-D1RA00914A-s737.png]

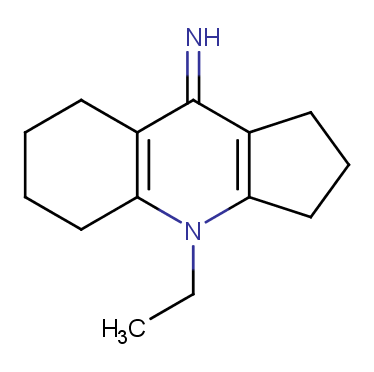

Supplement: RA-011-D1RA00914A-s738 [file RA-011-D1RA00914A-s738.png]

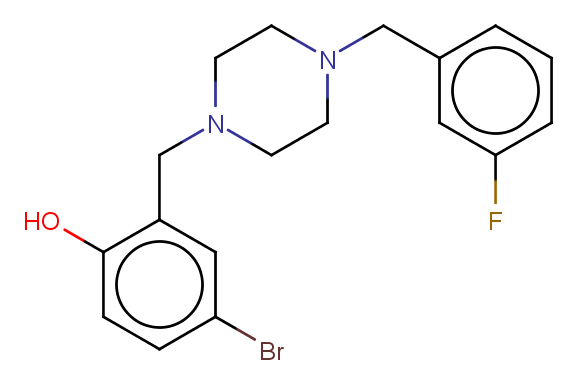

Supplement: RA-011-D1RA00914A-s739 [file RA-011-D1RA00914A-s739.png]

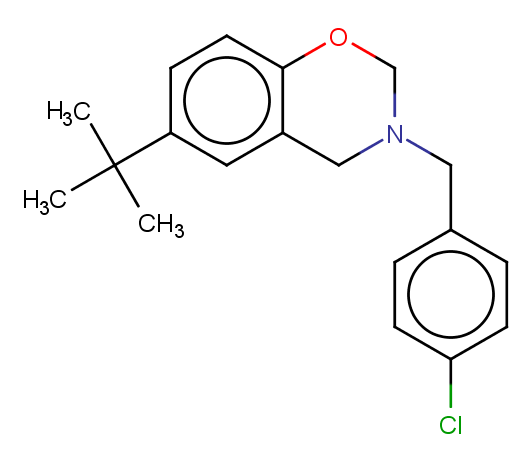

Supplement: RA-011-D1RA00914A-s740 [file RA-011-D1RA00914A-s740.png]

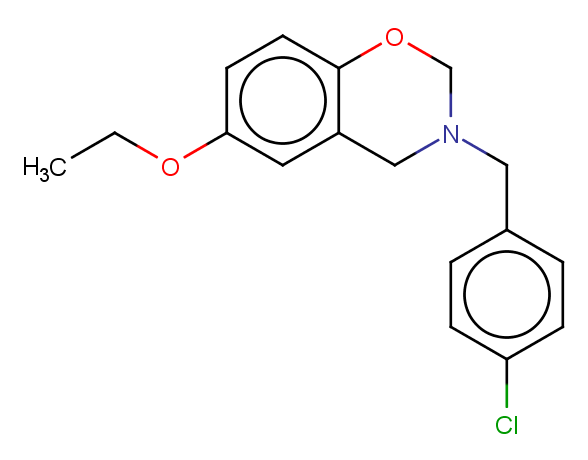

Supplement: RA-011-D1RA00914A-s741 [file RA-011-D1RA00914A-s741.png]

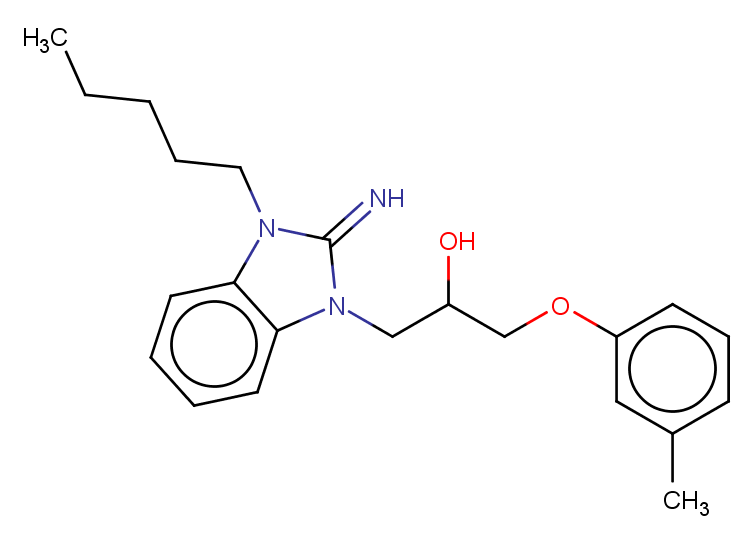

Supplement: RA-011-D1RA00914A-s742 [file RA-011-D1RA00914A-s742.png]

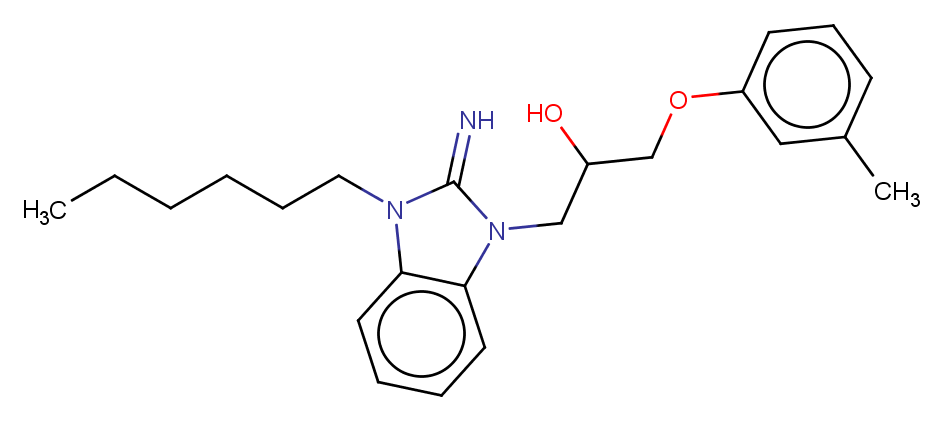

Supplement: RA-011-D1RA00914A-s743 [file RA-011-D1RA00914A-s743.png]

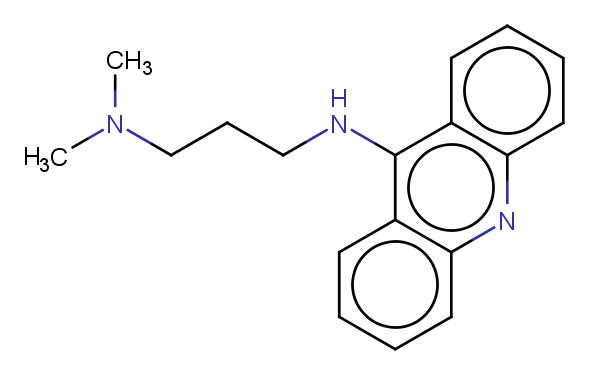

Supplement: RA-011-D1RA00914A-s744 [file RA-011-D1RA00914A-s744.png]

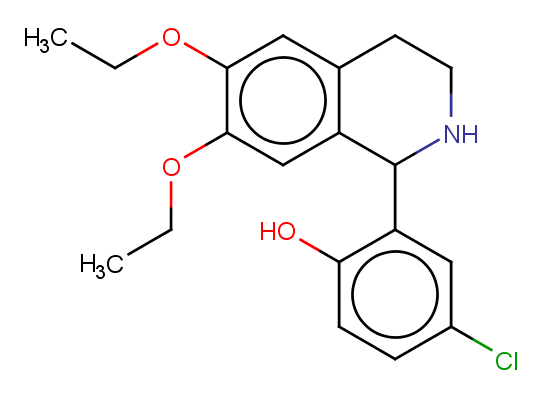

Supplement: RA-011-D1RA00914A-s745 [file RA-011-D1RA00914A-s745.png]

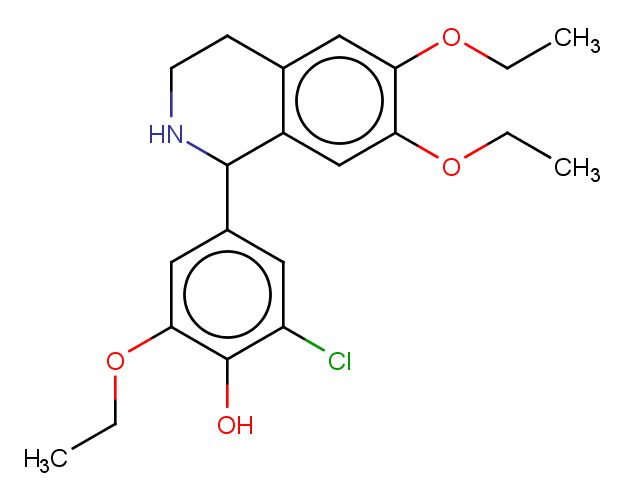

Supplement: RA-011-D1RA00914A-s746 [file RA-011-D1RA00914A-s746.png]

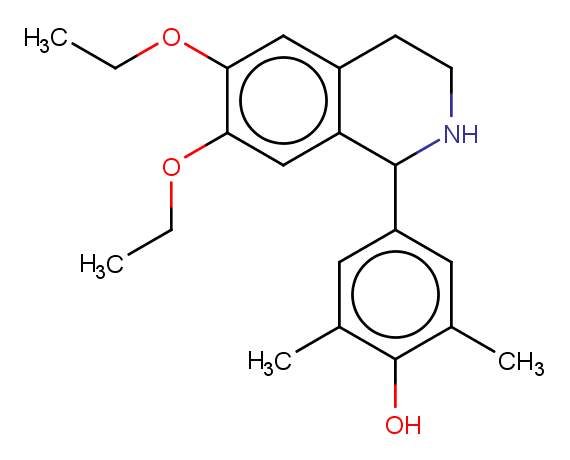

Supplement: RA-011-D1RA00914A-s747 [file RA-011-D1RA00914A-s747.png]

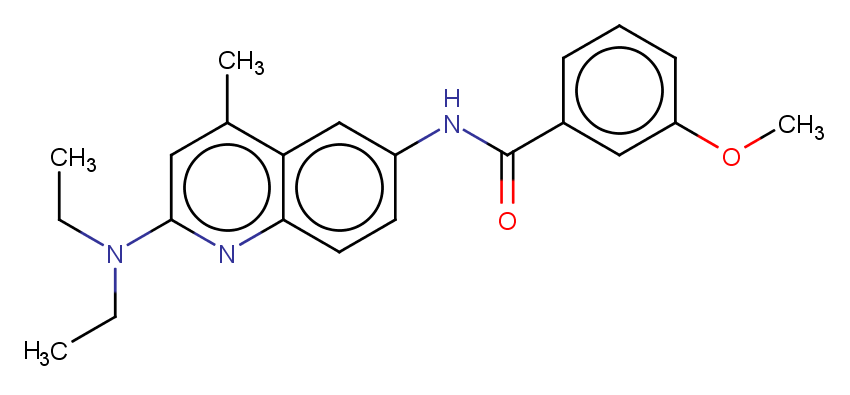

Supplement: RA-011-D1RA00914A-s748 [file RA-011-D1RA00914A-s748.png]

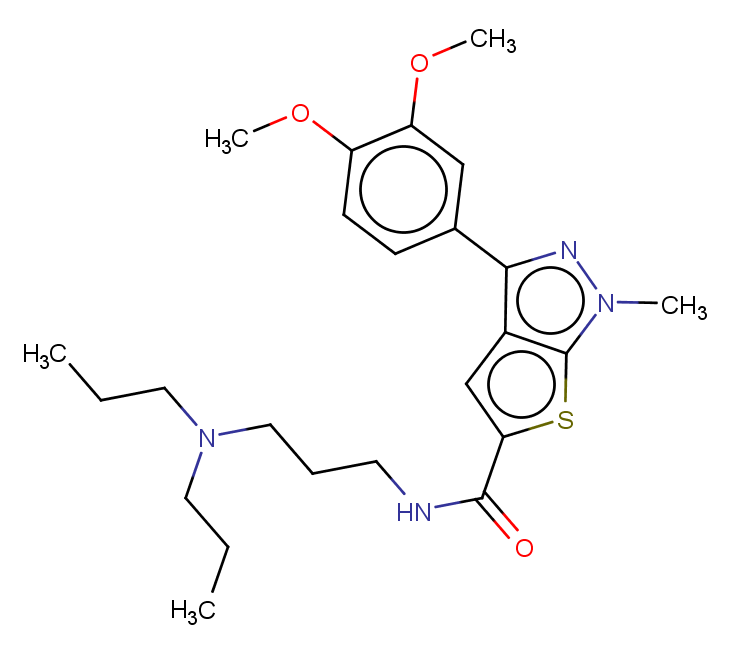

Supplement: RA-011-D1RA00914A-s749 [file RA-011-D1RA00914A-s749.png]

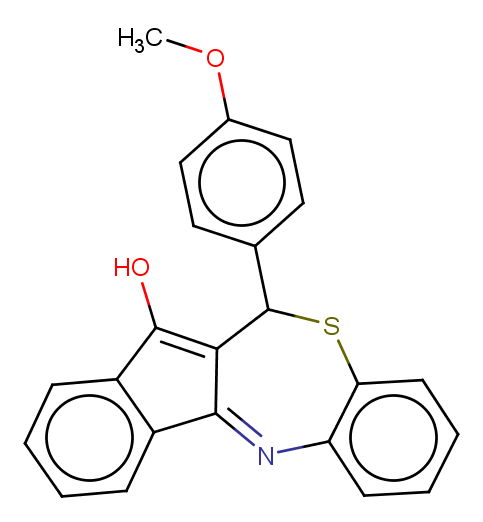

Supplement: RA-011-D1RA00914A-s750 [file RA-011-D1RA00914A-s750.png]

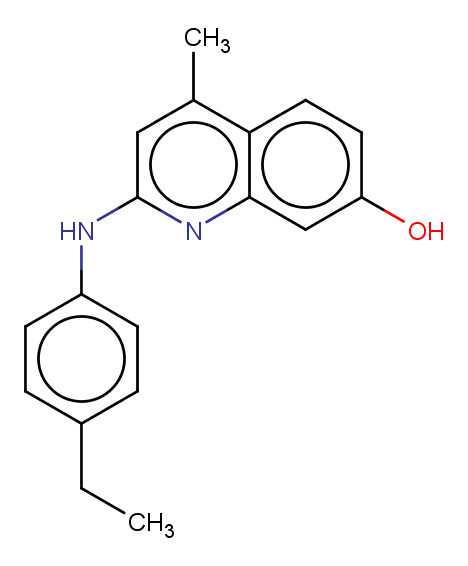

Supplement: RA-011-D1RA00914A-s751 [file RA-011-D1RA00914A-s751.png]

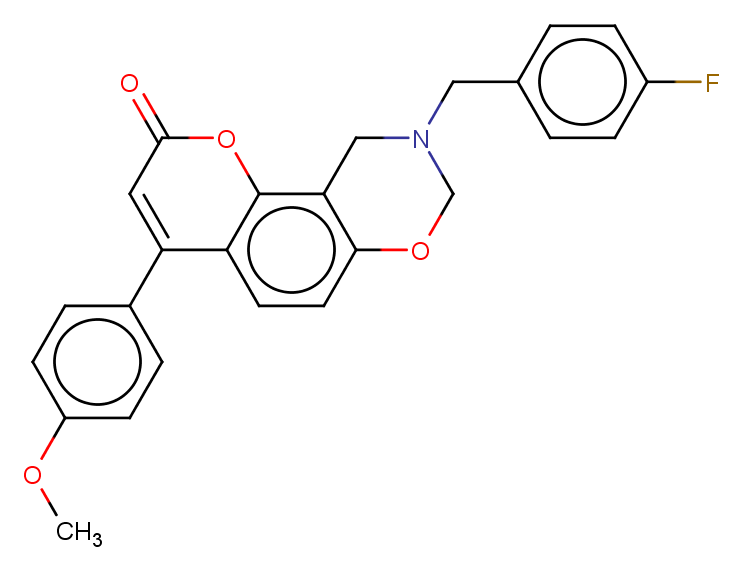

Supplement: RA-011-D1RA00914A-s752 [file RA-011-D1RA00914A-s752.png]

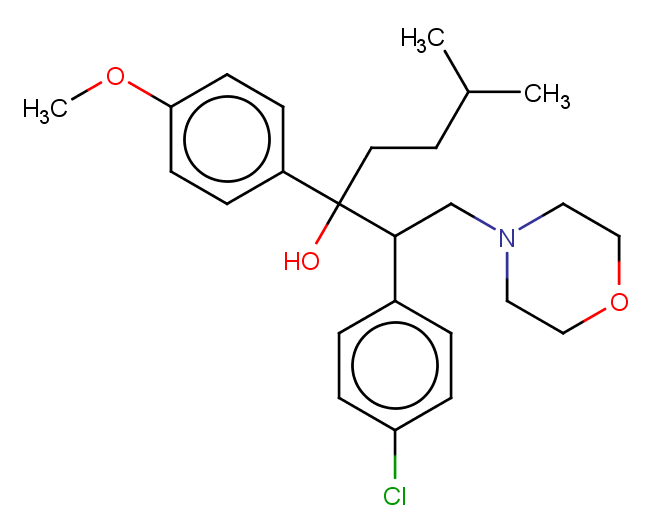

Supplement: RA-011-D1RA00914A-s753 [file RA-011-D1RA00914A-s753.png]

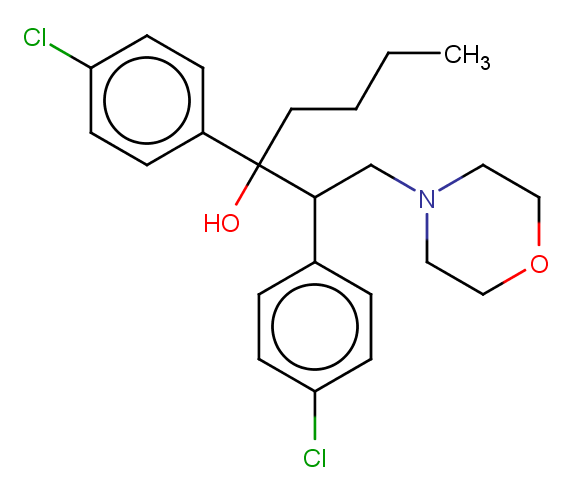

Supplement: RA-011-D1RA00914A-s754 [file RA-011-D1RA00914A-s754.png]

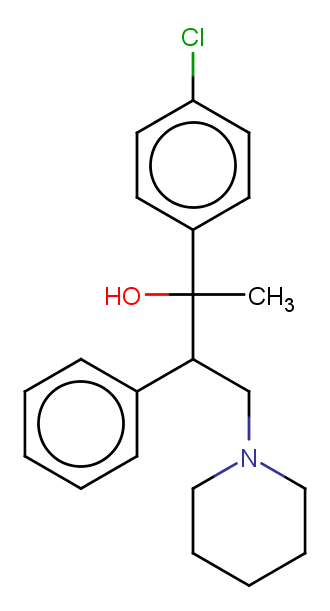

Supplement: RA-011-D1RA00914A-s755 [file RA-011-D1RA00914A-s755.png]

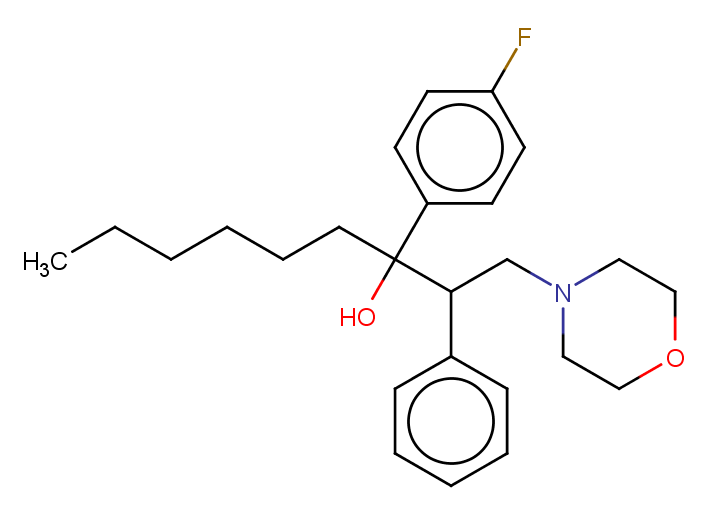

Supplement: RA-011-D1RA00914A-s756 [file RA-011-D1RA00914A-s756.png]

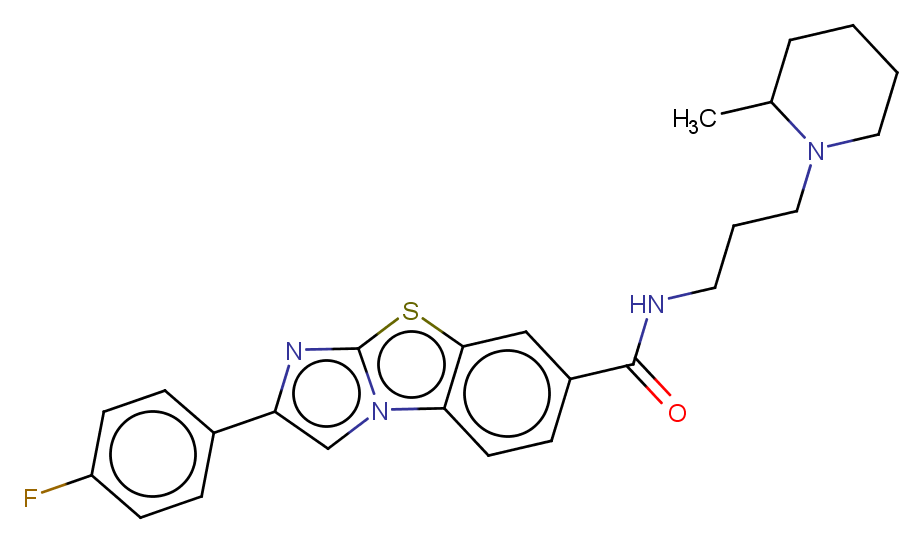

Supplement: RA-011-D1RA00914A-s757 [file RA-011-D1RA00914A-s757.png]

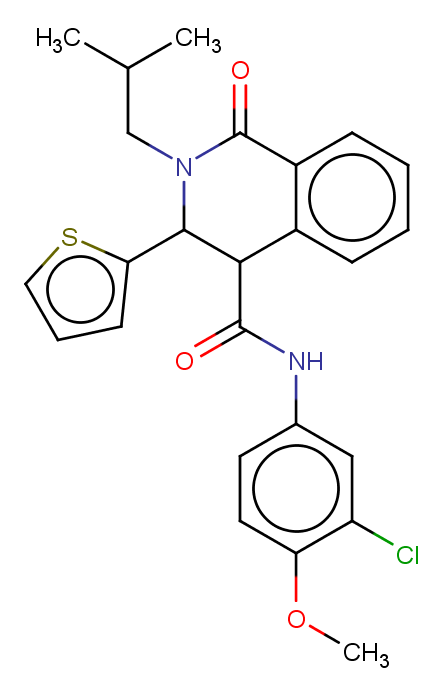

Supplement: RA-011-D1RA00914A-s758 [file RA-011-D1RA00914A-s758.png]

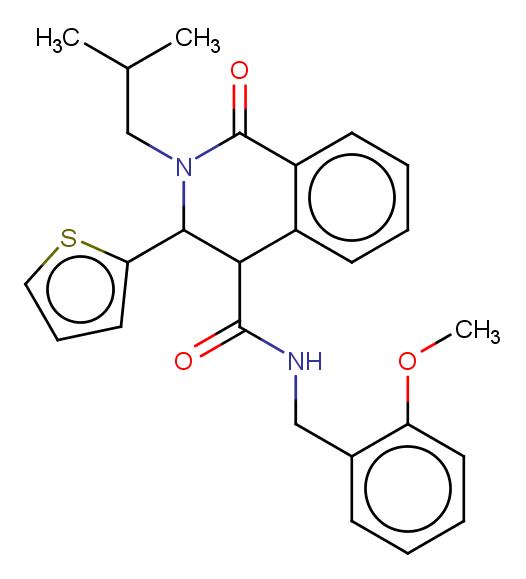

Supplement: RA-011-D1RA00914A-s759 [file RA-011-D1RA00914A-s759.png]

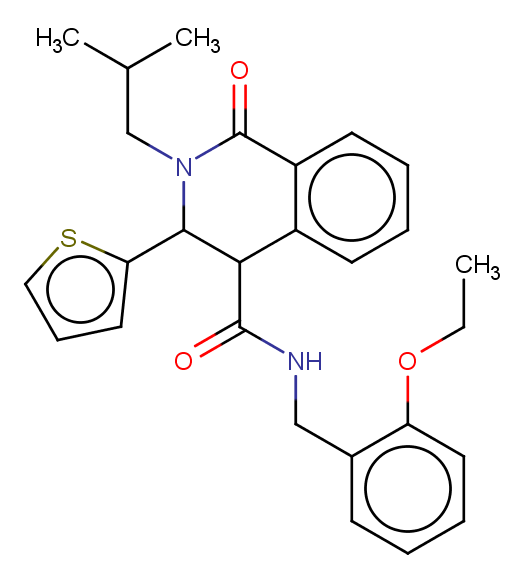

Supplement: RA-011-D1RA00914A-s760 [file RA-011-D1RA00914A-s760.png]

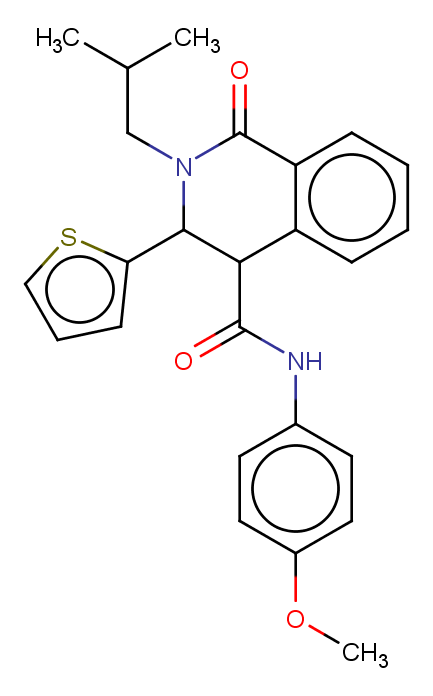

Supplement: RA-011-D1RA00914A-s761 [file RA-011-D1RA00914A-s761.png]

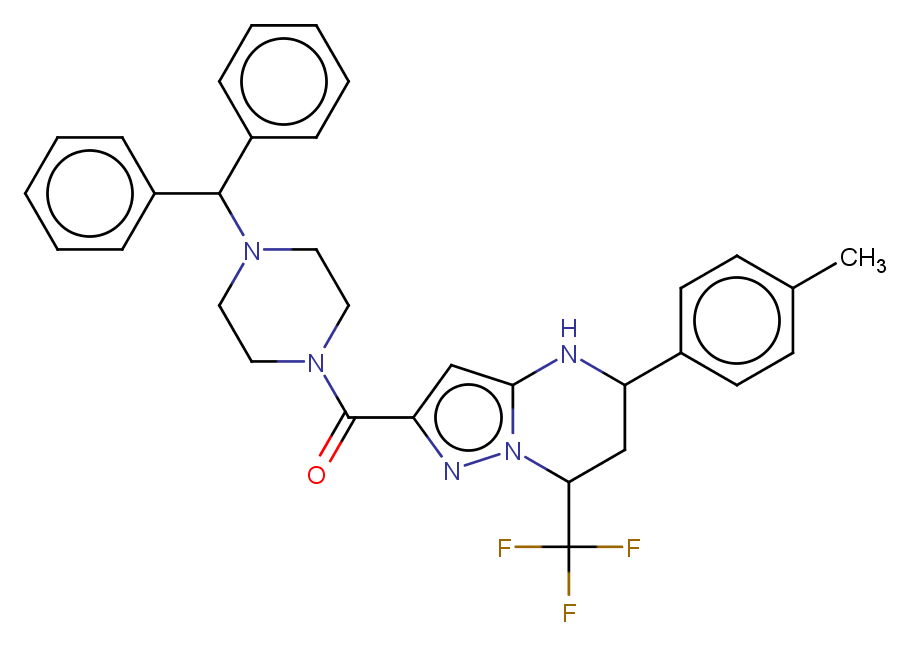

Supplement: RA-011-D1RA00914A-s762 [file RA-011-D1RA00914A-s762.png]

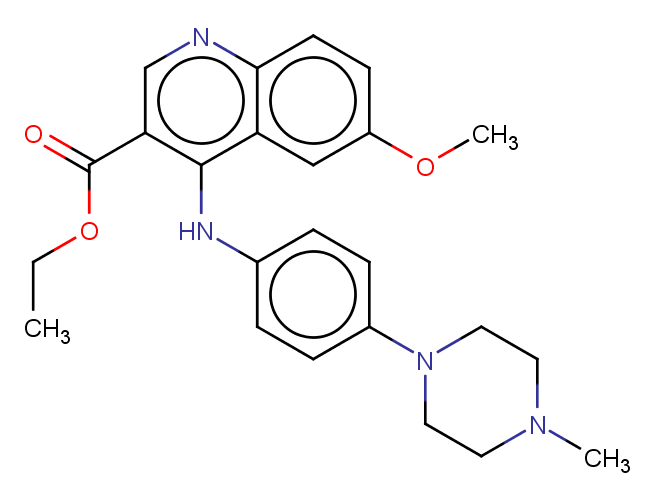

Supplement: RA-011-D1RA00914A-s763 [file RA-011-D1RA00914A-s763.png]

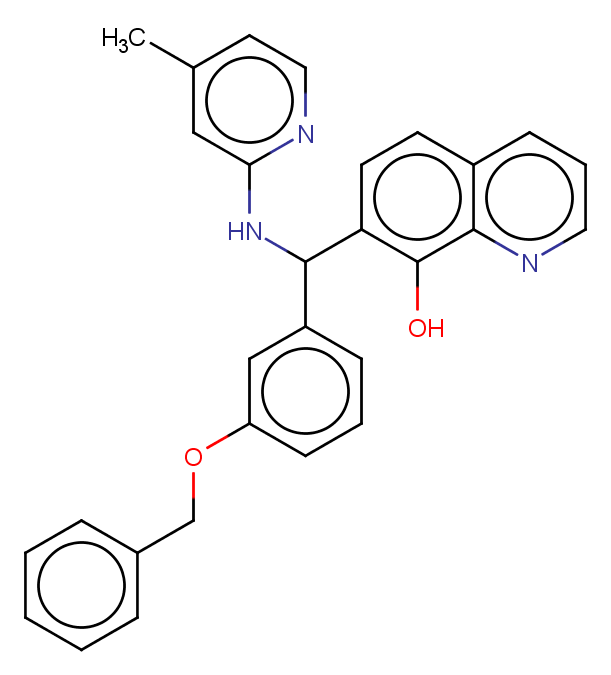

Supplement: RA-011-D1RA00914A-s764 [file RA-011-D1RA00914A-s764.png]

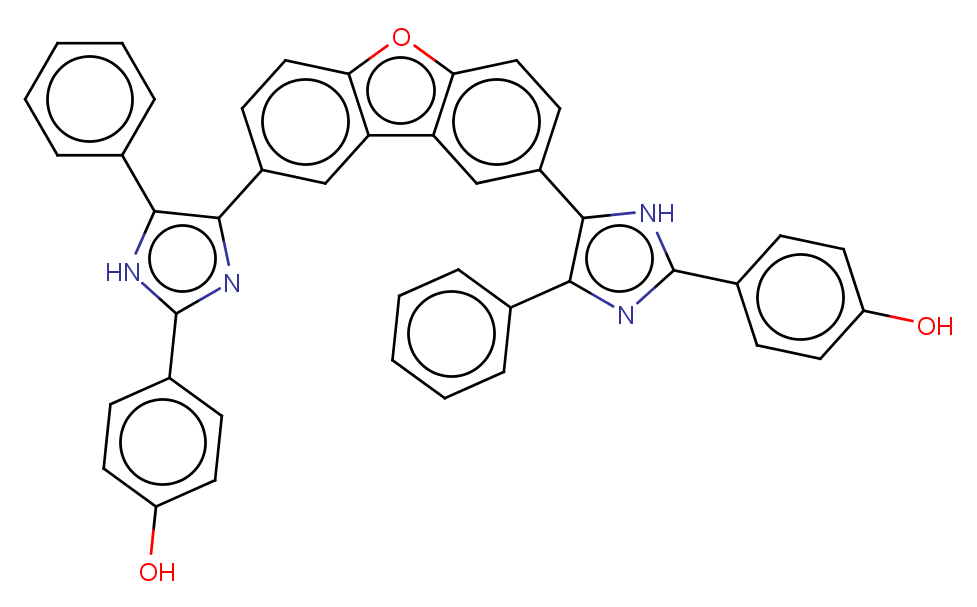

Supplement: RA-011-D1RA00914A-s765 [file RA-011-D1RA00914A-s765.png]

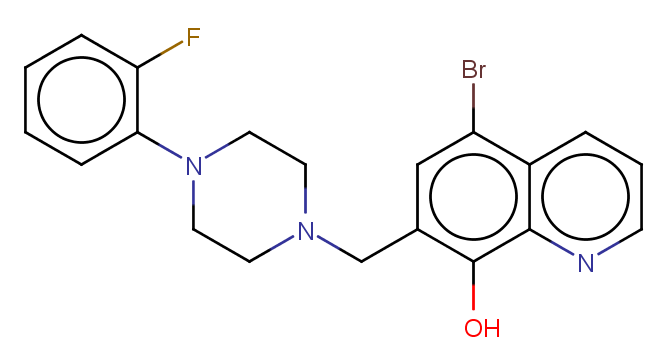

Supplement: RA-011-D1RA00914A-s766 [file RA-011-D1RA00914A-s766.png]

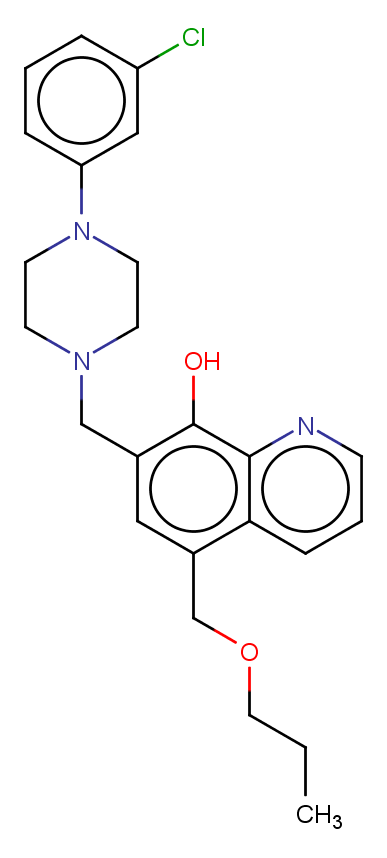

Supplement: RA-011-D1RA00914A-s767 [file RA-011-D1RA00914A-s767.png]

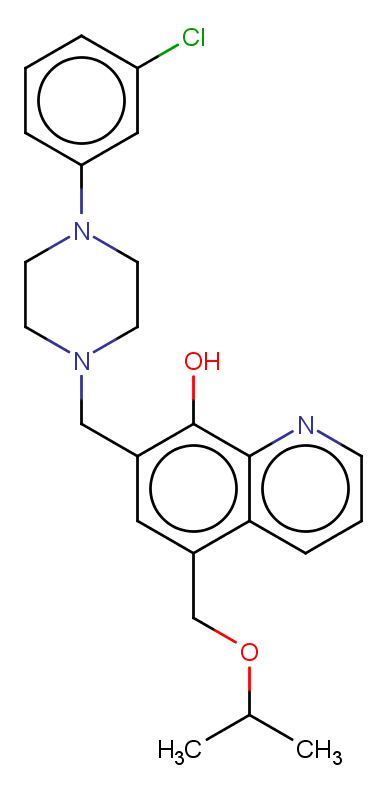

Supplement: RA-011-D1RA00914A-s768 [file RA-011-D1RA00914A-s768.png]

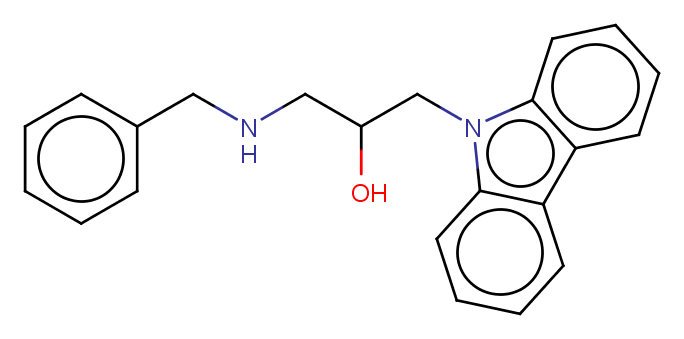

Supplement: RA-011-D1RA00914A-s769 [file RA-011-D1RA00914A-s769.png]

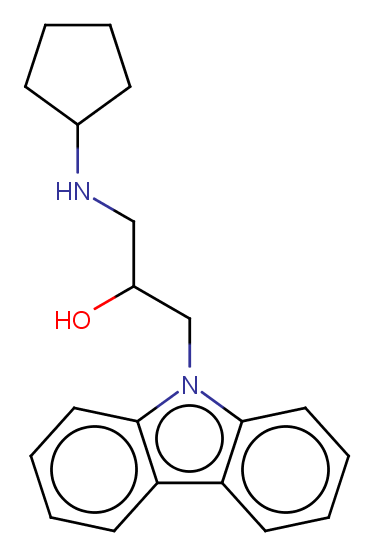

Supplement: RA-011-D1RA00914A-s770 [file RA-011-D1RA00914A-s770.png]

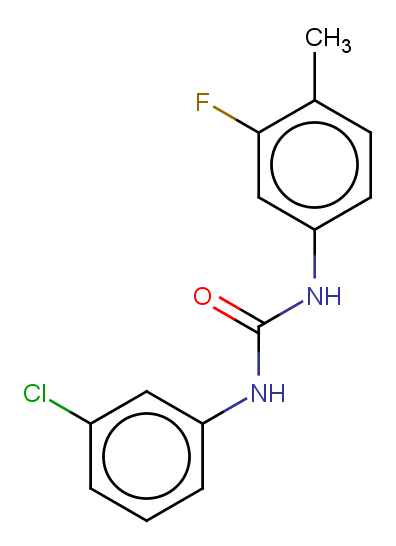

Supplement: RA-011-D1RA00914A-s771 [file RA-011-D1RA00914A-s771.png]
