# Supplementary figures and images for: Improved herbicide discovery using physico-chemical rules refined by antimalarial library screening (part 5 of 14)
Source: RSC Adv. 2021 Feb 23;11(15):8459–67. doi: 10.1039/d1ra00914a (PMC8695207; doi:10.1039/d1ra00914a)

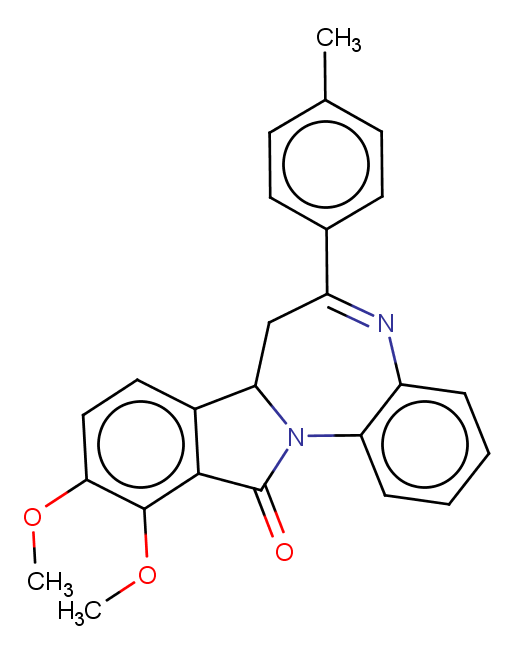

Supplement: RA-011-D1RA00914A-s772 [file RA-011-D1RA00914A-s772.png]

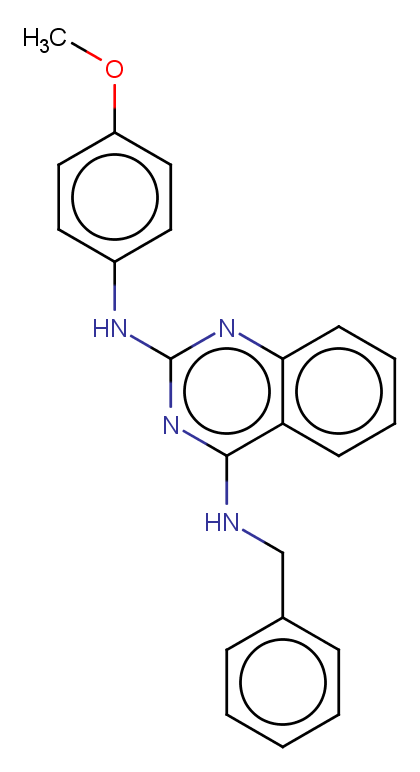

Supplement: RA-011-D1RA00914A-s773 [file RA-011-D1RA00914A-s773.png]

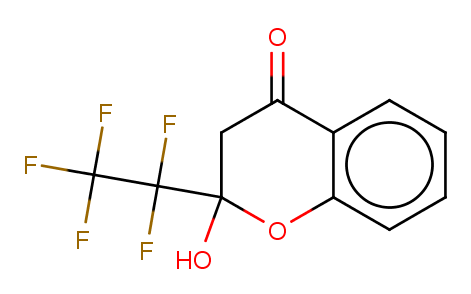

Supplement: RA-011-D1RA00914A-s774 [file RA-011-D1RA00914A-s774.png]

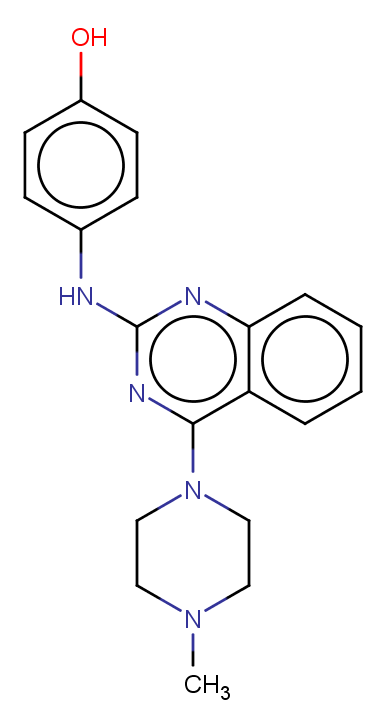

Supplement: RA-011-D1RA00914A-s775 [file RA-011-D1RA00914A-s775.png]

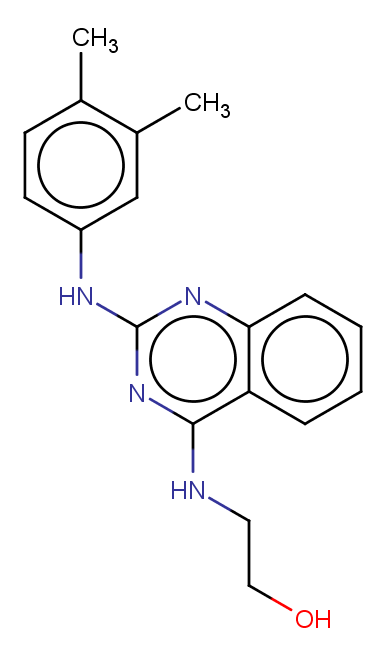

Supplement: RA-011-D1RA00914A-s776 [file RA-011-D1RA00914A-s776.png]

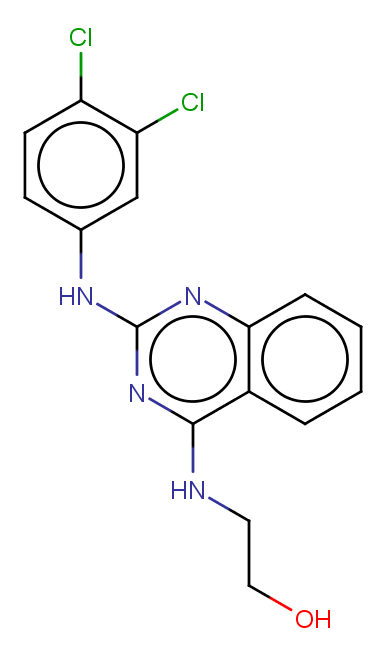

Supplement: RA-011-D1RA00914A-s777 [file RA-011-D1RA00914A-s777.png]

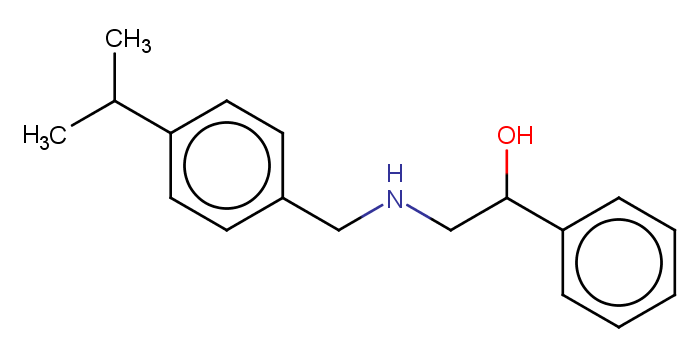

Supplement: RA-011-D1RA00914A-s778 [file RA-011-D1RA00914A-s778.png]

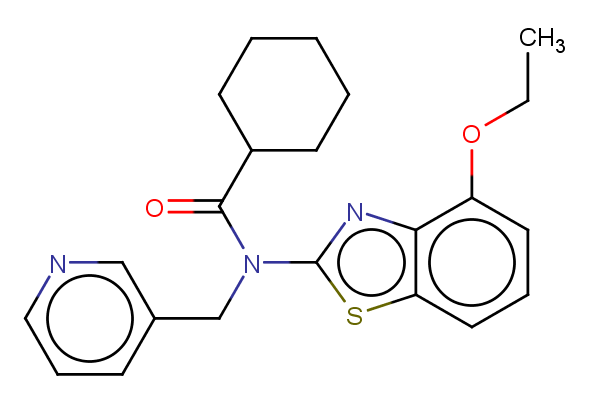

Supplement: RA-011-D1RA00914A-s779 [file RA-011-D1RA00914A-s779.png]

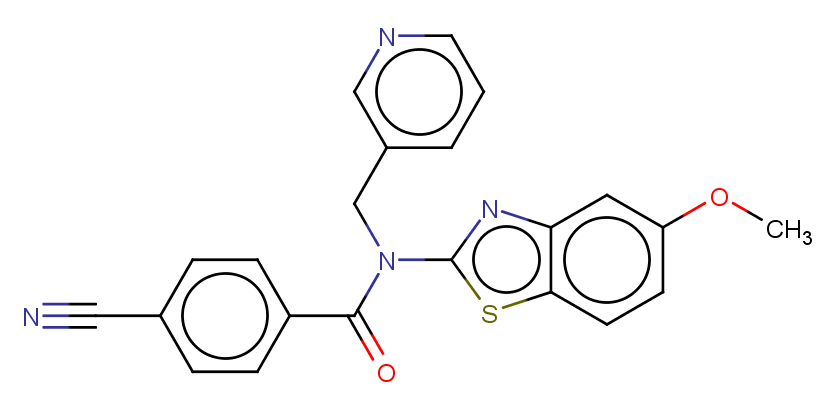

Supplement: RA-011-D1RA00914A-s780 [file RA-011-D1RA00914A-s780.png]

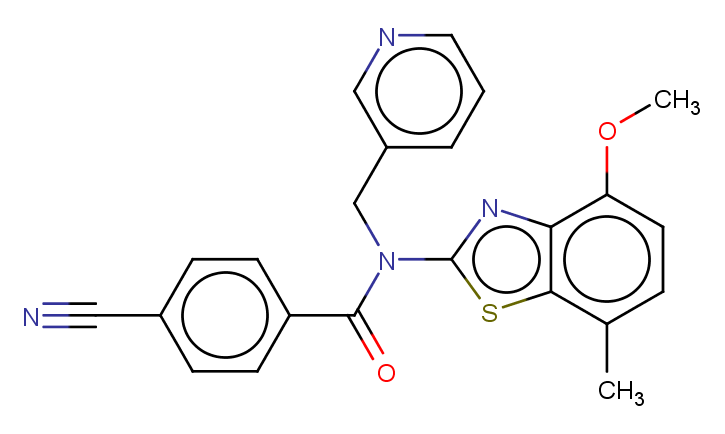

Supplement: RA-011-D1RA00914A-s781 [file RA-011-D1RA00914A-s781.png]

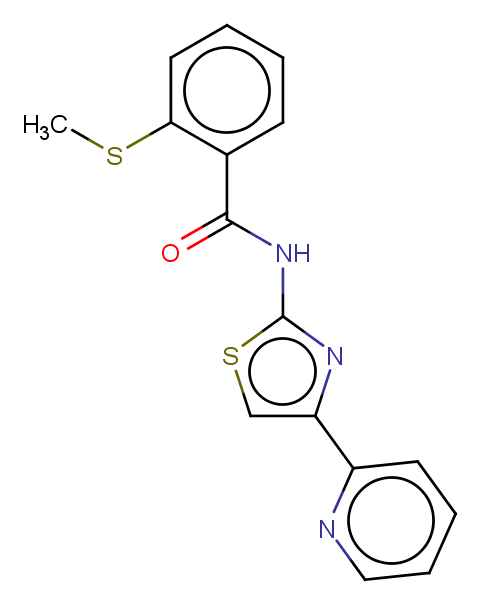

Supplement: RA-011-D1RA00914A-s782 [file RA-011-D1RA00914A-s782.png]

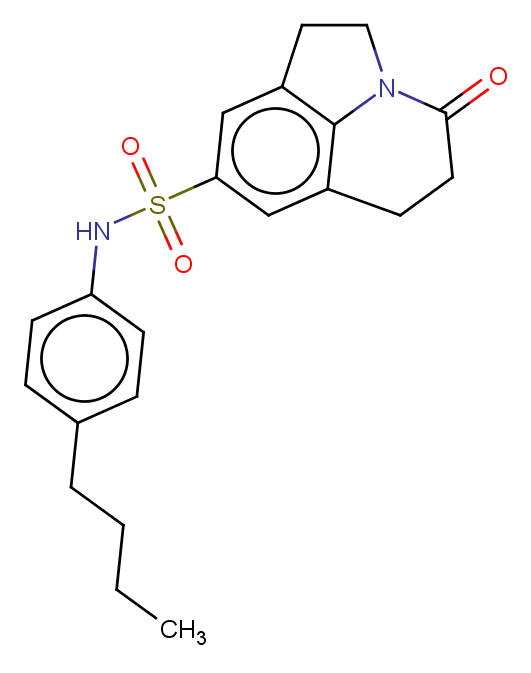

Supplement: RA-011-D1RA00914A-s783 [file RA-011-D1RA00914A-s783.png]

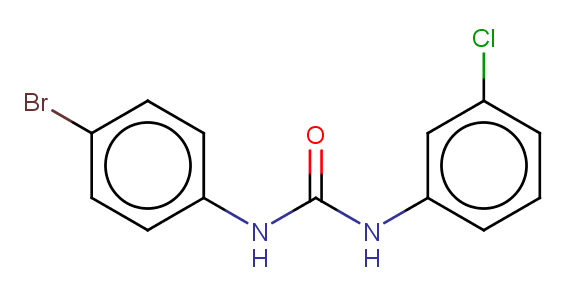

Supplement: RA-011-D1RA00914A-s784 [file RA-011-D1RA00914A-s784.png]

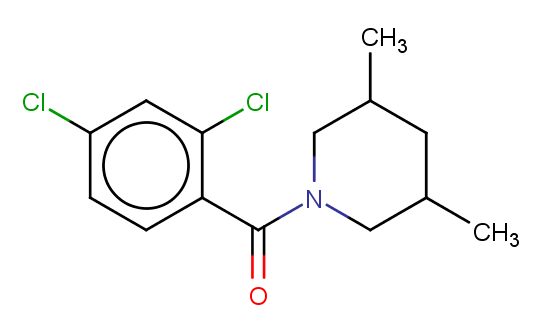

Supplement: RA-011-D1RA00914A-s785 [file RA-011-D1RA00914A-s785.png]

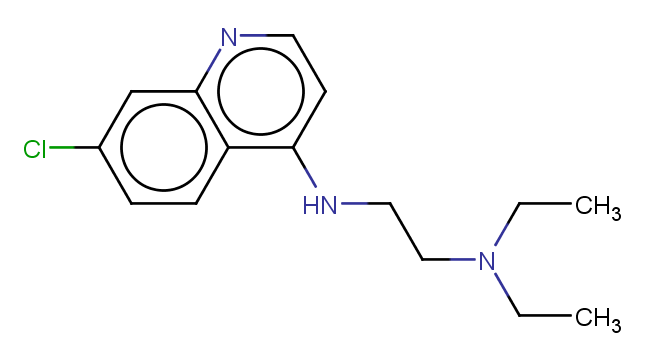

Supplement: RA-011-D1RA00914A-s786 [file RA-011-D1RA00914A-s786.png]

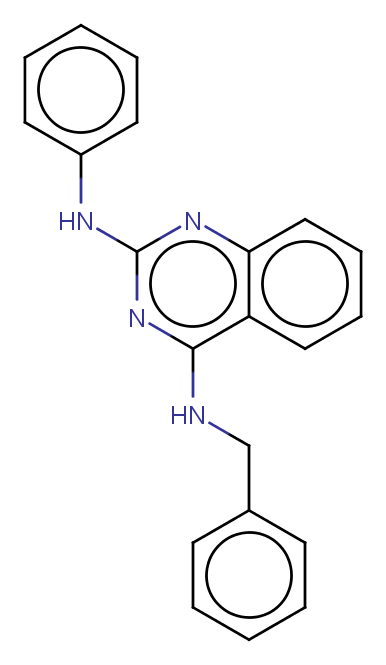

Supplement: RA-011-D1RA00914A-s787 [file RA-011-D1RA00914A-s787.png]

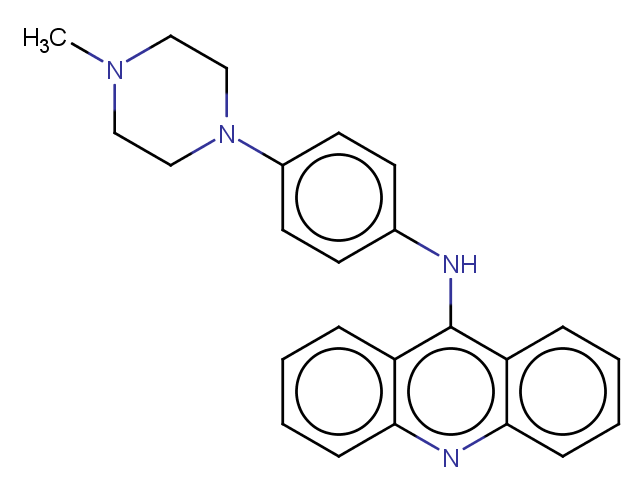

Supplement: RA-011-D1RA00914A-s788 [file RA-011-D1RA00914A-s788.png]

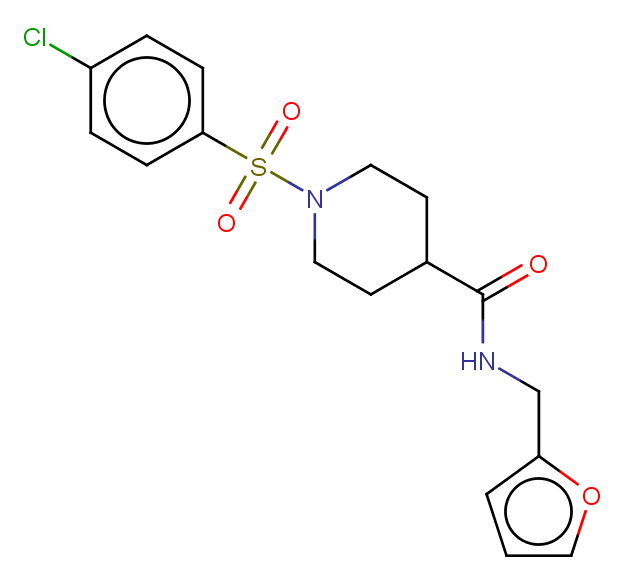

Supplement: RA-011-D1RA00914A-s789 [file RA-011-D1RA00914A-s789.png]

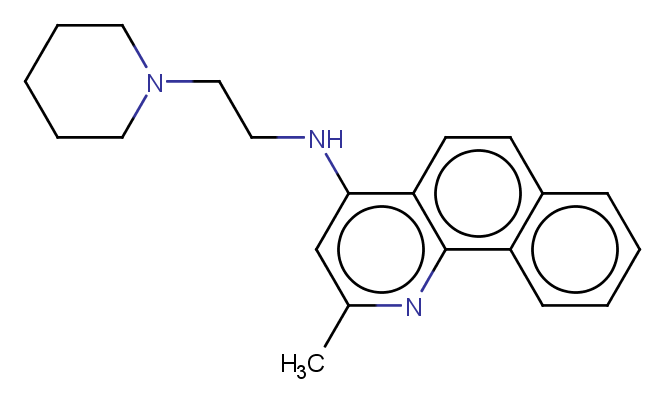

Supplement: RA-011-D1RA00914A-s790 [file RA-011-D1RA00914A-s790.png]

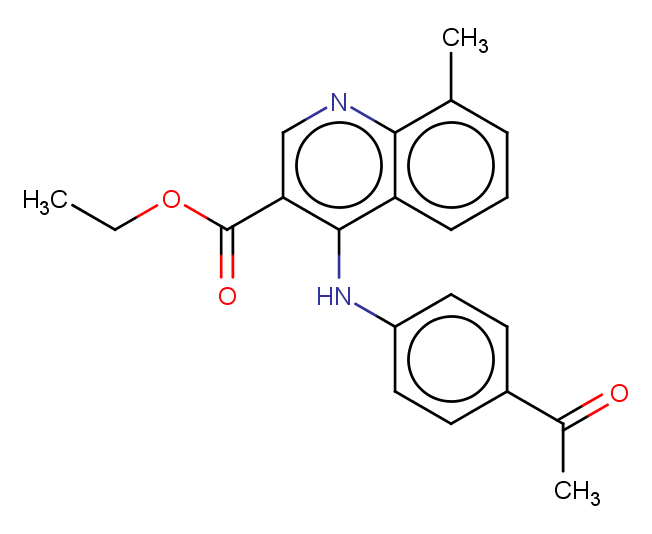

Supplement: RA-011-D1RA00914A-s791 [file RA-011-D1RA00914A-s791.png]

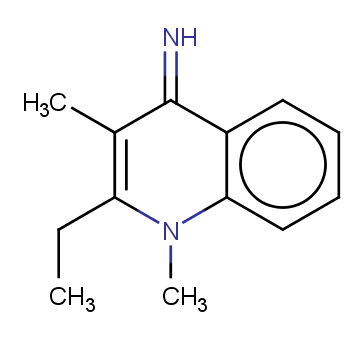

Supplement: RA-011-D1RA00914A-s792 [file RA-011-D1RA00914A-s792.png]

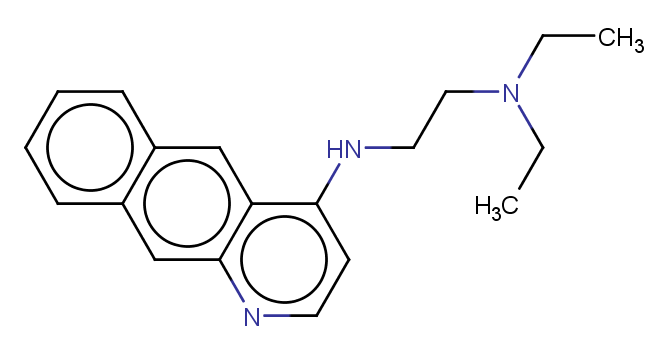

Supplement: RA-011-D1RA00914A-s793 [file RA-011-D1RA00914A-s793.png]

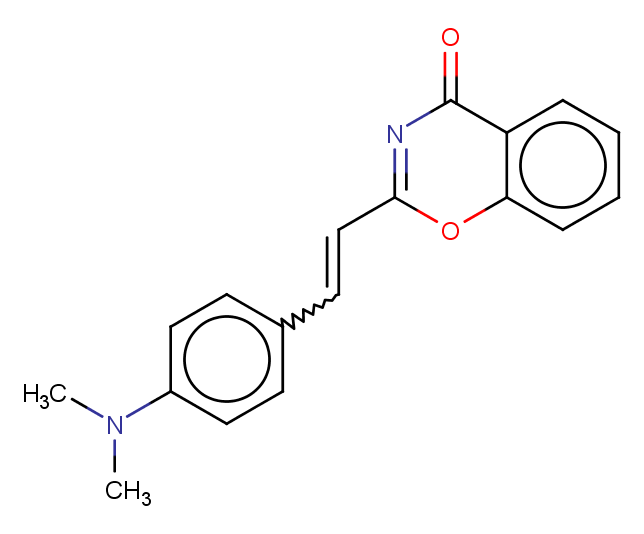

Supplement: RA-011-D1RA00914A-s794 [file RA-011-D1RA00914A-s794.png]

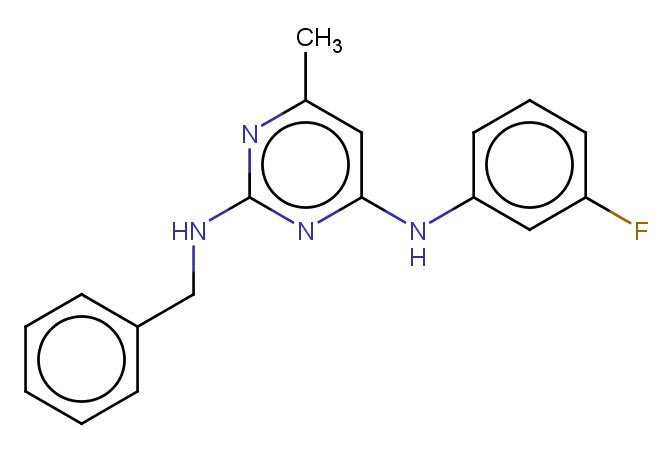

Supplement: RA-011-D1RA00914A-s795 [file RA-011-D1RA00914A-s795.png]

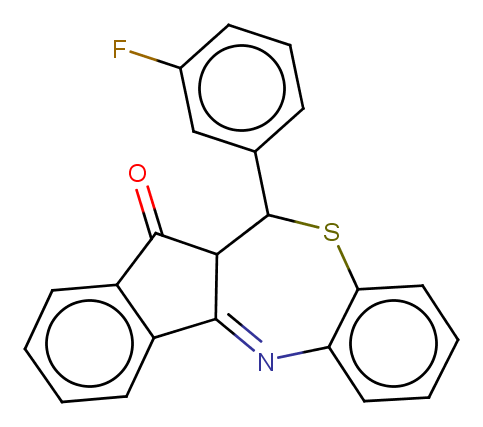

Supplement: RA-011-D1RA00914A-s796 [file RA-011-D1RA00914A-s796.png]

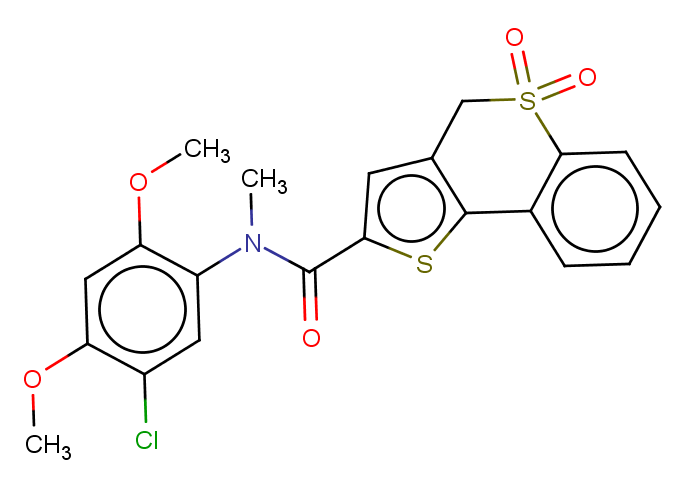

Supplement: RA-011-D1RA00914A-s797 [file RA-011-D1RA00914A-s797.png]

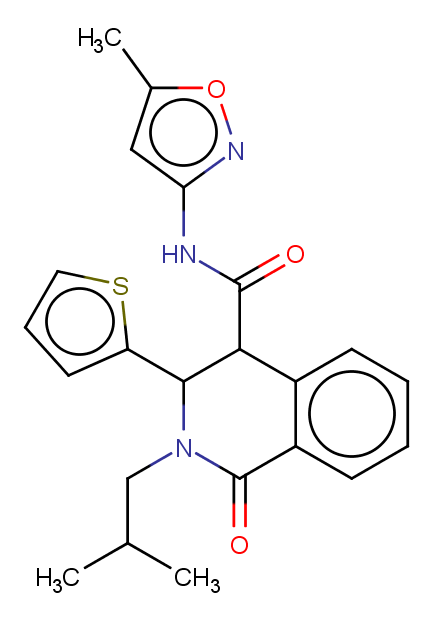

Supplement: RA-011-D1RA00914A-s798 [file RA-011-D1RA00914A-s798.png]

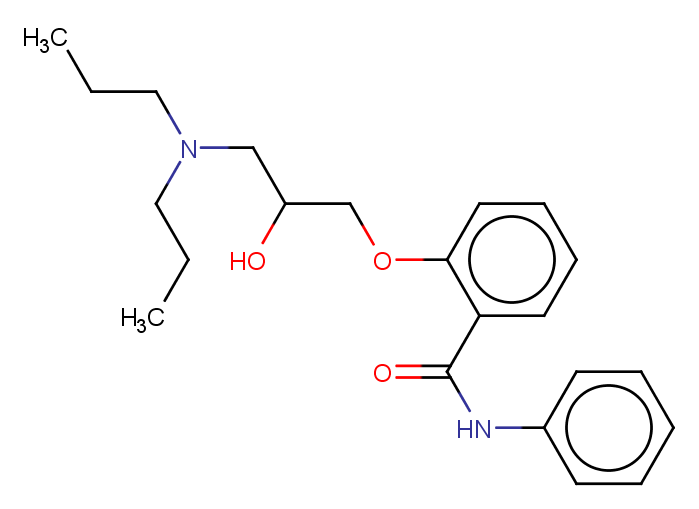

Supplement: RA-011-D1RA00914A-s799 [file RA-011-D1RA00914A-s799.png]

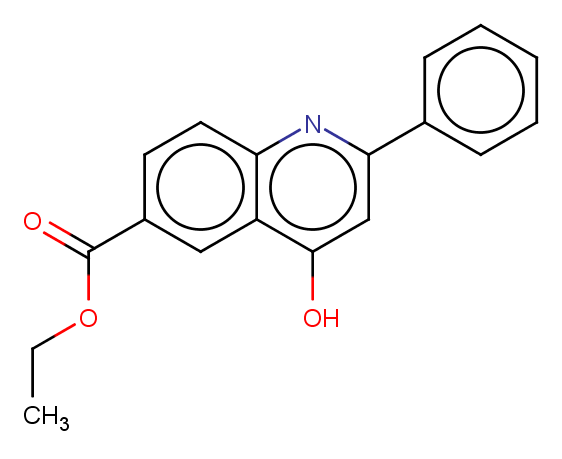

Supplement: RA-011-D1RA00914A-s800 [file RA-011-D1RA00914A-s800.png]

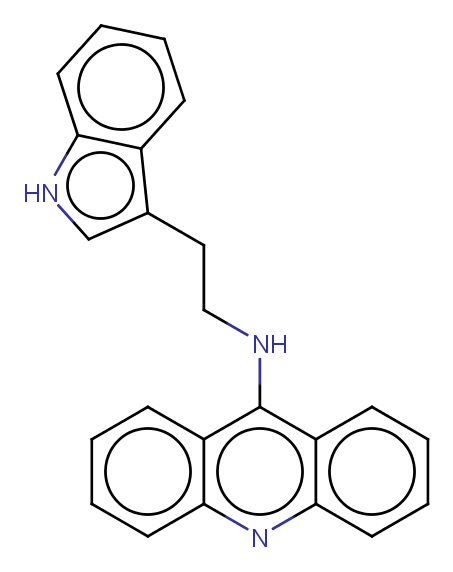

Supplement: RA-011-D1RA00914A-s801 [file RA-011-D1RA00914A-s801.png]

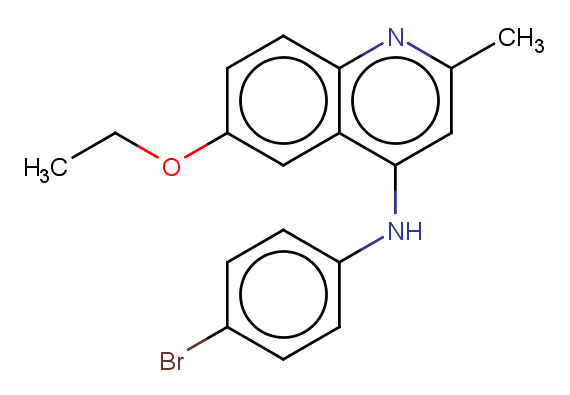

Supplement: RA-011-D1RA00914A-s802 [file RA-011-D1RA00914A-s802.png]

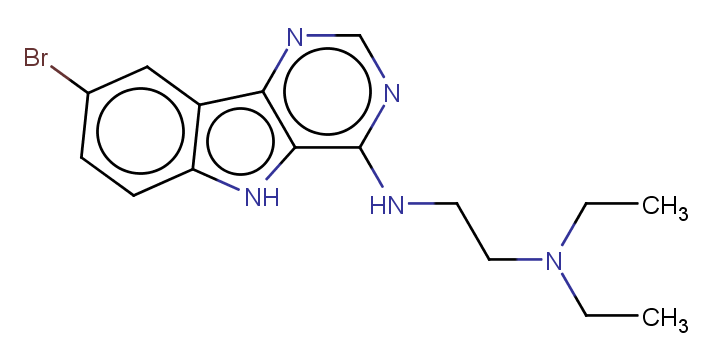

Supplement: RA-011-D1RA00914A-s803 [file RA-011-D1RA00914A-s803.png]

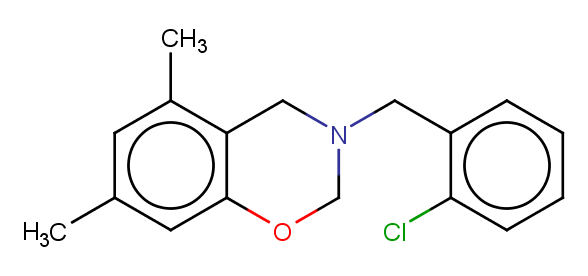

Supplement: RA-011-D1RA00914A-s804 [file RA-011-D1RA00914A-s804.png]

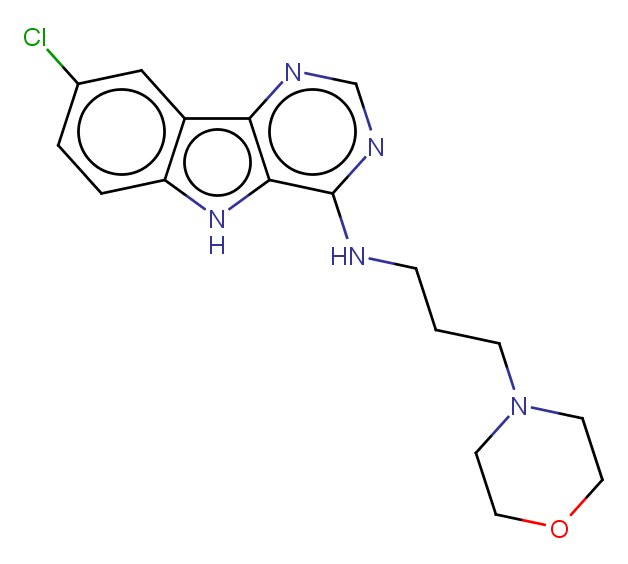

Supplement: RA-011-D1RA00914A-s805 [file RA-011-D1RA00914A-s805.png]

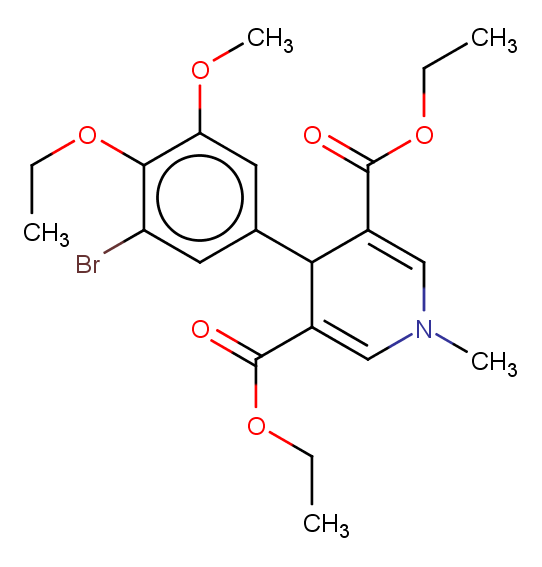

Supplement: RA-011-D1RA00914A-s806 [file RA-011-D1RA00914A-s806.png]

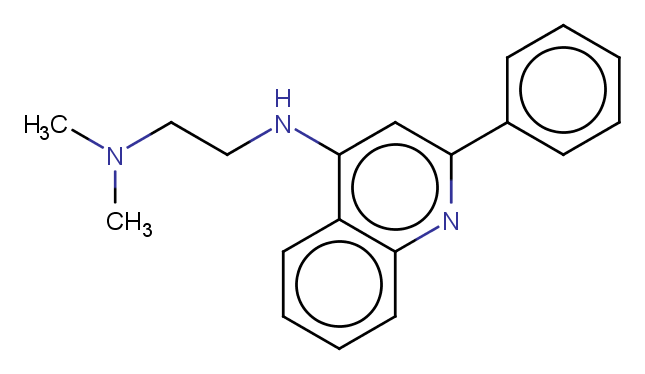

Supplement: RA-011-D1RA00914A-s807 [file RA-011-D1RA00914A-s807.png]

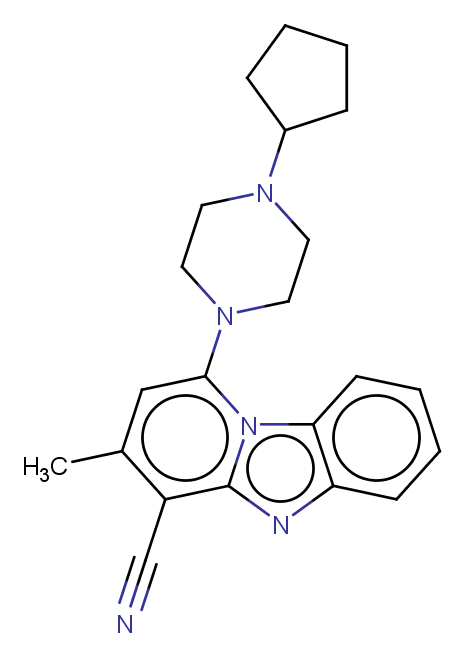

Supplement: RA-011-D1RA00914A-s808 [file RA-011-D1RA00914A-s808.png]

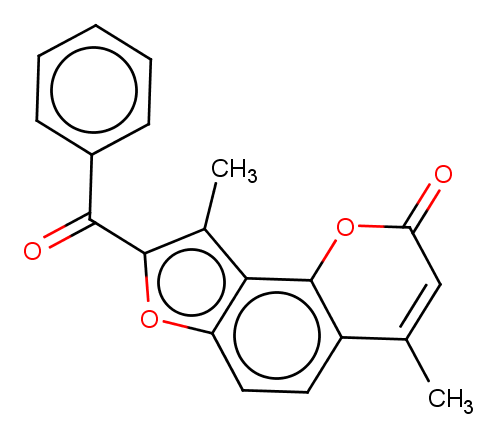

Supplement: RA-011-D1RA00914A-s809 [file RA-011-D1RA00914A-s809.png]

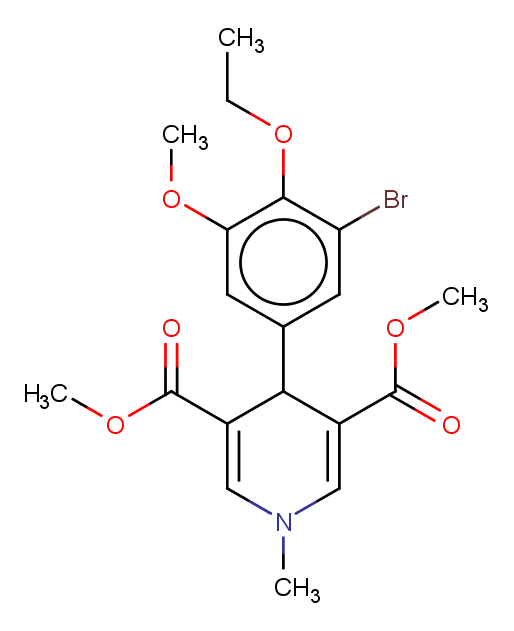

Supplement: RA-011-D1RA00914A-s810 [file RA-011-D1RA00914A-s810.png]

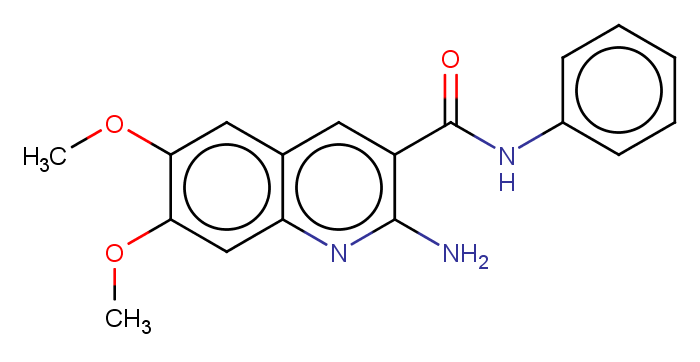

Supplement: RA-011-D1RA00914A-s811 [file RA-011-D1RA00914A-s811.png]

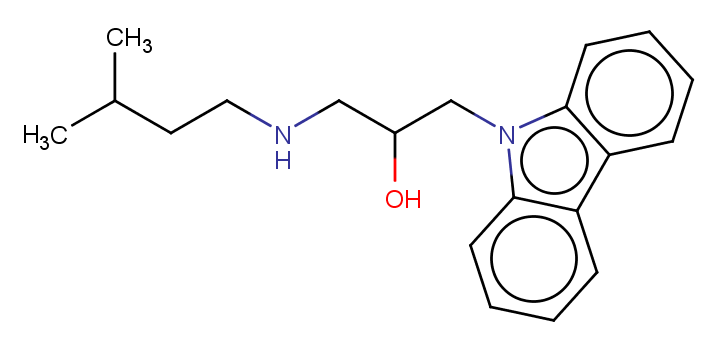

Supplement: RA-011-D1RA00914A-s812 [file RA-011-D1RA00914A-s812.png]

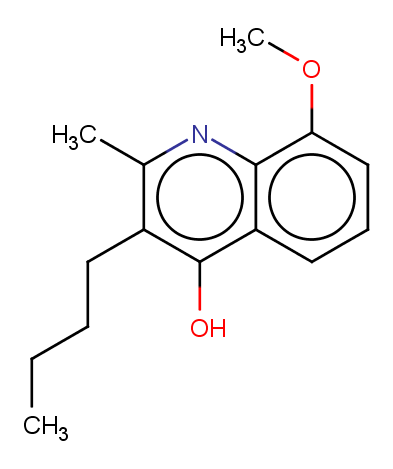

Supplement: RA-011-D1RA00914A-s813 [file RA-011-D1RA00914A-s813.png]

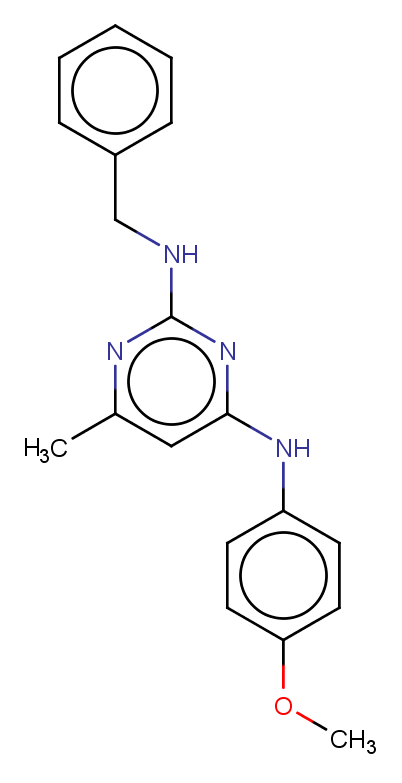

Supplement: RA-011-D1RA00914A-s814 [file RA-011-D1RA00914A-s814.png]

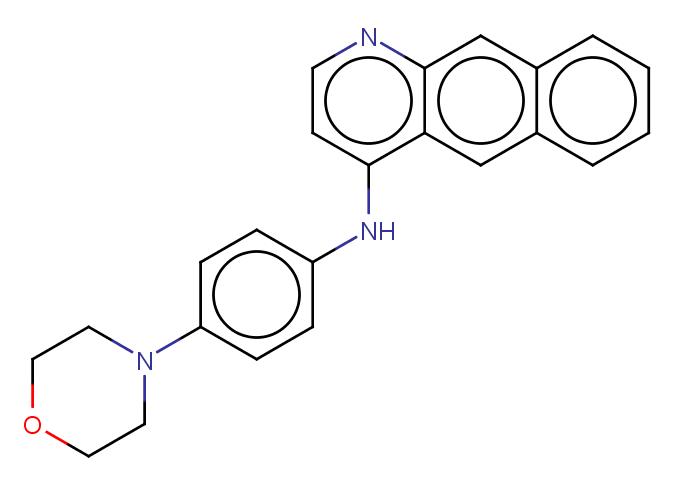

Supplement: RA-011-D1RA00914A-s815 [file RA-011-D1RA00914A-s815.png]

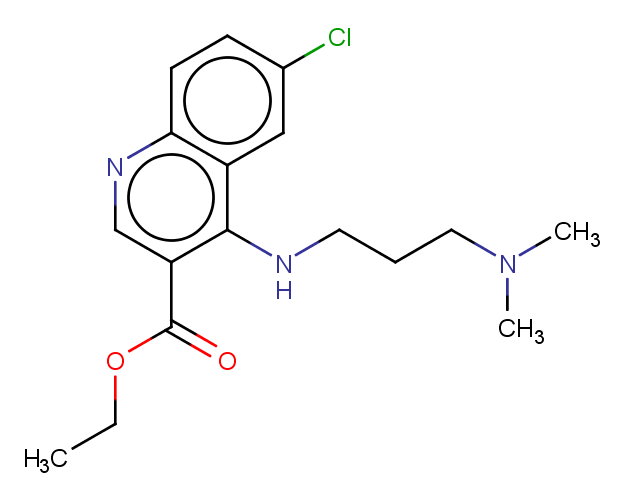

Supplement: RA-011-D1RA00914A-s816 [file RA-011-D1RA00914A-s816.png]

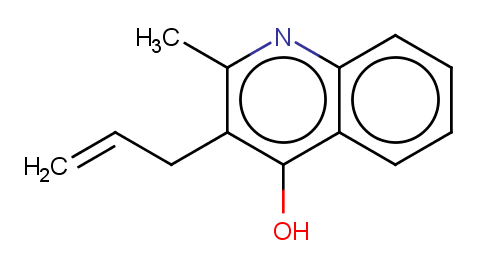

Supplement: RA-011-D1RA00914A-s817 [file RA-011-D1RA00914A-s817.png]

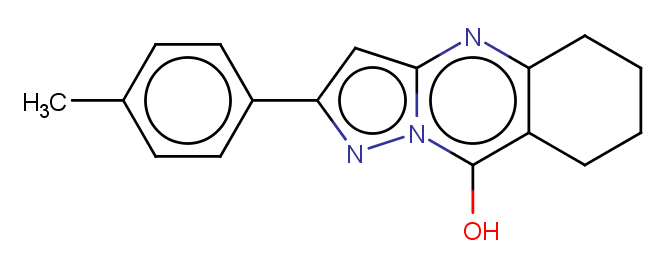

Supplement: RA-011-D1RA00914A-s818 [file RA-011-D1RA00914A-s818.png]

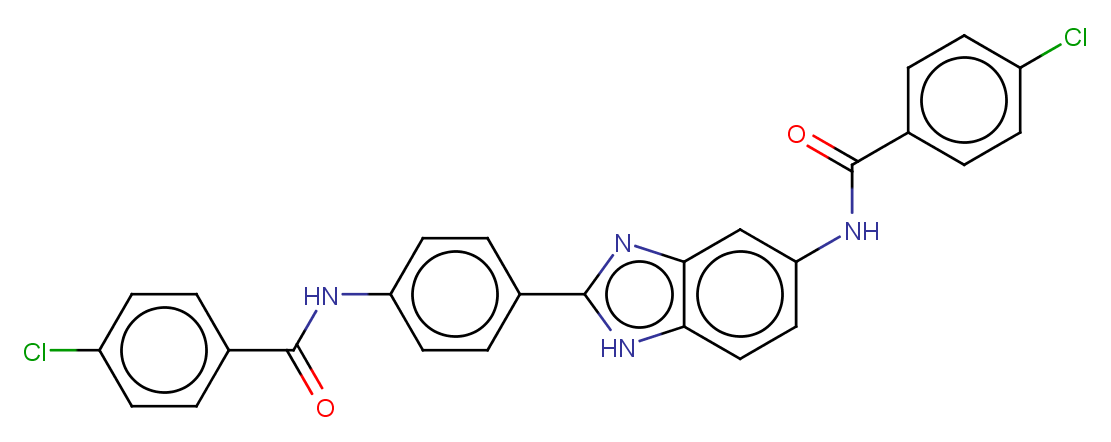

Supplement: RA-011-D1RA00914A-s819 [file RA-011-D1RA00914A-s819.png]

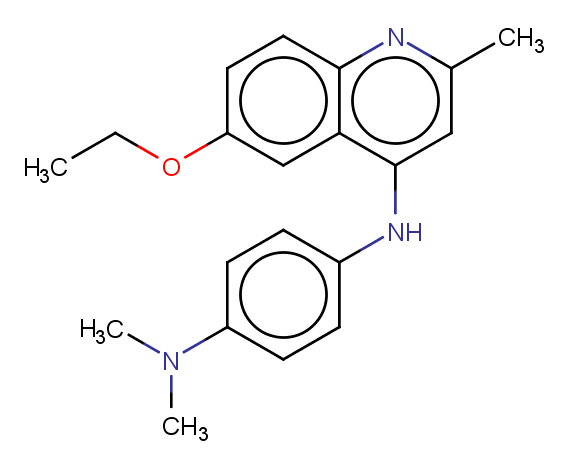

Supplement: RA-011-D1RA00914A-s820 [file RA-011-D1RA00914A-s820.png]

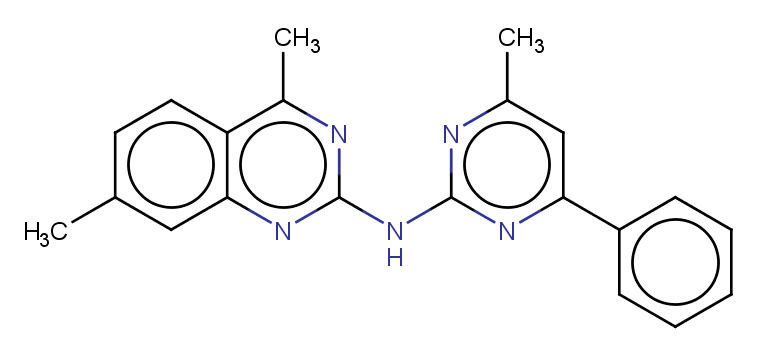

Supplement: RA-011-D1RA00914A-s821 [file RA-011-D1RA00914A-s821.png]

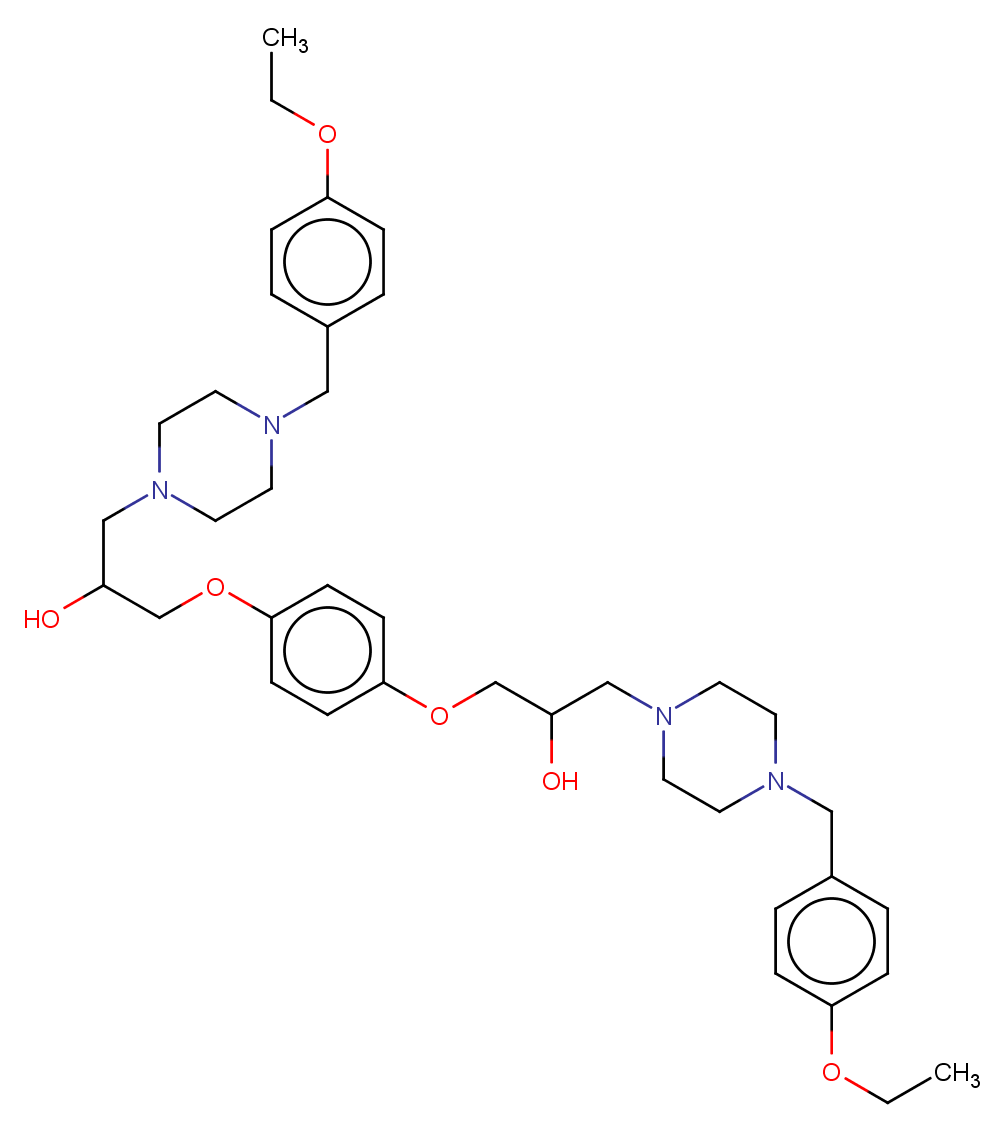

Supplement: RA-011-D1RA00914A-s822 [file RA-011-D1RA00914A-s822.png]

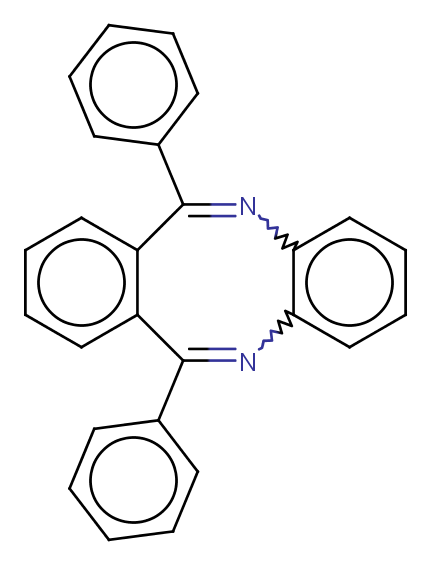

Supplement: RA-011-D1RA00914A-s823 [file RA-011-D1RA00914A-s823.png]

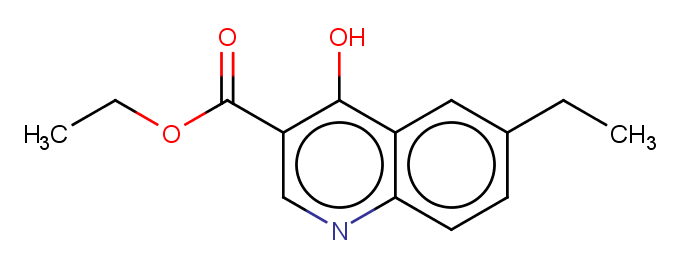

Supplement: RA-011-D1RA00914A-s824 [file RA-011-D1RA00914A-s824.png]

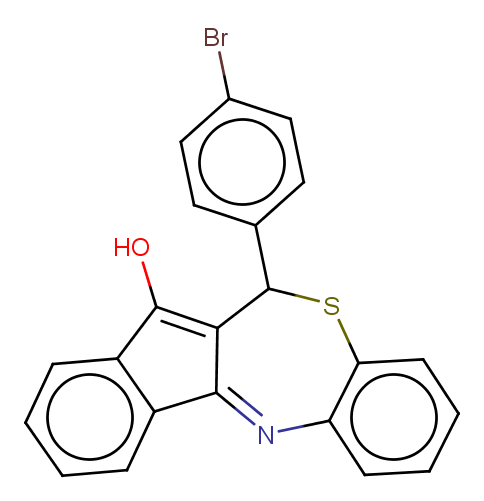

Supplement: RA-011-D1RA00914A-s825 [file RA-011-D1RA00914A-s825.png]

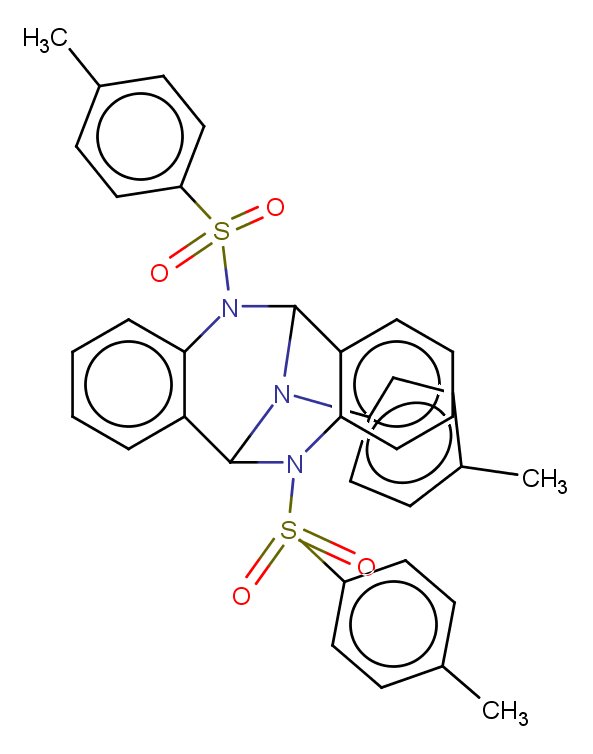

Supplement: RA-011-D1RA00914A-s826 [file RA-011-D1RA00914A-s826.png]

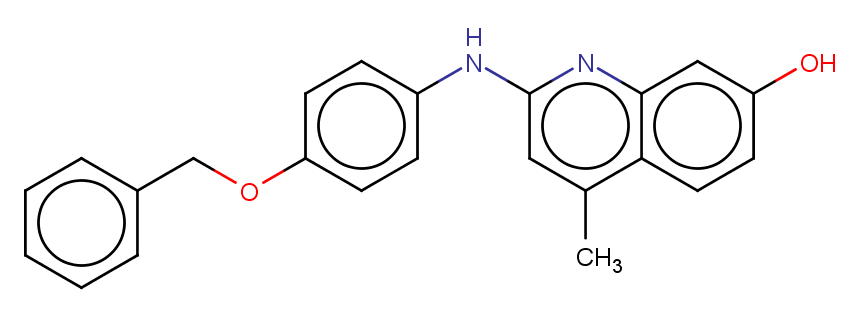

Supplement: RA-011-D1RA00914A-s827 [file RA-011-D1RA00914A-s827.png]

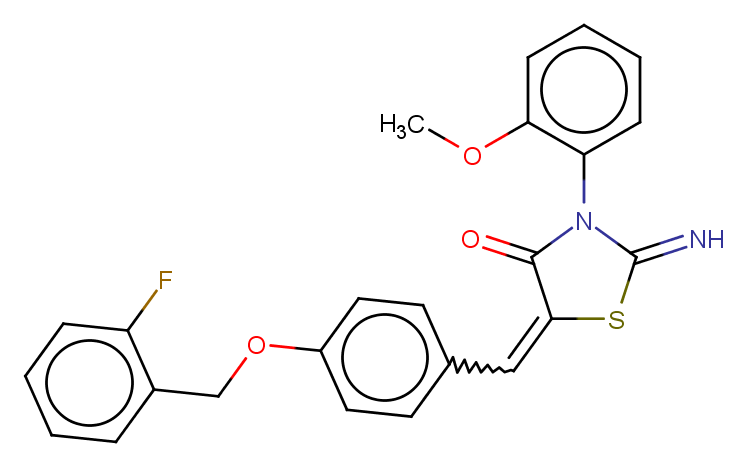

Supplement: RA-011-D1RA00914A-s828 [file RA-011-D1RA00914A-s828.png]

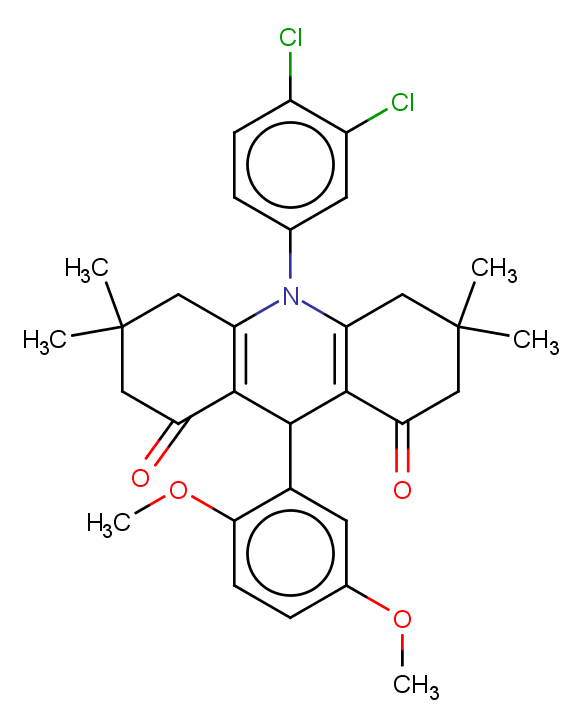

Supplement: RA-011-D1RA00914A-s829 [file RA-011-D1RA00914A-s829.png]

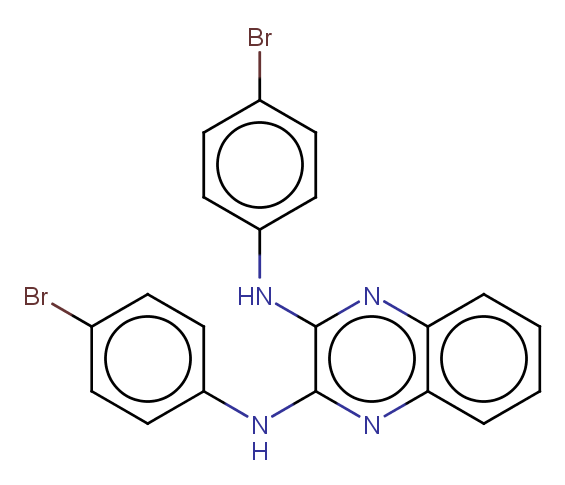

Supplement: RA-011-D1RA00914A-s830 [file RA-011-D1RA00914A-s830.png]

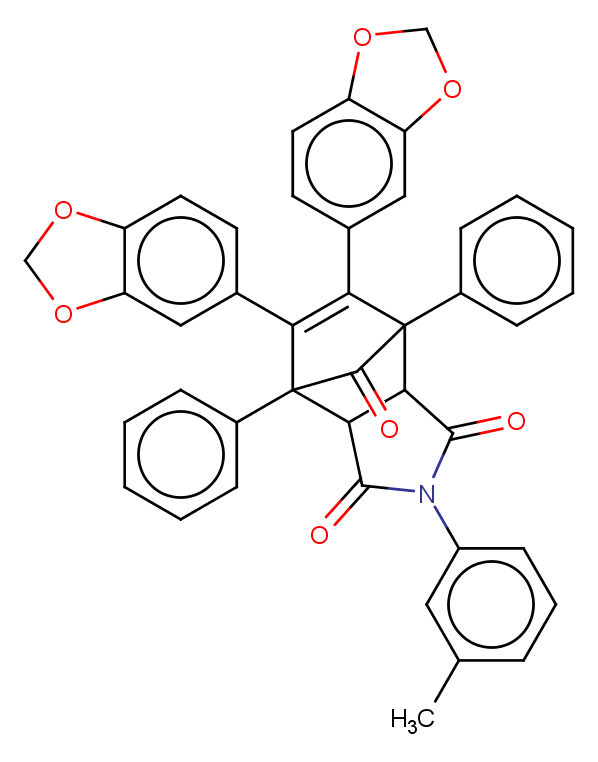

Supplement: RA-011-D1RA00914A-s831 [file RA-011-D1RA00914A-s831.png]

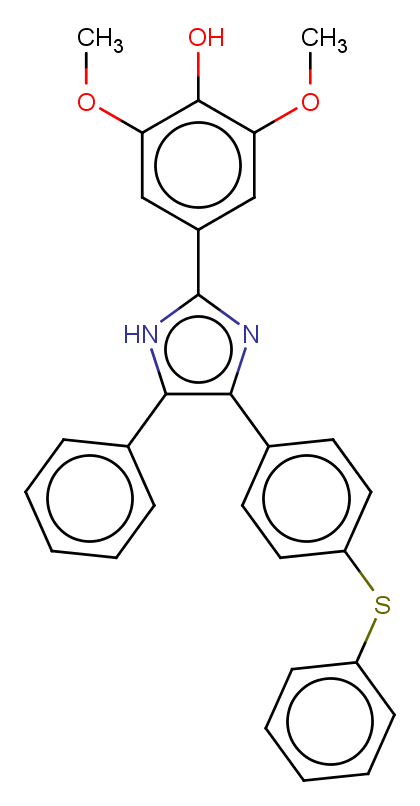

Supplement: RA-011-D1RA00914A-s832 [file RA-011-D1RA00914A-s832.png]

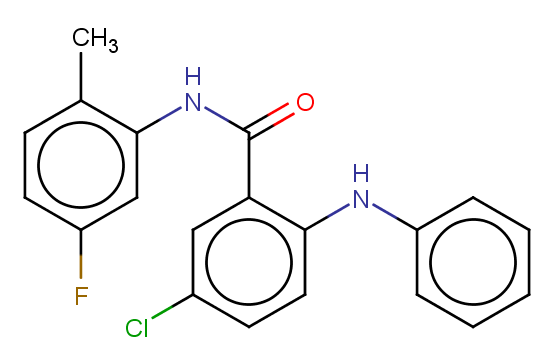

Supplement: RA-011-D1RA00914A-s833 [file RA-011-D1RA00914A-s833.png]

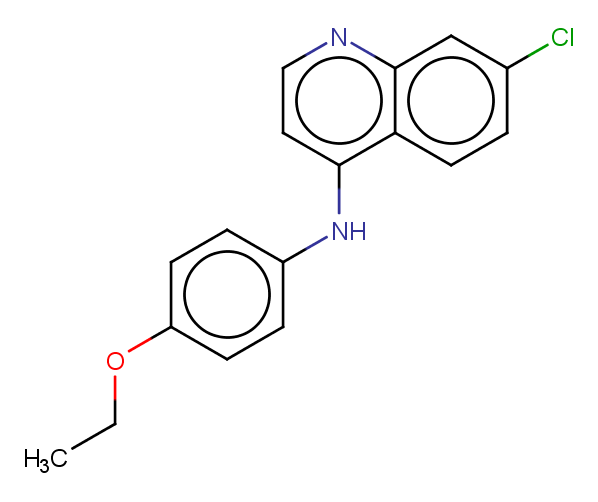

Supplement: RA-011-D1RA00914A-s834 [file RA-011-D1RA00914A-s834.png]

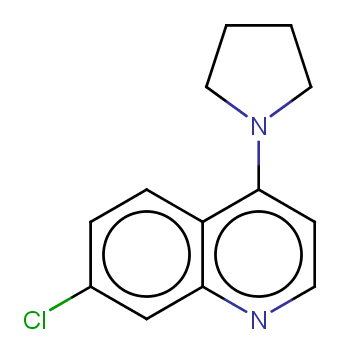

Supplement: RA-011-D1RA00914A-s835 [file RA-011-D1RA00914A-s835.png]

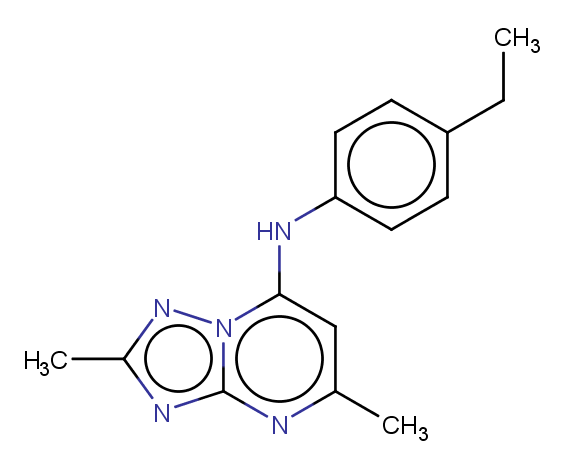

Supplement: RA-011-D1RA00914A-s836 [file RA-011-D1RA00914A-s836.png]

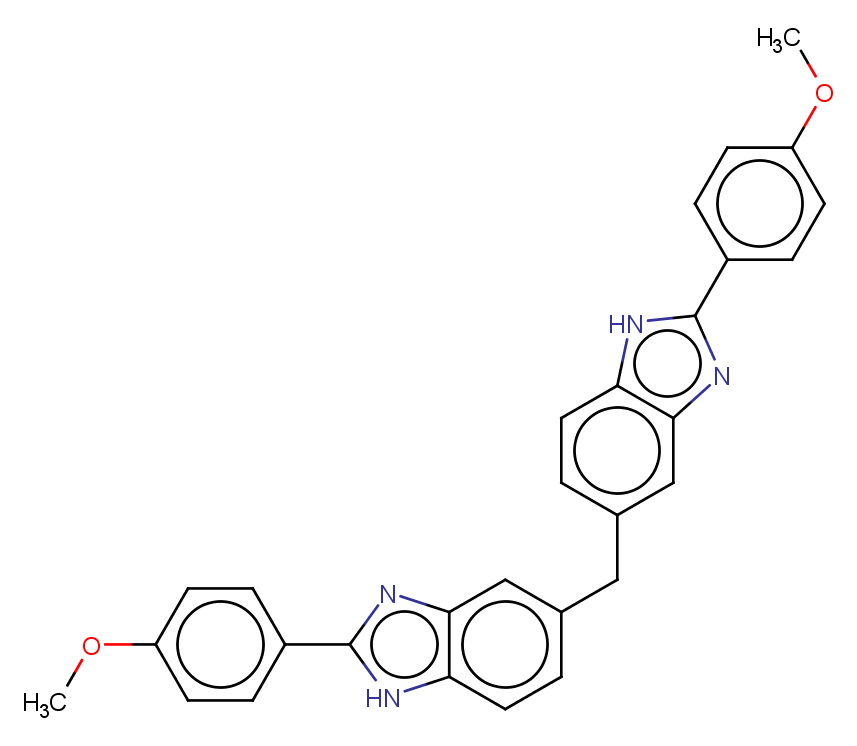

Supplement: RA-011-D1RA00914A-s837 [file RA-011-D1RA00914A-s837.png]

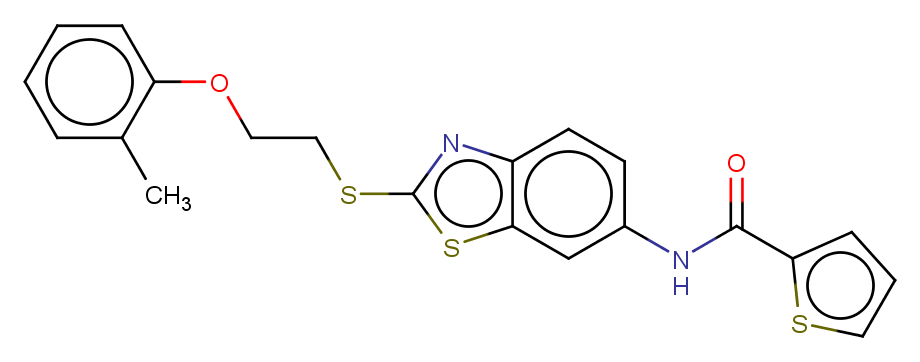

Supplement: RA-011-D1RA00914A-s838 [file RA-011-D1RA00914A-s838.png]

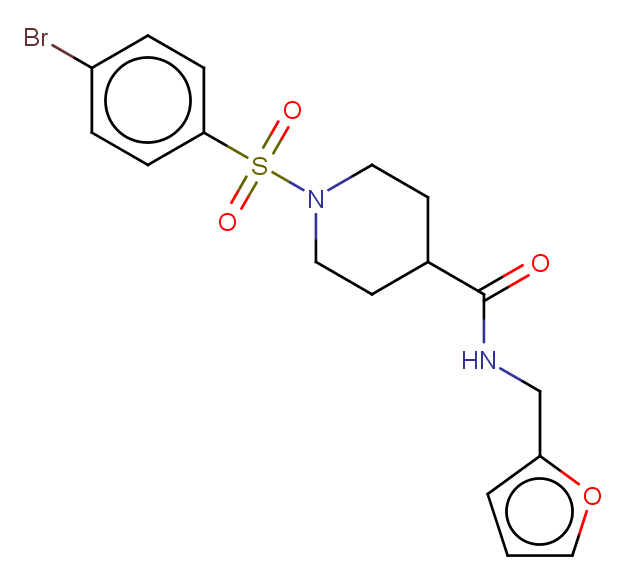

Supplement: RA-011-D1RA00914A-s839 [file RA-011-D1RA00914A-s839.png]

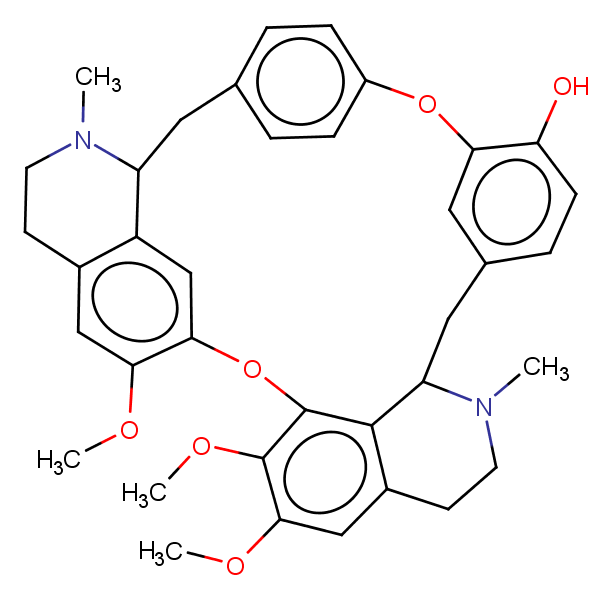

Supplement: RA-011-D1RA00914A-s840 [file RA-011-D1RA00914A-s840.png]

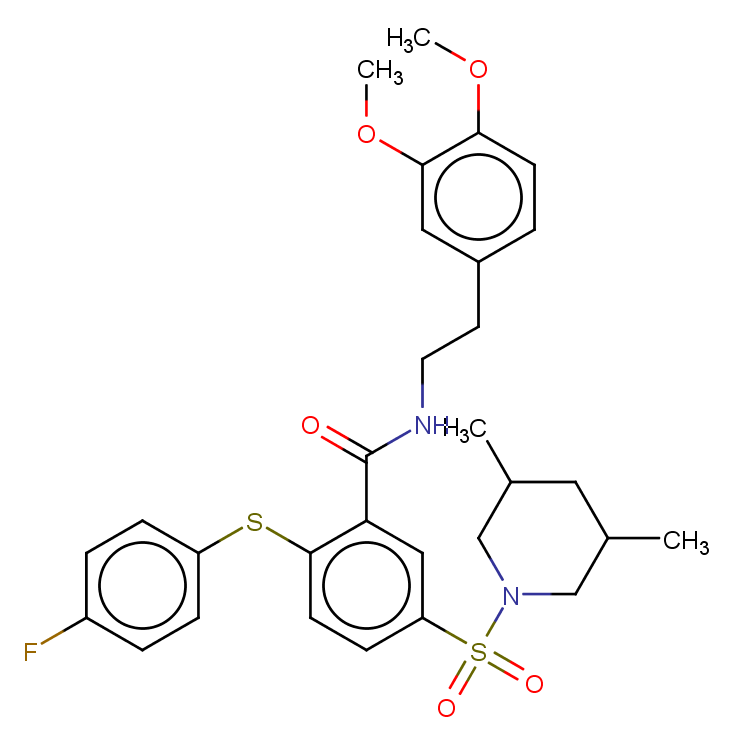

Supplement: RA-011-D1RA00914A-s841 [file RA-011-D1RA00914A-s841.png]

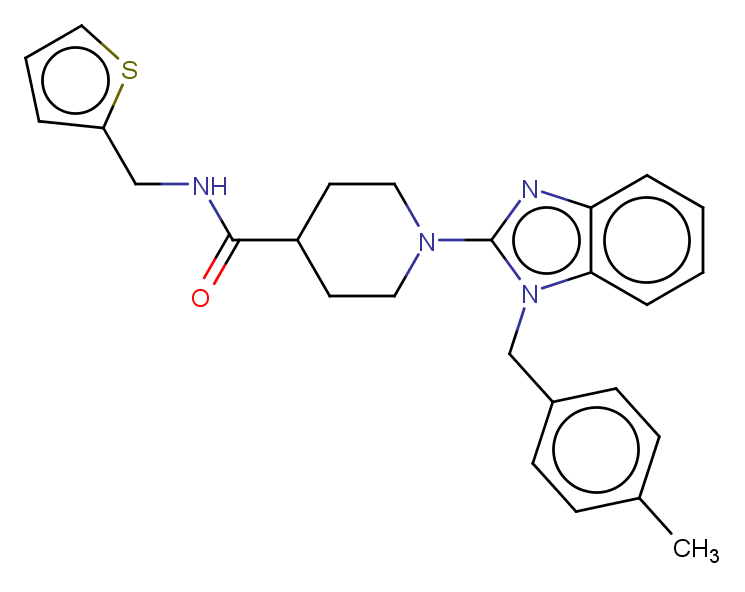

Supplement: RA-011-D1RA00914A-s842 [file RA-011-D1RA00914A-s842.png]

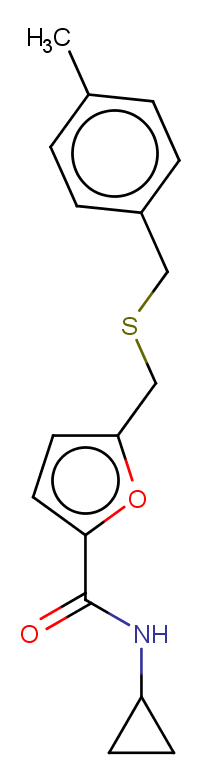

Supplement: RA-011-D1RA00914A-s843 [file RA-011-D1RA00914A-s843.png]

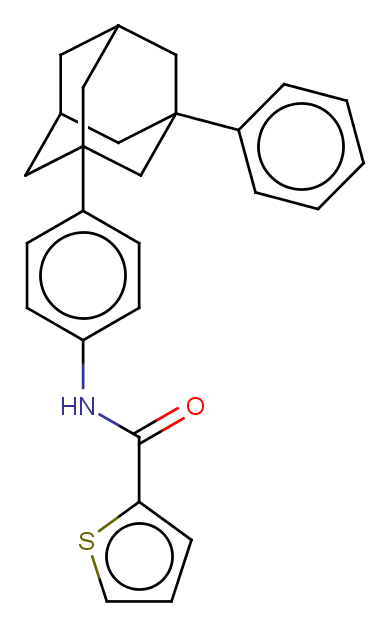

Supplement: RA-011-D1RA00914A-s844 [file RA-011-D1RA00914A-s844.png]

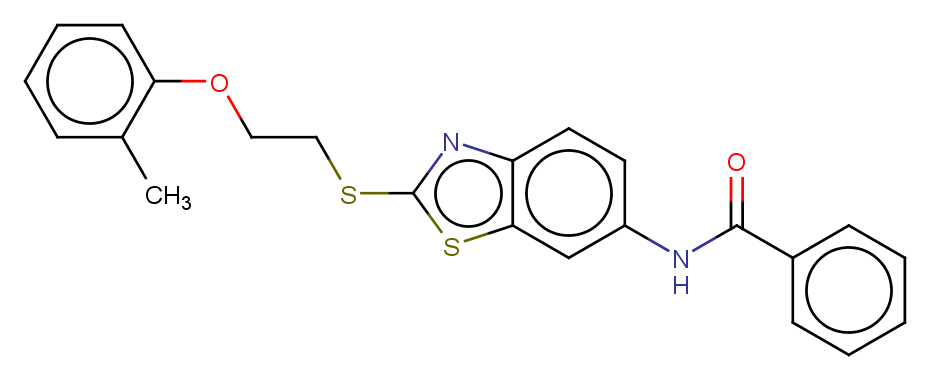

Supplement: RA-011-D1RA00914A-s845 [file RA-011-D1RA00914A-s845.png]

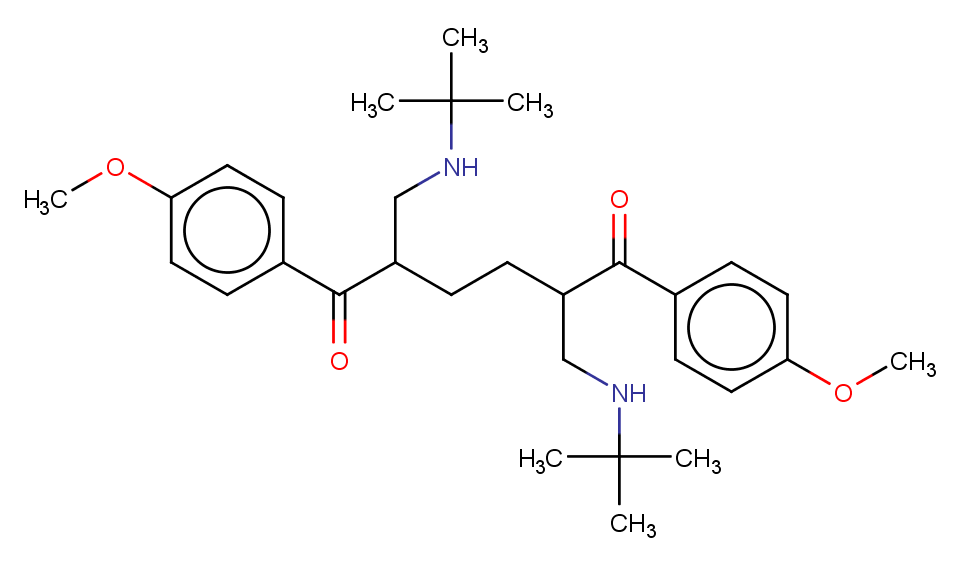

Supplement: RA-011-D1RA00914A-s846 [file RA-011-D1RA00914A-s846.png]

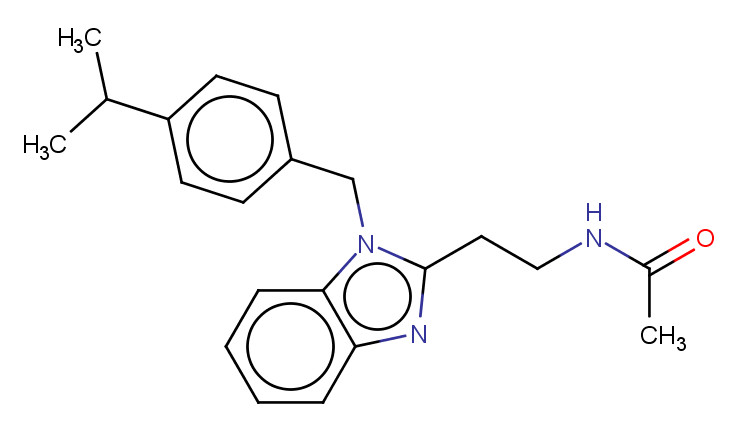

Supplement: RA-011-D1RA00914A-s847 [file RA-011-D1RA00914A-s847.png]

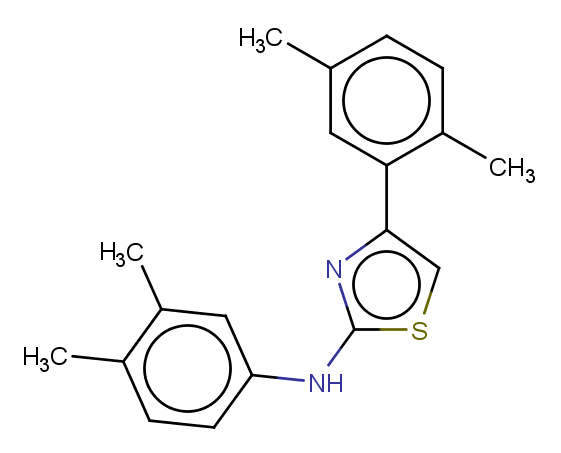

Supplement: RA-011-D1RA00914A-s848 [file RA-011-D1RA00914A-s848.png]

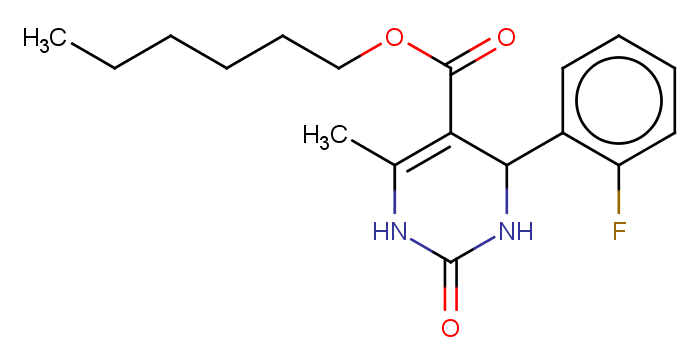

Supplement: RA-011-D1RA00914A-s849 [file RA-011-D1RA00914A-s849.png]

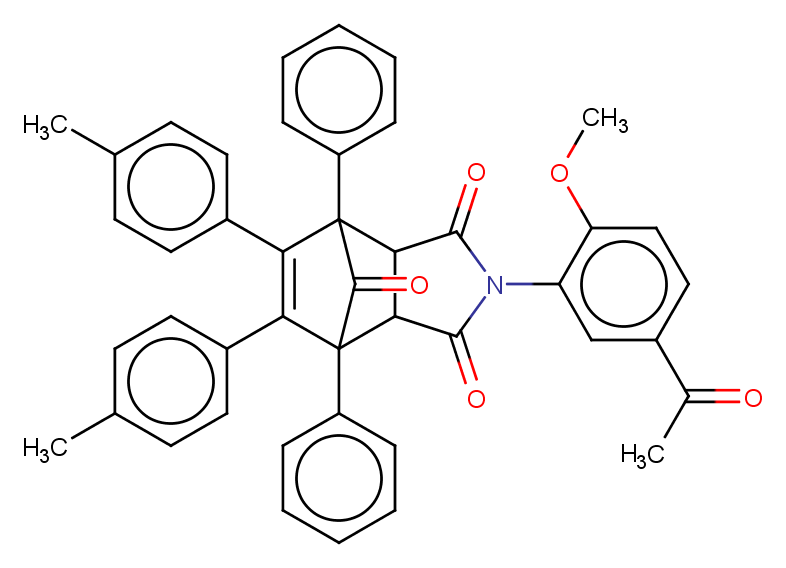

Supplement: RA-011-D1RA00914A-s850 [file RA-011-D1RA00914A-s850.png]

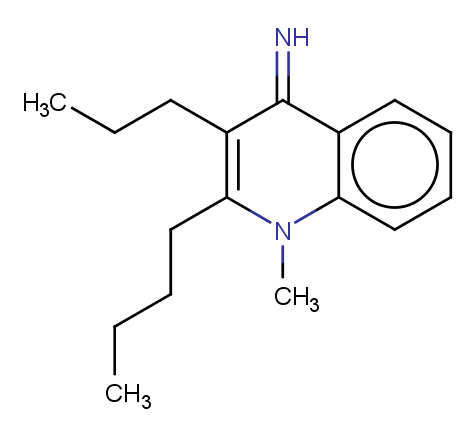

Supplement: RA-011-D1RA00914A-s851 [file RA-011-D1RA00914A-s851.png]

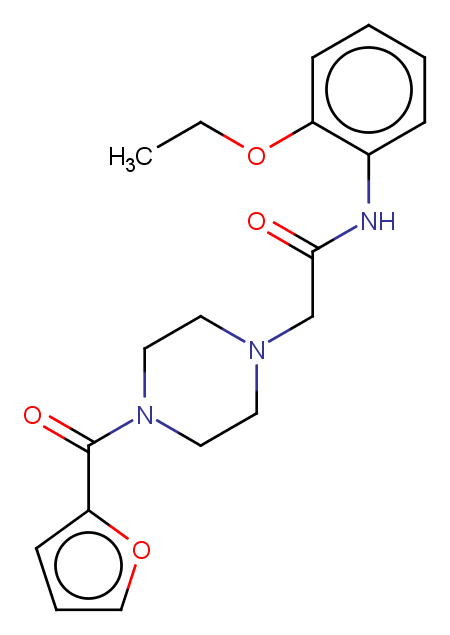

Supplement: RA-011-D1RA00914A-s852 [file RA-011-D1RA00914A-s852.png]

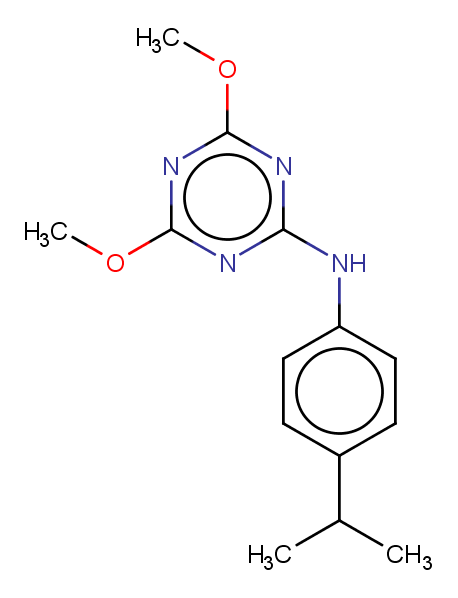

Supplement: RA-011-D1RA00914A-s853 [file RA-011-D1RA00914A-s853.png]

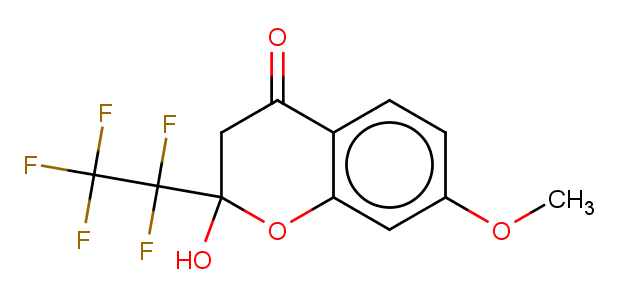

Supplement: RA-011-D1RA00914A-s854 [file RA-011-D1RA00914A-s854.png]

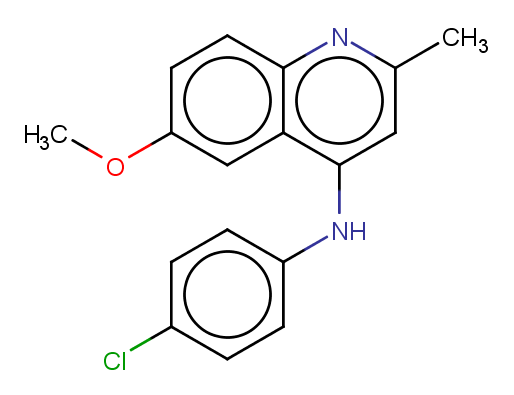

Supplement: RA-011-D1RA00914A-s855 [file RA-011-D1RA00914A-s855.png]

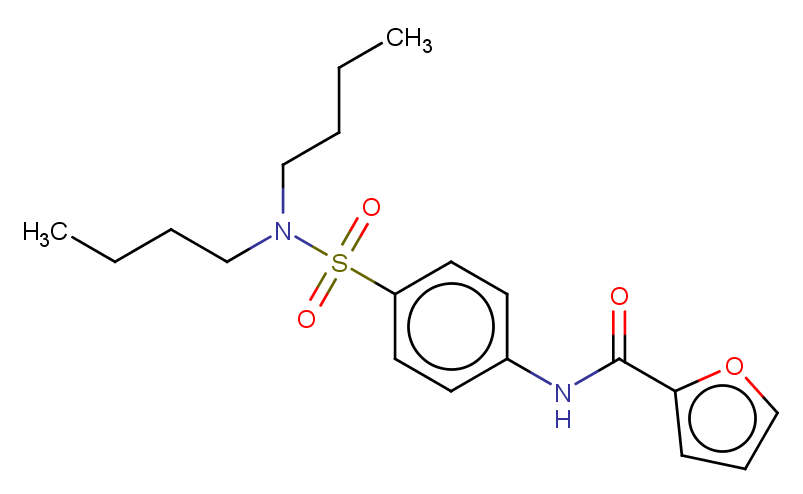

Supplement: RA-011-D1RA00914A-s856 [file RA-011-D1RA00914A-s856.png]

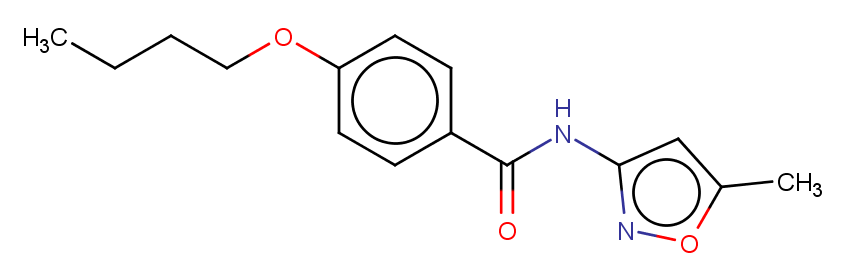

Supplement: RA-011-D1RA00914A-s857 [file RA-011-D1RA00914A-s857.png]

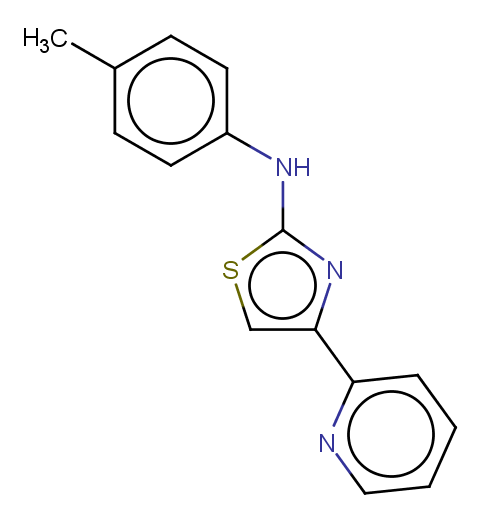

Supplement: RA-011-D1RA00914A-s858 [file RA-011-D1RA00914A-s858.png]

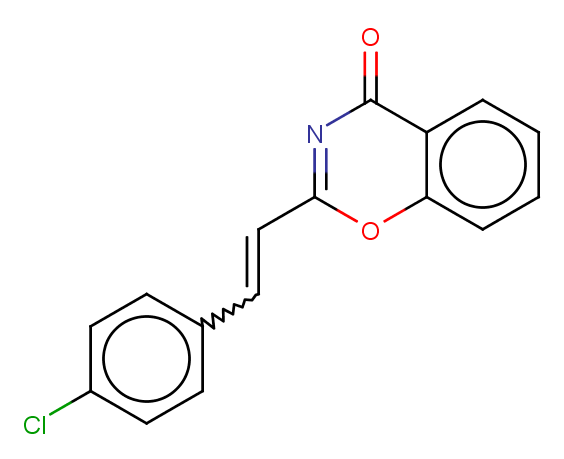

Supplement: RA-011-D1RA00914A-s859 [file RA-011-D1RA00914A-s859.png]

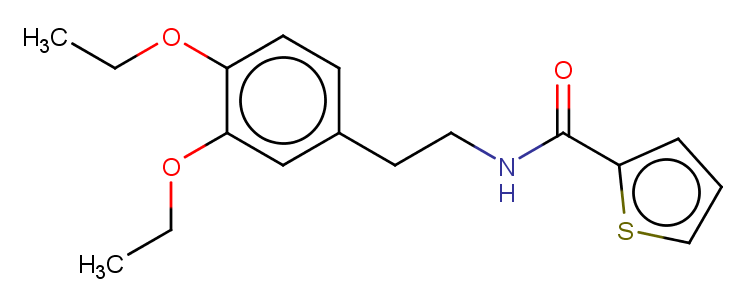

Supplement: RA-011-D1RA00914A-s860 [file RA-011-D1RA00914A-s860.png]

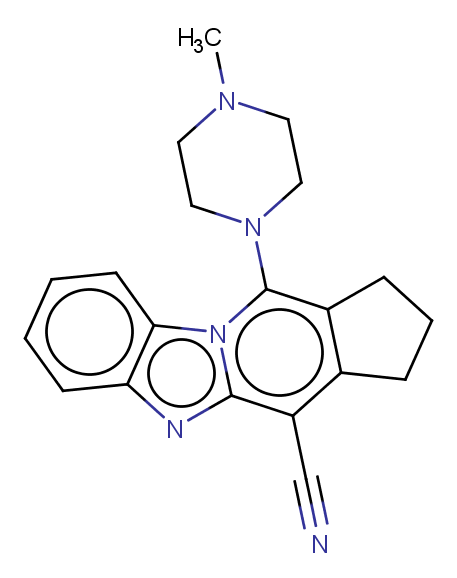

Supplement: RA-011-D1RA00914A-s861 [file RA-011-D1RA00914A-s861.png]

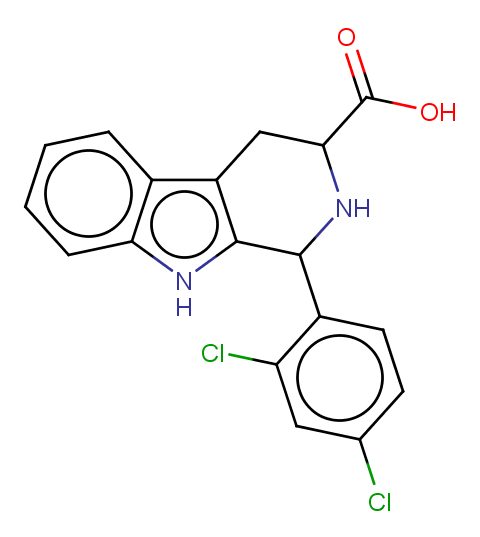

Supplement: RA-011-D1RA00914A-s862 [file RA-011-D1RA00914A-s862.png]

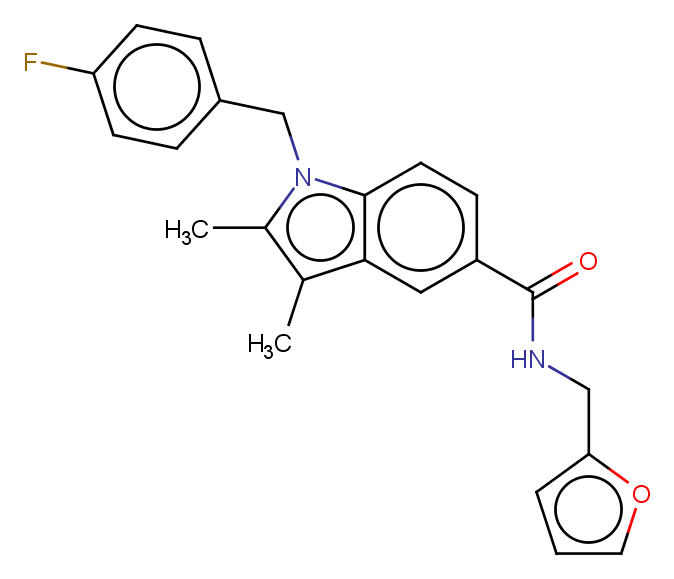

Supplement: RA-011-D1RA00914A-s863 [file RA-011-D1RA00914A-s863.png]

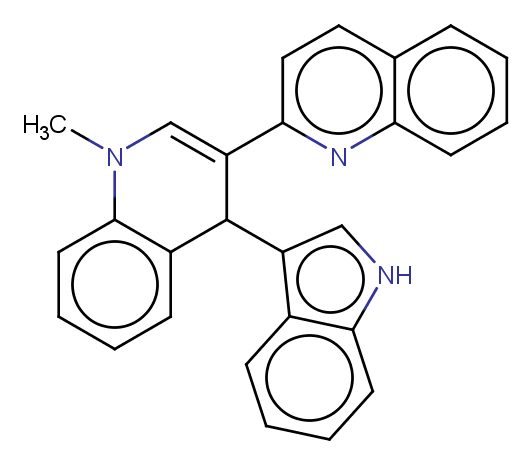

Supplement: RA-011-D1RA00914A-s864 [file RA-011-D1RA00914A-s864.png]

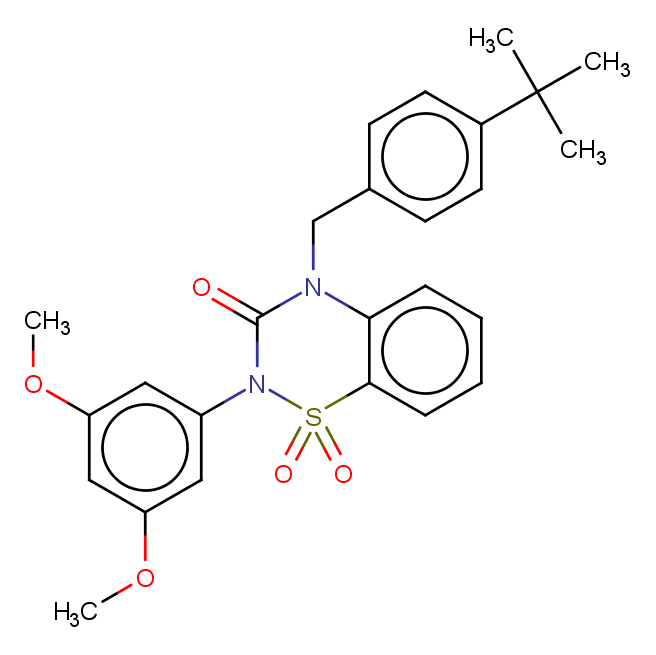

Supplement: RA-011-D1RA00914A-s865 [file RA-011-D1RA00914A-s865.png]

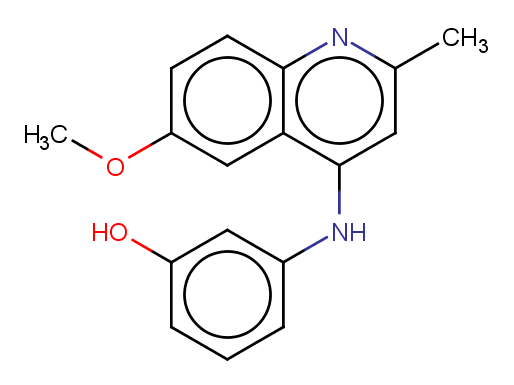

Supplement: RA-011-D1RA00914A-s866 [file RA-011-D1RA00914A-s866.png]

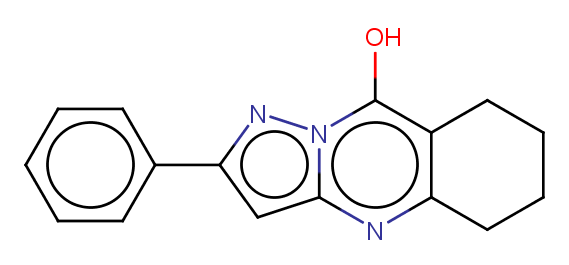

Supplement: RA-011-D1RA00914A-s867 [file RA-011-D1RA00914A-s867.png]

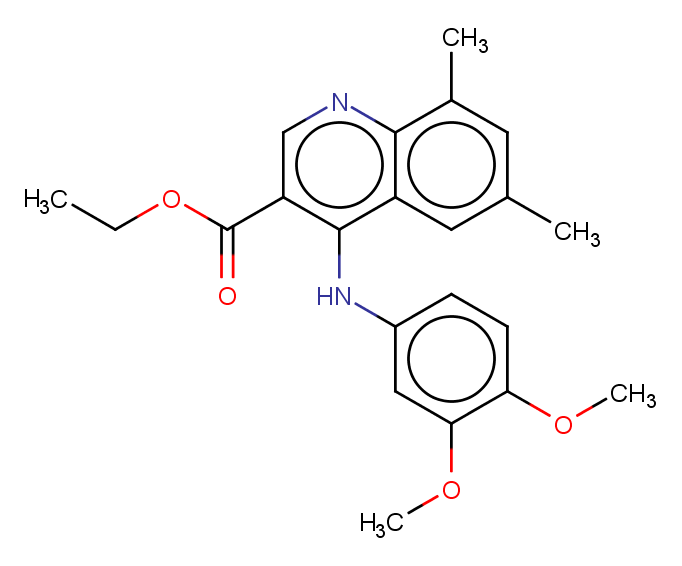

Supplement: RA-011-D1RA00914A-s868 [file RA-011-D1RA00914A-s868.png]

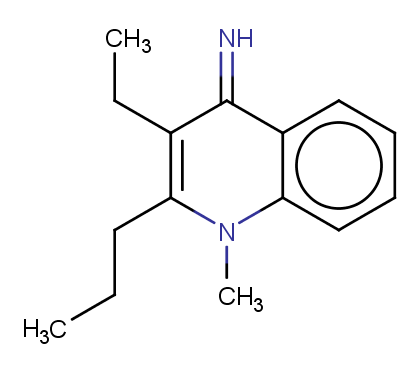

Supplement: RA-011-D1RA00914A-s869 [file RA-011-D1RA00914A-s869.png]

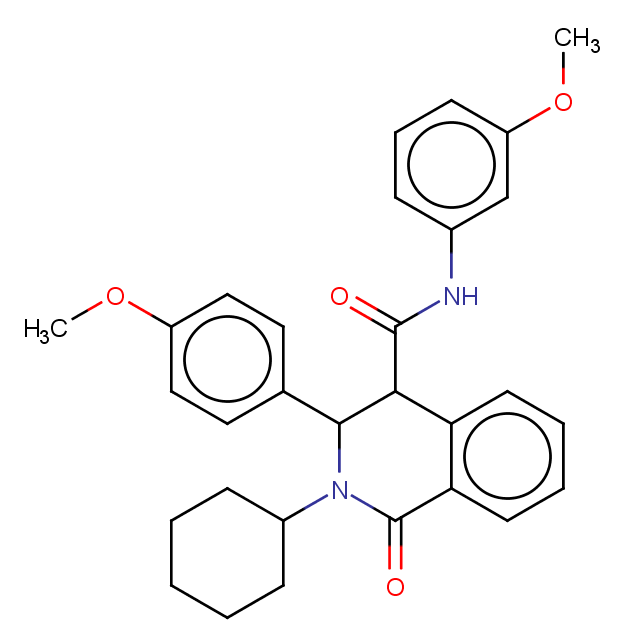

Supplement: RA-011-D1RA00914A-s870 [file RA-011-D1RA00914A-s870.png]

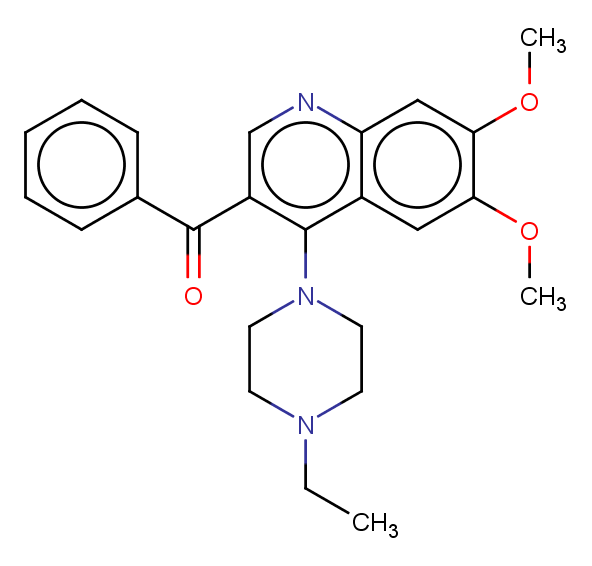

Supplement: RA-011-D1RA00914A-s871 [file RA-011-D1RA00914A-s871.png]
