# Supplementary figures and images for: Improved herbicide discovery using physico-chemical rules refined by antimalarial library screening (part 6 of 14)
Source: RSC Adv. 2021 Feb 23;11(15):8459–67. doi: 10.1039/d1ra00914a (PMC8695207; doi:10.1039/d1ra00914a)

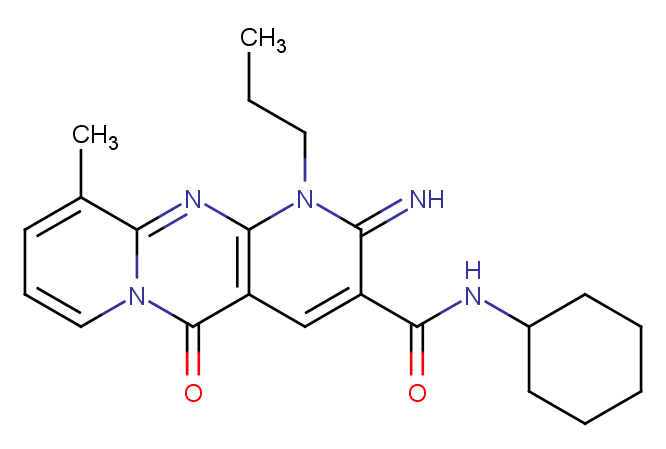

Supplement: RA-011-D1RA00914A-s872 [file RA-011-D1RA00914A-s872.png]

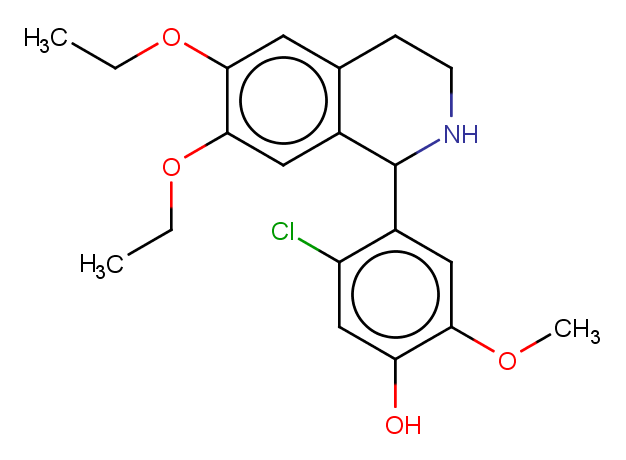

Supplement: RA-011-D1RA00914A-s873 [file RA-011-D1RA00914A-s873.png]

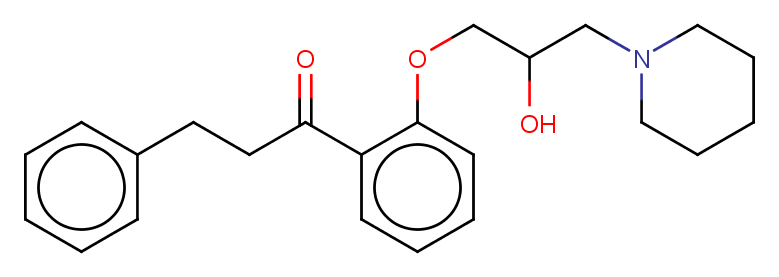

Supplement: RA-011-D1RA00914A-s874 [file RA-011-D1RA00914A-s874.png]

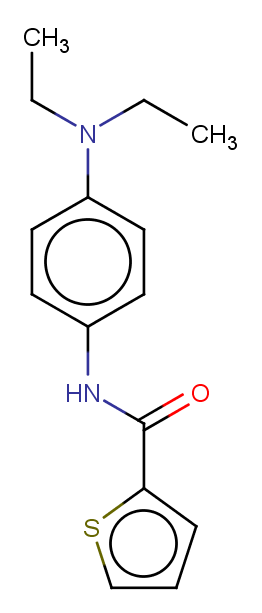

Supplement: RA-011-D1RA00914A-s875 [file RA-011-D1RA00914A-s875.png]

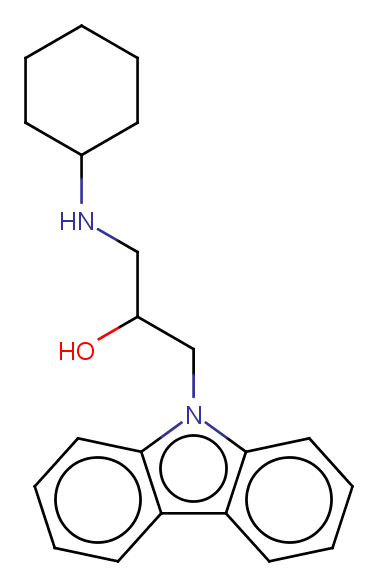

Supplement: RA-011-D1RA00914A-s876 [file RA-011-D1RA00914A-s876.png]

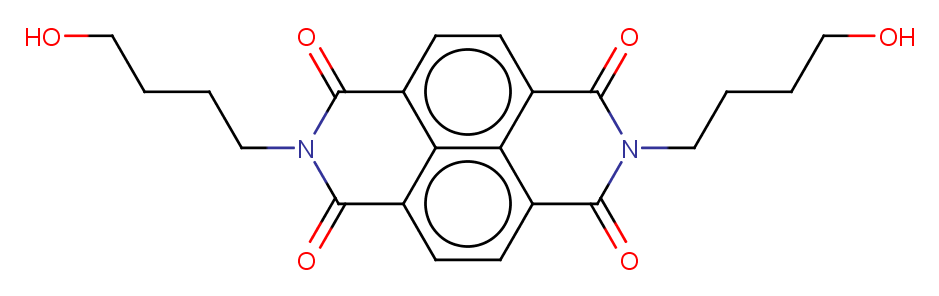

Supplement: RA-011-D1RA00914A-s877 [file RA-011-D1RA00914A-s877.png]

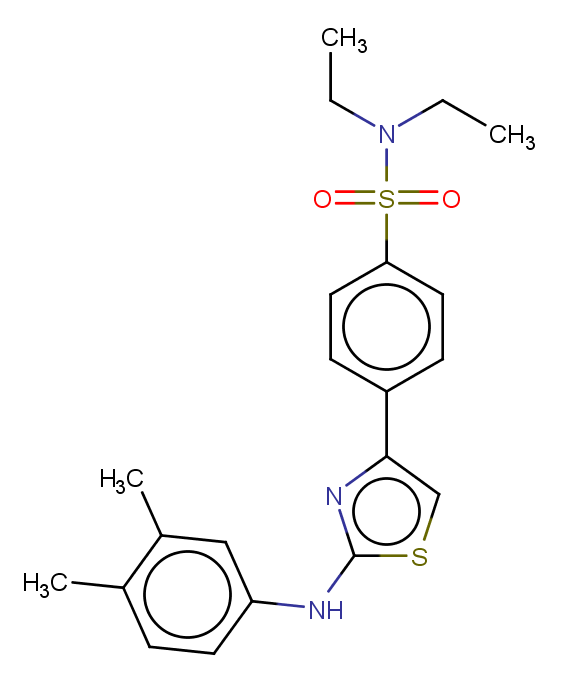

Supplement: RA-011-D1RA00914A-s878 [file RA-011-D1RA00914A-s878.png]

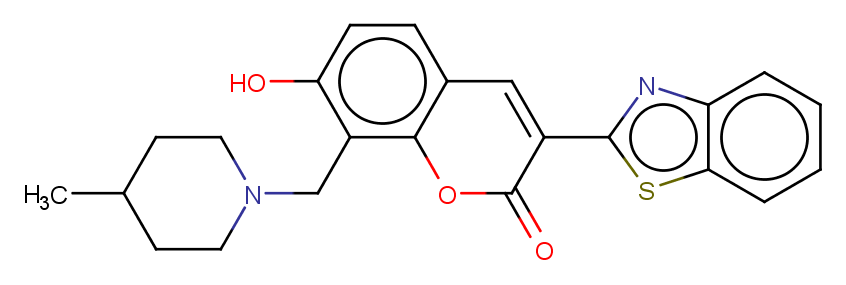

Supplement: RA-011-D1RA00914A-s879 [file RA-011-D1RA00914A-s879.png]

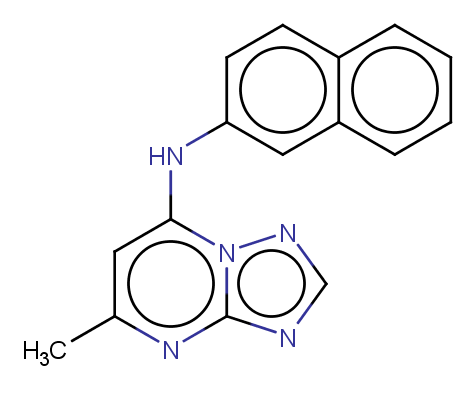

Supplement: RA-011-D1RA00914A-s880 [file RA-011-D1RA00914A-s880.png]

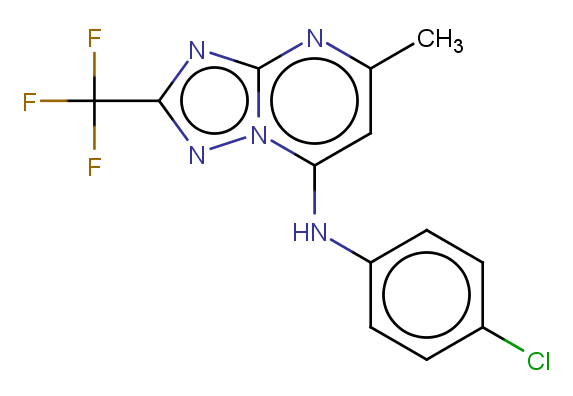

Supplement: RA-011-D1RA00914A-s881 [file RA-011-D1RA00914A-s881.png]

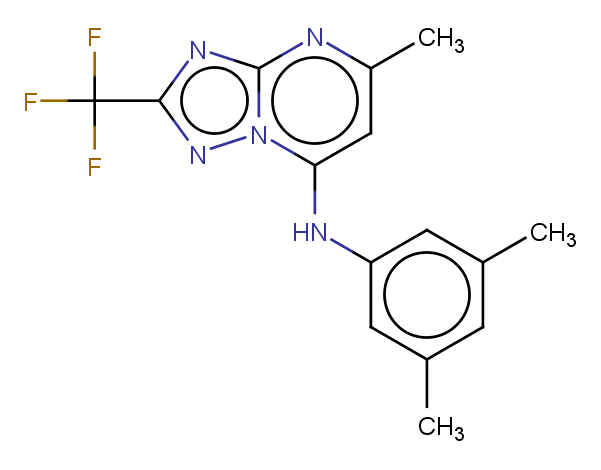

Supplement: RA-011-D1RA00914A-s882 [file RA-011-D1RA00914A-s882.png]

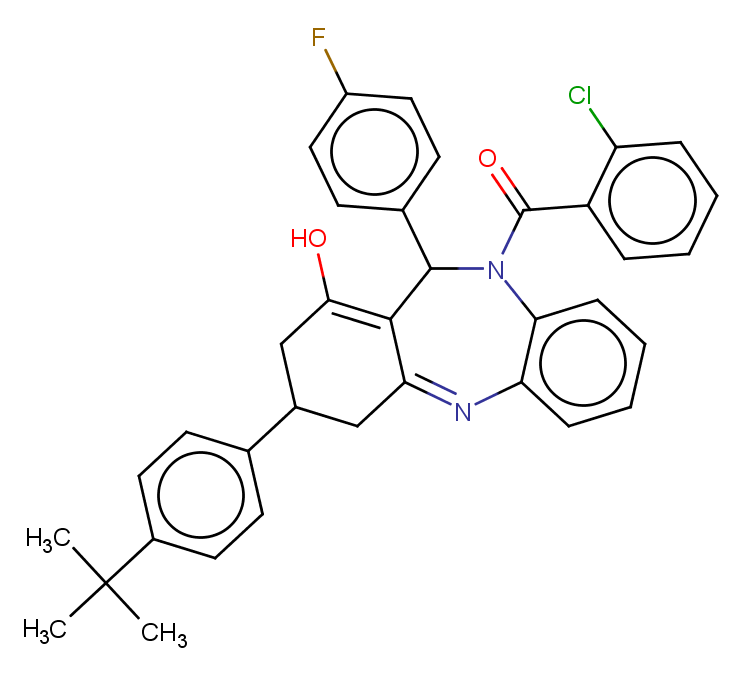

Supplement: RA-011-D1RA00914A-s883 [file RA-011-D1RA00914A-s883.png]

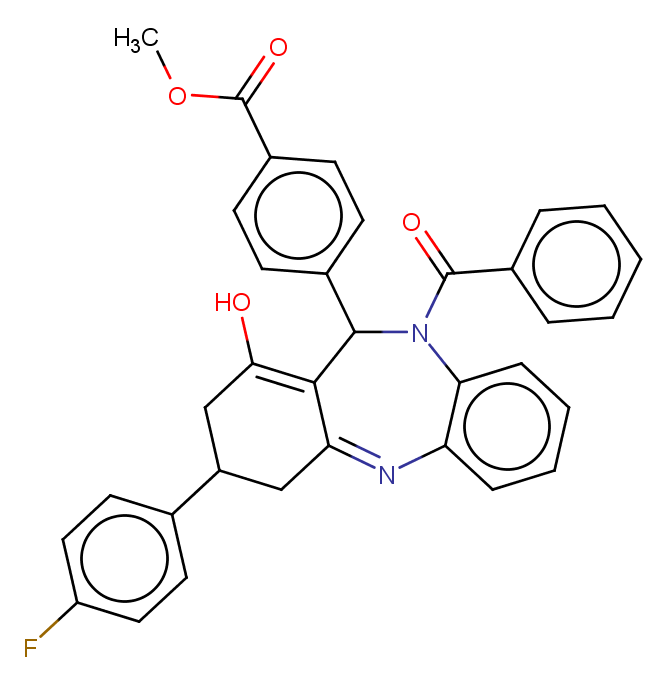

Supplement: RA-011-D1RA00914A-s884 [file RA-011-D1RA00914A-s884.png]

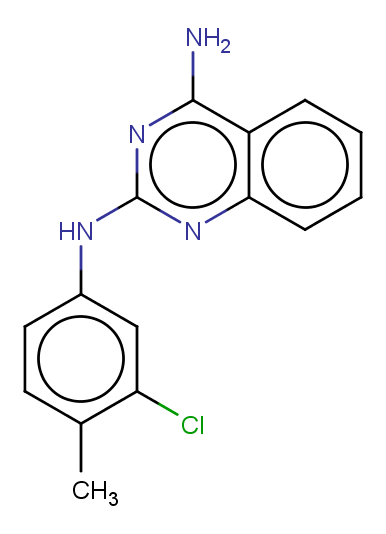

Supplement: RA-011-D1RA00914A-s885 [file RA-011-D1RA00914A-s885.png]

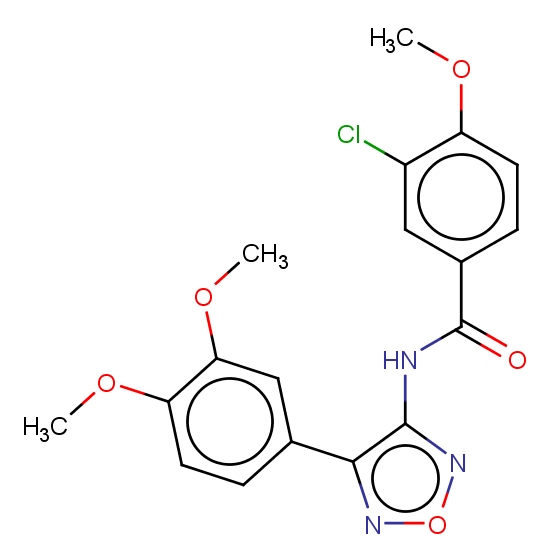

Supplement: RA-011-D1RA00914A-s886 [file RA-011-D1RA00914A-s886.png]

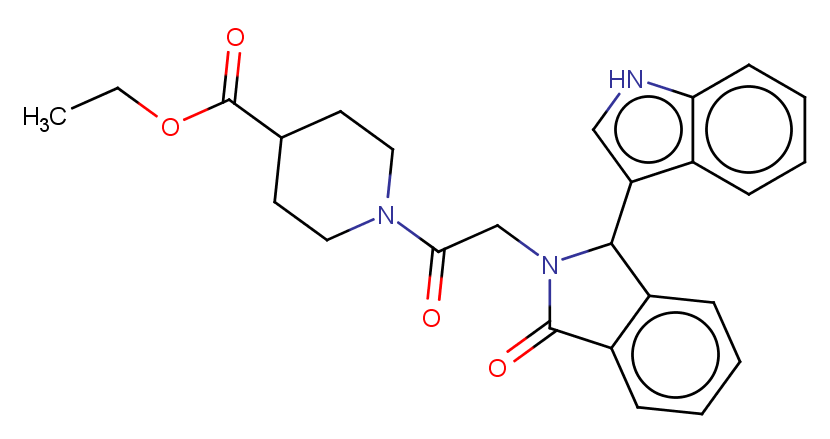

Supplement: RA-011-D1RA00914A-s887 [file RA-011-D1RA00914A-s887.png]

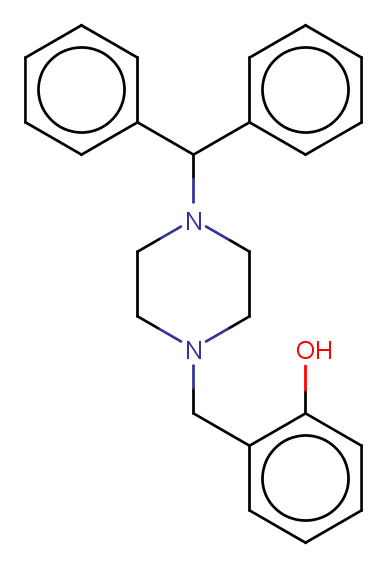

Supplement: RA-011-D1RA00914A-s888 [file RA-011-D1RA00914A-s888.png]

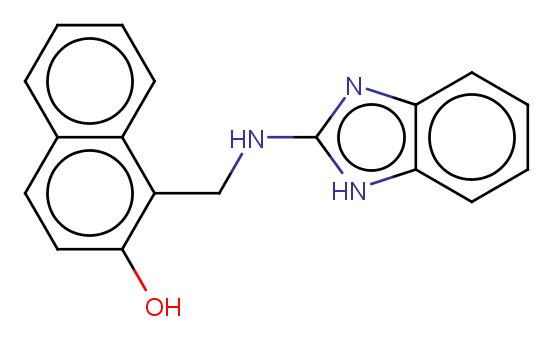

Supplement: RA-011-D1RA00914A-s889 [file RA-011-D1RA00914A-s889.png]

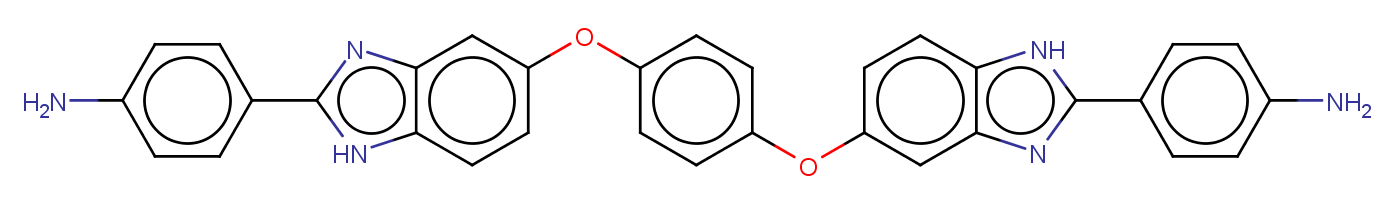

Supplement: RA-011-D1RA00914A-s890 [file RA-011-D1RA00914A-s890.png]

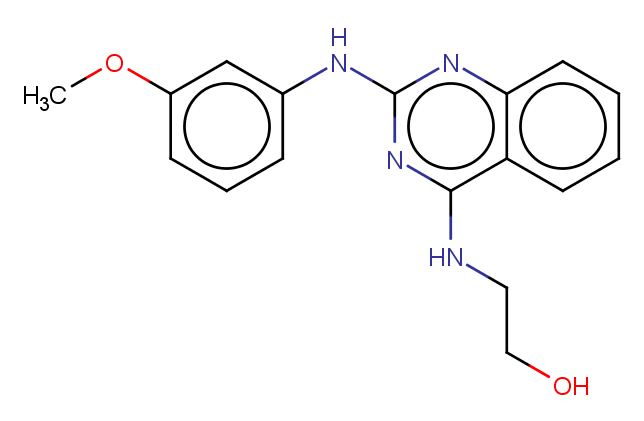

Supplement: RA-011-D1RA00914A-s891 [file RA-011-D1RA00914A-s891.png]

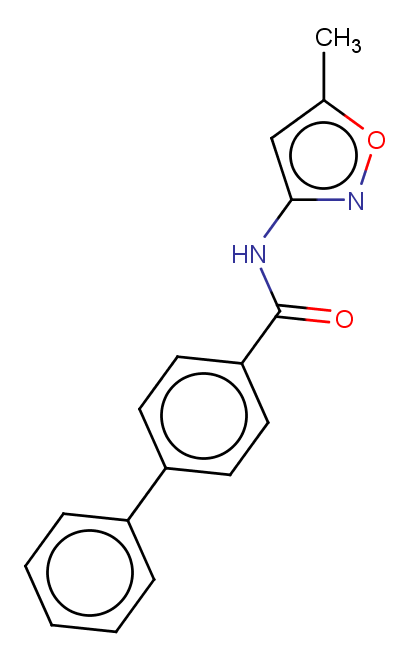

Supplement: RA-011-D1RA00914A-s892 [file RA-011-D1RA00914A-s892.png]

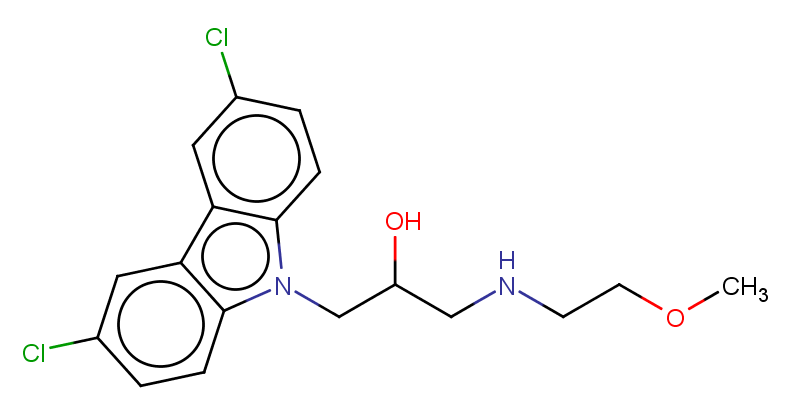

Supplement: RA-011-D1RA00914A-s893 [file RA-011-D1RA00914A-s893.png]

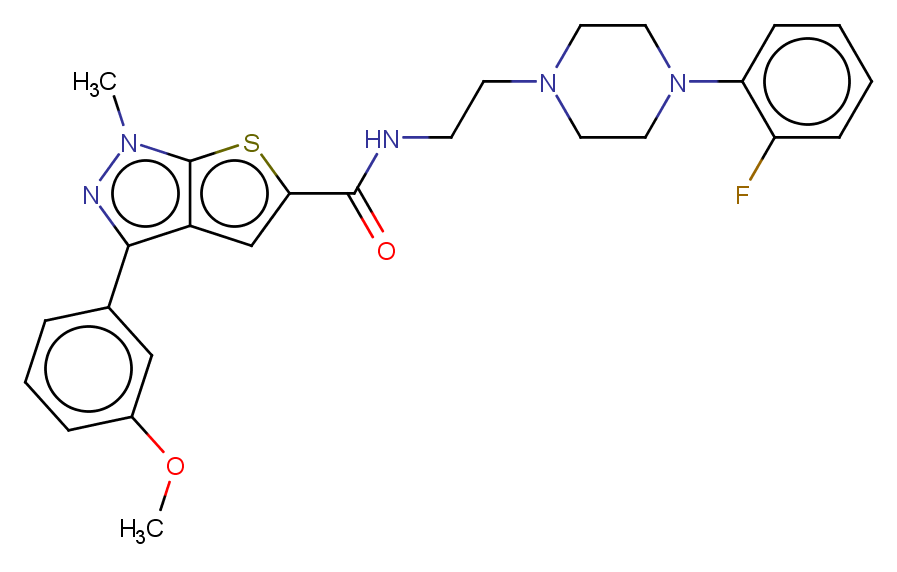

Supplement: RA-011-D1RA00914A-s894 [file RA-011-D1RA00914A-s894.png]

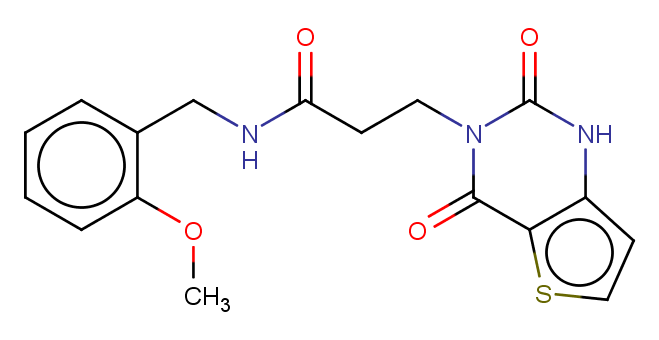

Supplement: RA-011-D1RA00914A-s895 [file RA-011-D1RA00914A-s895.png]

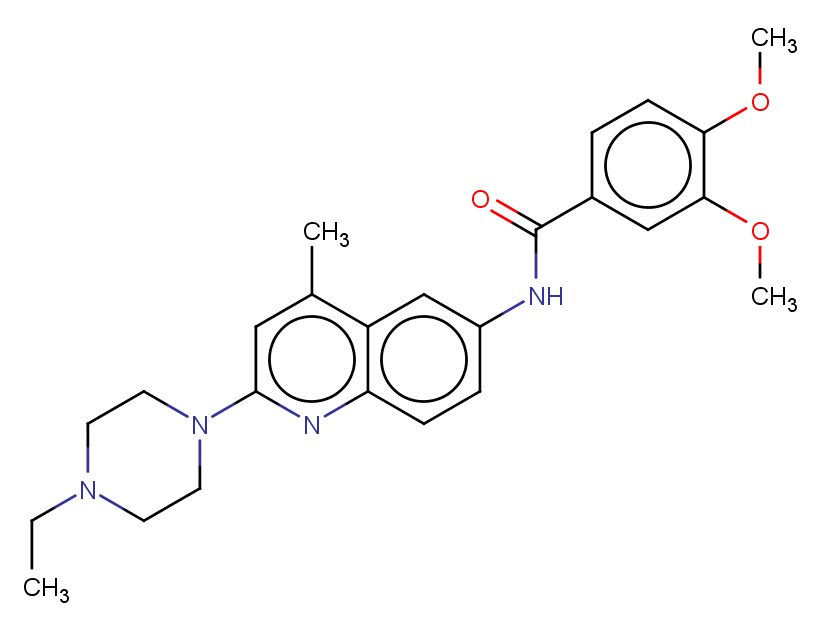

Supplement: RA-011-D1RA00914A-s896 [file RA-011-D1RA00914A-s896.png]

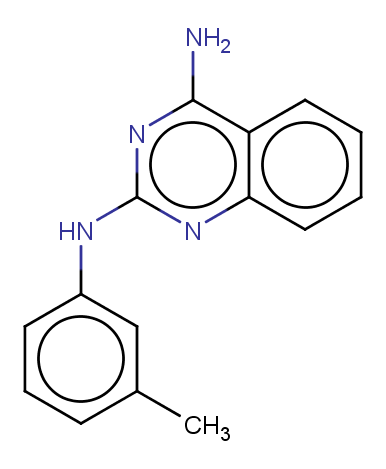

Supplement: RA-011-D1RA00914A-s897 [file RA-011-D1RA00914A-s897.png]

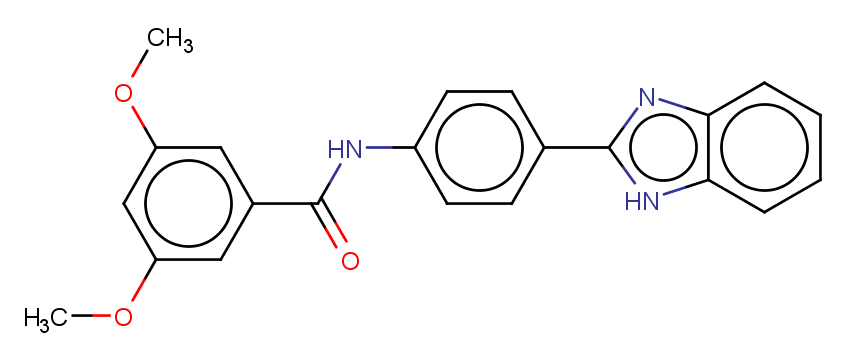

Supplement: RA-011-D1RA00914A-s898 [file RA-011-D1RA00914A-s898.png]

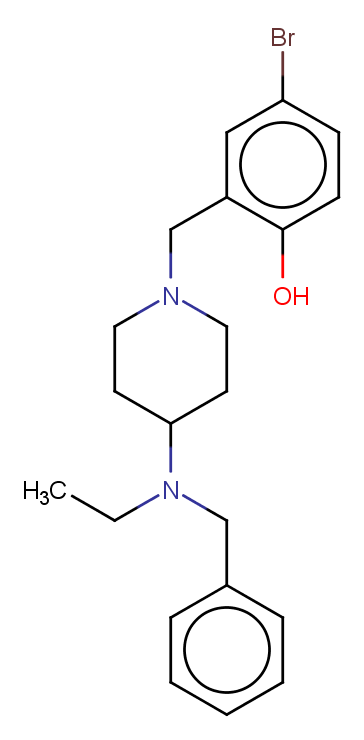

Supplement: RA-011-D1RA00914A-s899 [file RA-011-D1RA00914A-s899.png]

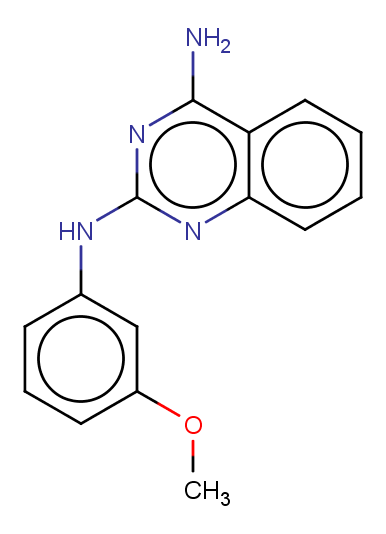

Supplement: RA-011-D1RA00914A-s900 [file RA-011-D1RA00914A-s900.png]

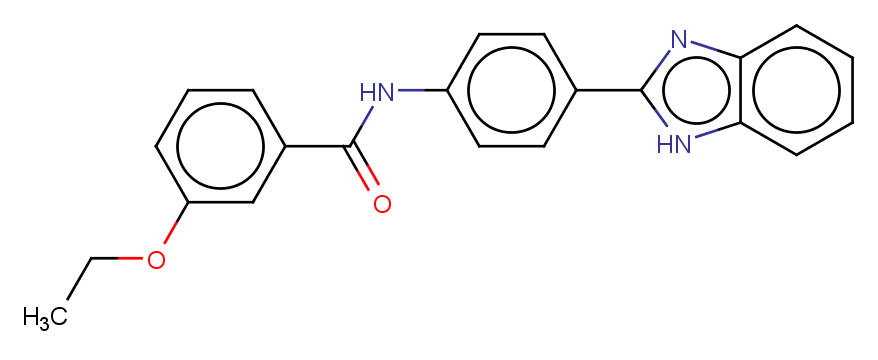

Supplement: RA-011-D1RA00914A-s901 [file RA-011-D1RA00914A-s901.png]

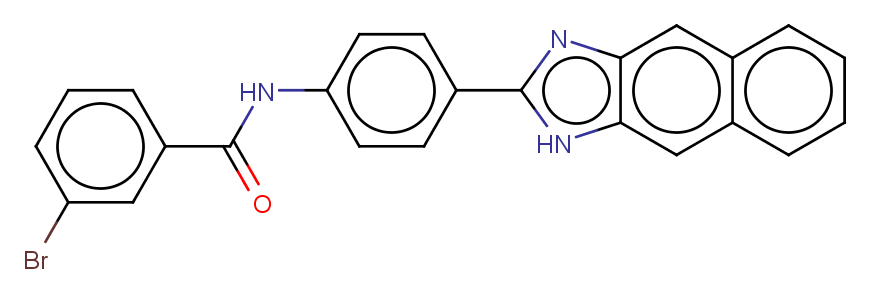

Supplement: RA-011-D1RA00914A-s902 [file RA-011-D1RA00914A-s902.png]

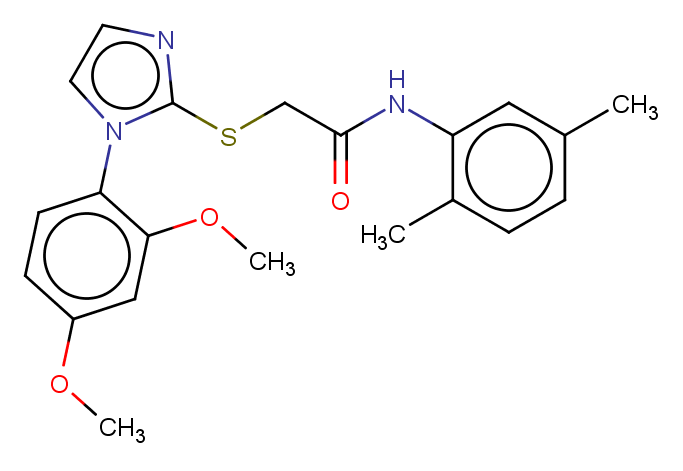

Supplement: RA-011-D1RA00914A-s903 [file RA-011-D1RA00914A-s903.png]

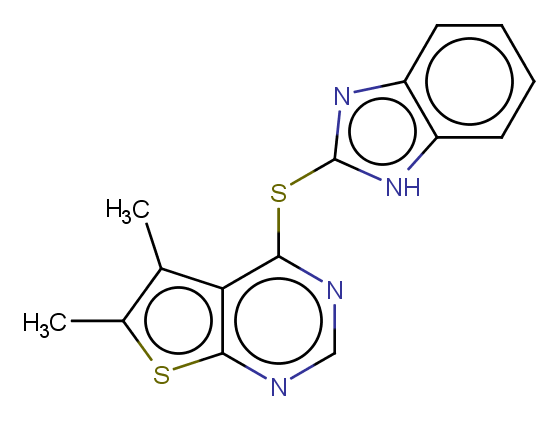

Supplement: RA-011-D1RA00914A-s904 [file RA-011-D1RA00914A-s904.png]

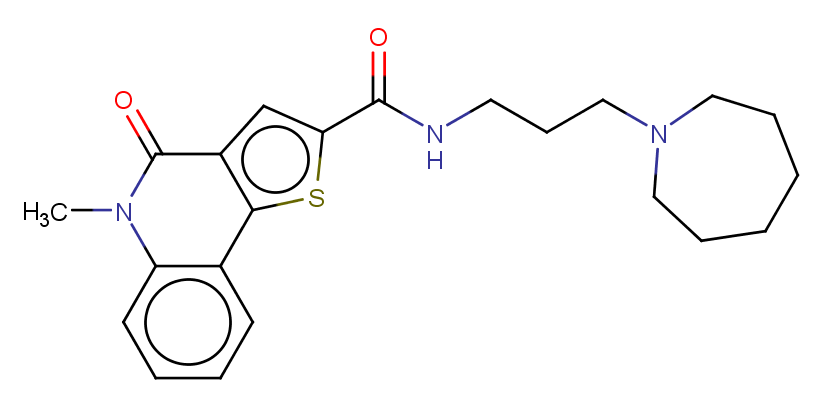

Supplement: RA-011-D1RA00914A-s905 [file RA-011-D1RA00914A-s905.png]

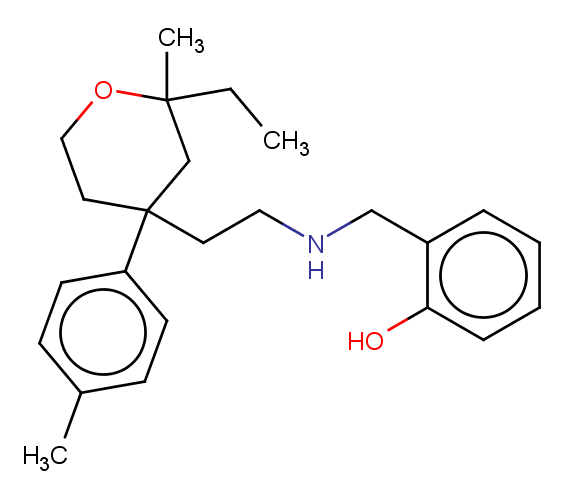

Supplement: RA-011-D1RA00914A-s906 [file RA-011-D1RA00914A-s906.png]

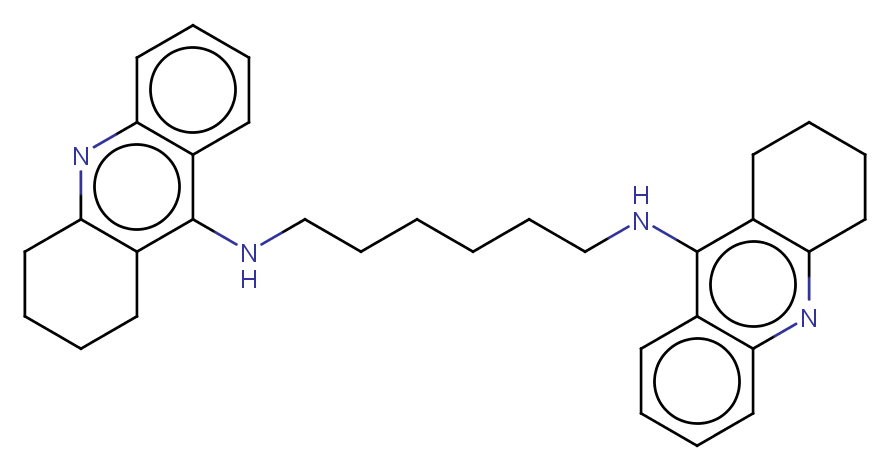

Supplement: RA-011-D1RA00914A-s907 [file RA-011-D1RA00914A-s907.png]

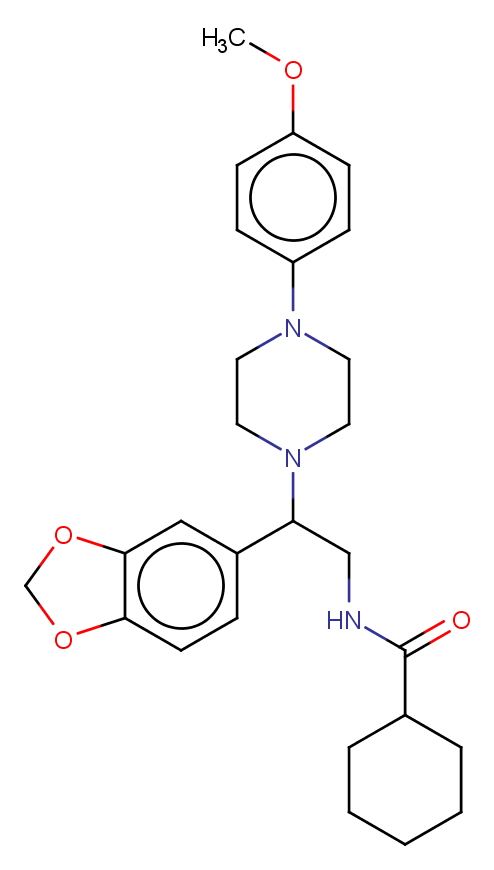

Supplement: RA-011-D1RA00914A-s908 [file RA-011-D1RA00914A-s908.png]

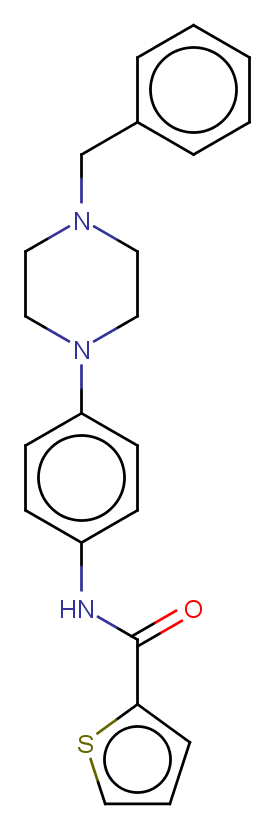

Supplement: RA-011-D1RA00914A-s909 [file RA-011-D1RA00914A-s909.png]

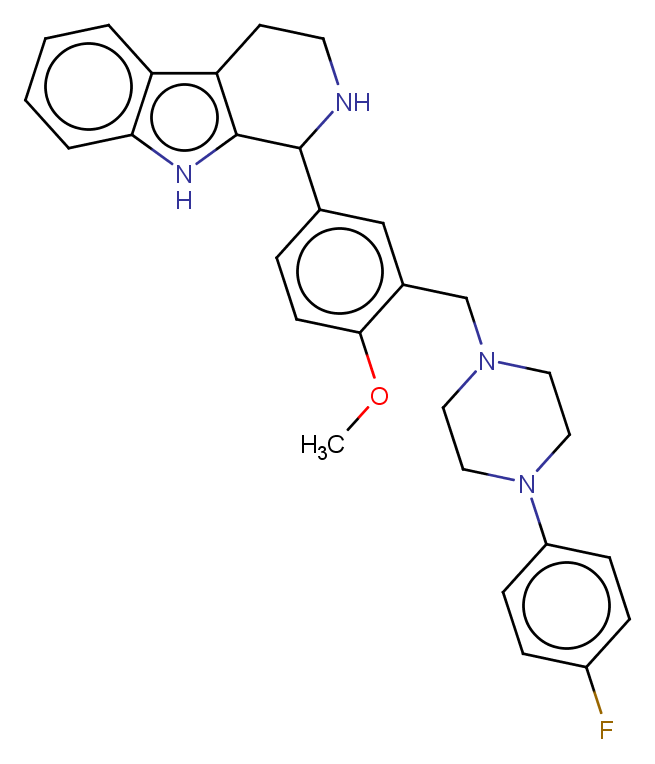

Supplement: RA-011-D1RA00914A-s910 [file RA-011-D1RA00914A-s910.png]

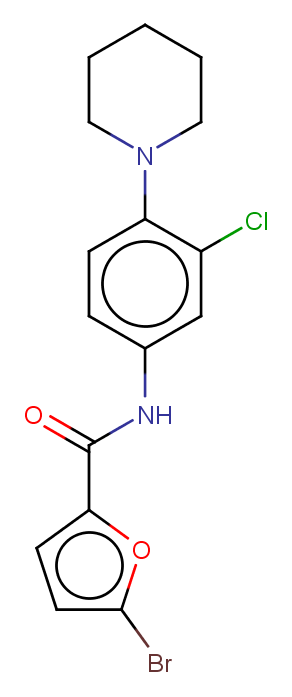

Supplement: RA-011-D1RA00914A-s911 [file RA-011-D1RA00914A-s911.png]

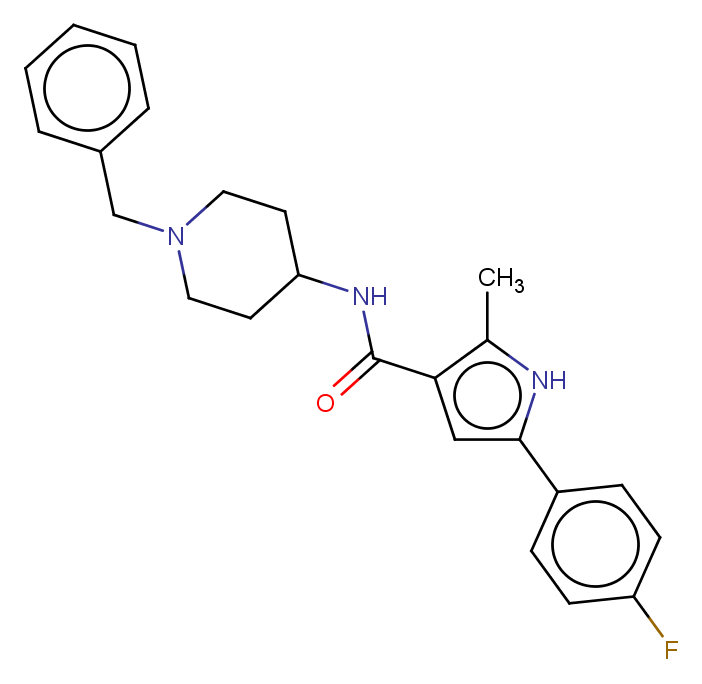

Supplement: RA-011-D1RA00914A-s912 [file RA-011-D1RA00914A-s912.png]

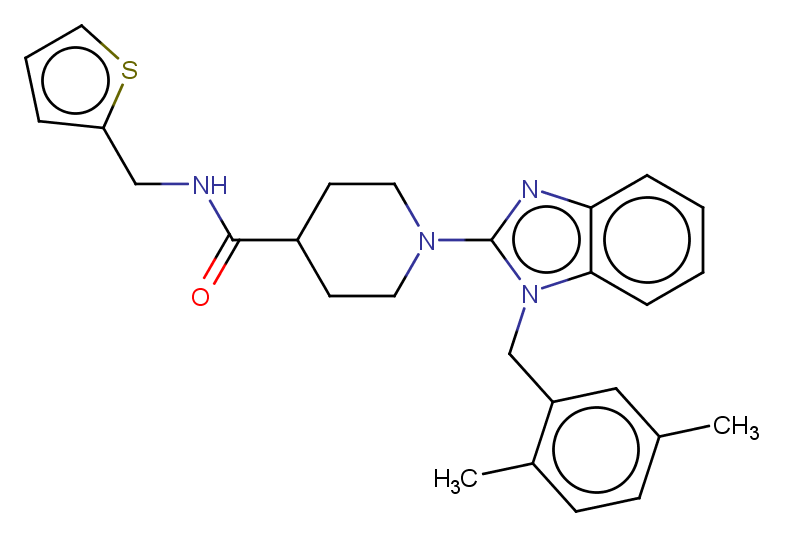

Supplement: RA-011-D1RA00914A-s913 [file RA-011-D1RA00914A-s913.png]

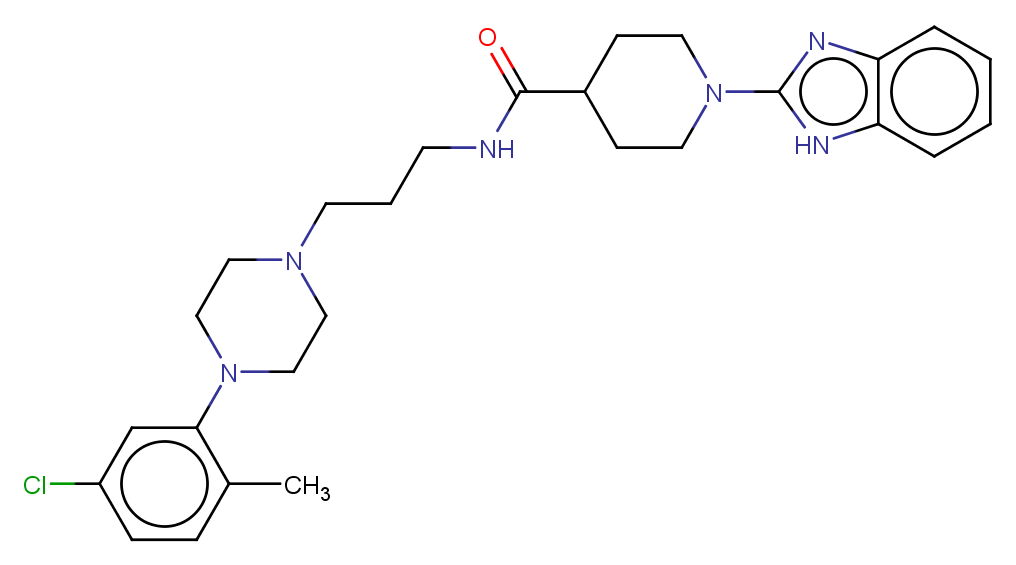

Supplement: RA-011-D1RA00914A-s914 [file RA-011-D1RA00914A-s914.png]

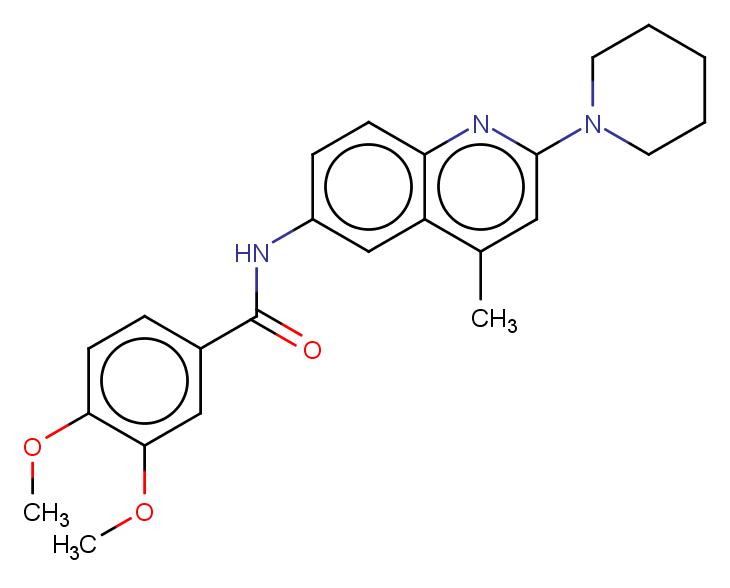

Supplement: RA-011-D1RA00914A-s915 [file RA-011-D1RA00914A-s915.png]

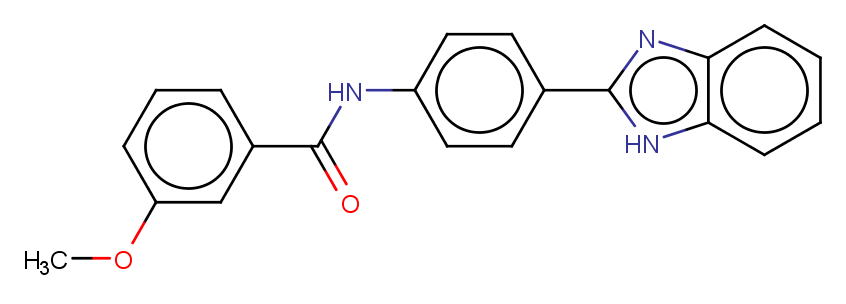

Supplement: RA-011-D1RA00914A-s916 [file RA-011-D1RA00914A-s916.png]

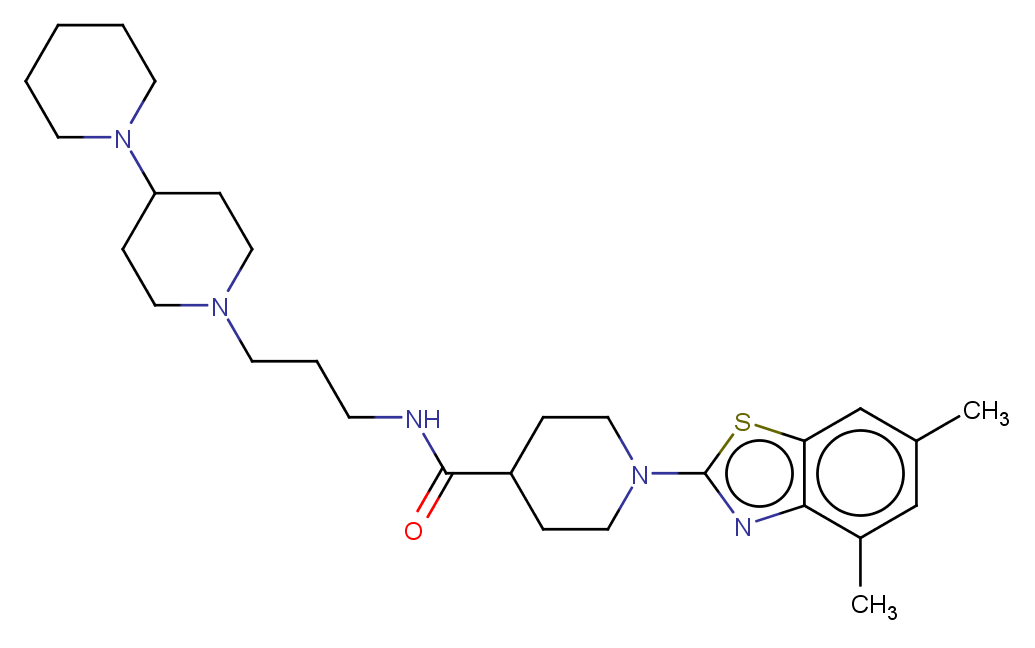

Supplement: RA-011-D1RA00914A-s917 [file RA-011-D1RA00914A-s917.png]

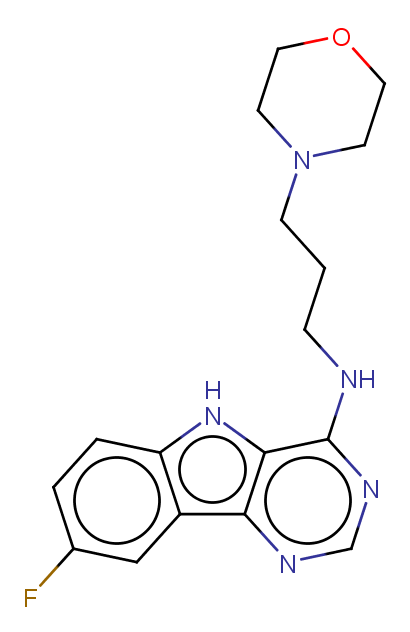

Supplement: RA-011-D1RA00914A-s918 [file RA-011-D1RA00914A-s918.png]

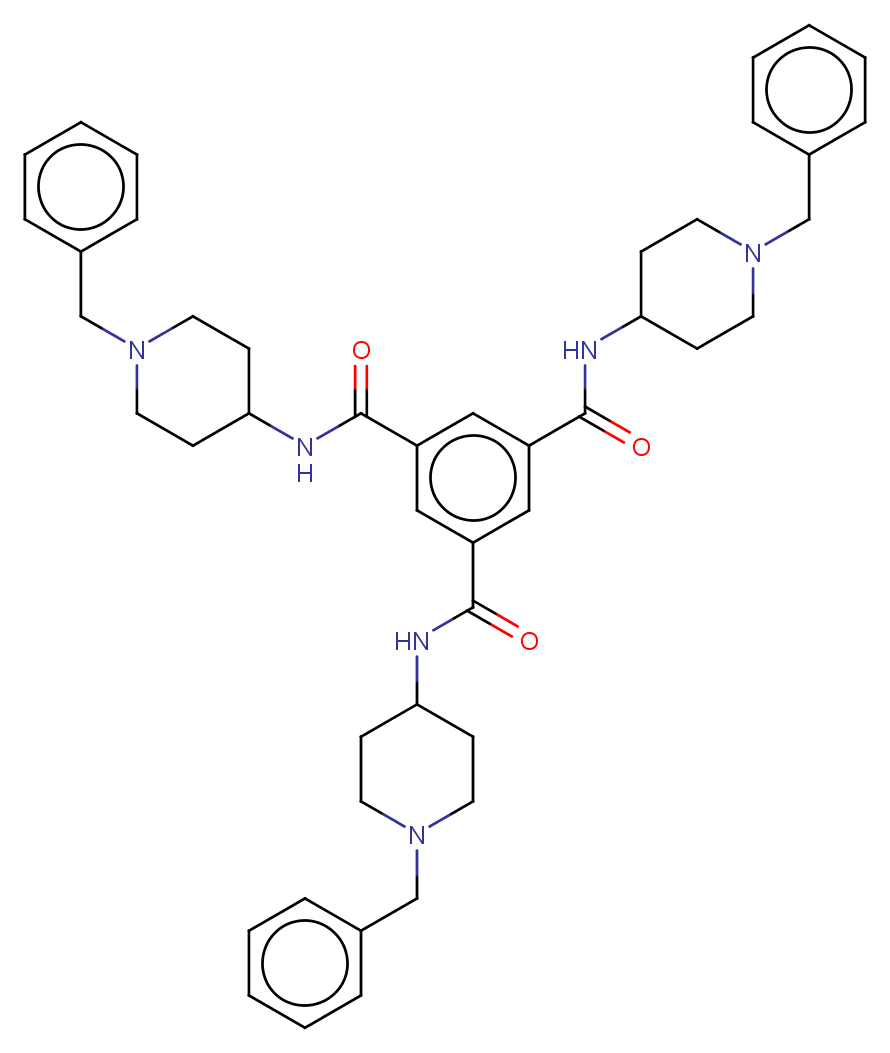

Supplement: RA-011-D1RA00914A-s919 [file RA-011-D1RA00914A-s919.png]

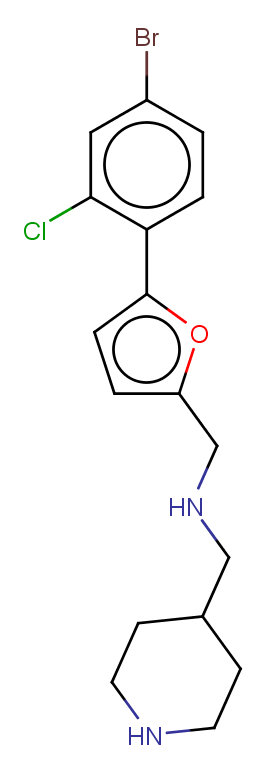

Supplement: RA-011-D1RA00914A-s920 [file RA-011-D1RA00914A-s920.png]

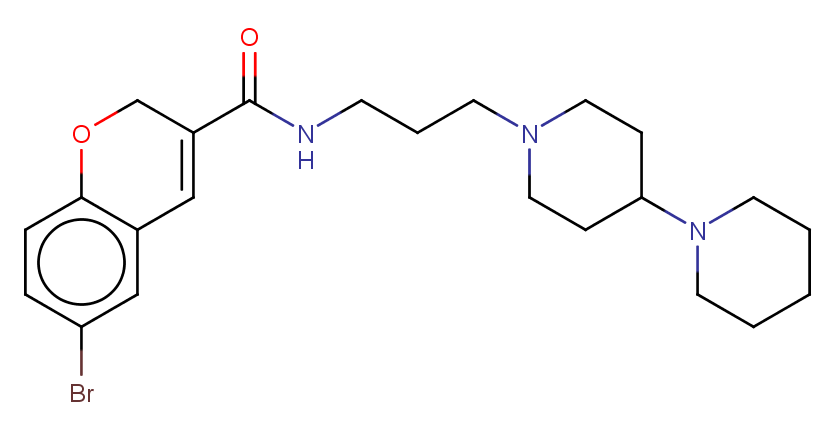

Supplement: RA-011-D1RA00914A-s921 [file RA-011-D1RA00914A-s921.png]

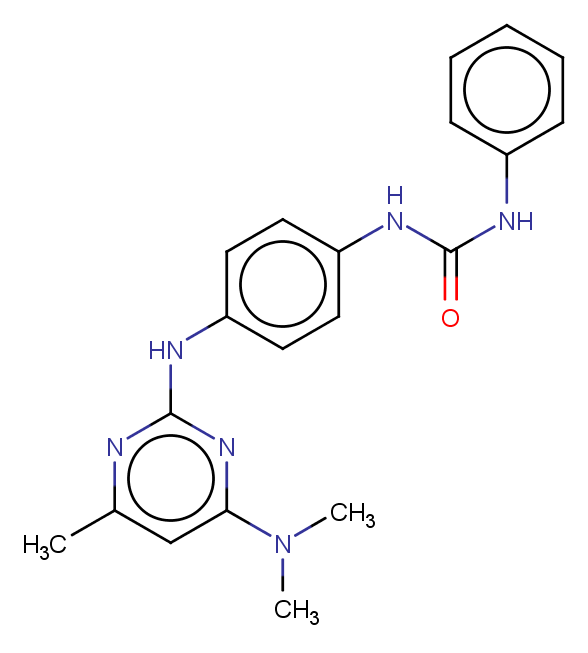

Supplement: RA-011-D1RA00914A-s922 [file RA-011-D1RA00914A-s922.png]

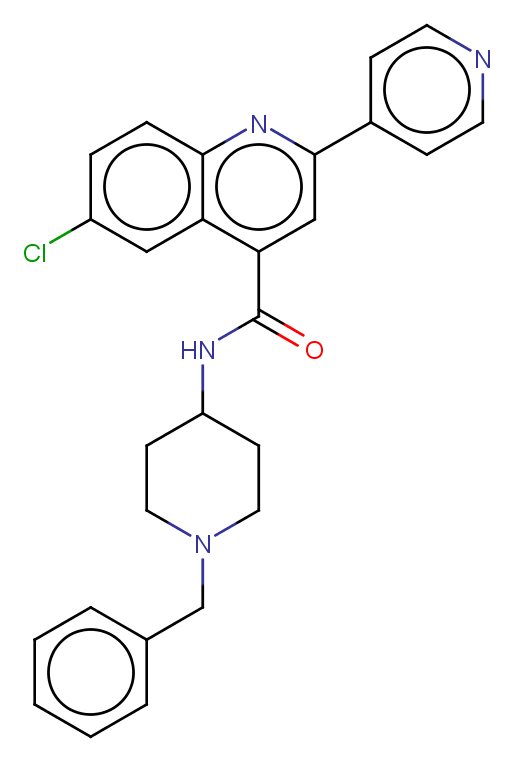

Supplement: RA-011-D1RA00914A-s923 [file RA-011-D1RA00914A-s923.png]

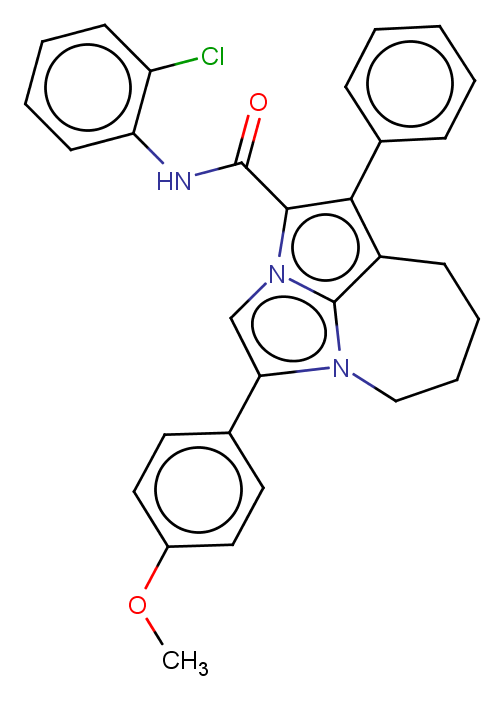

Supplement: RA-011-D1RA00914A-s924 [file RA-011-D1RA00914A-s924.png]

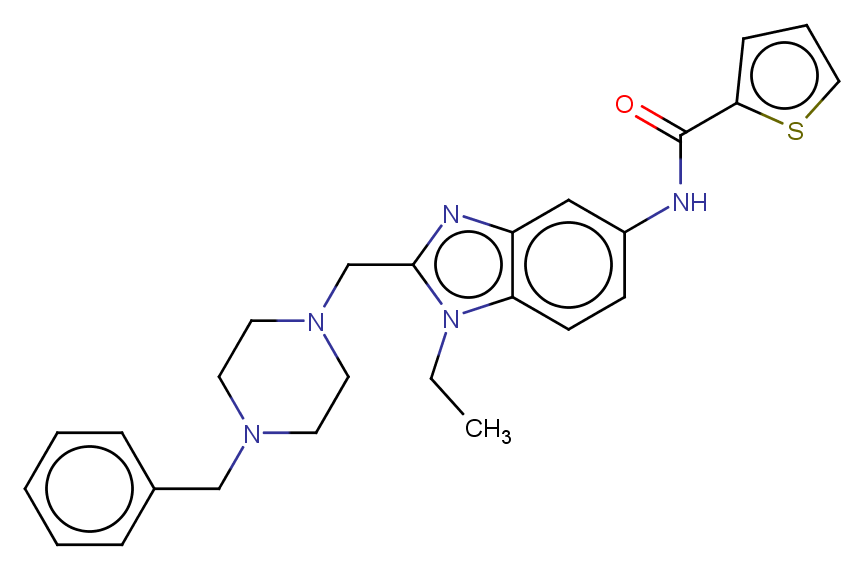

Supplement: RA-011-D1RA00914A-s925 [file RA-011-D1RA00914A-s925.png]

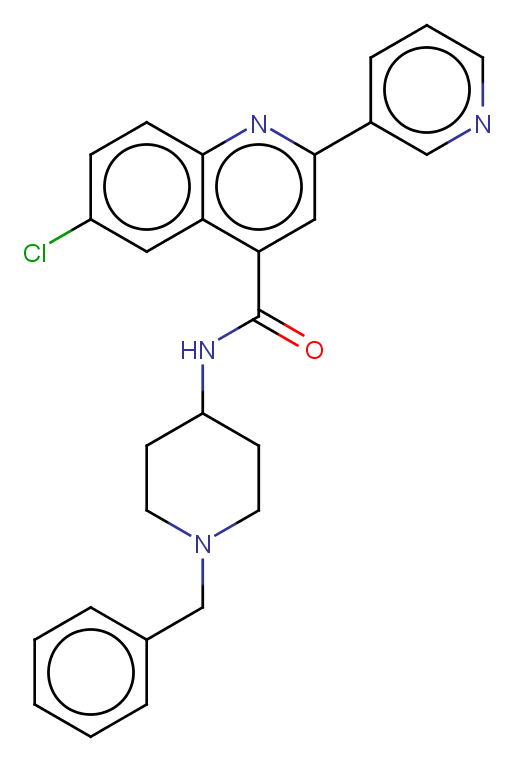

Supplement: RA-011-D1RA00914A-s926 [file RA-011-D1RA00914A-s926.png]

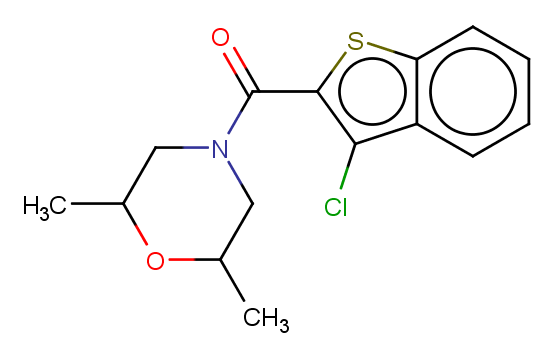

Supplement: RA-011-D1RA00914A-s927 [file RA-011-D1RA00914A-s927.png]

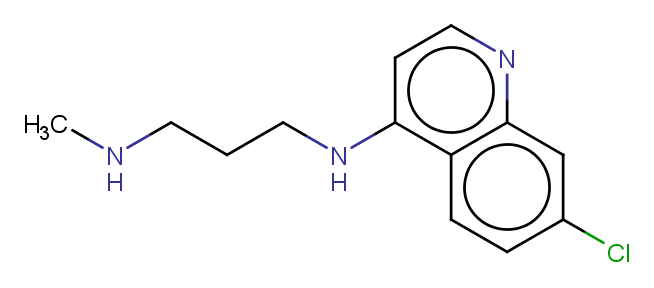

Supplement: RA-011-D1RA00914A-s928 [file RA-011-D1RA00914A-s928.png]

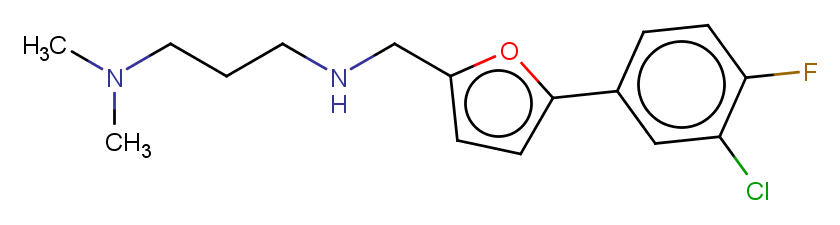

Supplement: RA-011-D1RA00914A-s929 [file RA-011-D1RA00914A-s929.png]

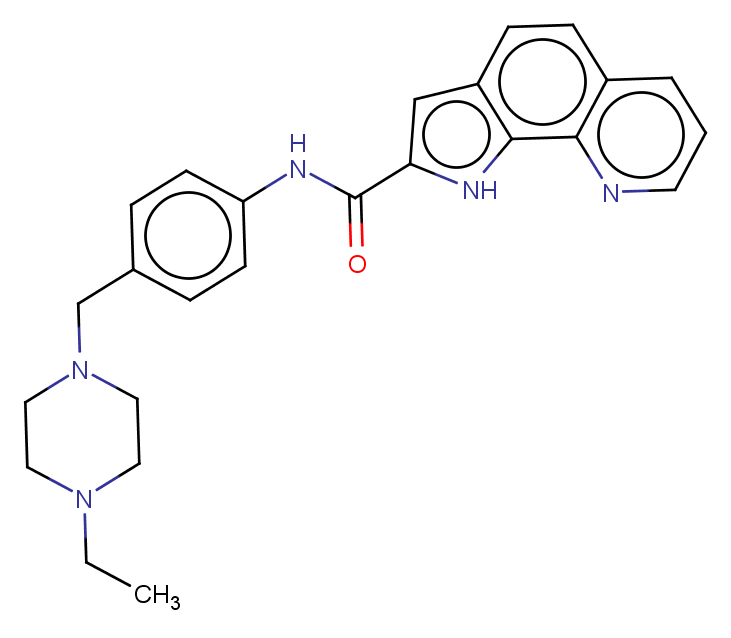

Supplement: RA-011-D1RA00914A-s930 [file RA-011-D1RA00914A-s930.png]

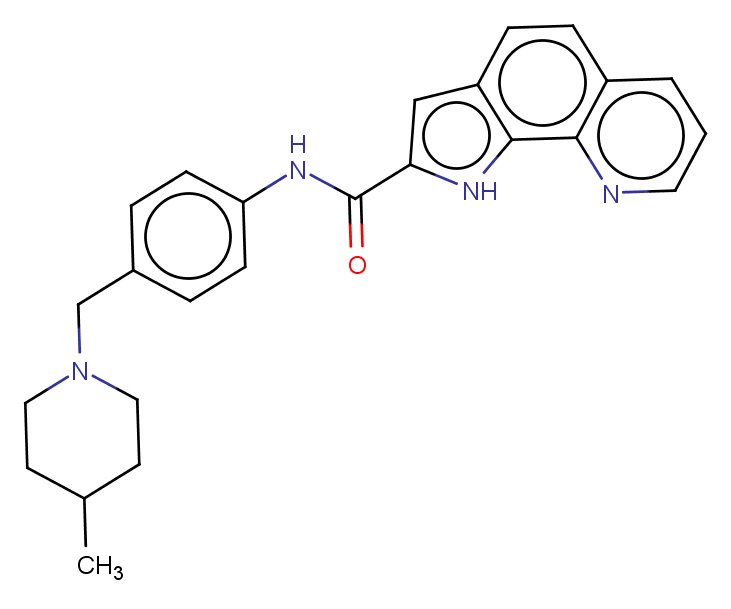

Supplement: RA-011-D1RA00914A-s931 [file RA-011-D1RA00914A-s931.png]

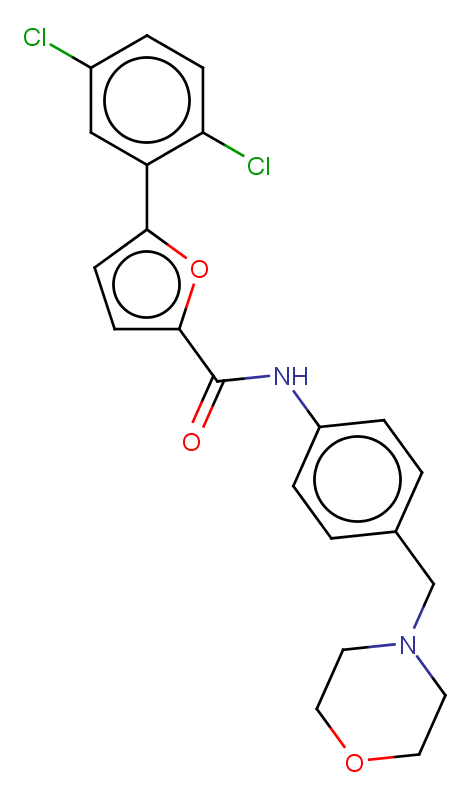

Supplement: RA-011-D1RA00914A-s932 [file RA-011-D1RA00914A-s932.png]

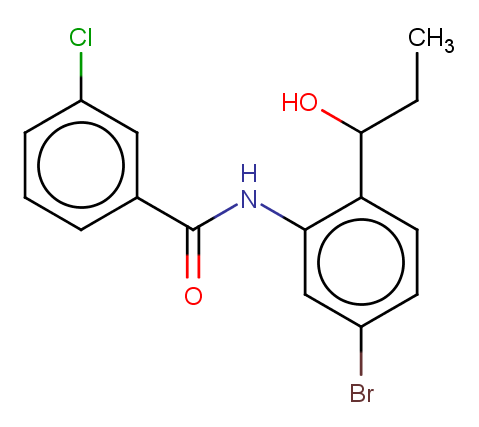

Supplement: RA-011-D1RA00914A-s933 [file RA-011-D1RA00914A-s933.png]

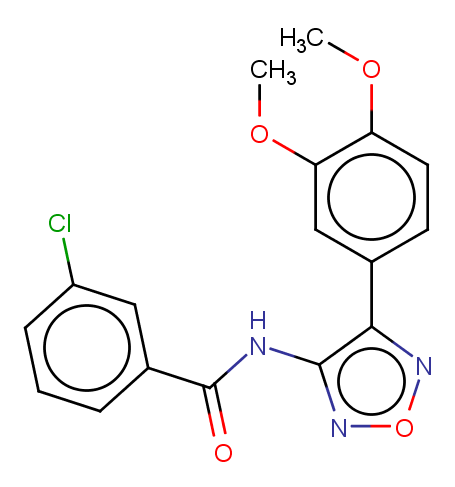

Supplement: RA-011-D1RA00914A-s934 [file RA-011-D1RA00914A-s934.png]

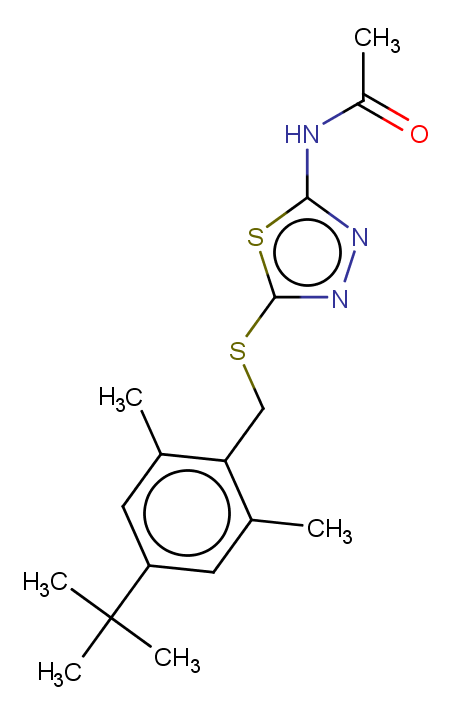

Supplement: RA-011-D1RA00914A-s935 [file RA-011-D1RA00914A-s935.png]

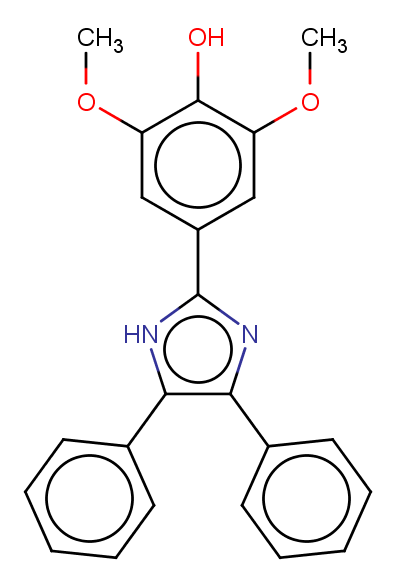

Supplement: RA-011-D1RA00914A-s936 [file RA-011-D1RA00914A-s936.png]

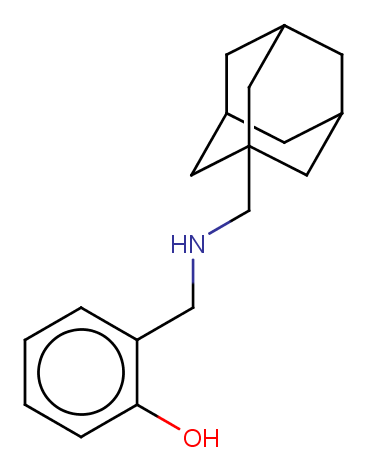

Supplement: RA-011-D1RA00914A-s937 [file RA-011-D1RA00914A-s937.png]

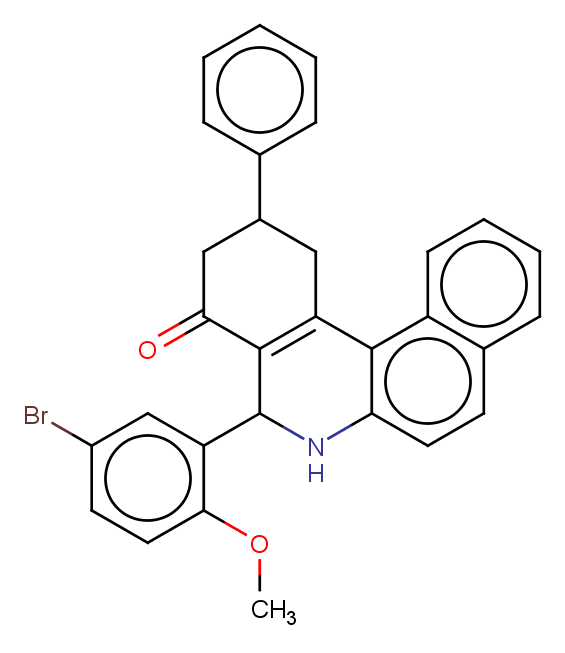

Supplement: RA-011-D1RA00914A-s938 [file RA-011-D1RA00914A-s938.png]

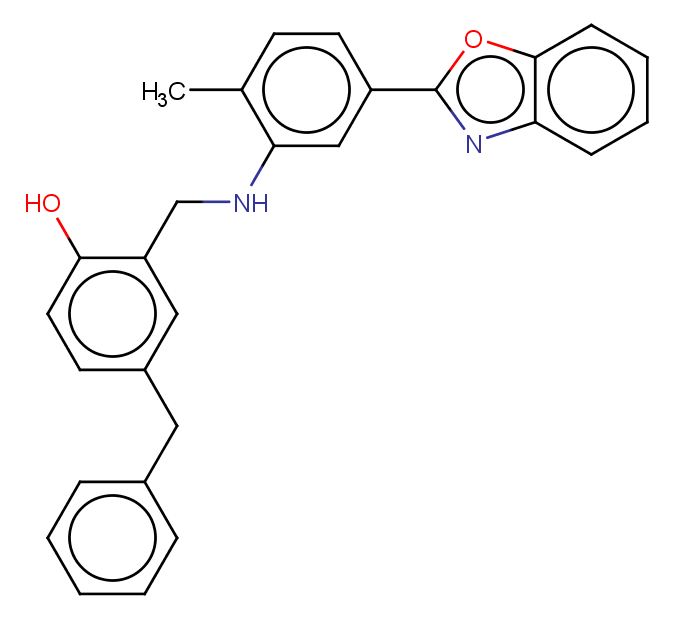

Supplement: RA-011-D1RA00914A-s939 [file RA-011-D1RA00914A-s939.png]

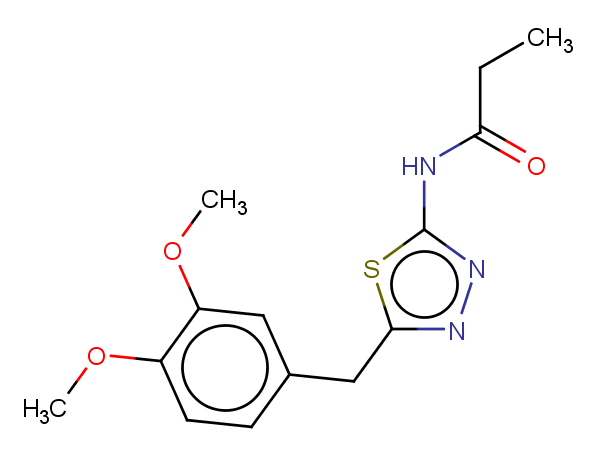

Supplement: RA-011-D1RA00914A-s940 [file RA-011-D1RA00914A-s940.png]

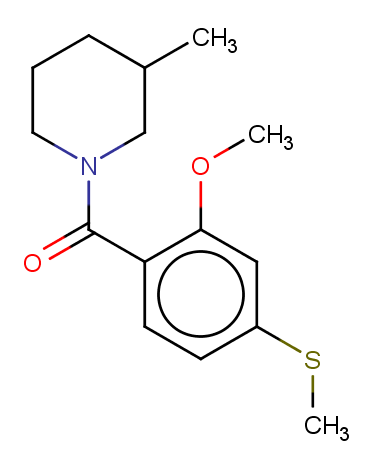

Supplement: RA-011-D1RA00914A-s941 [file RA-011-D1RA00914A-s941.png]

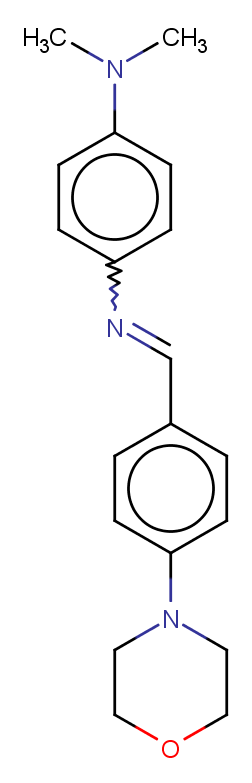

Supplement: RA-011-D1RA00914A-s942 [file RA-011-D1RA00914A-s942.png]

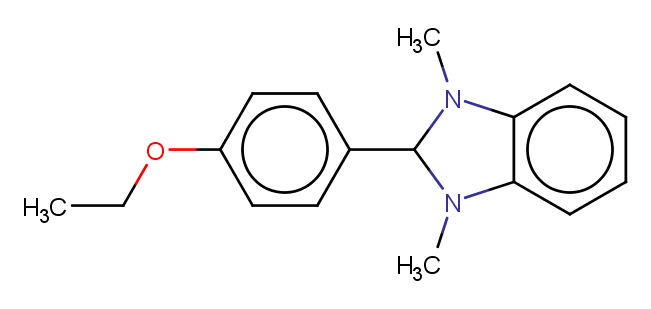

Supplement: RA-011-D1RA00914A-s943 [file RA-011-D1RA00914A-s943.png]

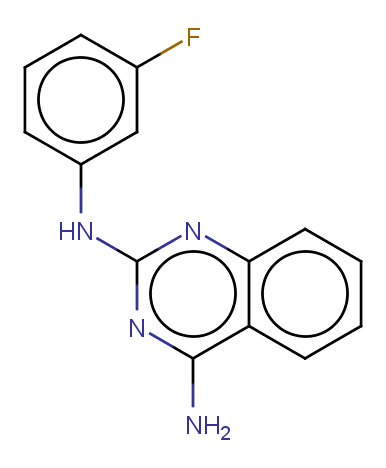

Supplement: RA-011-D1RA00914A-s944 [file RA-011-D1RA00914A-s944.png]

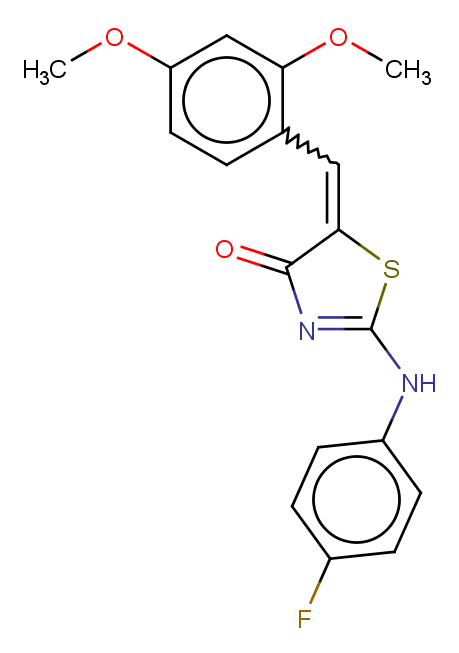

Supplement: RA-011-D1RA00914A-s945 [file RA-011-D1RA00914A-s945.png]

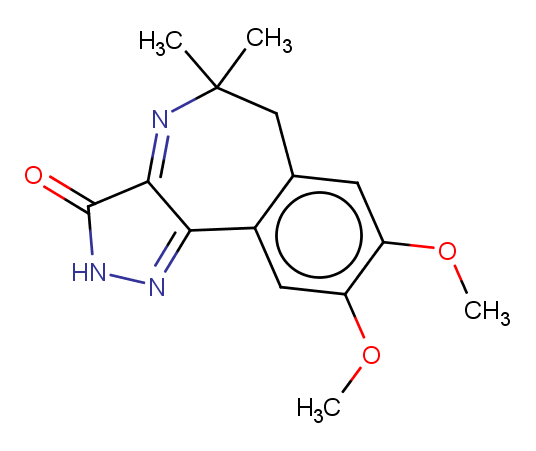

Supplement: RA-011-D1RA00914A-s946 [file RA-011-D1RA00914A-s946.png]

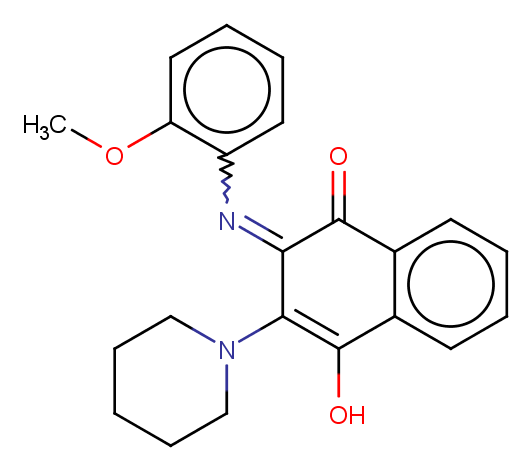

Supplement: RA-011-D1RA00914A-s947 [file RA-011-D1RA00914A-s947.png]

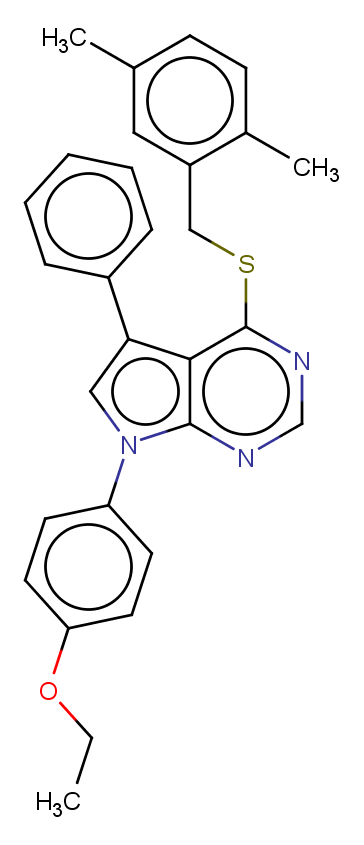

Supplement: RA-011-D1RA00914A-s948 [file RA-011-D1RA00914A-s948.png]

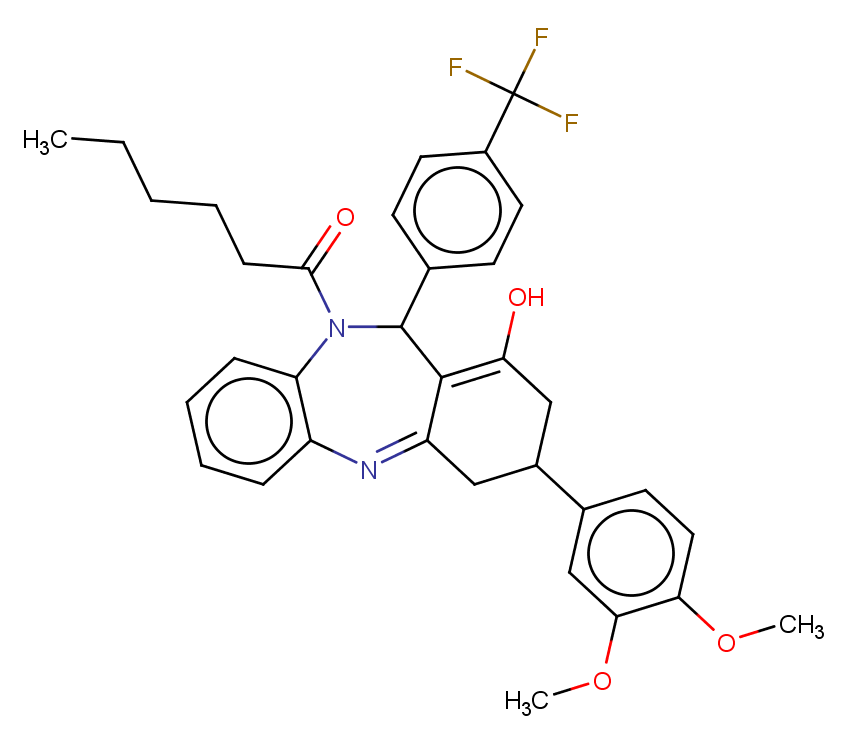

Supplement: RA-011-D1RA00914A-s949 [file RA-011-D1RA00914A-s949.png]

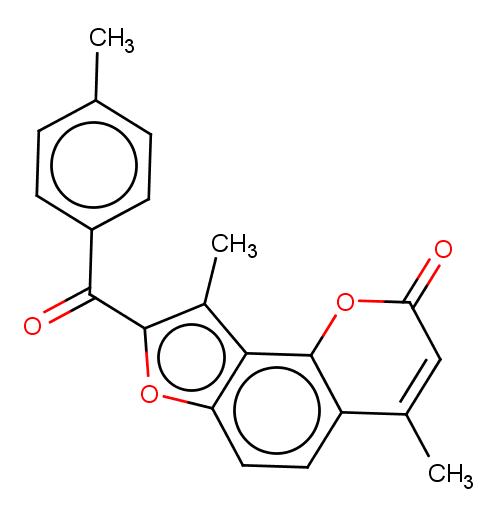

Supplement: RA-011-D1RA00914A-s950 [file RA-011-D1RA00914A-s950.png]

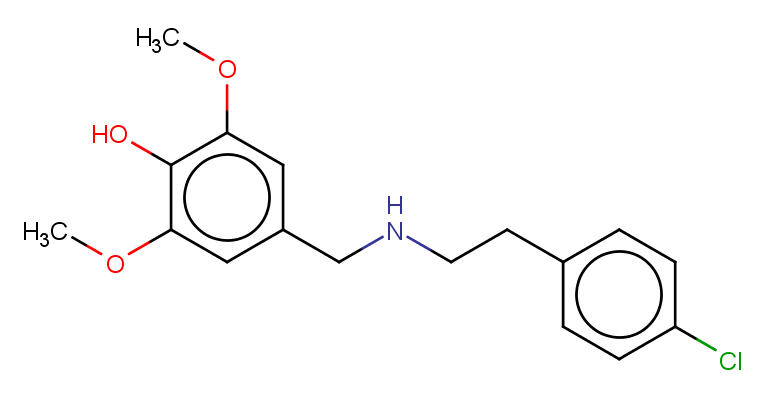

Supplement: RA-011-D1RA00914A-s951 [file RA-011-D1RA00914A-s951.png]

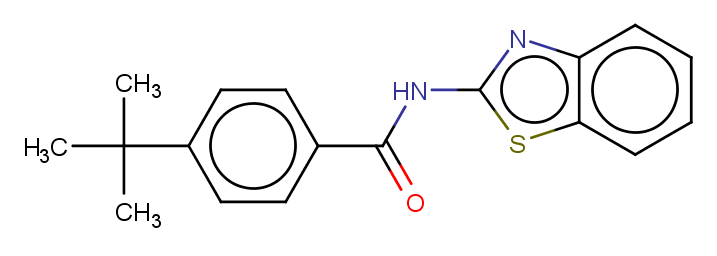

Supplement: RA-011-D1RA00914A-s952 [file RA-011-D1RA00914A-s952.png]

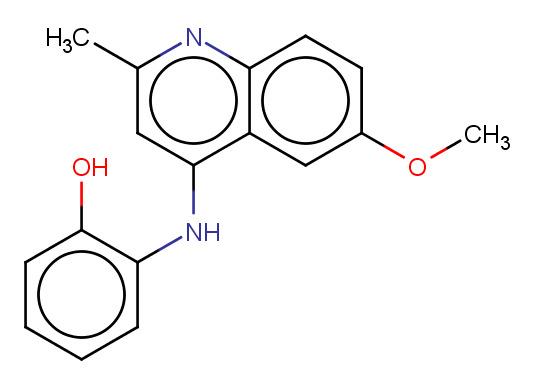

Supplement: RA-011-D1RA00914A-s953 [file RA-011-D1RA00914A-s953.png]

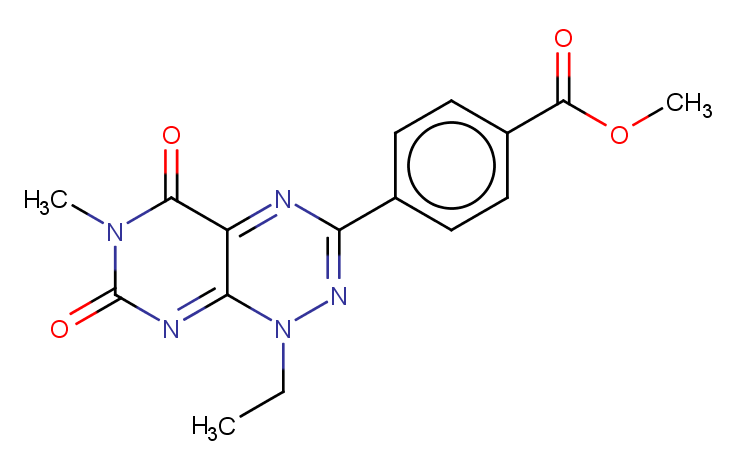

Supplement: RA-011-D1RA00914A-s954 [file RA-011-D1RA00914A-s954.png]

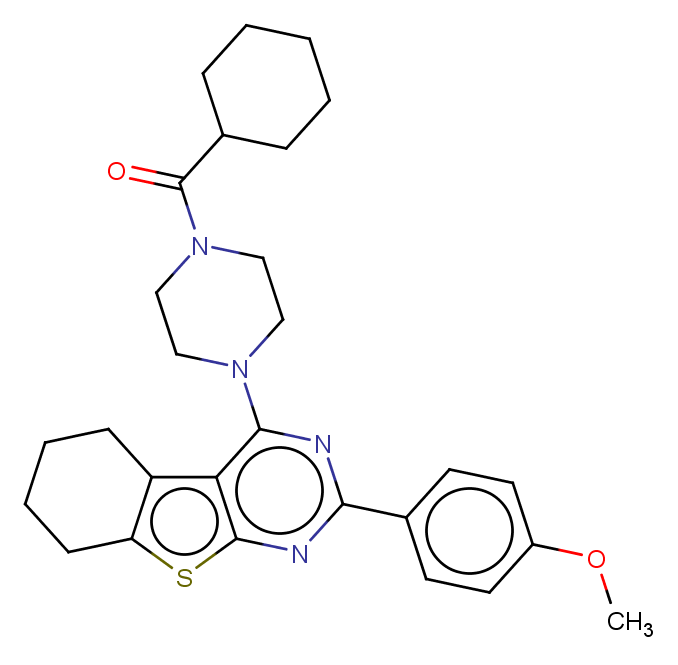

Supplement: RA-011-D1RA00914A-s955 [file RA-011-D1RA00914A-s955.png]

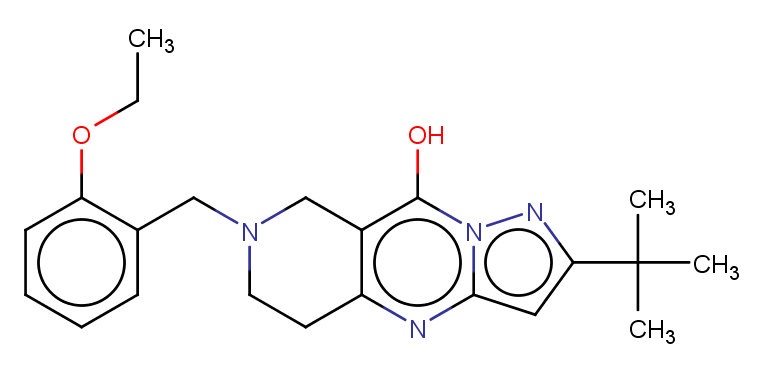

Supplement: RA-011-D1RA00914A-s956 [file RA-011-D1RA00914A-s956.png]

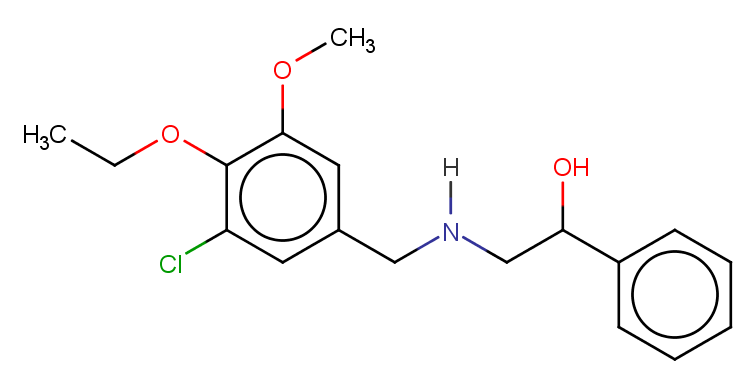

Supplement: RA-011-D1RA00914A-s957 [file RA-011-D1RA00914A-s957.png]

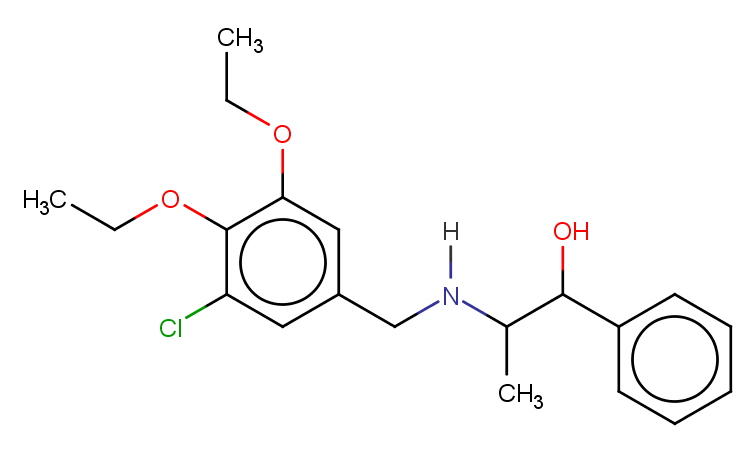

Supplement: RA-011-D1RA00914A-s958 [file RA-011-D1RA00914A-s958.png]

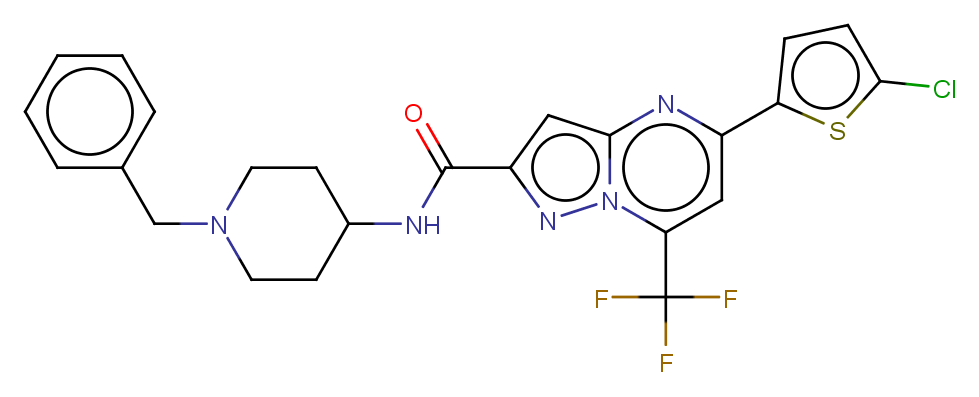

Supplement: RA-011-D1RA00914A-s959 [file RA-011-D1RA00914A-s959.png]

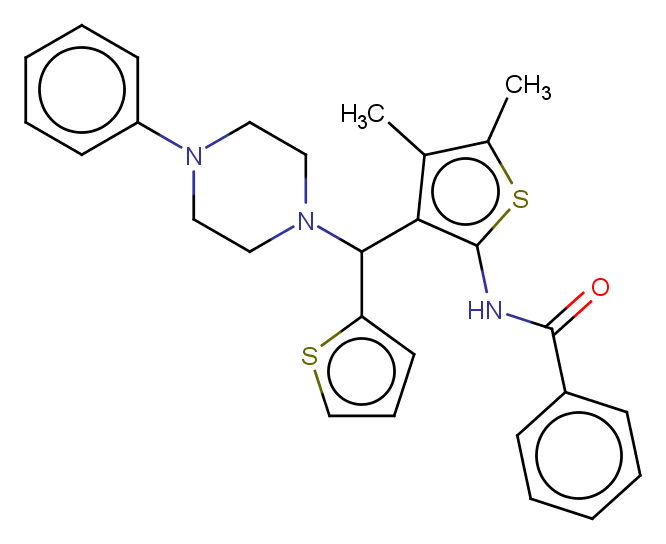

Supplement: RA-011-D1RA00914A-s960 [file RA-011-D1RA00914A-s960.png]

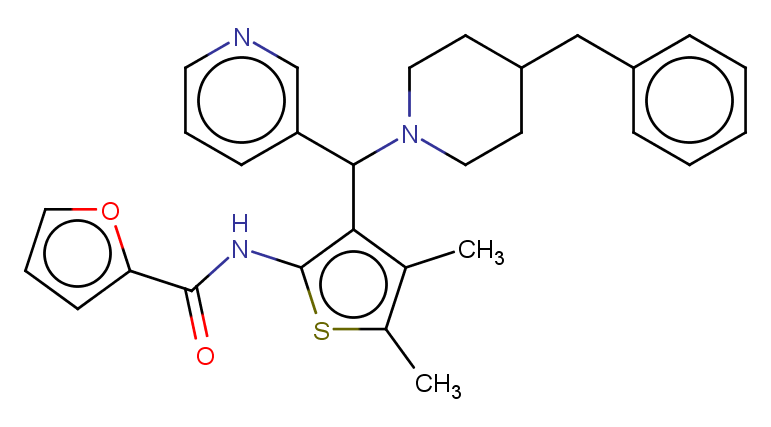

Supplement: RA-011-D1RA00914A-s961 [file RA-011-D1RA00914A-s961.png]

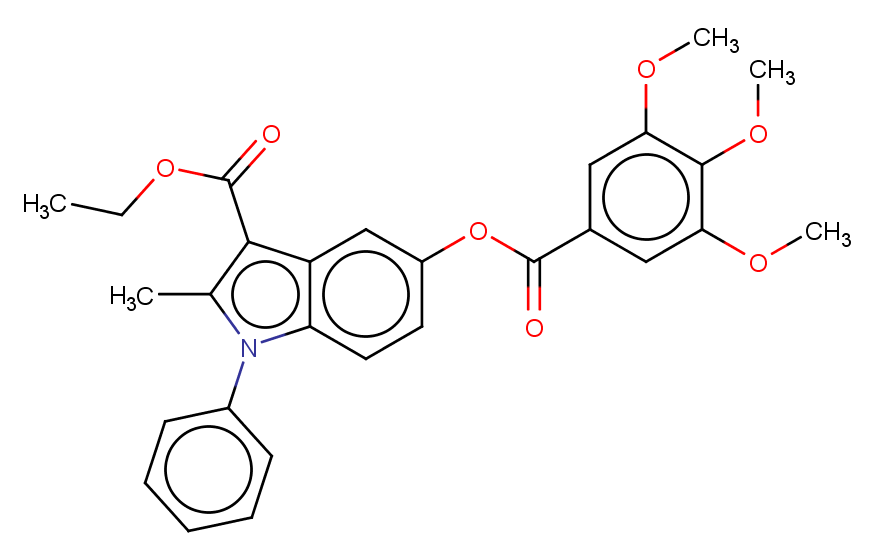

Supplement: RA-011-D1RA00914A-s962 [file RA-011-D1RA00914A-s962.png]

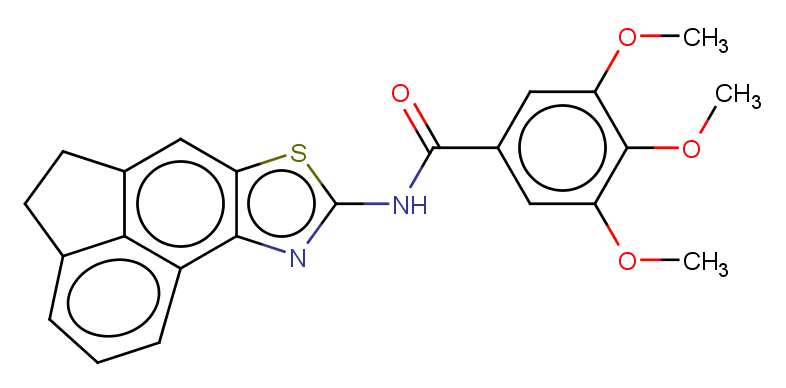

Supplement: RA-011-D1RA00914A-s963 [file RA-011-D1RA00914A-s963.png]

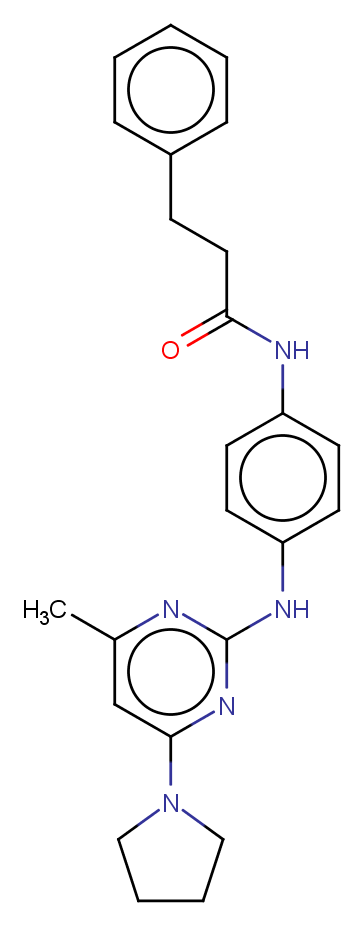

Supplement: RA-011-D1RA00914A-s964 [file RA-011-D1RA00914A-s964.png]

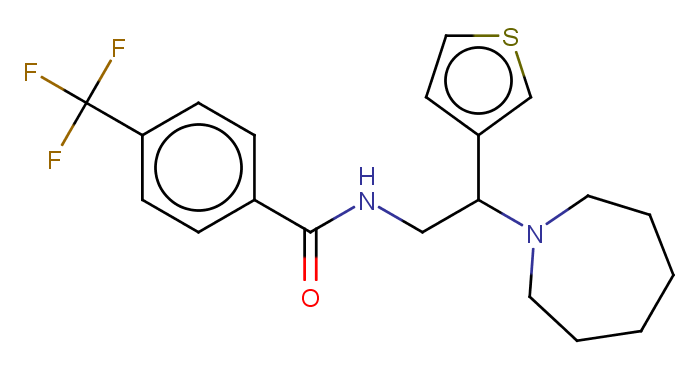

Supplement: RA-011-D1RA00914A-s965 [file RA-011-D1RA00914A-s965.png]

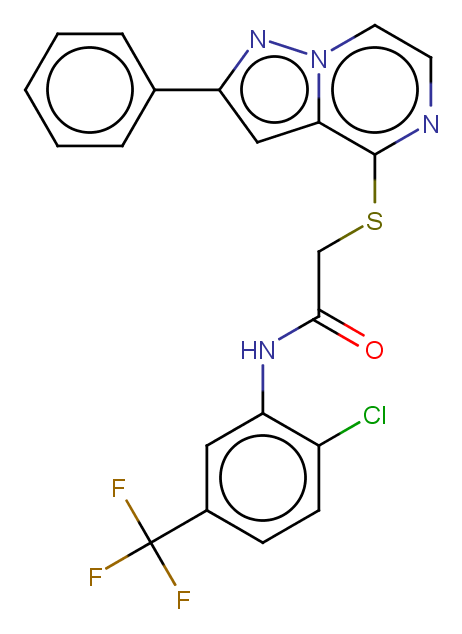

Supplement: RA-011-D1RA00914A-s966 [file RA-011-D1RA00914A-s966.png]

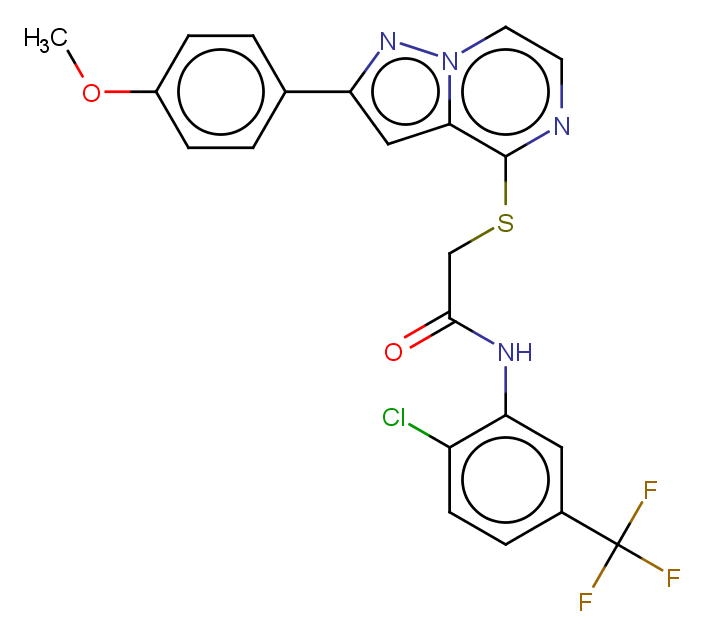

Supplement: RA-011-D1RA00914A-s967 [file RA-011-D1RA00914A-s967.png]

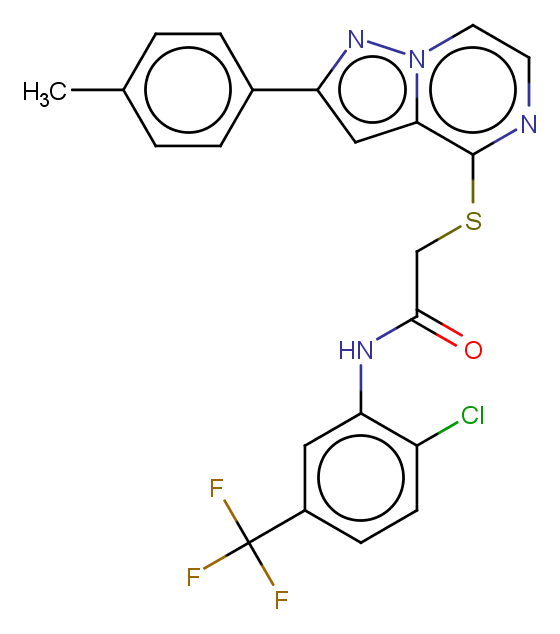

Supplement: RA-011-D1RA00914A-s968 [file RA-011-D1RA00914A-s968.png]

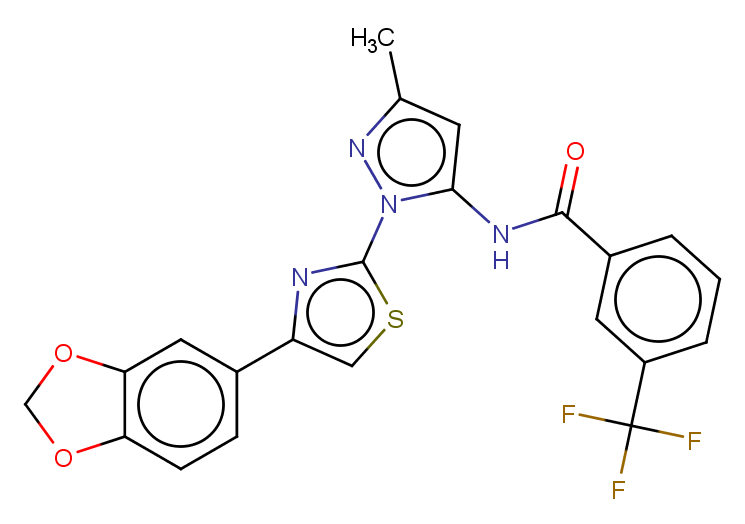

Supplement: RA-011-D1RA00914A-s969 [file RA-011-D1RA00914A-s969.png]

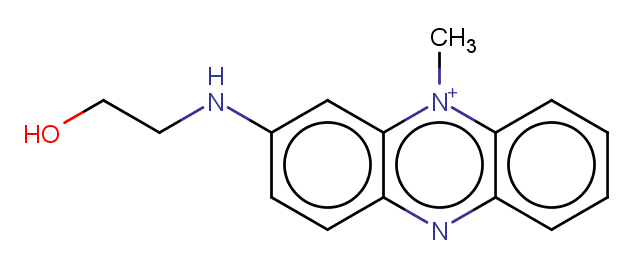

Supplement: RA-011-D1RA00914A-s970 [file RA-011-D1RA00914A-s970.png]

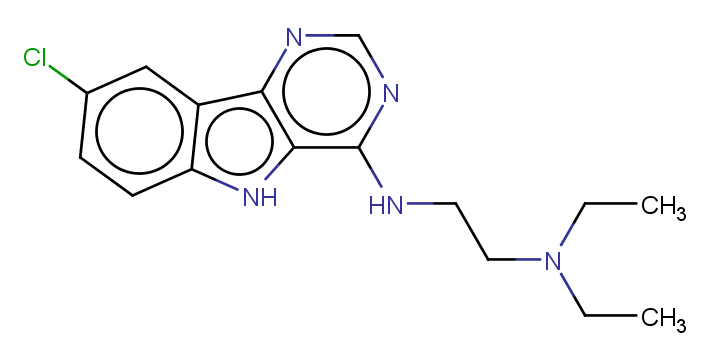

Supplement: RA-011-D1RA00914A-s971 [file RA-011-D1RA00914A-s971.png]
