# Supplementary figures and images for: Improved herbicide discovery using physico-chemical rules refined by antimalarial library screening (part 7 of 14)
Source: RSC Adv. 2021 Feb 23;11(15):8459–67. doi: 10.1039/d1ra00914a (PMC8695207; doi:10.1039/d1ra00914a)

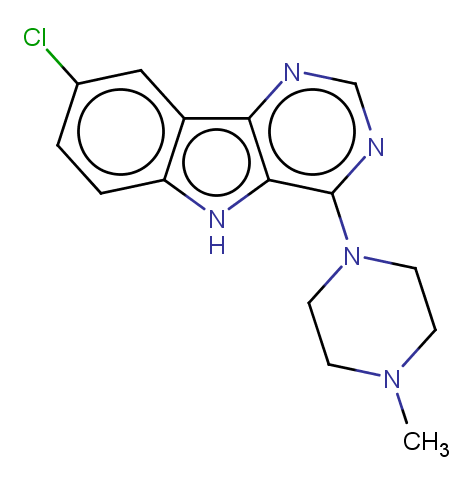

Supplement: RA-011-D1RA00914A-s972 [file RA-011-D1RA00914A-s972.png]

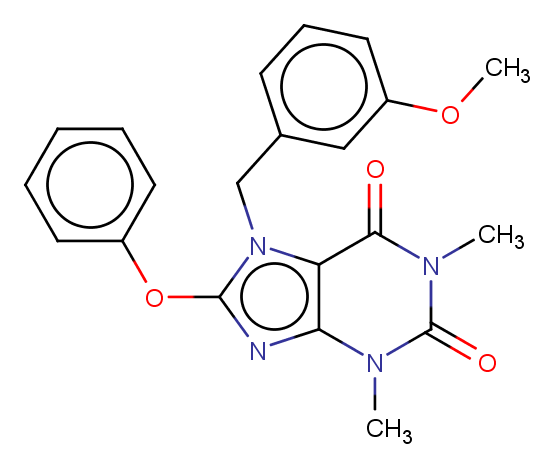

Supplement: RA-011-D1RA00914A-s973 [file RA-011-D1RA00914A-s973.png]

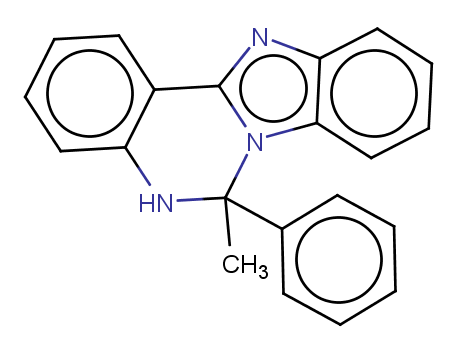

Supplement: RA-011-D1RA00914A-s974 [file RA-011-D1RA00914A-s974.png]

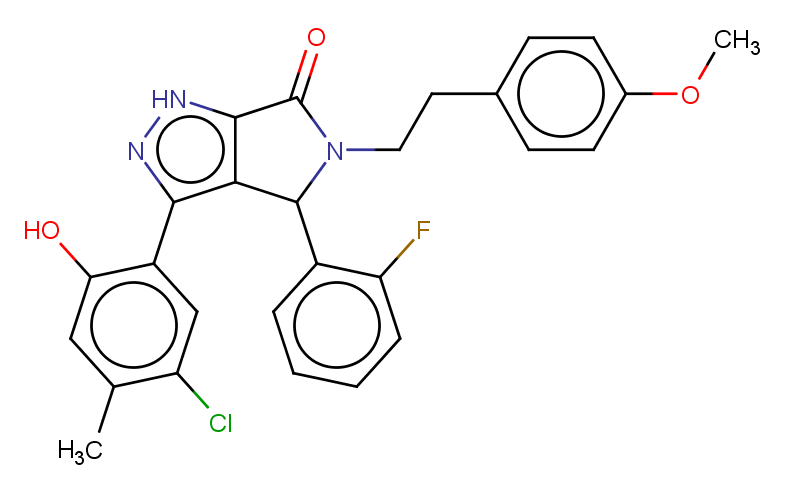

Supplement: RA-011-D1RA00914A-s975 [file RA-011-D1RA00914A-s975.png]

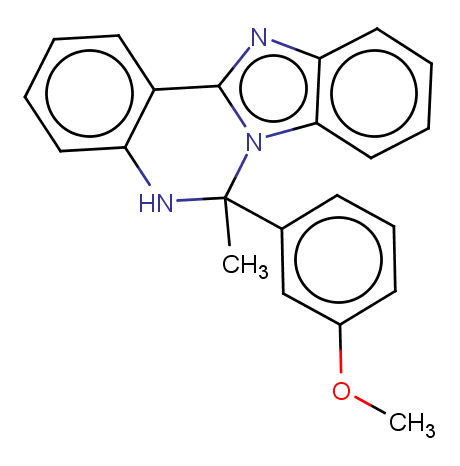

Supplement: RA-011-D1RA00914A-s976 [file RA-011-D1RA00914A-s976.png]

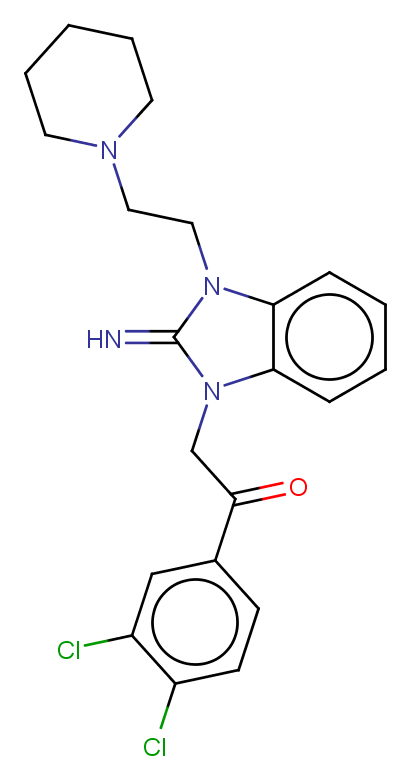

Supplement: RA-011-D1RA00914A-s977 [file RA-011-D1RA00914A-s977.png]

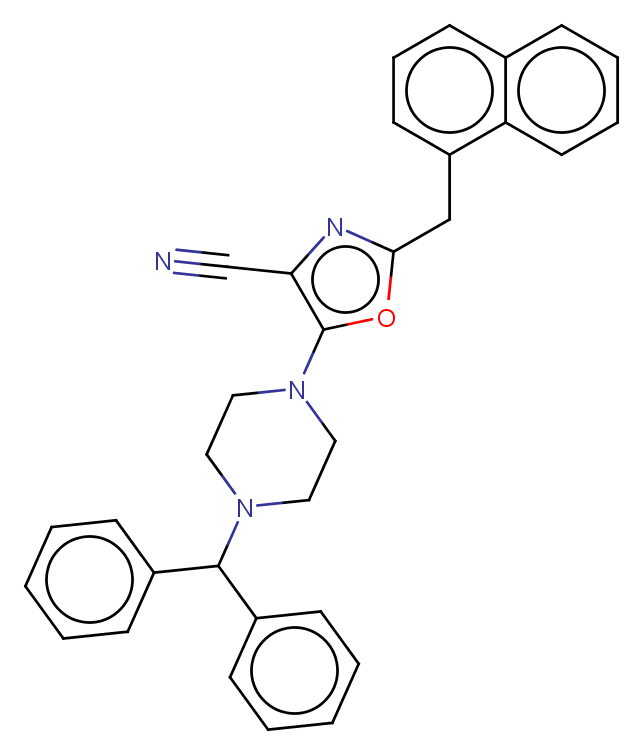

Supplement: RA-011-D1RA00914A-s978 [file RA-011-D1RA00914A-s978.png]

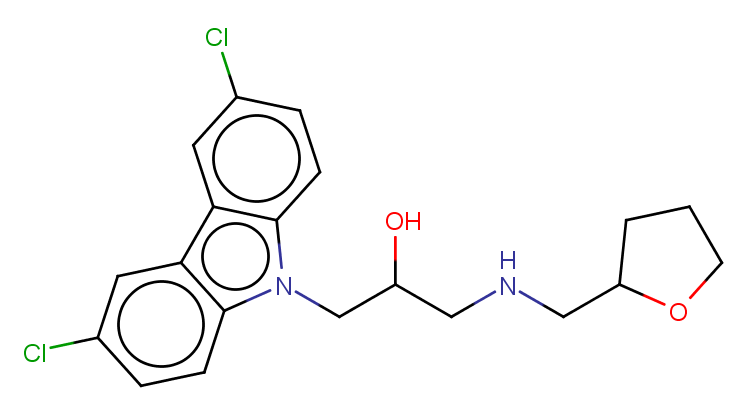

Supplement: RA-011-D1RA00914A-s979 [file RA-011-D1RA00914A-s979.png]

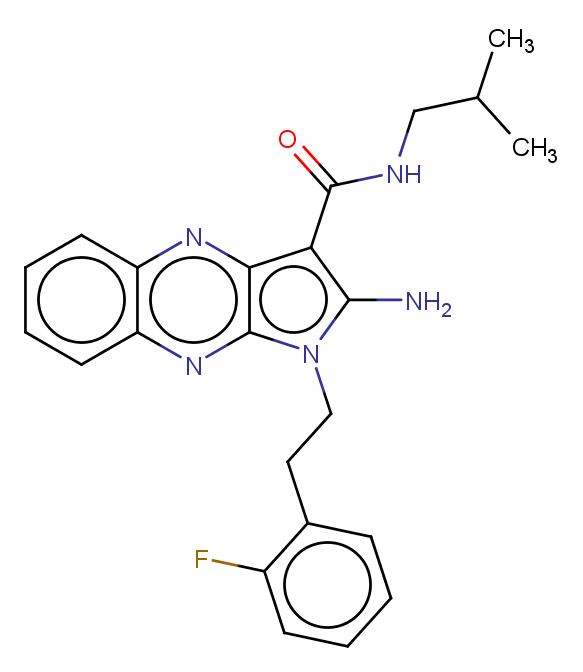

Supplement: RA-011-D1RA00914A-s980 [file RA-011-D1RA00914A-s980.png]

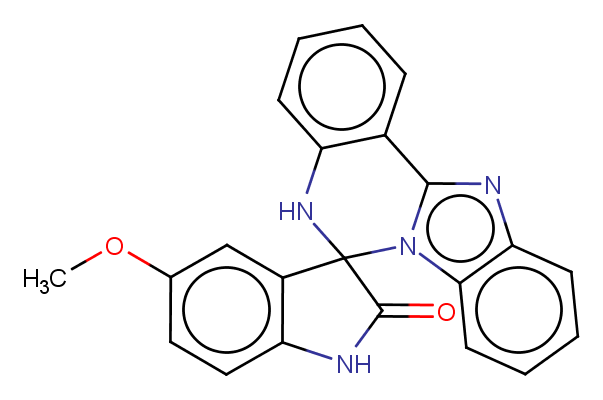

Supplement: RA-011-D1RA00914A-s981 [file RA-011-D1RA00914A-s981.png]

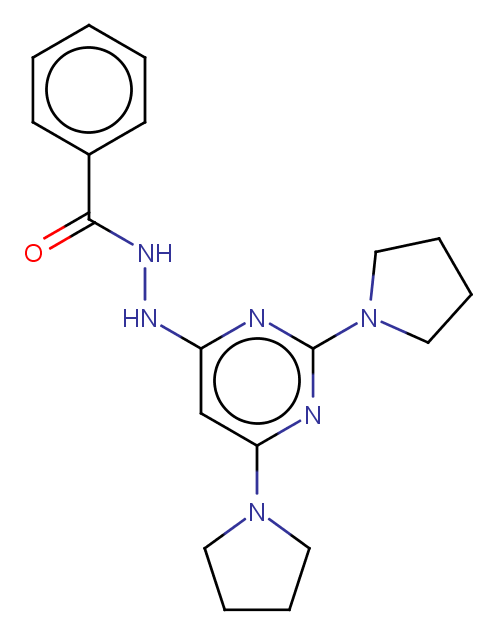

Supplement: RA-011-D1RA00914A-s982 [file RA-011-D1RA00914A-s982.png]

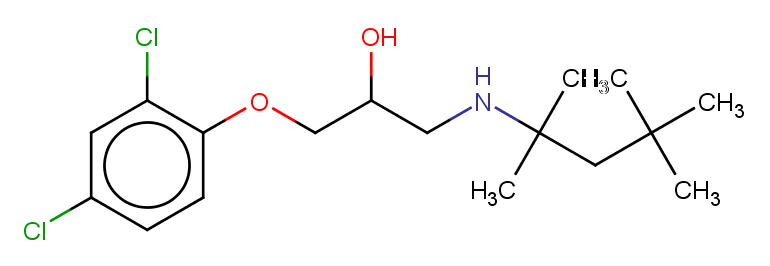

Supplement: RA-011-D1RA00914A-s983 [file RA-011-D1RA00914A-s983.png]

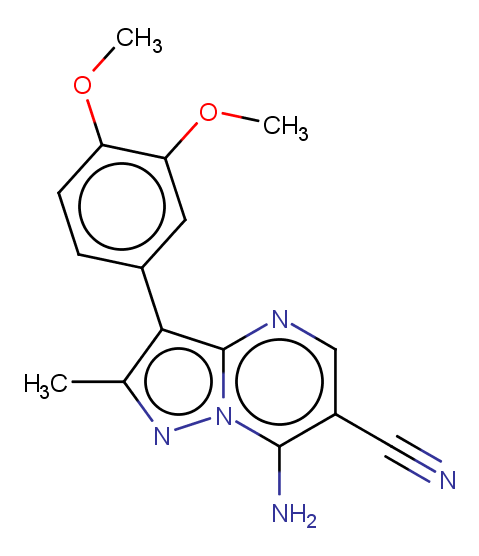

Supplement: RA-011-D1RA00914A-s984 [file RA-011-D1RA00914A-s984.png]

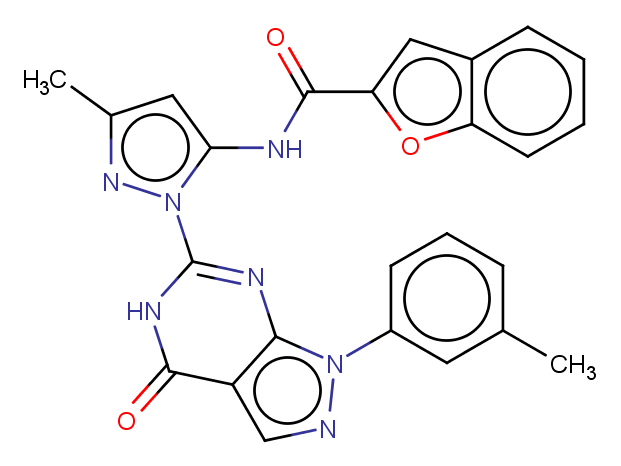

Supplement: RA-011-D1RA00914A-s985 [file RA-011-D1RA00914A-s985.png]

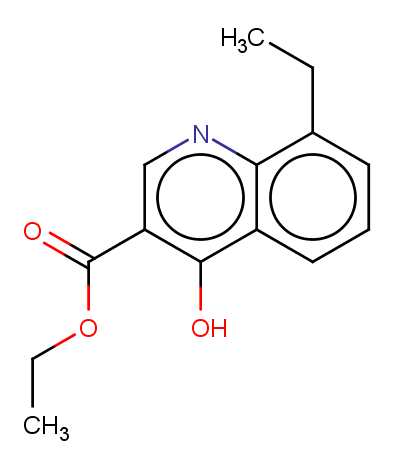

Supplement: RA-011-D1RA00914A-s986 [file RA-011-D1RA00914A-s986.png]

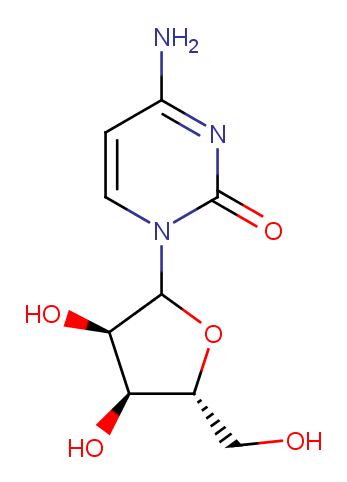

Supplement: RA-011-D1RA00914A-s987 [file RA-011-D1RA00914A-s987.png]

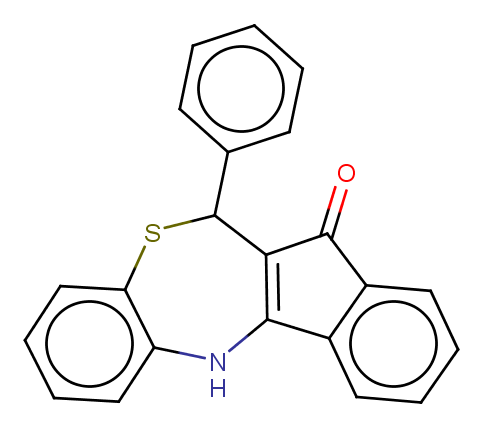

Supplement: RA-011-D1RA00914A-s988 [file RA-011-D1RA00914A-s988.png]

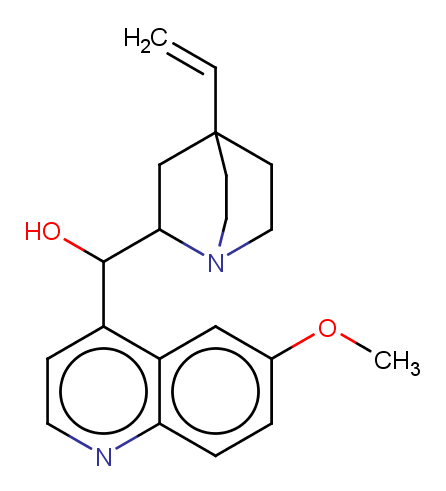

Supplement: RA-011-D1RA00914A-s989 [file RA-011-D1RA00914A-s989.png]

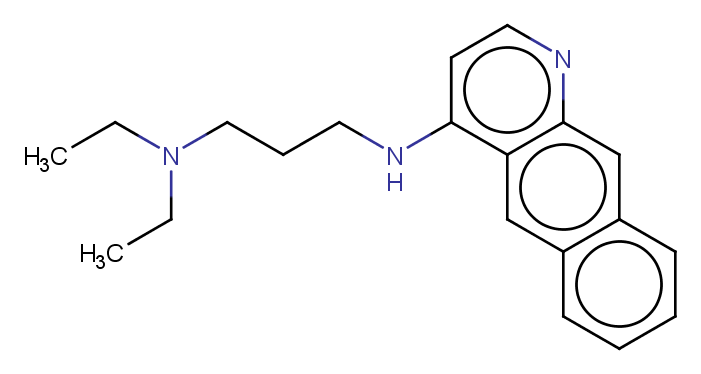

Supplement: RA-011-D1RA00914A-s990 [file RA-011-D1RA00914A-s990.png]

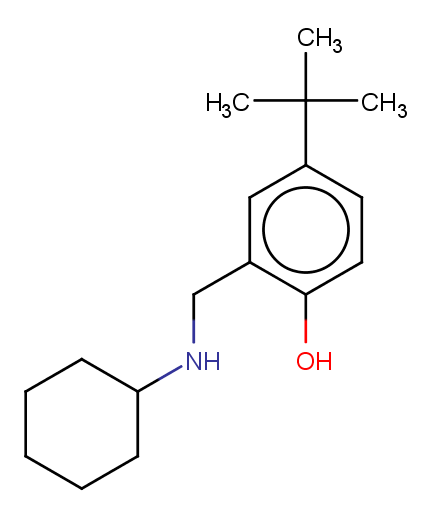

Supplement: RA-011-D1RA00914A-s991 [file RA-011-D1RA00914A-s991.png]

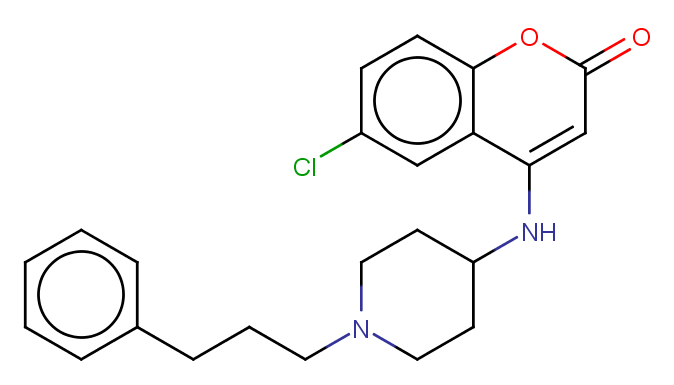

Supplement: RA-011-D1RA00914A-s992 [file RA-011-D1RA00914A-s992.png]

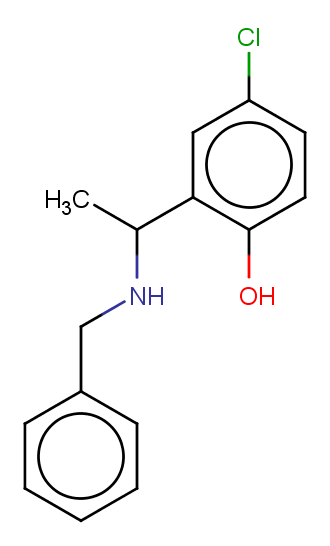

Supplement: RA-011-D1RA00914A-s993 [file RA-011-D1RA00914A-s993.png]

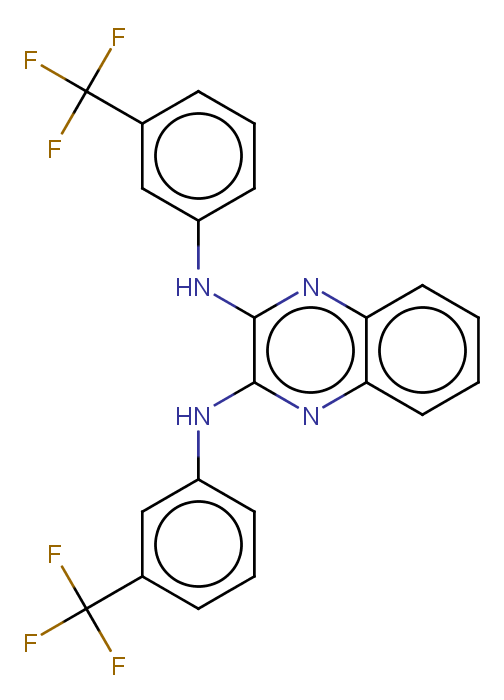

Supplement: RA-011-D1RA00914A-s994 [file RA-011-D1RA00914A-s994.png]

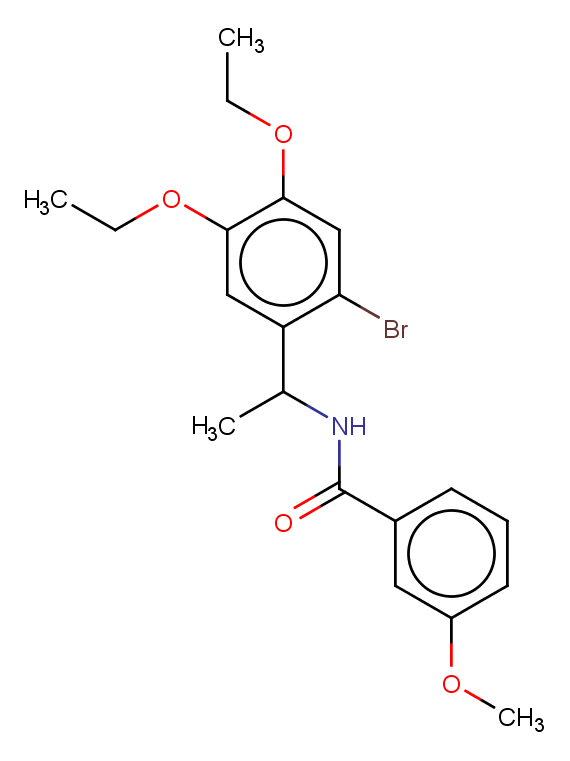

Supplement: RA-011-D1RA00914A-s995 [file RA-011-D1RA00914A-s995.png]

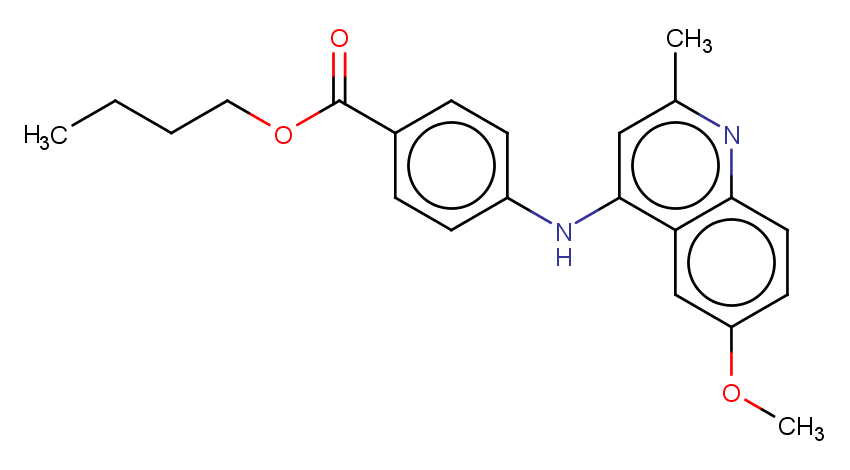

Supplement: RA-011-D1RA00914A-s996 [file RA-011-D1RA00914A-s996.png]

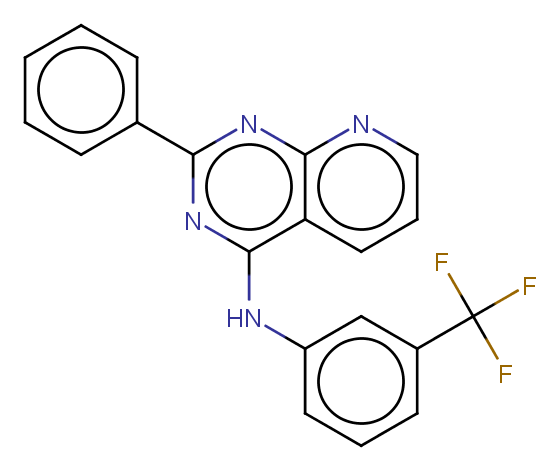

Supplement: RA-011-D1RA00914A-s997 [file RA-011-D1RA00914A-s997.png]

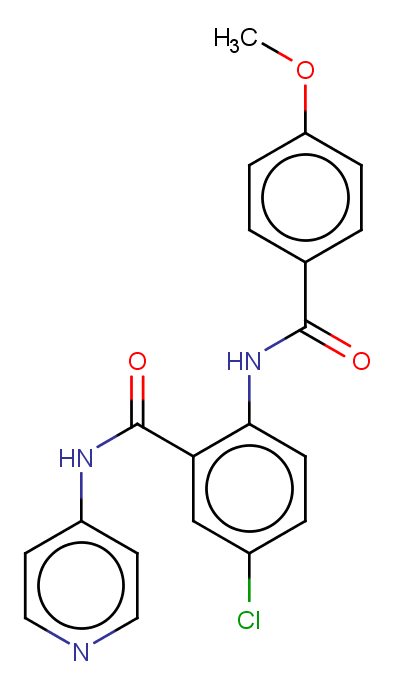

Supplement: RA-011-D1RA00914A-s998 [file RA-011-D1RA00914A-s998.png]

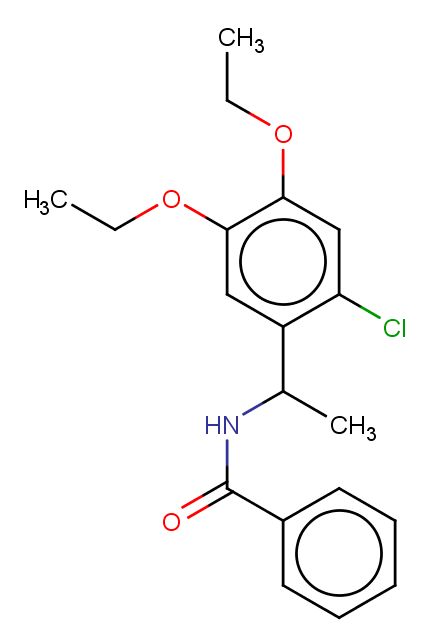

Supplement: RA-011-D1RA00914A-s999 [file RA-011-D1RA00914A-s999.png]

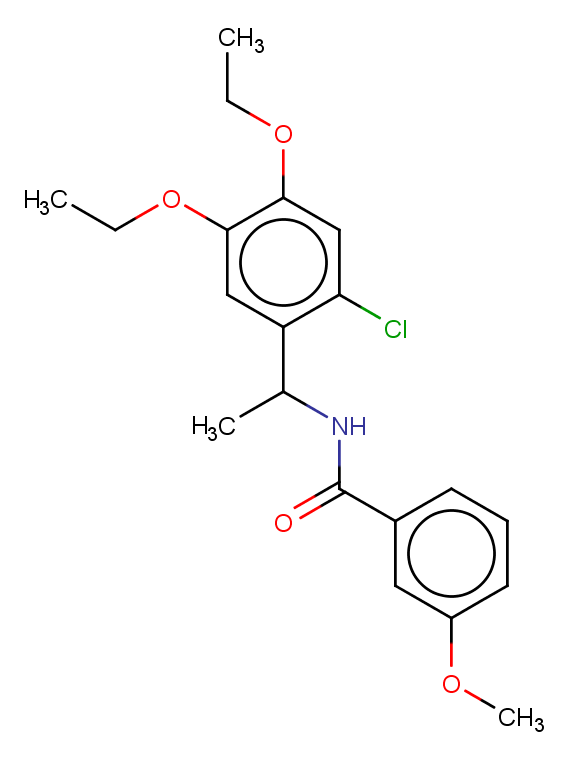

Supplement: RA-011-D1RA00914A-s1000 [file RA-011-D1RA00914A-s1000.png]

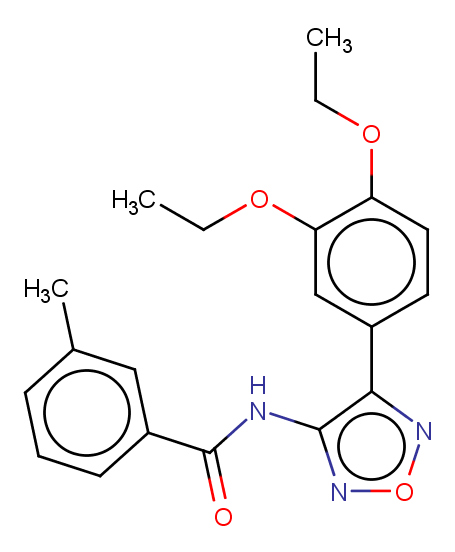

Supplement: RA-011-D1RA00914A-s1001 [file RA-011-D1RA00914A-s1001.png]

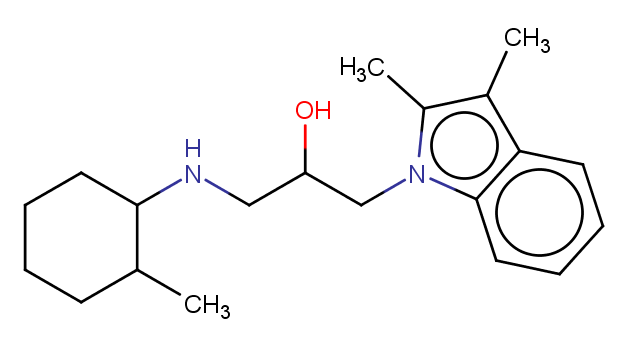

Supplement: RA-011-D1RA00914A-s1002 [file RA-011-D1RA00914A-s1002.png]

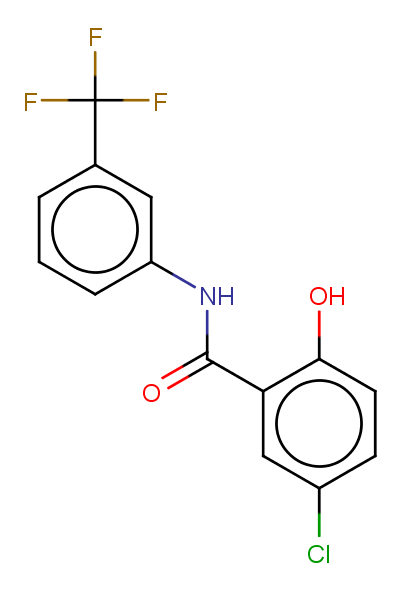

Supplement: RA-011-D1RA00914A-s1003 [file RA-011-D1RA00914A-s1003.png]

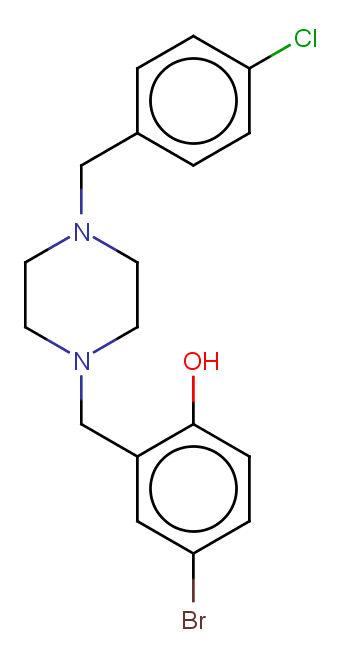

Supplement: RA-011-D1RA00914A-s1004 [file RA-011-D1RA00914A-s1004.png]

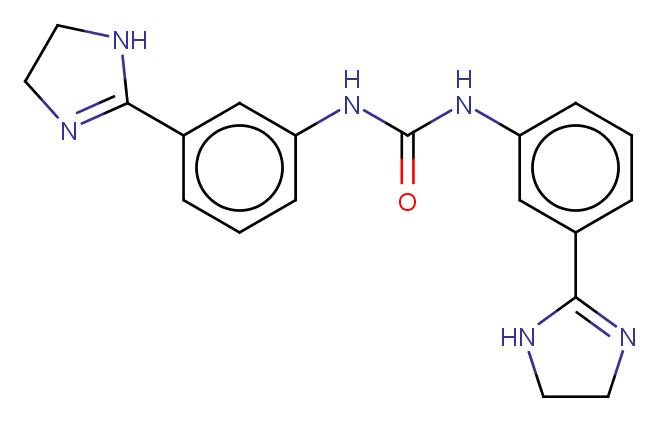

Supplement: RA-011-D1RA00914A-s1005 [file RA-011-D1RA00914A-s1005.png]

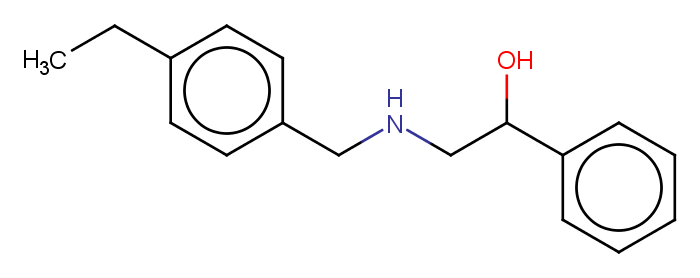

Supplement: RA-011-D1RA00914A-s1006 [file RA-011-D1RA00914A-s1006.png]

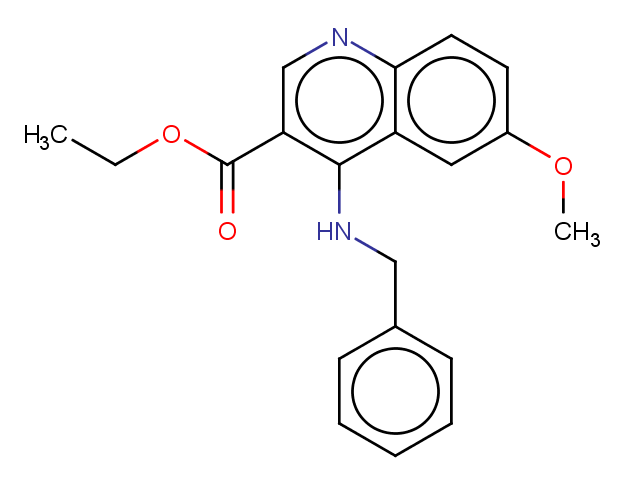

Supplement: RA-011-D1RA00914A-s1007 [file RA-011-D1RA00914A-s1007.png]

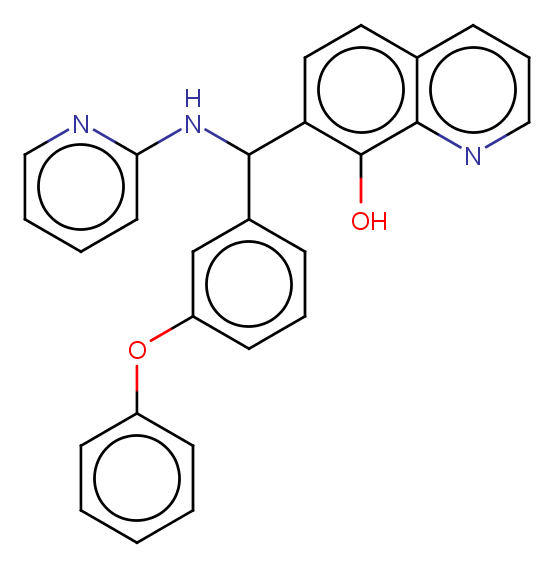

Supplement: RA-011-D1RA00914A-s1008 [file RA-011-D1RA00914A-s1008.png]

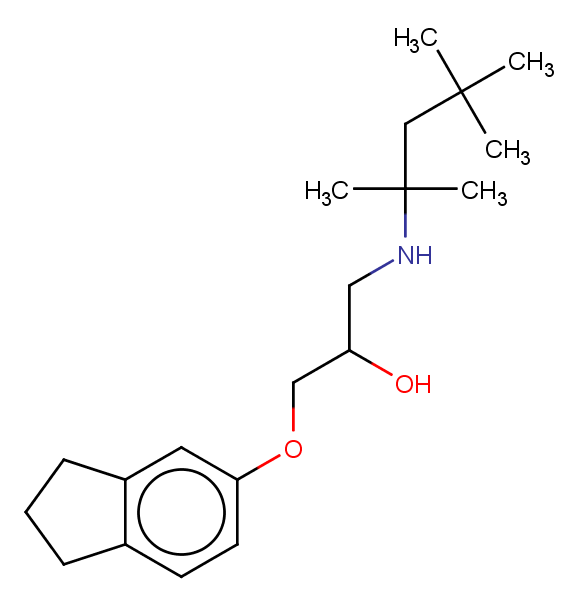

Supplement: RA-011-D1RA00914A-s1009 [file RA-011-D1RA00914A-s1009.png]

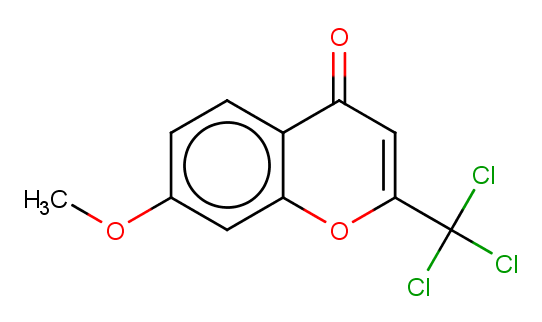

Supplement: RA-011-D1RA00914A-s1010 [file RA-011-D1RA00914A-s1010.png]

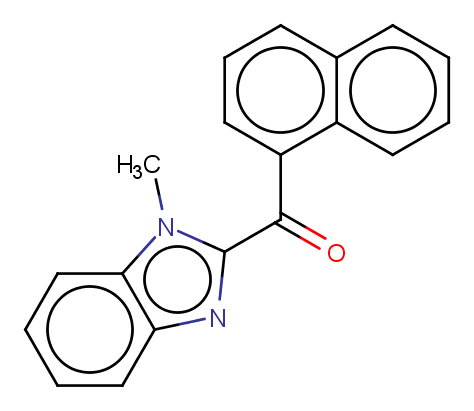

Supplement: RA-011-D1RA00914A-s1011 [file RA-011-D1RA00914A-s1011.png]

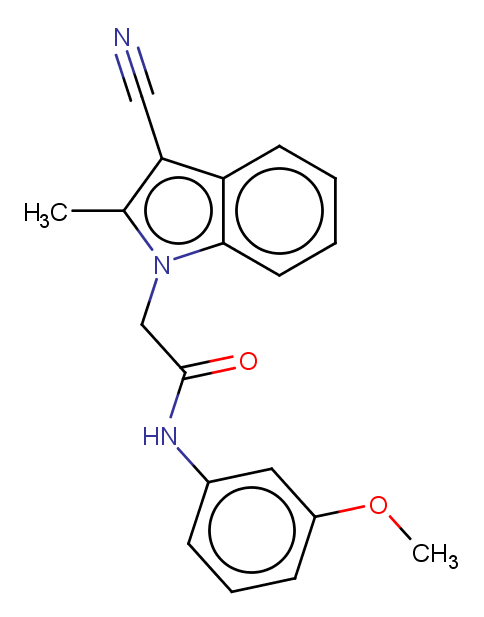

Supplement: RA-011-D1RA00914A-s1012 [file RA-011-D1RA00914A-s1012.png]

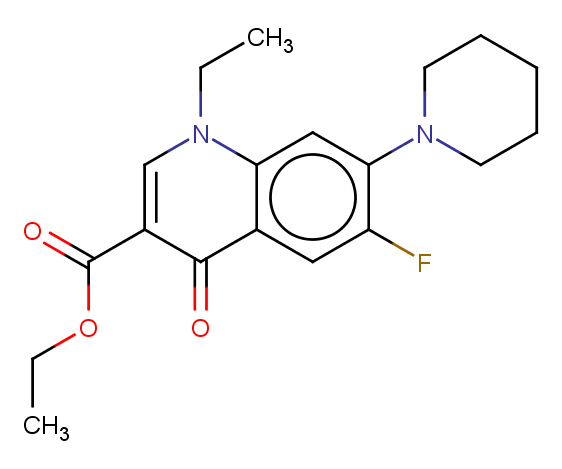

Supplement: RA-011-D1RA00914A-s1013 [file RA-011-D1RA00914A-s1013.png]

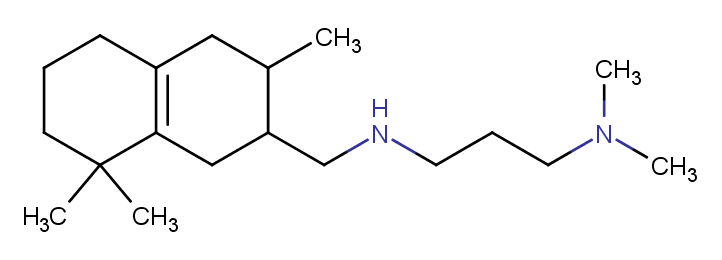

Supplement: RA-011-D1RA00914A-s1014 [file RA-011-D1RA00914A-s1014.png]

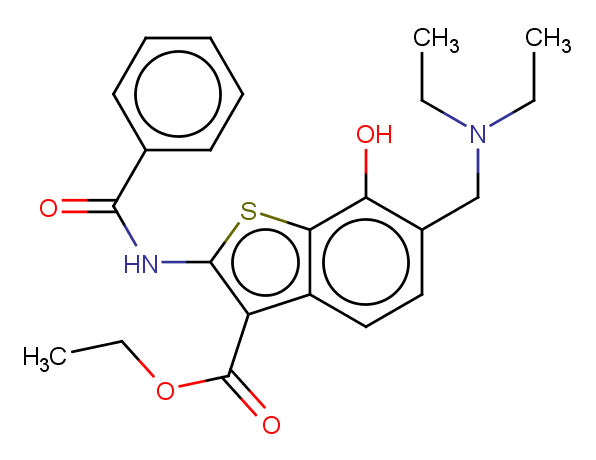

Supplement: RA-011-D1RA00914A-s1015 [file RA-011-D1RA00914A-s1015.png]

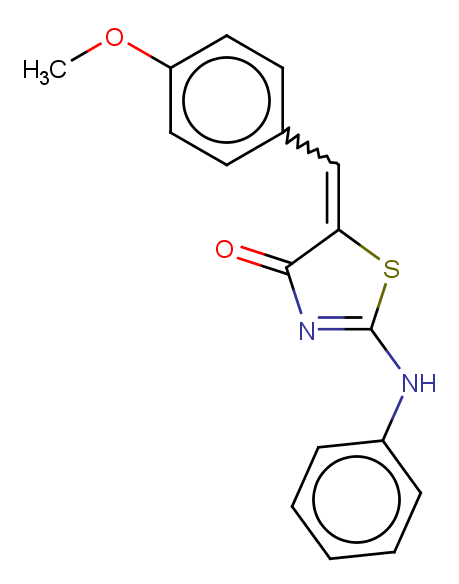

Supplement: RA-011-D1RA00914A-s1016 [file RA-011-D1RA00914A-s1016.png]

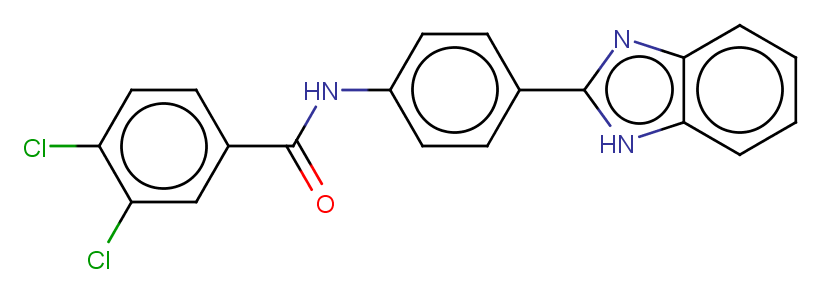

Supplement: RA-011-D1RA00914A-s1017 [file RA-011-D1RA00914A-s1017.png]

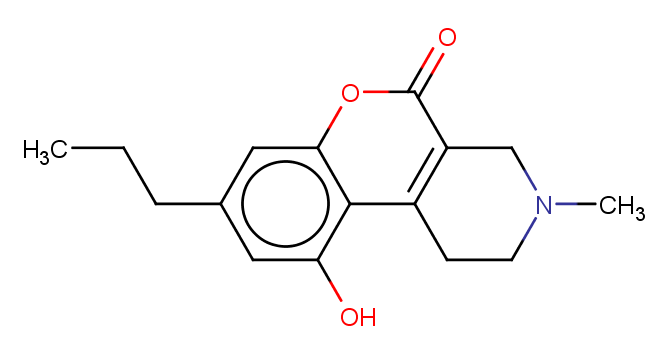

Supplement: RA-011-D1RA00914A-s1018 [file RA-011-D1RA00914A-s1018.png]

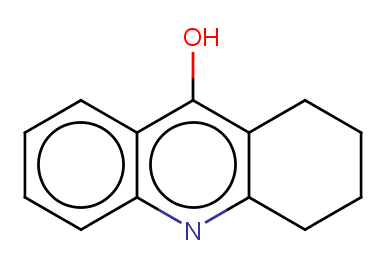

Supplement: RA-011-D1RA00914A-s1019 [file RA-011-D1RA00914A-s1019.png]

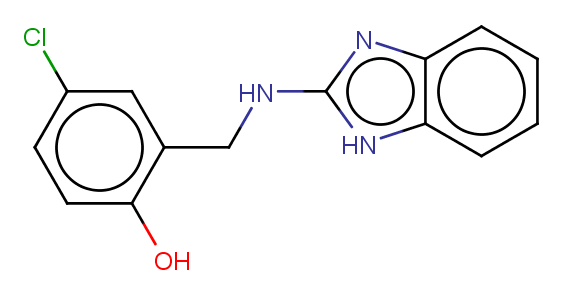

Supplement: RA-011-D1RA00914A-s1020 [file RA-011-D1RA00914A-s1020.png]

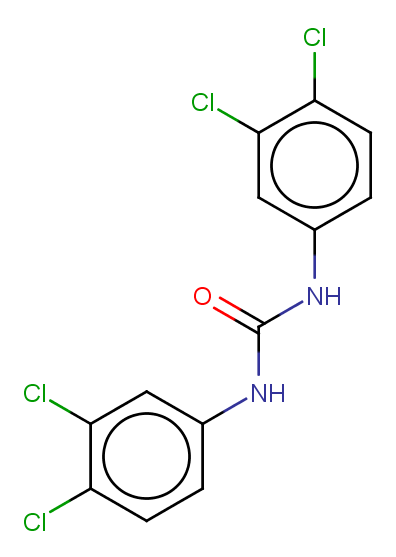

Supplement: RA-011-D1RA00914A-s1021 [file RA-011-D1RA00914A-s1021.png]

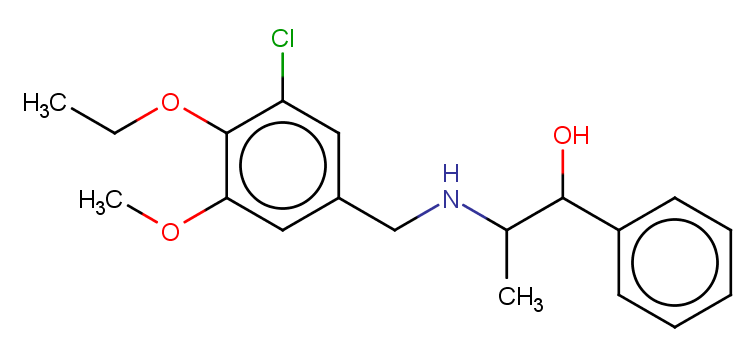

Supplement: RA-011-D1RA00914A-s1022 [file RA-011-D1RA00914A-s1022.png]

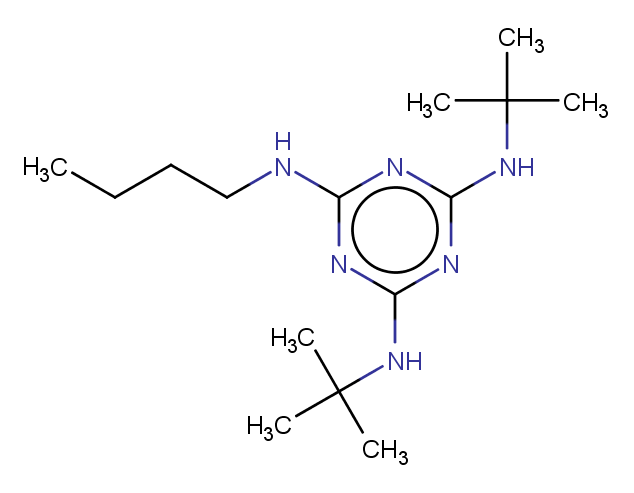

Supplement: RA-011-D1RA00914A-s1023 [file RA-011-D1RA00914A-s1023.png]

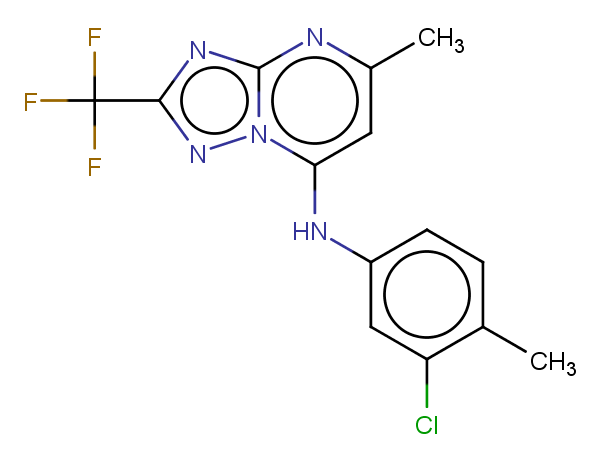

Supplement: RA-011-D1RA00914A-s1024 [file RA-011-D1RA00914A-s1024.png]

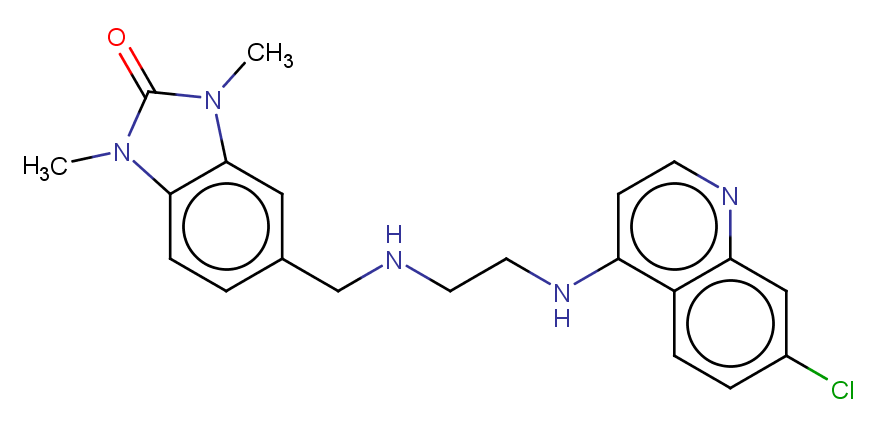

Supplement: RA-011-D1RA00914A-s1025 [file RA-011-D1RA00914A-s1025.png]

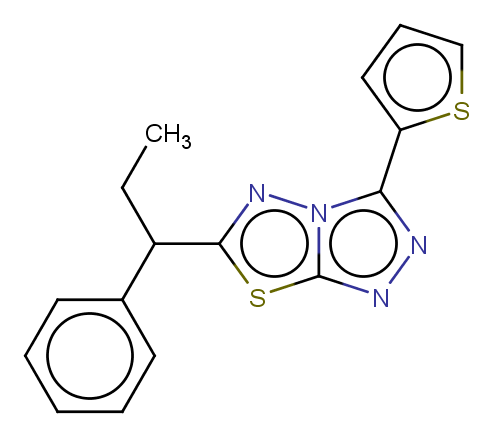

Supplement: RA-011-D1RA00914A-s1026 [file RA-011-D1RA00914A-s1026.png]

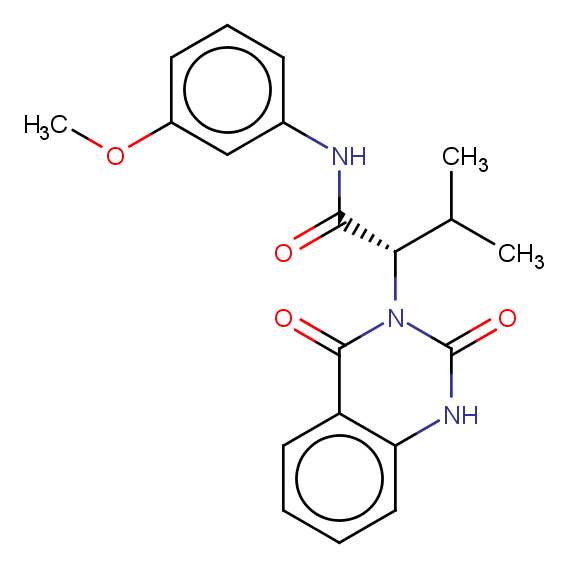

Supplement: RA-011-D1RA00914A-s1027 [file RA-011-D1RA00914A-s1027.png]

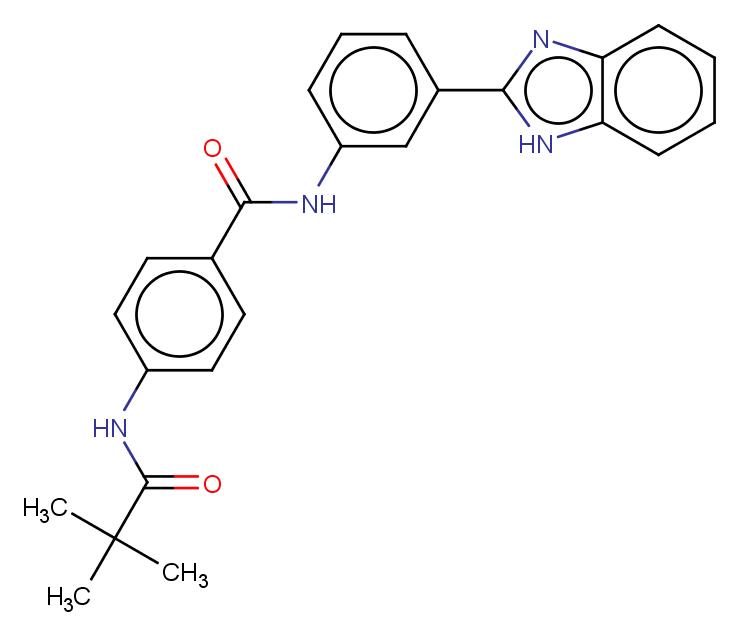

Supplement: RA-011-D1RA00914A-s1028 [file RA-011-D1RA00914A-s1028.png]

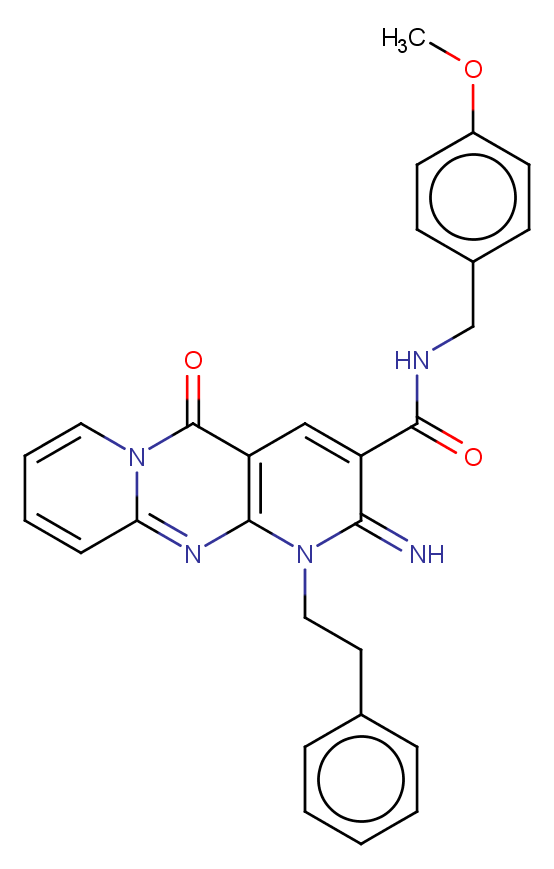

Supplement: RA-011-D1RA00914A-s1029 [file RA-011-D1RA00914A-s1029.png]

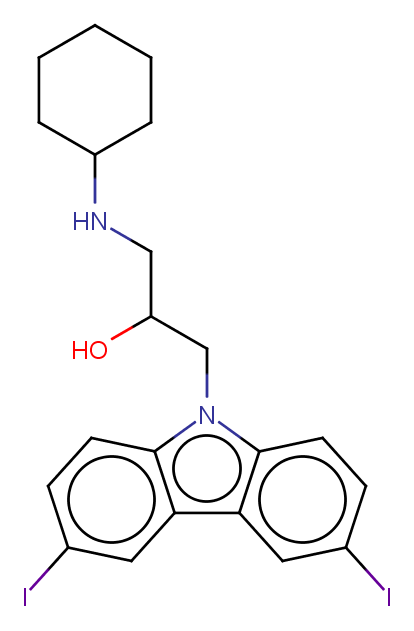

Supplement: RA-011-D1RA00914A-s1030 [file RA-011-D1RA00914A-s1030.png]

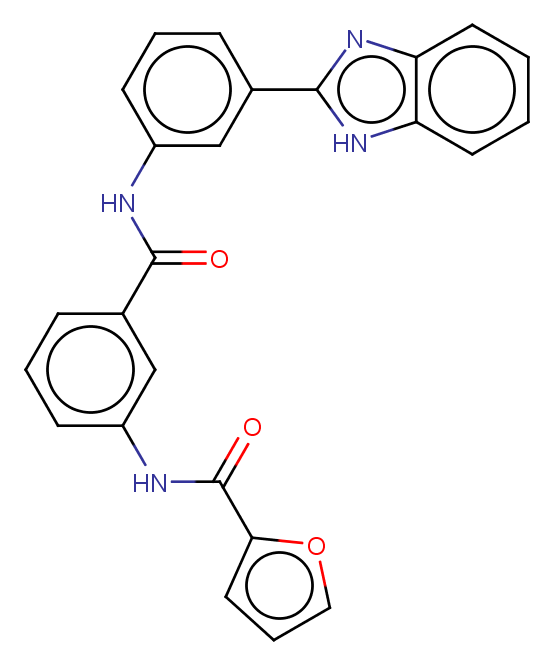

Supplement: RA-011-D1RA00914A-s1031 [file RA-011-D1RA00914A-s1031.png]

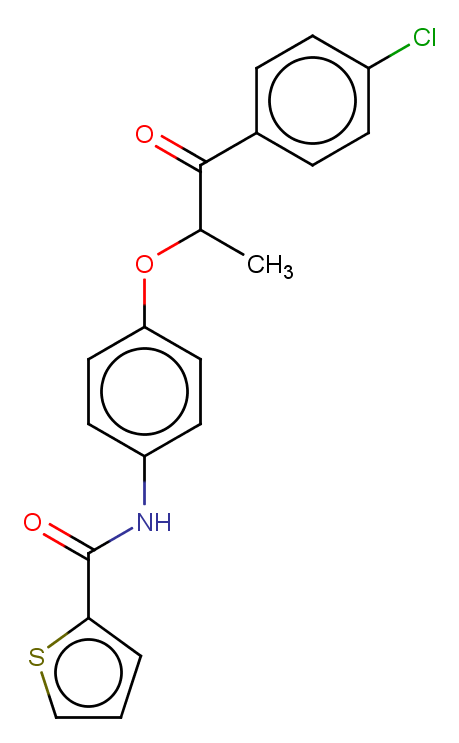

Supplement: RA-011-D1RA00914A-s1032 [file RA-011-D1RA00914A-s1032.png]

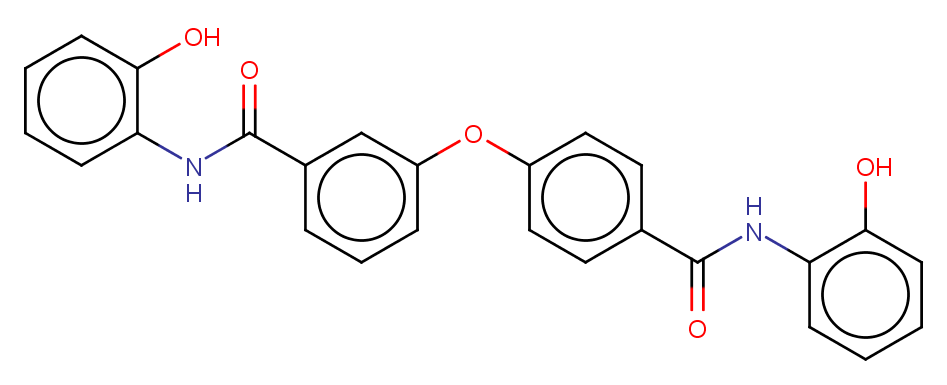

Supplement: RA-011-D1RA00914A-s1033 [file RA-011-D1RA00914A-s1033.png]

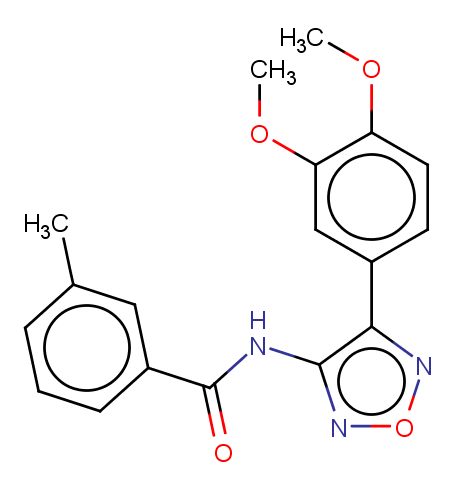

Supplement: RA-011-D1RA00914A-s1034 [file RA-011-D1RA00914A-s1034.png]

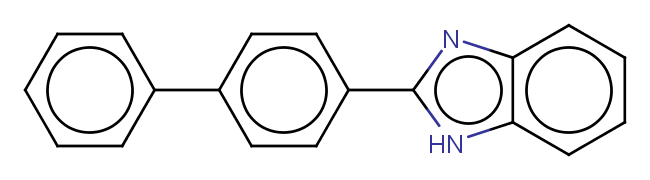

Supplement: RA-011-D1RA00914A-s1035 [file RA-011-D1RA00914A-s1035.png]

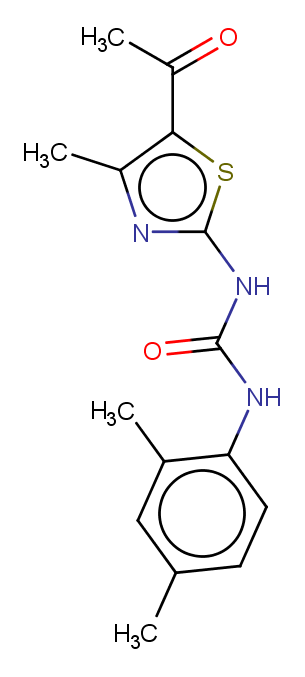

Supplement: RA-011-D1RA00914A-s1036 [file RA-011-D1RA00914A-s1036.png]

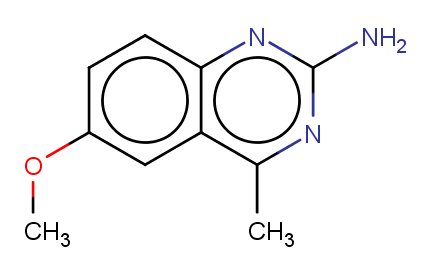

Supplement: RA-011-D1RA00914A-s1037 [file RA-011-D1RA00914A-s1037.png]

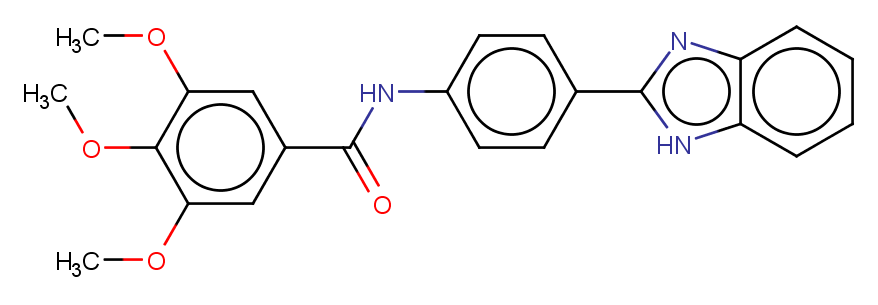

Supplement: RA-011-D1RA00914A-s1038 [file RA-011-D1RA00914A-s1038.png]

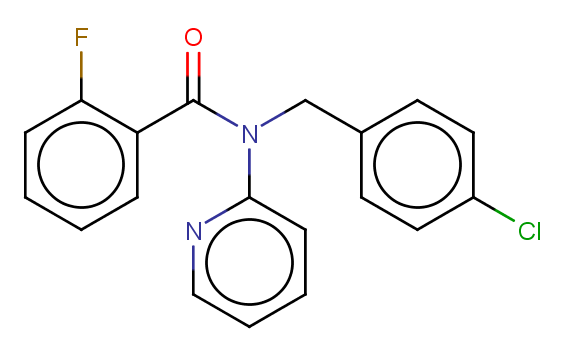

Supplement: RA-011-D1RA00914A-s1039 [file RA-011-D1RA00914A-s1039.png]

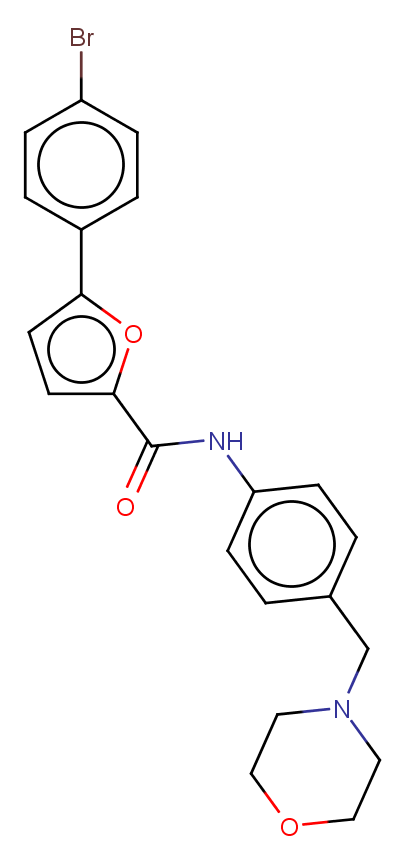

Supplement: RA-011-D1RA00914A-s1040 [file RA-011-D1RA00914A-s1040.png]

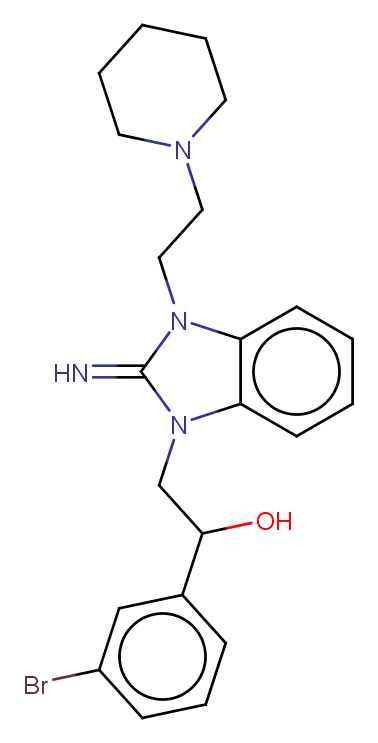

Supplement: RA-011-D1RA00914A-s1041 [file RA-011-D1RA00914A-s1041.png]

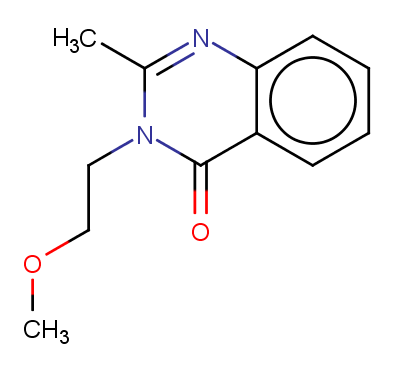

Supplement: RA-011-D1RA00914A-s1042 [file RA-011-D1RA00914A-s1042.png]

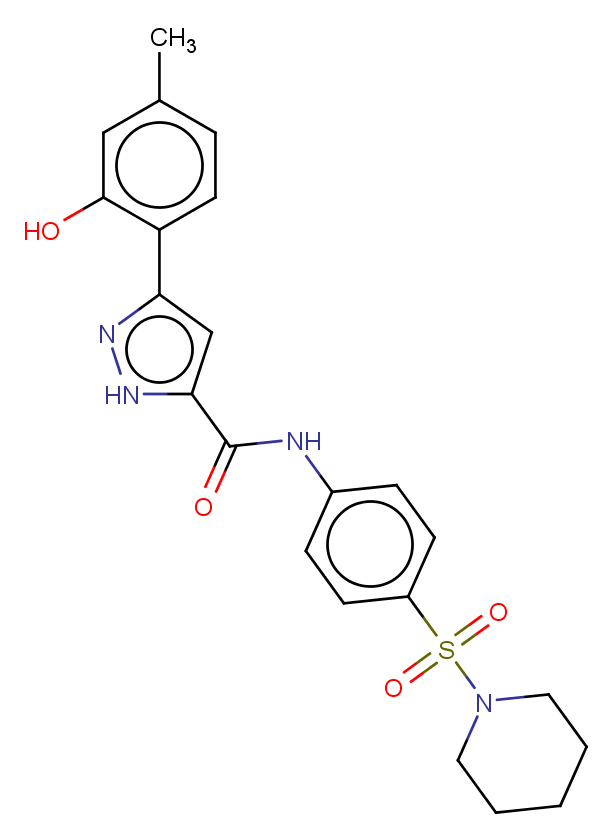

Supplement: RA-011-D1RA00914A-s1043 [file RA-011-D1RA00914A-s1043.png]

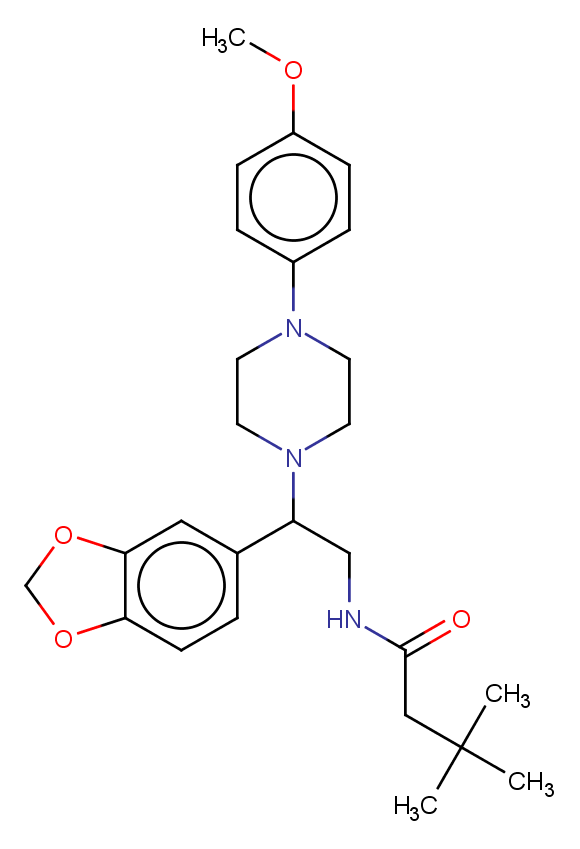

Supplement: RA-011-D1RA00914A-s1044 [file RA-011-D1RA00914A-s1044.png]

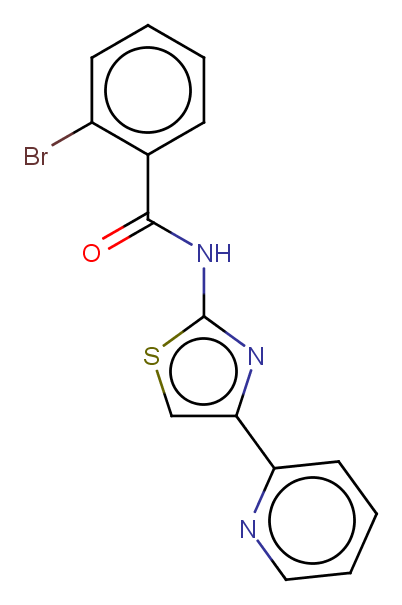

Supplement: RA-011-D1RA00914A-s1045 [file RA-011-D1RA00914A-s1045.png]

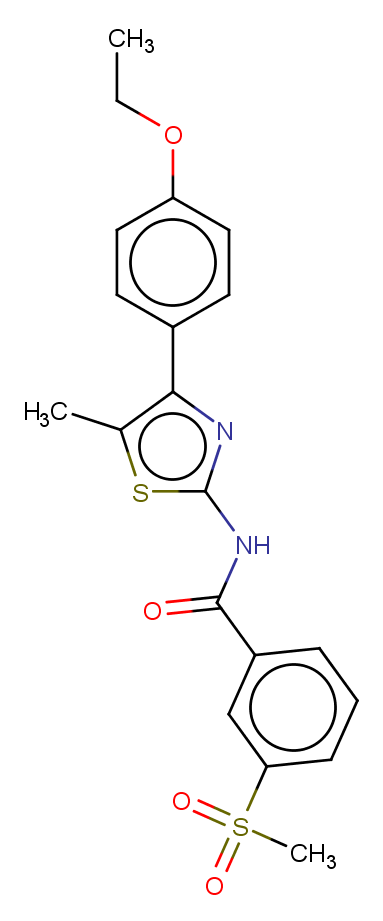

Supplement: RA-011-D1RA00914A-s1046 [file RA-011-D1RA00914A-s1046.png]

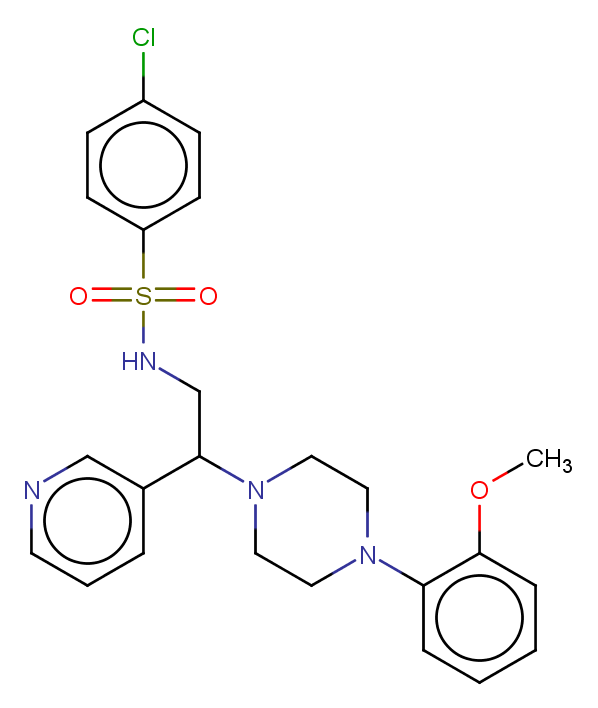

Supplement: RA-011-D1RA00914A-s1047 [file RA-011-D1RA00914A-s1047.png]

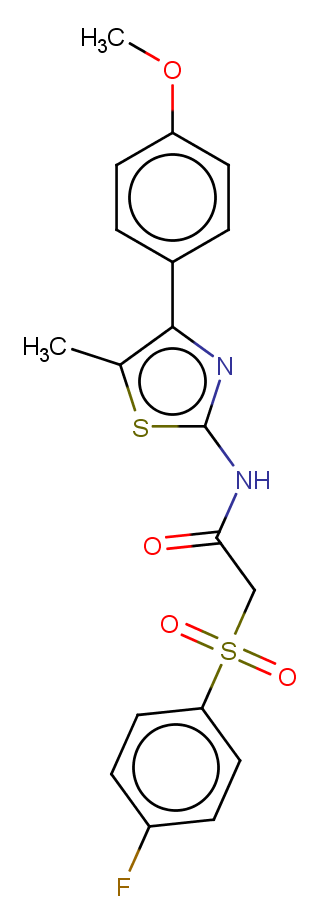

Supplement: RA-011-D1RA00914A-s1048 [file RA-011-D1RA00914A-s1048.png]

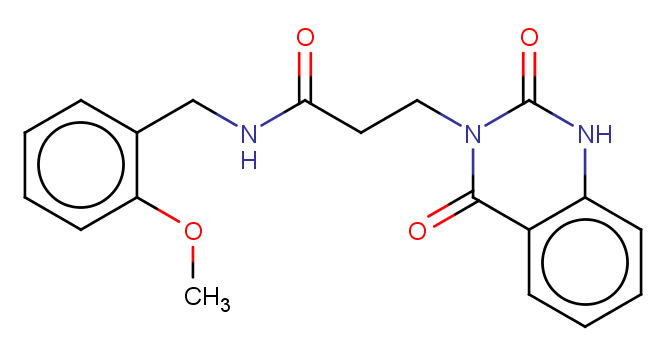

Supplement: RA-011-D1RA00914A-s1049 [file RA-011-D1RA00914A-s1049.png]

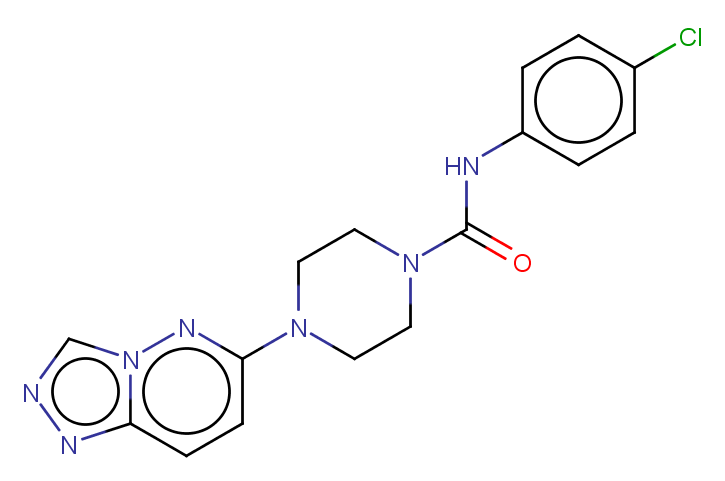

Supplement: RA-011-D1RA00914A-s1050 [file RA-011-D1RA00914A-s1050.png]

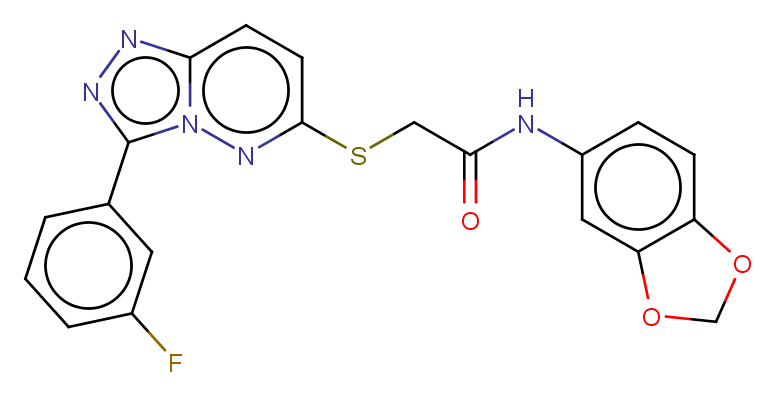

Supplement: RA-011-D1RA00914A-s1051 [file RA-011-D1RA00914A-s1051.png]

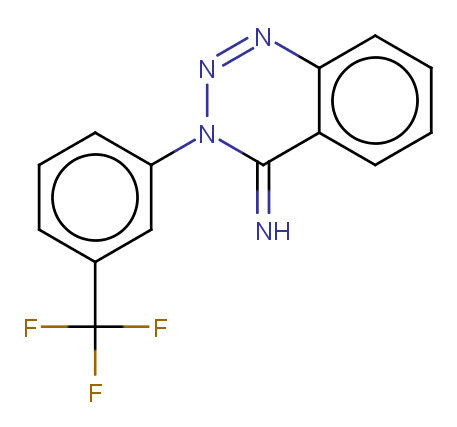

Supplement: RA-011-D1RA00914A-s1052 [file RA-011-D1RA00914A-s1052.png]

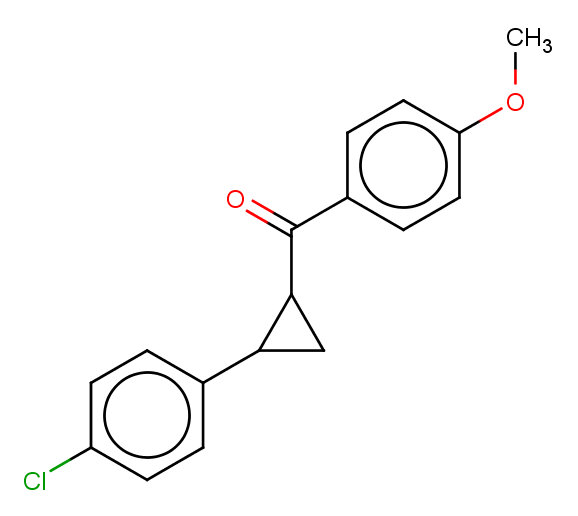

Supplement: RA-011-D1RA00914A-s1053 [file RA-011-D1RA00914A-s1053.png]

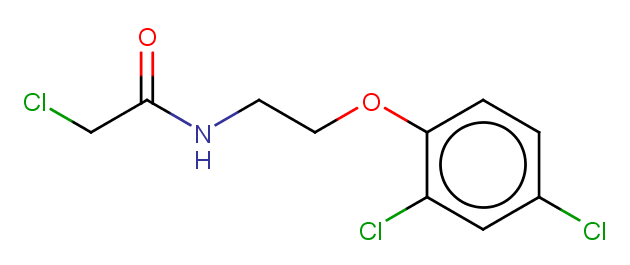

Supplement: RA-011-D1RA00914A-s1054 [file RA-011-D1RA00914A-s1054.png]

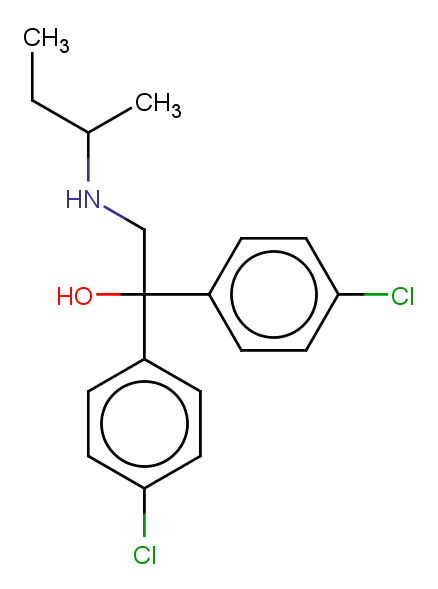

Supplement: RA-011-D1RA00914A-s1055 [file RA-011-D1RA00914A-s1055.png]

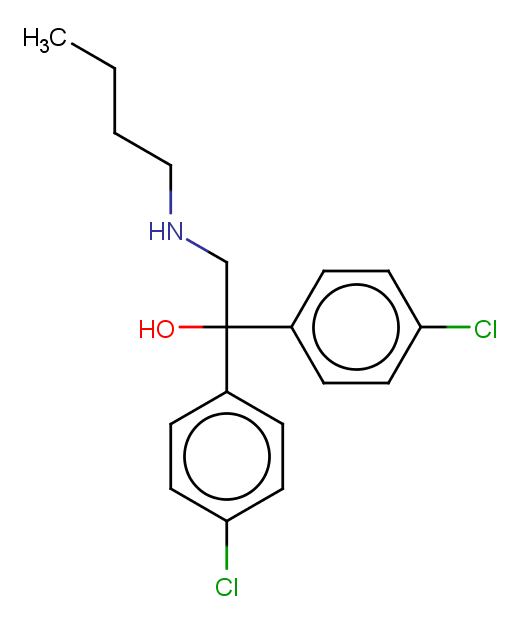

Supplement: RA-011-D1RA00914A-s1056 [file RA-011-D1RA00914A-s1056.png]

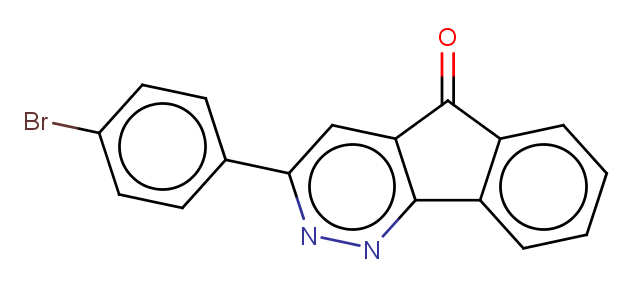

Supplement: RA-011-D1RA00914A-s1057 [file RA-011-D1RA00914A-s1057.png]

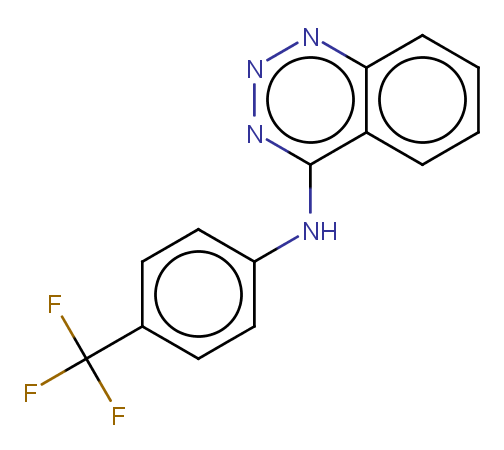

Supplement: RA-011-D1RA00914A-s1058 [file RA-011-D1RA00914A-s1058.png]

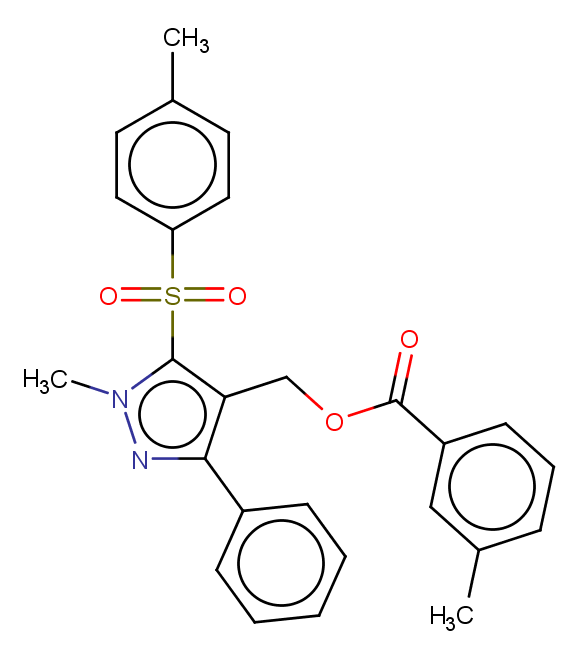

Supplement: RA-011-D1RA00914A-s1059 [file RA-011-D1RA00914A-s1059.png]

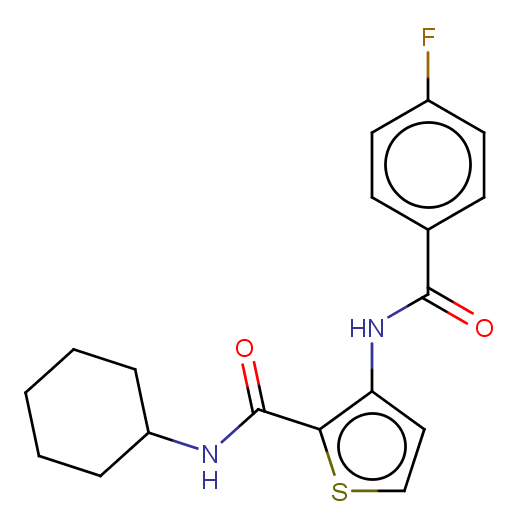

Supplement: RA-011-D1RA00914A-s1060 [file RA-011-D1RA00914A-s1060.png]

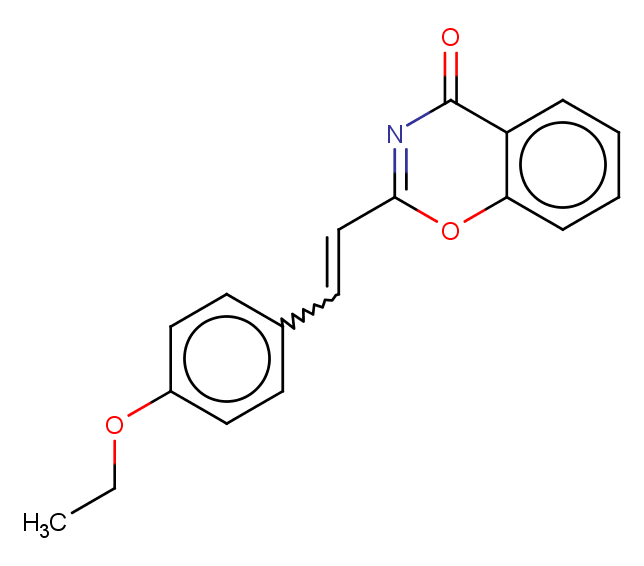

Supplement: RA-011-D1RA00914A-s1061 [file RA-011-D1RA00914A-s1061.png]

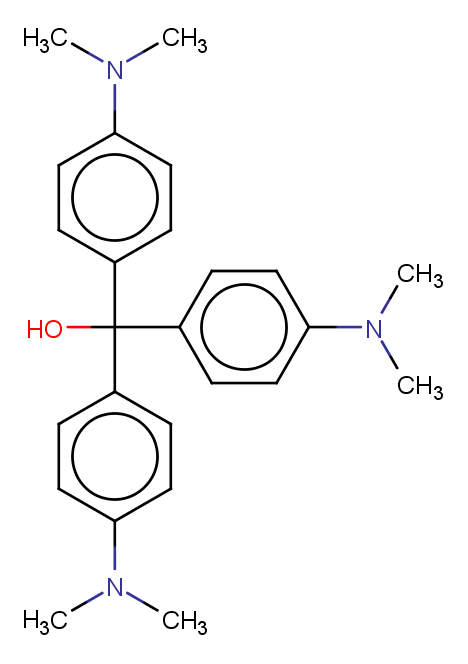

Supplement: RA-011-D1RA00914A-s1062 [file RA-011-D1RA00914A-s1062.png]

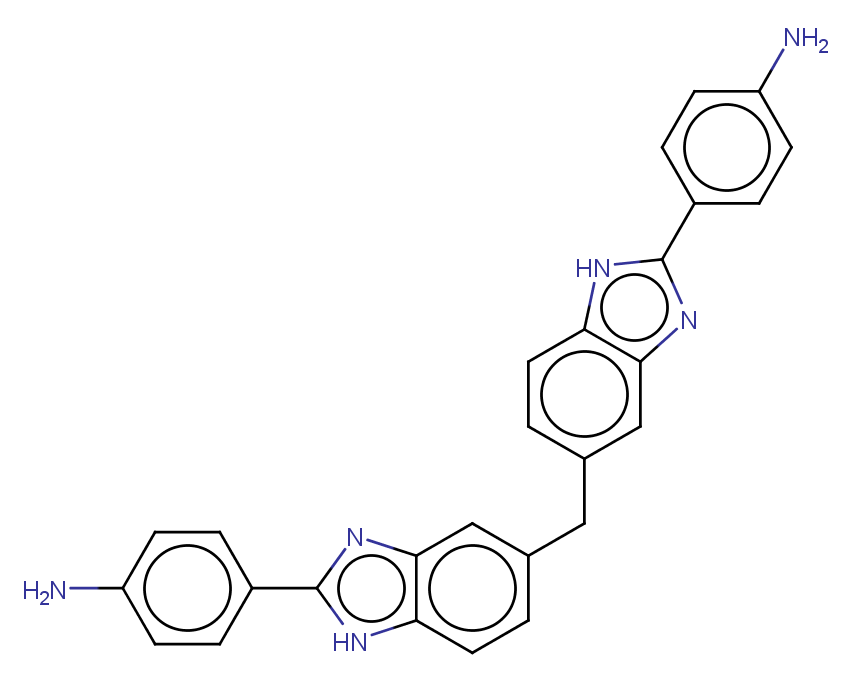

Supplement: RA-011-D1RA00914A-s1063 [file RA-011-D1RA00914A-s1063.png]

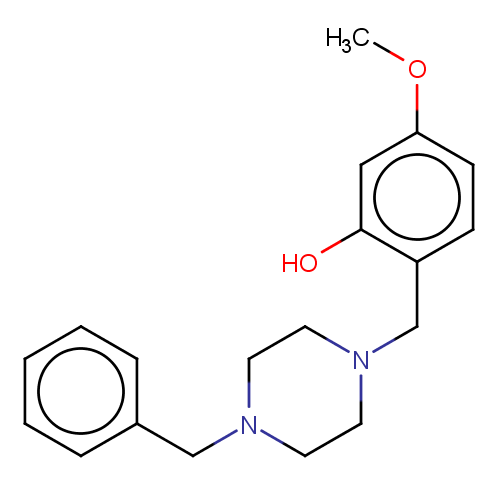

Supplement: RA-011-D1RA00914A-s1064 [file RA-011-D1RA00914A-s1064.png]

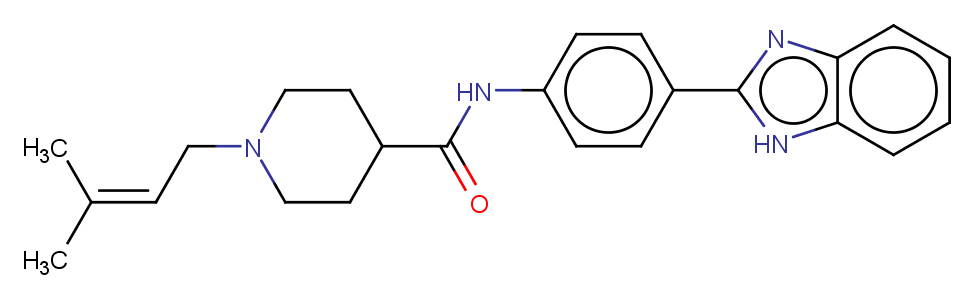

Supplement: RA-011-D1RA00914A-s1065 [file RA-011-D1RA00914A-s1065.png]

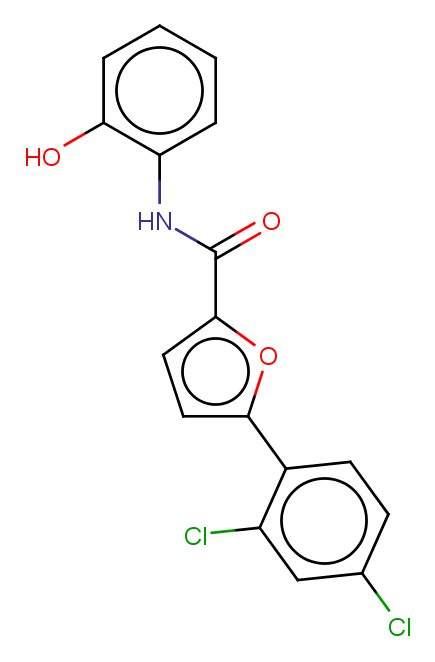

Supplement: RA-011-D1RA00914A-s1066 [file RA-011-D1RA00914A-s1066.png]

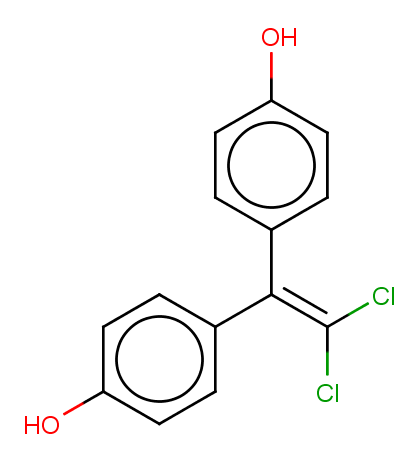

Supplement: RA-011-D1RA00914A-s1067 [file RA-011-D1RA00914A-s1067.png]

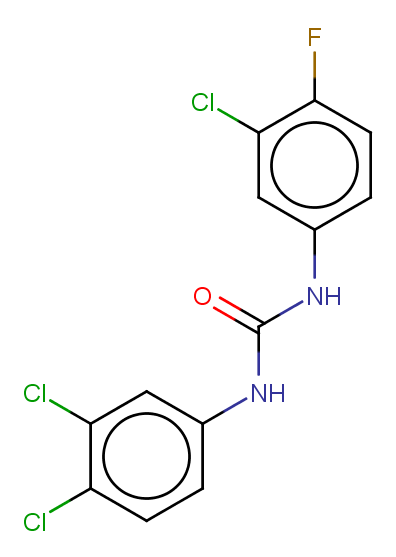

Supplement: RA-011-D1RA00914A-s1068 [file RA-011-D1RA00914A-s1068.png]

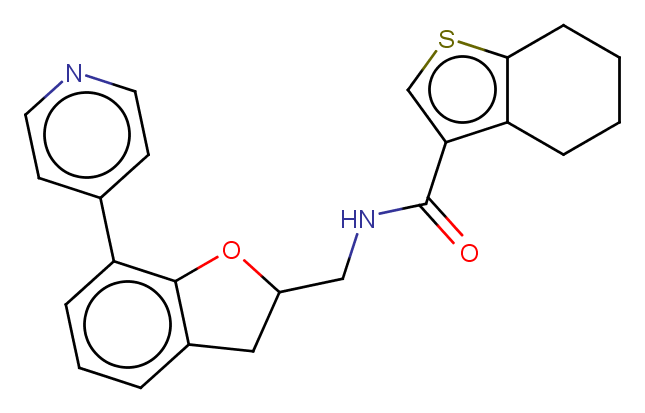

Supplement: RA-011-D1RA00914A-s1069 [file RA-011-D1RA00914A-s1069.png]

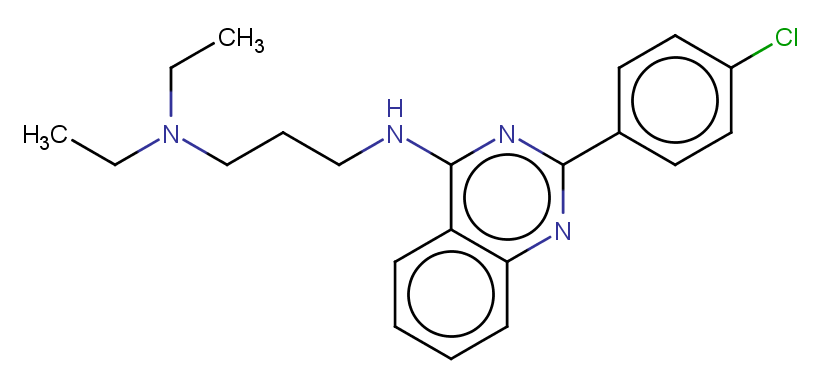

Supplement: RA-011-D1RA00914A-s1070 [file RA-011-D1RA00914A-s1070.png]

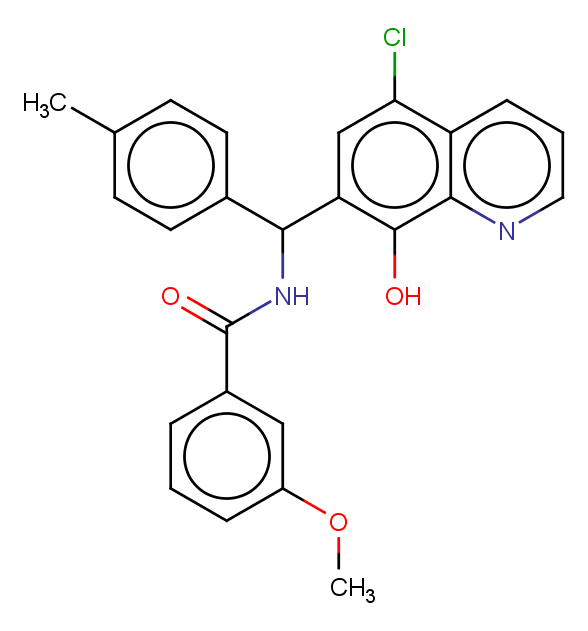

Supplement: RA-011-D1RA00914A-s1071 [file RA-011-D1RA00914A-s1071.png]
