# Supplementary figures and images for: Improved herbicide discovery using physico-chemical rules refined by antimalarial library screening (part 8 of 14)
Source: RSC Adv. 2021 Feb 23;11(15):8459–67. doi: 10.1039/d1ra00914a (PMC8695207; doi:10.1039/d1ra00914a)

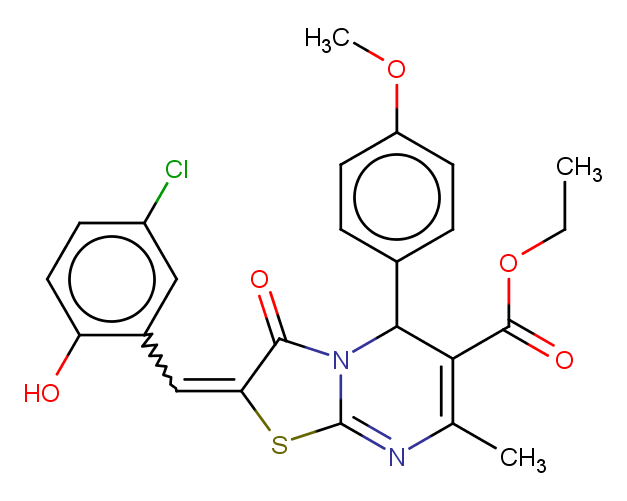

Supplement: RA-011-D1RA00914A-s1072 [file RA-011-D1RA00914A-s1072.png]

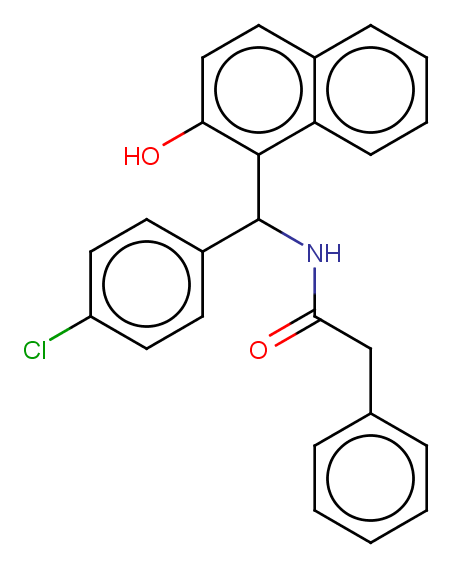

Supplement: RA-011-D1RA00914A-s1073 [file RA-011-D1RA00914A-s1073.png]

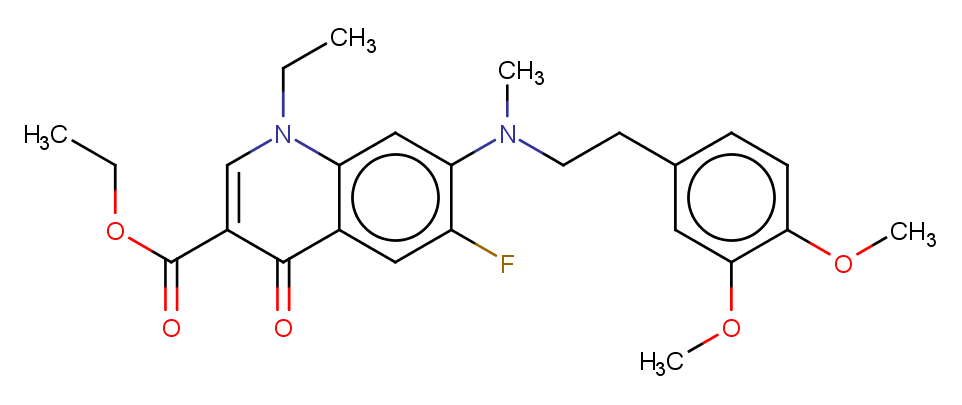

Supplement: RA-011-D1RA00914A-s1074 [file RA-011-D1RA00914A-s1074.png]

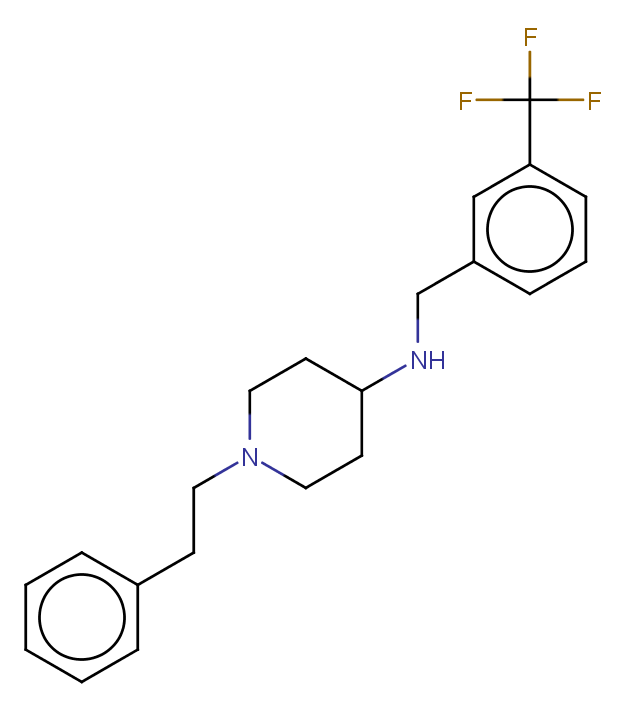

Supplement: RA-011-D1RA00914A-s1075 [file RA-011-D1RA00914A-s1075.png]

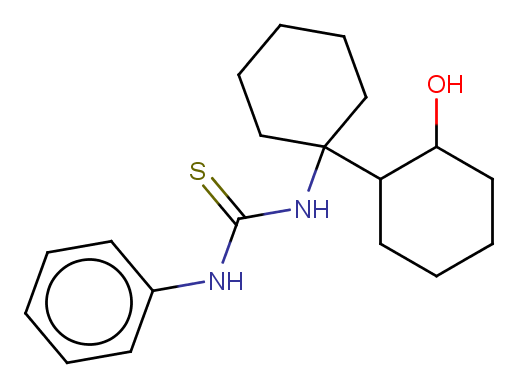

Supplement: RA-011-D1RA00914A-s1076 [file RA-011-D1RA00914A-s1076.png]

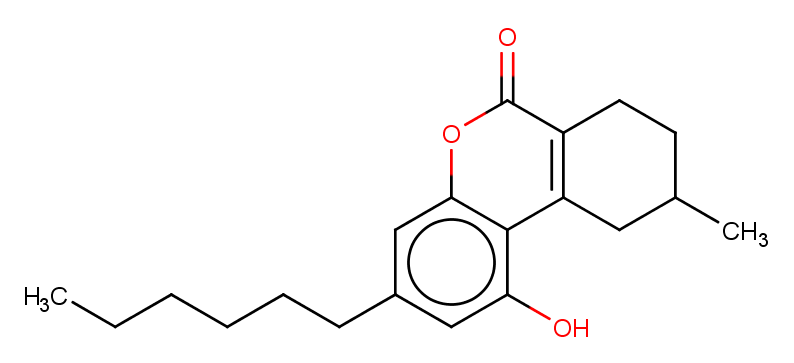

Supplement: RA-011-D1RA00914A-s1077 [file RA-011-D1RA00914A-s1077.png]

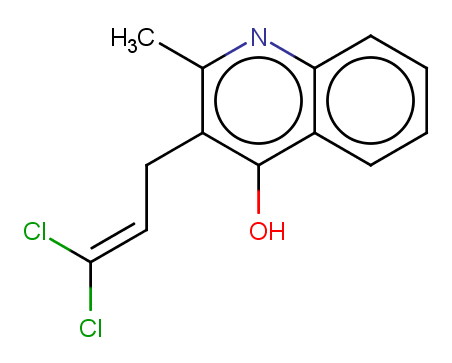

Supplement: RA-011-D1RA00914A-s1078 [file RA-011-D1RA00914A-s1078.png]

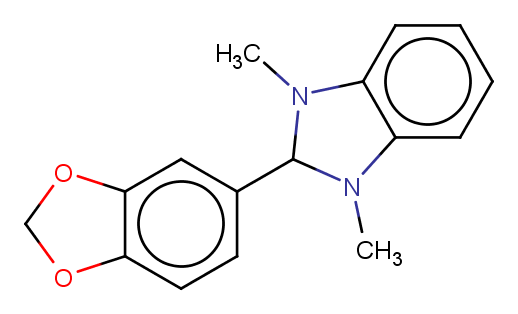

Supplement: RA-011-D1RA00914A-s1079 [file RA-011-D1RA00914A-s1079.png]

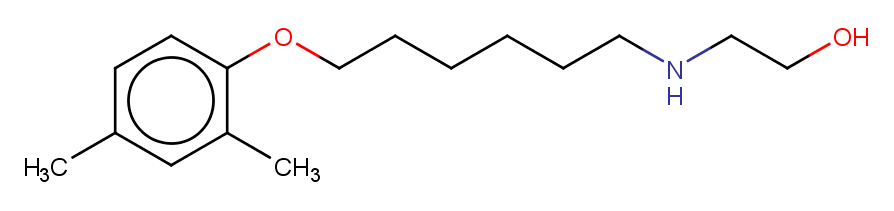

Supplement: RA-011-D1RA00914A-s1080 [file RA-011-D1RA00914A-s1080.png]

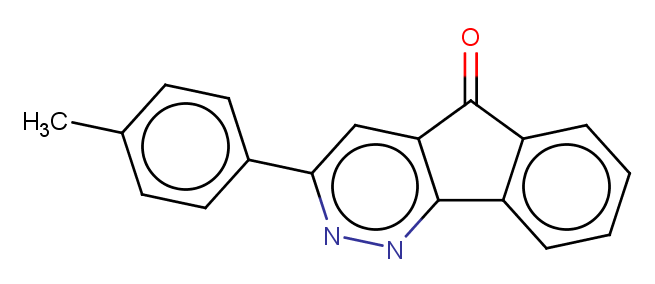

Supplement: RA-011-D1RA00914A-s1081 [file RA-011-D1RA00914A-s1081.png]

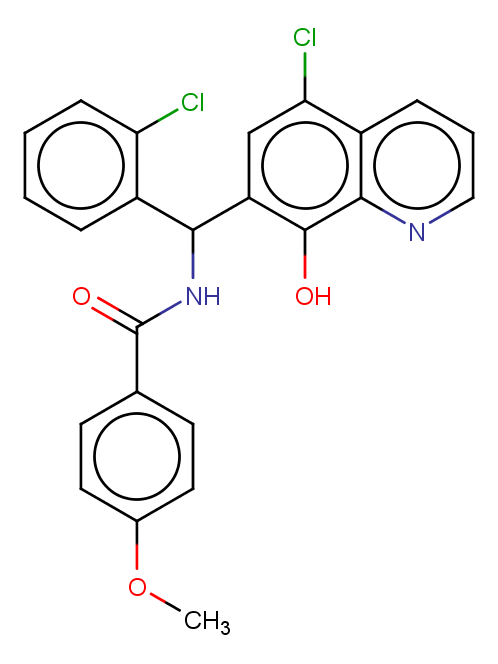

Supplement: RA-011-D1RA00914A-s1082 [file RA-011-D1RA00914A-s1082.png]

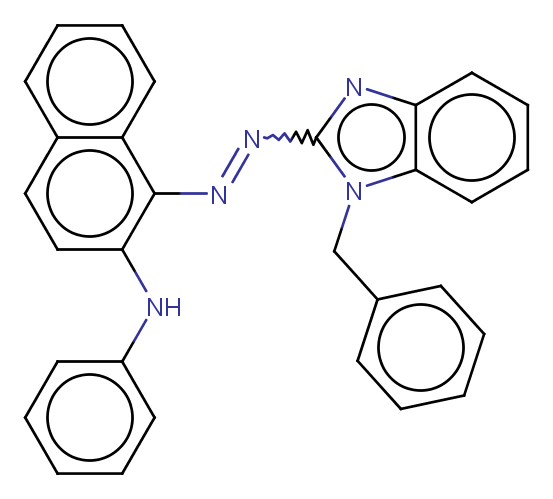

Supplement: RA-011-D1RA00914A-s1083 [file RA-011-D1RA00914A-s1083.png]

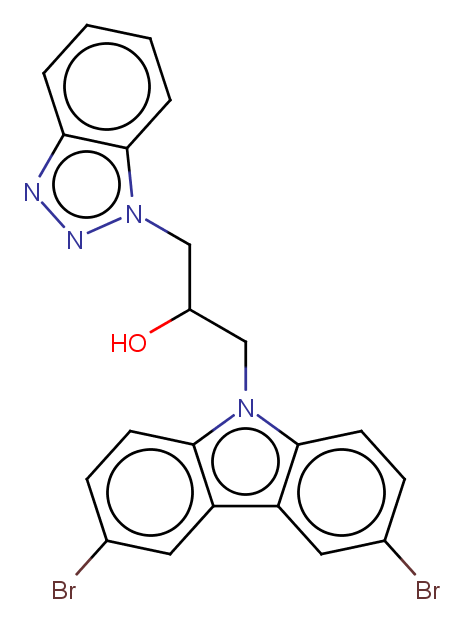

Supplement: RA-011-D1RA00914A-s1084 [file RA-011-D1RA00914A-s1084.png]

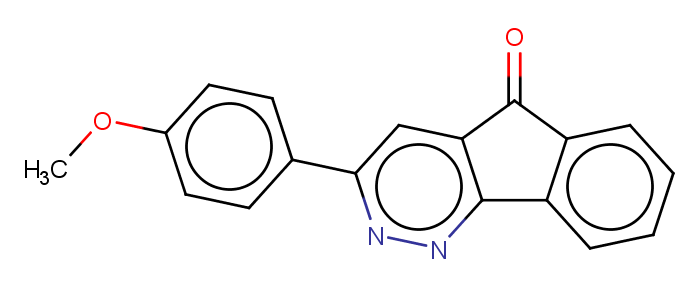

Supplement: RA-011-D1RA00914A-s1085 [file RA-011-D1RA00914A-s1085.png]

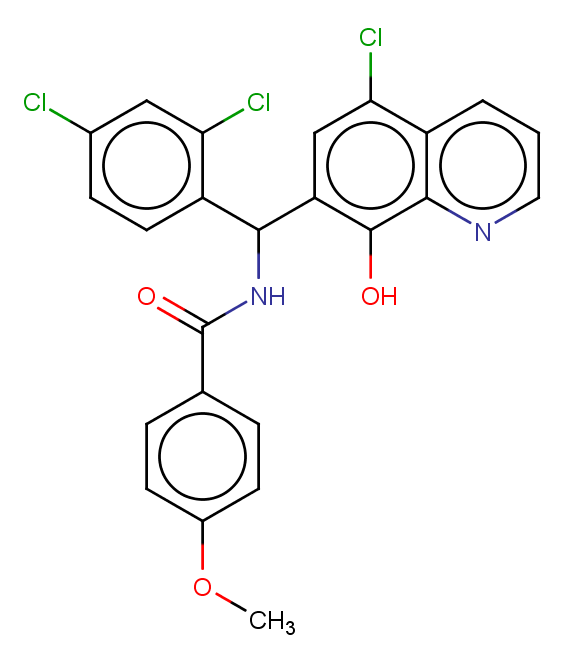

Supplement: RA-011-D1RA00914A-s1086 [file RA-011-D1RA00914A-s1086.png]

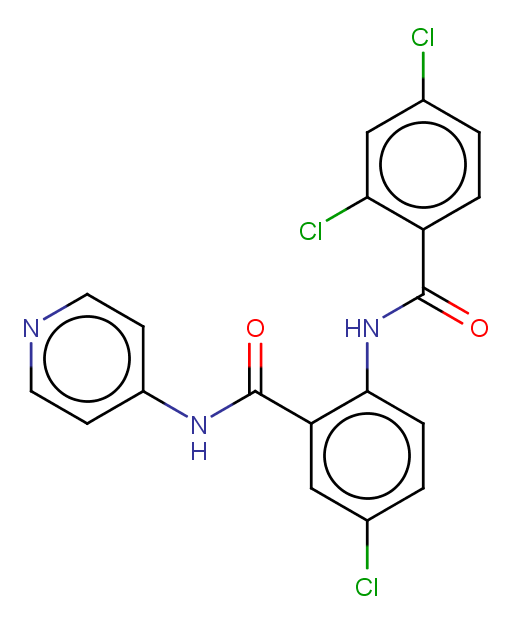

Supplement: RA-011-D1RA00914A-s1087 [file RA-011-D1RA00914A-s1087.png]

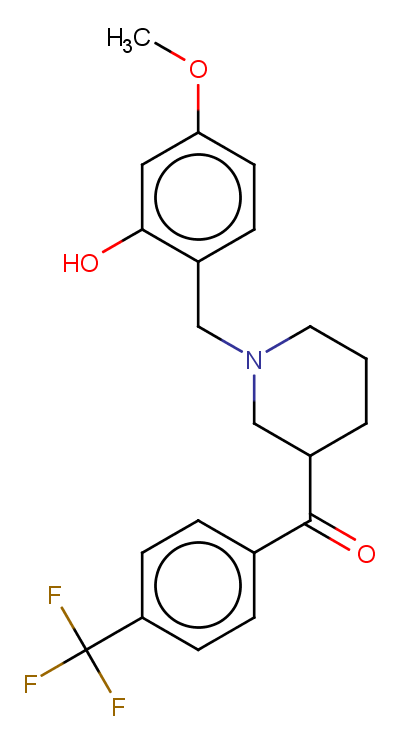

Supplement: RA-011-D1RA00914A-s1088 [file RA-011-D1RA00914A-s1088.png]

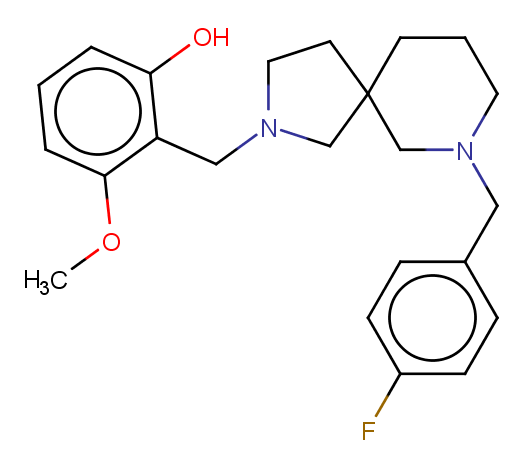

Supplement: RA-011-D1RA00914A-s1089 [file RA-011-D1RA00914A-s1089.png]

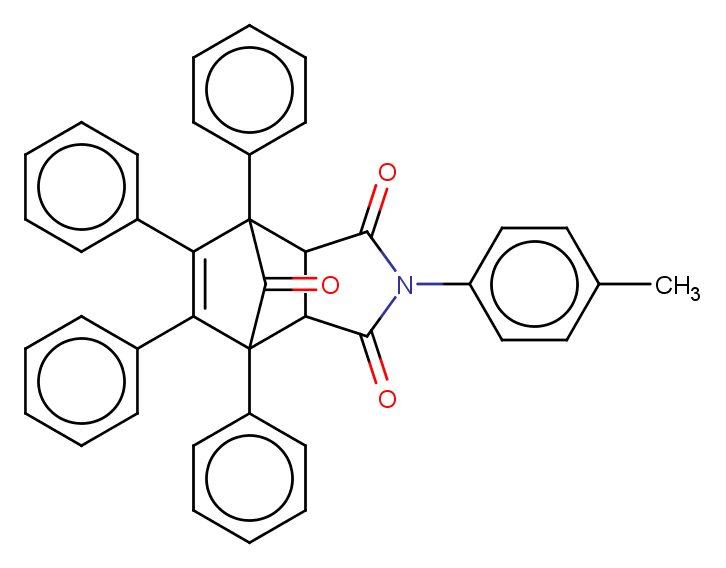

Supplement: RA-011-D1RA00914A-s1090 [file RA-011-D1RA00914A-s1090.png]

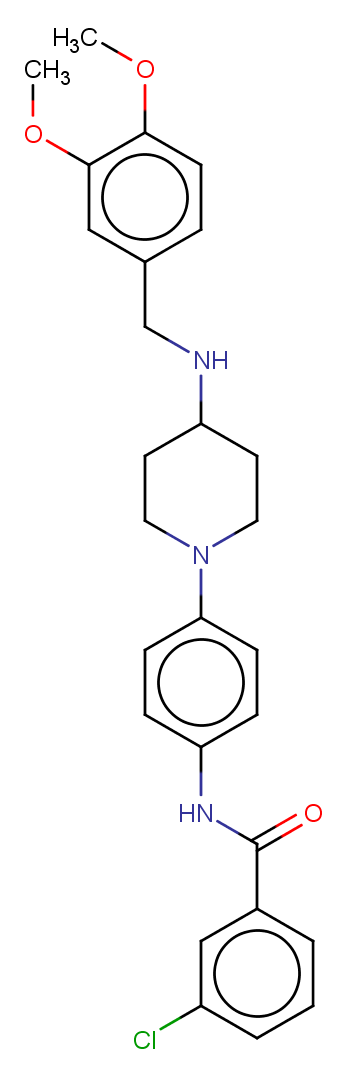

Supplement: RA-011-D1RA00914A-s1091 [file RA-011-D1RA00914A-s1091.png]

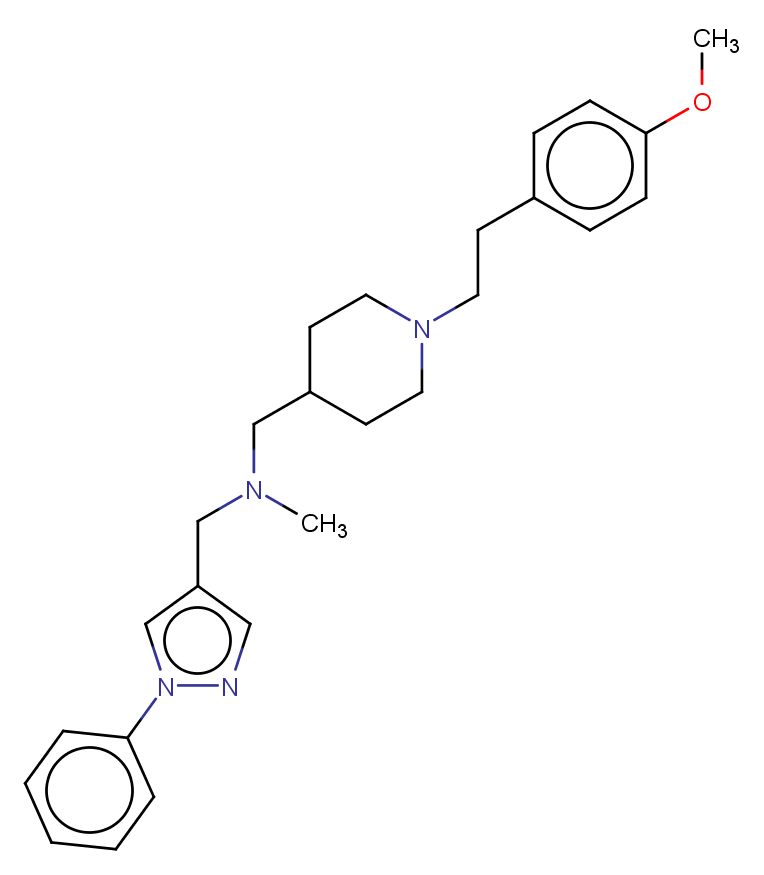

Supplement: RA-011-D1RA00914A-s1092 [file RA-011-D1RA00914A-s1092.png]

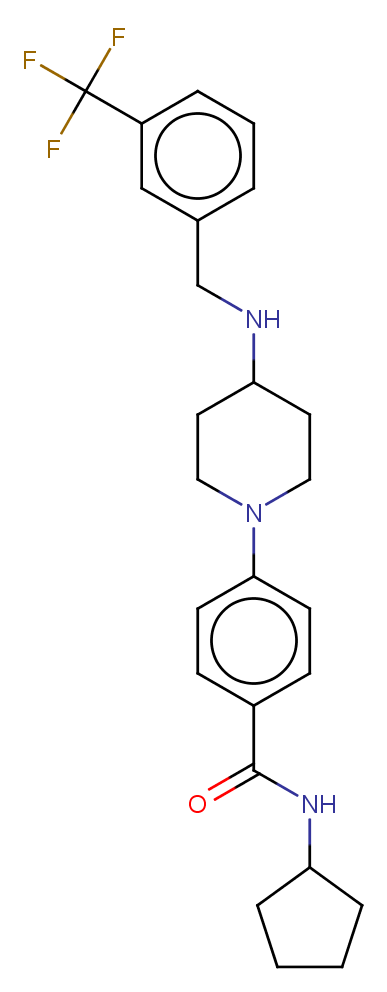

Supplement: RA-011-D1RA00914A-s1093 [file RA-011-D1RA00914A-s1093.png]

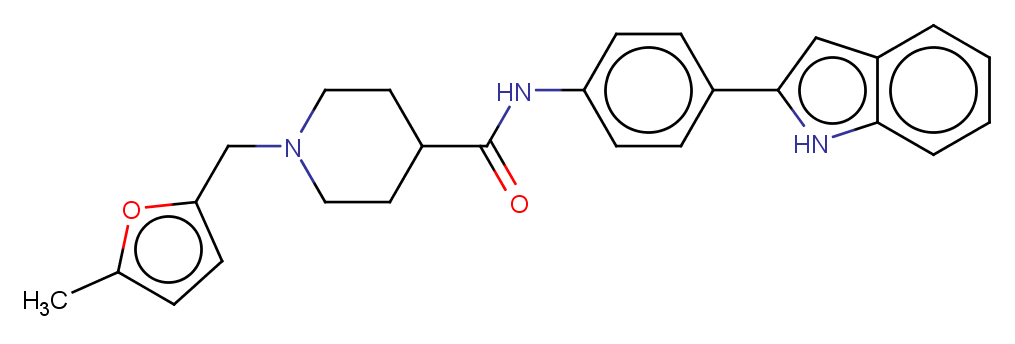

Supplement: RA-011-D1RA00914A-s1094 [file RA-011-D1RA00914A-s1094.png]

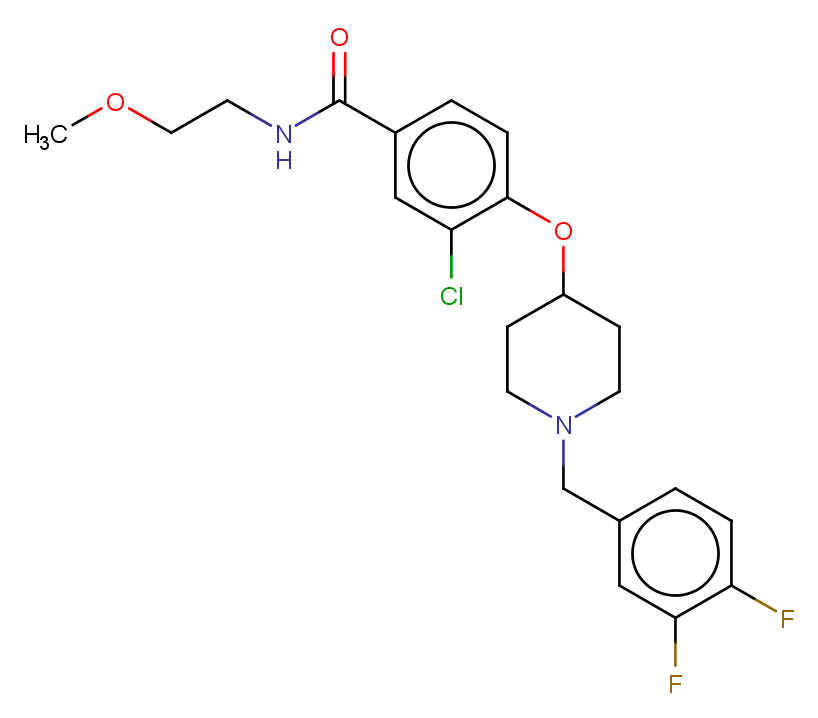

Supplement: RA-011-D1RA00914A-s1095 [file RA-011-D1RA00914A-s1095.png]

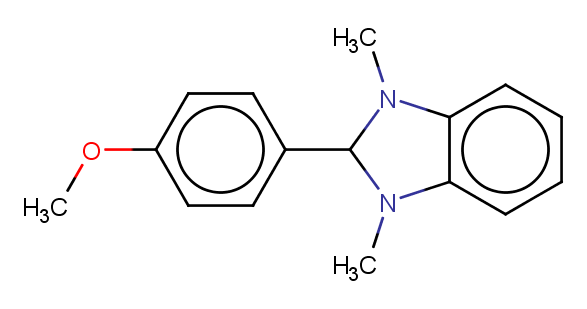

Supplement: RA-011-D1RA00914A-s1096 [file RA-011-D1RA00914A-s1096.png]

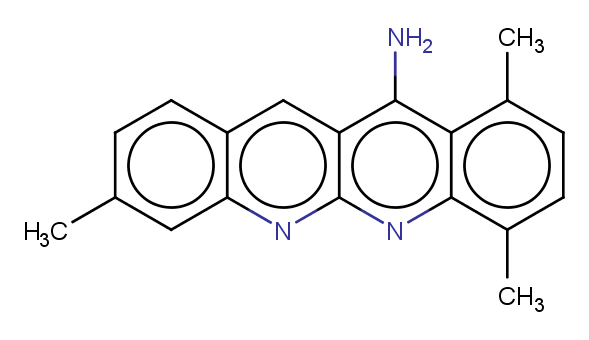

Supplement: RA-011-D1RA00914A-s1097 [file RA-011-D1RA00914A-s1097.png]

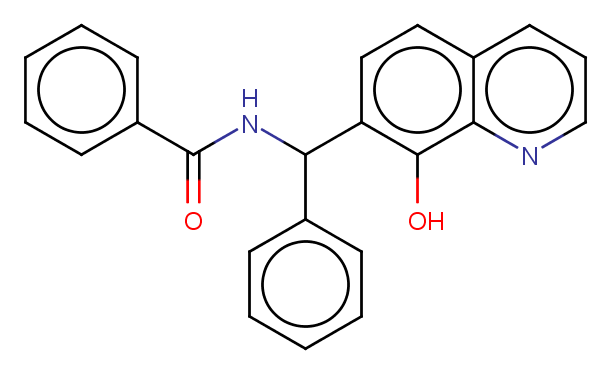

Supplement: RA-011-D1RA00914A-s1098 [file RA-011-D1RA00914A-s1098.png]

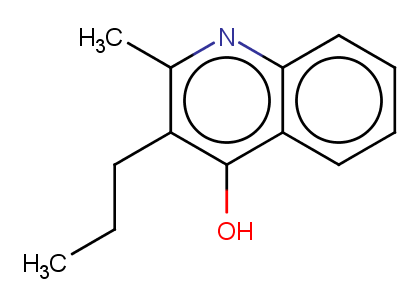

Supplement: RA-011-D1RA00914A-s1099 [file RA-011-D1RA00914A-s1099.png]

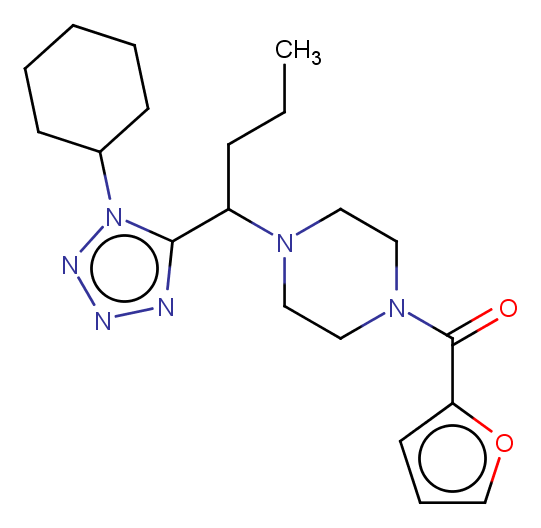

Supplement: RA-011-D1RA00914A-s1100 [file RA-011-D1RA00914A-s1100.png]

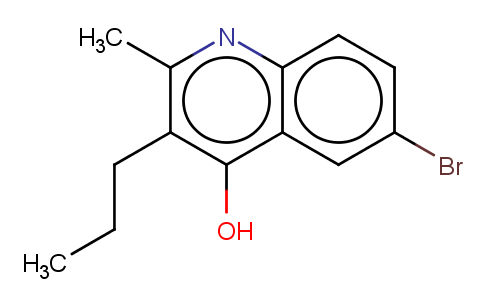

Supplement: RA-011-D1RA00914A-s1101 [file RA-011-D1RA00914A-s1101.png]

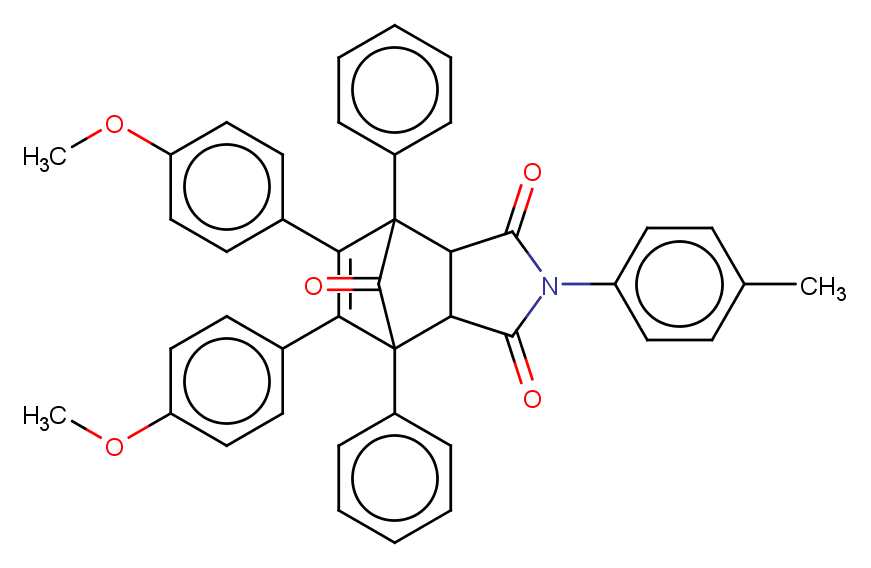

Supplement: RA-011-D1RA00914A-s1102 [file RA-011-D1RA00914A-s1102.png]

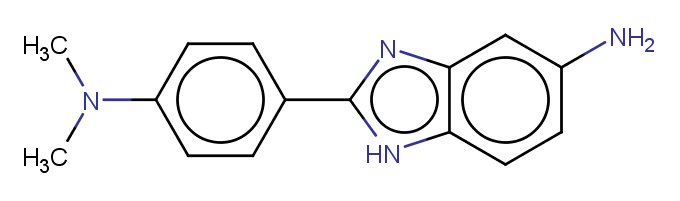

Supplement: RA-011-D1RA00914A-s1103 [file RA-011-D1RA00914A-s1103.png]

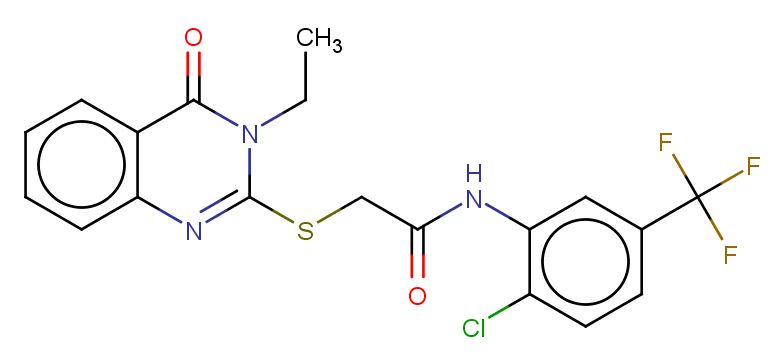

Supplement: RA-011-D1RA00914A-s1104 [file RA-011-D1RA00914A-s1104.png]

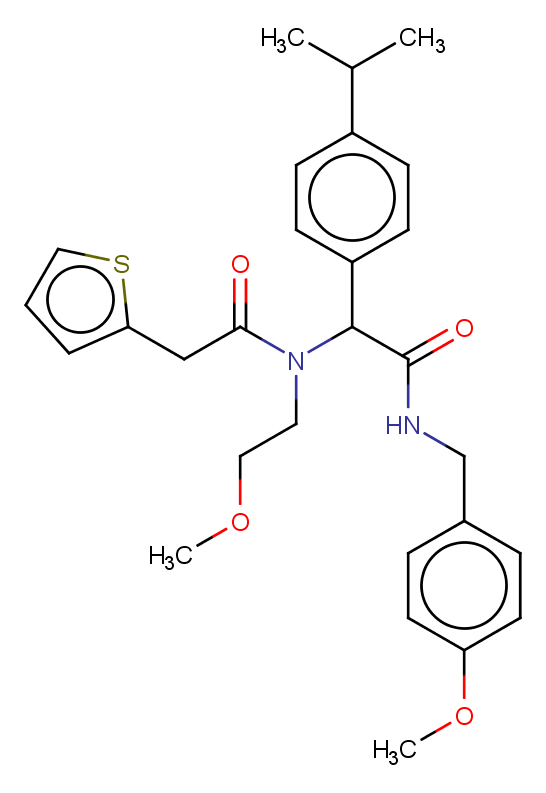

Supplement: RA-011-D1RA00914A-s1105 [file RA-011-D1RA00914A-s1105.png]

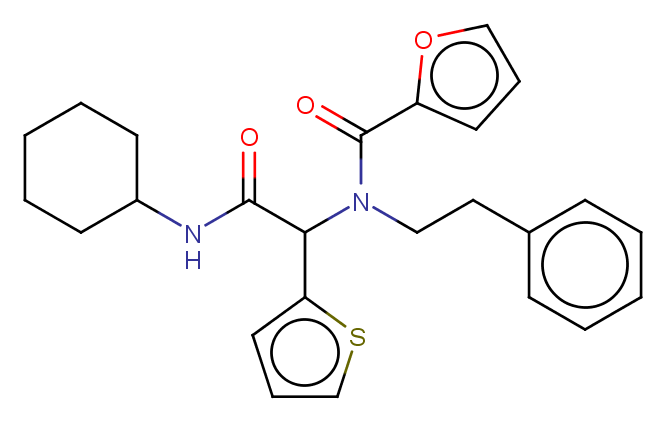

Supplement: RA-011-D1RA00914A-s1106 [file RA-011-D1RA00914A-s1106.png]

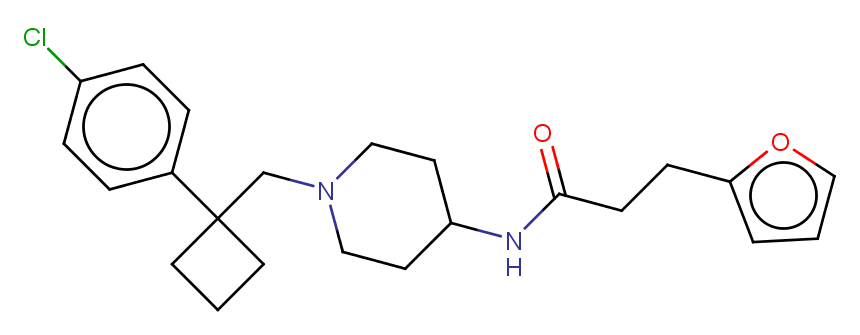

Supplement: RA-011-D1RA00914A-s1107 [file RA-011-D1RA00914A-s1107.png]

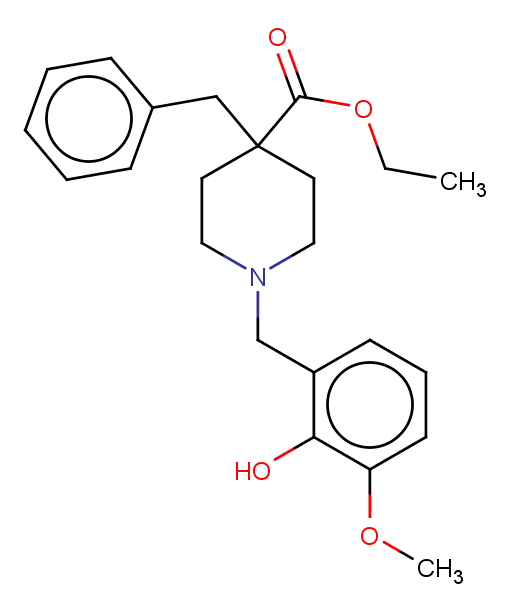

Supplement: RA-011-D1RA00914A-s1108 [file RA-011-D1RA00914A-s1108.png]

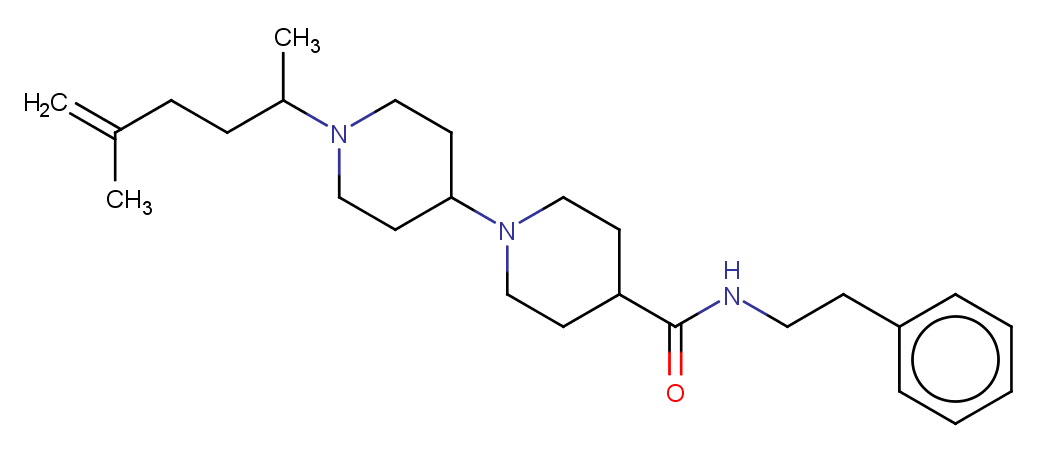

Supplement: RA-011-D1RA00914A-s1109 [file RA-011-D1RA00914A-s1109.png]

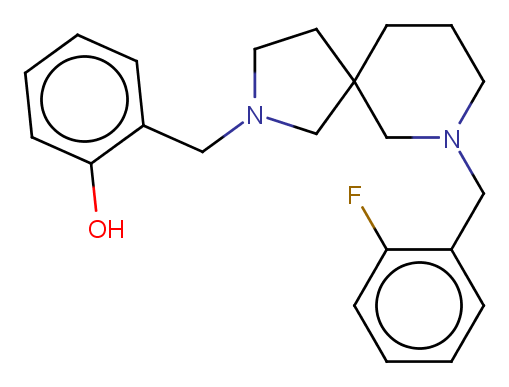

Supplement: RA-011-D1RA00914A-s1110 [file RA-011-D1RA00914A-s1110.png]

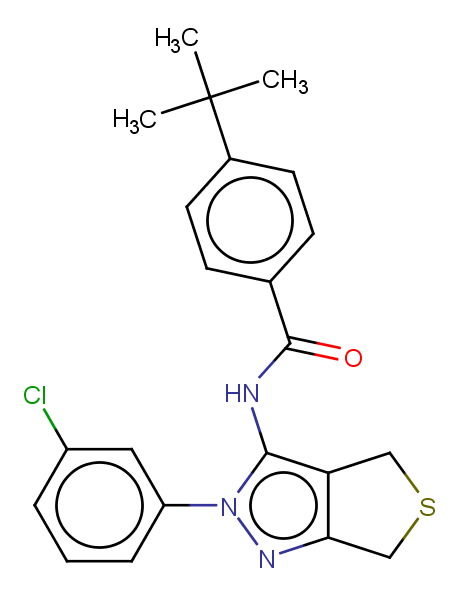

Supplement: RA-011-D1RA00914A-s1111 [file RA-011-D1RA00914A-s1111.png]

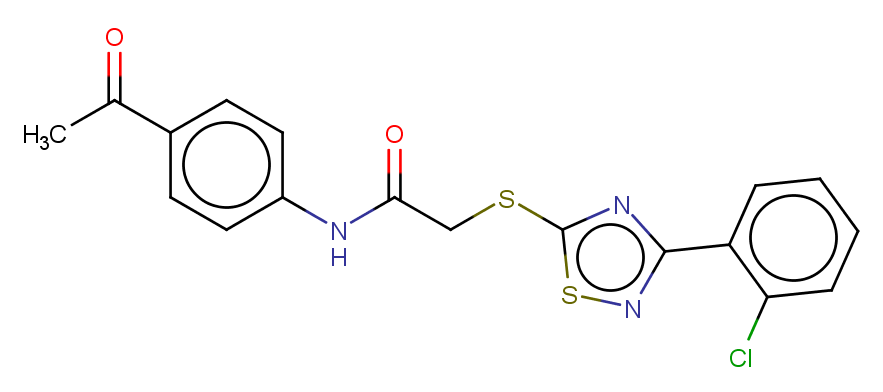

Supplement: RA-011-D1RA00914A-s1112 [file RA-011-D1RA00914A-s1112.png]

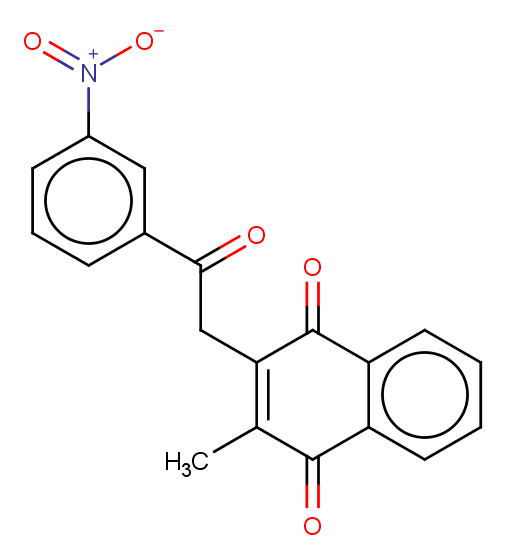

Supplement: RA-011-D1RA00914A-s1113 [file RA-011-D1RA00914A-s1113.png]

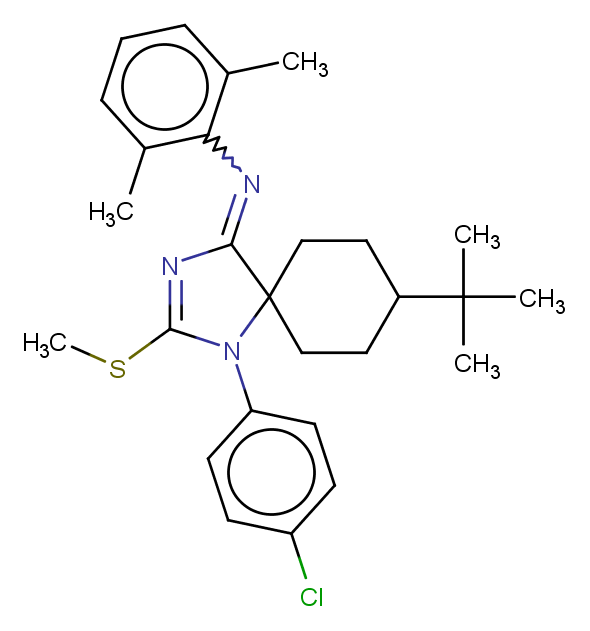

Supplement: RA-011-D1RA00914A-s1114 [file RA-011-D1RA00914A-s1114.png]

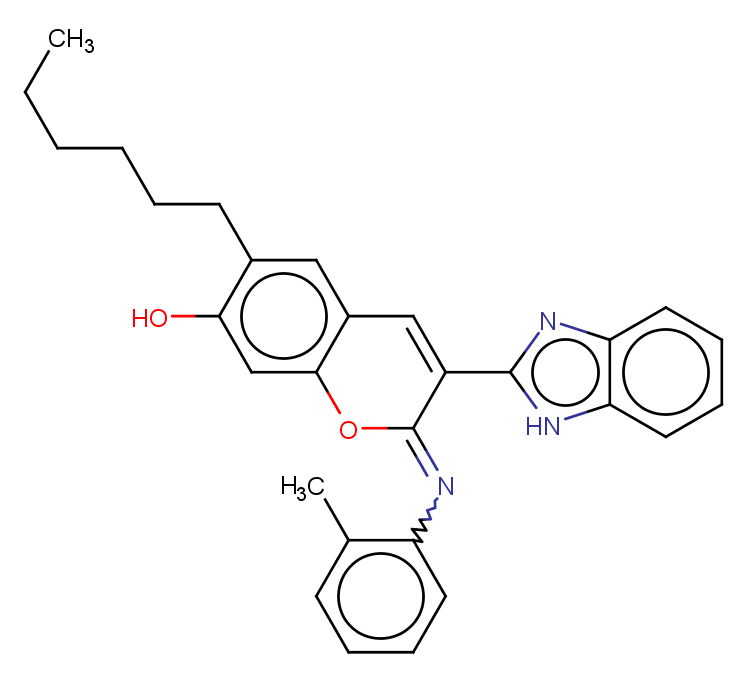

Supplement: RA-011-D1RA00914A-s1115 [file RA-011-D1RA00914A-s1115.png]

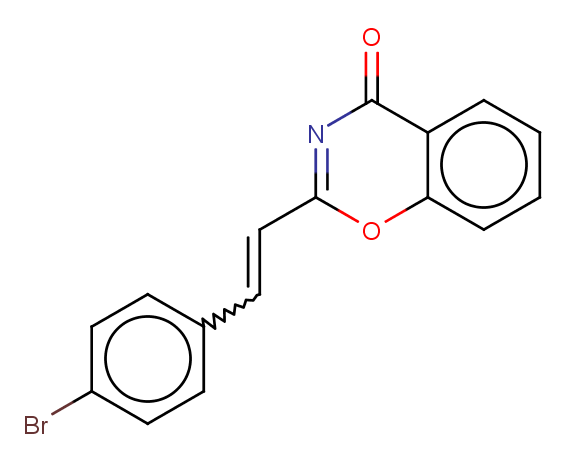

Supplement: RA-011-D1RA00914A-s1116 [file RA-011-D1RA00914A-s1116.png]

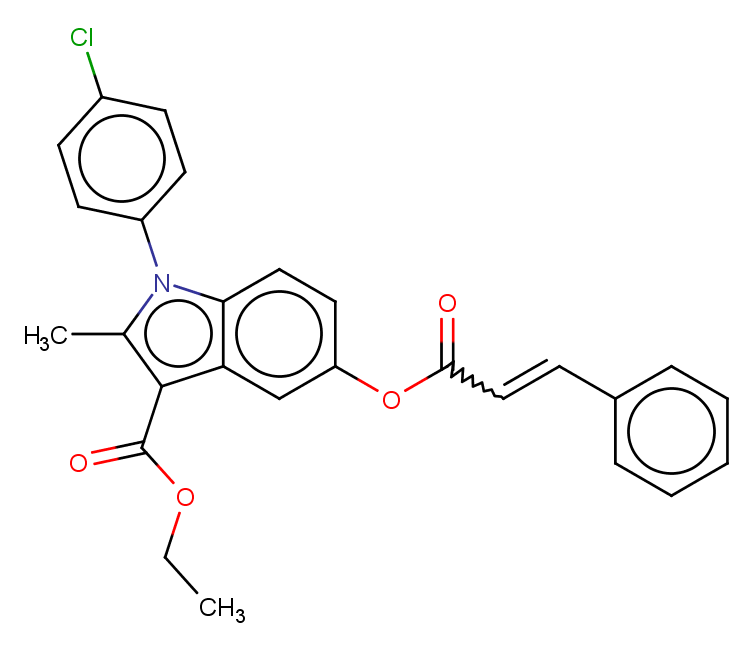

Supplement: RA-011-D1RA00914A-s1117 [file RA-011-D1RA00914A-s1117.png]

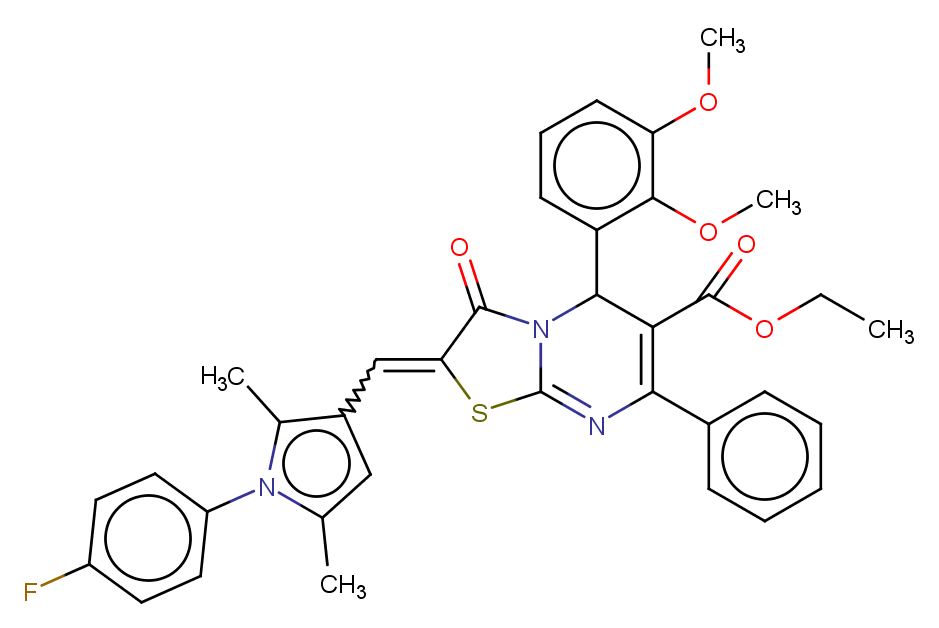

Supplement: RA-011-D1RA00914A-s1118 [file RA-011-D1RA00914A-s1118.png]

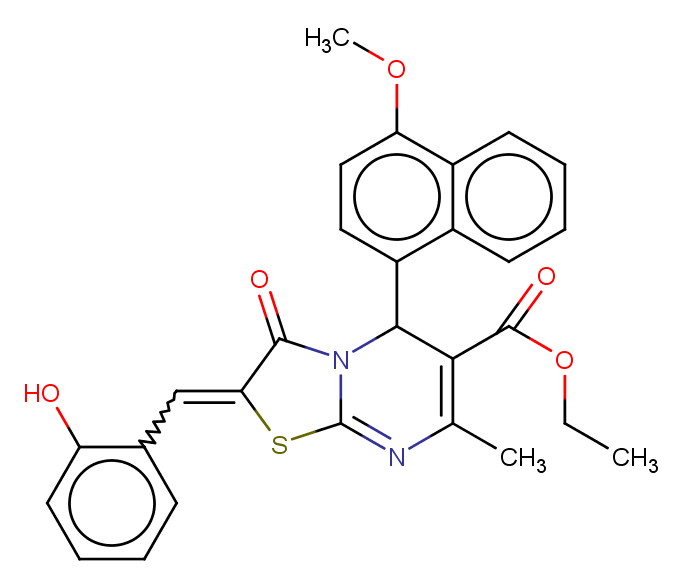

Supplement: RA-011-D1RA00914A-s1119 [file RA-011-D1RA00914A-s1119.png]

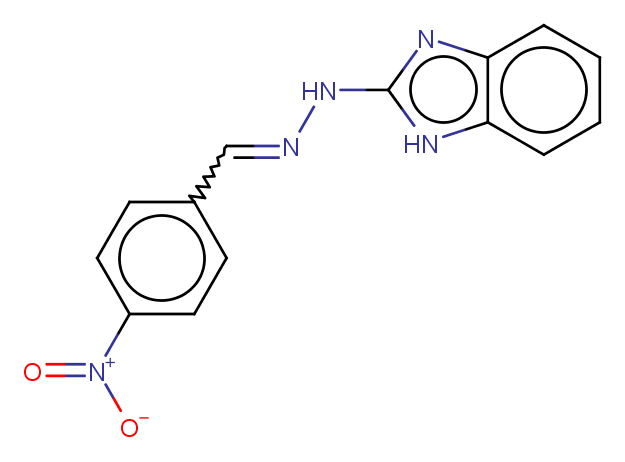

Supplement: RA-011-D1RA00914A-s1120 [file RA-011-D1RA00914A-s1120.png]

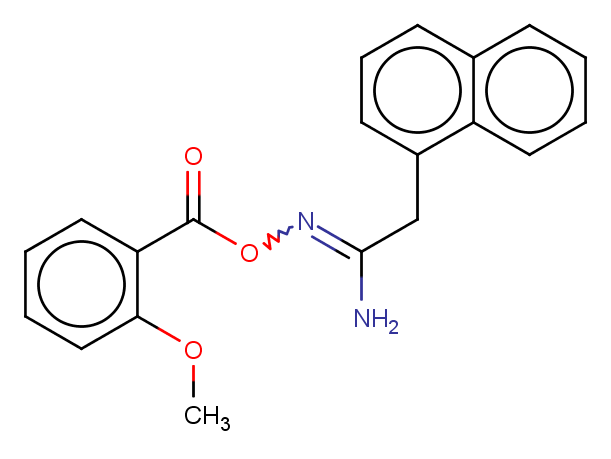

Supplement: RA-011-D1RA00914A-s1121 [file RA-011-D1RA00914A-s1121.png]

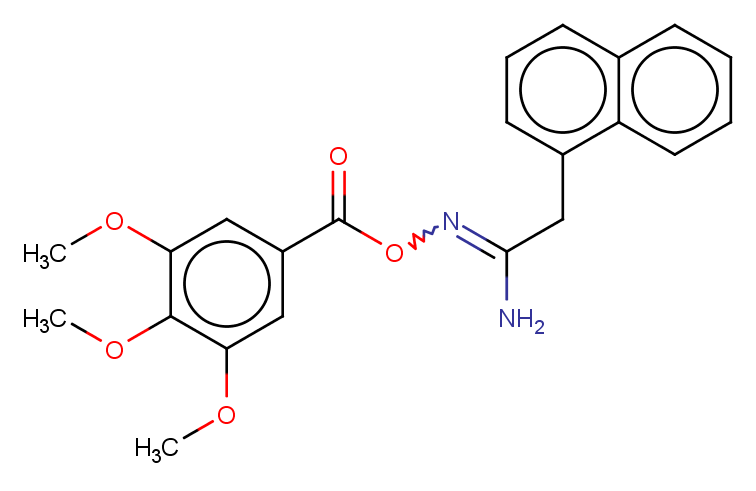

Supplement: RA-011-D1RA00914A-s1122 [file RA-011-D1RA00914A-s1122.png]

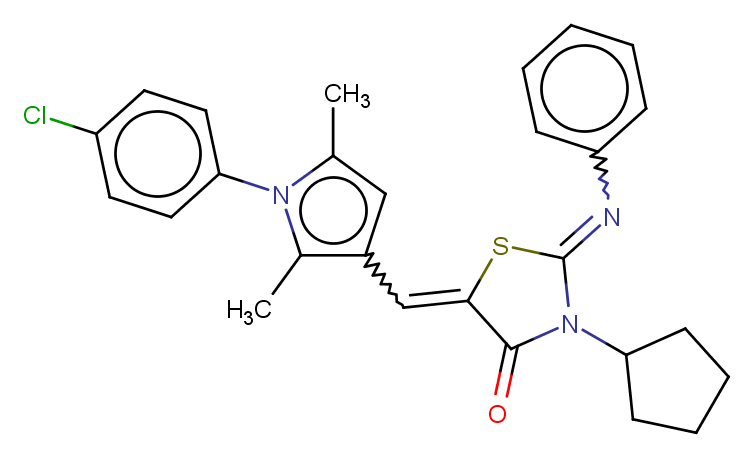

Supplement: RA-011-D1RA00914A-s1123 [file RA-011-D1RA00914A-s1123.png]

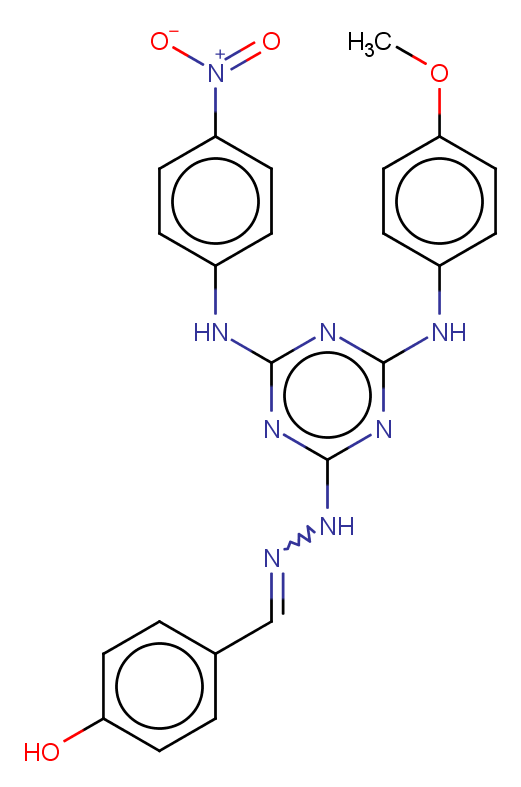

Supplement: RA-011-D1RA00914A-s1124 [file RA-011-D1RA00914A-s1124.png]

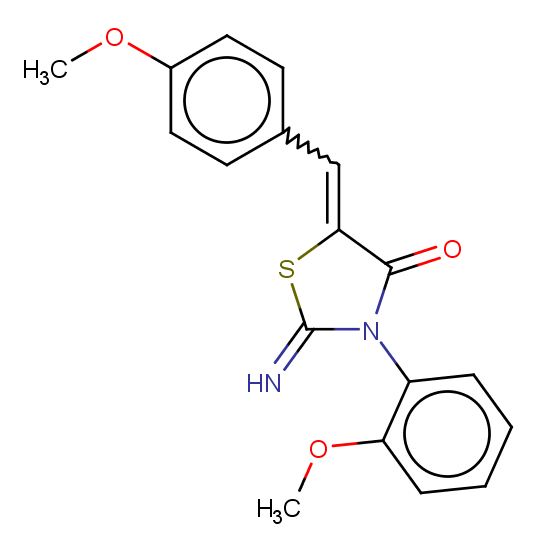

Supplement: RA-011-D1RA00914A-s1125 [file RA-011-D1RA00914A-s1125.png]

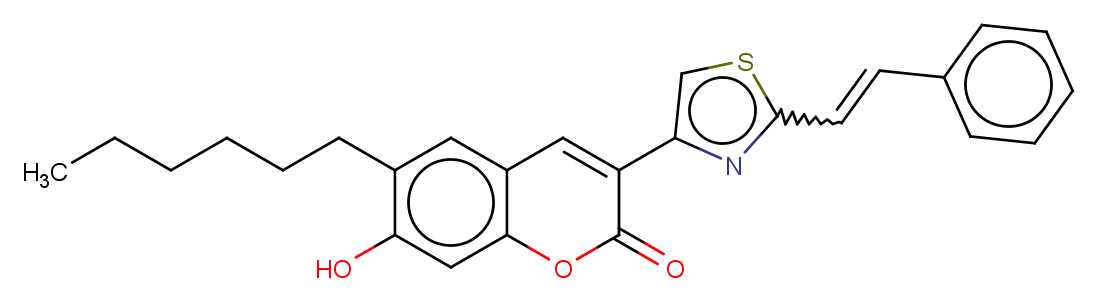

Supplement: RA-011-D1RA00914A-s1126 [file RA-011-D1RA00914A-s1126.png]

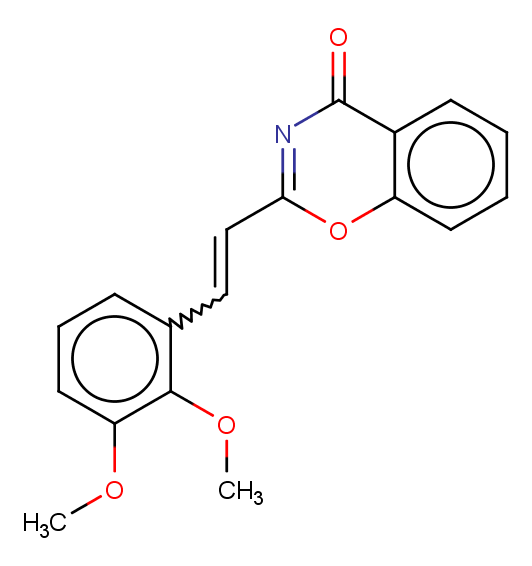

Supplement: RA-011-D1RA00914A-s1127 [file RA-011-D1RA00914A-s1127.png]

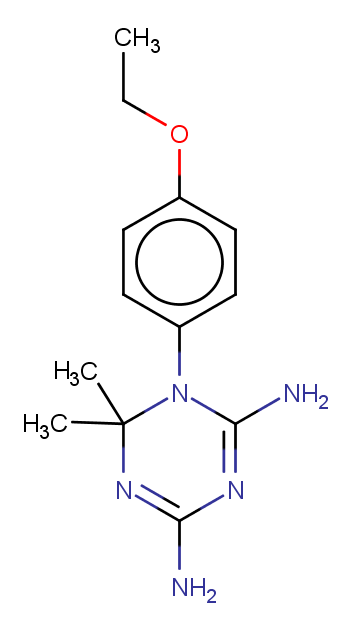

Supplement: RA-011-D1RA00914A-s1128 [file RA-011-D1RA00914A-s1128.png]

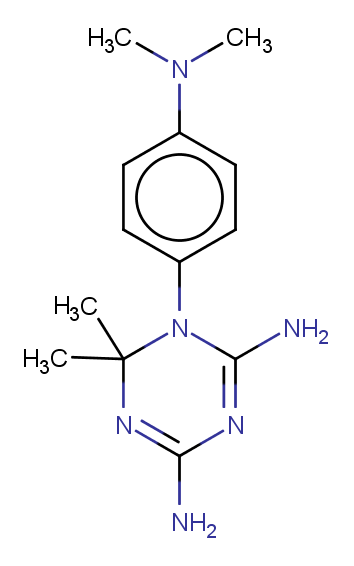

Supplement: RA-011-D1RA00914A-s1129 [file RA-011-D1RA00914A-s1129.png]

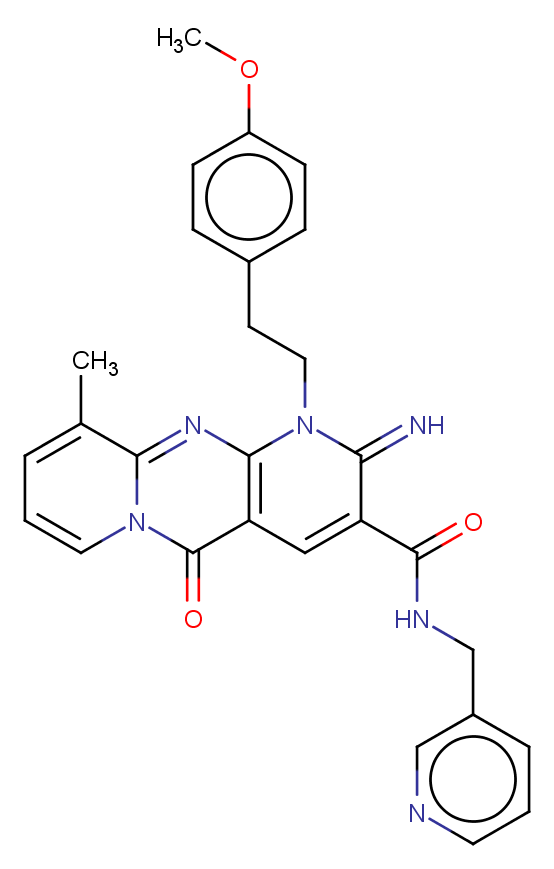

Supplement: RA-011-D1RA00914A-s1130 [file RA-011-D1RA00914A-s1130.png]

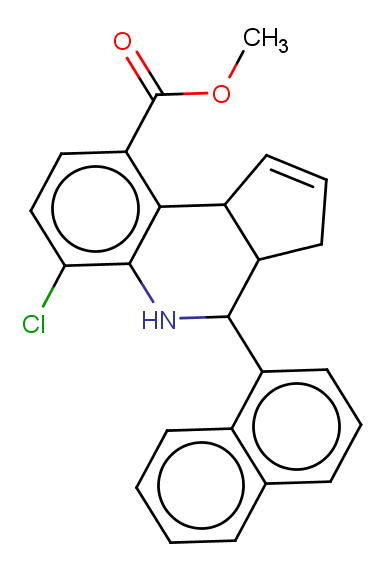

Supplement: RA-011-D1RA00914A-s1131 [file RA-011-D1RA00914A-s1131.png]

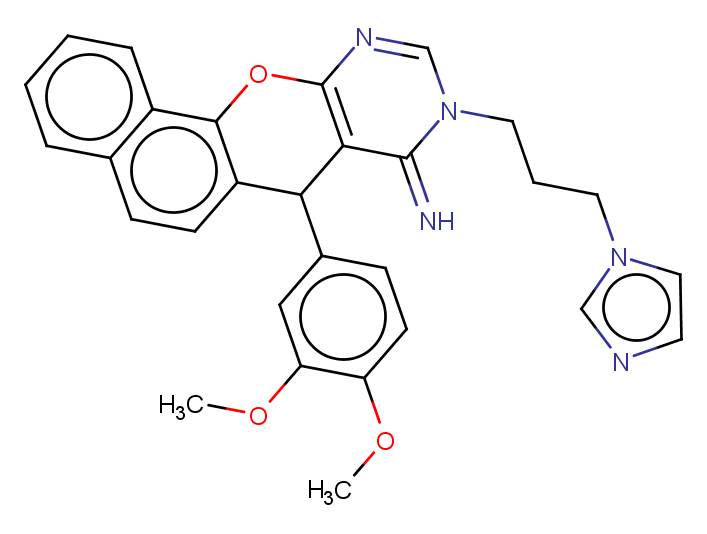

Supplement: RA-011-D1RA00914A-s1132 [file RA-011-D1RA00914A-s1132.png]

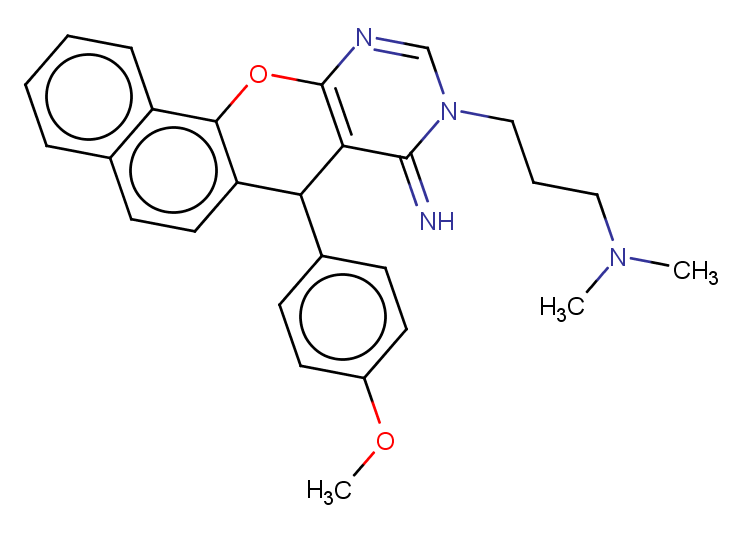

Supplement: RA-011-D1RA00914A-s1133 [file RA-011-D1RA00914A-s1133.png]

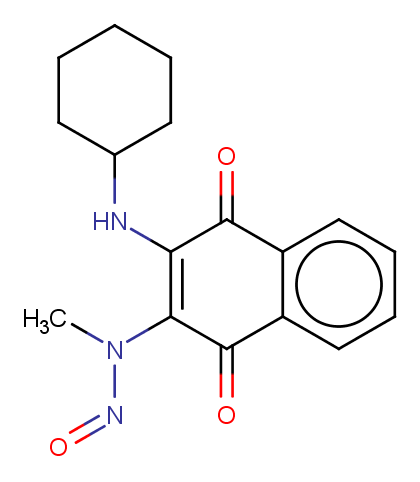

Supplement: RA-011-D1RA00914A-s1134 [file RA-011-D1RA00914A-s1134.png]

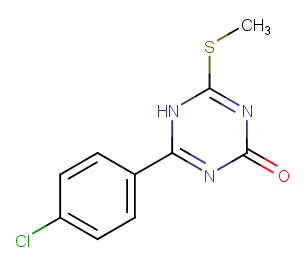

Supplement: RA-011-D1RA00914A-s1137 [file RA-011-D1RA00914A-s1137.png]

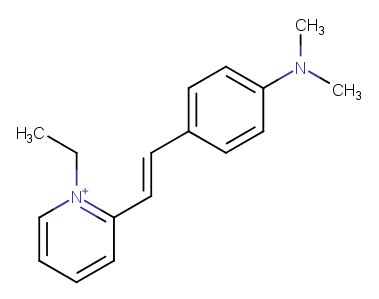

Supplement: RA-011-D1RA00914A-s1138 [file RA-011-D1RA00914A-s1138.png]

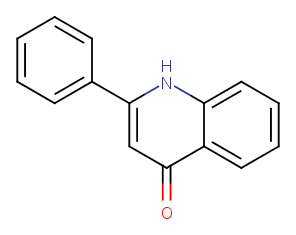

Supplement: RA-011-D1RA00914A-s1139 [file RA-011-D1RA00914A-s1139.png]

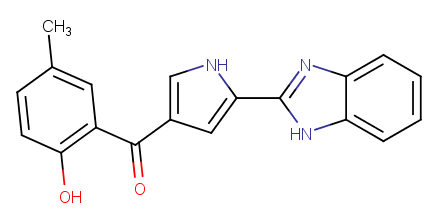

Supplement: RA-011-D1RA00914A-s1140 [file RA-011-D1RA00914A-s1140.png]

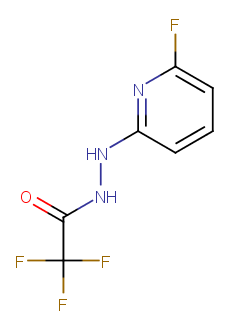

Supplement: RA-011-D1RA00914A-s1141 [file RA-011-D1RA00914A-s1141.png]

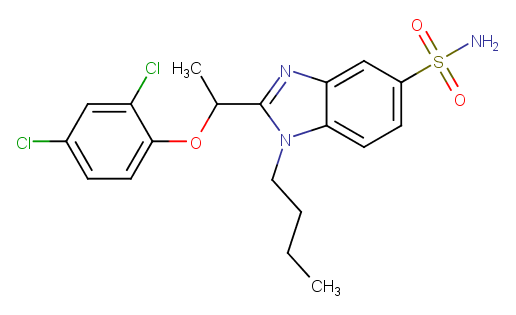

Supplement: RA-011-D1RA00914A-s1142 [file RA-011-D1RA00914A-s1142.png]

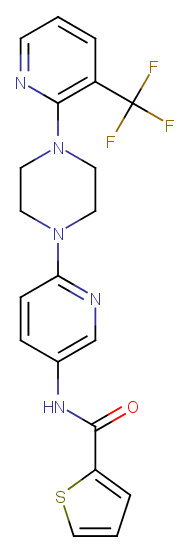

Supplement: RA-011-D1RA00914A-s1143 [file RA-011-D1RA00914A-s1143.png]

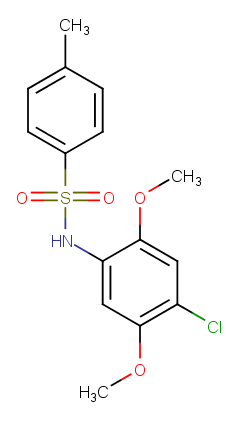

Supplement: RA-011-D1RA00914A-s1144 [file RA-011-D1RA00914A-s1144.png]

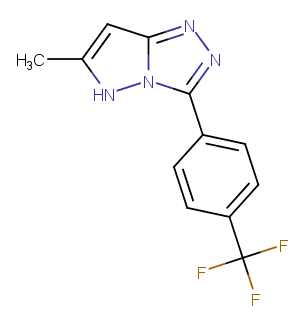

Supplement: RA-011-D1RA00914A-s1145 [file RA-011-D1RA00914A-s1145.png]

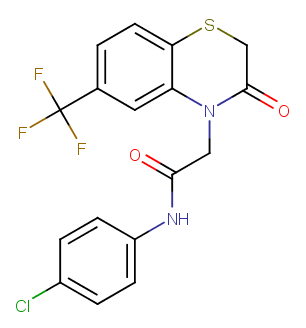

Supplement: RA-011-D1RA00914A-s1146 [file RA-011-D1RA00914A-s1146.png]

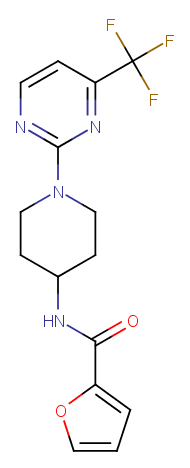

Supplement: RA-011-D1RA00914A-s1147 [file RA-011-D1RA00914A-s1147.png]

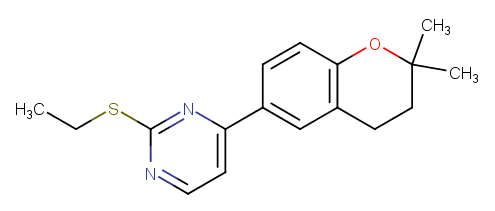

Supplement: RA-011-D1RA00914A-s1148 [file RA-011-D1RA00914A-s1148.png]

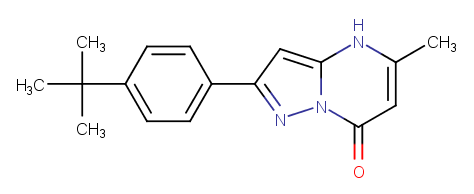

Supplement: RA-011-D1RA00914A-s1149 [file RA-011-D1RA00914A-s1149.png]

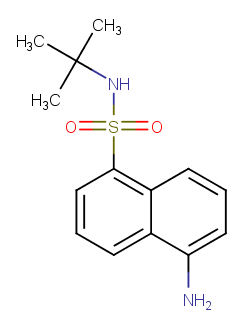

Supplement: RA-011-D1RA00914A-s1150 [file RA-011-D1RA00914A-s1150.png]

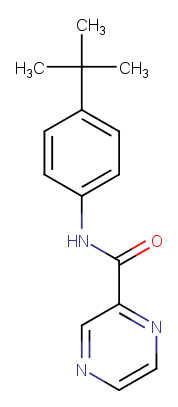

Supplement: RA-011-D1RA00914A-s1151 [file RA-011-D1RA00914A-s1151.png]

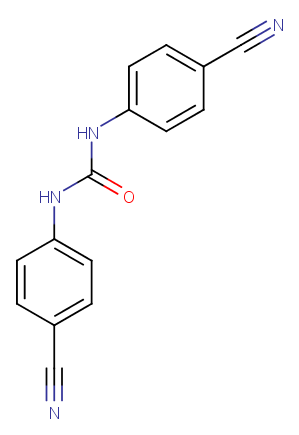

Supplement: RA-011-D1RA00914A-s1152 [file RA-011-D1RA00914A-s1152.png]

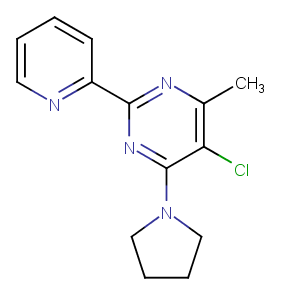

Supplement: RA-011-D1RA00914A-s1153 [file RA-011-D1RA00914A-s1153.png]

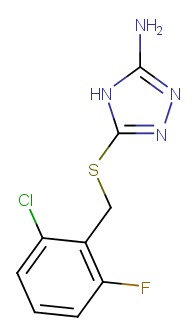

Supplement: RA-011-D1RA00914A-s1154 [file RA-011-D1RA00914A-s1154.png]

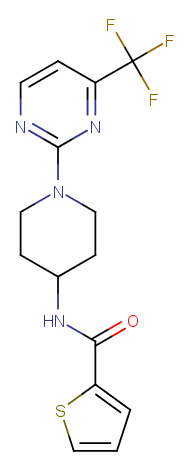

Supplement: RA-011-D1RA00914A-s1155 [file RA-011-D1RA00914A-s1155.png]

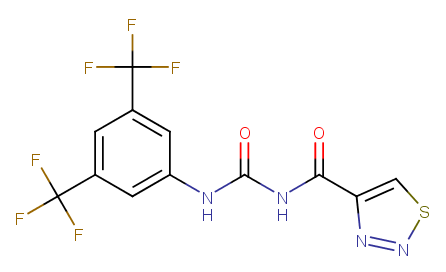

Supplement: RA-011-D1RA00914A-s1156 [file RA-011-D1RA00914A-s1156.png]

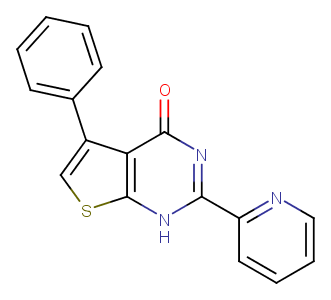

Supplement: RA-011-D1RA00914A-s1157 [file RA-011-D1RA00914A-s1157.png]

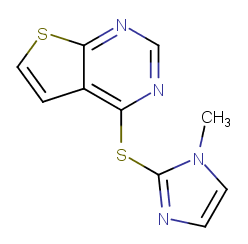

Supplement: RA-011-D1RA00914A-s1158 [file RA-011-D1RA00914A-s1158.png]

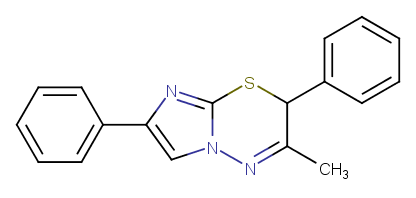

Supplement: RA-011-D1RA00914A-s1159 [file RA-011-D1RA00914A-s1159.png]

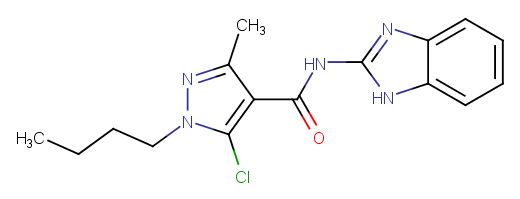

Supplement: RA-011-D1RA00914A-s1160 [file RA-011-D1RA00914A-s1160.png]

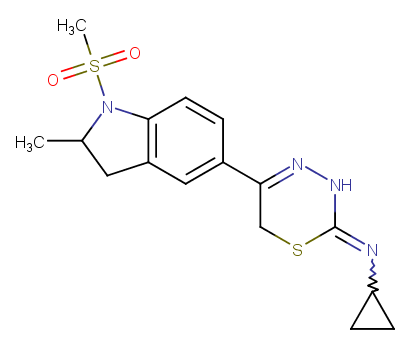

Supplement: RA-011-D1RA00914A-s1161 [file RA-011-D1RA00914A-s1161.png]

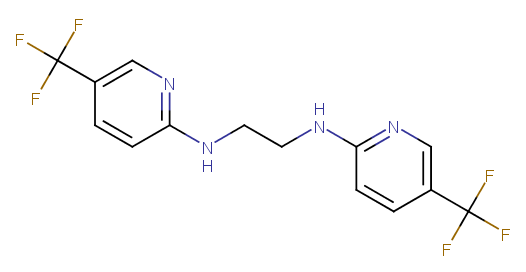

Supplement: RA-011-D1RA00914A-s1162 [file RA-011-D1RA00914A-s1162.png]

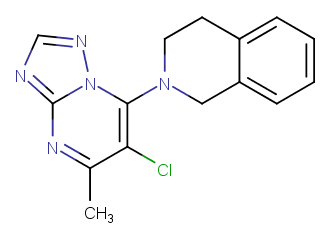

Supplement: RA-011-D1RA00914A-s1163 [file RA-011-D1RA00914A-s1163.png]

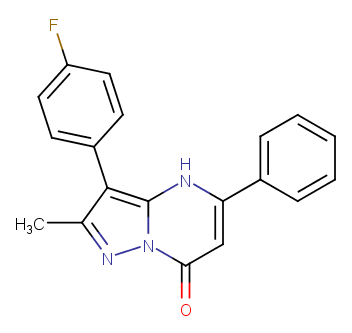

Supplement: RA-011-D1RA00914A-s1164 [file RA-011-D1RA00914A-s1164.png]

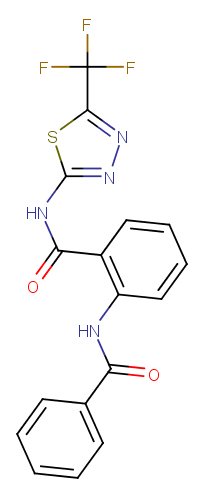

Supplement: RA-011-D1RA00914A-s1165 [file RA-011-D1RA00914A-s1165.png]

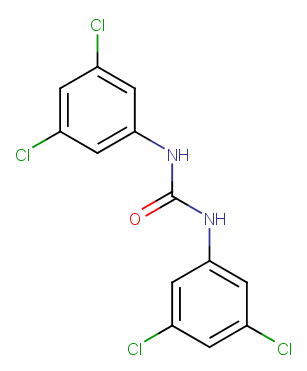

Supplement: RA-011-D1RA00914A-s1166 [file RA-011-D1RA00914A-s1166.png]

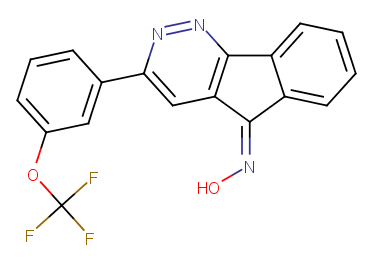

Supplement: RA-011-D1RA00914A-s1167 [file RA-011-D1RA00914A-s1167.png]

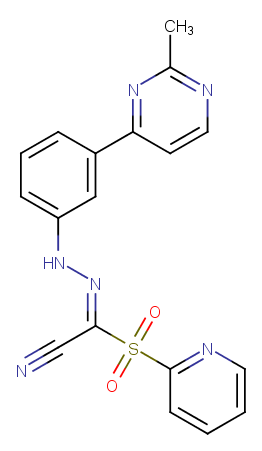

Supplement: RA-011-D1RA00914A-s1168 [file RA-011-D1RA00914A-s1168.png]

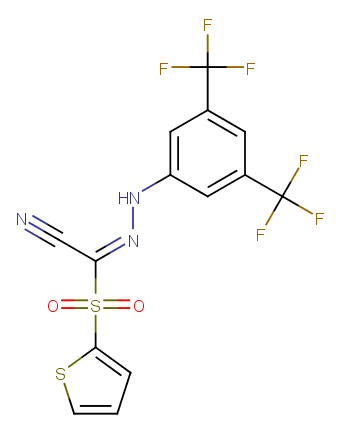

Supplement: RA-011-D1RA00914A-s1169 [file RA-011-D1RA00914A-s1169.png]

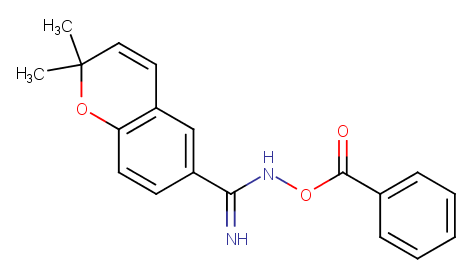

Supplement: RA-011-D1RA00914A-s1170 [file RA-011-D1RA00914A-s1170.png]

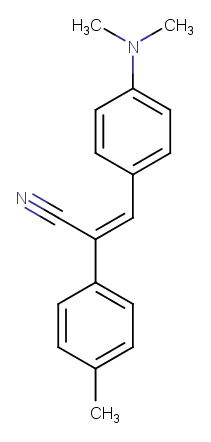

Supplement: RA-011-D1RA00914A-s1171 [file RA-011-D1RA00914A-s1171.png]

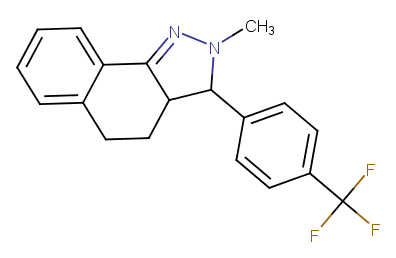

Supplement: RA-011-D1RA00914A-s1172 [file RA-011-D1RA00914A-s1172.png]

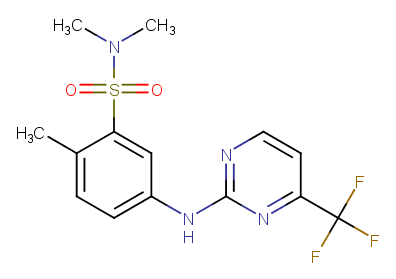

Supplement: RA-011-D1RA00914A-s1173 [file RA-011-D1RA00914A-s1173.png]
