# Supplementary figures and images for: Improved herbicide discovery using physico-chemical rules refined by antimalarial library screening (part 9 of 14)
Source: RSC Adv. 2021 Feb 23;11(15):8459–67. doi: 10.1039/d1ra00914a (PMC8695207; doi:10.1039/d1ra00914a)

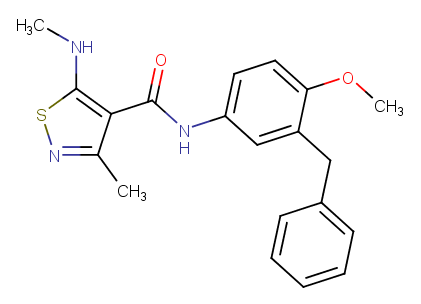

Supplement: RA-011-D1RA00914A-s1174 [file RA-011-D1RA00914A-s1174.png]

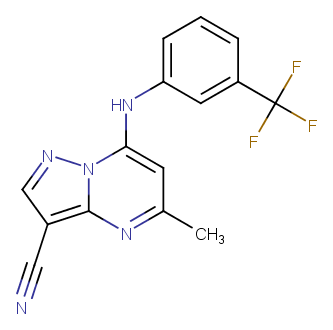

Supplement: RA-011-D1RA00914A-s1175 [file RA-011-D1RA00914A-s1175.png]

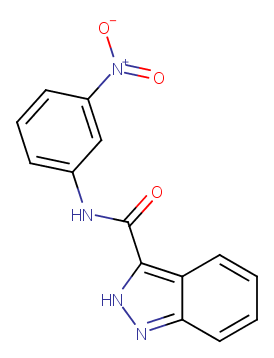

Supplement: RA-011-D1RA00914A-s1176 [file RA-011-D1RA00914A-s1176.png]

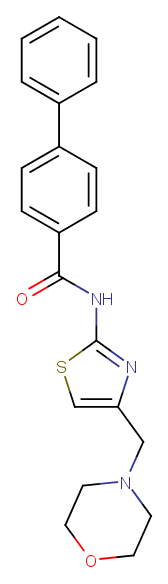

Supplement: RA-011-D1RA00914A-s1177 [file RA-011-D1RA00914A-s1177.png]

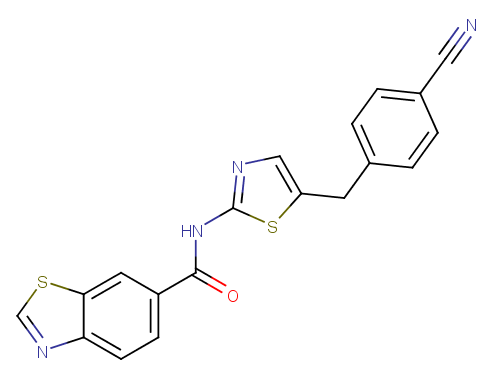

Supplement: RA-011-D1RA00914A-s1178 [file RA-011-D1RA00914A-s1178.png]

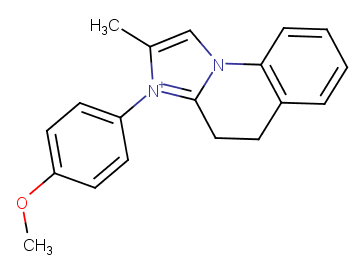

Supplement: RA-011-D1RA00914A-s1179 [file RA-011-D1RA00914A-s1179.png]

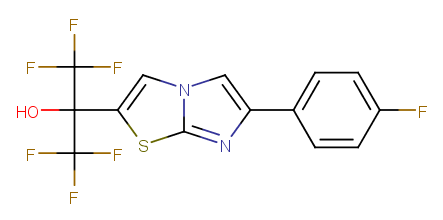

Supplement: RA-011-D1RA00914A-s1180 [file RA-011-D1RA00914A-s1180.png]

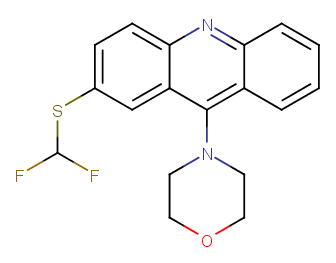

Supplement: RA-011-D1RA00914A-s1181 [file RA-011-D1RA00914A-s1181.png]

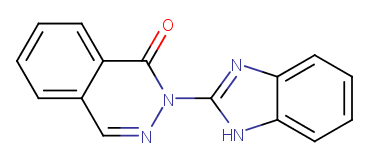

Supplement: RA-011-D1RA00914A-s1182 [file RA-011-D1RA00914A-s1182.png]

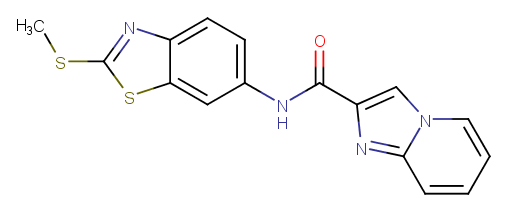

Supplement: RA-011-D1RA00914A-s1183 [file RA-011-D1RA00914A-s1183.png]

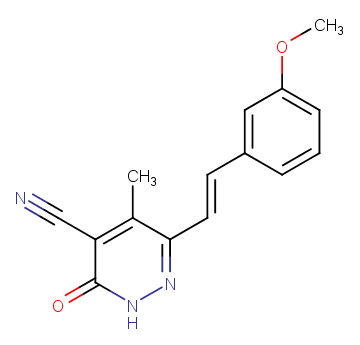

Supplement: RA-011-D1RA00914A-s1184 [file RA-011-D1RA00914A-s1184.png]

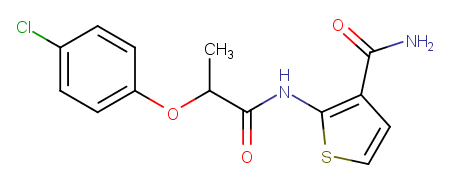

Supplement: RA-011-D1RA00914A-s1185 [file RA-011-D1RA00914A-s1185.png]

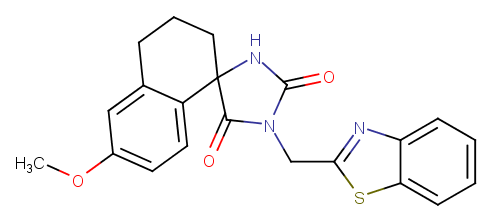

Supplement: RA-011-D1RA00914A-s1186 [file RA-011-D1RA00914A-s1186.png]

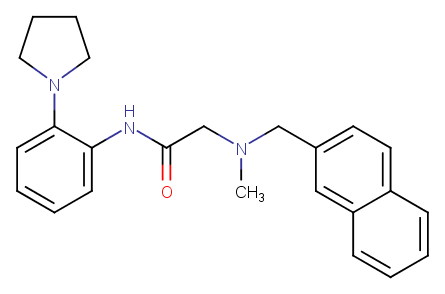

Supplement: RA-011-D1RA00914A-s1187 [file RA-011-D1RA00914A-s1187.png]

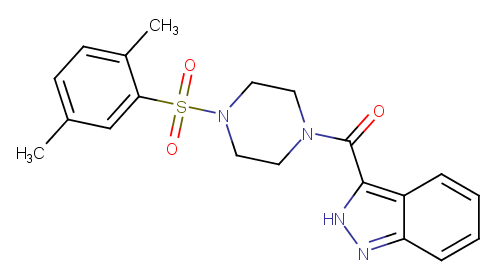

Supplement: RA-011-D1RA00914A-s1188 [file RA-011-D1RA00914A-s1188.png]

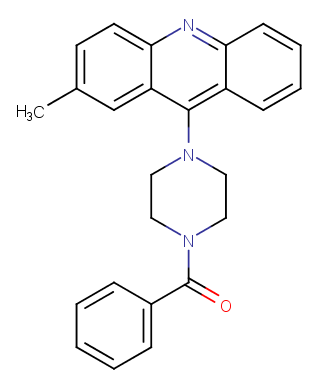

Supplement: RA-011-D1RA00914A-s1189 [file RA-011-D1RA00914A-s1189.png]

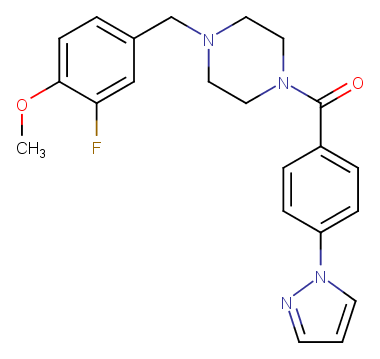

Supplement: RA-011-D1RA00914A-s1190 [file RA-011-D1RA00914A-s1190.png]

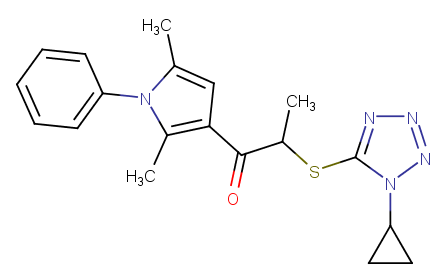

Supplement: RA-011-D1RA00914A-s1191 [file RA-011-D1RA00914A-s1191.png]

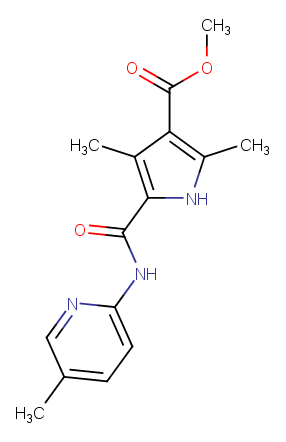

Supplement: RA-011-D1RA00914A-s1192 [file RA-011-D1RA00914A-s1192.png]

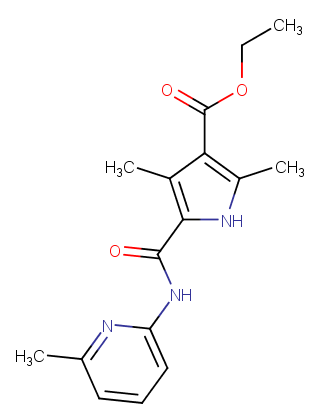

Supplement: RA-011-D1RA00914A-s1193 [file RA-011-D1RA00914A-s1193.png]

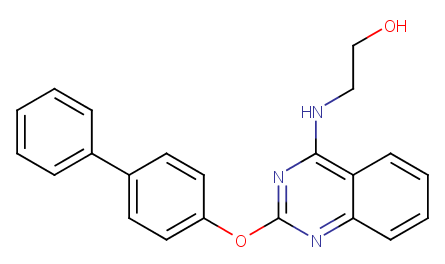

Supplement: RA-011-D1RA00914A-s1194 [file RA-011-D1RA00914A-s1194.png]

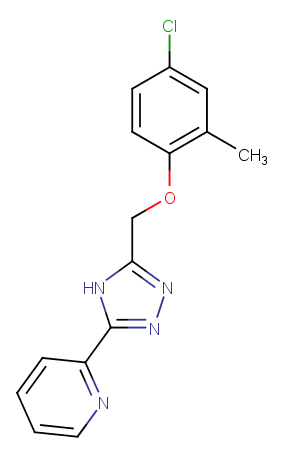

Supplement: RA-011-D1RA00914A-s1195 [file RA-011-D1RA00914A-s1195.png]

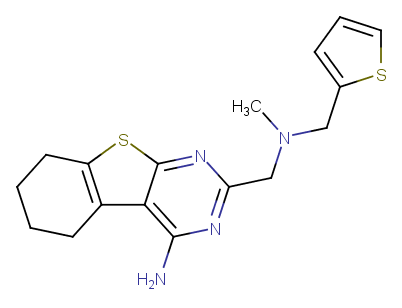

Supplement: RA-011-D1RA00914A-s1196 [file RA-011-D1RA00914A-s1196.png]

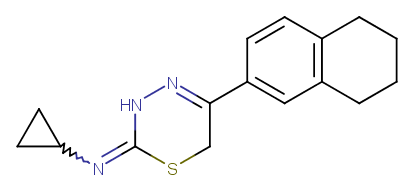

Supplement: RA-011-D1RA00914A-s1197 [file RA-011-D1RA00914A-s1197.png]

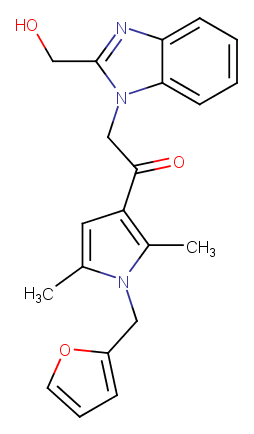

Supplement: RA-011-D1RA00914A-s1198 [file RA-011-D1RA00914A-s1198.png]

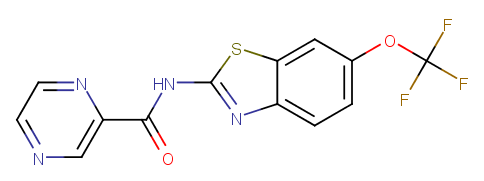

Supplement: RA-011-D1RA00914A-s1199 [file RA-011-D1RA00914A-s1199.png]

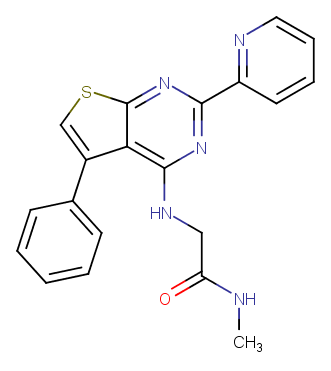

Supplement: RA-011-D1RA00914A-s1200 [file RA-011-D1RA00914A-s1200.png]

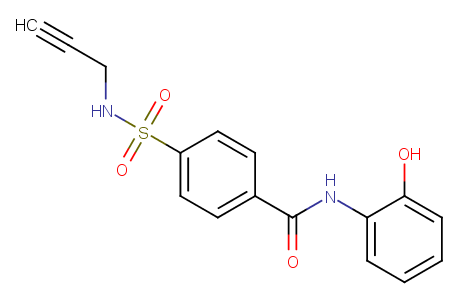

Supplement: RA-011-D1RA00914A-s1201 [file RA-011-D1RA00914A-s1201.png]

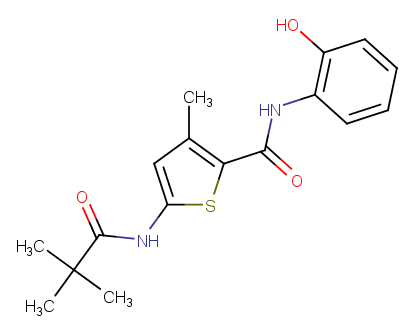

Supplement: RA-011-D1RA00914A-s1202 [file RA-011-D1RA00914A-s1202.png]

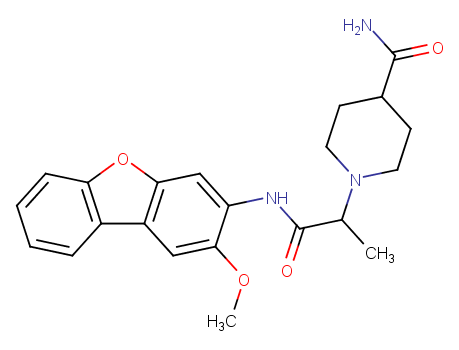

Supplement: RA-011-D1RA00914A-s1203 [file RA-011-D1RA00914A-s1203.png]

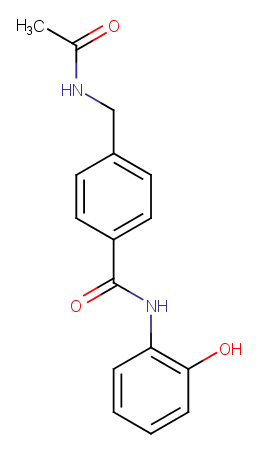

Supplement: RA-011-D1RA00914A-s1204 [file RA-011-D1RA00914A-s1204.png]

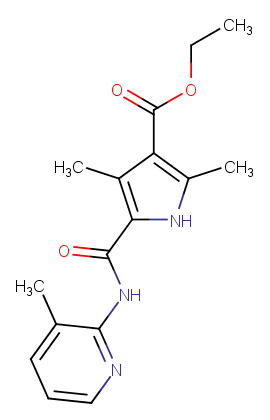

Supplement: RA-011-D1RA00914A-s1205 [file RA-011-D1RA00914A-s1205.png]

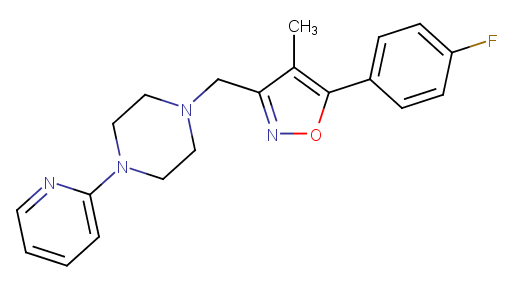

Supplement: RA-011-D1RA00914A-s1206 [file RA-011-D1RA00914A-s1206.png]

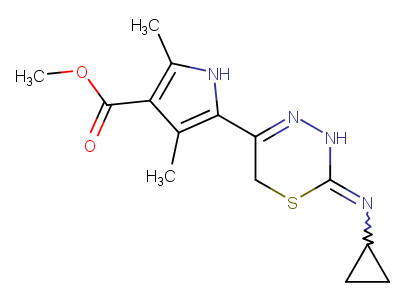

Supplement: RA-011-D1RA00914A-s1207 [file RA-011-D1RA00914A-s1207.png]

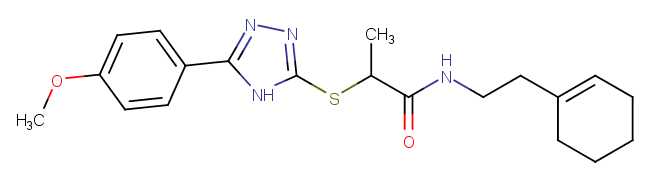

Supplement: RA-011-D1RA00914A-s1208 [file RA-011-D1RA00914A-s1208.png]

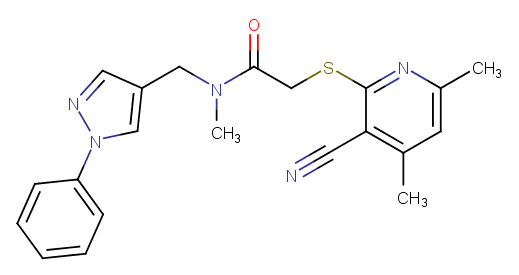

Supplement: RA-011-D1RA00914A-s1209 [file RA-011-D1RA00914A-s1209.png]

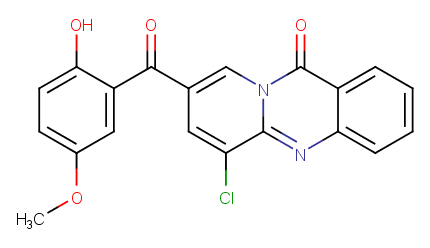

Supplement: RA-011-D1RA00914A-s1210 [file RA-011-D1RA00914A-s1210.png]

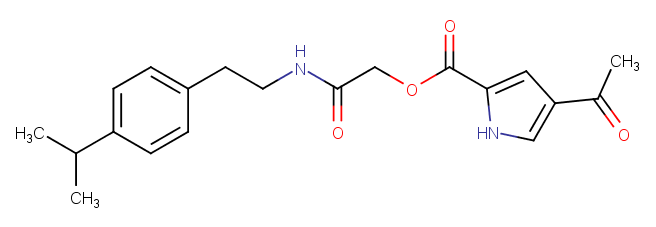

Supplement: RA-011-D1RA00914A-s1211 [file RA-011-D1RA00914A-s1211.png]

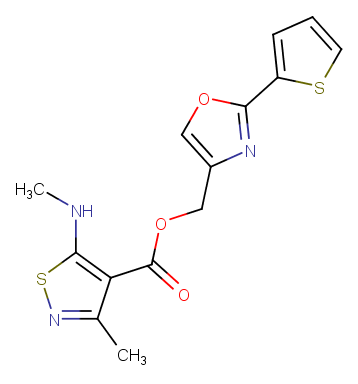

Supplement: RA-011-D1RA00914A-s1212 [file RA-011-D1RA00914A-s1212.png]

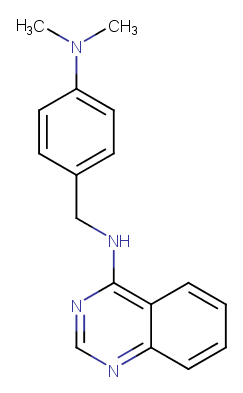

Supplement: RA-011-D1RA00914A-s1213 [file RA-011-D1RA00914A-s1213.png]

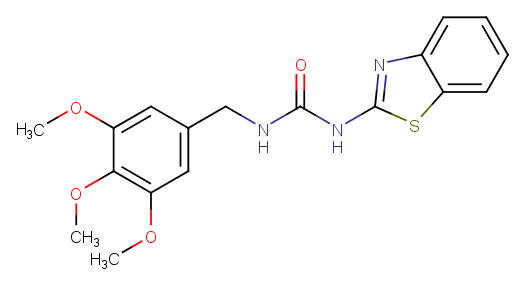

Supplement: RA-011-D1RA00914A-s1214 [file RA-011-D1RA00914A-s1214.png]

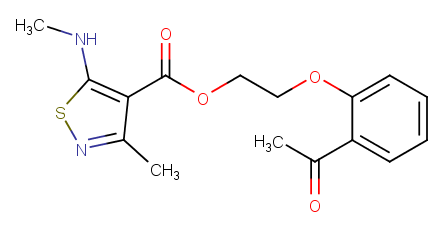

Supplement: RA-011-D1RA00914A-s1215 [file RA-011-D1RA00914A-s1215.png]

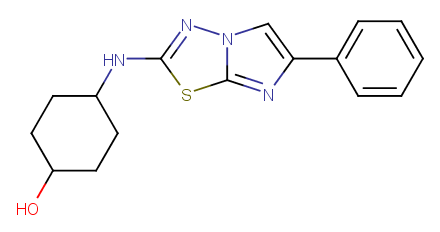

Supplement: RA-011-D1RA00914A-s1216 [file RA-011-D1RA00914A-s1216.png]

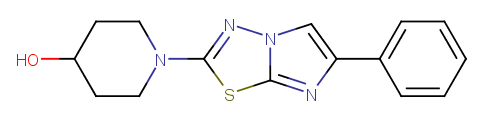

Supplement: RA-011-D1RA00914A-s1217 [file RA-011-D1RA00914A-s1217.png]

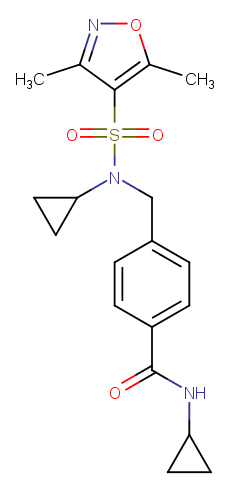

Supplement: RA-011-D1RA00914A-s1218 [file RA-011-D1RA00914A-s1218.png]

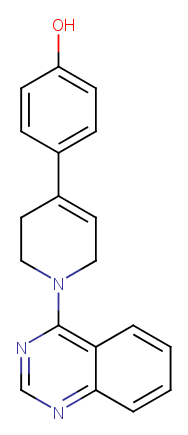

Supplement: RA-011-D1RA00914A-s1219 [file RA-011-D1RA00914A-s1219.png]

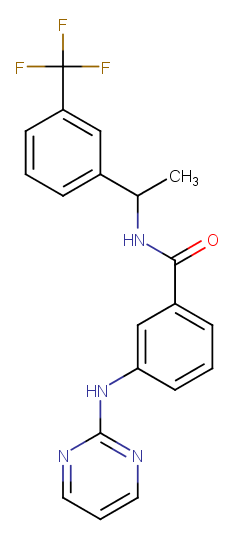

Supplement: RA-011-D1RA00914A-s1220 [file RA-011-D1RA00914A-s1220.png]

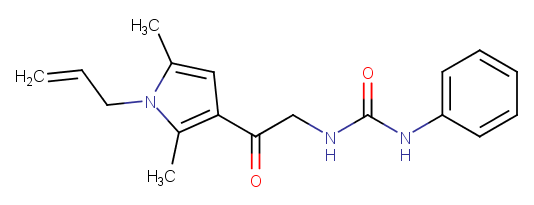

Supplement: RA-011-D1RA00914A-s1221 [file RA-011-D1RA00914A-s1221.png]

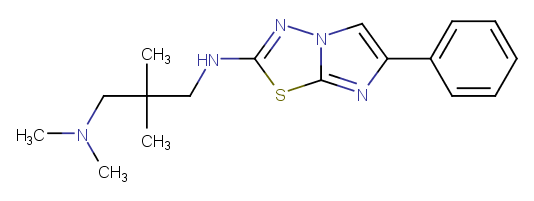

Supplement: RA-011-D1RA00914A-s1222 [file RA-011-D1RA00914A-s1222.png]

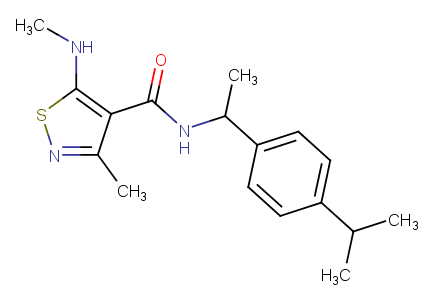

Supplement: RA-011-D1RA00914A-s1223 [file RA-011-D1RA00914A-s1223.png]

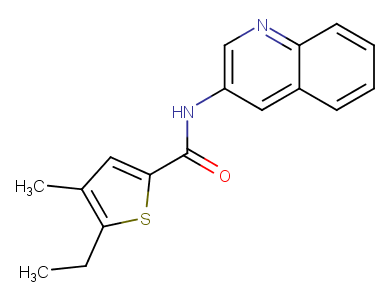

Supplement: RA-011-D1RA00914A-s1224 [file RA-011-D1RA00914A-s1224.png]

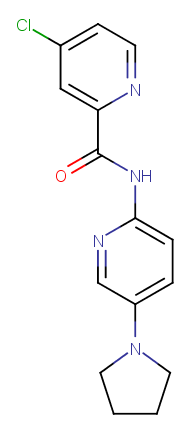

Supplement: RA-011-D1RA00914A-s1225 [file RA-011-D1RA00914A-s1225.png]

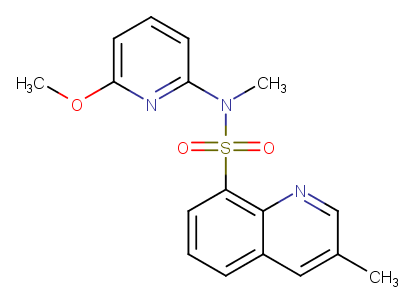

Supplement: RA-011-D1RA00914A-s1226 [file RA-011-D1RA00914A-s1226.png]

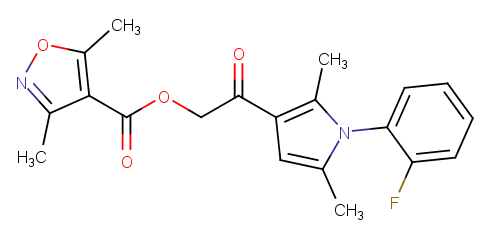

Supplement: RA-011-D1RA00914A-s1227 [file RA-011-D1RA00914A-s1227.png]

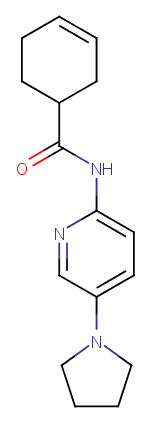

Supplement: RA-011-D1RA00914A-s1228 [file RA-011-D1RA00914A-s1228.png]

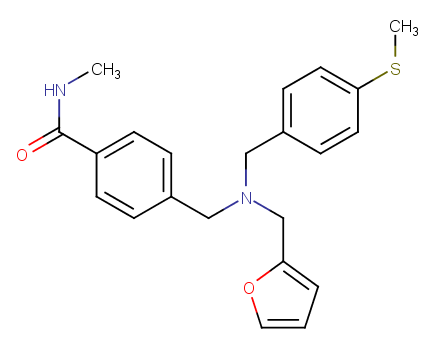

Supplement: RA-011-D1RA00914A-s1229 [file RA-011-D1RA00914A-s1229.png]

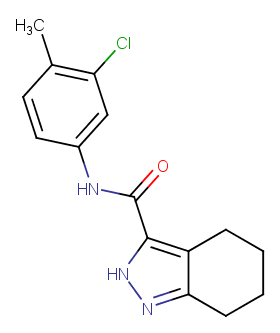

Supplement: RA-011-D1RA00914A-s1230 [file RA-011-D1RA00914A-s1230.png]

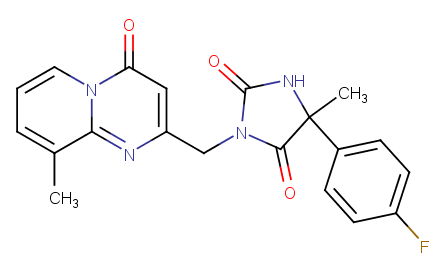

Supplement: RA-011-D1RA00914A-s1231 [file RA-011-D1RA00914A-s1231.png]

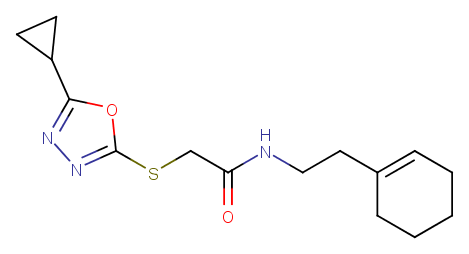

Supplement: RA-011-D1RA00914A-s1232 [file RA-011-D1RA00914A-s1232.png]

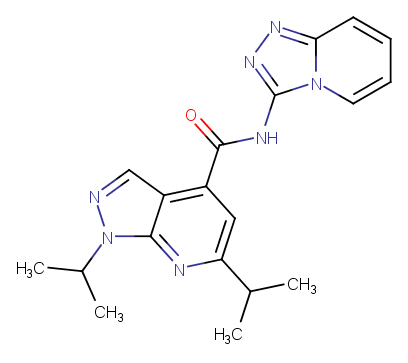

Supplement: RA-011-D1RA00914A-s1233 [file RA-011-D1RA00914A-s1233.png]

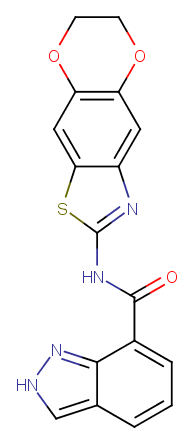

Supplement: RA-011-D1RA00914A-s1234 [file RA-011-D1RA00914A-s1234.png]

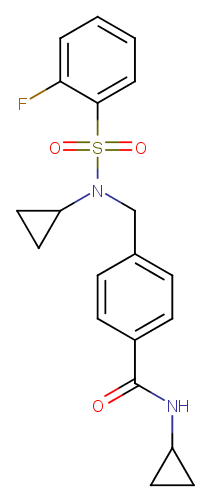

Supplement: RA-011-D1RA00914A-s1235 [file RA-011-D1RA00914A-s1235.png]

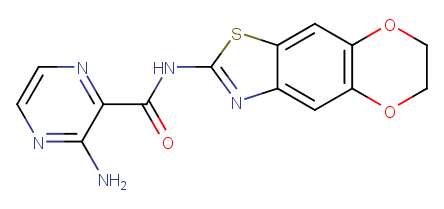

Supplement: RA-011-D1RA00914A-s1236 [file RA-011-D1RA00914A-s1236.png]

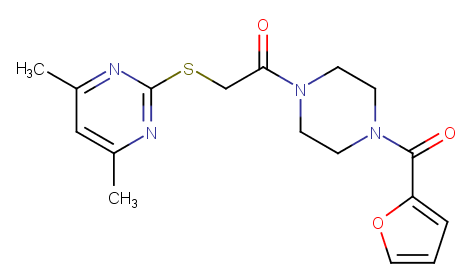

Supplement: RA-011-D1RA00914A-s1237 [file RA-011-D1RA00914A-s1237.png]

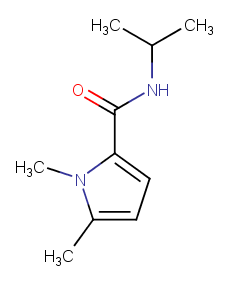

Supplement: RA-011-D1RA00914A-s1238 [file RA-011-D1RA00914A-s1238.png]

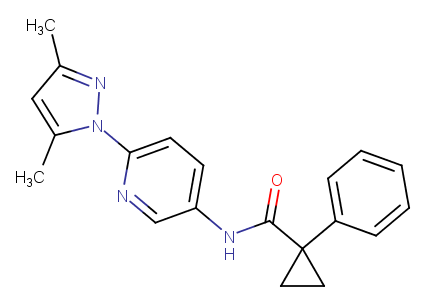

Supplement: RA-011-D1RA00914A-s1239 [file RA-011-D1RA00914A-s1239.png]

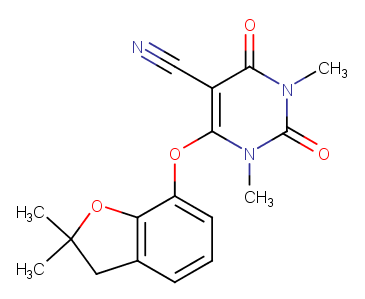

Supplement: RA-011-D1RA00914A-s1240 [file RA-011-D1RA00914A-s1240.png]

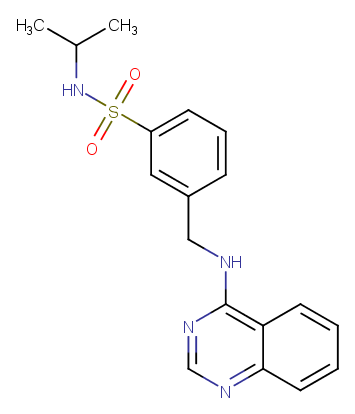

Supplement: RA-011-D1RA00914A-s1241 [file RA-011-D1RA00914A-s1241.png]

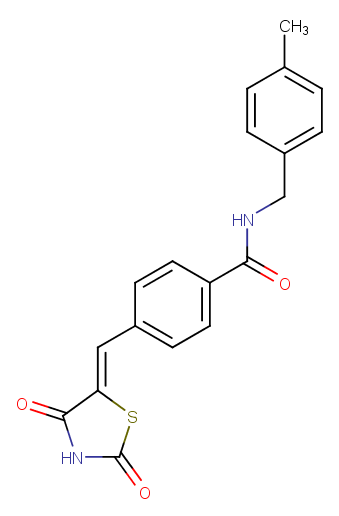

Supplement: RA-011-D1RA00914A-s1242 [file RA-011-D1RA00914A-s1242.png]

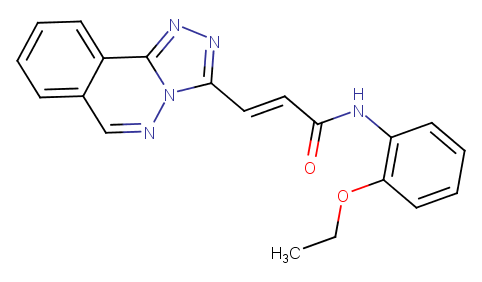

Supplement: RA-011-D1RA00914A-s1243 [file RA-011-D1RA00914A-s1243.png]

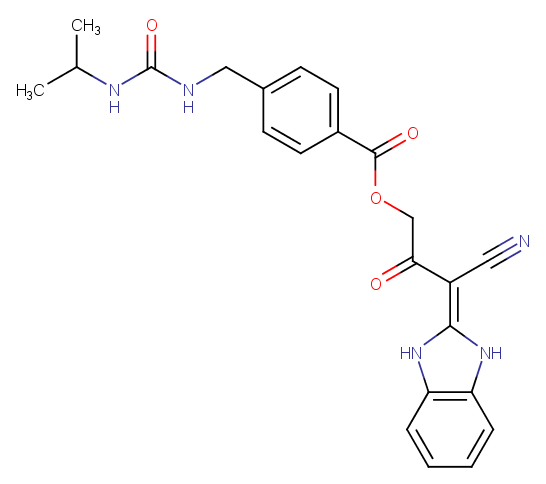

Supplement: RA-011-D1RA00914A-s1244 [file RA-011-D1RA00914A-s1244.png]

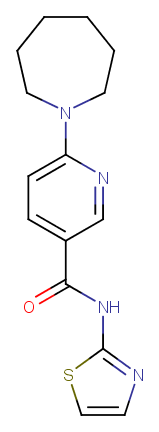

Supplement: RA-011-D1RA00914A-s1245 [file RA-011-D1RA00914A-s1245.png]

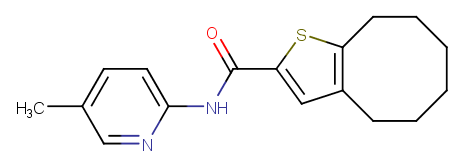

Supplement: RA-011-D1RA00914A-s1246 [file RA-011-D1RA00914A-s1246.png]

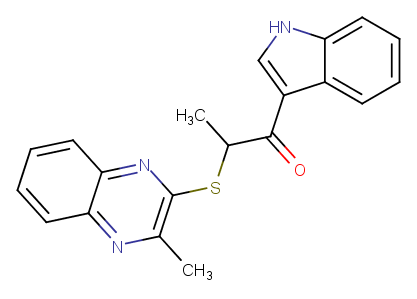

Supplement: RA-011-D1RA00914A-s1247 [file RA-011-D1RA00914A-s1247.png]

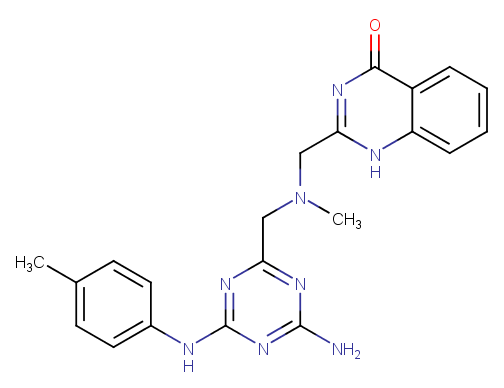

Supplement: RA-011-D1RA00914A-s1248 [file RA-011-D1RA00914A-s1248.png]

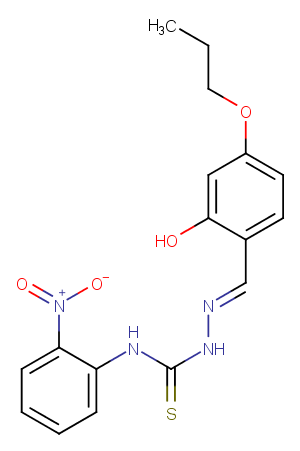

Supplement: RA-011-D1RA00914A-s1249 [file RA-011-D1RA00914A-s1249.png]

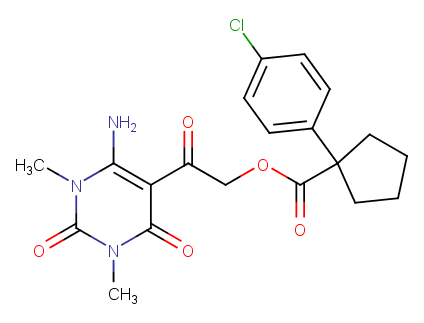

Supplement: RA-011-D1RA00914A-s1250 [file RA-011-D1RA00914A-s1250.png]

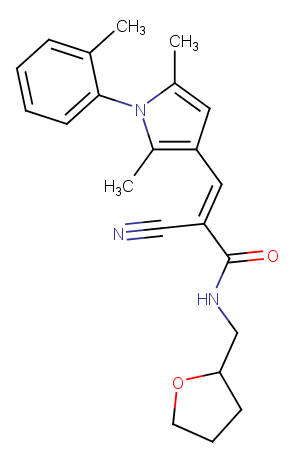

Supplement: RA-011-D1RA00914A-s1251 [file RA-011-D1RA00914A-s1251.png]

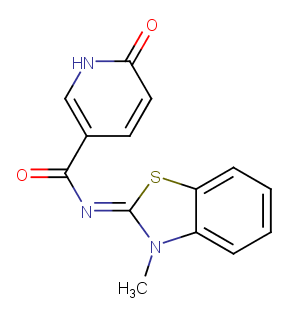

Supplement: RA-011-D1RA00914A-s1252 [file RA-011-D1RA00914A-s1252.png]

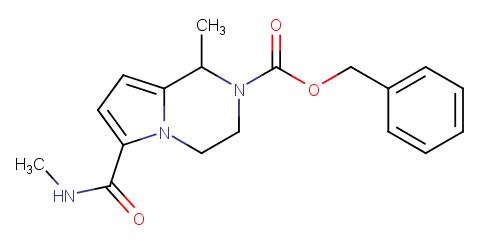

Supplement: RA-011-D1RA00914A-s1253 [file RA-011-D1RA00914A-s1253.png]

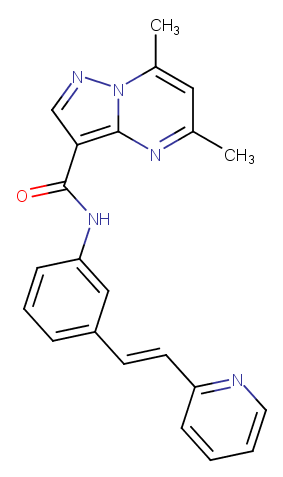

Supplement: RA-011-D1RA00914A-s1254 [file RA-011-D1RA00914A-s1254.png]

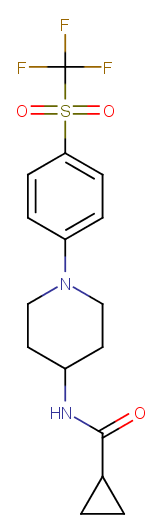

Supplement: RA-011-D1RA00914A-s1255 [file RA-011-D1RA00914A-s1255.png]

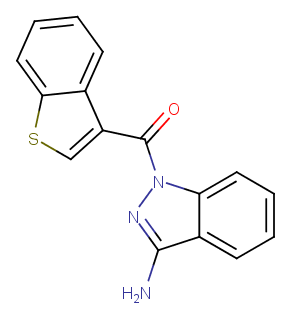

Supplement: RA-011-D1RA00914A-s1256 [file RA-011-D1RA00914A-s1256.png]

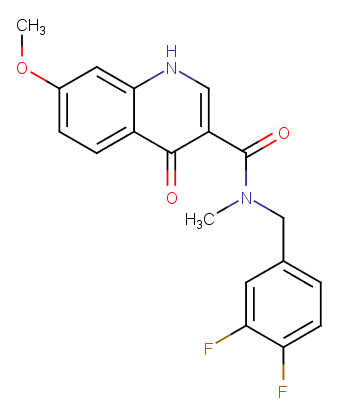

Supplement: RA-011-D1RA00914A-s1257 [file RA-011-D1RA00914A-s1257.png]

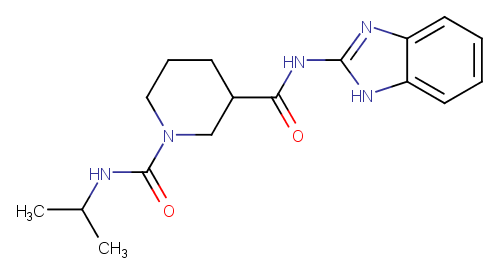

Supplement: RA-011-D1RA00914A-s1258 [file RA-011-D1RA00914A-s1258.png]

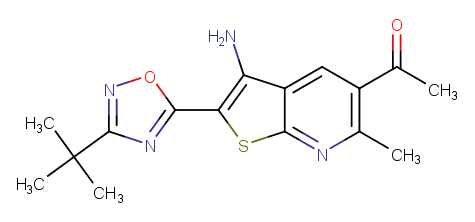

Supplement: RA-011-D1RA00914A-s1259 [file RA-011-D1RA00914A-s1259.png]

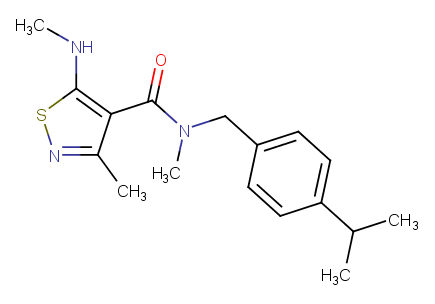

Supplement: RA-011-D1RA00914A-s1260 [file RA-011-D1RA00914A-s1260.png]

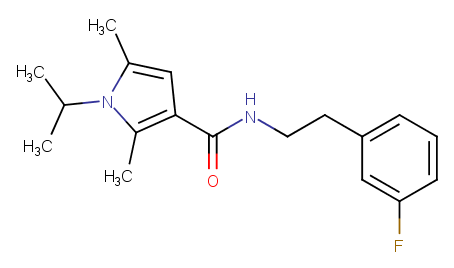

Supplement: RA-011-D1RA00914A-s1261 [file RA-011-D1RA00914A-s1261.png]

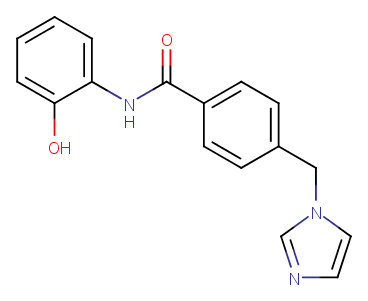

Supplement: RA-011-D1RA00914A-s1262 [file RA-011-D1RA00914A-s1262.png]

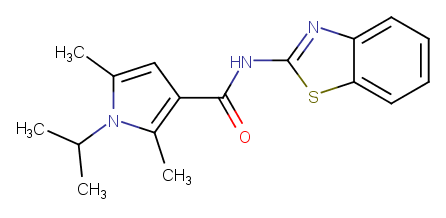

Supplement: RA-011-D1RA00914A-s1263 [file RA-011-D1RA00914A-s1263.png]

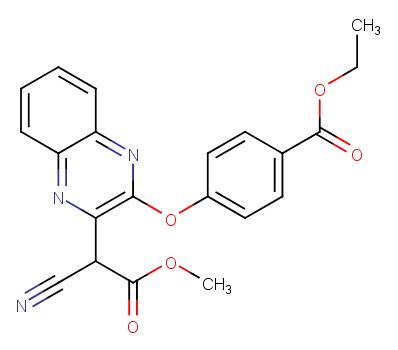

Supplement: RA-011-D1RA00914A-s1264 [file RA-011-D1RA00914A-s1264.png]

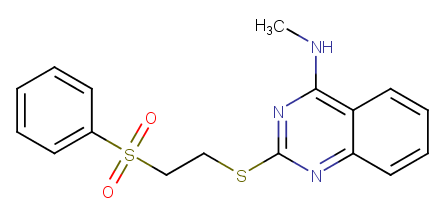

Supplement: RA-011-D1RA00914A-s1265 [file RA-011-D1RA00914A-s1265.png]

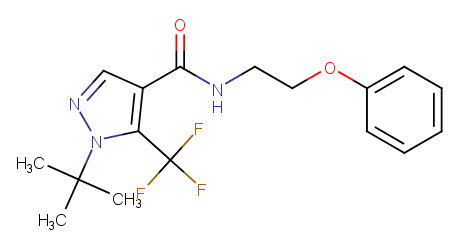

Supplement: RA-011-D1RA00914A-s1266 [file RA-011-D1RA00914A-s1266.png]

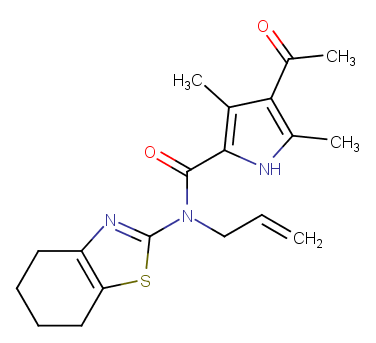

Supplement: RA-011-D1RA00914A-s1267 [file RA-011-D1RA00914A-s1267.png]

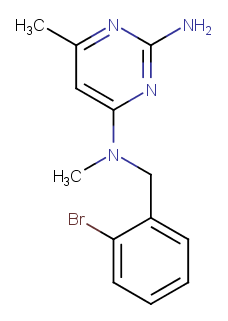

Supplement: RA-011-D1RA00914A-s1268 [file RA-011-D1RA00914A-s1268.png]

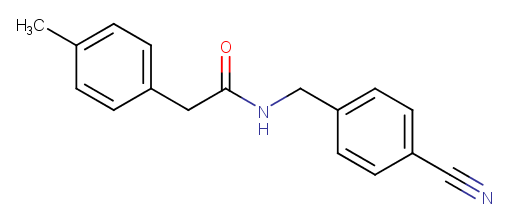

Supplement: RA-011-D1RA00914A-s1269 [file RA-011-D1RA00914A-s1269.png]

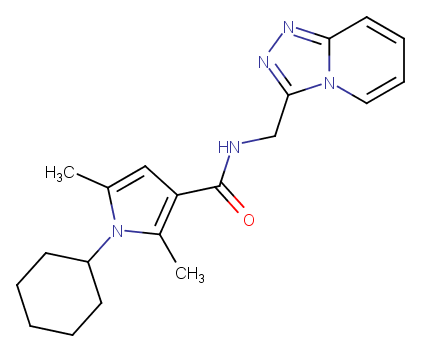

Supplement: RA-011-D1RA00914A-s1270 [file RA-011-D1RA00914A-s1270.png]

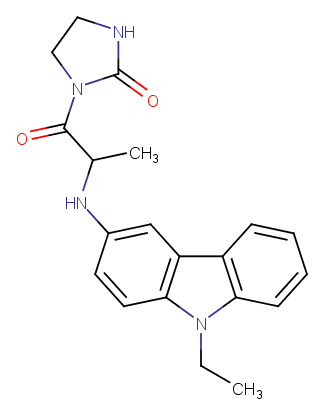

Supplement: RA-011-D1RA00914A-s1271 [file RA-011-D1RA00914A-s1271.png]

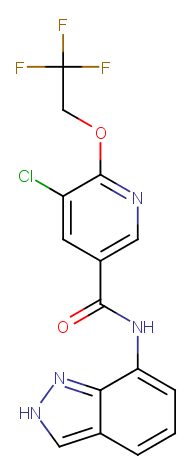

Supplement: RA-011-D1RA00914A-s1272 [file RA-011-D1RA00914A-s1272.png]

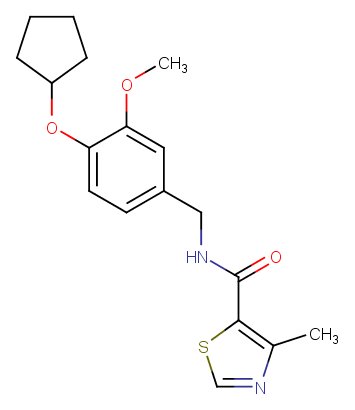

Supplement: RA-011-D1RA00914A-s1273 [file RA-011-D1RA00914A-s1273.png]
