# Supplementary figures and images for: Improved herbicide discovery using physico-chemical rules refined by antimalarial library screening (part 10 of 14)
Source: RSC Adv. 2021 Feb 23;11(15):8459–67. doi: 10.1039/d1ra00914a (PMC8695207; doi:10.1039/d1ra00914a)

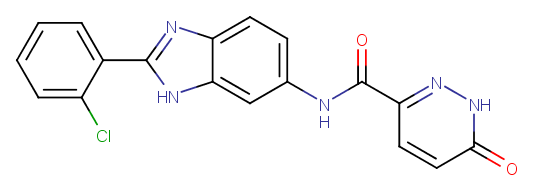

Supplement: RA-011-D1RA00914A-s1274 [file RA-011-D1RA00914A-s1274.png]

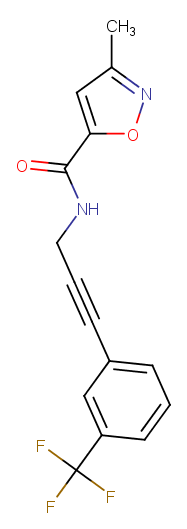

Supplement: RA-011-D1RA00914A-s1275 [file RA-011-D1RA00914A-s1275.png]

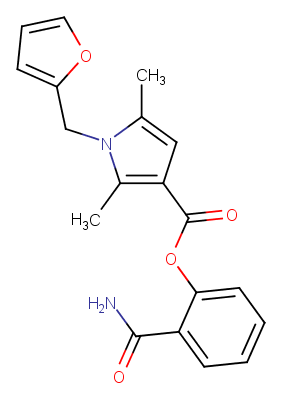

Supplement: RA-011-D1RA00914A-s1276 [file RA-011-D1RA00914A-s1276.png]

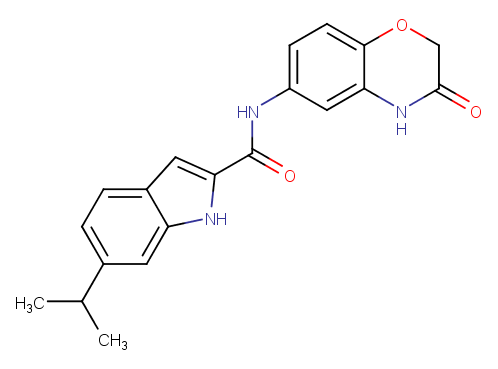

Supplement: RA-011-D1RA00914A-s1277 [file RA-011-D1RA00914A-s1277.png]

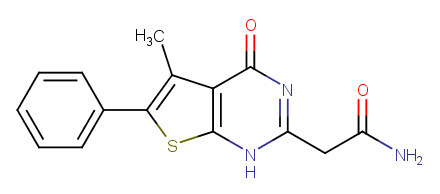

Supplement: RA-011-D1RA00914A-s1278 [file RA-011-D1RA00914A-s1278.png]

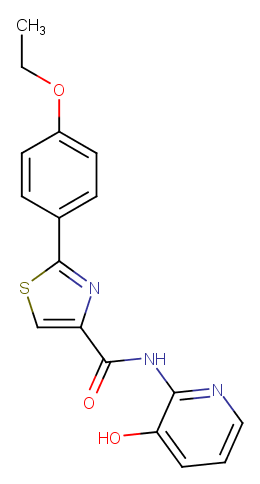

Supplement: RA-011-D1RA00914A-s1279 [file RA-011-D1RA00914A-s1279.png]

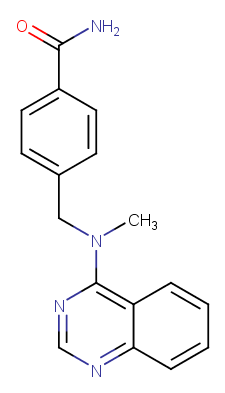

Supplement: RA-011-D1RA00914A-s1280 [file RA-011-D1RA00914A-s1280.png]

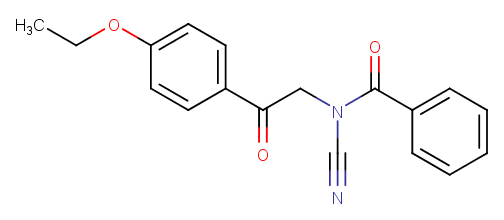

Supplement: RA-011-D1RA00914A-s1281 [file RA-011-D1RA00914A-s1281.png]

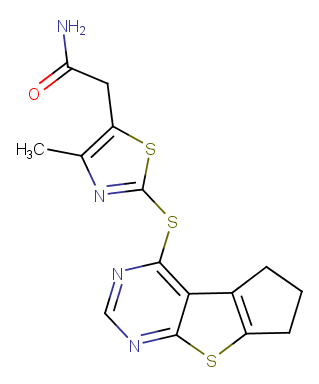

Supplement: RA-011-D1RA00914A-s1282 [file RA-011-D1RA00914A-s1282.png]

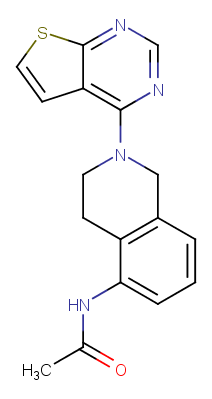

Supplement: RA-011-D1RA00914A-s1283 [file RA-011-D1RA00914A-s1283.png]

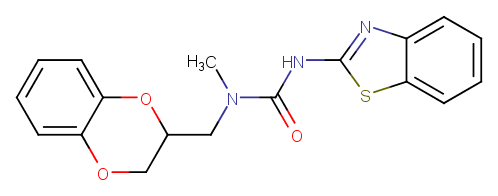

Supplement: RA-011-D1RA00914A-s1284 [file RA-011-D1RA00914A-s1284.png]

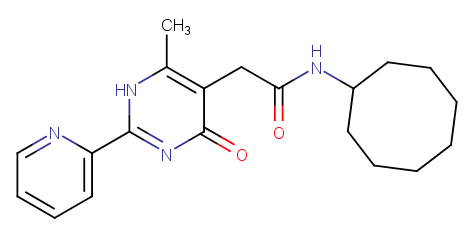

Supplement: RA-011-D1RA00914A-s1285 [file RA-011-D1RA00914A-s1285.png]

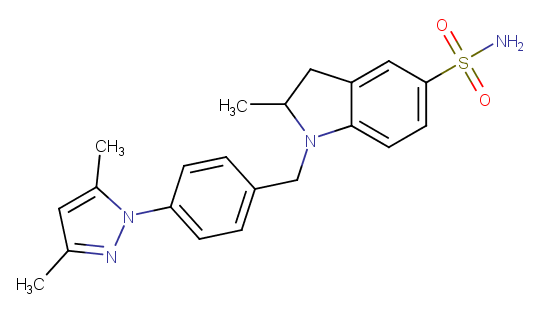

Supplement: RA-011-D1RA00914A-s1286 [file RA-011-D1RA00914A-s1286.png]

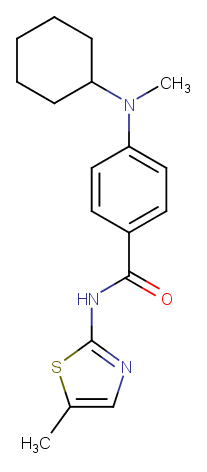

Supplement: RA-011-D1RA00914A-s1287 [file RA-011-D1RA00914A-s1287.png]

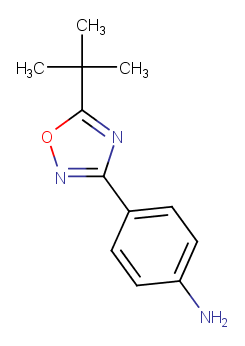

Supplement: RA-011-D1RA00914A-s1288 [file RA-011-D1RA00914A-s1288.png]

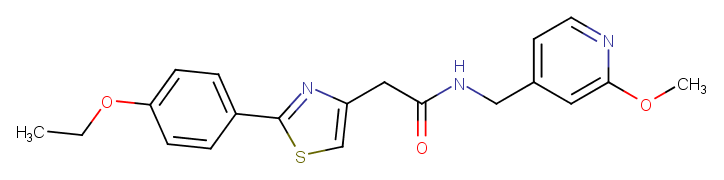

Supplement: RA-011-D1RA00914A-s1289 [file RA-011-D1RA00914A-s1289.png]

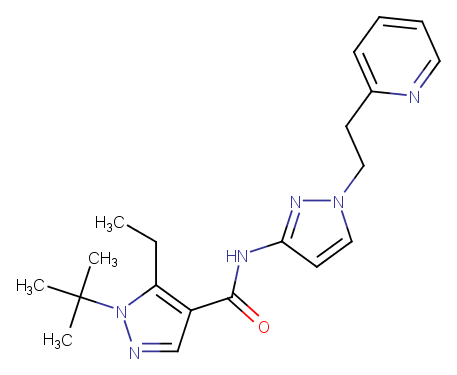

Supplement: RA-011-D1RA00914A-s1290 [file RA-011-D1RA00914A-s1290.png]

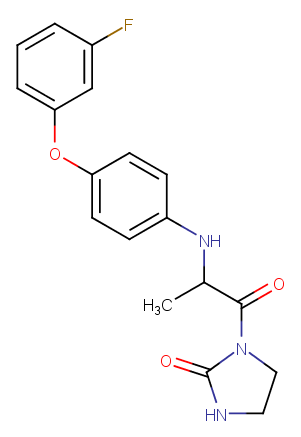

Supplement: RA-011-D1RA00914A-s1291 [file RA-011-D1RA00914A-s1291.png]

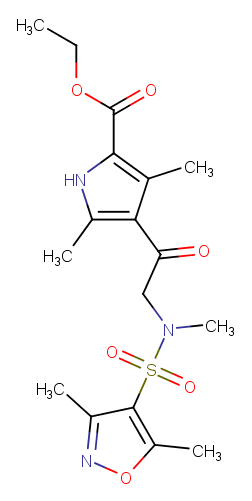

Supplement: RA-011-D1RA00914A-s1292 [file RA-011-D1RA00914A-s1292.png]

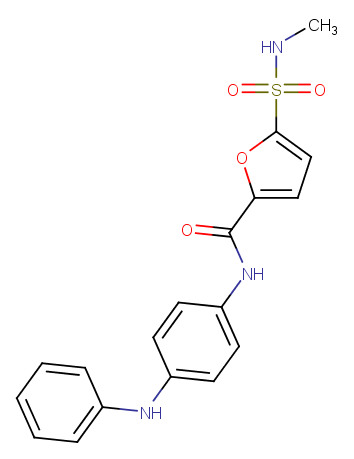

Supplement: RA-011-D1RA00914A-s1293 [file RA-011-D1RA00914A-s1293.png]

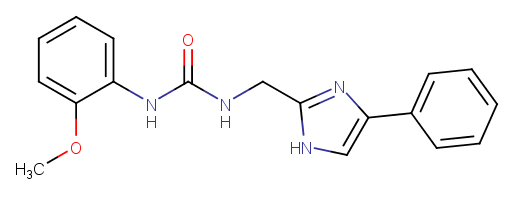

Supplement: RA-011-D1RA00914A-s1294 [file RA-011-D1RA00914A-s1294.png]

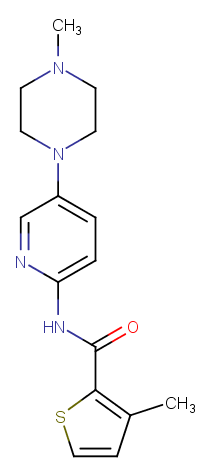

Supplement: RA-011-D1RA00914A-s1295 [file RA-011-D1RA00914A-s1295.png]

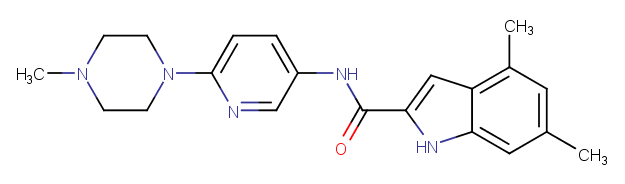

Supplement: RA-011-D1RA00914A-s1296 [file RA-011-D1RA00914A-s1296.png]

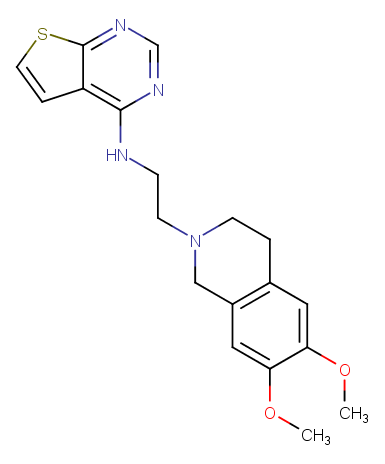

Supplement: RA-011-D1RA00914A-s1297 [file RA-011-D1RA00914A-s1297.png]

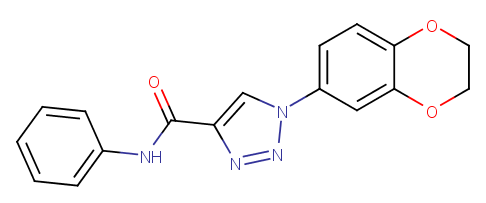

Supplement: RA-011-D1RA00914A-s1298 [file RA-011-D1RA00914A-s1298.png]

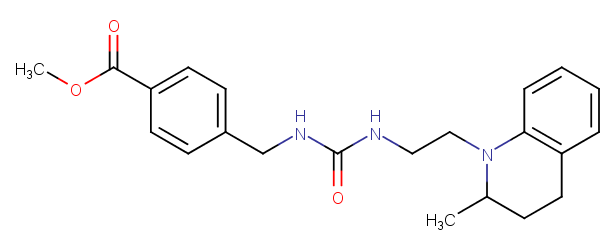

Supplement: RA-011-D1RA00914A-s1299 [file RA-011-D1RA00914A-s1299.png]

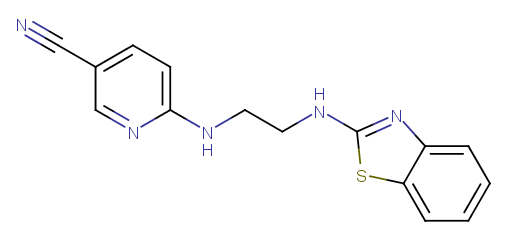

Supplement: RA-011-D1RA00914A-s1300 [file RA-011-D1RA00914A-s1300.png]

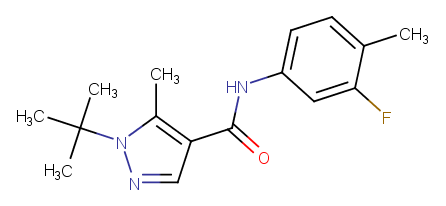

Supplement: RA-011-D1RA00914A-s1301 [file RA-011-D1RA00914A-s1301.png]

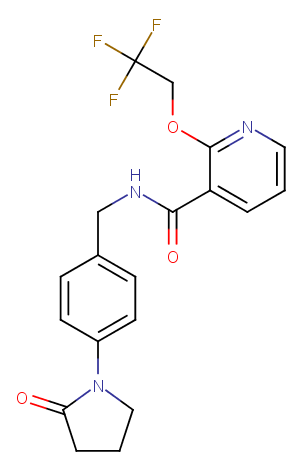

Supplement: RA-011-D1RA00914A-s1302 [file RA-011-D1RA00914A-s1302.png]

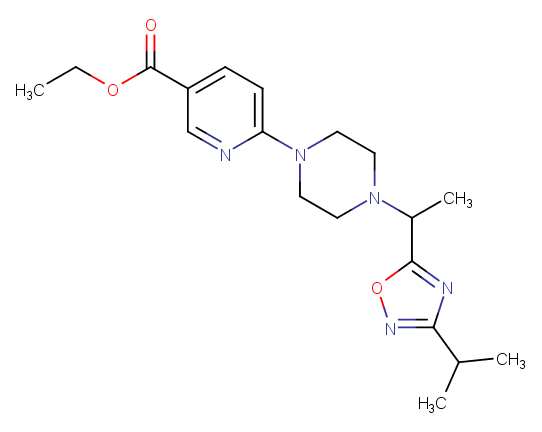

Supplement: RA-011-D1RA00914A-s1303 [file RA-011-D1RA00914A-s1303.png]

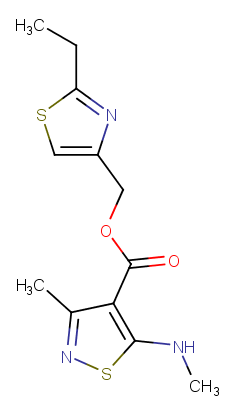

Supplement: RA-011-D1RA00914A-s1304 [file RA-011-D1RA00914A-s1304.png]

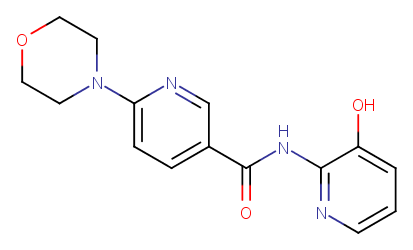

Supplement: RA-011-D1RA00914A-s1305 [file RA-011-D1RA00914A-s1305.png]

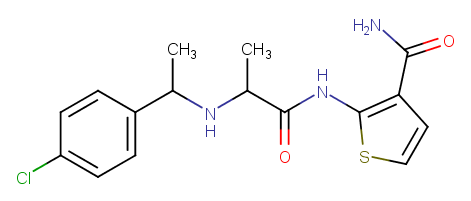

Supplement: RA-011-D1RA00914A-s1306 [file RA-011-D1RA00914A-s1306.png]

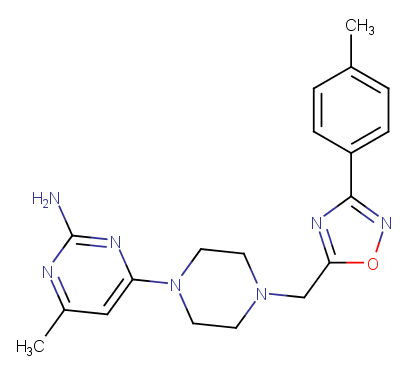

Supplement: RA-011-D1RA00914A-s1307 [file RA-011-D1RA00914A-s1307.png]

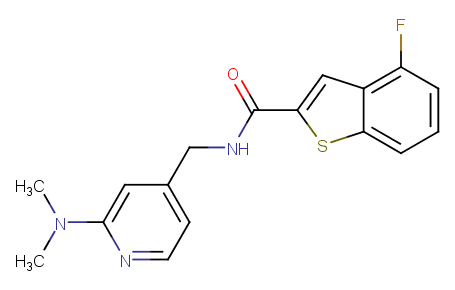

Supplement: RA-011-D1RA00914A-s1308 [file RA-011-D1RA00914A-s1308.png]

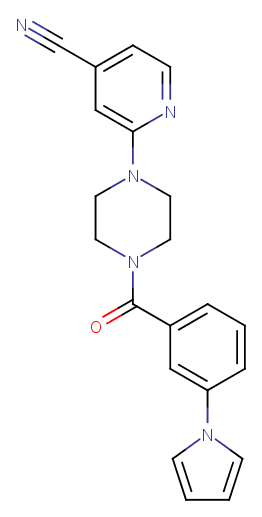

Supplement: RA-011-D1RA00914A-s1309 [file RA-011-D1RA00914A-s1309.png]

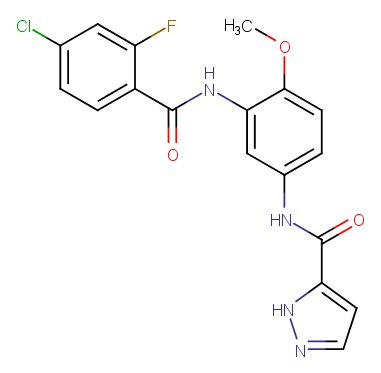

Supplement: RA-011-D1RA00914A-s1310 [file RA-011-D1RA00914A-s1310.png]

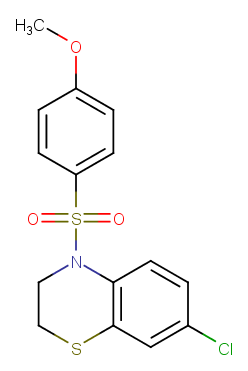

Supplement: RA-011-D1RA00914A-s1311 [file RA-011-D1RA00914A-s1311.png]

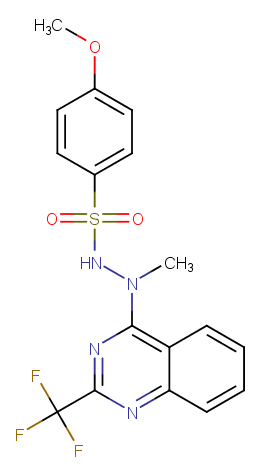

Supplement: RA-011-D1RA00914A-s1312 [file RA-011-D1RA00914A-s1312.png]

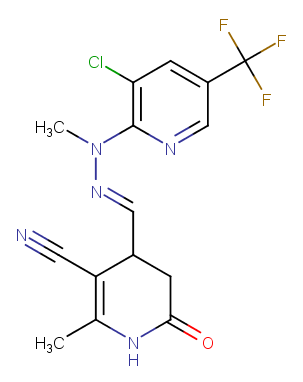

Supplement: RA-011-D1RA00914A-s1313 [file RA-011-D1RA00914A-s1313.png]

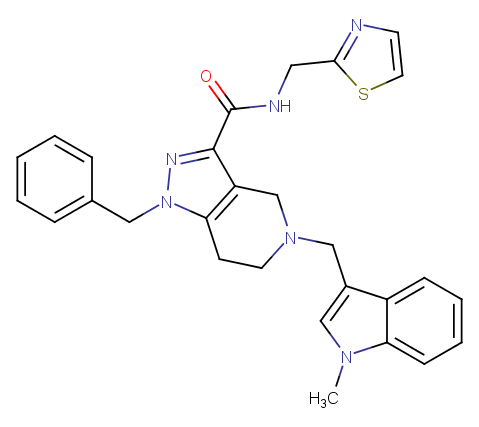

Supplement: RA-011-D1RA00914A-s1314 [file RA-011-D1RA00914A-s1314.png]

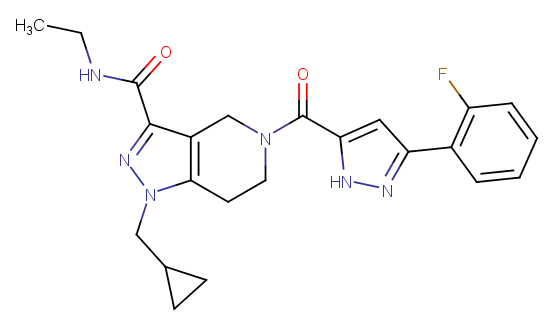

Supplement: RA-011-D1RA00914A-s1315 [file RA-011-D1RA00914A-s1315.png]

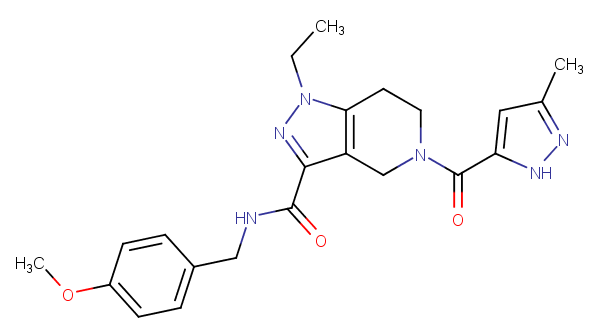

Supplement: RA-011-D1RA00914A-s1316 [file RA-011-D1RA00914A-s1316.png]

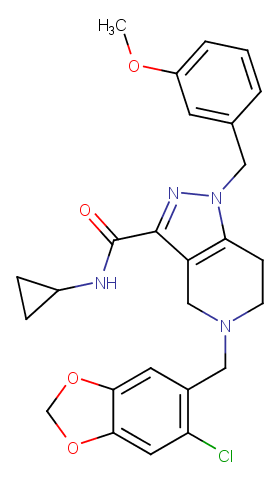

Supplement: RA-011-D1RA00914A-s1317 [file RA-011-D1RA00914A-s1317.png]

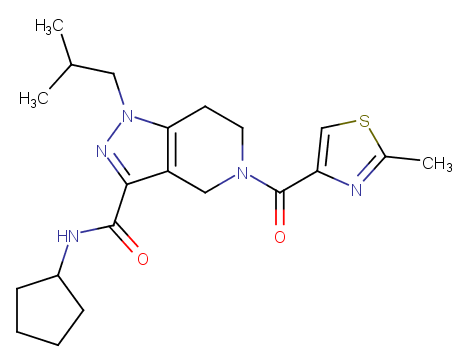

Supplement: RA-011-D1RA00914A-s1318 [file RA-011-D1RA00914A-s1318.png]

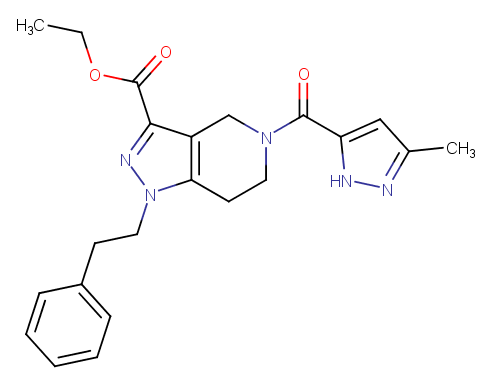

Supplement: RA-011-D1RA00914A-s1319 [file RA-011-D1RA00914A-s1319.png]

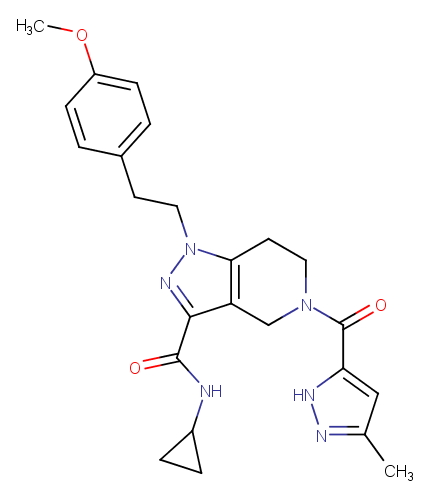

Supplement: RA-011-D1RA00914A-s1320 [file RA-011-D1RA00914A-s1320.png]

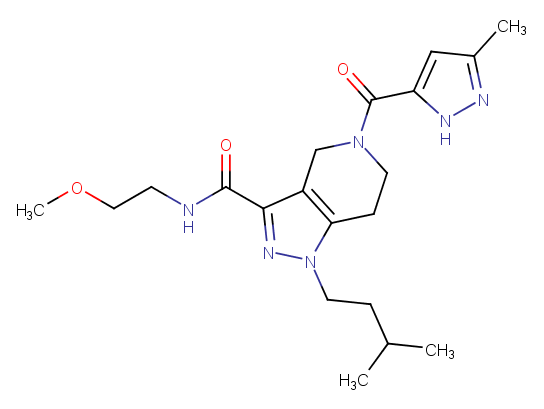

Supplement: RA-011-D1RA00914A-s1321 [file RA-011-D1RA00914A-s1321.png]

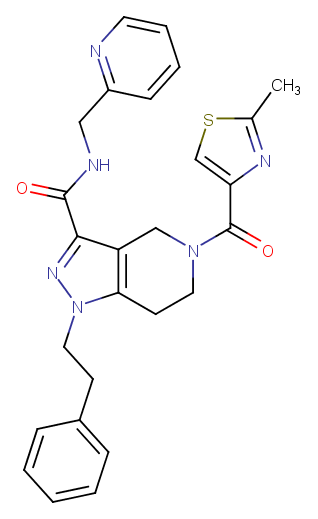

Supplement: RA-011-D1RA00914A-s1322 [file RA-011-D1RA00914A-s1322.png]

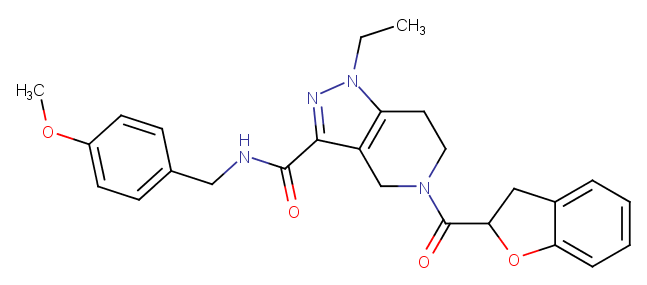

Supplement: RA-011-D1RA00914A-s1323 [file RA-011-D1RA00914A-s1323.png]

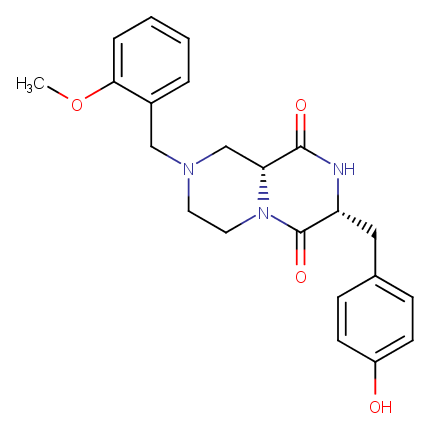

Supplement: RA-011-D1RA00914A-s1324 [file RA-011-D1RA00914A-s1324.png]

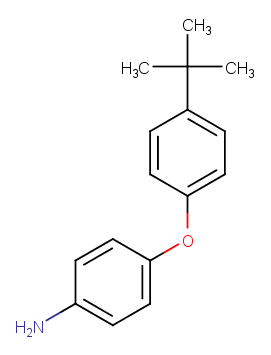

Supplement: RA-011-D1RA00914A-s1325 [file RA-011-D1RA00914A-s1325.png]

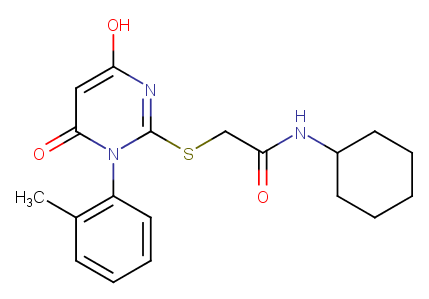

Supplement: RA-011-D1RA00914A-s1326 [file RA-011-D1RA00914A-s1326.png]

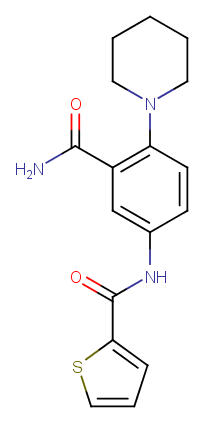

Supplement: RA-011-D1RA00914A-s1327 [file RA-011-D1RA00914A-s1327.png]

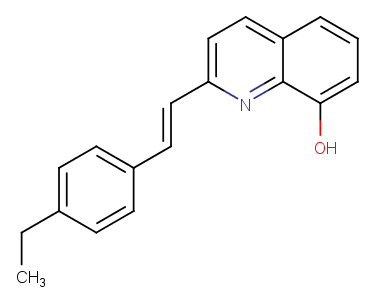

Supplement: RA-011-D1RA00914A-s1328 [file RA-011-D1RA00914A-s1328.png]

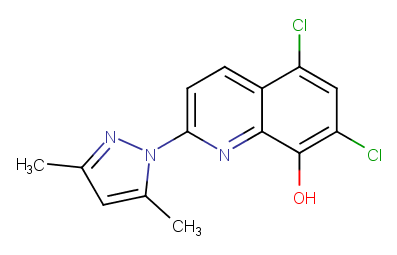

Supplement: RA-011-D1RA00914A-s1329 [file RA-011-D1RA00914A-s1329.png]

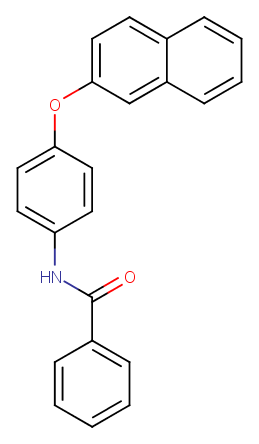

Supplement: RA-011-D1RA00914A-s1330 [file RA-011-D1RA00914A-s1330.png]

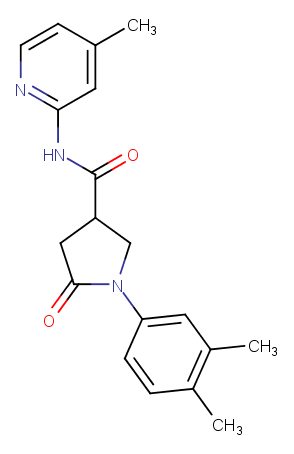

Supplement: RA-011-D1RA00914A-s1331 [file RA-011-D1RA00914A-s1331.png]

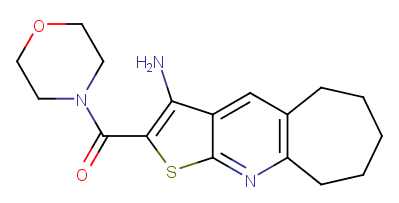

Supplement: RA-011-D1RA00914A-s1332 [file RA-011-D1RA00914A-s1332.png]

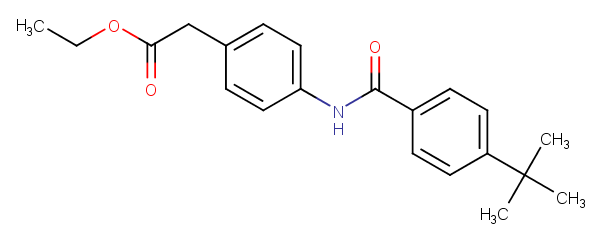

Supplement: RA-011-D1RA00914A-s1333 [file RA-011-D1RA00914A-s1333.png]

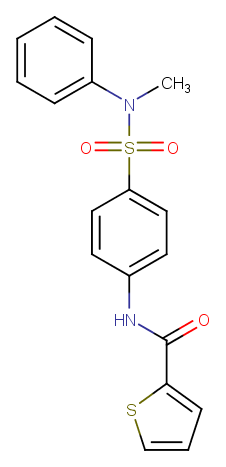

Supplement: RA-011-D1RA00914A-s1334 [file RA-011-D1RA00914A-s1334.png]

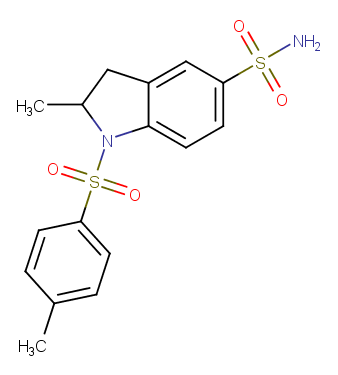

Supplement: RA-011-D1RA00914A-s1335 [file RA-011-D1RA00914A-s1335.png]

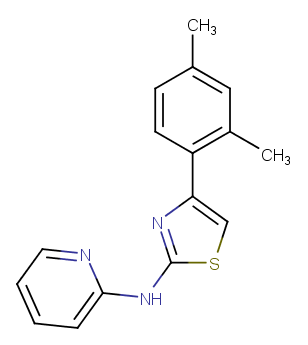

Supplement: RA-011-D1RA00914A-s1336 [file RA-011-D1RA00914A-s1336.png]

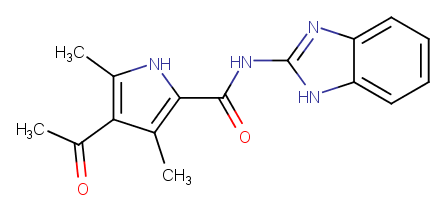

Supplement: RA-011-D1RA00914A-s1337 [file RA-011-D1RA00914A-s1337.png]

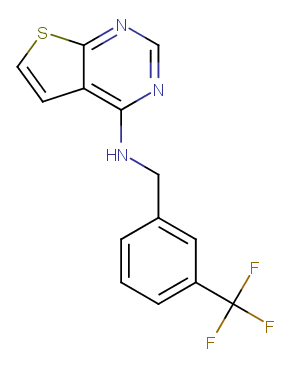

Supplement: RA-011-D1RA00914A-s1338 [file RA-011-D1RA00914A-s1338.png]

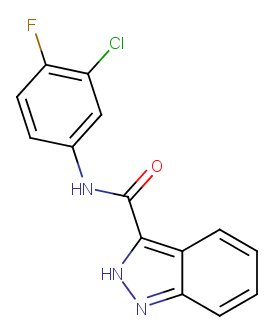

Supplement: RA-011-D1RA00914A-s1339 [file RA-011-D1RA00914A-s1339.png]

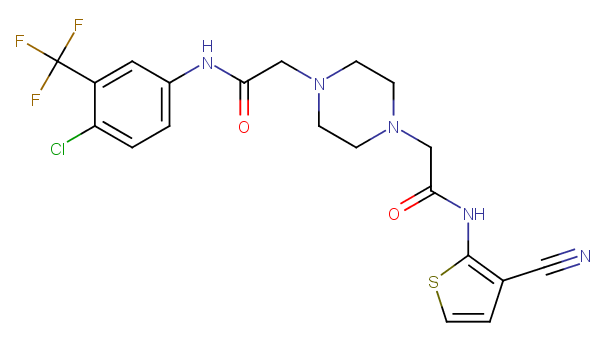

Supplement: RA-011-D1RA00914A-s1340 [file RA-011-D1RA00914A-s1340.png]

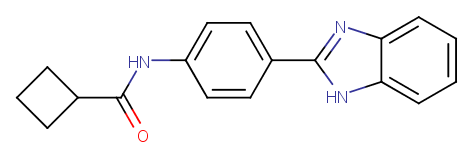

Supplement: RA-011-D1RA00914A-s1341 [file RA-011-D1RA00914A-s1341.png]

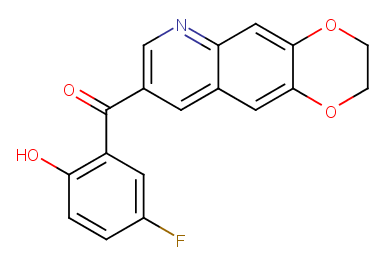

Supplement: RA-011-D1RA00914A-s1342 [file RA-011-D1RA00914A-s1342.png]

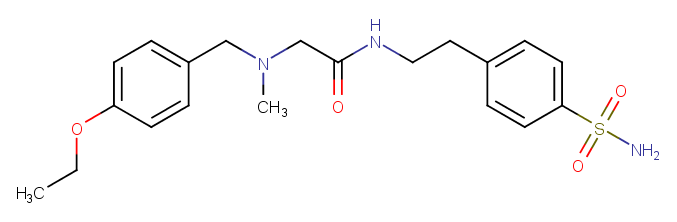

Supplement: RA-011-D1RA00914A-s1343 [file RA-011-D1RA00914A-s1343.png]

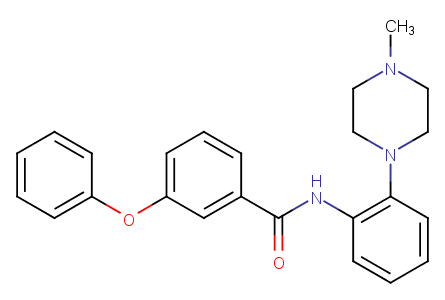

Supplement: RA-011-D1RA00914A-s1344 [file RA-011-D1RA00914A-s1344.png]

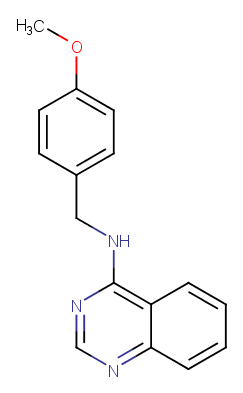

Supplement: RA-011-D1RA00914A-s1345 [file RA-011-D1RA00914A-s1345.png]

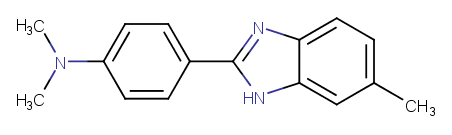

Supplement: RA-011-D1RA00914A-s1346 [file RA-011-D1RA00914A-s1346.png]

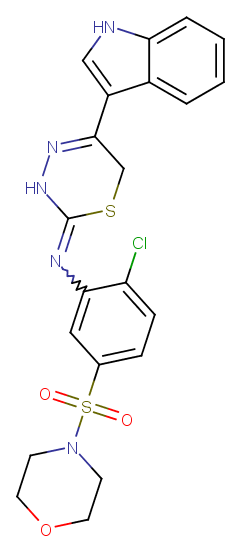

Supplement: RA-011-D1RA00914A-s1347 [file RA-011-D1RA00914A-s1347.png]

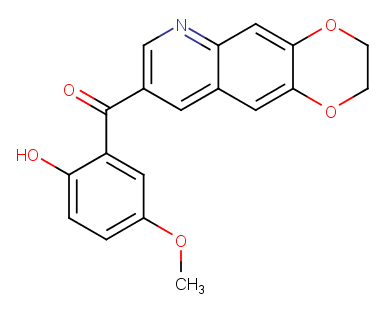

Supplement: RA-011-D1RA00914A-s1348 [file RA-011-D1RA00914A-s1348.png]

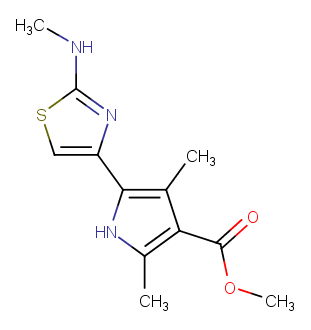

Supplement: RA-011-D1RA00914A-s1349 [file RA-011-D1RA00914A-s1349.png]

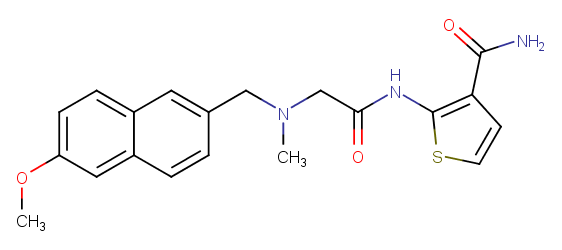

Supplement: RA-011-D1RA00914A-s1350 [file RA-011-D1RA00914A-s1350.png]

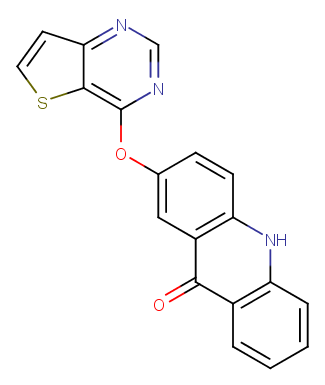

Supplement: RA-011-D1RA00914A-s1351 [file RA-011-D1RA00914A-s1351.png]

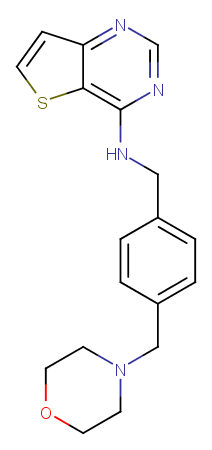

Supplement: RA-011-D1RA00914A-s1352 [file RA-011-D1RA00914A-s1352.png]

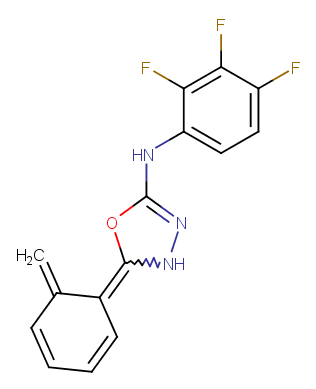

Supplement: RA-011-D1RA00914A-s1353 [file RA-011-D1RA00914A-s1353.png]

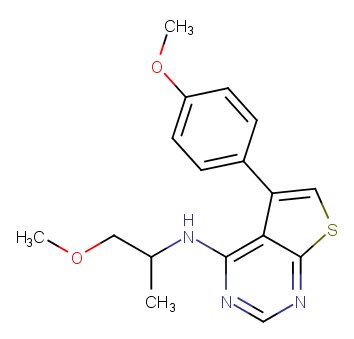

Supplement: RA-011-D1RA00914A-s1354 [file RA-011-D1RA00914A-s1354.png]

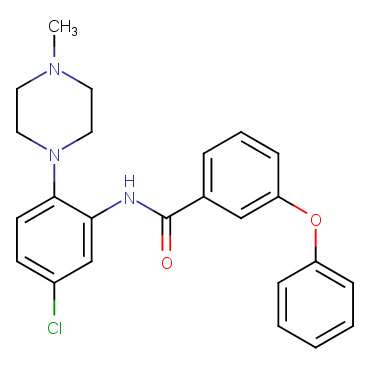

Supplement: RA-011-D1RA00914A-s1355 [file RA-011-D1RA00914A-s1355.png]

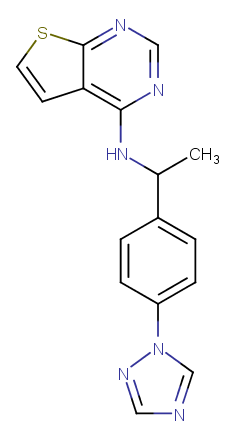

Supplement: RA-011-D1RA00914A-s1356 [file RA-011-D1RA00914A-s1356.png]

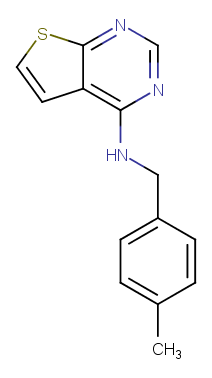

Supplement: RA-011-D1RA00914A-s1357 [file RA-011-D1RA00914A-s1357.png]

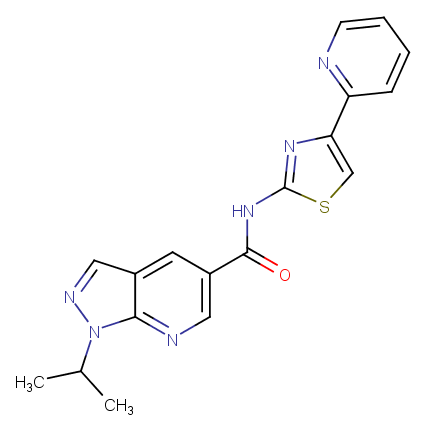

Supplement: RA-011-D1RA00914A-s1358 [file RA-011-D1RA00914A-s1358.png]

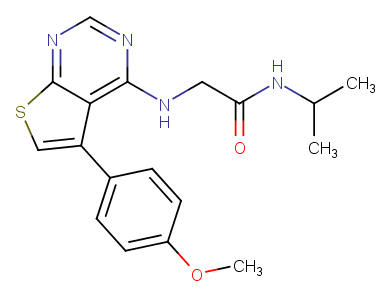

Supplement: RA-011-D1RA00914A-s1359 [file RA-011-D1RA00914A-s1359.png]

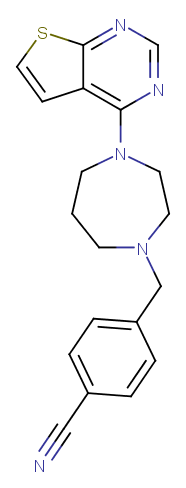

Supplement: RA-011-D1RA00914A-s1360 [file RA-011-D1RA00914A-s1360.png]

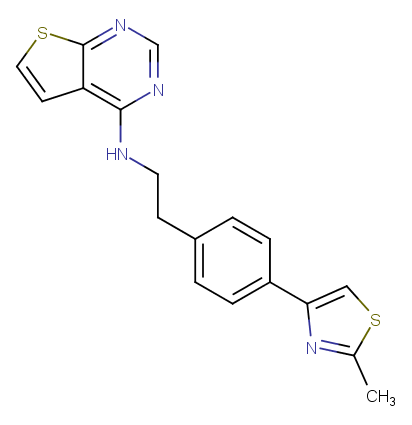

Supplement: RA-011-D1RA00914A-s1361 [file RA-011-D1RA00914A-s1361.png]

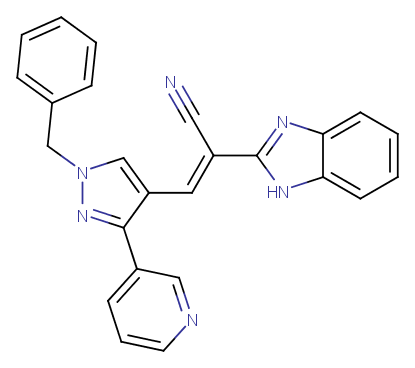

Supplement: RA-011-D1RA00914A-s1362 [file RA-011-D1RA00914A-s1362.png]

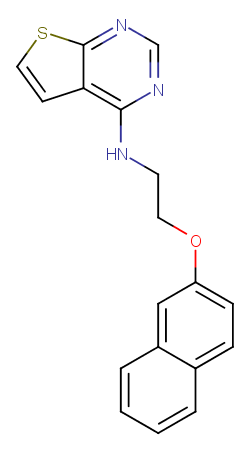

Supplement: RA-011-D1RA00914A-s1363 [file RA-011-D1RA00914A-s1363.png]

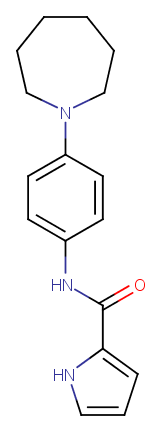

Supplement: RA-011-D1RA00914A-s1364 [file RA-011-D1RA00914A-s1364.png]

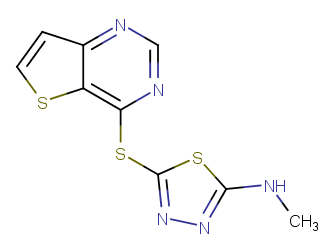

Supplement: RA-011-D1RA00914A-s1365 [file RA-011-D1RA00914A-s1365.png]

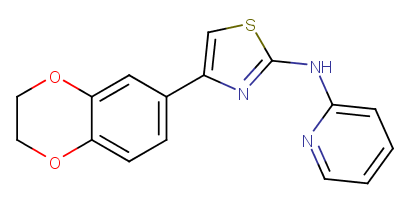

Supplement: RA-011-D1RA00914A-s1366 [file RA-011-D1RA00914A-s1366.png]

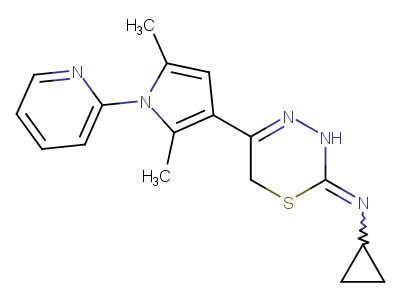

Supplement: RA-011-D1RA00914A-s1367 [file RA-011-D1RA00914A-s1367.png]

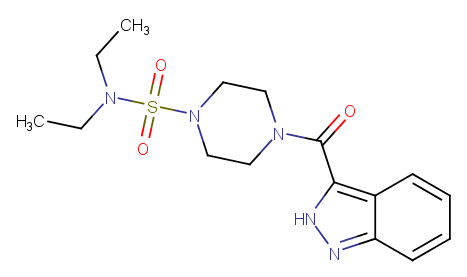

Supplement: RA-011-D1RA00914A-s1368 [file RA-011-D1RA00914A-s1368.png]

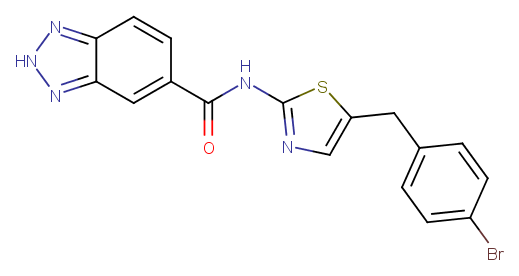

Supplement: RA-011-D1RA00914A-s1369 [file RA-011-D1RA00914A-s1369.png]

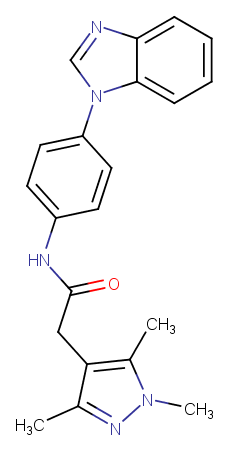

Supplement: RA-011-D1RA00914A-s1370 [file RA-011-D1RA00914A-s1370.png]

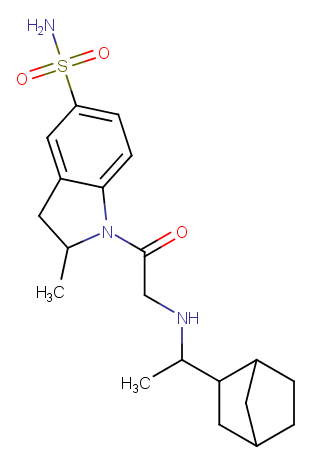

Supplement: RA-011-D1RA00914A-s1371 [file RA-011-D1RA00914A-s1371.png]

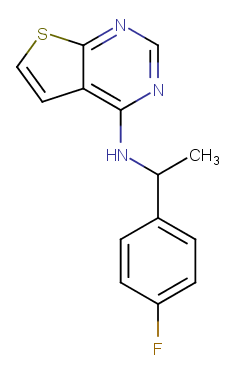

Supplement: RA-011-D1RA00914A-s1372 [file RA-011-D1RA00914A-s1372.png]

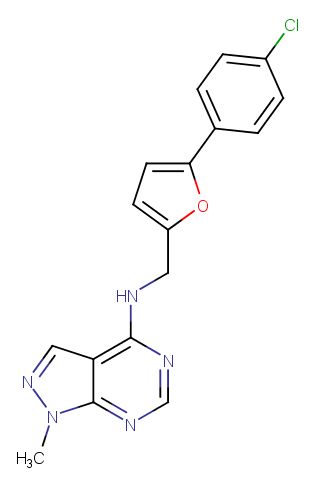

Supplement: RA-011-D1RA00914A-s1373 [file RA-011-D1RA00914A-s1373.png]
