# Supplementary figures and images for: Improved herbicide discovery using physico-chemical rules refined by antimalarial library screening (part 11 of 14)
Source: RSC Adv. 2021 Feb 23;11(15):8459–67. doi: 10.1039/d1ra00914a (PMC8695207; doi:10.1039/d1ra00914a)

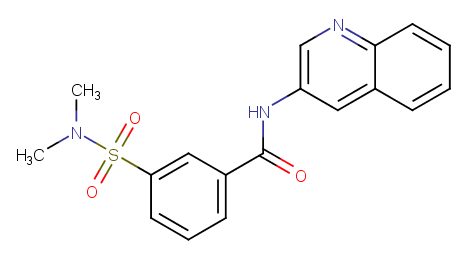

Supplement: RA-011-D1RA00914A-s1374 [file RA-011-D1RA00914A-s1374.png]

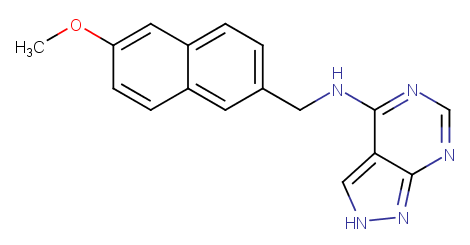

Supplement: RA-011-D1RA00914A-s1375 [file RA-011-D1RA00914A-s1375.png]

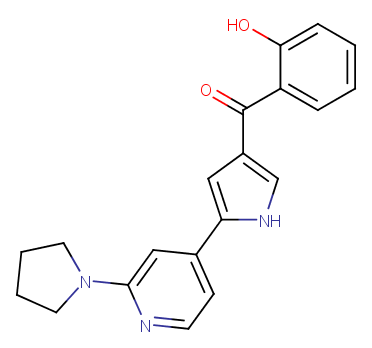

Supplement: RA-011-D1RA00914A-s1376 [file RA-011-D1RA00914A-s1376.png]

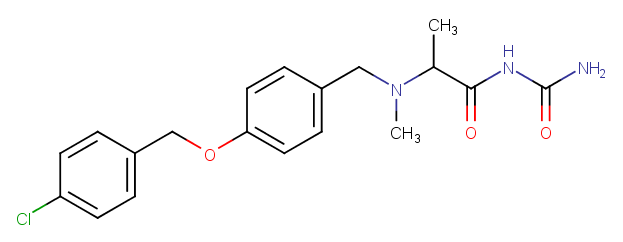

Supplement: RA-011-D1RA00914A-s1377 [file RA-011-D1RA00914A-s1377.png]

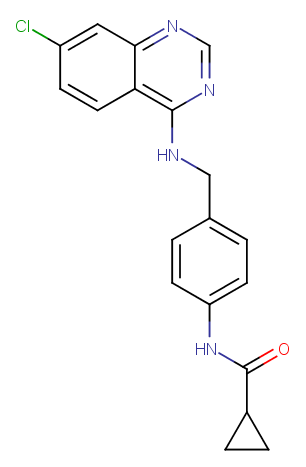

Supplement: RA-011-D1RA00914A-s1378 [file RA-011-D1RA00914A-s1378.png]

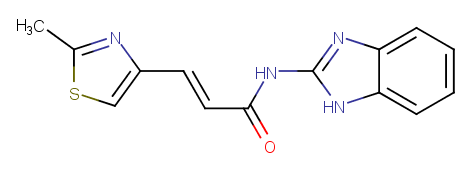

Supplement: RA-011-D1RA00914A-s1379 [file RA-011-D1RA00914A-s1379.png]

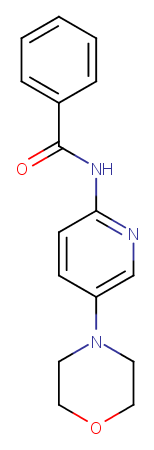

Supplement: RA-011-D1RA00914A-s1380 [file RA-011-D1RA00914A-s1380.png]

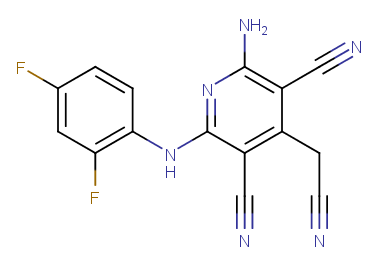

Supplement: RA-011-D1RA00914A-s1381 [file RA-011-D1RA00914A-s1381.png]

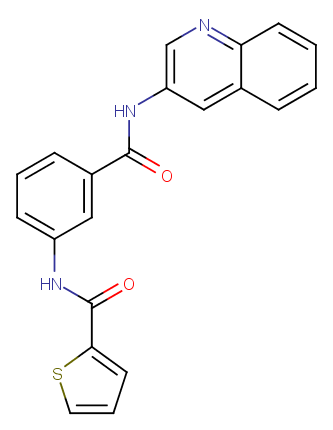

Supplement: RA-011-D1RA00914A-s1382 [file RA-011-D1RA00914A-s1382.png]

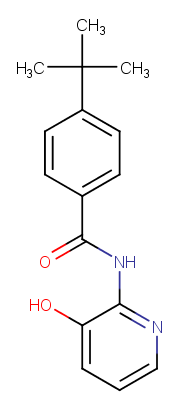

Supplement: RA-011-D1RA00914A-s1383 [file RA-011-D1RA00914A-s1383.png]

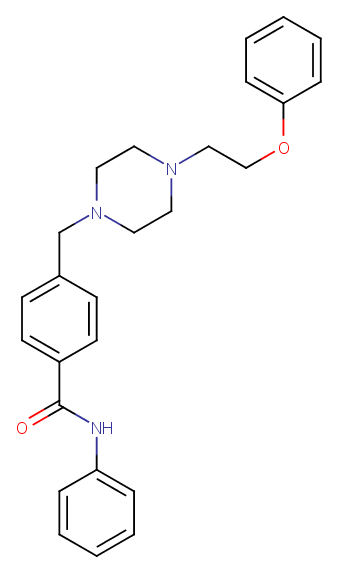

Supplement: RA-011-D1RA00914A-s1384 [file RA-011-D1RA00914A-s1384.png]

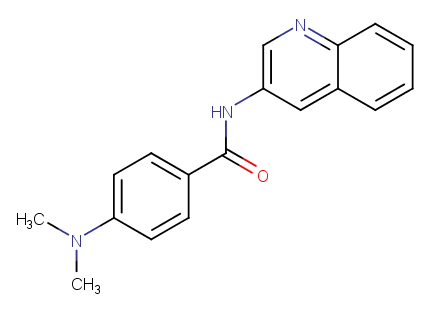

Supplement: RA-011-D1RA00914A-s1385 [file RA-011-D1RA00914A-s1385.png]

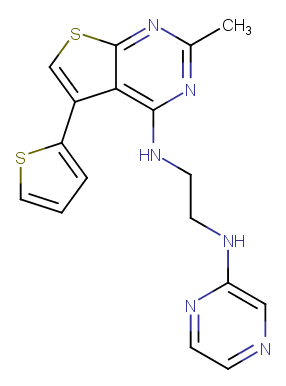

Supplement: RA-011-D1RA00914A-s1386 [file RA-011-D1RA00914A-s1386.png]

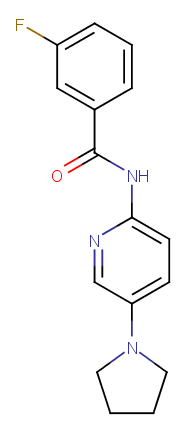

Supplement: RA-011-D1RA00914A-s1387 [file RA-011-D1RA00914A-s1387.png]

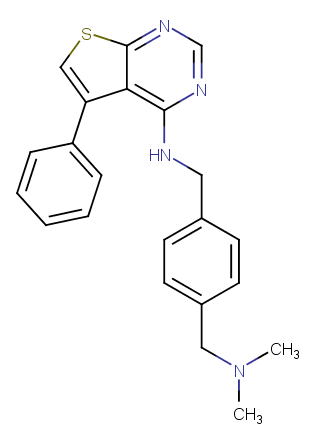

Supplement: RA-011-D1RA00914A-s1388 [file RA-011-D1RA00914A-s1388.png]

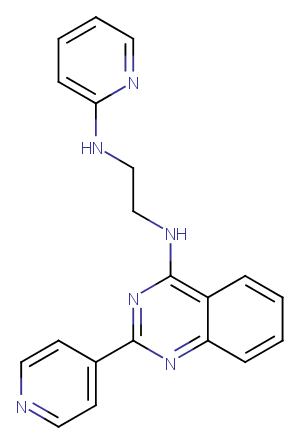

Supplement: RA-011-D1RA00914A-s1389 [file RA-011-D1RA00914A-s1389.png]

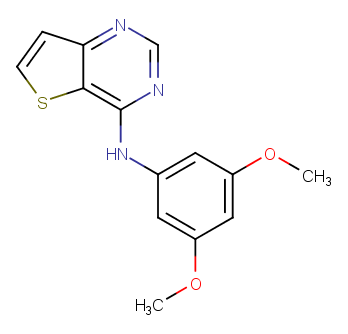

Supplement: RA-011-D1RA00914A-s1390 [file RA-011-D1RA00914A-s1390.png]

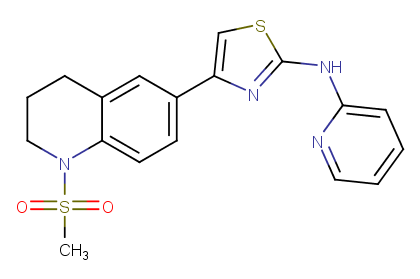

Supplement: RA-011-D1RA00914A-s1391 [file RA-011-D1RA00914A-s1391.png]

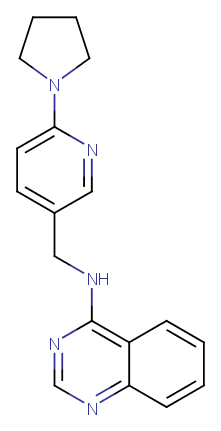

Supplement: RA-011-D1RA00914A-s1392 [file RA-011-D1RA00914A-s1392.png]

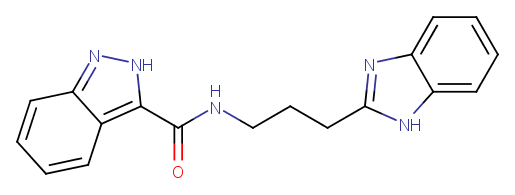

Supplement: RA-011-D1RA00914A-s1393 [file RA-011-D1RA00914A-s1393.png]

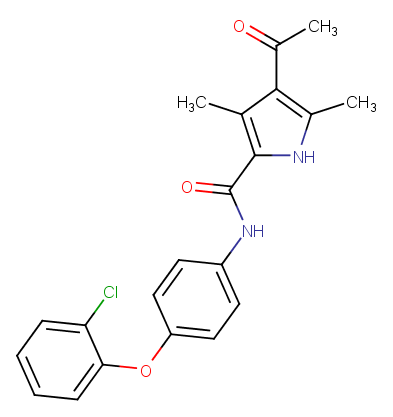

Supplement: RA-011-D1RA00914A-s1394 [file RA-011-D1RA00914A-s1394.png]

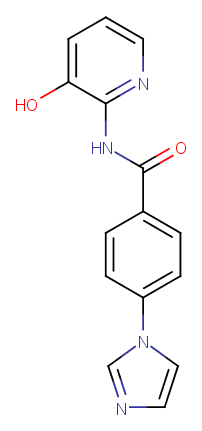

Supplement: RA-011-D1RA00914A-s1395 [file RA-011-D1RA00914A-s1395.png]

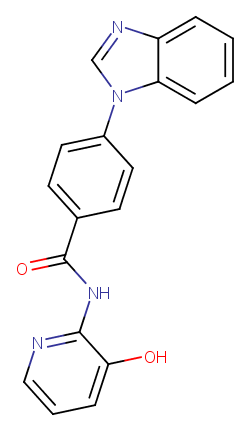

Supplement: RA-011-D1RA00914A-s1396 [file RA-011-D1RA00914A-s1396.png]

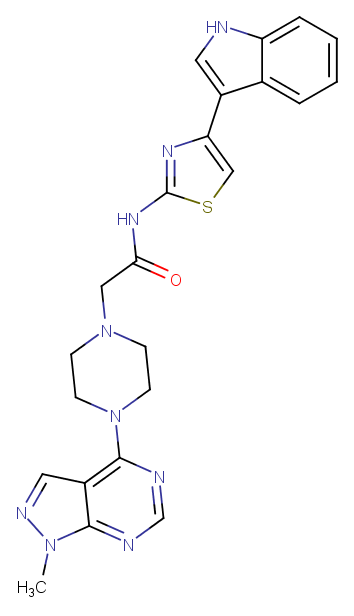

Supplement: RA-011-D1RA00914A-s1397 [file RA-011-D1RA00914A-s1397.png]

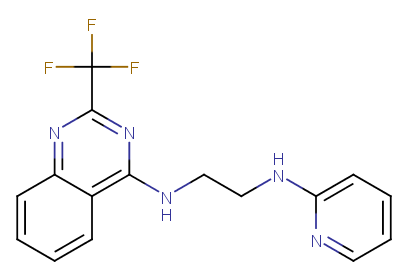

Supplement: RA-011-D1RA00914A-s1398 [file RA-011-D1RA00914A-s1398.png]

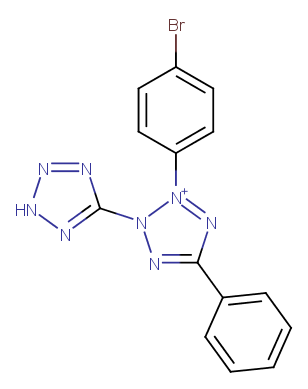

Supplement: RA-011-D1RA00914A-s1399 [file RA-011-D1RA00914A-s1399.png]

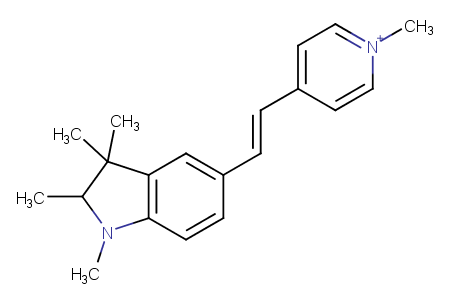

Supplement: RA-011-D1RA00914A-s1400 [file RA-011-D1RA00914A-s1400.png]

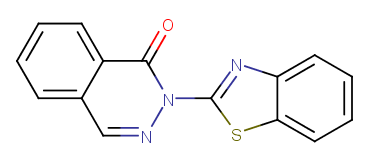

Supplement: RA-011-D1RA00914A-s1401 [file RA-011-D1RA00914A-s1401.png]

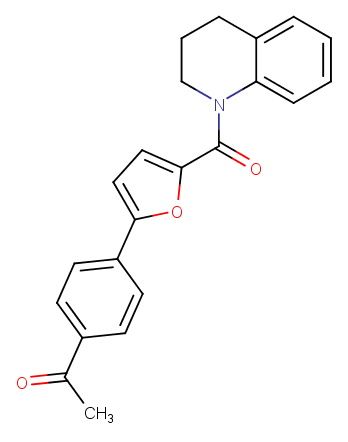

Supplement: RA-011-D1RA00914A-s1402 [file RA-011-D1RA00914A-s1402.png]

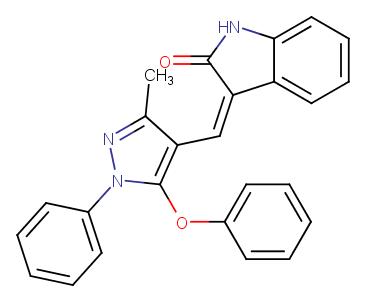

Supplement: RA-011-D1RA00914A-s1403 [file RA-011-D1RA00914A-s1403.png]

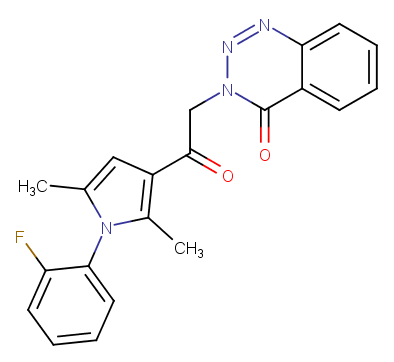

Supplement: RA-011-D1RA00914A-s1404 [file RA-011-D1RA00914A-s1404.png]

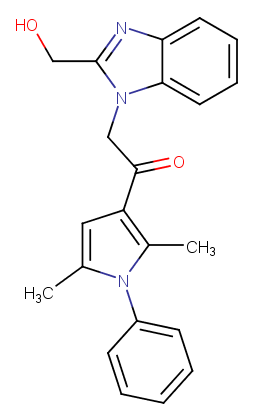

Supplement: RA-011-D1RA00914A-s1405 [file RA-011-D1RA00914A-s1405.png]

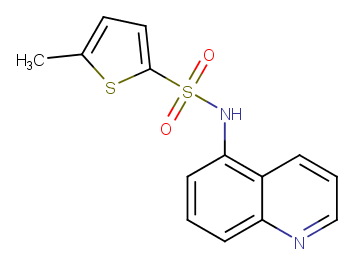

Supplement: RA-011-D1RA00914A-s1406 [file RA-011-D1RA00914A-s1406.png]

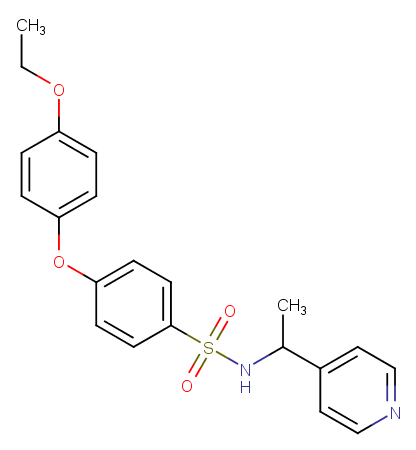

Supplement: RA-011-D1RA00914A-s1407 [file RA-011-D1RA00914A-s1407.png]

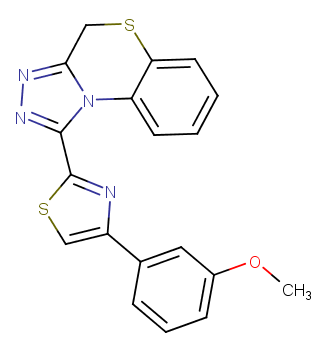

Supplement: RA-011-D1RA00914A-s1408 [file RA-011-D1RA00914A-s1408.png]

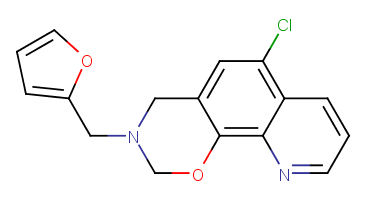

Supplement: RA-011-D1RA00914A-s1409 [file RA-011-D1RA00914A-s1409.png]

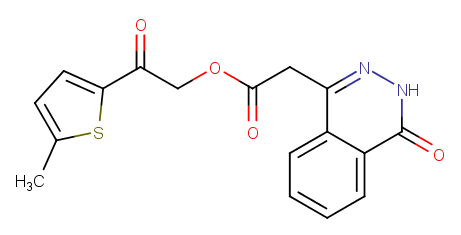

Supplement: RA-011-D1RA00914A-s1410 [file RA-011-D1RA00914A-s1410.png]

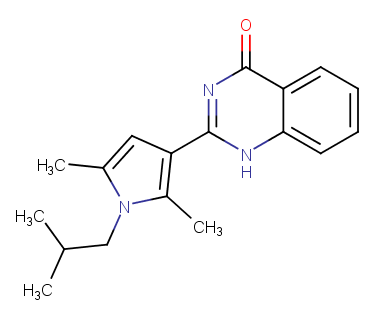

Supplement: RA-011-D1RA00914A-s1411 [file RA-011-D1RA00914A-s1411.png]

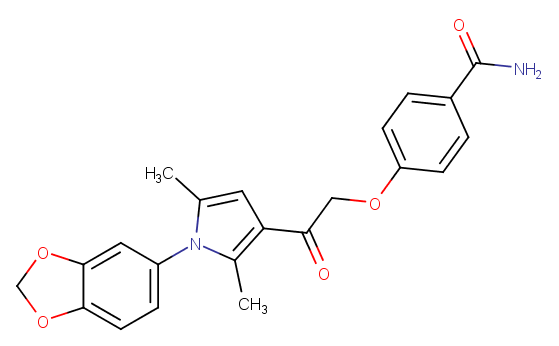

Supplement: RA-011-D1RA00914A-s1412 [file RA-011-D1RA00914A-s1412.png]

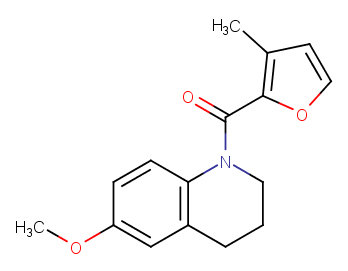

Supplement: RA-011-D1RA00914A-s1413 [file RA-011-D1RA00914A-s1413.png]

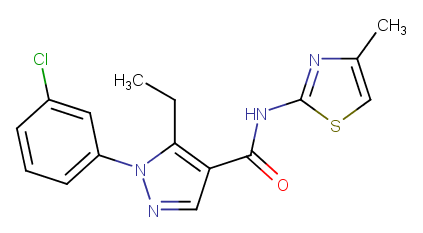

Supplement: RA-011-D1RA00914A-s1414 [file RA-011-D1RA00914A-s1414.png]

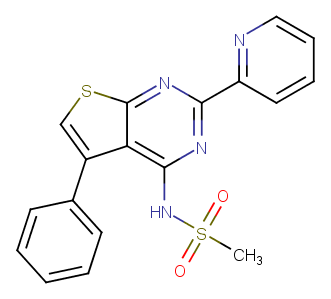

Supplement: RA-011-D1RA00914A-s1415 [file RA-011-D1RA00914A-s1415.png]

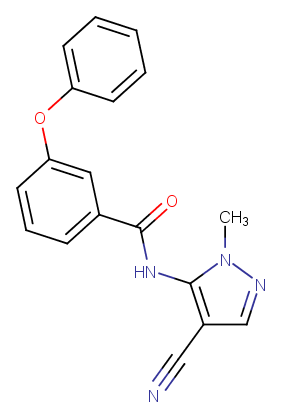

Supplement: RA-011-D1RA00914A-s1416 [file RA-011-D1RA00914A-s1416.png]

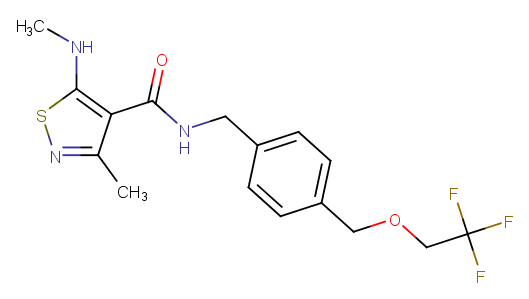

Supplement: RA-011-D1RA00914A-s1417 [file RA-011-D1RA00914A-s1417.png]

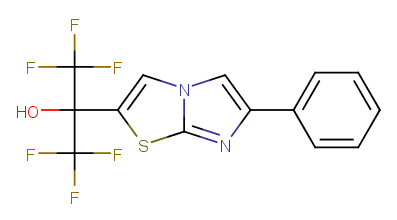

Supplement: RA-011-D1RA00914A-s1418 [file RA-011-D1RA00914A-s1418.png]

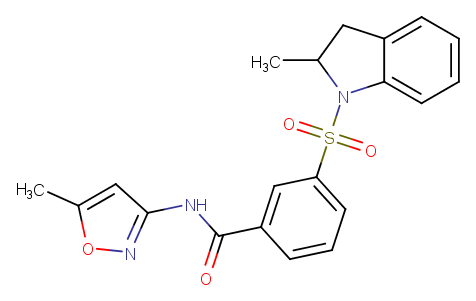

Supplement: RA-011-D1RA00914A-s1419 [file RA-011-D1RA00914A-s1419.png]

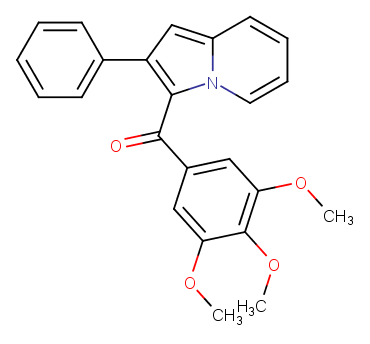

Supplement: RA-011-D1RA00914A-s1420 [file RA-011-D1RA00914A-s1420.png]

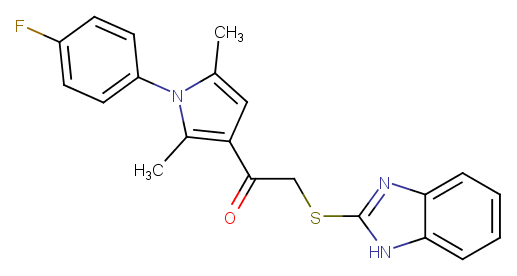

Supplement: RA-011-D1RA00914A-s1421 [file RA-011-D1RA00914A-s1421.png]

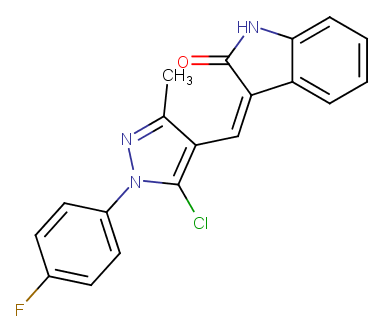

Supplement: RA-011-D1RA00914A-s1422 [file RA-011-D1RA00914A-s1422.png]

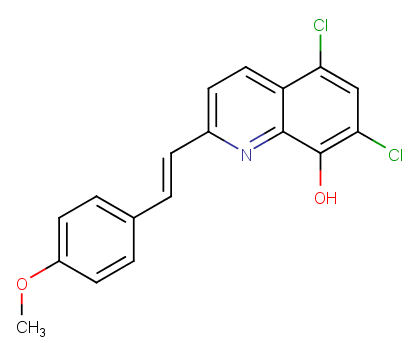

Supplement: RA-011-D1RA00914A-s1423 [file RA-011-D1RA00914A-s1423.png]

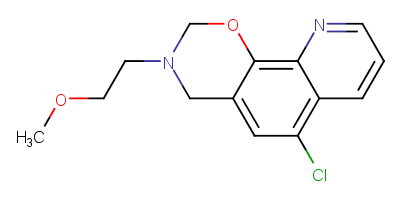

Supplement: RA-011-D1RA00914A-s1424 [file RA-011-D1RA00914A-s1424.png]

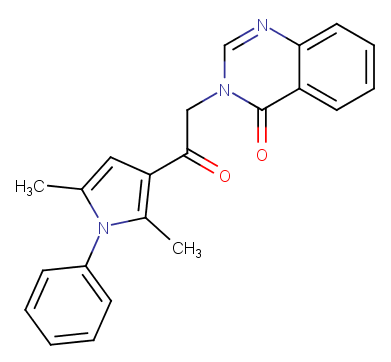

Supplement: RA-011-D1RA00914A-s1425 [file RA-011-D1RA00914A-s1425.png]

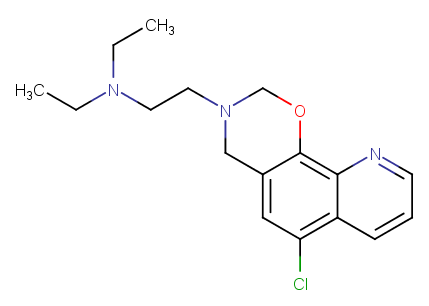

Supplement: RA-011-D1RA00914A-s1426 [file RA-011-D1RA00914A-s1426.png]

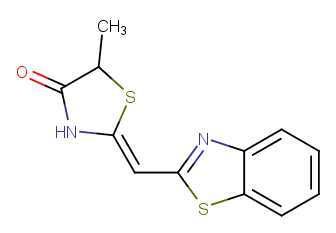

Supplement: RA-011-D1RA00914A-s1427 [file RA-011-D1RA00914A-s1427.png]

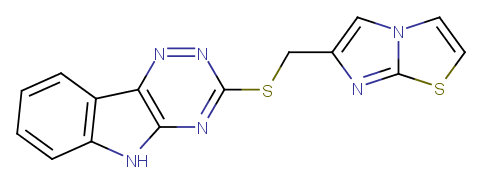

Supplement: RA-011-D1RA00914A-s1428 [file RA-011-D1RA00914A-s1428.png]

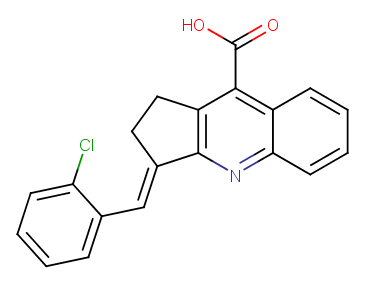

Supplement: RA-011-D1RA00914A-s1429 [file RA-011-D1RA00914A-s1429.png]

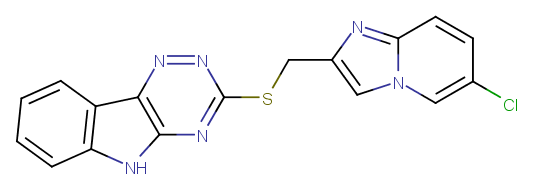

Supplement: RA-011-D1RA00914A-s1430 [file RA-011-D1RA00914A-s1430.png]

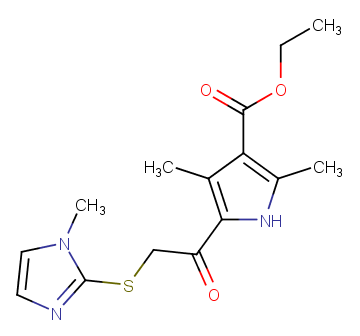

Supplement: RA-011-D1RA00914A-s1431 [file RA-011-D1RA00914A-s1431.png]

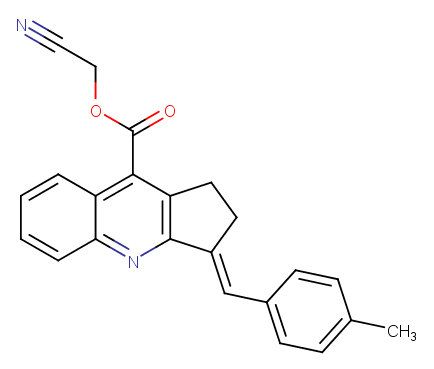

Supplement: RA-011-D1RA00914A-s1432 [file RA-011-D1RA00914A-s1432.png]

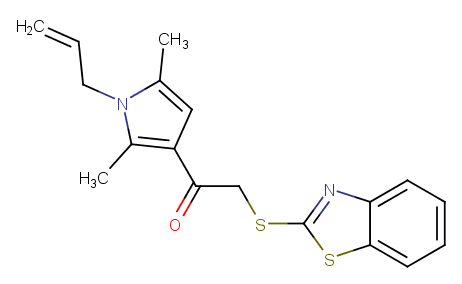

Supplement: RA-011-D1RA00914A-s1433 [file RA-011-D1RA00914A-s1433.png]

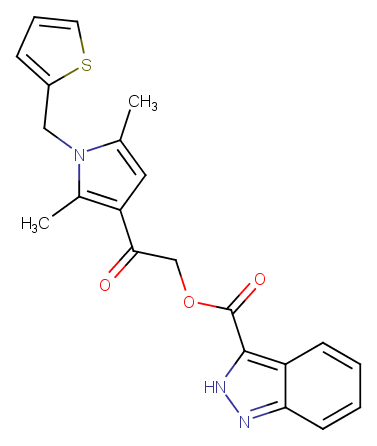

Supplement: RA-011-D1RA00914A-s1434 [file RA-011-D1RA00914A-s1434.png]

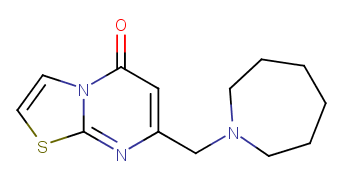

Supplement: RA-011-D1RA00914A-s1435 [file RA-011-D1RA00914A-s1435.png]

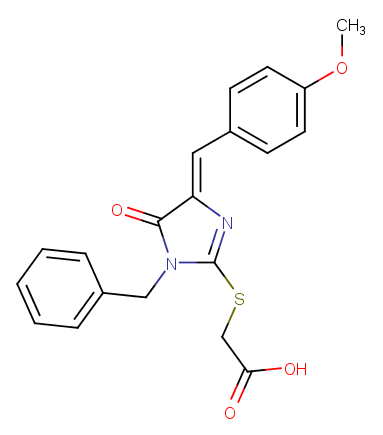

Supplement: RA-011-D1RA00914A-s1436 [file RA-011-D1RA00914A-s1436.png]

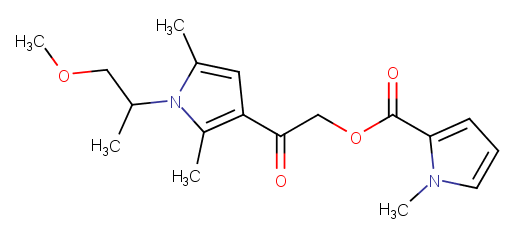

Supplement: RA-011-D1RA00914A-s1437 [file RA-011-D1RA00914A-s1437.png]

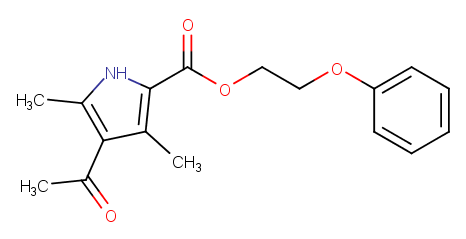

Supplement: RA-011-D1RA00914A-s1438 [file RA-011-D1RA00914A-s1438.png]

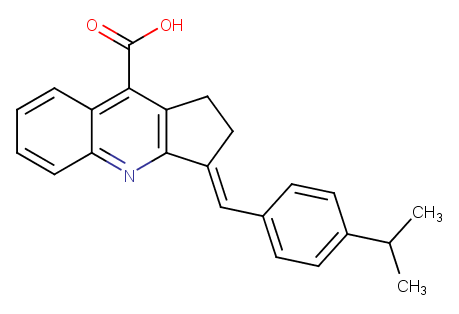

Supplement: RA-011-D1RA00914A-s1439 [file RA-011-D1RA00914A-s1439.png]

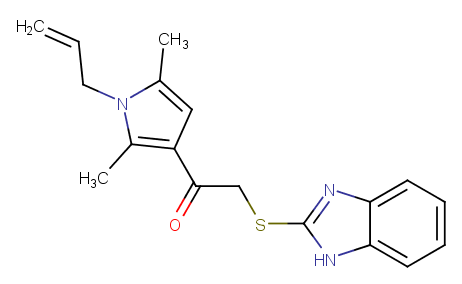

Supplement: RA-011-D1RA00914A-s1440 [file RA-011-D1RA00914A-s1440.png]

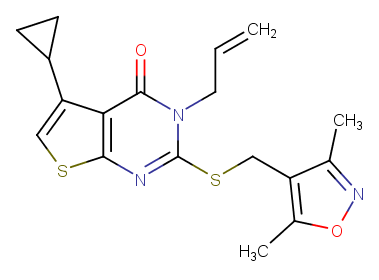

Supplement: RA-011-D1RA00914A-s1441 [file RA-011-D1RA00914A-s1441.png]

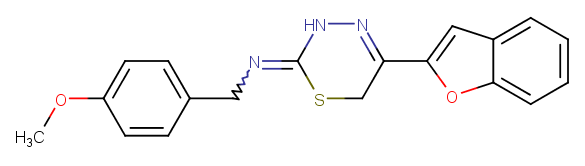

Supplement: RA-011-D1RA00914A-s1442 [file RA-011-D1RA00914A-s1442.png]

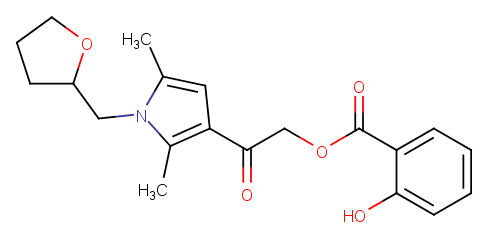

Supplement: RA-011-D1RA00914A-s1443 [file RA-011-D1RA00914A-s1443.png]

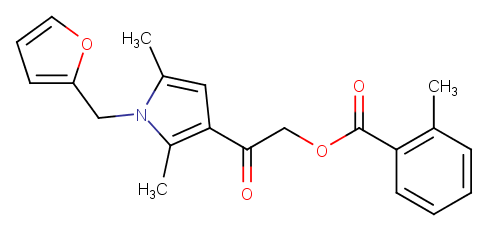

Supplement: RA-011-D1RA00914A-s1444 [file RA-011-D1RA00914A-s1444.png]

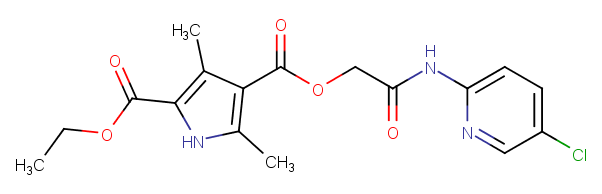

Supplement: RA-011-D1RA00914A-s1445 [file RA-011-D1RA00914A-s1445.png]

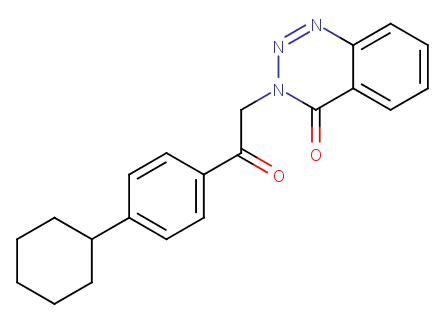

Supplement: RA-011-D1RA00914A-s1446 [file RA-011-D1RA00914A-s1446.png]

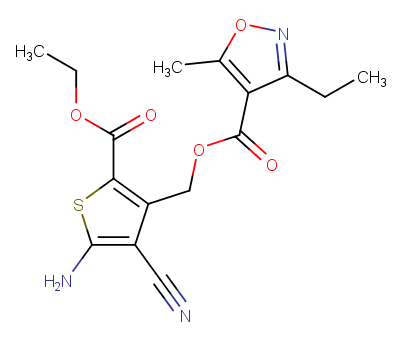

Supplement: RA-011-D1RA00914A-s1447 [file RA-011-D1RA00914A-s1447.png]

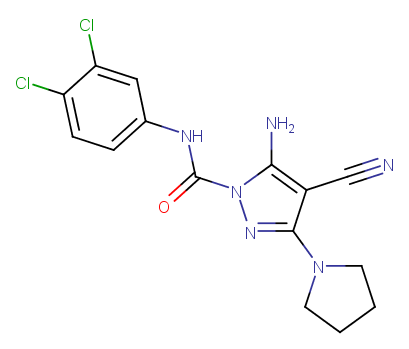

Supplement: RA-011-D1RA00914A-s1448 [file RA-011-D1RA00914A-s1448.png]

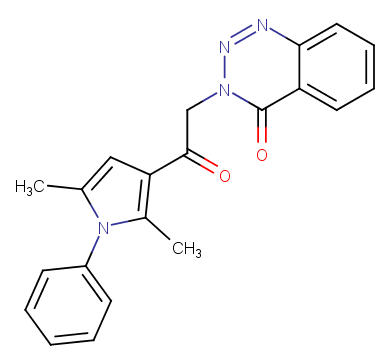

Supplement: RA-011-D1RA00914A-s1449 [file RA-011-D1RA00914A-s1449.png]

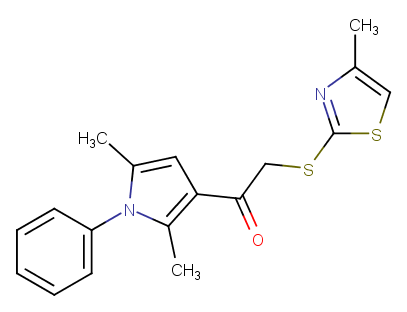

Supplement: RA-011-D1RA00914A-s1450 [file RA-011-D1RA00914A-s1450.png]

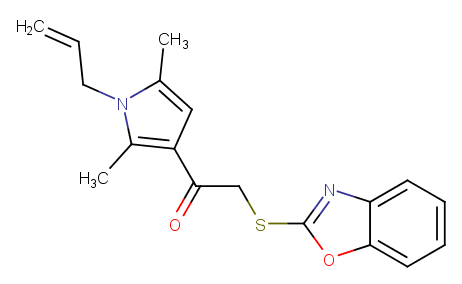

Supplement: RA-011-D1RA00914A-s1451 [file RA-011-D1RA00914A-s1451.png]

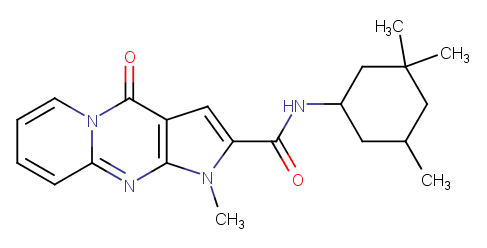

Supplement: RA-011-D1RA00914A-s1452 [file RA-011-D1RA00914A-s1452.png]

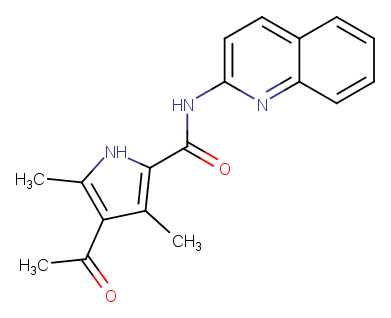

Supplement: RA-011-D1RA00914A-s1453 [file RA-011-D1RA00914A-s1453.png]

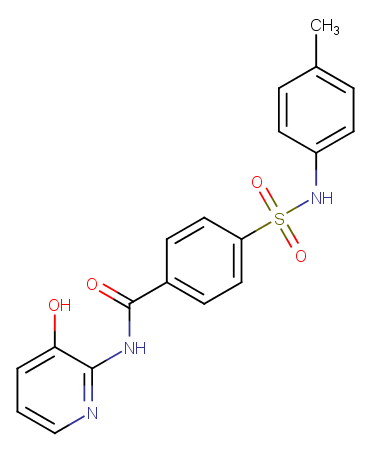

Supplement: RA-011-D1RA00914A-s1454 [file RA-011-D1RA00914A-s1454.png]

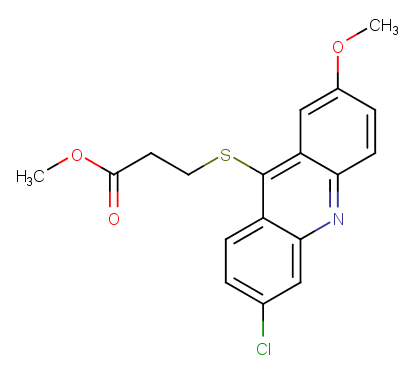

Supplement: RA-011-D1RA00914A-s1455 [file RA-011-D1RA00914A-s1455.png]

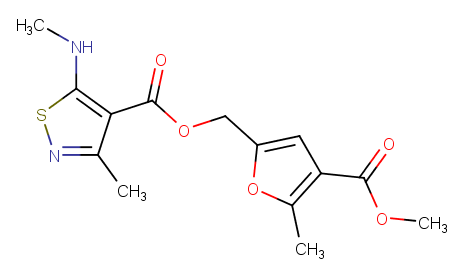

Supplement: RA-011-D1RA00914A-s1456 [file RA-011-D1RA00914A-s1456.png]

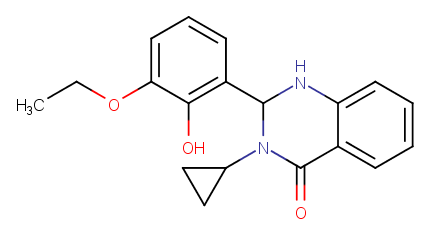

Supplement: RA-011-D1RA00914A-s1457 [file RA-011-D1RA00914A-s1457.png]

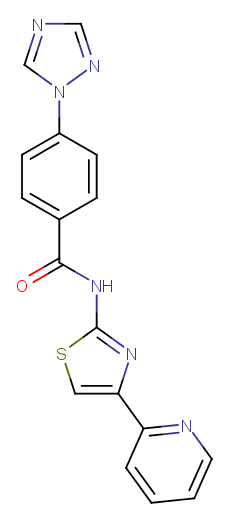

Supplement: RA-011-D1RA00914A-s1458 [file RA-011-D1RA00914A-s1458.png]

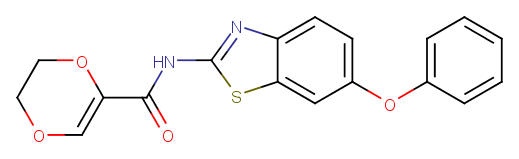

Supplement: RA-011-D1RA00914A-s1459 [file RA-011-D1RA00914A-s1459.png]

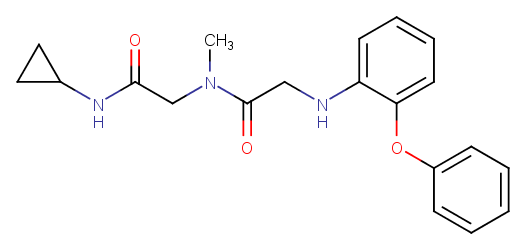

Supplement: RA-011-D1RA00914A-s1460 [file RA-011-D1RA00914A-s1460.png]

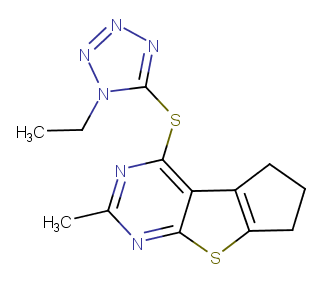

Supplement: RA-011-D1RA00914A-s1461 [file RA-011-D1RA00914A-s1461.png]

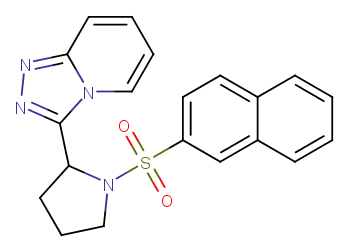

Supplement: RA-011-D1RA00914A-s1462 [file RA-011-D1RA00914A-s1462.png]

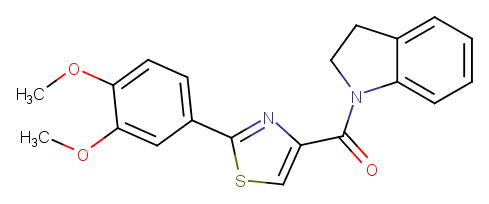

Supplement: RA-011-D1RA00914A-s1463 [file RA-011-D1RA00914A-s1463.png]

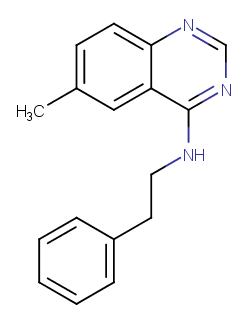

Supplement: RA-011-D1RA00914A-s1464 [file RA-011-D1RA00914A-s1464.png]

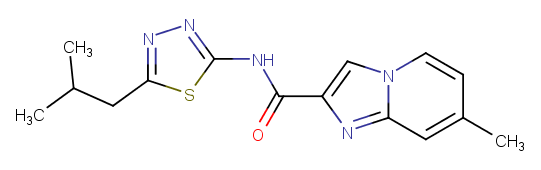

Supplement: RA-011-D1RA00914A-s1465 [file RA-011-D1RA00914A-s1465.png]

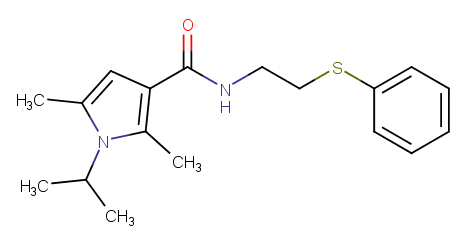

Supplement: RA-011-D1RA00914A-s1466 [file RA-011-D1RA00914A-s1466.png]

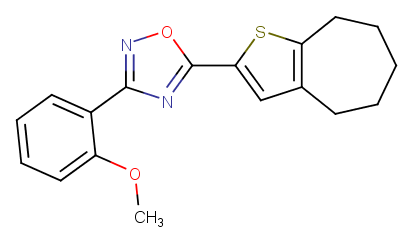

Supplement: RA-011-D1RA00914A-s1467 [file RA-011-D1RA00914A-s1467.png]

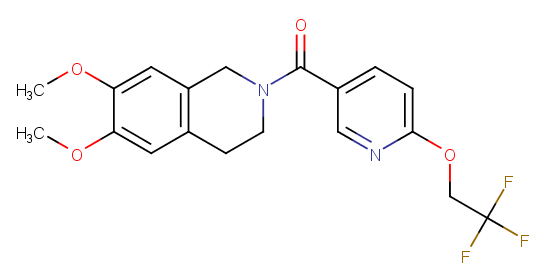

Supplement: RA-011-D1RA00914A-s1468 [file RA-011-D1RA00914A-s1468.png]

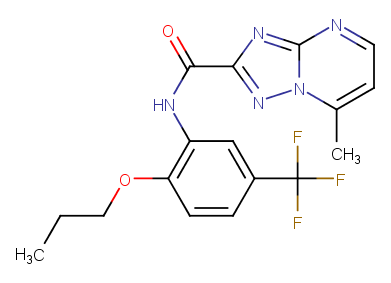

Supplement: RA-011-D1RA00914A-s1469 [file RA-011-D1RA00914A-s1469.png]

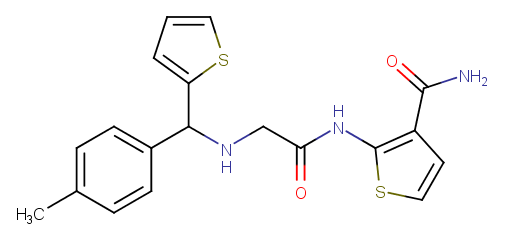

Supplement: RA-011-D1RA00914A-s1470 [file RA-011-D1RA00914A-s1470.png]

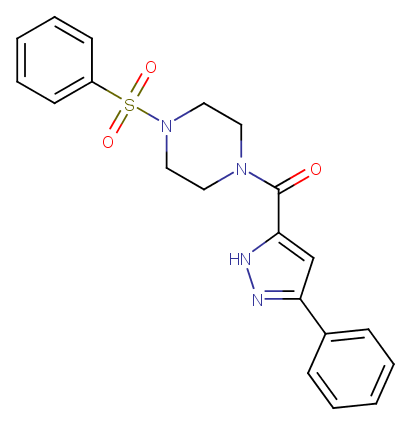

Supplement: RA-011-D1RA00914A-s1471 [file RA-011-D1RA00914A-s1471.png]

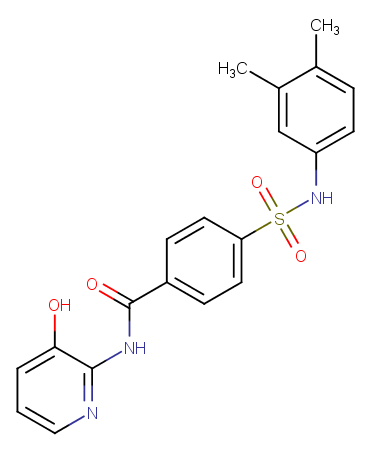

Supplement: RA-011-D1RA00914A-s1472 [file RA-011-D1RA00914A-s1472.png]

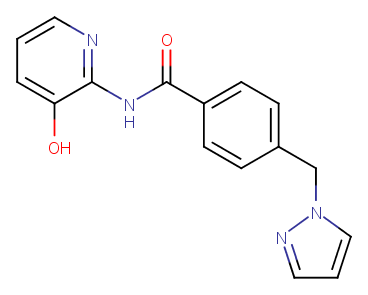

Supplement: RA-011-D1RA00914A-s1473 [file RA-011-D1RA00914A-s1473.png]
