# Supplementary figures and images for: Improved herbicide discovery using physico-chemical rules refined by antimalarial library screening (part 12 of 14)
Source: RSC Adv. 2021 Feb 23;11(15):8459–67. doi: 10.1039/d1ra00914a (PMC8695207; doi:10.1039/d1ra00914a)

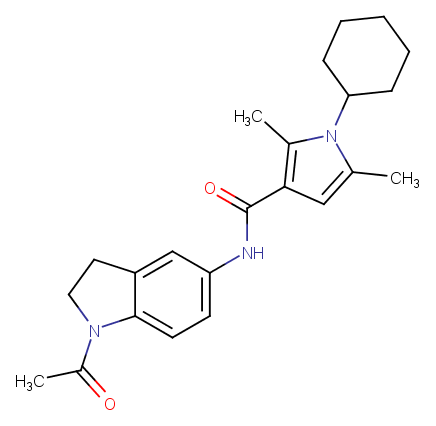

Supplement: RA-011-D1RA00914A-s1474 [file RA-011-D1RA00914A-s1474.png]

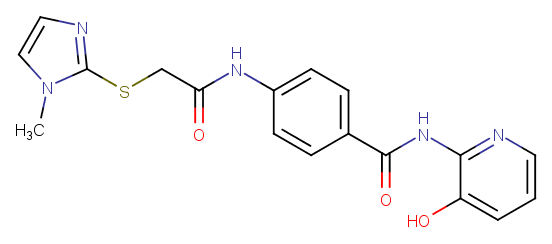

Supplement: RA-011-D1RA00914A-s1475 [file RA-011-D1RA00914A-s1475.png]

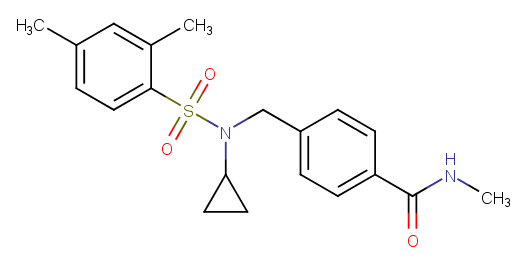

Supplement: RA-011-D1RA00914A-s1476 [file RA-011-D1RA00914A-s1476.png]

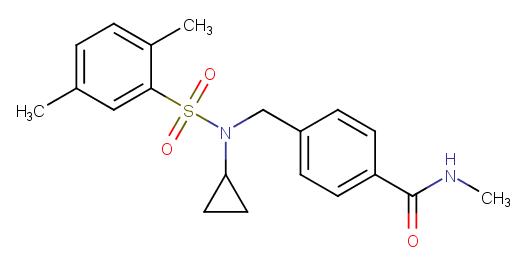

Supplement: RA-011-D1RA00914A-s1477 [file RA-011-D1RA00914A-s1477.png]

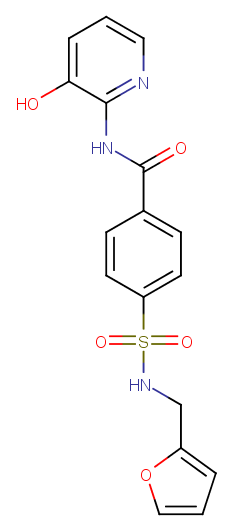

Supplement: RA-011-D1RA00914A-s1478 [file RA-011-D1RA00914A-s1478.png]

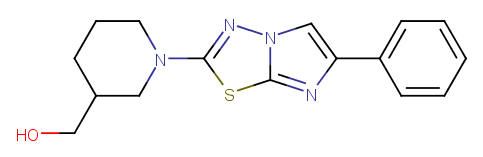

Supplement: RA-011-D1RA00914A-s1479 [file RA-011-D1RA00914A-s1479.png]

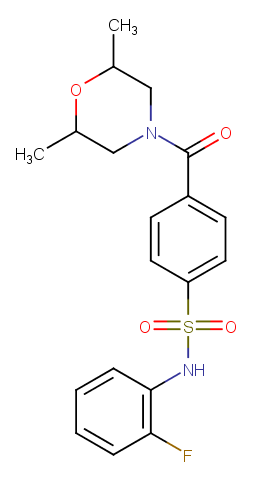

Supplement: RA-011-D1RA00914A-s1480 [file RA-011-D1RA00914A-s1480.png]

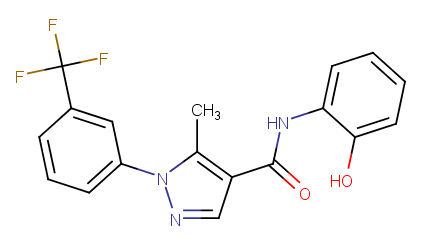

Supplement: RA-011-D1RA00914A-s1481 [file RA-011-D1RA00914A-s1481.png]

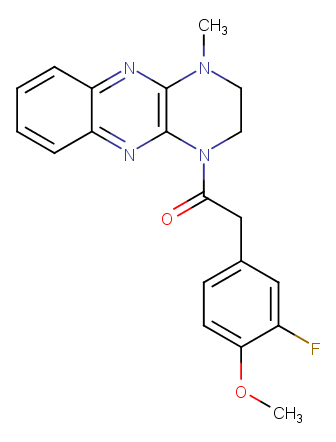

Supplement: RA-011-D1RA00914A-s1482 [file RA-011-D1RA00914A-s1482.png]

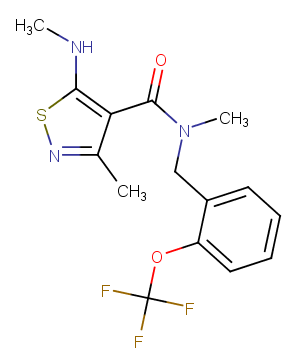

Supplement: RA-011-D1RA00914A-s1483 [file RA-011-D1RA00914A-s1483.png]

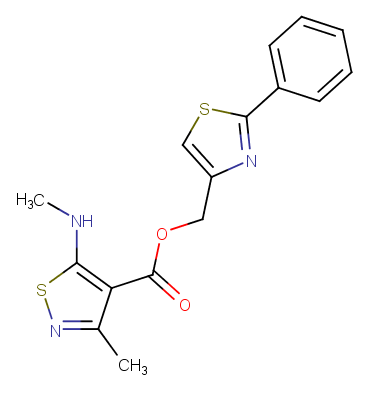

Supplement: RA-011-D1RA00914A-s1484 [file RA-011-D1RA00914A-s1484.png]

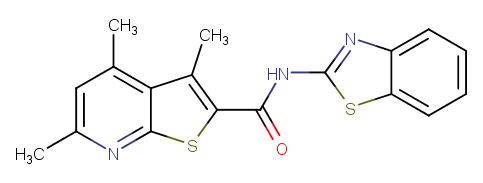

Supplement: RA-011-D1RA00914A-s1485 [file RA-011-D1RA00914A-s1485.png]

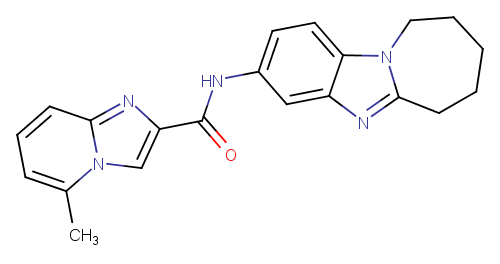

Supplement: RA-011-D1RA00914A-s1486 [file RA-011-D1RA00914A-s1486.png]

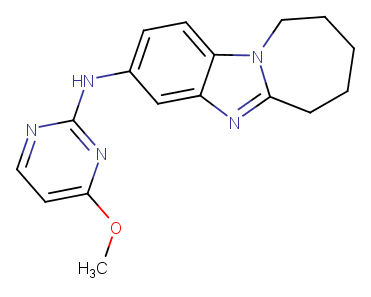

Supplement: RA-011-D1RA00914A-s1487 [file RA-011-D1RA00914A-s1487.png]

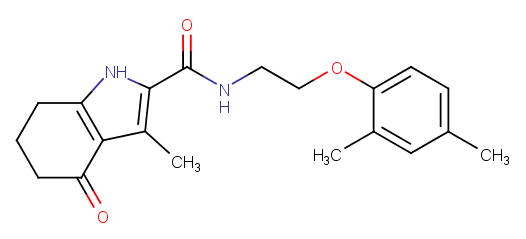

Supplement: RA-011-D1RA00914A-s1488 [file RA-011-D1RA00914A-s1488.png]

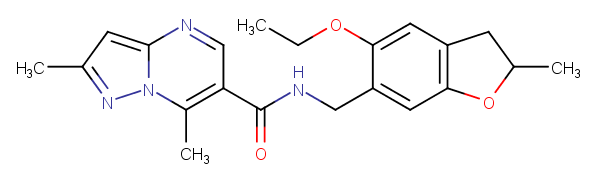

Supplement: RA-011-D1RA00914A-s1489 [file RA-011-D1RA00914A-s1489.png]

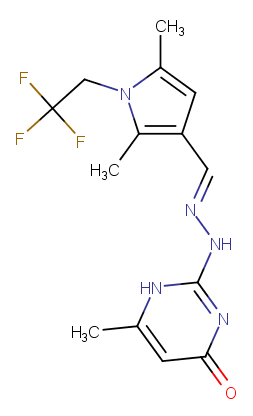

Supplement: RA-011-D1RA00914A-s1490 [file RA-011-D1RA00914A-s1490.png]

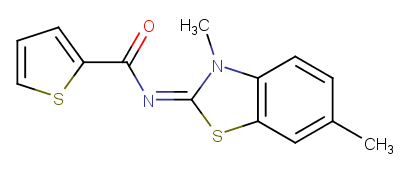

Supplement: RA-011-D1RA00914A-s1491 [file RA-011-D1RA00914A-s1491.png]

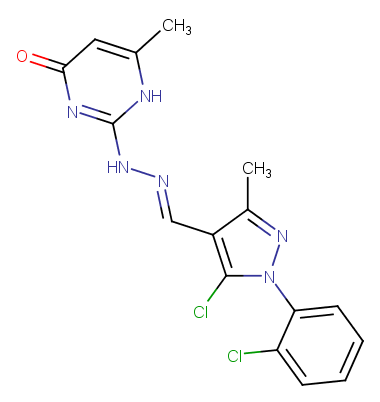

Supplement: RA-011-D1RA00914A-s1492 [file RA-011-D1RA00914A-s1492.png]

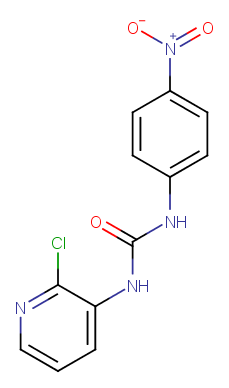

Supplement: RA-011-D1RA00914A-s1493 [file RA-011-D1RA00914A-s1493.png]

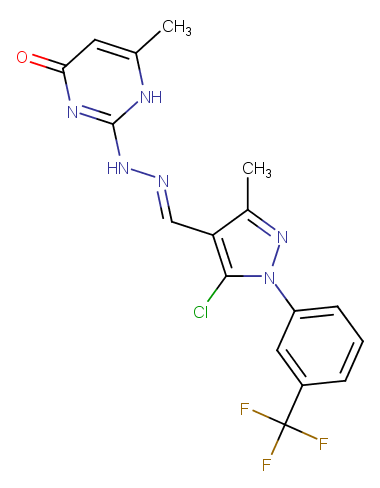

Supplement: RA-011-D1RA00914A-s1494 [file RA-011-D1RA00914A-s1494.png]

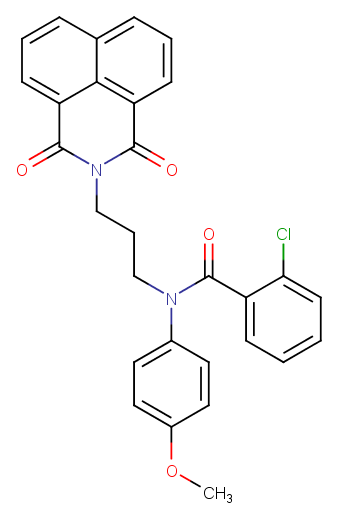

Supplement: RA-011-D1RA00914A-s1495 [file RA-011-D1RA00914A-s1495.png]

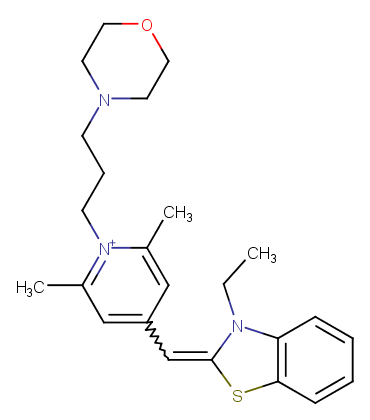

Supplement: RA-011-D1RA00914A-s1496 [file RA-011-D1RA00914A-s1496.png]

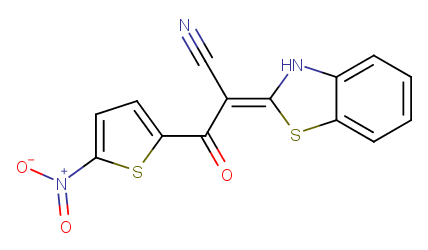

Supplement: RA-011-D1RA00914A-s1497 [file RA-011-D1RA00914A-s1497.png]

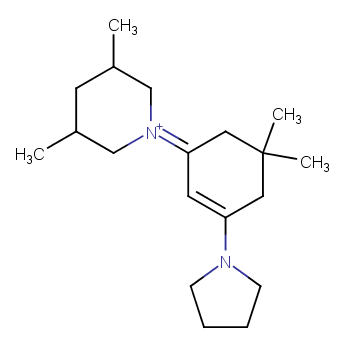

Supplement: RA-011-D1RA00914A-s1498 [file RA-011-D1RA00914A-s1498.png]

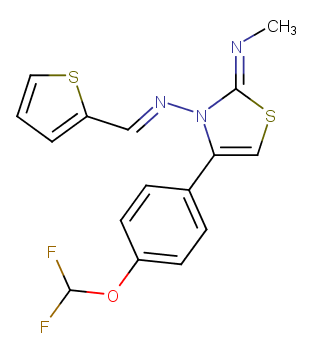

Supplement: RA-011-D1RA00914A-s1499 [file RA-011-D1RA00914A-s1499.png]

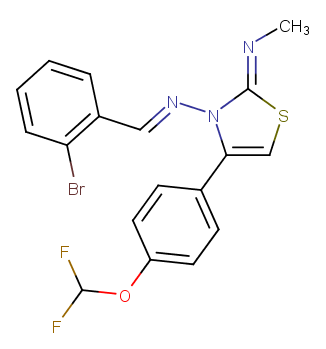

Supplement: RA-011-D1RA00914A-s1500 [file RA-011-D1RA00914A-s1500.png]

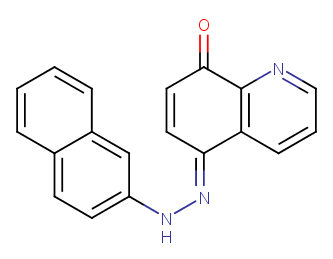

Supplement: RA-011-D1RA00914A-s1501 [file RA-011-D1RA00914A-s1501.png]

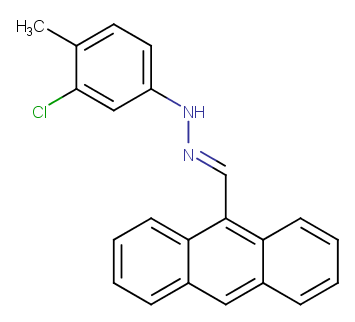

Supplement: RA-011-D1RA00914A-s1502 [file RA-011-D1RA00914A-s1502.png]

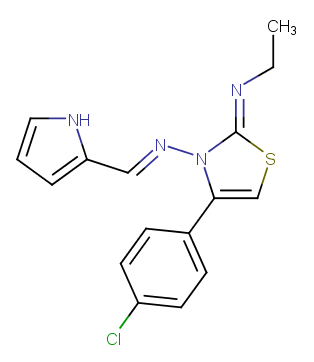

Supplement: RA-011-D1RA00914A-s1503 [file RA-011-D1RA00914A-s1503.png]

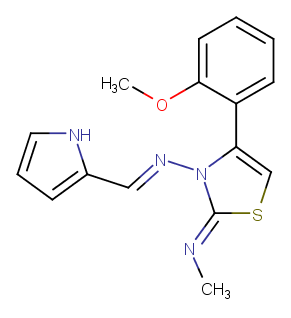

Supplement: RA-011-D1RA00914A-s1504 [file RA-011-D1RA00914A-s1504.png]

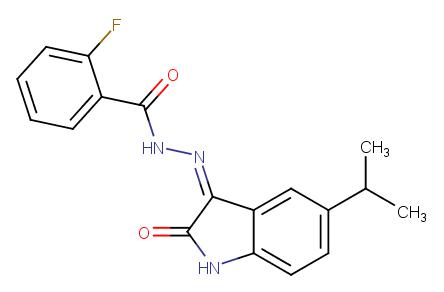

Supplement: RA-011-D1RA00914A-s1505 [file RA-011-D1RA00914A-s1505.png]

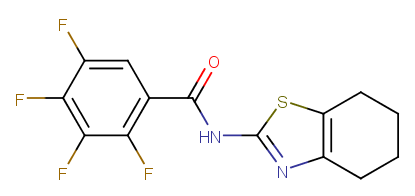

Supplement: RA-011-D1RA00914A-s1506 [file RA-011-D1RA00914A-s1506.png]

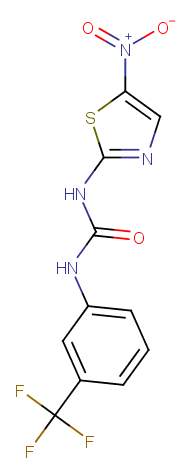

Supplement: RA-011-D1RA00914A-s1507 [file RA-011-D1RA00914A-s1507.png]

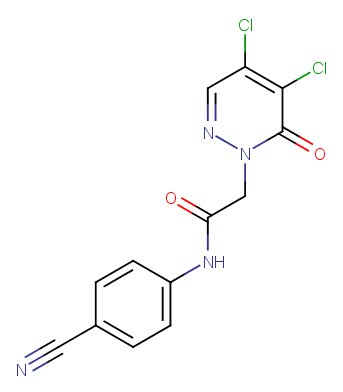

Supplement: RA-011-D1RA00914A-s1508 [file RA-011-D1RA00914A-s1508.png]

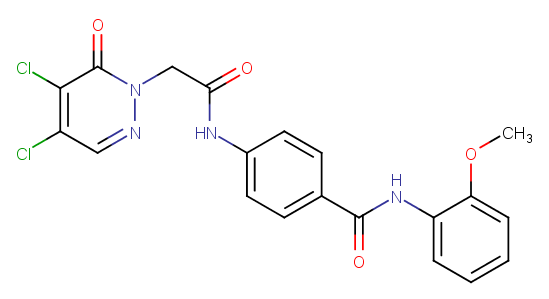

Supplement: RA-011-D1RA00914A-s1509 [file RA-011-D1RA00914A-s1509.png]

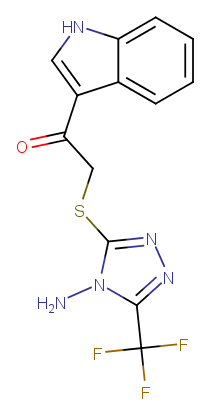

Supplement: RA-011-D1RA00914A-s1510 [file RA-011-D1RA00914A-s1510.png]

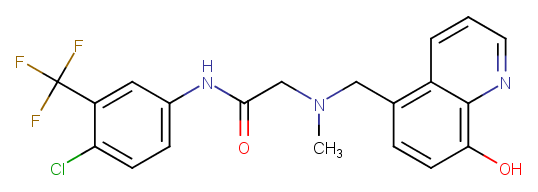

Supplement: RA-011-D1RA00914A-s1511 [file RA-011-D1RA00914A-s1511.png]

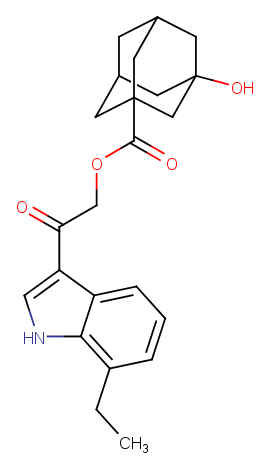

Supplement: RA-011-D1RA00914A-s1512 [file RA-011-D1RA00914A-s1512.png]

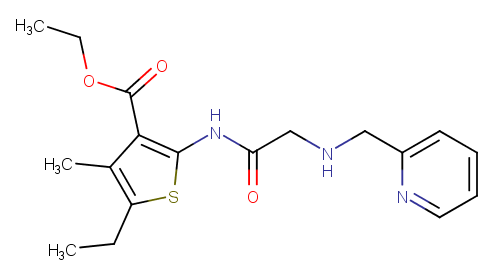

Supplement: RA-011-D1RA00914A-s1513 [file RA-011-D1RA00914A-s1513.png]

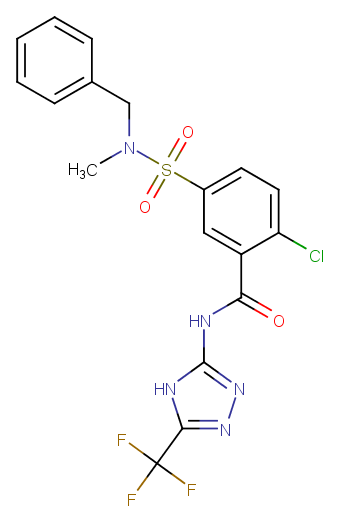

Supplement: RA-011-D1RA00914A-s1514 [file RA-011-D1RA00914A-s1514.png]

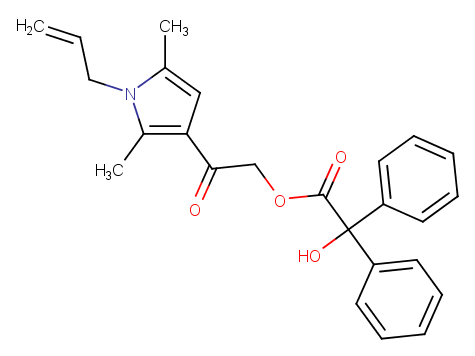

Supplement: RA-011-D1RA00914A-s1515 [file RA-011-D1RA00914A-s1515.png]

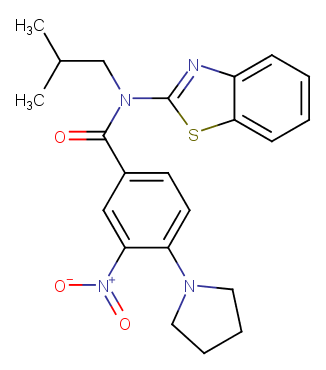

Supplement: RA-011-D1RA00914A-s1516 [file RA-011-D1RA00914A-s1516.png]

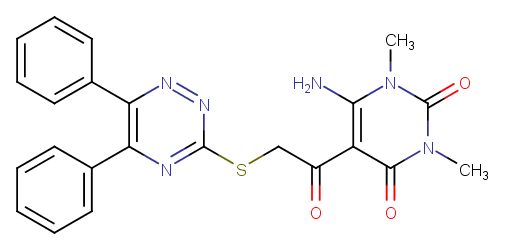

Supplement: RA-011-D1RA00914A-s1517 [file RA-011-D1RA00914A-s1517.png]

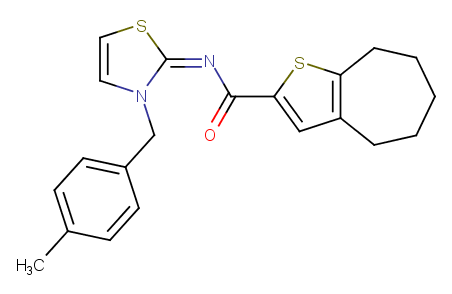

Supplement: RA-011-D1RA00914A-s1518 [file RA-011-D1RA00914A-s1518.png]

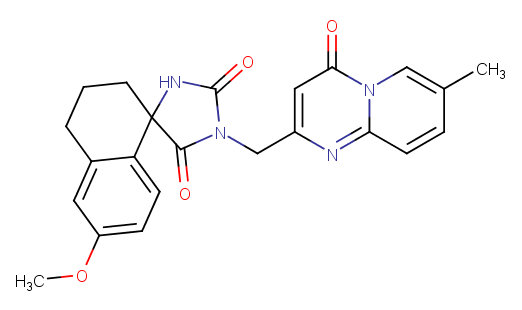

Supplement: RA-011-D1RA00914A-s1519 [file RA-011-D1RA00914A-s1519.png]

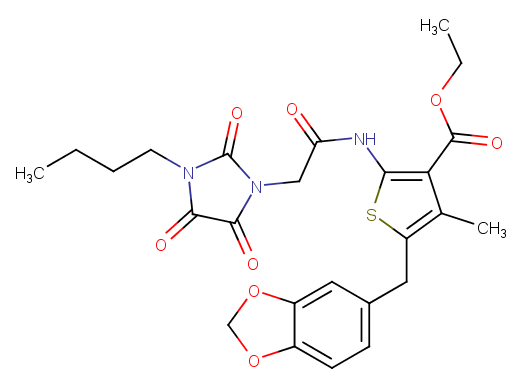

Supplement: RA-011-D1RA00914A-s1520 [file RA-011-D1RA00914A-s1520.png]

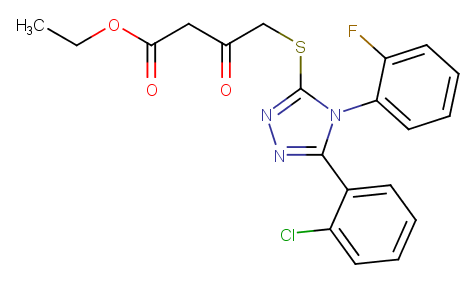

Supplement: RA-011-D1RA00914A-s1521 [file RA-011-D1RA00914A-s1521.png]

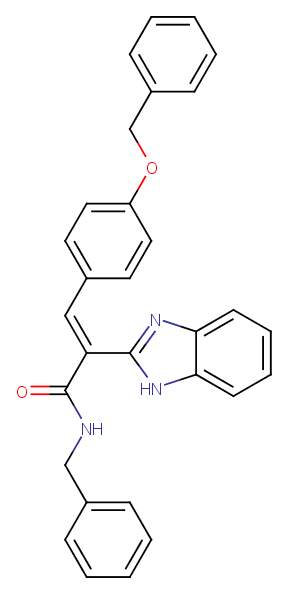

Supplement: RA-011-D1RA00914A-s1522 [file RA-011-D1RA00914A-s1522.png]

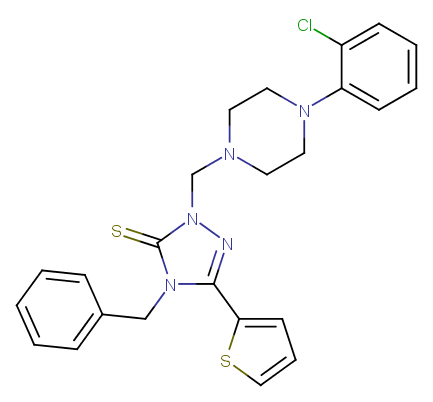

Supplement: RA-011-D1RA00914A-s1523 [file RA-011-D1RA00914A-s1523.png]

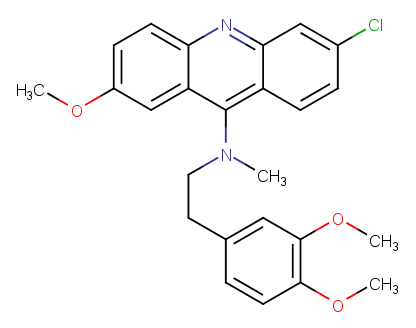

Supplement: RA-011-D1RA00914A-s1524 [file RA-011-D1RA00914A-s1524.png]

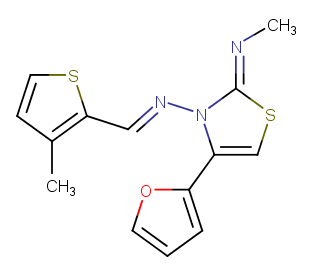

Supplement: RA-011-D1RA00914A-s1525 [file RA-011-D1RA00914A-s1525.png]

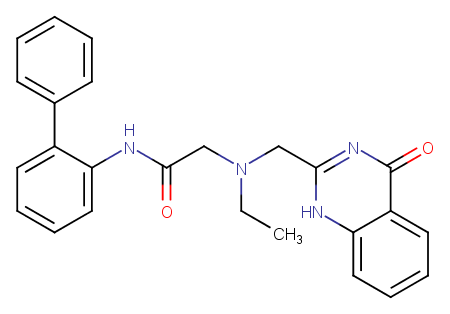

Supplement: RA-011-D1RA00914A-s1526 [file RA-011-D1RA00914A-s1526.png]

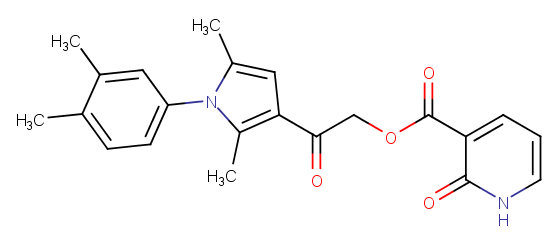

Supplement: RA-011-D1RA00914A-s1527 [file RA-011-D1RA00914A-s1527.png]

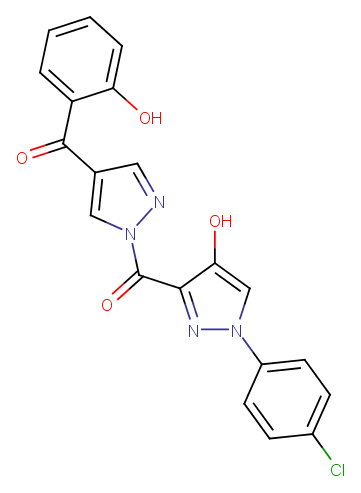

Supplement: RA-011-D1RA00914A-s1528 [file RA-011-D1RA00914A-s1528.png]

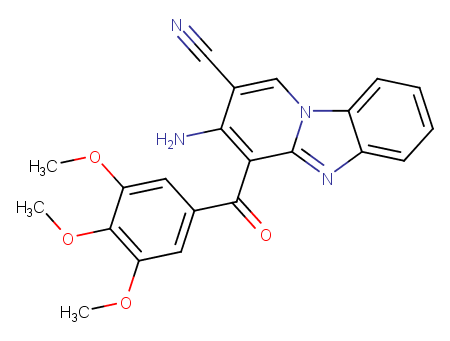

Supplement: RA-011-D1RA00914A-s1529 [file RA-011-D1RA00914A-s1529.png]

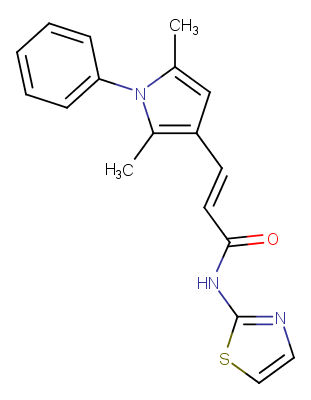

Supplement: RA-011-D1RA00914A-s1530 [file RA-011-D1RA00914A-s1530.png]

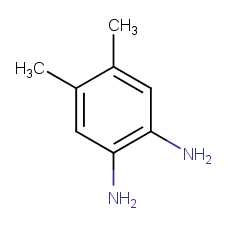

Supplement: RA-011-D1RA00914A-s1531 [file RA-011-D1RA00914A-s1531.png]

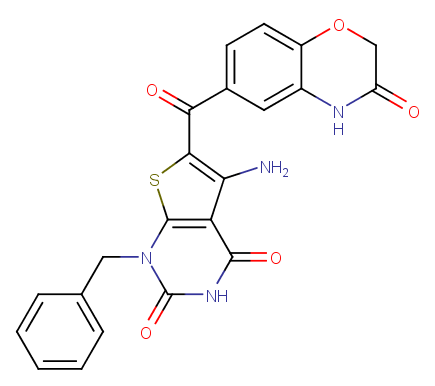

Supplement: RA-011-D1RA00914A-s1532 [file RA-011-D1RA00914A-s1532.png]

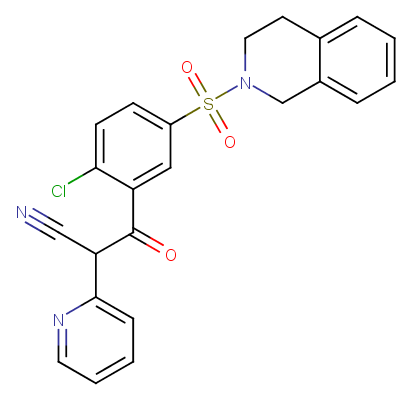

Supplement: RA-011-D1RA00914A-s1533 [file RA-011-D1RA00914A-s1533.png]

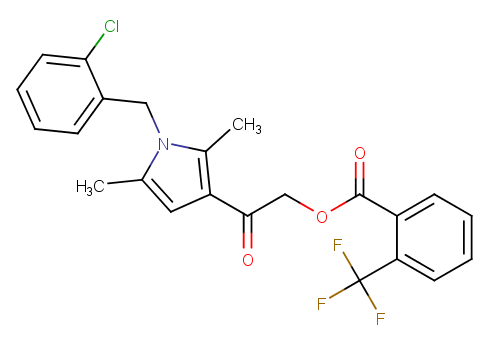

Supplement: RA-011-D1RA00914A-s1534 [file RA-011-D1RA00914A-s1534.png]

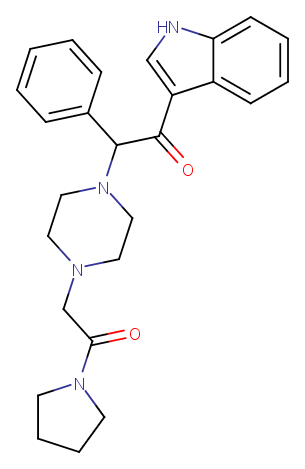

Supplement: RA-011-D1RA00914A-s1535 [file RA-011-D1RA00914A-s1535.png]

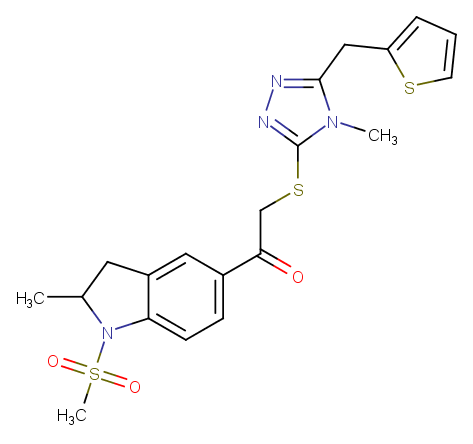

Supplement: RA-011-D1RA00914A-s1536 [file RA-011-D1RA00914A-s1536.png]

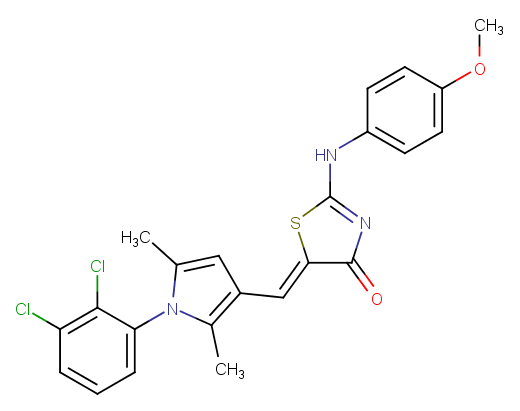

Supplement: RA-011-D1RA00914A-s1537 [file RA-011-D1RA00914A-s1537.png]

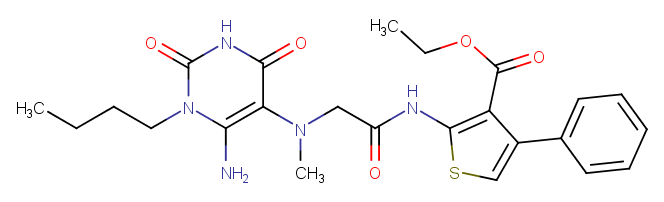

Supplement: RA-011-D1RA00914A-s1538 [file RA-011-D1RA00914A-s1538.png]

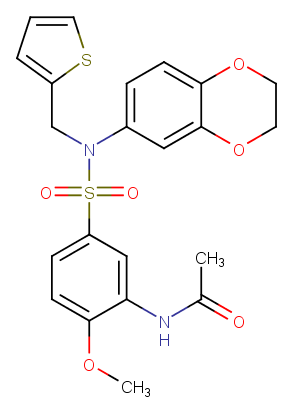

Supplement: RA-011-D1RA00914A-s1539 [file RA-011-D1RA00914A-s1539.png]

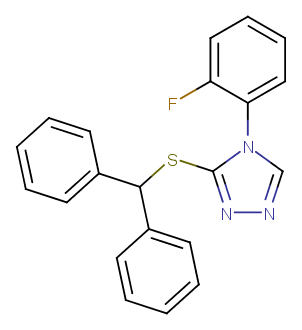

Supplement: RA-011-D1RA00914A-s1540 [file RA-011-D1RA00914A-s1540.png]

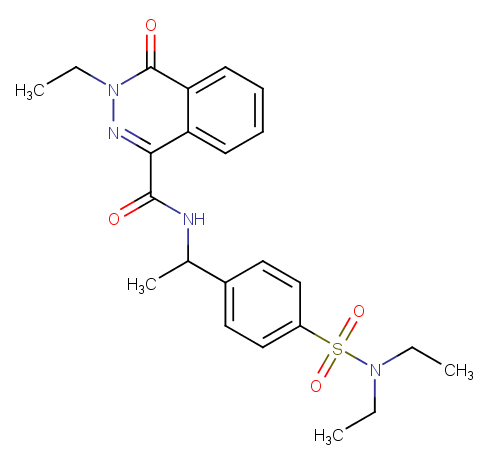

Supplement: RA-011-D1RA00914A-s1541 [file RA-011-D1RA00914A-s1541.png]

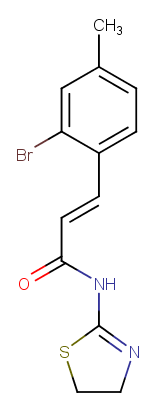

Supplement: RA-011-D1RA00914A-s1542 [file RA-011-D1RA00914A-s1542.png]

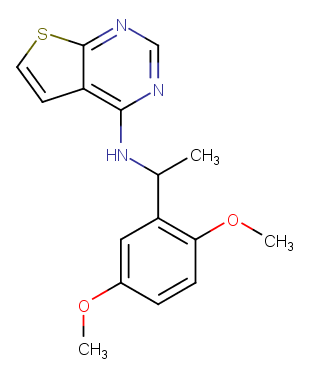

Supplement: RA-011-D1RA00914A-s1543 [file RA-011-D1RA00914A-s1543.png]

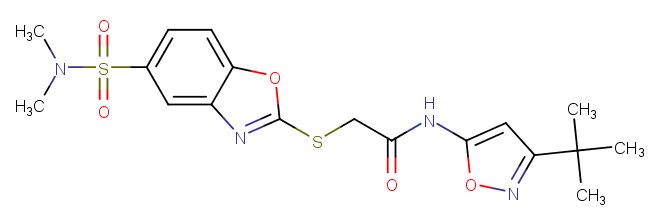

Supplement: RA-011-D1RA00914A-s1544 [file RA-011-D1RA00914A-s1544.png]

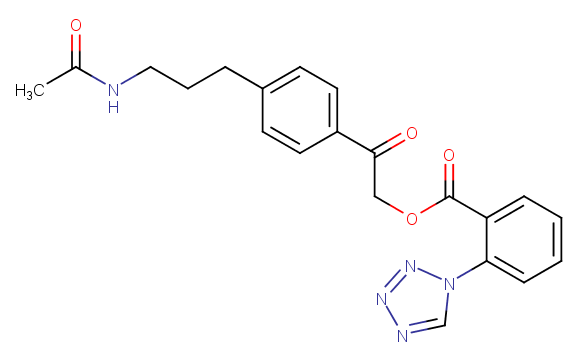

Supplement: RA-011-D1RA00914A-s1545 [file RA-011-D1RA00914A-s1545.png]

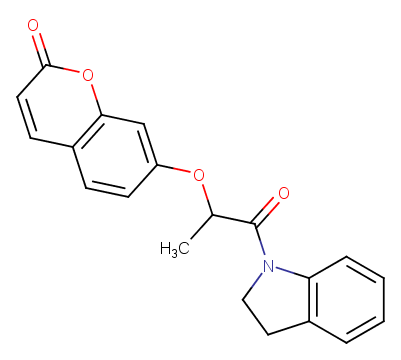

Supplement: RA-011-D1RA00914A-s1546 [file RA-011-D1RA00914A-s1546.png]

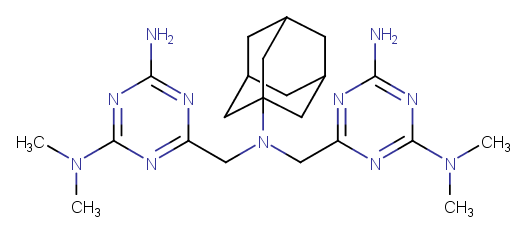

Supplement: RA-011-D1RA00914A-s1547 [file RA-011-D1RA00914A-s1547.png]

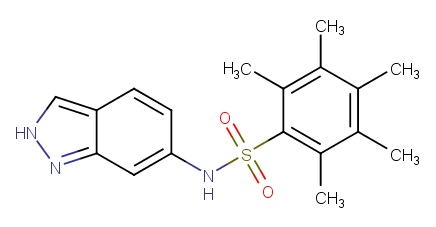

Supplement: RA-011-D1RA00914A-s1548 [file RA-011-D1RA00914A-s1548.png]

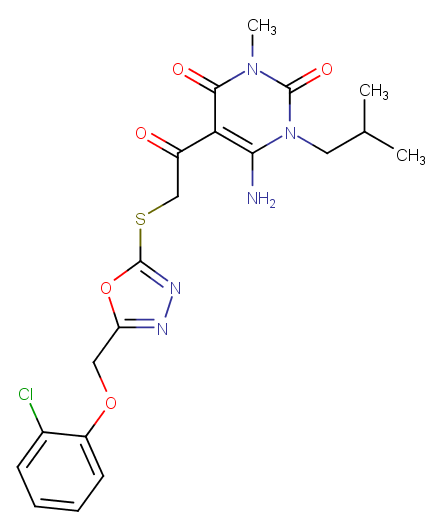

Supplement: RA-011-D1RA00914A-s1549 [file RA-011-D1RA00914A-s1549.png]

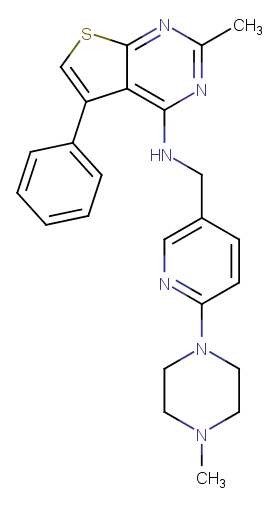

Supplement: RA-011-D1RA00914A-s1550 [file RA-011-D1RA00914A-s1550.png]

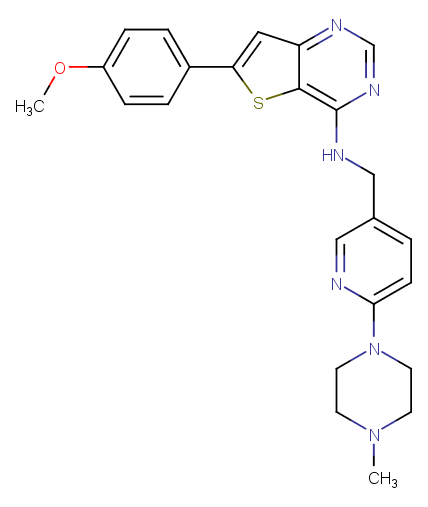

Supplement: RA-011-D1RA00914A-s1551 [file RA-011-D1RA00914A-s1551.png]

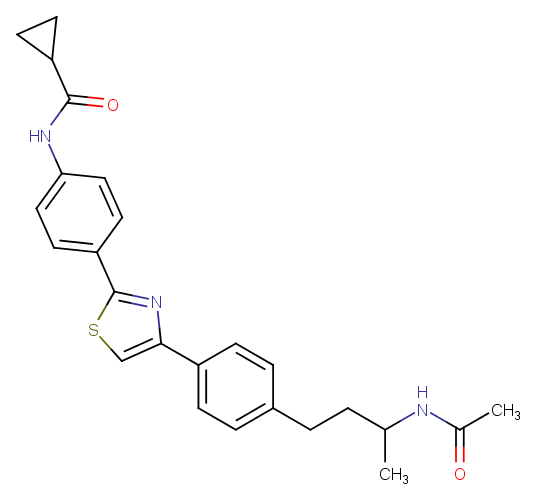

Supplement: RA-011-D1RA00914A-s1552 [file RA-011-D1RA00914A-s1552.png]

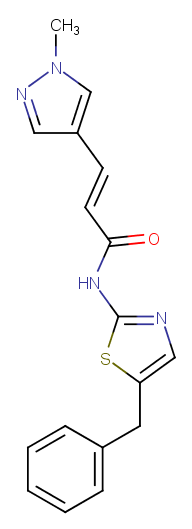

Supplement: RA-011-D1RA00914A-s1553 [file RA-011-D1RA00914A-s1553.png]

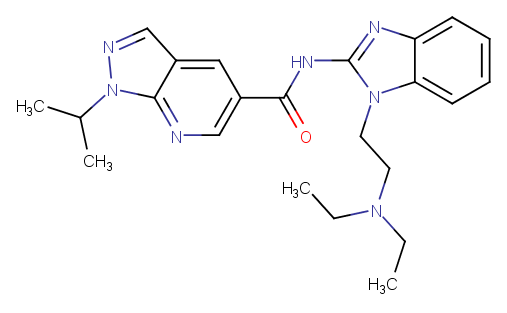

Supplement: RA-011-D1RA00914A-s1554 [file RA-011-D1RA00914A-s1554.png]

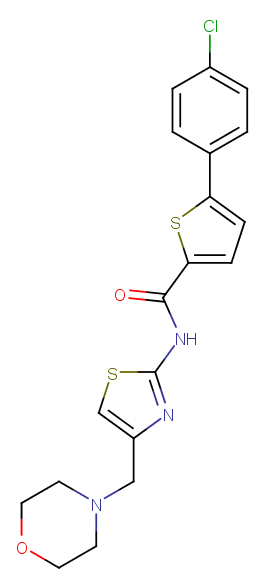

Supplement: RA-011-D1RA00914A-s1555 [file RA-011-D1RA00914A-s1555.png]

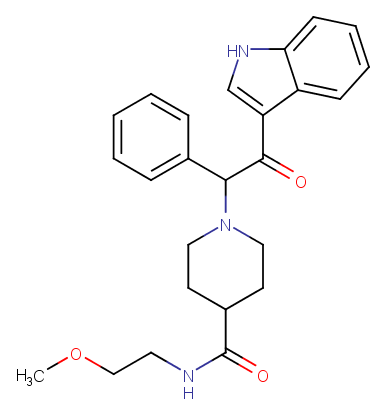

Supplement: RA-011-D1RA00914A-s1556 [file RA-011-D1RA00914A-s1556.png]

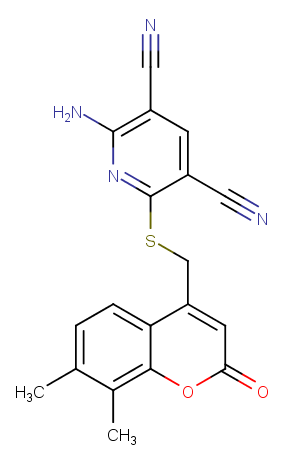

Supplement: RA-011-D1RA00914A-s1557 [file RA-011-D1RA00914A-s1557.png]

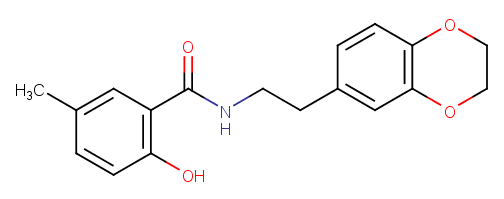

Supplement: RA-011-D1RA00914A-s1558 [file RA-011-D1RA00914A-s1558.png]

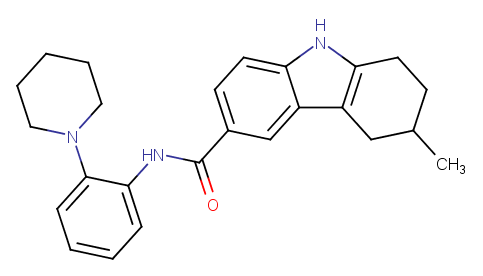

Supplement: RA-011-D1RA00914A-s1559 [file RA-011-D1RA00914A-s1559.png]

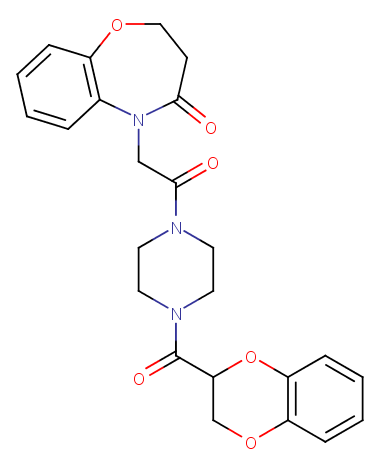

Supplement: RA-011-D1RA00914A-s1560 [file RA-011-D1RA00914A-s1560.png]

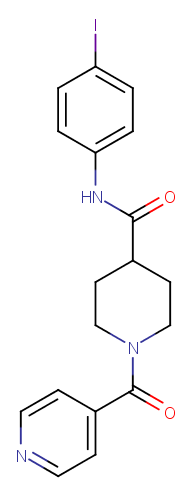

Supplement: RA-011-D1RA00914A-s1561 [file RA-011-D1RA00914A-s1561.png]

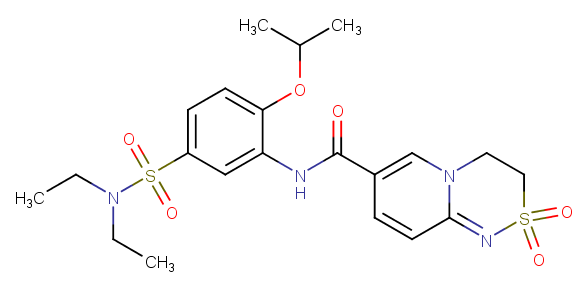

Supplement: RA-011-D1RA00914A-s1562 [file RA-011-D1RA00914A-s1562.png]

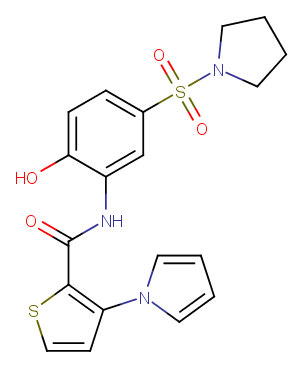

Supplement: RA-011-D1RA00914A-s1563 [file RA-011-D1RA00914A-s1563.png]

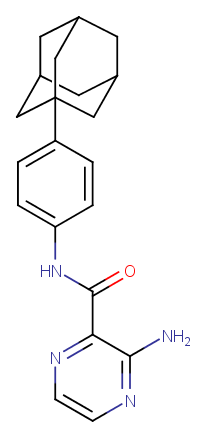

Supplement: RA-011-D1RA00914A-s1564 [file RA-011-D1RA00914A-s1564.png]

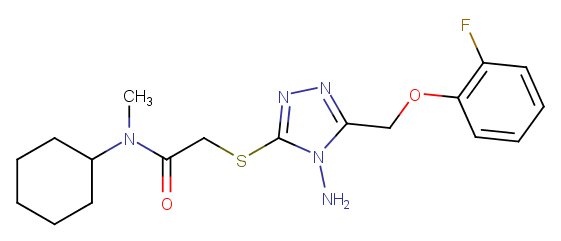

Supplement: RA-011-D1RA00914A-s1565 [file RA-011-D1RA00914A-s1565.png]

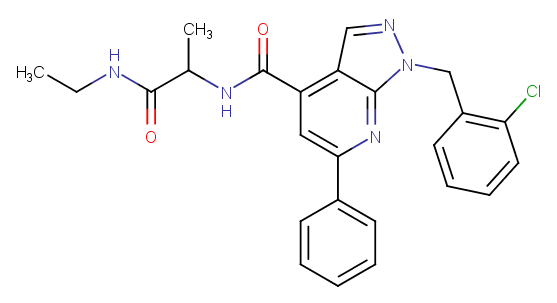

Supplement: RA-011-D1RA00914A-s1566 [file RA-011-D1RA00914A-s1566.png]

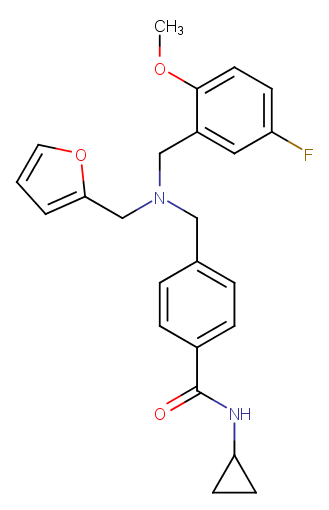

Supplement: RA-011-D1RA00914A-s1567 [file RA-011-D1RA00914A-s1567.png]

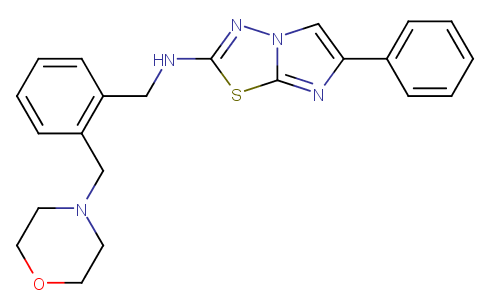

Supplement: RA-011-D1RA00914A-s1568 [file RA-011-D1RA00914A-s1568.png]

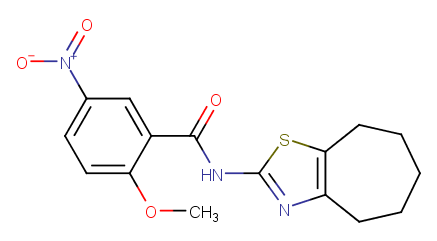

Supplement: RA-011-D1RA00914A-s1569 [file RA-011-D1RA00914A-s1569.png]

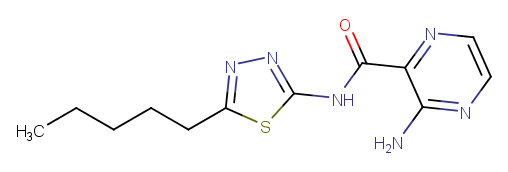

Supplement: RA-011-D1RA00914A-s1570 [file RA-011-D1RA00914A-s1570.png]

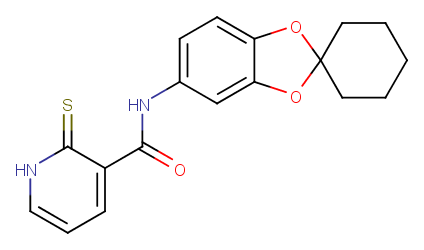

Supplement: RA-011-D1RA00914A-s1571 [file RA-011-D1RA00914A-s1571.png]

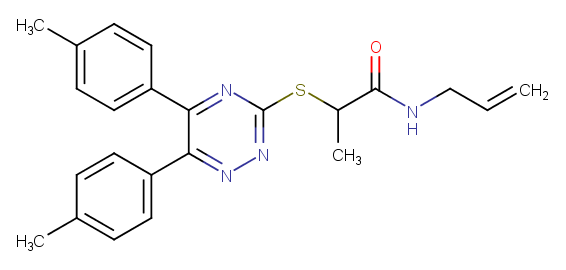

Supplement: RA-011-D1RA00914A-s1572 [file RA-011-D1RA00914A-s1572.png]

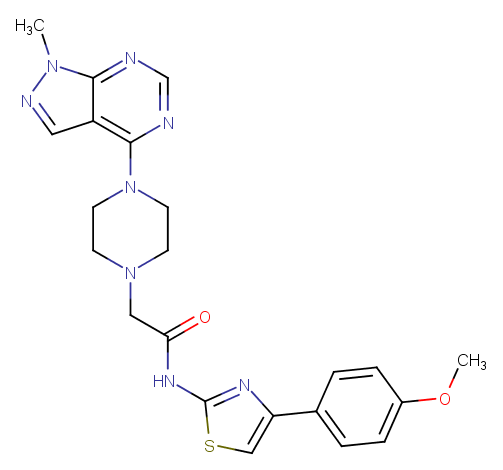

Supplement: RA-011-D1RA00914A-s1573 [file RA-011-D1RA00914A-s1573.png]
