# Supplementary figures and images for: Improved herbicide discovery using physico-chemical rules refined by antimalarial library screening (part 13 of 14)
Source: RSC Adv. 2021 Feb 23;11(15):8459–67. doi: 10.1039/d1ra00914a (PMC8695207; doi:10.1039/d1ra00914a)

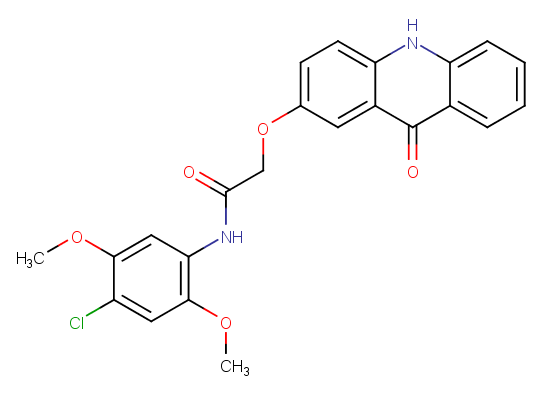

Supplement: RA-011-D1RA00914A-s1574 [file RA-011-D1RA00914A-s1574.png]

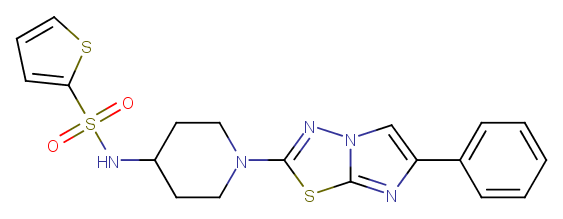

Supplement: RA-011-D1RA00914A-s1575 [file RA-011-D1RA00914A-s1575.png]

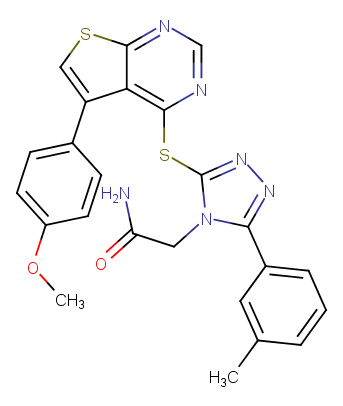

Supplement: RA-011-D1RA00914A-s1576 [file RA-011-D1RA00914A-s1576.png]

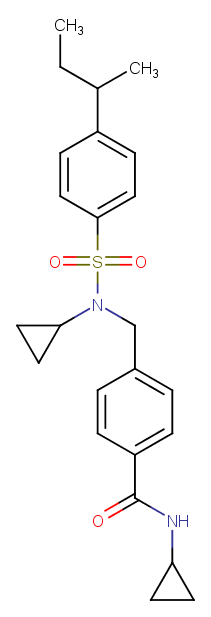

Supplement: RA-011-D1RA00914A-s1577 [file RA-011-D1RA00914A-s1577.png]

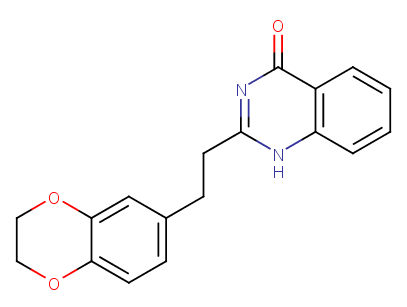

Supplement: RA-011-D1RA00914A-s1578 [file RA-011-D1RA00914A-s1578.png]

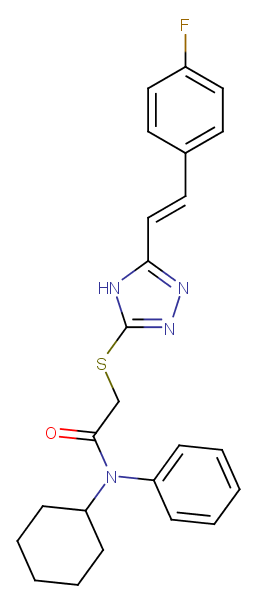

Supplement: RA-011-D1RA00914A-s1579 [file RA-011-D1RA00914A-s1579.png]

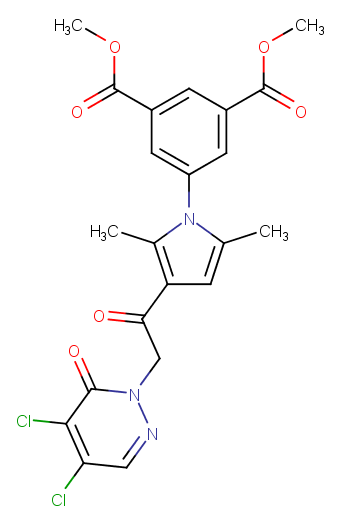

Supplement: RA-011-D1RA00914A-s1580 [file RA-011-D1RA00914A-s1580.png]

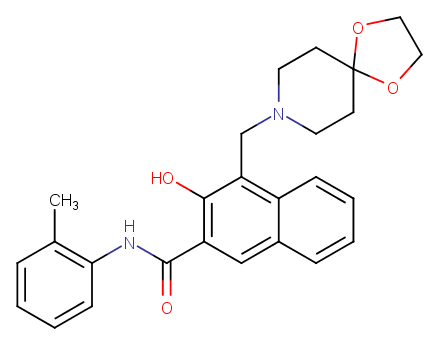

Supplement: RA-011-D1RA00914A-s1581 [file RA-011-D1RA00914A-s1581.png]

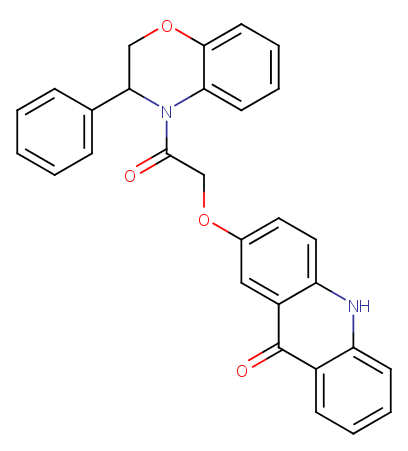

Supplement: RA-011-D1RA00914A-s1582 [file RA-011-D1RA00914A-s1582.png]

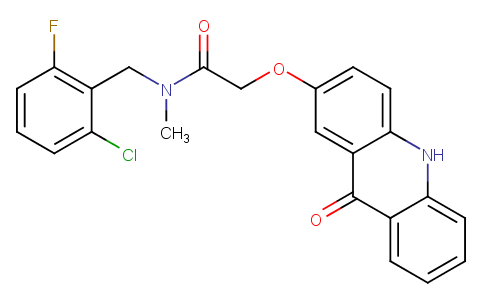

Supplement: RA-011-D1RA00914A-s1583 [file RA-011-D1RA00914A-s1583.png]

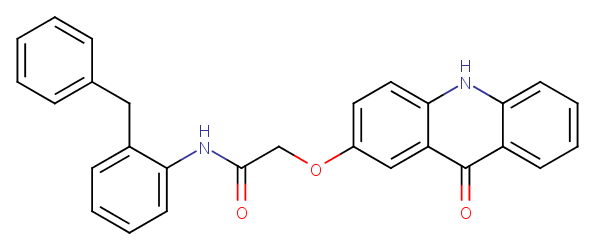

Supplement: RA-011-D1RA00914A-s1584 [file RA-011-D1RA00914A-s1584.png]

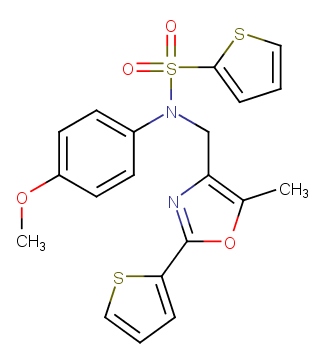

Supplement: RA-011-D1RA00914A-s1585 [file RA-011-D1RA00914A-s1585.png]

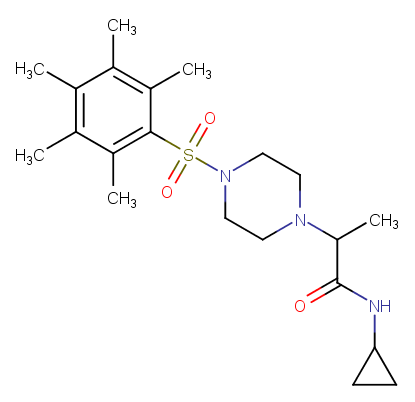

Supplement: RA-011-D1RA00914A-s1586 [file RA-011-D1RA00914A-s1586.png]

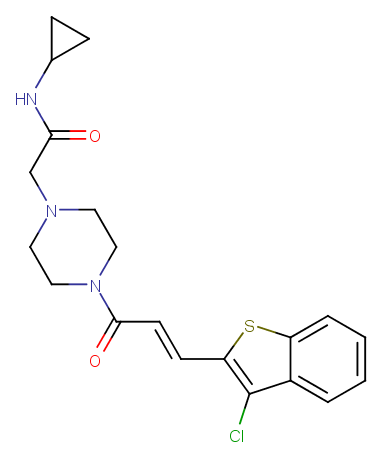

Supplement: RA-011-D1RA00914A-s1587 [file RA-011-D1RA00914A-s1587.png]

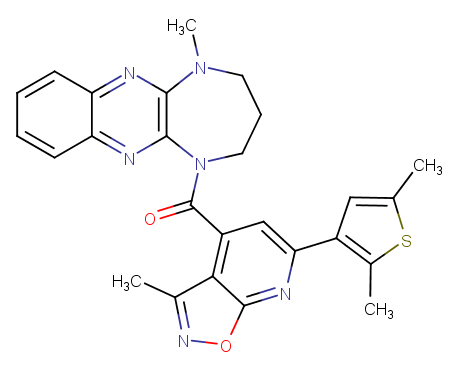

Supplement: RA-011-D1RA00914A-s1588 [file RA-011-D1RA00914A-s1588.png]

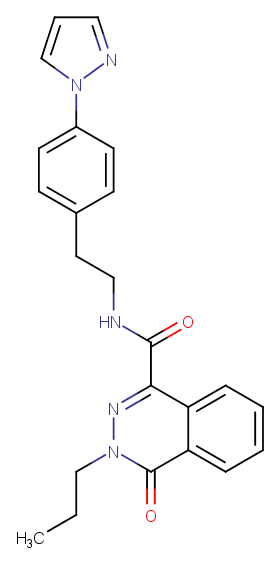

Supplement: RA-011-D1RA00914A-s1589 [file RA-011-D1RA00914A-s1589.png]

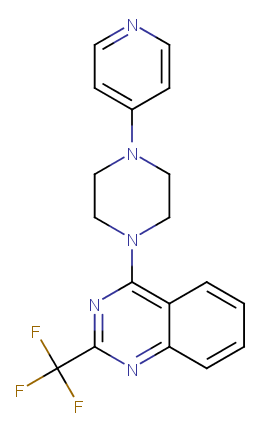

Supplement: RA-011-D1RA00914A-s1590 [file RA-011-D1RA00914A-s1590.png]

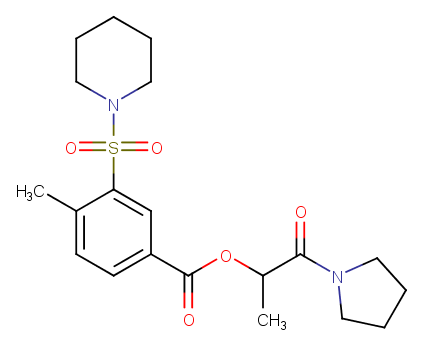

Supplement: RA-011-D1RA00914A-s1591 [file RA-011-D1RA00914A-s1591.png]

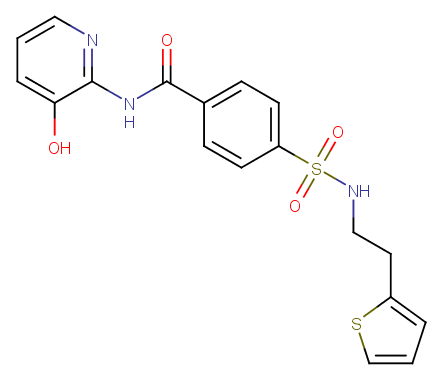

Supplement: RA-011-D1RA00914A-s1592 [file RA-011-D1RA00914A-s1592.png]

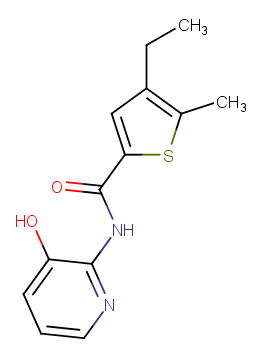

Supplement: RA-011-D1RA00914A-s1593 [file RA-011-D1RA00914A-s1593.png]

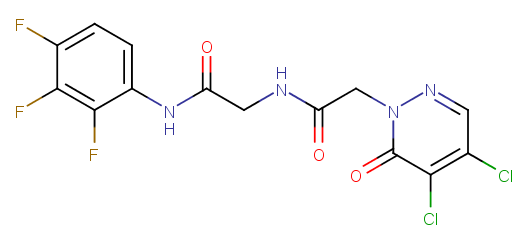

Supplement: RA-011-D1RA00914A-s1594 [file RA-011-D1RA00914A-s1594.png]

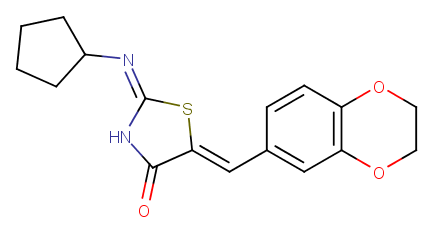

Supplement: RA-011-D1RA00914A-s1595 [file RA-011-D1RA00914A-s1595.png]

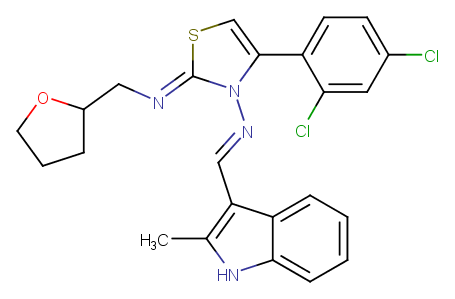

Supplement: RA-011-D1RA00914A-s1596 [file RA-011-D1RA00914A-s1596.png]

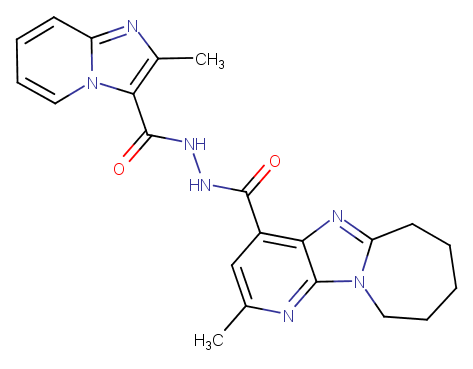

Supplement: RA-011-D1RA00914A-s1597 [file RA-011-D1RA00914A-s1597.png]

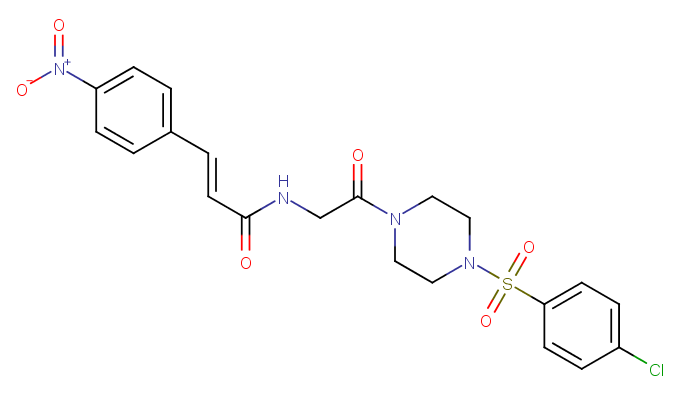

Supplement: RA-011-D1RA00914A-s1598 [file RA-011-D1RA00914A-s1598.png]

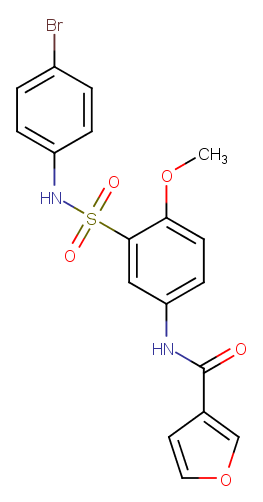

Supplement: RA-011-D1RA00914A-s1599 [file RA-011-D1RA00914A-s1599.png]

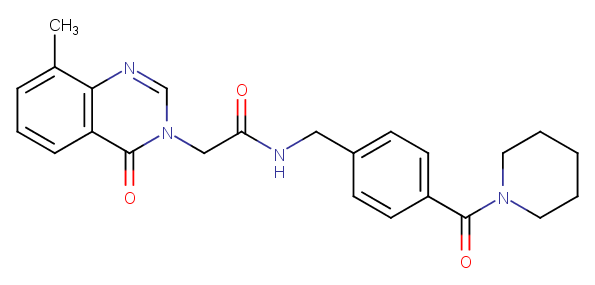

Supplement: RA-011-D1RA00914A-s1600 [file RA-011-D1RA00914A-s1600.png]

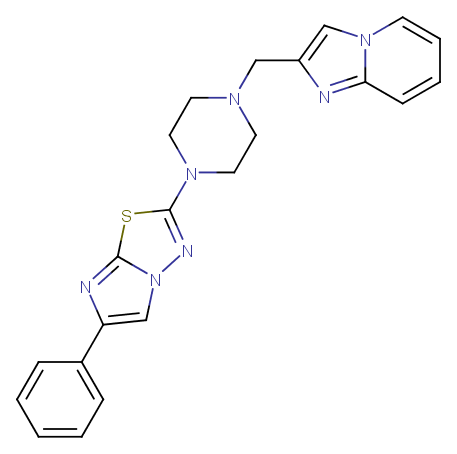

Supplement: RA-011-D1RA00914A-s1601 [file RA-011-D1RA00914A-s1601.png]

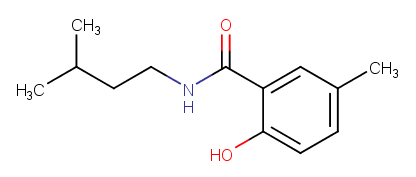

Supplement: RA-011-D1RA00914A-s1602 [file RA-011-D1RA00914A-s1602.png]

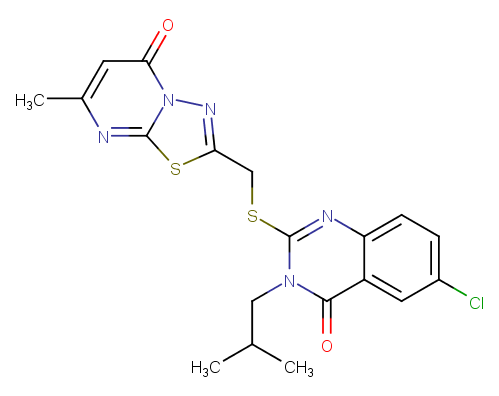

Supplement: RA-011-D1RA00914A-s1603 [file RA-011-D1RA00914A-s1603.png]

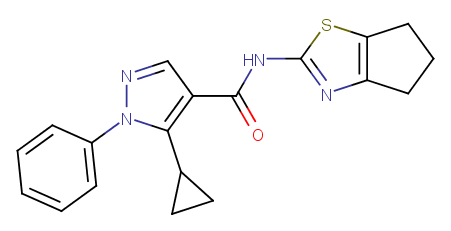

Supplement: RA-011-D1RA00914A-s1604 [file RA-011-D1RA00914A-s1604.png]

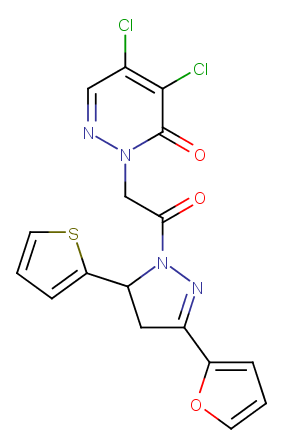

Supplement: RA-011-D1RA00914A-s1605 [file RA-011-D1RA00914A-s1605.png]

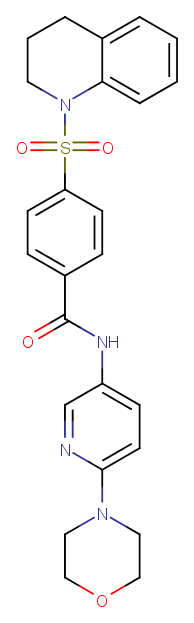

Supplement: RA-011-D1RA00914A-s1606 [file RA-011-D1RA00914A-s1606.png]

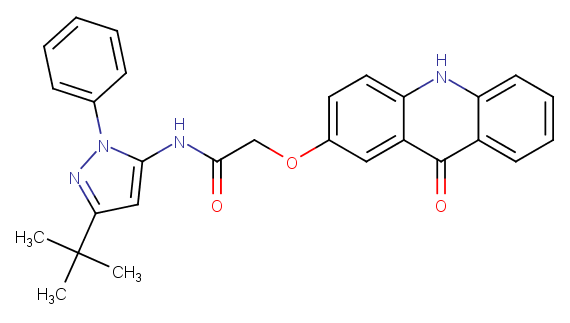

Supplement: RA-011-D1RA00914A-s1607 [file RA-011-D1RA00914A-s1607.png]

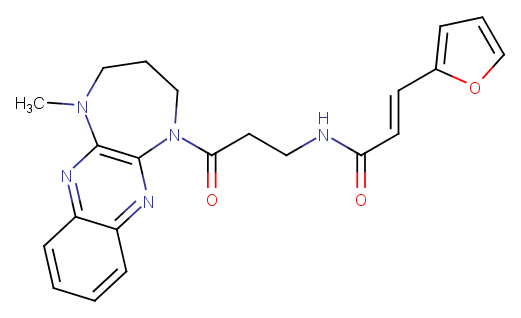

Supplement: RA-011-D1RA00914A-s1608 [file RA-011-D1RA00914A-s1608.png]

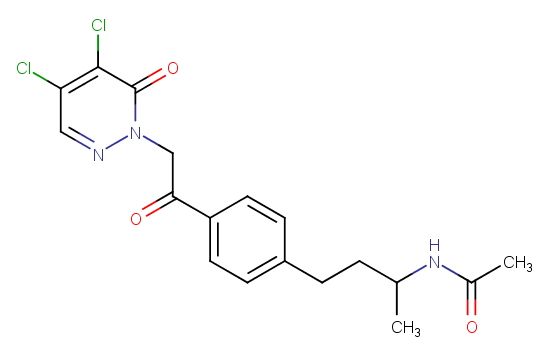

Supplement: RA-011-D1RA00914A-s1609 [file RA-011-D1RA00914A-s1609.png]

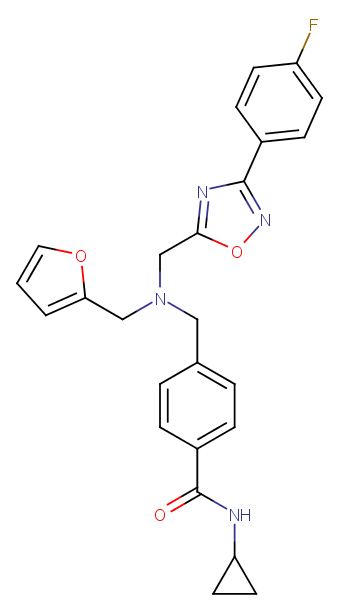

Supplement: RA-011-D1RA00914A-s1610 [file RA-011-D1RA00914A-s1610.png]

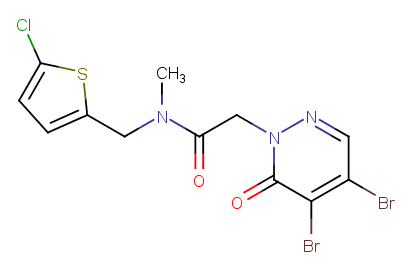

Supplement: RA-011-D1RA00914A-s1611 [file RA-011-D1RA00914A-s1611.png]

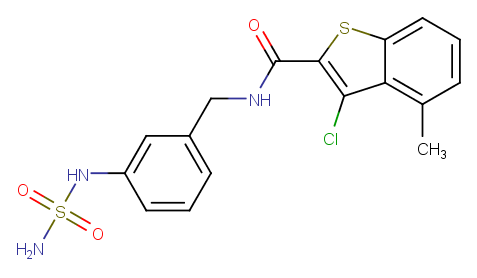

Supplement: RA-011-D1RA00914A-s1612 [file RA-011-D1RA00914A-s1612.png]

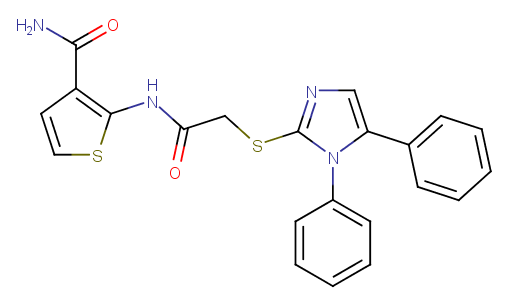

Supplement: RA-011-D1RA00914A-s1613 [file RA-011-D1RA00914A-s1613.png]

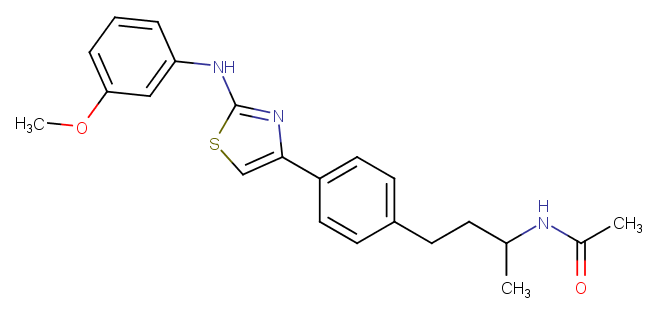

Supplement: RA-011-D1RA00914A-s1614 [file RA-011-D1RA00914A-s1614.png]

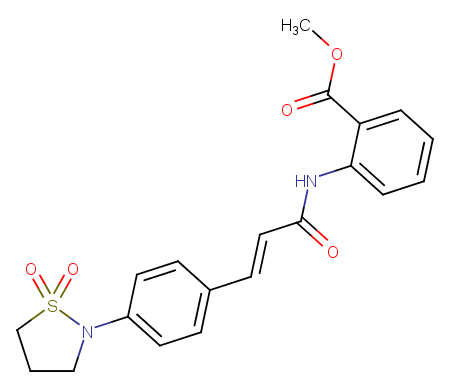

Supplement: RA-011-D1RA00914A-s1615 [file RA-011-D1RA00914A-s1615.png]

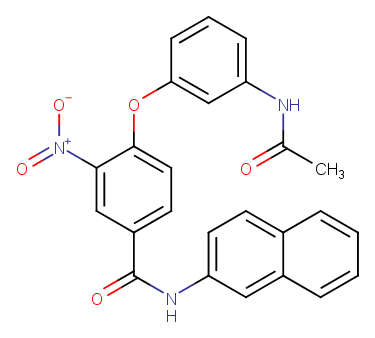

Supplement: RA-011-D1RA00914A-s1616 [file RA-011-D1RA00914A-s1616.png]

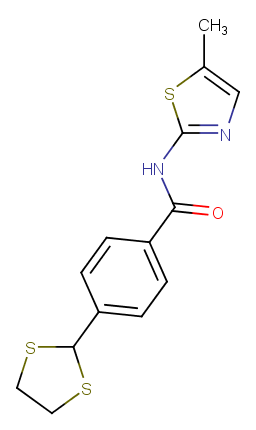

Supplement: RA-011-D1RA00914A-s1617 [file RA-011-D1RA00914A-s1617.png]

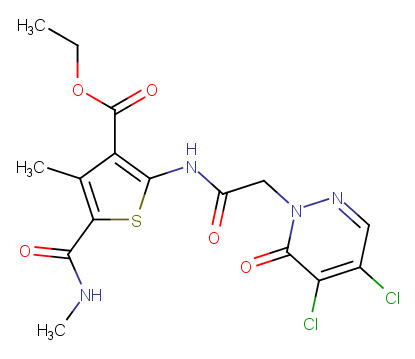

Supplement: RA-011-D1RA00914A-s1618 [file RA-011-D1RA00914A-s1618.png]

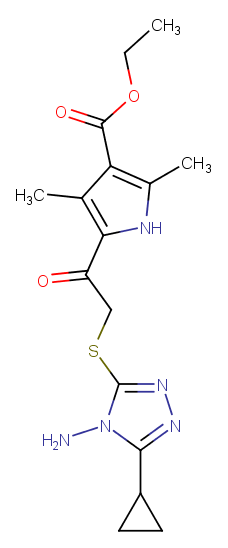

Supplement: RA-011-D1RA00914A-s1619 [file RA-011-D1RA00914A-s1619.png]

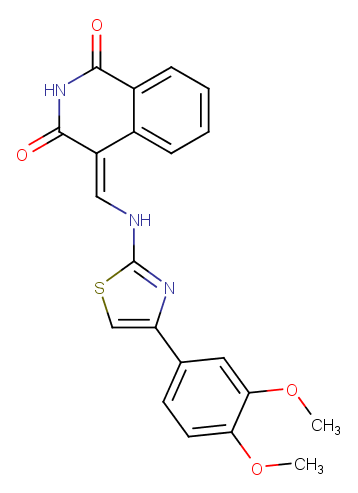

Supplement: RA-011-D1RA00914A-s1620 [file RA-011-D1RA00914A-s1620.png]

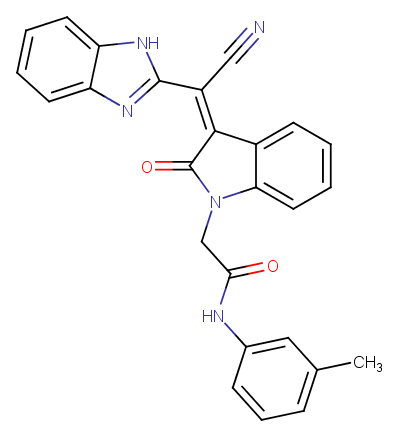

Supplement: RA-011-D1RA00914A-s1621 [file RA-011-D1RA00914A-s1621.png]

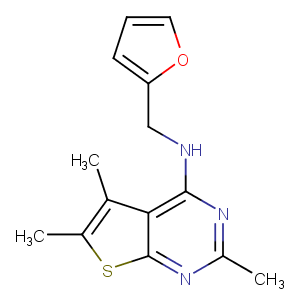

Supplement: RA-011-D1RA00914A-s1622 [file RA-011-D1RA00914A-s1622.png]

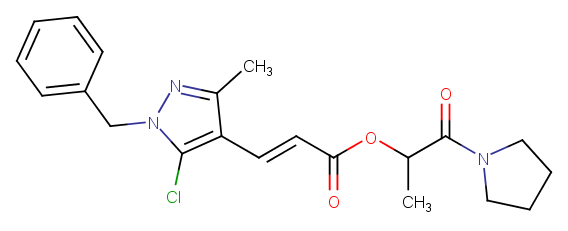

Supplement: RA-011-D1RA00914A-s1623 [file RA-011-D1RA00914A-s1623.png]

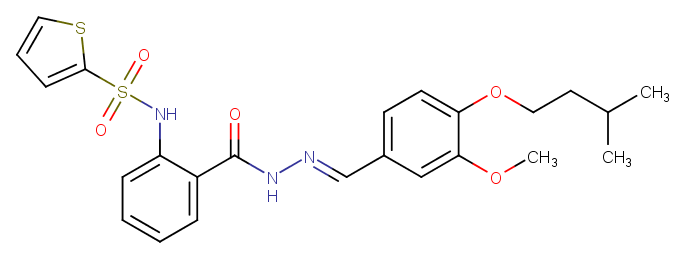

Supplement: RA-011-D1RA00914A-s1624 [file RA-011-D1RA00914A-s1624.png]

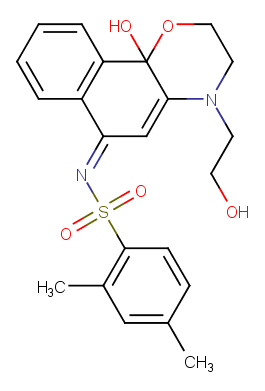

Supplement: RA-011-D1RA00914A-s1625 [file RA-011-D1RA00914A-s1625.png]

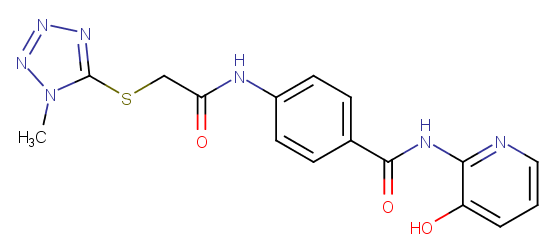

Supplement: RA-011-D1RA00914A-s1626 [file RA-011-D1RA00914A-s1626.png]

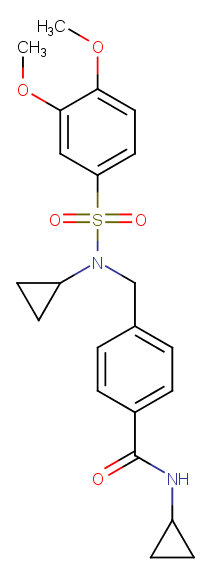

Supplement: RA-011-D1RA00914A-s1627 [file RA-011-D1RA00914A-s1627.png]

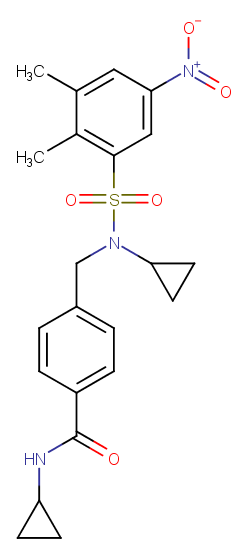

Supplement: RA-011-D1RA00914A-s1628 [file RA-011-D1RA00914A-s1628.png]

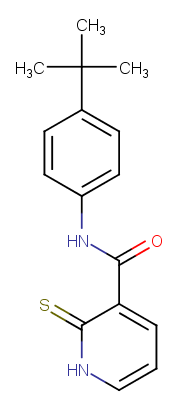

Supplement: RA-011-D1RA00914A-s1629 [file RA-011-D1RA00914A-s1629.png]

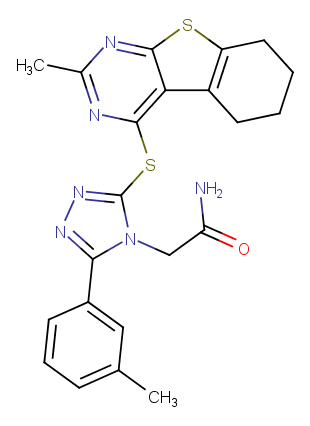

Supplement: RA-011-D1RA00914A-s1630 [file RA-011-D1RA00914A-s1630.png]

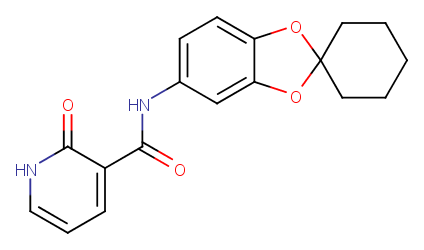

Supplement: RA-011-D1RA00914A-s1631 [file RA-011-D1RA00914A-s1631.png]

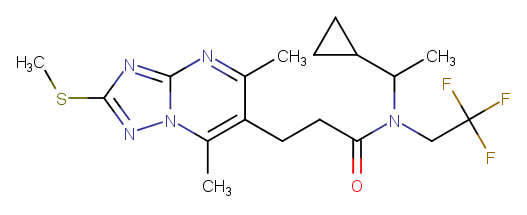

Supplement: RA-011-D1RA00914A-s1632 [file RA-011-D1RA00914A-s1632.png]

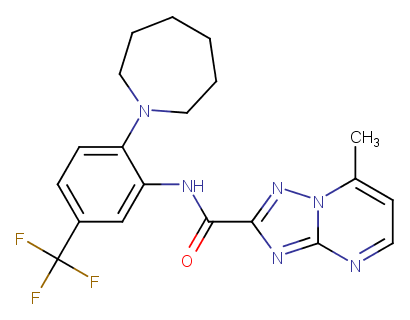

Supplement: RA-011-D1RA00914A-s1633 [file RA-011-D1RA00914A-s1633.png]

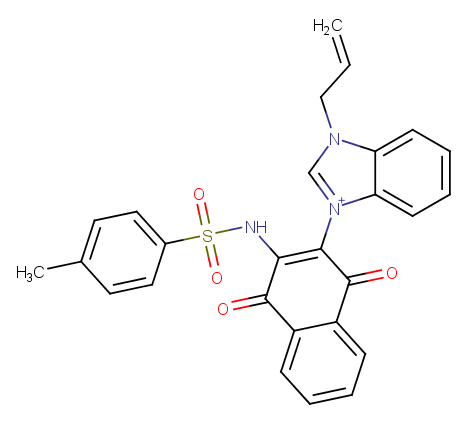

Supplement: RA-011-D1RA00914A-s1634 [file RA-011-D1RA00914A-s1634.png]

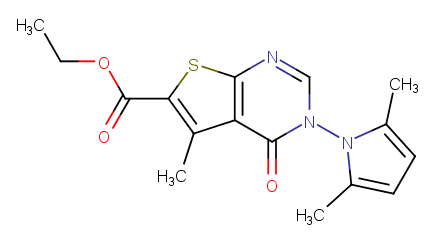

Supplement: RA-011-D1RA00914A-s1635 [file RA-011-D1RA00914A-s1635.png]

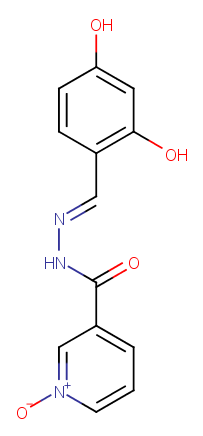

Supplement: RA-011-D1RA00914A-s1636 [file RA-011-D1RA00914A-s1636.png]

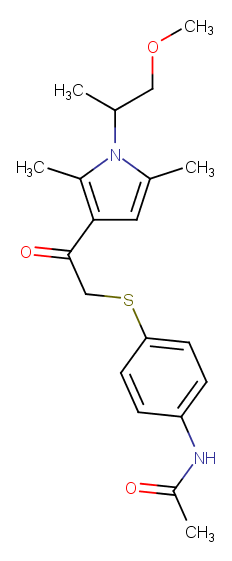

Supplement: RA-011-D1RA00914A-s1637 [file RA-011-D1RA00914A-s1637.png]

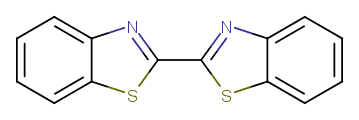

Supplement: RA-011-D1RA00914A-s1638 [file RA-011-D1RA00914A-s1638.png]

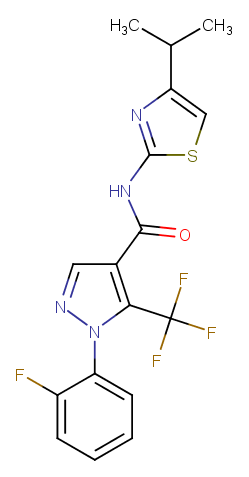

Supplement: RA-011-D1RA00914A-s1639 [file RA-011-D1RA00914A-s1639.png]

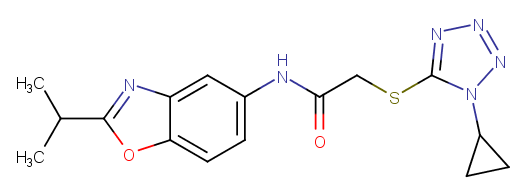

Supplement: RA-011-D1RA00914A-s1640 [file RA-011-D1RA00914A-s1640.png]

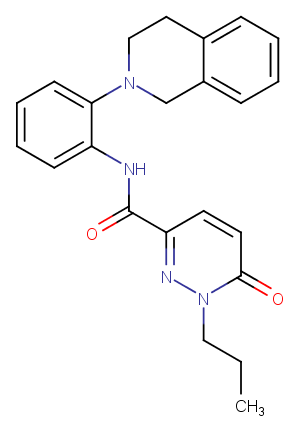

Supplement: RA-011-D1RA00914A-s1641 [file RA-011-D1RA00914A-s1641.png]

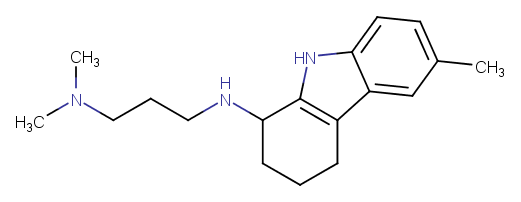

Supplement: RA-011-D1RA00914A-s1642 [file RA-011-D1RA00914A-s1642.png]

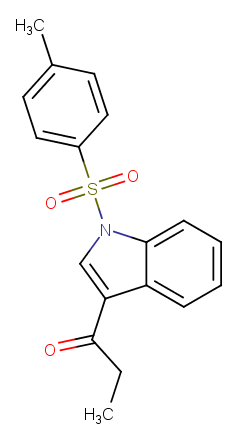

Supplement: RA-011-D1RA00914A-s1643 [file RA-011-D1RA00914A-s1643.png]

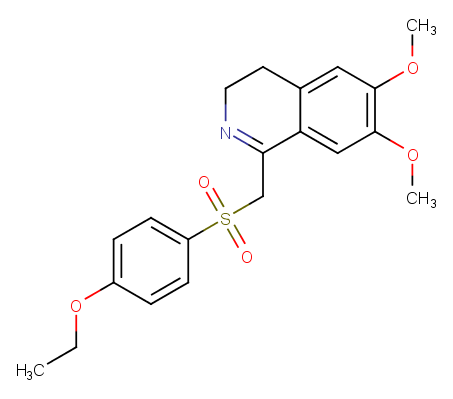

Supplement: RA-011-D1RA00914A-s1644 [file RA-011-D1RA00914A-s1644.png]

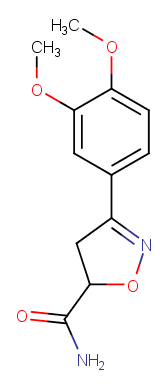

Supplement: RA-011-D1RA00914A-s1645 [file RA-011-D1RA00914A-s1645.png]

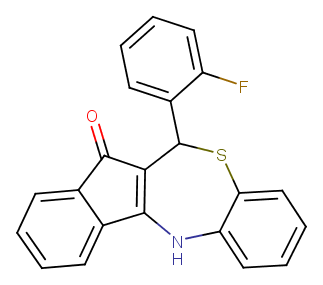

Supplement: RA-011-D1RA00914A-s1646 [file RA-011-D1RA00914A-s1646.png]

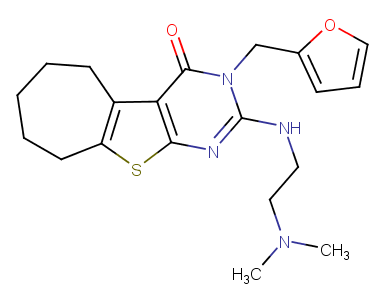

Supplement: RA-011-D1RA00914A-s1647 [file RA-011-D1RA00914A-s1647.png]

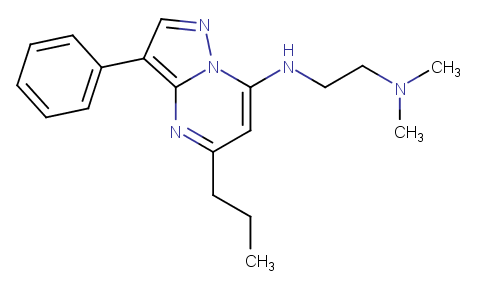

Supplement: RA-011-D1RA00914A-s1648 [file RA-011-D1RA00914A-s1648.png]

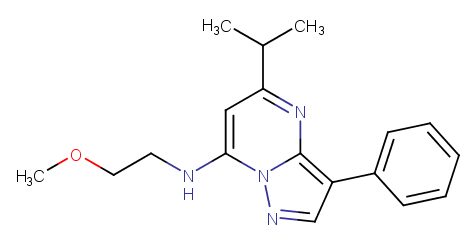

Supplement: RA-011-D1RA00914A-s1649 [file RA-011-D1RA00914A-s1649.png]

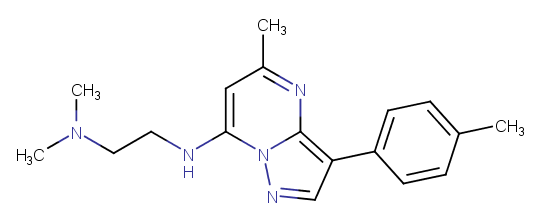

Supplement: RA-011-D1RA00914A-s1650 [file RA-011-D1RA00914A-s1650.png]

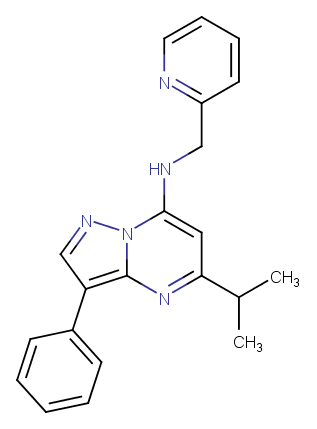

Supplement: RA-011-D1RA00914A-s1651 [file RA-011-D1RA00914A-s1651.png]

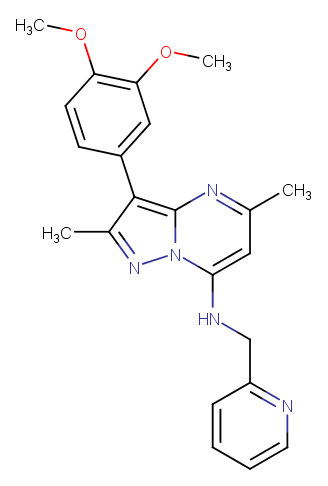

Supplement: RA-011-D1RA00914A-s1652 [file RA-011-D1RA00914A-s1652.png]

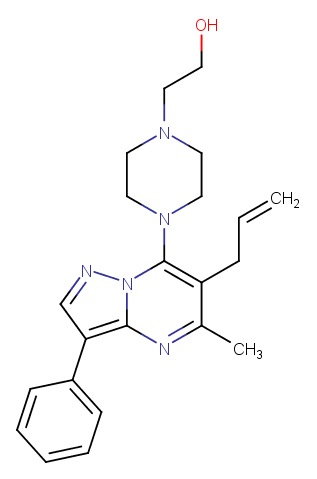

Supplement: RA-011-D1RA00914A-s1653 [file RA-011-D1RA00914A-s1653.png]

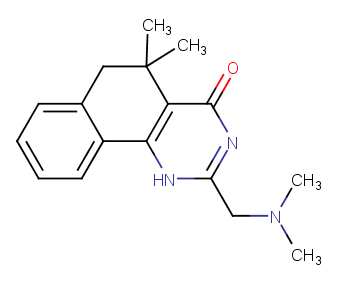

Supplement: RA-011-D1RA00914A-s1654 [file RA-011-D1RA00914A-s1654.png]

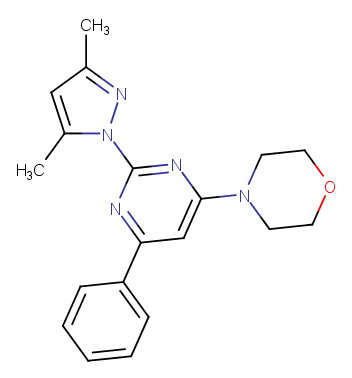

Supplement: RA-011-D1RA00914A-s1655 [file RA-011-D1RA00914A-s1655.png]

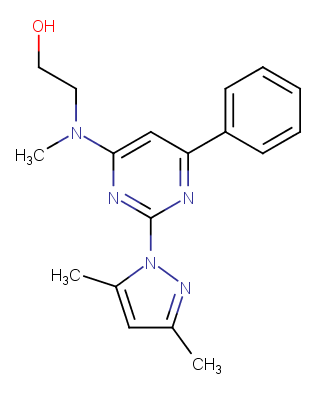

Supplement: RA-011-D1RA00914A-s1656 [file RA-011-D1RA00914A-s1656.png]

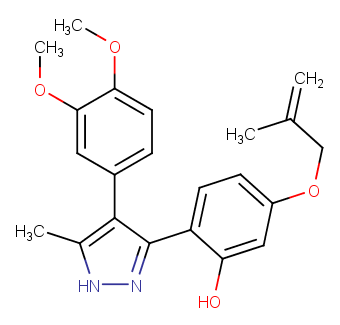

Supplement: RA-011-D1RA00914A-s1657 [file RA-011-D1RA00914A-s1657.png]

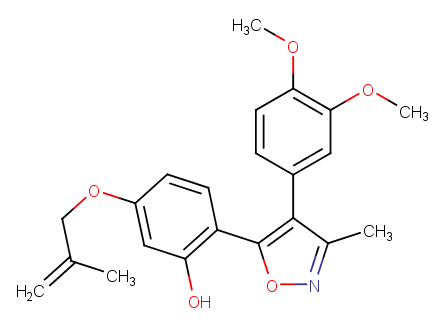

Supplement: RA-011-D1RA00914A-s1658 [file RA-011-D1RA00914A-s1658.png]

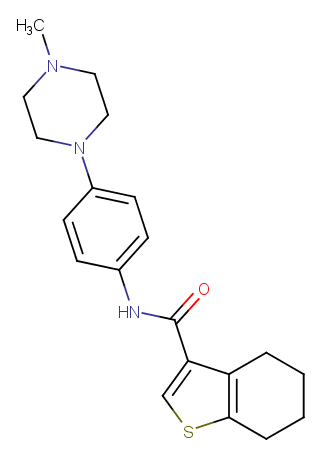

Supplement: RA-011-D1RA00914A-s1659 [file RA-011-D1RA00914A-s1659.png]

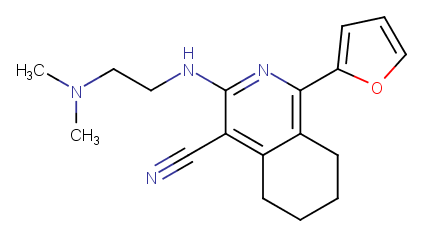

Supplement: RA-011-D1RA00914A-s1660 [file RA-011-D1RA00914A-s1660.png]

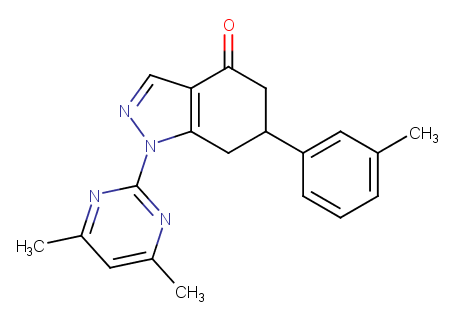

Supplement: RA-011-D1RA00914A-s1661 [file RA-011-D1RA00914A-s1661.png]

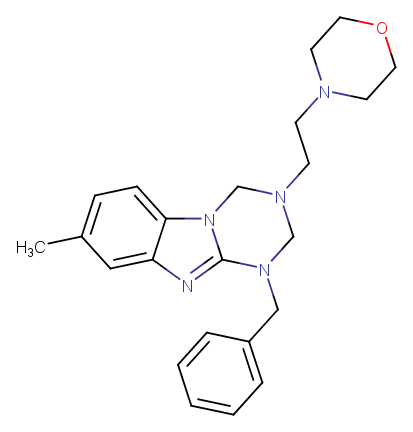

Supplement: RA-011-D1RA00914A-s1662 [file RA-011-D1RA00914A-s1662.png]

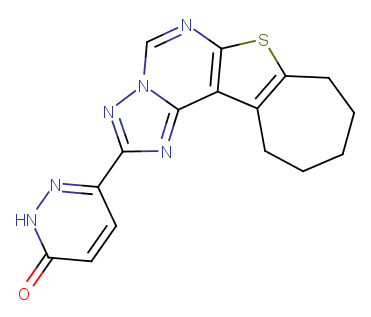

Supplement: RA-011-D1RA00914A-s1663 [file RA-011-D1RA00914A-s1663.png]

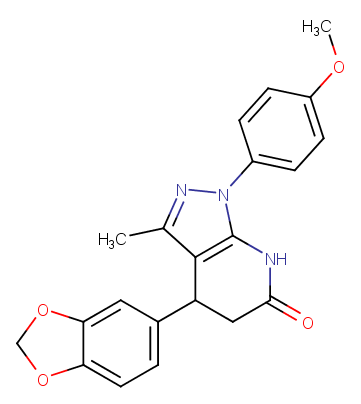

Supplement: RA-011-D1RA00914A-s1664 [file RA-011-D1RA00914A-s1664.png]

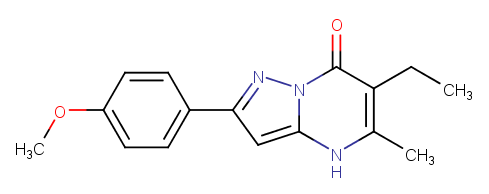

Supplement: RA-011-D1RA00914A-s1665 [file RA-011-D1RA00914A-s1665.png]

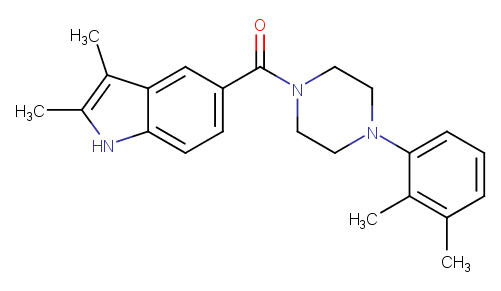

Supplement: RA-011-D1RA00914A-s1666 [file RA-011-D1RA00914A-s1666.png]

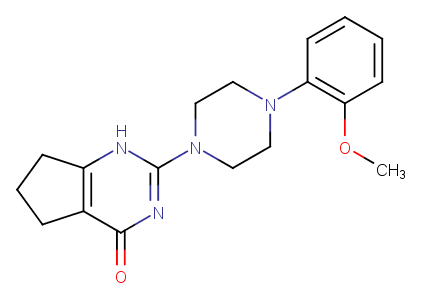

Supplement: RA-011-D1RA00914A-s1667 [file RA-011-D1RA00914A-s1667.png]

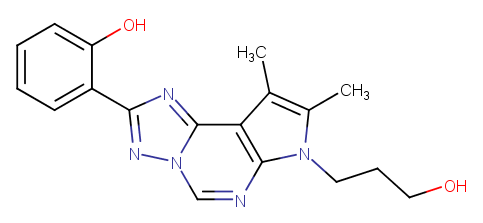

Supplement: RA-011-D1RA00914A-s1668 [file RA-011-D1RA00914A-s1668.png]

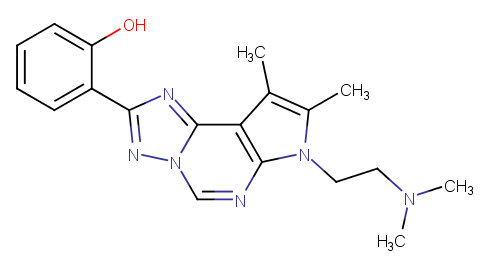

Supplement: RA-011-D1RA00914A-s1669 [file RA-011-D1RA00914A-s1669.png]

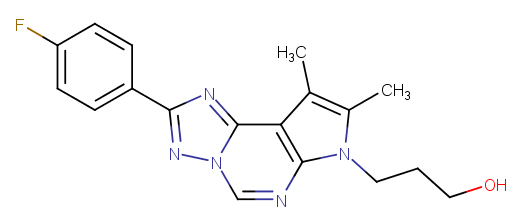

Supplement: RA-011-D1RA00914A-s1670 [file RA-011-D1RA00914A-s1670.png]

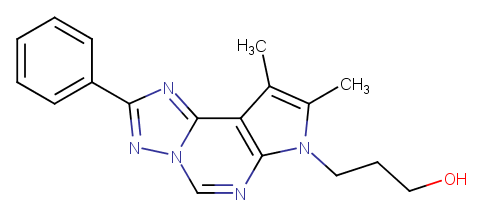

Supplement: RA-011-D1RA00914A-s1671 [file RA-011-D1RA00914A-s1671.png]

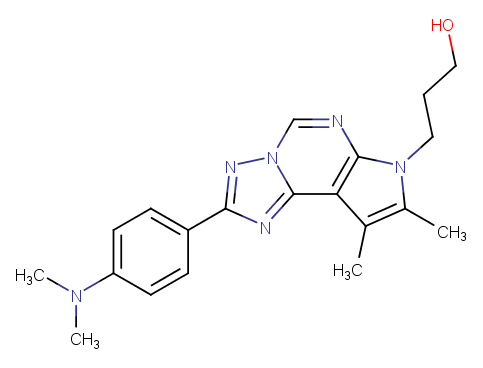

Supplement: RA-011-D1RA00914A-s1672 [file RA-011-D1RA00914A-s1672.png]

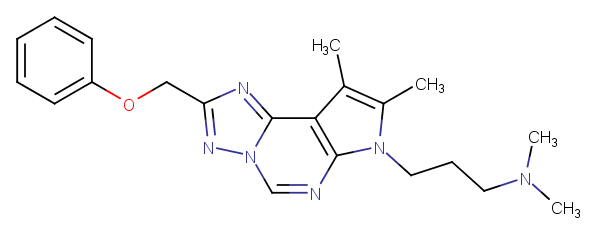

Supplement: RA-011-D1RA00914A-s1673 [file RA-011-D1RA00914A-s1673.png]
