# Supplementary figures and images for: Improved herbicide discovery using physico-chemical rules refined by antimalarial library screening (part 14 of 14)
Source: RSC Adv. 2021 Feb 23;11(15):8459–67. doi: 10.1039/d1ra00914a (PMC8695207; doi:10.1039/d1ra00914a)

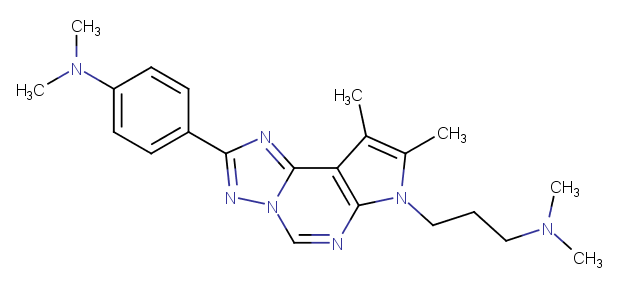

Supplement: RA-011-D1RA00914A-s1674 [file RA-011-D1RA00914A-s1674.png]

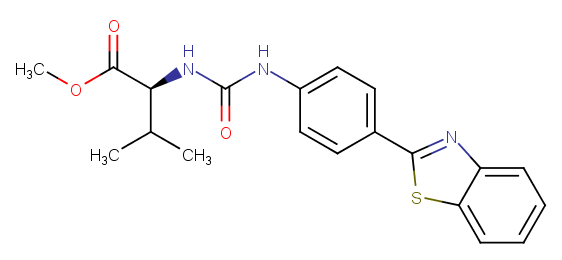

Supplement: RA-011-D1RA00914A-s1675 [file RA-011-D1RA00914A-s1675.png]

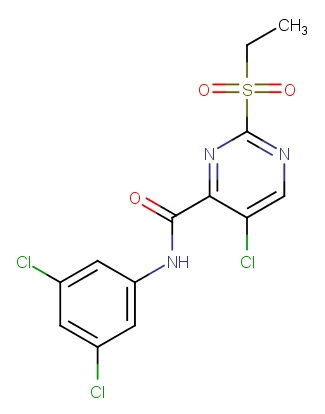

Supplement: RA-011-D1RA00914A-s1676 [file RA-011-D1RA00914A-s1676.png]

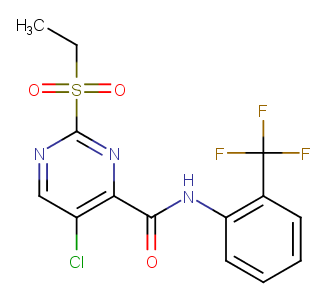

Supplement: RA-011-D1RA00914A-s1677 [file RA-011-D1RA00914A-s1677.png]

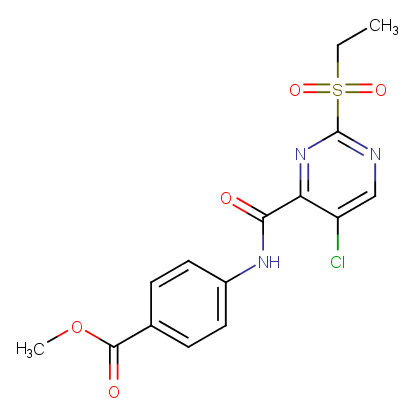

Supplement: RA-011-D1RA00914A-s1678 [file RA-011-D1RA00914A-s1678.png]

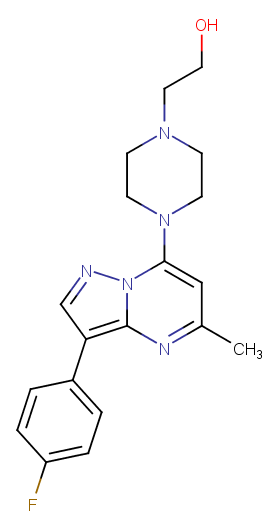

Supplement: RA-011-D1RA00914A-s1679 [file RA-011-D1RA00914A-s1679.png]

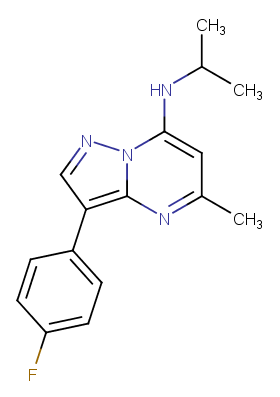

Supplement: RA-011-D1RA00914A-s1680 [file RA-011-D1RA00914A-s1680.png]

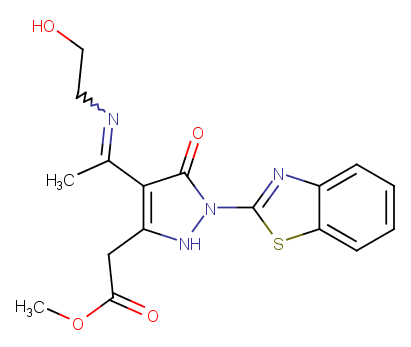

Supplement: RA-011-D1RA00914A-s1681 [file RA-011-D1RA00914A-s1681.png]

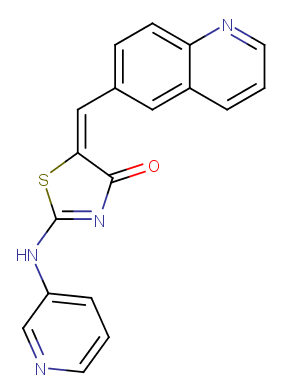

Supplement: RA-011-D1RA00914A-s1682 [file RA-011-D1RA00914A-s1682.png]

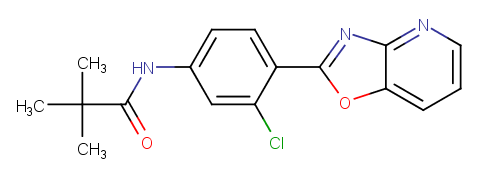

Supplement: RA-011-D1RA00914A-s1683 [file RA-011-D1RA00914A-s1683.png]

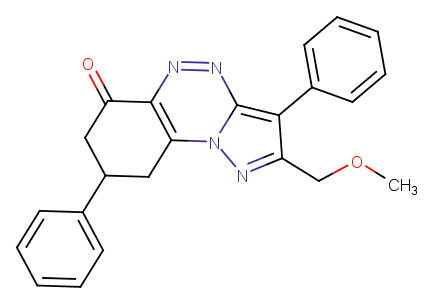

Supplement: RA-011-D1RA00914A-s1684 [file RA-011-D1RA00914A-s1684.png]

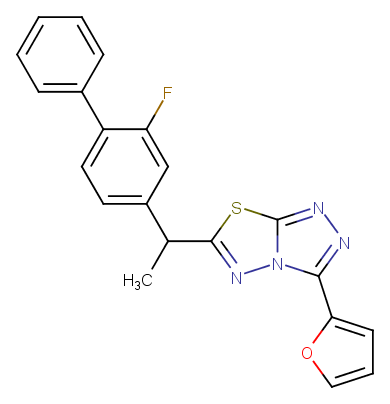

Supplement: RA-011-D1RA00914A-s1685 [file RA-011-D1RA00914A-s1685.png]

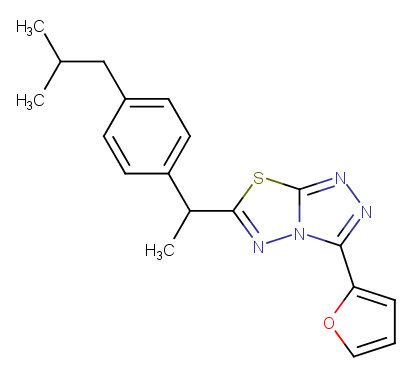

Supplement: RA-011-D1RA00914A-s1686 [file RA-011-D1RA00914A-s1686.png]

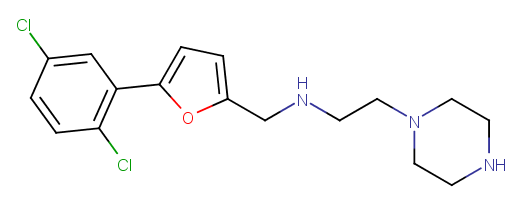

Supplement: RA-011-D1RA00914A-s1687 [file RA-011-D1RA00914A-s1687.png]

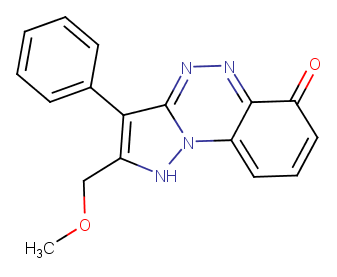

Supplement: RA-011-D1RA00914A-s1688 [file RA-011-D1RA00914A-s1688.png]

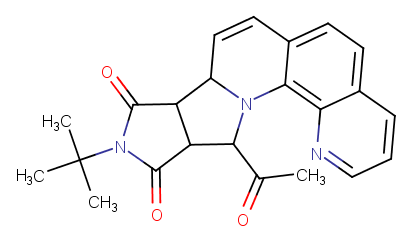

Supplement: RA-011-D1RA00914A-s1689 [file RA-011-D1RA00914A-s1689.png]

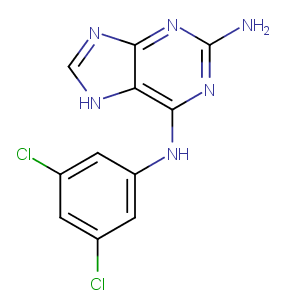

Supplement: RA-011-D1RA00914A-s1690 [file RA-011-D1RA00914A-s1690.png]

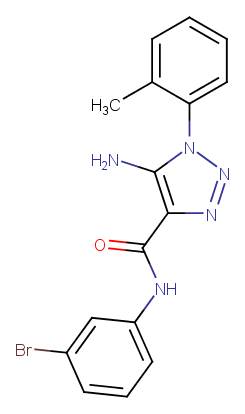

Supplement: RA-011-D1RA00914A-s1691 [file RA-011-D1RA00914A-s1691.png]

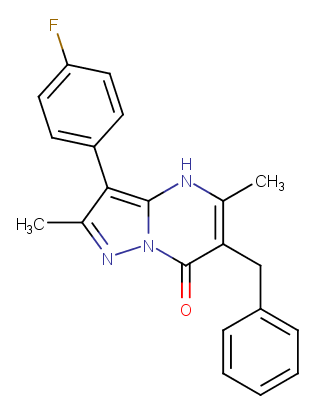

Supplement: RA-011-D1RA00914A-s1692 [file RA-011-D1RA00914A-s1692.png]

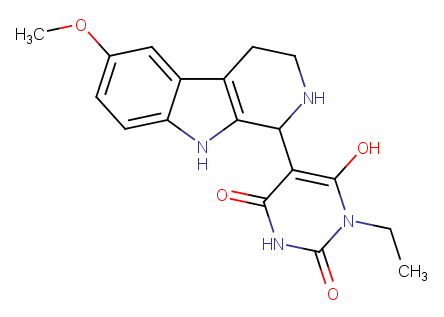

Supplement: RA-011-D1RA00914A-s1693 [file RA-011-D1RA00914A-s1693.png]

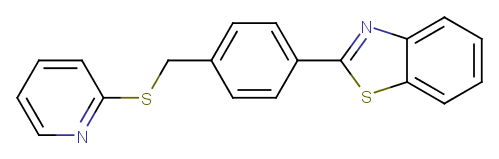

Supplement: RA-011-D1RA00914A-s1694 [file RA-011-D1RA00914A-s1694.png]

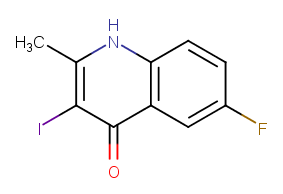

Supplement: RA-011-D1RA00914A-s1695 [file RA-011-D1RA00914A-s1695.png]

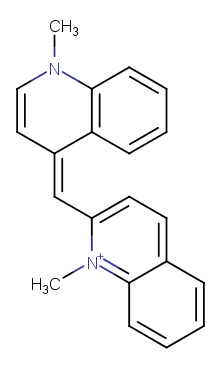

Supplement: RA-011-D1RA00914A-s1696 [file RA-011-D1RA00914A-s1696.png]

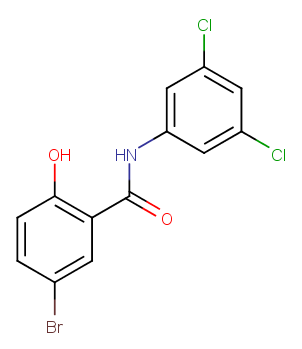

Supplement: RA-011-D1RA00914A-s1697 [file RA-011-D1RA00914A-s1697.png]

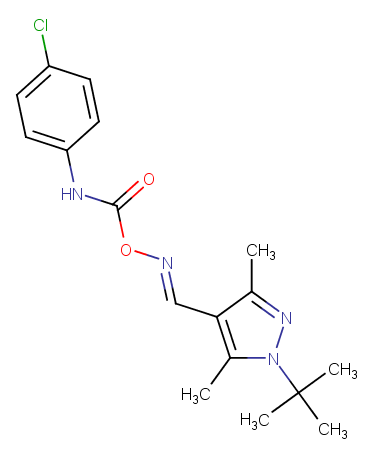

Supplement: RA-011-D1RA00914A-s1698 [file RA-011-D1RA00914A-s1698.png]

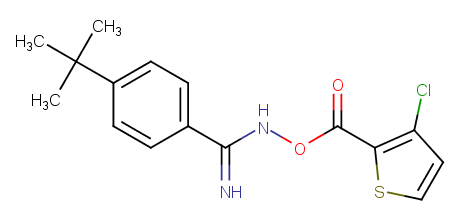

Supplement: RA-011-D1RA00914A-s1699 [file RA-011-D1RA00914A-s1699.png]

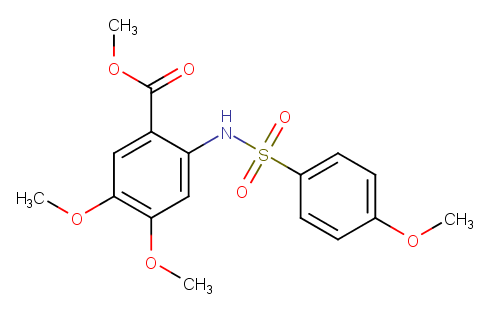

Supplement: RA-011-D1RA00914A-s1700 [file RA-011-D1RA00914A-s1700.png]

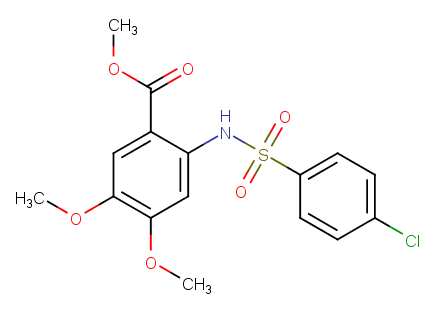

Supplement: RA-011-D1RA00914A-s1701 [file RA-011-D1RA00914A-s1701.png]

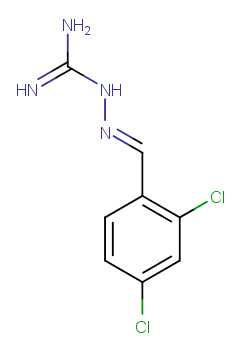

Supplement: RA-011-D1RA00914A-s1702 [file RA-011-D1RA00914A-s1702.png]

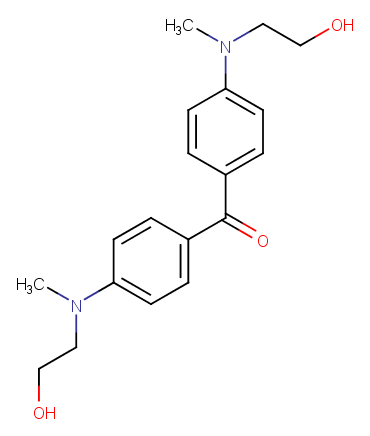

Supplement: RA-011-D1RA00914A-s1703 [file RA-011-D1RA00914A-s1703.png]

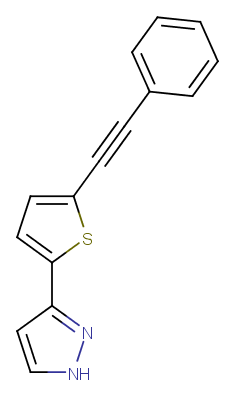

Supplement: RA-011-D1RA00914A-s1704 [file RA-011-D1RA00914A-s1704.png]

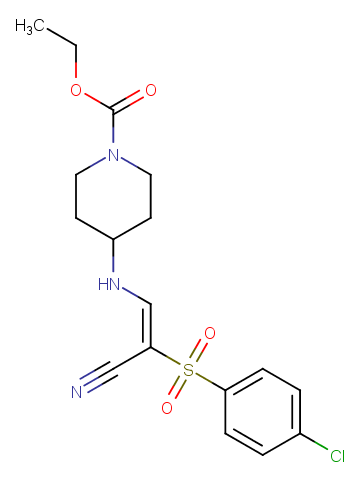

Supplement: RA-011-D1RA00914A-s1705 [file RA-011-D1RA00914A-s1705.png]

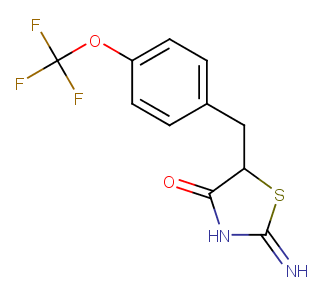

Supplement: RA-011-D1RA00914A-s1706 [file RA-011-D1RA00914A-s1706.png]

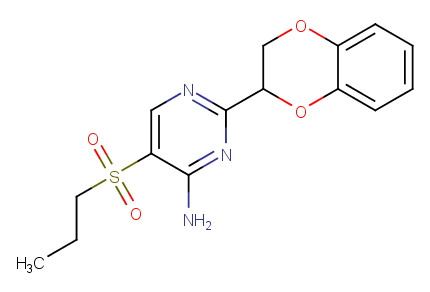

Supplement: RA-011-D1RA00914A-s1707 [file RA-011-D1RA00914A-s1707.png]

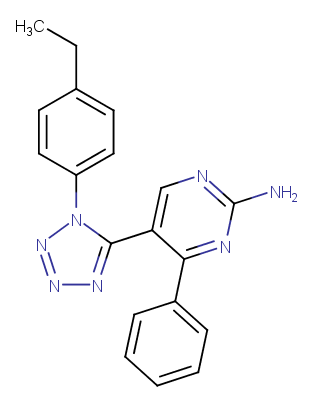

Supplement: RA-011-D1RA00914A-s1708 [file RA-011-D1RA00914A-s1708.png]

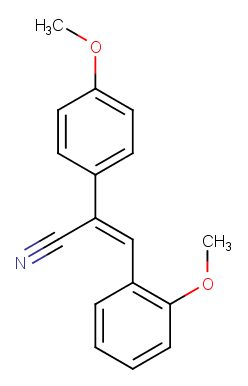

Supplement: RA-011-D1RA00914A-s1709 [file RA-011-D1RA00914A-s1709.png]

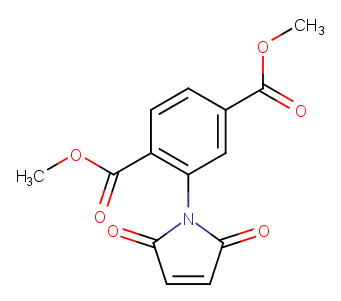

Supplement: RA-011-D1RA00914A-s1710 [file RA-011-D1RA00914A-s1710.png]

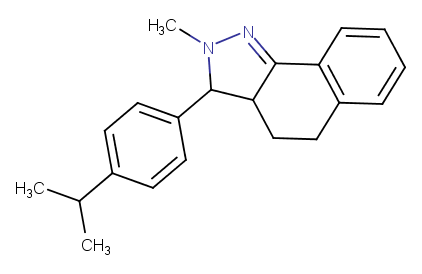

Supplement: RA-011-D1RA00914A-s1711 [file RA-011-D1RA00914A-s1711.png]

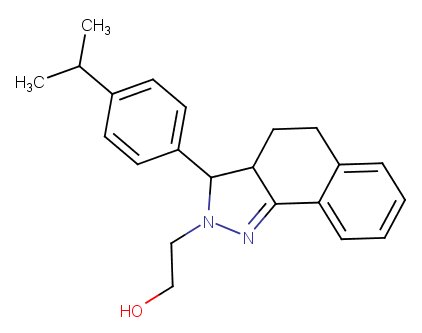

Supplement: RA-011-D1RA00914A-s1712 [file RA-011-D1RA00914A-s1712.png]

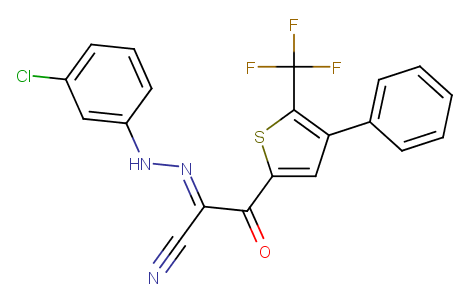

Supplement: RA-011-D1RA00914A-s1713 [file RA-011-D1RA00914A-s1713.png]

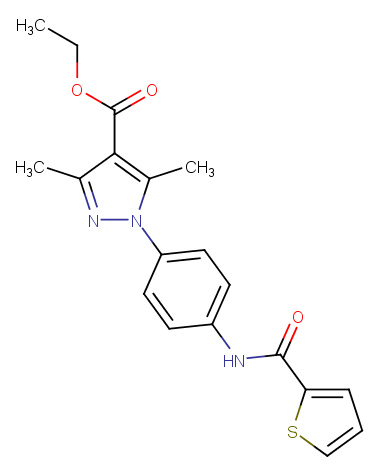

Supplement: RA-011-D1RA00914A-s1714 [file RA-011-D1RA00914A-s1714.png]

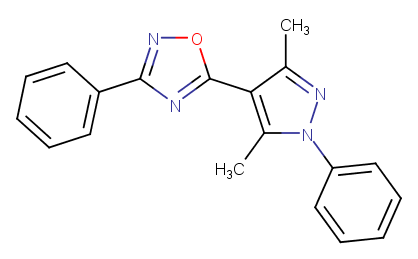

Supplement: RA-011-D1RA00914A-s1715 [file RA-011-D1RA00914A-s1715.png]

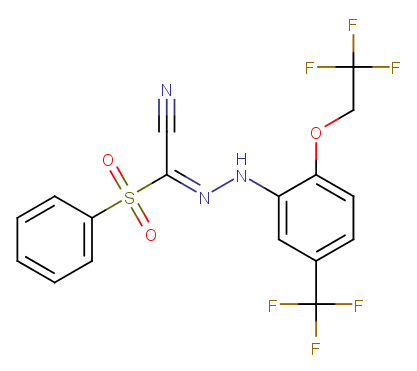

Supplement: RA-011-D1RA00914A-s1716 [file RA-011-D1RA00914A-s1716.png]

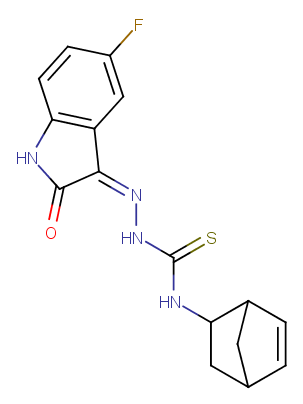

Supplement: RA-011-D1RA00914A-s1717 [file RA-011-D1RA00914A-s1717.png]

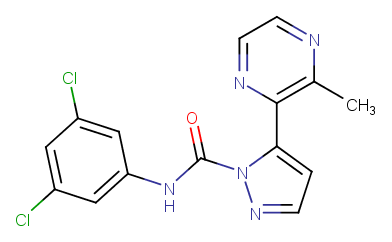

Supplement: RA-011-D1RA00914A-s1718 [file RA-011-D1RA00914A-s1718.png]

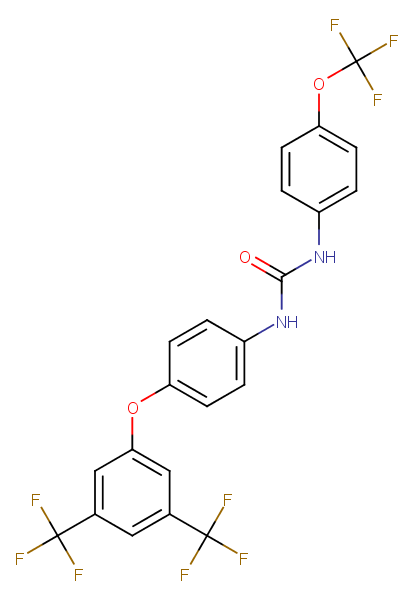

Supplement: RA-011-D1RA00914A-s1719 [file RA-011-D1RA00914A-s1719.png]

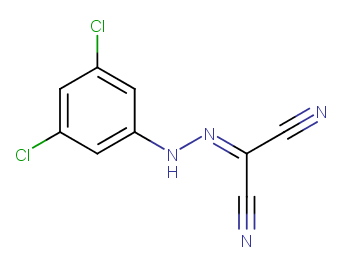

Supplement: RA-011-D1RA00914A-s1720 [file RA-011-D1RA00914A-s1720.png]

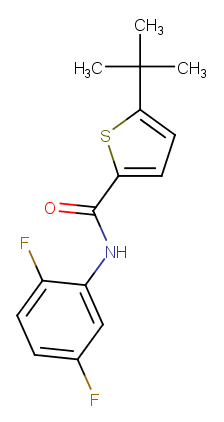

Supplement: RA-011-D1RA00914A-s1721 [file RA-011-D1RA00914A-s1721.png]

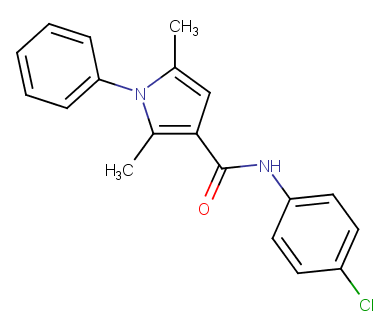

Supplement: RA-011-D1RA00914A-s1722 [file RA-011-D1RA00914A-s1722.png]

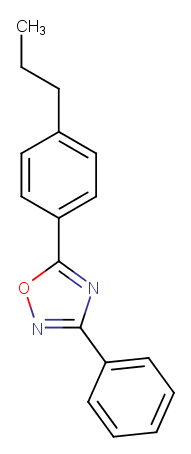

Supplement: RA-011-D1RA00914A-s1723 [file RA-011-D1RA00914A-s1723.png]

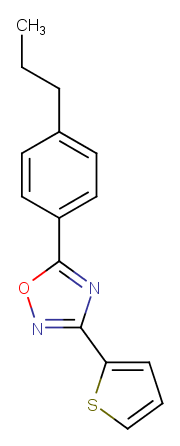

Supplement: RA-011-D1RA00914A-s1724 [file RA-011-D1RA00914A-s1724.png]

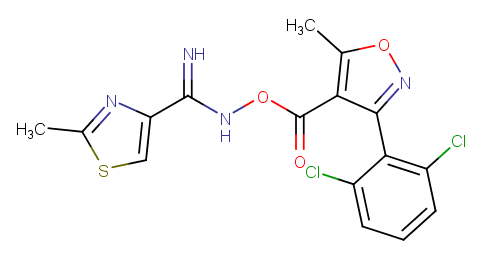

Supplement: RA-011-D1RA00914A-s1725 [file RA-011-D1RA00914A-s1725.png]

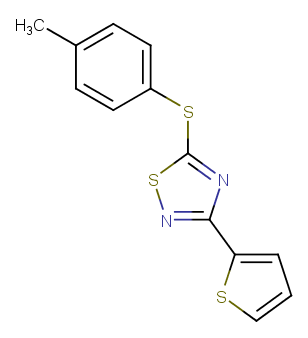

Supplement: RA-011-D1RA00914A-s1726 [file RA-011-D1RA00914A-s1726.png]

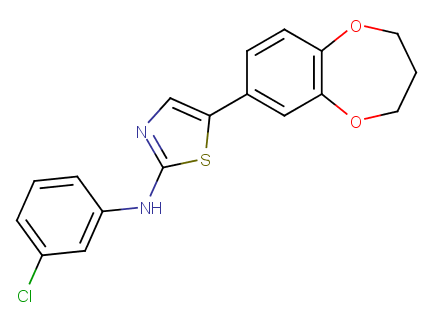

Supplement: RA-011-D1RA00914A-s1727 [file RA-011-D1RA00914A-s1727.png]

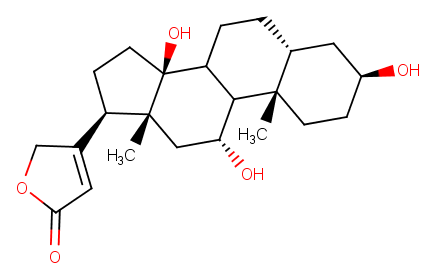

Supplement: RA-011-D1RA00914A-s1728 [file RA-011-D1RA00914A-s1728.png]

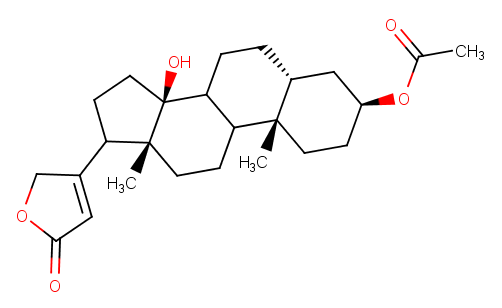

Supplement: RA-011-D1RA00914A-s1729 [file RA-011-D1RA00914A-s1729.png]

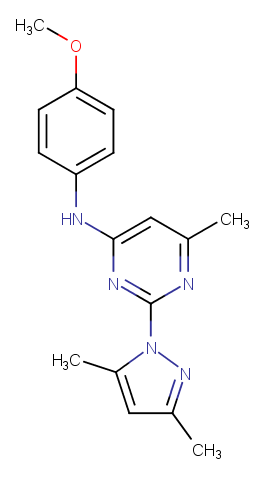

Supplement: RA-011-D1RA00914A-s1730 [file RA-011-D1RA00914A-s1730.png]

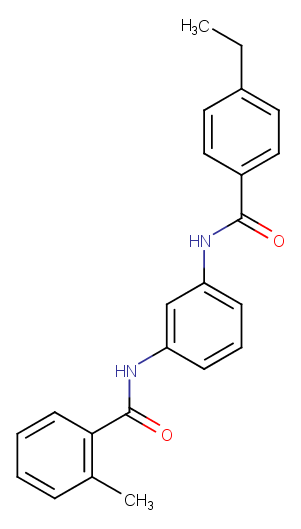

Supplement: RA-011-D1RA00914A-s1731 [file RA-011-D1RA00914A-s1731.png]

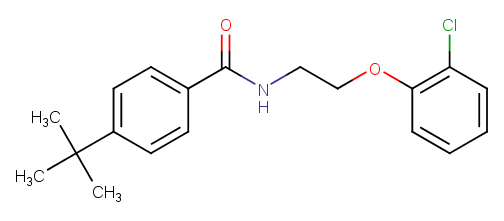

Supplement: RA-011-D1RA00914A-s1732 [file RA-011-D1RA00914A-s1732.png]

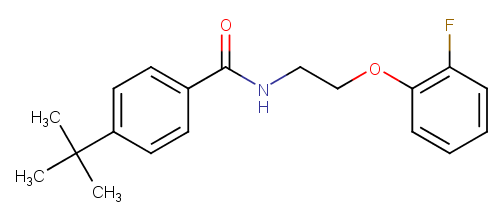

Supplement: RA-011-D1RA00914A-s1733 [file RA-011-D1RA00914A-s1733.png]

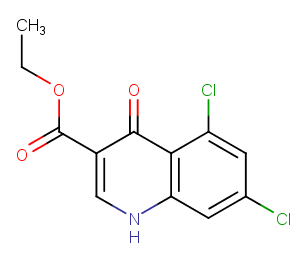

Supplement: RA-011-D1RA00914A-s1734 [file RA-011-D1RA00914A-s1734.png]

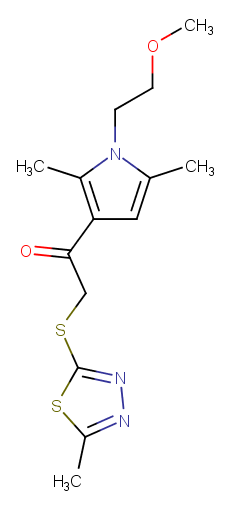

Supplement: RA-011-D1RA00914A-s1735 [file RA-011-D1RA00914A-s1735.png]

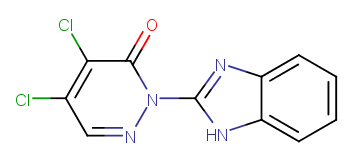

Supplement: RA-011-D1RA00914A-s1736 [file RA-011-D1RA00914A-s1736.png]

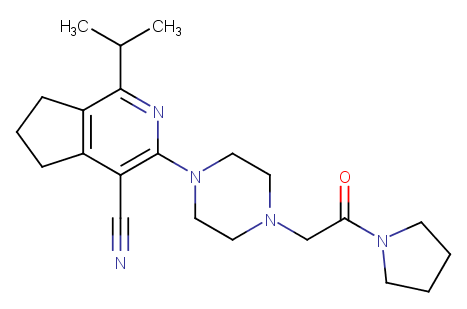

Supplement: RA-011-D1RA00914A-s1737 [file RA-011-D1RA00914A-s1737.png]

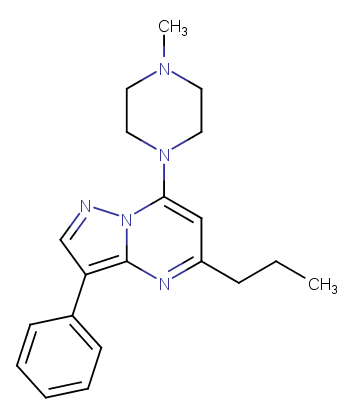

Supplement: RA-011-D1RA00914A-s1738 [file RA-011-D1RA00914A-s1738.png]

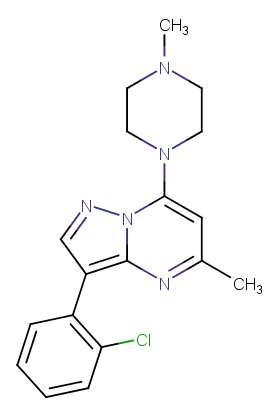

Supplement: RA-011-D1RA00914A-s1739 [file RA-011-D1RA00914A-s1739.png]

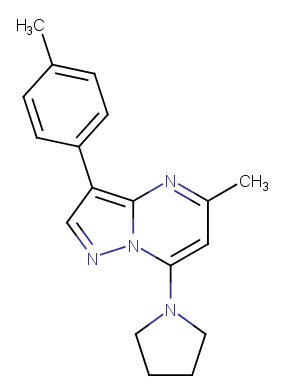

Supplement: RA-011-D1RA00914A-s1740 [file RA-011-D1RA00914A-s1740.png]

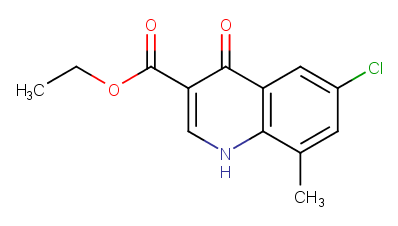

Supplement: RA-011-D1RA00914A-s1741 [file RA-011-D1RA00914A-s1741.png]

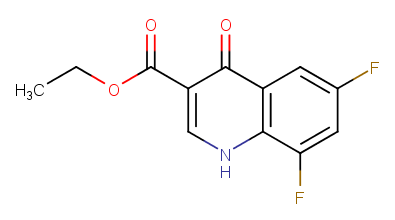

Supplement: RA-011-D1RA00914A-s1742 [file RA-011-D1RA00914A-s1742.png]

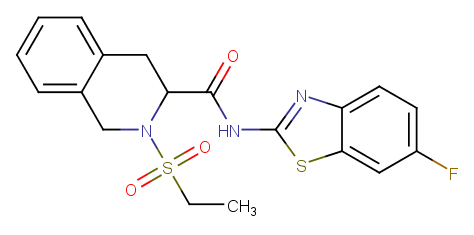

Supplement: RA-011-D1RA00914A-s1743 [file RA-011-D1RA00914A-s1743.png]

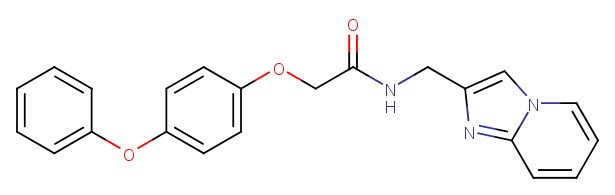

Supplement: RA-011-D1RA00914A-s1744 [file RA-011-D1RA00914A-s1744.png]

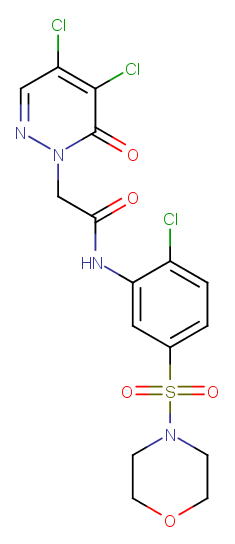

Supplement: RA-011-D1RA00914A-s1745 [file RA-011-D1RA00914A-s1745.png]

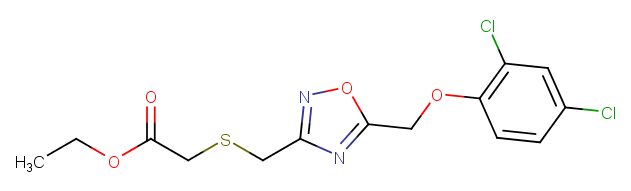

Supplement: RA-011-D1RA00914A-s1746 [file RA-011-D1RA00914A-s1746.png]

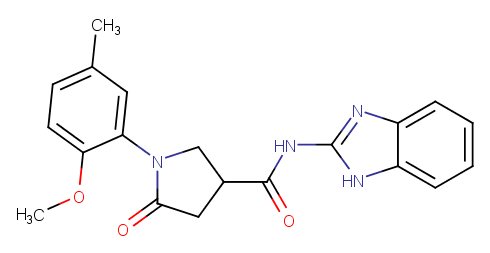

Supplement: RA-011-D1RA00914A-s1747 [file RA-011-D1RA00914A-s1747.png]

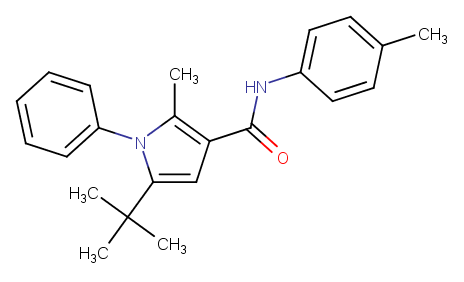

Supplement: RA-011-D1RA00914A-s1748 [file RA-011-D1RA00914A-s1748.png]

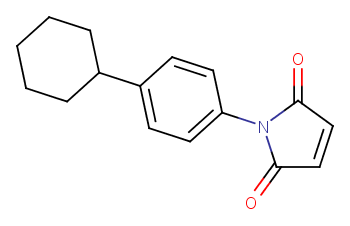

Supplement: RA-011-D1RA00914A-s1749 [file RA-011-D1RA00914A-s1749.png]

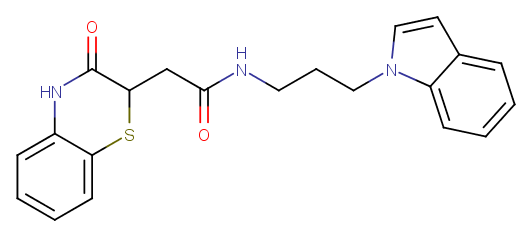

Supplement: RA-011-D1RA00914A-s1750 [file RA-011-D1RA00914A-s1750.png]

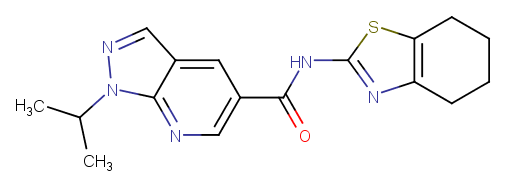

Supplement: RA-011-D1RA00914A-s1751 [file RA-011-D1RA00914A-s1751.png]

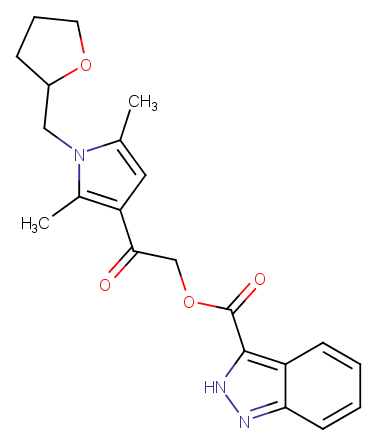

Supplement: RA-011-D1RA00914A-s1752 [file RA-011-D1RA00914A-s1752.png]

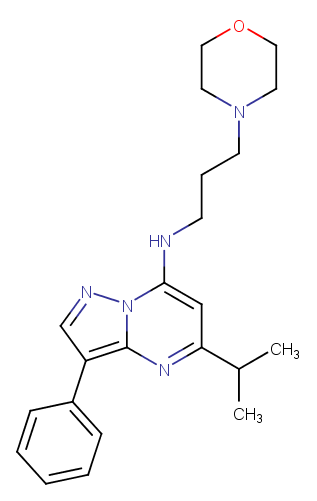

Supplement: RA-011-D1RA00914A-s1753 [file RA-011-D1RA00914A-s1753.png]

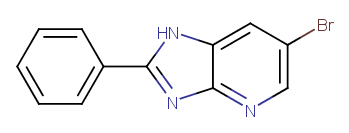

Supplement: RA-011-D1RA00914A-s1754 [file RA-011-D1RA00914A-s1754.png]

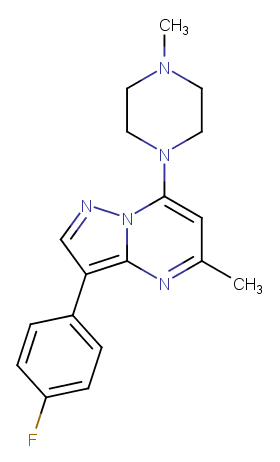

Supplement: RA-011-D1RA00914A-s1755 [file RA-011-D1RA00914A-s1755.png]

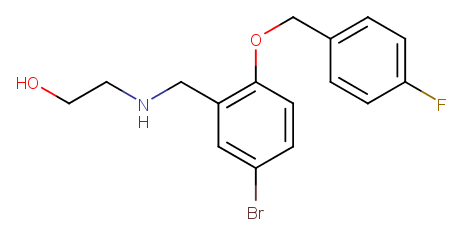

Supplement: RA-011-D1RA00914A-s1756 [file RA-011-D1RA00914A-s1756.png]

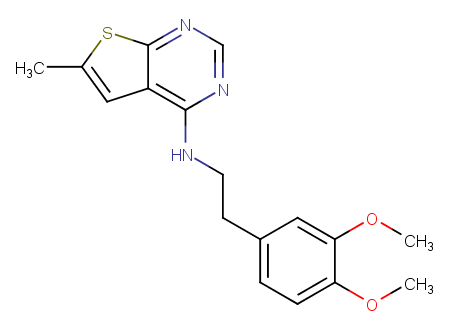

Supplement: RA-011-D1RA00914A-s1757 [file RA-011-D1RA00914A-s1757.png]

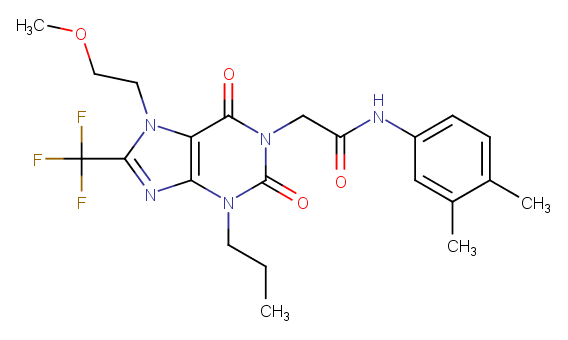

Supplement: RA-011-D1RA00914A-s1758 [file RA-011-D1RA00914A-s1758.png]

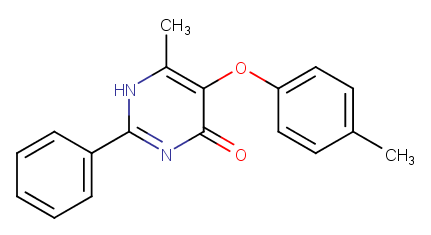

Supplement: RA-011-D1RA00914A-s1759 [file RA-011-D1RA00914A-s1759.png]

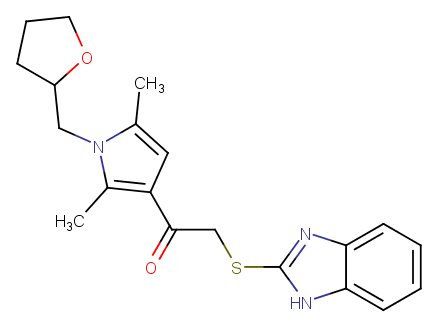

Supplement: RA-011-D1RA00914A-s1760 [file RA-011-D1RA00914A-s1760.png]

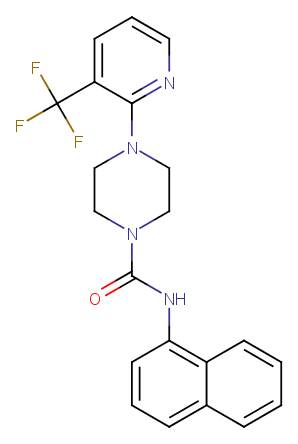

Supplement: RA-011-D1RA00914A-s1761 [file RA-011-D1RA00914A-s1761.png]

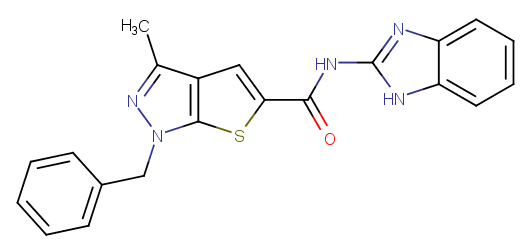

Supplement: RA-011-D1RA00914A-s1762 [file RA-011-D1RA00914A-s1762.png]

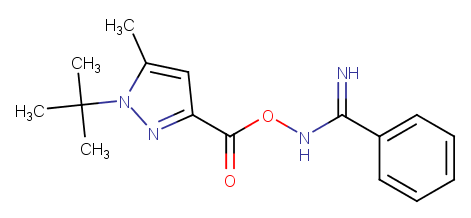

Supplement: RA-011-D1RA00914A-s1763 [file RA-011-D1RA00914A-s1763.png]

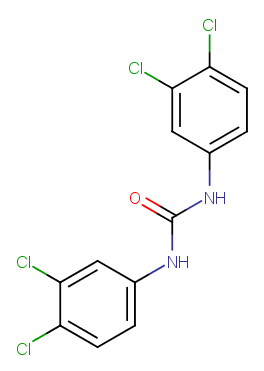

Supplement: RA-011-D1RA00914A-s1764 [file RA-011-D1RA00914A-s1764.png]

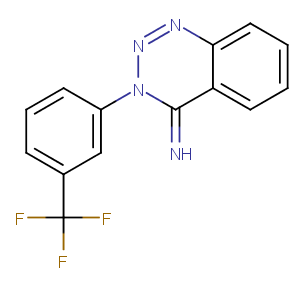

Supplement: RA-011-D1RA00914A-s1765 [file RA-011-D1RA00914A-s1765.png]

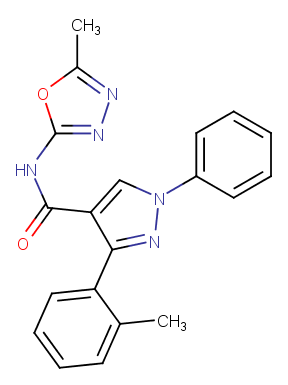

Supplement: RA-011-D1RA00914A-s1766 [file RA-011-D1RA00914A-s1766.png]

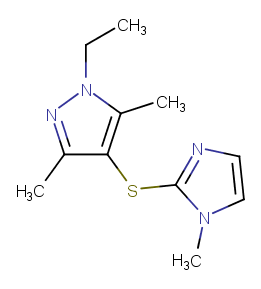

Supplement: RA-011-D1RA00914A-s1767 [file RA-011-D1RA00914A-s1767.png]
